# A small-molecule stabilizer of the calpastatin–calpain-2 complex restores mitochondrial function and mitigates neurodegeneration

**DOI:** 10.1126/sciadv.aeb1174

**Published:** 2026-03-27

**Authors:** Di Hu, Xiaoyan Sun, Yutong Shang, Kathleen Lundberg, Drew J. Adams, Xin Qi

**Affiliations:** ^1^ Department of Physiology and Biophysics, Case Western Reserve University School of Medicine, Cleveland, OH 44106, USA.; ^2^ Center for Mitochondrial Research and Therapeutics, Case Western Reserve University School of Medicine, Cleveland, OH 44106, USA.; ^3^ Proteomics Center, Case Western Reserve University School of Medicine, Cleveland, OH 44106, USA.; ^4^ Department of Genetics and Genome Sciences, Case Western Reserve University School of Medicine, Cleveland, OH 44106, USA.

## Abstract

Mitochondrial dysfunction and dysregulated proteolysis drive Huntington’s disease (HD), tauopathy, and related neurodegenerative disorders. Calpain-2, a Ca^2+^-activated protease restrained by calpastatin (CAST), is pathologically overactivated, yet no therapies directly target this axis. We identify A36, a brain-penetrant small molecule derived from CHIR99021 that selectively stabilizes the CAST–calpain-2 complex without inhibiting GSK3. A36 acts as a protein-protein interaction stabilizer, enhancing CAST–calpain-2 binding, preventing CAST degradation, and thereby limiting calpain-2 activation and mitochondrial damage. In patients with HD induced pluripotent stem cell–derived neurons and mutant mouse striatal neurons, A36 normalized mitochondrial morphology and membrane potential, reduced oxidative stress, and improved survival. In vivo, A36 displayed favorable pharmacokinetics and central nervous system exposure; treatment reduced striatal neurodegeneration, mutant huntingtin aggregation, and motor deficits in HD R6/2 mice, and lowered phosphorylated tau, neuroinflammation, and cognitive decline in tauopathy PS19 mice. These findings establish pharmacological stabilization of CAST–calpain-2 as a therapeutic strategy and position A36 as a mechanism-selective modulator with broad neurodegenerative disease potential.

## Teaser

Medicinal chemistry identified a small molecule offering translational potential for neurodegenerative diseases.

## INTRODUCTION

Mitochondrial impairment and dysregulated proteolysis are intersecting pathological mechanisms that underlie neurodegeneration in a wide range of disorders, including Huntington’s disease (HD), Alzheimer’s disease (AD), Parkinson’s disease (PD), and amyotrophic lateral sclerosis (ALS) (*1*, *2*). Neurons are especially vulnerable to these insults due to their high metabolic demand, calcium sensitivity, and reliance on intact mitochondrial dynamics and synaptic architecture (*3*). Early mitochondrial impairments, such as calcium dysregulation and disrupted fission-fusion balance, compromise energy metabolism and axonal transport, leading to synaptic loss, toxic protein accumulation, and neuronal degeneration (*3*).

Mitochondrial damage is frequently exacerbated by calpain-2 hyperactivation, a pathological process linked to excessive proteolysis, mitochondrial fragmentation, and neuronal degeneration. Calpain-2, a calcium-activated cysteine protease, plays a key role in modulating cytoskeletal integrity, synaptic plasticity, and protein homeostasis (*4*). Under normal conditions, calpain-2 activity is tightly regulated by its endogenous inhibitor, calpastatin (CAST) (*5*). However, in stressed or disease states, CAST is frequently down-regulated, leading to calpain-2 hyperactivation (*6*, *7*). This, in turn, promotes proteolytic cleavage of critical neuronal proteins involved in cytoskeletal stability (e.g., neurofilament-L and neurofilament-M) (*8*, *9*), synaptic transmission (e.g., PSD-95, SNAP25) (*10*, *11*), and mitochondrial function (*12*). Moreover, calpain-2 directly or indirectly drives phosphorylation and overactivation of the mitochondrial fission protein Drp1, resulting in excessive mitochondrial fragmentation and bioenergetic failure (*13*, *14*). In addition, calpain-2 activity contributes to the generation and aggregation of pathogenic proteins, including phosphorylated tau (*15*), α-synuclein (*16*), mutant huntingtin (mtHtt) (*17*), and misfolded TDP-43 (*18*) and SOD1 (*19*), thereby amplifying neurodegenerative cascades.

Despite the therapeutic potential of calpain inhibition, broad-spectrum calpain inhibitors have failed in clinical trials because of off-target toxicity and lack of isoform specificity (*20*). In contrast, genetic up-regulation of CAST has demonstrated consistent neuroprotective effects in multiple preclinical models, suppressing calpain-2–driven pathology without interfering with its physiological roles (*6*, *17*, *19*, *21*, *22*). These findings position pharmacologic CAST stabilization as a compelling strategy to modulate proteolytic stress and preserve mitochondrial integrity in neurodegenerative disease.

In previous work, we identified CHIR99021, a known glycogen synthase kinase 3 (GSK3) inhibitor, as a potent mitochondrial enhancer that reduces neurodegeneration in HD models by stabilizing CAST (*23*). Its protective effects were independent of GSK3 inhibition (*23*), pointing to an alternative CAST-dependent mechanism. However, the kinase inhibitory activity of CHIR99021 limits its translational utility due to potential off-target effects on key signaling pathways. To overcome this, we launched a focused medicinal chemistry campaign to develop CHIR99021 analogs that enhance CAST-stabilizing effects while eliminating GSK3 inhibition. This effort led to the identification of compound 82 (designed as A36), a brain-penetrant small molecule that selectively stabilizes the CAST–calpain-2 complex. Mechanistically, A36 functions as a protein-protein interaction (PPI) stabilizer, enhancing the CAST–calpain-2 interaction and protecting CAST from degradation. This prevents calpain-2 hyperactivation, suppresses Drp1-mediated mitochondrial fragmentation, and preserves mitochondrial function. In multiple disease-relevant models, including patient induced pluripotent stem cell (iPSC)–derived neurons and mouse models of HD and tauopathy, A36 demonstrated target engagement, restored mitochondrial integrity and function, reduced neuroinflammation and toxic protein accumulation, and improved animal behavioral outcomes.

These findings establish CAST stabilization as a mechanistically targeted and disease-relevant therapeutic approach. A36 represents a brain-penetrant PPI stabilizer of CAST–calpain-2 complex that addresses convergent drivers of neurodegeneration, offering translational potential for HD, tauopathies, and other neurodegenerative diseases marked by mitochondrial and proteolytic dysfunction.

## RESULTS

### Discovery of A36, a CAST stabilizer with mitochondrial protective activity

**Fig. 1.**
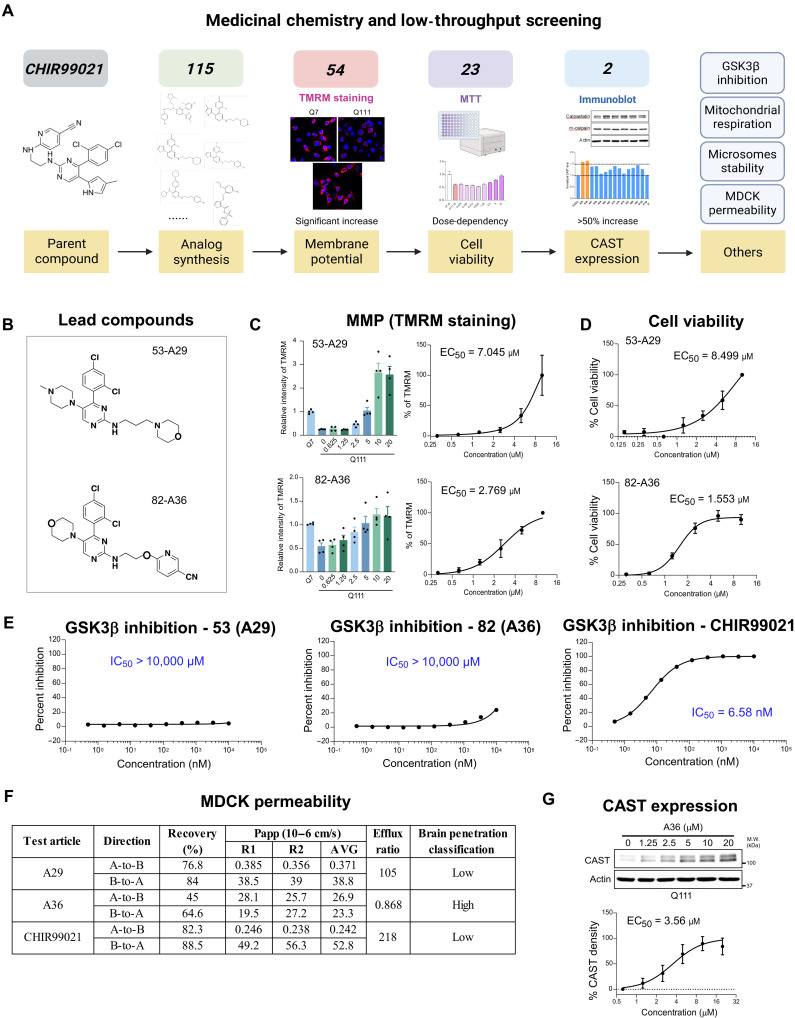
Medicinal chemistry optimization identifies A36 as a lead compound. (**A**) Schematic overview of the medicinal chemistry workflow and focused screening strategy used to identify optimized CHIR99021 analogs. (**B**) Chemical structures of lead compounds 53 (designated A29) and 82 (designated A36). (**C**) MMP measured by TMRM fluorescence in Q7 (HdhQ7) and Q111 (HdhQ111) mouse striatal cells treated with A29 or A36. Histograms depict fluorescence intensity; dose-response curves were used to calculate EC_50_. *n* = 4. (**D**) Cell viability assays in HdhQ111 cells following treatment with A29 or A36. EC_50_ values were derived from viability curves. *n* = 4. (**E**) In vitro GSK3β kinase inhibition assay showing IC_50_ for CHIR99021 (6.58 nM), A29 (>10,000 nM), and A36 (>10,000 nM). *n* = 2. (**F**) Summary of MDCK cell permeability for A29, A36, and CHIR99021. (**G**) Immunoblot analysis of CAST protein levels in Q111 cells treated with A36. A36 enhanced CAST expression in a dose-dependent manner; EC_50_ = 3.56 μM. *n* = 9. All quantitative data are presented as means ± SEM.

Our prior work demonstrated that CHIR99021 enhances mitochondrial membrane potential (MMP) and promotes the survival of striatal neuron–like cells carrying the HD genotype via a mechanism independent of its canonical GSK3 inhibition (*23*). Motivated by this observation, we initiated a medicinal chemistry campaign to develop CHIR99021 analogs that improve neuroprotective efficacy, eliminate GSK3 activity, and enhance central nervous system (CNS) drug–like properties (Fig. 1A). In addition, we sought to better understand the neuroprotective mechanism by which these molecules act. Note that analog synthesis procedure and quality control are shown in the supporting documents 1 and 2, respectively.

We began by synthesizing a panel of first-generation CHIR99021 analogs (table S1) and evaluated their effects on MMP and cell viability using TMRM (tetramethylrhodamine, methyl ester) and MTT (3-[4,5-dimethylthiazol-2-yl]-2,5 diphenyl tetrazolium bromide) assays, respectively, in HdhQ111 striatal neurons at a fixed concentration of 5 μM. HdhQ111 cells, which are immortalized striatal-like neurons derived from HD knock-in mice, are a well-characterized cellular model that exhibits mitochondrial damage and neurodegenerative features (*24*–*27*). This model was previously used in our high-throughput screen that identified CHIR99021 (*23*) and was used here for screening all analogs. Most analogs failed to demonstrate protective activity (fig. S1, A and B). The two compounds that retained protective effects (**7** and **11**) also retained potent GSK3 inhibition (table S1), indicating the need to further refine the scaffold.

To selectively abrogate GSK3 activity, we modified the aminopyridine moiety of CHIR99021, a known pharmacophore for GSK3 binding (*28*, *29*), replacing it with alternative heteroaryl and cycloalkyl groups (table S2). However, this second set of analogs showed little to no activity in either assay (fig. S1, C and D). We then modified compound **11**, which had shown protective efficacy (fig. S1, A and B), by altering its piperidine functionality (table S3). These analogs also lacked notable activity (fig. S1, E and F).

We next investigated additional aminopyridine replacements to abrogate GSK3 activity, this time with the 4-piperidyl aryl substitution rather than the 2,4-dichloro substituent (table S4). Compounds **41** and **46** emerged as active in both TMRM and MTT assays (fig. S1, G and H) and did not stabilize GSK3β in a cellular thermal shift assay (CETSA) (table S4), suggesting the desired disconnect from GSK3 engagement. Building on the scaffold of **41**, we synthesized a series of analogs varying the imidazole substituent while retaining the propylmorpholine side chain (table S5). Among these, compounds **53** and **57** demonstrated strong activity in mitochondrial and cell viability assays (fig. S2, A and B), with **53** showing no activity in both GSK3β CETSA and enzymatic assays (supporting file 3 and 4). We further diversified compound **53**, modifying the morpholine ring and adjacent substituents (table S6). While most changes abolished activity, certain analogs such as **64**, **66**, **67**, and **70** (table S6) retained moderate activity (fig. S2, C and D), again without GSK3 inhibition.

To identify additional high-potency, GSK3-sparing candidates, we tested alkoxy pyridine replacements, a modification previously shown to diminish GSK3 affinity (*30*). While the alkoxypyridine analog of CHIR99021 was inactive (compound **2**; table 1), analogs incorporating the alkoxypyridine scaffold and modified imidazoles yielded compounds **82** and **83**, both active in TMRM and MTT assays and devoid of GSK3β activity by CETSA and enzyme assays (fig. S2, E and F, and table S7). Structure-activity relationship (SAR) analysis revealed that even minor changes to the imidazole or side-chain groups abolished activity (table S7), indicating tight structure–activity constraints. This alkoxypyridine strategy was also used successfully in analogs containing the 4-piperidylaryl motif (e.g., **98**; table S8). A set of close-in analogs of **98** were also comparably active and were confirmed as negative in the GSK3β CETSA (e.g., **101** to **103**; table S8) (fig. S2, G and H). Notably, the *N*-methylpiperazine motif was essential for activity in the TMRM and MTT assays, as various replacements were inactive (**104** to **108**). However, the *N*-methyl substituent could be elaborated to isopropyl, and the pyridyl linkage could be modified to pyrimidine without loss of activity (e.g., **111** and **113**; table S8).

We previously reported that the mitochondrial and neuroprotective effects of CHIR99021 are mediated by its ability to elevate and stabilize CAST protein levels (*23*). We next evaluated whether these analogs similarly modulate CAST level. Western blot analysis of 24 active analogs revealed that 12 increased CAST protein expression, with compounds **53** and **82** (Fig. 1B) producing a robust ~1.5-fold up-regulation (Fig. 1B and fig. S3), comparable to CHIR99021 (*23*).

We prioritized compounds **53** and **82** for further dose-response evaluation. While compound **53** exhibited modest effects, compound **82** demonstrated potent mitochondrial protection with a median effective concentration (EC_50_) of ~2.77 μM (TMRM assay) and enhanced cell viability with an EC_50_ of ~1.55 μM (MTT assay) (Fig. 1, C and D). Both compounds showed minimal activity against GSK3 in kinase assay and CETSA (Fig. 1E and fig. S4A). To assess their pharmacological properties, we also evaluated compound **53** and **82** in in vitro models of blood-brain barrier permeability [Madin-Darby canine kidney cells transfected with the MDR1 gene (MDR1-MDCK)] and metabolic stability using mouse liver microsomes. Both compounds exhibited modest microsomal stability (fig. S4B). However, compound **82** showed a marked improvement in the MDR1-MDCK assay with an efflux ratio of 0.868 (Fig. 1F), indicating improved predicted CNS permeability. Compound **82** also up-regulated CAST protein level, but not mRNA level, in a dose-dependent manner, with an EC_50_ of ~3.55 μM (Fig. 1G and fig. S5A).

Together, these data identify compound **82**, hereafter designated as **A36**, as a promising lead that combines CAST stabilization, mitochondrial protection, absence of GSK3 inhibition, and favorable CNS drug–like properties. A36 was therefore selected for further in vitro and in vivo characterization.

### A36 treatment restores mitochondrial function and CAST expression in HD neuronal cells

**Fig. 2.**
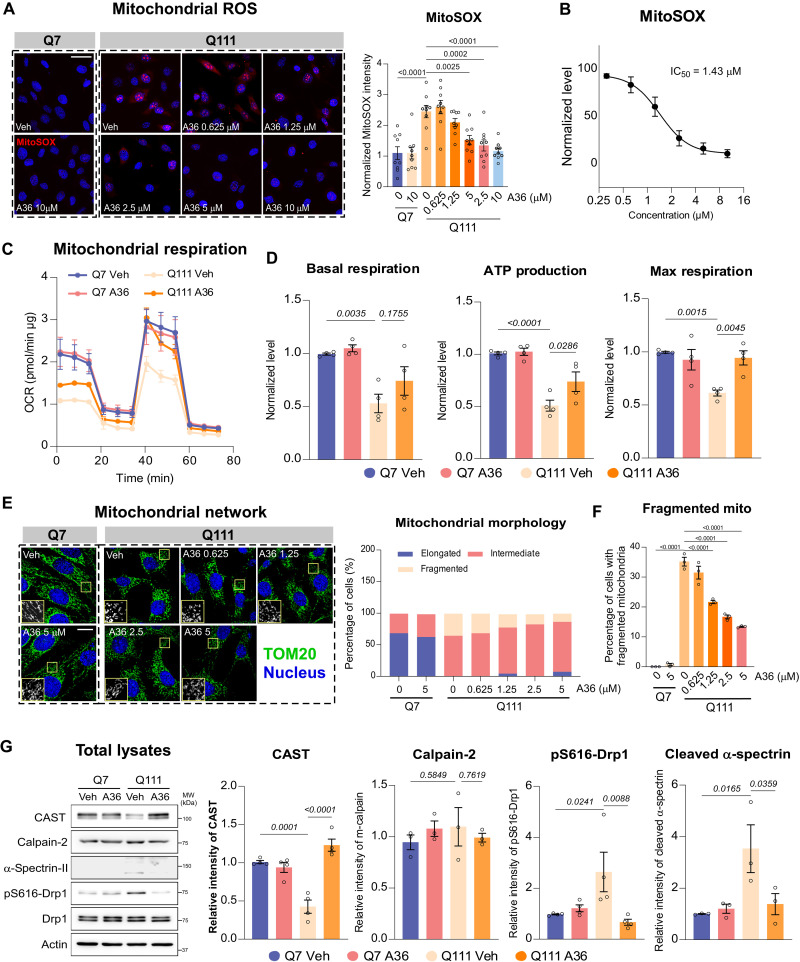
A36 treatment restores mitochondrial activity in HD striatal cells. (**A**) Representative images of MitoSOX fluorescence staining in Q7 (HdhQ7) and Q111 (HdhQ111) cells treated with dimethyl sulfoxide (DMSO) (Veh) or A36 at different concentrations. Quantification of MitoSOX intensity is shown in histogram. *n* = 9. Scale bar, 50 μm. (**B**) IC_50_ of A36 (1.43 μM) on mitochondrial ROS production is calculated. (**C**) Measurement of mitochondrial oxygen consumption rate (OCR) in Q7 and Q111 cells treated with Veh or A36 by Seahorse analyzer. *n* = 4. (**D**) Quantification of mitochondrial basal respiration, ATP production, and maximal respiration is shown in histogram. *n* = 4. (**E**) Representative images of TOM20 immunostaining in Q7 and Q111 cells treated with Veh or A36. Histograms summarize the percentage of cells with elongated, intermediate, or fragmented mitochondria. *n* = 3. Scale bar, 10 μm. (**F**) Histogram summarizes the percentage of cells with fragmented mitochondria. *n* = 3. (**G**) Representative blot of CAST, calpain-2, cleaved α-spectrin II, pS616-Drp1, and Drp1 in Veh- or A36-treated Q7 and Q111 cells. Quantification of the relative density is shown in histogram. *n* = 4. Quantitative data for all panels are presented as means ± SEM. Data are compared by one-way analysis of variance (ANOVA) with Tukey’s post hoc test.

To further evaluate the neuroprotective effects of compound A36 on mitochondrial function in HD, we assessed its impact on mitochondrial reactive oxygen species (ROS) production using HdhQ7 [wild-type (WT)] and HdhQ111 (HD mutant) striatal neurons. While A36 had minimal effect on mitochondrial ROS levels in HdhQ7 cells, it significantly reduced mitochondrial ROS in HdhQ111 cells in a dose-dependent manner, with a median inhibitory concentration of 1.43 μM as measured by MitoSOX fluorescence (Fig. 2, A and B). Consistently, A36 treatment enhanced mitochondrial respiration, increasing adenosine triphosphate (ATP) production and maximal respiratory capacity in HdhQ111 cells, whereas no significant effects were observed in HdhQ7 controls (Fig. 2, C and D). Given that mitochondrial dysfunction in HD is associated with excessive mitochondrial fission and network fragmentation (*24*, *25*), we next analyzed mitochondrial morphology via TOM20 (a mitochondrial marker) immunostaining in striatal neurons following A36 treatment. In line with prior reports, mitochondria in HdhQ111 neurons displayed predominantly fragmented or intermediate morphology compared to the tubular networks observed in HdhQ7 cells (Fig. 2E). A36 treatment significantly decreased the percentage of cells with fragmented mitochondria in a dose-dependent manner (Fig. 2, E and F), indicating normalization of mitochondrial dynamics.

Previously, we demonstrated that CHIR99021-mediated up-regulation of CAST attenuates Drp1-driven mitochondrial fragmentation in HD models (*23*). Consistently, A36 treatment significantly reduced Drp1 phosphorylation at Ser^616^ (p-Drp1 S616) (Fig. 2G) and decreased its mitochondrial translocation (fig. S5B), thereby normalizing mitochondrial dynamics. Moreover, A36 treatment restored CAST expression in HdhQ111 cells (Fig. 2G), supporting its role as a CAST stabilizer. CAST is an endogenous inhibitor of the cysteine protease calpain, which typically exists as heterodimers with either calpain-1 (μ-calpain) or calpain-2 (m-calpain). Given the established role of calpain-2 hyperactivation in HD-related neurodegeneration (*17*, *31*), we assessed calpain-2 activity following A36 treatment. A36 significantly suppressed calpain-2 activity, as evidenced by reduced cleavage of its substrate α-spectrin II, while total calpain-2 protein levels remained unchanged (Fig. 2G). Together, these findings suggest that A36 protects mitochondrial function in HD by stabilizing CAST, inhibiting pathological calpain-2 activation, and preventing Drp1-mediated mitochondrial fragmentation, which recapitulates the neuroprotective effects of its parent compound, CHIR99021.

### A36 acts as a PPI stabilizer of the CAST–calpain-2 complex

**Fig. 3.**
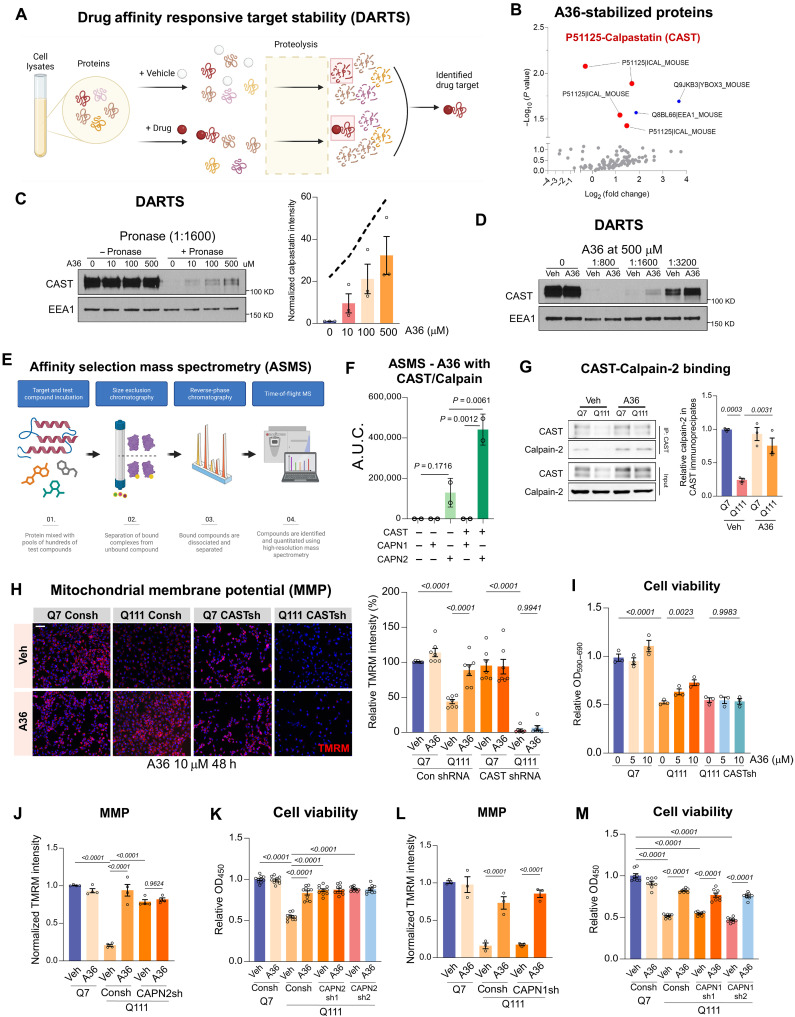
A36 targets CAST and stabilizes the CAST–calpain-2 interaction. (**A** and **B**) DARTS workflow and volcano plot showing peptides protected from Pronase digestion by A36; significantly enriched (≥twofold, *P* < 0.05) peptides include CAST (P51125, red) and EEA1 (Q8BL77, blue). (**C** and **D**) DARTS immunoblots from Q111 (HdhQ111) lysates showing A36-dependent Pronase protection of CAST, but not EEA1 (*n* = 4). (**E**) ASMS workflow for detecting direct compound–protein interactions. (**F**) ASMS of recombinant proteins showing selective binding of A36 to CAST–calpain-2, but not CAST–calpain-1, complexes (means ± SD). A.U.C., area under the curve. *n* = 2. (**G**) Coimmunoprecipitation of calpain-2 from Q7 (HdhQ7) and Q111 cells treated with A36 using an anti-CAST antibody. Immunoblot and quantification of coprecipitated calpain-2 are shown. *n* = 3. (**H**) Representative TMRM fluorescence staining images and quantification of MMP in control (Con shRNA, Consh) or CAST knockdown (CAST shRNA, CASTsh) Q7 and Q111 cells treated with DMSO or A36. *n* = 7. Scale bar, 50 μm. (**I**) Cell viability measured by MTT assay in CASTsh-expressing Q7 and Q111 cells treated with vehicle or A36. *n* = 3. (**J** and **K**) TMRM fluorescence intensity (*n* = 4) and cell viability (*n* = 10) in Consh or calpain-2 knockdown (CAPN2 sh1 or sh2) HdhQ111 cells with or without A36. (**L** and **M**) TMRM intensity (*n* = 3) and cell viability (*n* = 8) in Consh or calpain-1 knockdown (CAPN1 sh1 or sh2) HdhQ111 cells with or without A36 treatment. Quantitative data for all panels [except (F)] are presented as means ± SEM. Data are compared by one-way ANOVA with Tukey’s post hoc test. h, hours.

To further elucidate the mechanism by which A36 confers mitochondrial and neuronal protection, we sought to identify its direct molecular target. We used drug affinity responsive target stability (DARTS) assay, a biochemical approach that leverages the principle that ligand binding enhances protein stability by shielding it from proteolytic degradation (Fig. 3A). Using this method, HdhQ111 mouse striatal cell lysates were incubated with A36 or vehicle, followed by limited protease digestion (Pronase), and analyzed by unbiased label-free liquid chromatography–tandem mass spectrometry (LC-MS/MS). LC-MS/MS analysis identified five peptides that were significantly more resistant to proteolysis following A36 treatment (fold change >2, *P* < 0.05), suggesting potential interactions (Fig. 3B). Notably, three of these peptides were derived from CAST, indicating that A36 may directly bind and stabilize CAST (Fig. 3B). To validate this interaction, we performed DARTS–Western blot assays using HdhQ111 lysates treated with increasing concentrations of A36. A36 dose-dependently increased the stability of CAST, while EEA1, another protein identified in the LC-MS/MS, showed no change, suggesting that the interaction with CAST is selective (Fig. 3, B and C). In addition, we observed that CAST stabilization by A36 was dependent on Pronase concentration: At intermediate concentration of Pronase, A36 protected CAST from degradation compared to vehicle, whereas CAST was fully degraded at high Pronase concentrations and partially retained in both groups at low concentrations. This pattern is consistent with our hypothesis that CAST is likely to be a direct target of A36 (Fig. 3D).

Calpain proteases play opposing roles in neuronal homeostasis, with calpain-1 supporting synaptic integrity and neuroprotection, whereas calpain-2 drives neurodegeneration. CAST, an endogenous inhibitor of calpain activity, forms regulatory complexes with both isoforms to modulate their proteolytic activity and localization. To determine whether A36 influences these protein complexes, we used Affinity Selection Mass Spectrometry (ASMS), a label-free, high-throughput technique designed to identify ligand-target interactions within protein complexes (Fig. 3E). ASMS allows for the quantitative assessment of small-molecule binding affinities, making it well-suited for studying dynamic PPI such as those involving CAST and calpain isoforms. Recombinant CAST, calpain-1, and calpain-2 proteins were incubated with A36, followed by ASMS analysis. A36 showed no detectable binding to calpain-1 or the CAST–calpain-1 complex. In contrast, it bound to calpain-2 alone and even more remarkably to the CAST–calpain-2 complex (Fig. 3F). These results suggest that A36 selectively stabilizes and enhances the CAST–calpain-2 complex. Consistent with the ASMS findings, immunoprecipitation from HdhQ111 cells revealed that A36 treatment enhanced the interaction between endogenous CAST and calpain-2 (Fig. 3G), but not CAST and calpain-1 (fig. S5C). However, A36 had no observed effects in HdhQ7 WT cells. Together, these data indicate that A36 acts selectively on the CAST–calpain-2 axis rather than broadly affecting calpain regulation.

To establish whether A36-mediated mitochondrial enhancement and neuroprotection require CAST and calpain-2, we examined its effects in CAST- or calpain-2–deficient neuronal cells. A36 treatment significantly improved MMP and cell viability in HdhQ111 cells, but this effect was abolished in CAST short hairpin RNA (shRNA)–expressing HdhQ111 cells (Fig. 3, H and I). Similarly, knockdown of calpain-2 with two independent shRNAs abolished A36-mediated mitochondrial protection and neuronal survival (Fig. 3, J and K). However, knockdown of calpain-1 had no effect on A36 neuroprotective properties (Fig. 3, L and M). Thus, A36 requires both CAST and calpain-2 to exert its protective activity, and calpain-1 is not involved in this mechanism, consistent with the selective effect on CAST–calpain-2 binding.

Given that both CAST and calpain-2 exhibit intrinsically disordered and flexible structural elements, we propose that A36 functions as a PPI stabilizer to stabilize a key interface within the CAST–calpain-2 complex. By reinforcing this interaction, A36 may simultaneously enhance CAST stability and promote its interaction with calpain-2, preventing calpain-2 aberrant activation. As a result, A36 preserves mitochondrial integrity and supports neuronal survival in diseased cells. This chemical biology–driven approach suggests a previously unidentified therapeutic strategy for targeting proteolytic dysregulation in neurodegenerative diseases through selective stabilization of disease-relevant protein complexes.

### A36 treatment restores neuronal function and CAST expression in MSNs derived from iPSCs of patients with HD

**Fig. 4.**
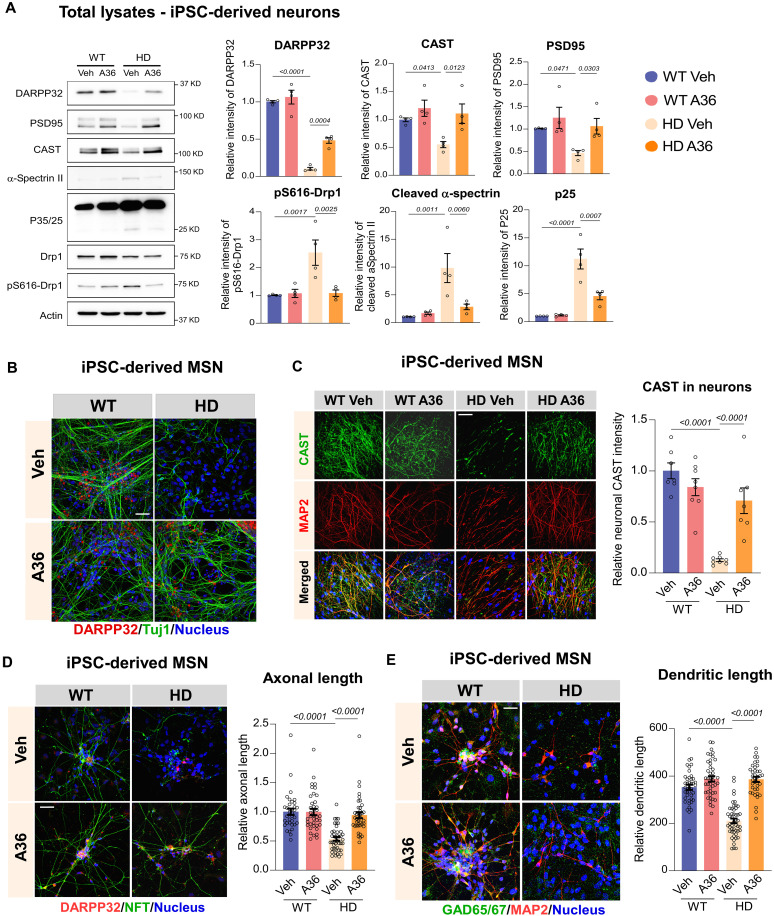
A36 treatment is protective in MSN derived from iPSC of patients with HD. After 25-day differentiation, the patient iPSCs-derived neurons were treated with DMSO (Veh) or A36 at 1 μM for five consecutive days and subjected to the following biochemical analyses. (**A**) Total protein lysates were harvested from neurons and subjected to Western blot analyses. Shown are the representative blots. Quantification of the density of indicated proteins is shown in histogram. *n* = 4 batches of differentiation. (**B**) Representative images of DARPP32 and Tuj1 staining in the Veh- or A36-treated neurons derived from WT or iPSCs from patients with HD. *n* = 4 batches of differentiation; scale bar, 50 μm. (**C**) Representative images of CAST and MAP2 staining in WT and neurons of patients with HD treated with Veh or A36. Quantification of the neuronal CAST intensity is shown in histogram. *n* = 7 to 8 areas per group; scale bar, 50 μm. (**D**) Representative images of DARPP32 and neurofilament (NFT) staining in WT and neurons of patients with HD treated with Veh or A36. Quantification of the neuronal axonal length is shown in histogram. *n* = at least 30 neurons per group; scale bar, 20 μm. (**E**) Representative images of GAD65/67 and MAP2 staining in WT and neurons of patients with HD treated with Veh or A36. Quantification of neuronal dendritic length is shown in histograms. *n* = at least 30 neurons per group; scale bar, 20 μm. Quantitative data for all panels are presented as means ± SEM. Data are compared by one-way ANOVA with Tukey’s post hoc test.

HD is characterized by the selective degeneration of striatal GABAergic medium spiny neurons (MSNs) (*32*). In our previous work, we established an iPSC model derived from patients with HD by differentiating iPSCs harboring pathogenic CAG expansions in the Htt gene into GABAergic MSNs (HD-MSNs) (*23*, *24*). These MSNs from patients with HD recapitulated key disease features, including shortened neuritic processes, heightened vulnerability to stress, mitochondrial depolarization and fragmentation, and progressive cell loss. This model provides a patient-relevant platform to evaluate small molecules that modulate mitochondrial function and neuronal survival. To assess the therapeutic potential of A36, we treated HD-MSNs with 1 μM A36 for five consecutive days and analyzed neuronal and mitochondrial markers by Western blotting and immunocytochemistry. Compared to control MSNs, HD-MSNs exhibited marked reductions in DARPP32 and PSD95, established markers of MSN identity and synaptic density, indicative of neuronal degeneration and synaptic deterioration (Fig. 4A). These deficits were accompanied by elevated levels of p-Drp1 S616, consistent with CAST down-regulation and calpain-2 hyperactivation (Fig. 4A). Notably, A36 treatment significantly restored expression of DARPP32 and PSD95 in HD-MSNs while reducing p-Drp1^S616^ levels (Fig. 4, A and B). This restoration was accompanied by increased CAST protein expression and suppression of calpain-2 activity (Fig. 4, A and C), supporting the proposed mechanism by which A36 stabilizes CAST and the CAST–calpain-2 complex and inhibits pathological mitochondrial fragmentation. Furthermore, A36 significantly rescued neurite outgrowth in HD-MSNs, restoring both axonal length (labeled with antineurofilament) and dendritic length (labeled with anti-MAP2) to near-control levels (Fig. 4, D and E). Together, these findings demonstrate that A36 exerts robust neuroprotective effects in HD-MSNs by restoring CAST expression, inhibiting calpain-2 activity and mitochondrial dysfunction, and preserving neuronal integrity.

### A36 treatment mitigates HD-associated neuropathology and motor deficits in HD R6/2 mice

**Fig. 5.**
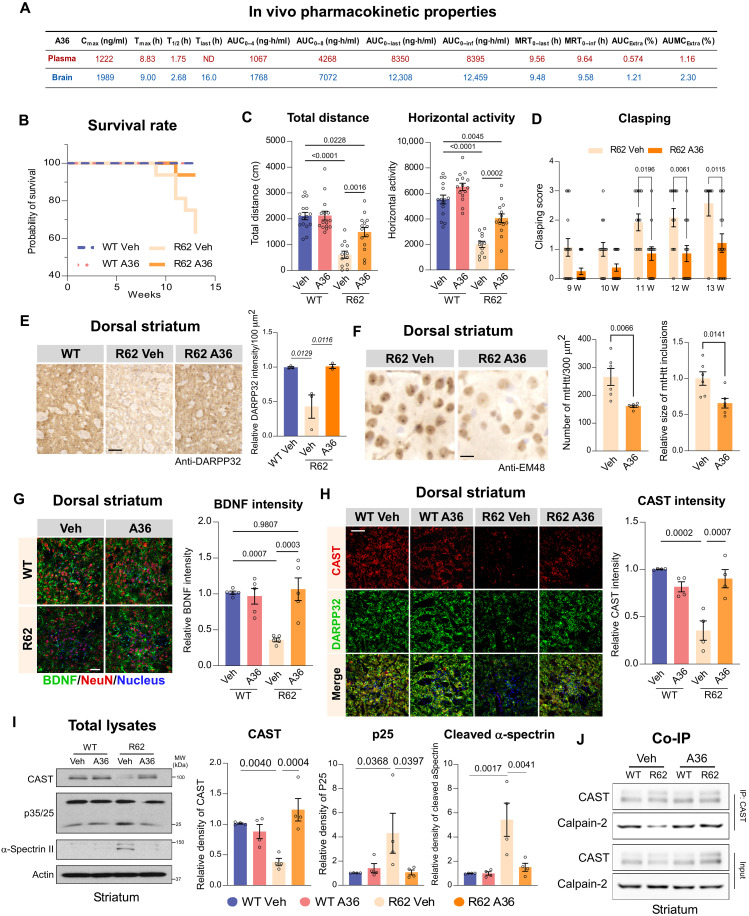
A36 treatment mitigates neuropathology and motor deficits in HD R6/2 mouse model. (**A**) Pharmacokinetics of A36 in plasma and brain of WT mice after three intraperitoneal injections (10 mg/kg, 4-hour intervals). (**B**) Survival of vehicle (Veh)– or A36-treated WT and R6/2 mice from 6 to 12 weeks (Log-rank test, two-tailed). *n* = 10 to 15 mice per group. (**C**) Open-field total distance travel and horizontal activity in WT and R6/2 mice. *n* = 10 to 15 mice per group. (**D**) Hindlimb clasping scores in Veh- or A36-treated R6/2 mice from 9 to 12 weeks (W). (**E**) Representative images of DARRP32 staining on brain slides from the R6/2 and WT mice. Quantification of striatal DARPP32 intensity per 100 μm^2^. *n* = 3 mice per group; scale bar, 100 μm. (**F**) EM48 staining of mtHtt aggregates and quantification of aggregate number per 300 μm^2^. *n* = 6 mice per group; scale bar, 10 μm. (**G**) Representative images of BDNF and NeuN staining on the brain slides from the R6/2 and WT mice treated with Veh or A36. Quantification of the intensity of BDNF is shown in histogram. *n* = 5 mice per group; scale bar, 20 μm. (**H**) DARPP32/CAST staining and quantification of CAST intensity in MSNs. *n* = 4 mice per group; scale bar, 20 μm. (**I**) Immunoblot analysis of striatal lysates and quantification of indicated proteins. *n* = 4 mice per group. (**J**) Coimmunoprecipitation of CAST from striatal lysates followed by immunoblotting (*n* = 3); quantification of CAST-calpain binding is shown in fig. S6I. Quantitative data for all panels are presented as means ± SEM. Data are compared by one-way ANOVA with Tukey’s post hoc test in (C), (E), (G), (H), and (I), and unpaired Student’s *t* test in (D) and (F).

We next evaluated the in vivo efficacy of A36 using an HD mouse model. We used R6/2 transgenic mice, which express a fragment of the human mtHtt protein and rapidly develop HD-associated pathologies, including mtHtt accumulation, striatal degeneration, and motor deficits (*33*). This model is widely used as a primary screening tool for HD drug candidates. Before efficacy testing, we characterized the pharmacokinetic properties of A36 in vivo. WT mice received three intraperitoneal injections of A36 at 4-hour intervals, after which concentrations of plasma and brain samples harvested at various time points were quantified by mass spectrometry. Consistent with in vitro MDR1-MDCK assay results indicating a low efflux ratio, A36 demonstrated efficient brain penetration and favorable blood-brain barrier permeability. Notably, A36 exhibited a longer duration of brain exposure, supporting its suitability for CNS-targeted therapy (Fig. 5A).

Next, we administered A36 to both HD R6/2 mice and their WT littermates via intraperitoneal injection at 10 mg kg^−1^ day^−1^, 5 days per week, from 6 to 12 weeks of age (fig. S6A). A36 treatment significantly extended the life span of R6/2 mice, reducing mortality by 30% at 12 weeks of age (Fig. 5B), although it had only a mild effect on body weight (fig. S6B). Compared to their WT littermates, R6/2 mice exhibited severe motor deficits, as evidenced by a significant reduction in total travel distance and horizontal activity in the open field test, as well as frequent hindlimb clasping behavior (Fig. 5, C and D). A36 treatment rescued both locomotor activity and clasping behavior, suggesting protective effects on motor function (Fig. 5, C and D). This improvement in motor activity was associated with the mitigation of mtHtt-related neuropathology. Specifically, A36 treatment significantly increased the immunodensity of DARPP32, a marker of MSN (*34*), and reduced mtHtt aggregates in the dorsal striatum of R6/2 mice (Fig. 5, E and F, and fig. S6, C and D). In addition, A36 restored brain-derived neurotrophic factor (BDNF) levels and reduced microglial activation, both of which are dysregulated in HD pathology (Fig. 5G and fig. S6, E and F).

The loss of oligodendrocytes and subsequent demyelination have been increasingly recognized as key contributors to HD pathogenesis, exacerbating neuronal degeneration. A36 treatment significantly increased the number of CC1^+^ mature oligodendrocytes and the intensity of myelin basic protein^+^ myelin in the corpus callosum in R6/2 mice, both of which were notably diminished in HD mice compared to WT littermates (fig. S6, G and H).

To validate the on-target effects of A36, we then examined the interaction between CAST and calpain-2. R6/2 mice exhibited concomitant CAST down-regulation and increased cleavage of calpain-2 substrates, such as α-spectrin and p35, in the dorsal striatum compared to WT controls (Fig. 5, H and I). A36 treatment significantly restored CAST expression and reduced calpain-2 substrate cleavage (Fig. 5, H and I). Consistently, the CAST–calpain-2 interaction was disrupted in R6/2 mice but was restored upon A36 treatment (Fig. 5J and fig.S6I). This dosing regimen was well-tolerated; A36 treatment had no adverse effects on behavior, body weight, or survival rates in WT mice over the 6-week treatment period (Fig. 5 and fig. S6). Together, these findings demonstrate the protective effects of A36 treatment in the HD R6/2 mouse model.

### A36 treatment mitigates tauopathy in PS19 tau mice

**Fig. 6.**
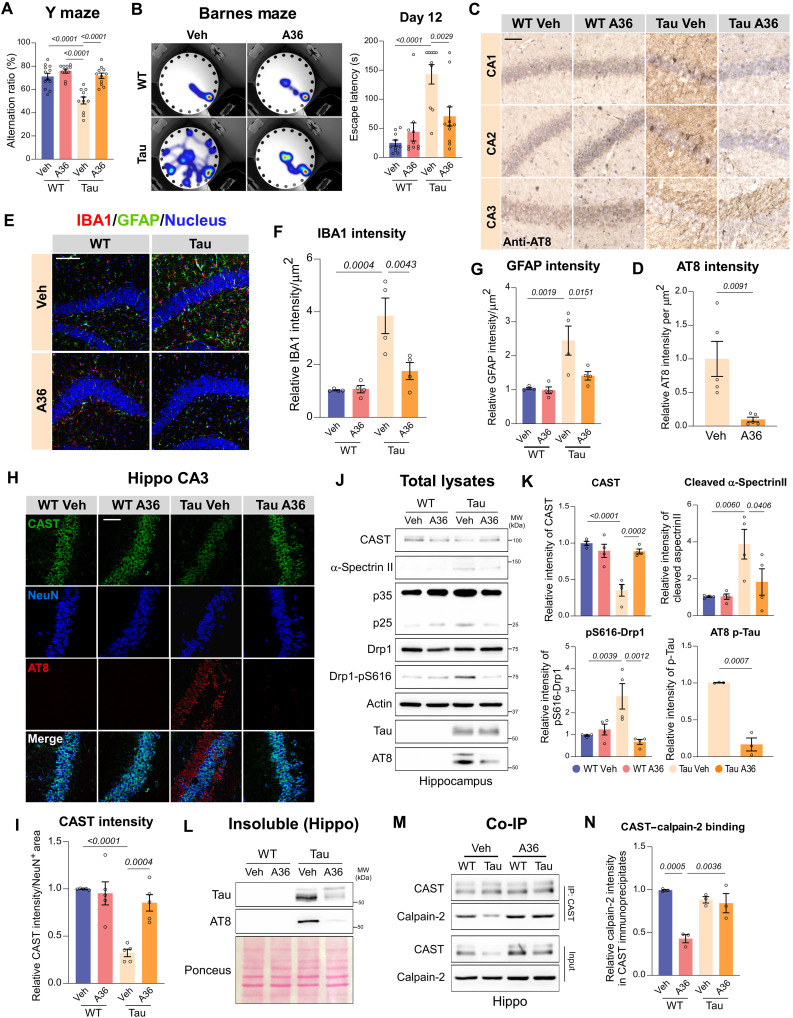
A36 treatment mitigates tauopathy and cognitive deficits in the PS19 mouse model. (**A**) Y-maze alternation ratios in 9-month-old PS19 (tau) and WT littermates. *n* = 12 mice per group. (**B**) Barnes maze performance of 9-month-old PS19 and WT mice on days 5 (fig. S7B) and 12 after 3-day training; representative 180-s heatmaps and escape latency (days 1 to 3). *n* = 10 to 12 mice per group. (**C** and **D**) Representative images of AT8-phosphorylated tau (p-Tau) staining in hippocampus of PS19 and WT mice treated with vehicle (Veh) or A36, and quantification of p-tau intensity. Scale bar, 50 μm. *n* = 5 mice per group. (**E** to **G**) IBA1 and GFAP immunostaining and quantification of IBA1 and GFAP intensity. *n* = 4 mice per group; scale bar, 100 μm. (**H** and **I**) Representative images of CAST/NeuN/p-Tau triple staining and quantification of neuronal CAST intensity in CA3. Scale bar, 50 μm. *n* = 5 mice per group. (**J** and **K**) Immunoblot analysis of hippocampal lysates and quantification of indicated proteins. *n* = 4 mice per group. (**L**) Triton insoluble hippocampal fractions were extracted using 2% SDS and subjected to Western blot analysis. Shown are the representative blots. Quantification of soluble tau, insoluble p-tau, and insoluble tau was shown in fig. S7 (C and D). (**M** and **N**) Coimmunoprecipitation of CAST from hippocampal lysates and quantification of calpain-2 in CAST immunoprecipitates. *n* = 3 mice per group. Quantitative data for all panels are presented as means ± SEM. Data are compared by one-way ANOVA with Tukey’s post hoc test in (A), (B), (F), (G), (K), (I), and (N), and unpaired Student’s *t* test in (D).

Hyperactivation of calpain-2 and CAST down-regulation are pathological markers of various neurological disorders, including neurodegenerative diseases such as AD, PD, HD, and ALS. Conversely, genetic up-regulation of CAST, leading to calpain-2 suppression, has been shown to effectively mitigate neuropathology in preclinical models of neurodegenerative diseases. This suggests that CAST-mediated inhibition of calpain-2 may serve as a common therapeutic strategy for a spectrum of neurodegenerative conditions. Therefore, we sought to determine whether A36 could serve as a broad-spectrum treatment by evaluating its protective effects in the PS19 transgenic mice, which recapitulate tauopathy and AD pathology. The PS19 transgenic mice express the mutant P301S human tau protein under the control of the mouse prion protein promoter, leading to progressive neuronal loss and brain atrophy, particularly in the hippocampus (*35*). These mice develop widespread neurofibrillary tangle–like inclusions in the brain, accompanied by neuroinflammation. Behaviorally, PS19 mice exhibit age-associated cognitive impairments, including selective deficits in spatial learning and memory (*35*). Using the same treatment regimen as we did in HD mice, we administered A36 at 10 mg kg^−1^ day^−1^, 5 days per week, to PS19 mice and their WT littermates from four to 9 months of age (fig. S7A). Four months of A36 treatment significantly improved both short-term and long-term memory in PS19 mice, as demonstrated by enhanced performance in the spontaneous alternation test and reduced escape latency in the Y maze and Barnes maze tests, respectively (Fig. 6, A and B, and fig. S7B).

PS19 mice exhibited widespread hippocampal expression of phosphorylated tau, specifically labeled by the anti-AT8 antibody, compared to WT controls (Fig. 6, C and D). This pathological tau accumulation was significantly diminished upon A36 treatment (Fig. 6, C and D). In addition, A36 mitigated neuroinflammation in PS19 mice, as evidenced by a significant reduction in glial fibrillary acidic protein (GFAP) and IBA1 immunodensity, markers of astrocytes and microglia, respectively, within the hippocampus (Fig. 6, E to G). In relation to the CAST–calpain-2 signaling pathway, both immunostaining and Western blot analysis revealed a significant decrease in CAST protein levels in the PS19 mouse hippocampus (Fig. 6, H to K). This reduction was accompanied by increased levels of p25, cleaved α-spectrin II, and p-Drp1 S616 (Fig. 6, J and K). Notably, A36 treatment restored the expression of these proteins and reduced phosphorylated tau (AT8-positive) in both the detergent-soluble and insoluble fractions while eliminating insoluble tau aggregates (Fig. 6, J to L, and fig. S7, C and D). Last, we observed a weakened interaction between CAST and calpain-2 in PS19 mice compared to their WT littermates, which was enhanced following A36 treatment (Fig. 6, M and N). These findings suggest that A36 treatment mitigates tau-related pathology and cognitive deficits by stabilizing the CAST–calpain-2 complex and restoring CAST level, thereby preventing pathological calpain-2 hyperactivation.

## DISCUSSION

**Fig. 7.**
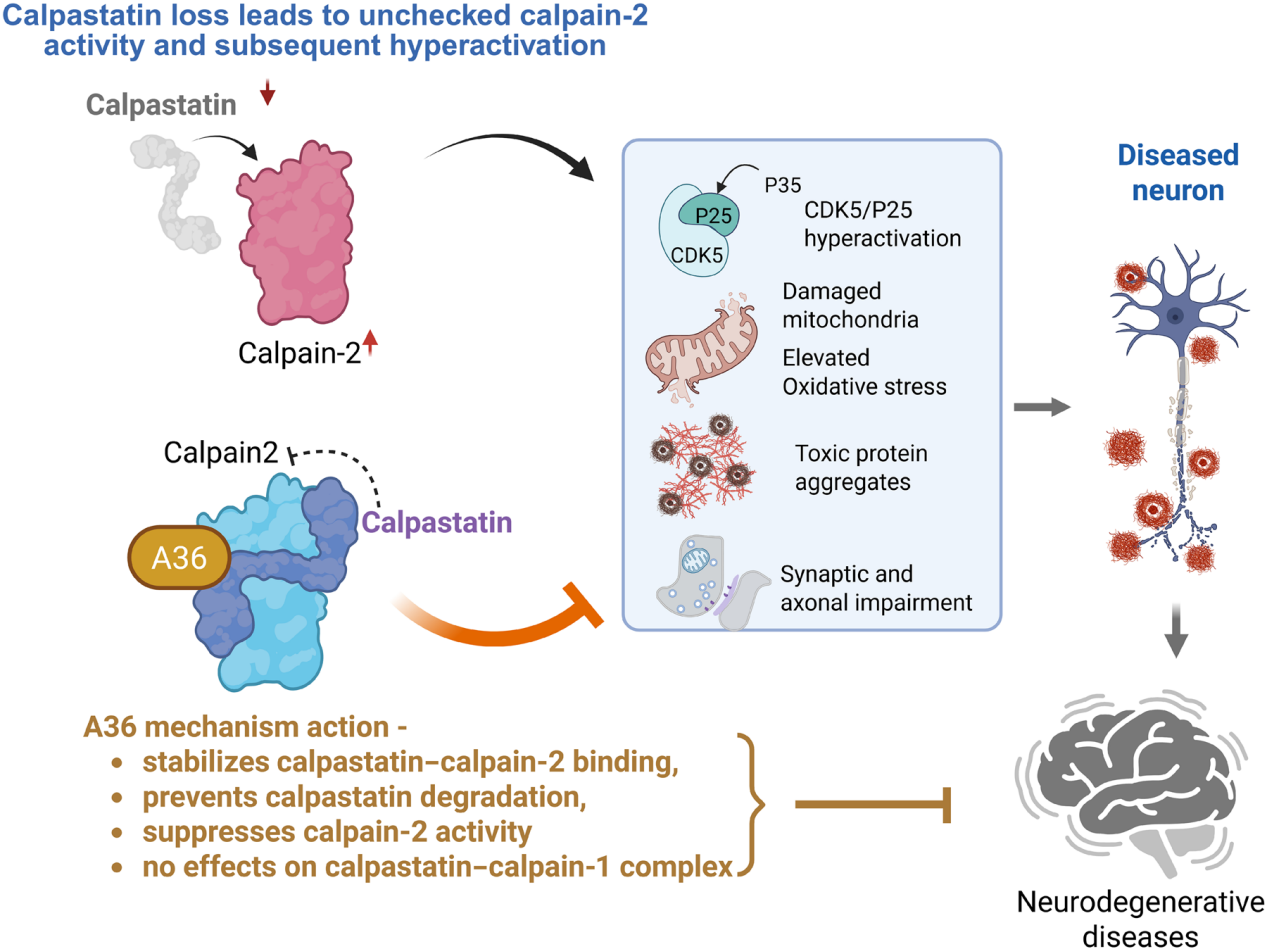
Schematic summary of the findings in this study. A36 selectively stabilizes CAST–calpain-2 binding, preventing CAST degradation, limiting CDK5-dependent mitochondrial damage and oxidative stress, and thereby reducing protein aggregation and neuropathology.

This study identifies A36, a small-molecule stabilizer of the CAST–calpain-2 complex, as a promising therapeutic candidate for neurodegenerative diseases (Fig. 7). Mitochondrial dysfunction and calpain-2 hyperactivation are key drivers of neurodegeneration, yet direct pharmacological modulation of these pathways remains challenging. Through medicinal chemistry optimization of CHIR99021, a previously identified mitochondrial enhancer, we developed A36 for its high CNS penetration and selective stabilization of CAST, effectively preventing calpain-2–mediated mitochondrial fragmentation and proteolytic dysregulation. Mechanistic studies demonstrate that A36 functions as a PPI stabilizer of the CAST–calpain-2 interaction to prevent CAST degradation and suppress pathological calpain-2 activity. The neuroprotective effects of A36, validated in in vitro and in vivo models of HD and tauopathies, establish small-molecule stabilization of the CAST–calpain-2 complex as a useful strategy for restoring mitochondrial function and proteolytic balance in neurodegeneration. Our study thus positions A36 as a promising lead for therapeutic development in HD, tauopathies, and other neurological disorders in which mitochondrial damage and calpains activation are featured.

We propose that dysregulation of CAST leads to calpain overactivation, which in turn promotes mitochondrial fragmentation and excessive ROS production, thereby exacerbating the aggregation of multiple prion-like proteins in the brain. Both calpain-2 and CAST have been documented to localize on mitochondria (*36*, *37*). While mitochondrial accumulation of calpain-2 induces mitochondrial depolarization, oxidative stress, and energy depletion, overexpression of CAST mitigates mitochondrial damage caused by calpain in response to various stressors (*36*, *37*). Mitochondrial health relies on a precise balance between fusion and fission processes (*38*); however, excessive fission and mitochondrial fragmentation lead to mitochondrial oxidative stress and bioenergetic failure (*39*), which contribute to neurodegenerative diseases such as HD and AD. In HD, CAST degradation permits calpain-dependent cleavage of p35 to p25, resulting in aberrant activation of CDK5, p-Drp1 S616, and consequent mitochondrial fragmentation (*23*). The ensuing mitochondrial oxidative stress directly accelerates mtHtt aggregation (*40*) and indirectly impairs proteasome function and autophagic clearance (*41*–*45*), together driving rapid accumulation of mtHtt aggregates. Consistent with this model, treatment with A36 markedly reduces mitochondrial ROS and mtHtt burden, likely by modulating the CAST–calpain–CDK5–Drp1 signaling axis. Calpain overactivation has also been implicated in tauopathy, which precedes synaptic degeneration and neuronal loss in AD (*46*–*48*). Pathological calpain activity can generate a spectrum of toxic tau species through CDK5 activation (*49*, *50*). Furthermore, activated calpain can directly cleave and activate Drp1, exacerbating mitochondrial fragmentation and neuronal damage in AD (*13*). These suggest that A36 may act through the same signaling axis to preserve mitochondrial function and thereby limit tau aggregation. In addition, calpain may directly cleave mtHtt and tau into aggregation-prone truncated fragments, further contributing to proteostasis collapse following CAST loss (*17*, *31*, *51*–*53*). Determining whether the protective effects of A36 strictly depend on mitochondrial enhancement will be crucial to establish mitochondrial damage as a central driver of neurodegenerative pathology.

Our data, together with previous reports, indicate a predominant role for calpain-2–mediated down-regulation of CAST in HD. CAST, the endogenous inhibitor of calpains, is itself a physiological substrate for calpains 1 and 2 and undergoes limited proteolysis when calpain activity is elevated (*54*, *55*). Calpains cleave CAST within its inhibitory domains to generate smaller fragments with markedly reduced inhibitory potency, effectively converting CAST into a “suicide substrate” under conditions of sustained calpain activation (*56*–*60*). Across multiple stress paradigms, the extent of CAST degradation closely correlates with calpain activation, indicating that calpain-mediated CAST cleavage functions as a feed-forward amplifier for protease activity (*21*, *47*, *61*). Notably, calpain-2 overactivation is linked to neuronal injury in neurodegenerative conditions including HD, and calpain-2 knockdown confers neuroprotection in HD neurons. In our models, the interaction between calpain-2 and CAST is significantly reduced, consistent with enhanced calpain-2–mediated cleavage as a major cause of CAST depletion. The finding that A36 selectively stabilizes the CAST–calpain-2 complex, but not the CAST–calpain-1 complex, further supports this model. However, we cannot exclude the possibility that additional proteases may also contribute to CAST degradation, which remains an area for future investigation. Together, these observations highlight CAST degradation—particularly via calpain-2—as a key driver of dysregulated calpain signaling in HD and as a promising target for therapeutic intervention.

Identifying shared molecular hubs with essential pathogenic impact may provide insight into developing common intervention strategies for a broad spectrum of neurodegenerative diseases. Calpain-2, a cysteine protease primarily found in neurons, is typically maintained in an inactive precursor state and activated by micromolar calcium levels (*62*, *63*). Calpain-2 activation is associated with the cleavage and aggregation of prion-like proteins, which play a pivotal role in the pathogenesis of neurodegenerative diseases, including AD, HD, PD, and ALS. Under physiological conditions, calpain-2 activity is tightly regulated by its endogenous inhibitor, CAST, which limits cellular proteolysis. However, directly inhibiting calpain has proven challenging because of poor specificity and potential toxicity. An alternative approach is the selective suppression of calpain-2 activity via CAST up-regulation. To our knowledge, genetic up-regulation of CAST is one of the few universal strategies that has demonstrated beneficial effects in animal models of neurodegeneration. For instance, CAST overexpression in fly and mouse HD models ameliorated HD-associated neuropathology and behavioral deficits (*17*). In PS19 mice, CAST knock-in suppressed calpain-mediated tau cleavage, reducing tauopathy and cognitive deficits (*64*). Neuron-specific CAST overexpression inhibited the calpain-CDK5 signaling axis, thereby reducing the production of toxic SOD1 oligomers in the SOD1 G93A mouse model of ALS (*19*). Moreover, CAST overexpression mitigated calpain-mediated α-synuclein cleavage and aggregation, astrogliosis, and synaptic impairment in the A30P SNCA mouse model of PD (*21*). Collectively, these findings suggest that CAST up-regulation may represent a common therapeutic strategy for neurodegenerative diseases. Our finding that A36 selectively stabilizes the CAST–calpain-2 complex, rather than directly inhibiting the calpain catalytic site, highlights a mechanistically distinct strategy compared with classical calpain inhibitors. Conventional calpain inhibitors, including peptidic and peptidomimetic compounds, often lack isoform selectivity, interact with other cysteine proteases, and globally suppress calpain activity, raising concerns about interference with essential physiological processes such as synaptic plasticity, cytoskeletal remodeling, and cell survival signaling (*4*, *65*–*67*). By contrast, A36 appears to function as a context-dependent modulator that reinforces the endogenous brake provided by CAST on calpain-2, thereby attenuating pathological overactivation while potentially preserving a basal level of physiological protease activity. This “interface-stabilizing” mode of action, which is conceptually closer to a molecular glue than a classical active-site inhibitor, represents a previously unidentified therapeutic avenue for targeting calpain dysregulation in neurodegenerative conditions such as HD and AD, which are characterized by calpain-2 overactivation.

An additional advantage of A36 over several related analogs in our series is the elimination of detectable GSK3β inhibitory activity. GSK3β is a pleiotropic kinase with broad roles in metabolism, synaptic plasticity, gene expression, and cell survival (*68*, *69*), and chronic GSK3 inhibition has been associated with metabolic disturbances, altered insulin signaling, and neuropsychiatric effects in preclinical and clinical settings (*70*–*72*). Thus, by removing GSK3β inhibition while retaining robust stabilization of the CAST–calpain-2 complex, A36 functions as a much “cleaner” chemical probe of calpain-2–driven pathology. This separation of activities allows us to ascribe the observed rescue of disease relevant phenotypes primarily to calpain-2 modulation rather than to indirect effects on GSK3β-dependent signaling pathways, thereby strengthening the mechanistic link between calpain-2 overactivation, CAST depletion, and neuronal vulnerability.

At the same time, several limitations of A36 in our current study warrant consideration. First, although improved over CHIR99021, A36 still lacks optimal pharmacokinetic properties, including enhanced metabolic stability and a sufficiently long in vivo half-life. Second, the long-term consequences of chronically reinforcing CAST function in HD and AD remain unknown; these considerations underscore the need to evaluate potential side effects as A36 or next-generation analogs move toward in vivo testing. Further optimization of pharmacological properties, comparative assessment of efficacy and safety across related compounds, and exploration of brain-targeted or reversible dosing strategies will be important to maximize neuroprotection while minimizing unintended effects on calpain-dependent physiology.

In summary, this study identifies A36 as a brain-penetrant small molecule that selectively stabilizes the CAST–calpain-2 complex, prevents CAST degradation, restores mitochondrial integrity, and mitigates neurodegeneration in both HD and tauopathy models. By acting as a PPI stabilizer, A36 circumvents the limitations of broad-spectrum calpain inhibitors and recapitulates the protective effects of CAST overexpression through a pharmacologic approach. Its efficacy in both genetic and sporadic models of disease underscores the therapeutic relevance of targeting CAST–calpain-2–mediated proteolysis as a shared pathological driver in neurodegeneration. Given the broad implication of CAST–calpain-2 imbalance in multiple disorders, including ALS, PD, AD, and HD, this work establishes pharmacological stabilization of this complex as a unifying and disease-relevant strategy. Future studies are warranted to expand A36’s application across diverse neurodegenerative contexts, explore its long-term safety, and optimize its drug-like properties. Ultimately, A36 may offer a compelling reagent for the development of targeted therapies aimed at restoring mitochondrial function and proteolytic homeostasis in the aging and diseased brain.

## MATERIALS AND METHODS

### Study design

The primary aim of this study was to optimize CHIR99021 to develop a small-molecule compound with enhanced mitochondrial protective activity, stabilization of CAST, and minimal GSK3 inhibition, for application in neurodegenerative disease models. To achieve this, we performed a targeted medicinal chemistry campaign to generate and screen CHIR99021 analogs using validated MMP and cell viability assays in HD mouse striatal neurons. Candidate compounds were further evaluated for CAST stabilization and GSK3 activity using immunoblotting, kinase assays, and CETSA, which led to the identification of A36 as a lead molecule. The second aim was to elucidate the mechanism of action underlying A36-mediated mitochondrial and cellular protection. DARTS assays coupled with mass spectrometry identified CAST as a direct interactor of A36. ASMS further revealed that A36 binds preferentially to the CAST–calpain-2 complex but not to the CAST–calpain-1 complex. Coimmunoprecipitation demonstrated this selective stabilization of CAST–calpain-2 complex. Functional studies using genetic knockdown of CAST, CAPN1, and CAPN2 confirmed that the neuroprotective activity of A36 requires the presence of CAST and calpain-2. The third aim was to assess the therapeutic efficacy of A36 in disease-relevant models of neurodegeneration, including MSNs derived from iPSCs from patients with HD, HD R6/2 mice, and PS19 tauopathy mice. A36 pharmacokinetics and brain penetration were also evaluated in vivo. Power analysis was performed to determine the appropriate sample size for animal experiments aiming to detect beneficial effects of A36 treatment on both neuropathology and behavioral deficits. In all in vivo experiments described, mice were assigned to treatment groups cage by cage, and investigators assessing the outcomes in mice were blinded to the treatment group. The number of replicates, group sizes, and statistical tests used in the analysis are indicated in the respective figure legends. Samples were excluded only if they did not meet technical requirements for downstream analyses (insufficient cell number or poor sample quality). No samples were removed on the basis of statistical outlier analysis.

### SDS-PAGE and Western blotting

Protein concentrations were determined by Bradford assay (Bio-Rad Laboratories, Hercules, CA, USA). Protein (15 to 25 μg) was resuspended in 5× Laemmli buffer, boiled at 100°C for 5 min, and subjected to SDS–polyacrylamide gel electrophoresis (SDS-PAGE). Separated proteins were then transferred to nitrocellulose membranes (Bio-Rad Laboratories) and blocked for 1 hour in 5% nonfat milk in tris-buffered saline containing 0.1% Tween 20 (TBST). Membranes were then probed overnight with the primary antibodies. After washing three times in TBST, membranes were incubated for 1 hour at room temperature with secondary anti-rabbit or anti-mouse immunoglobulin G (IgG, 31430/31460, Thermo Fisher Scientific, 1:5000), followed by visualization with enhanced chemiluminescence. Representative blots have been cropped for presentation.

Protein phosphatase inhibitor cocktail (P5726) and protease inhibitor cocktails (8340) were purchased from MilliporeSigma (Burlington, MA, USA). CHIR99021 (S2924) was from Selleckchem (Houston, TX, USA). The antibodies used for the Western blot are listed as follows: Anti–DARPP-32 (ab40801, Abcam, Cambridge, UK, 1:3000), anti-BDNF (ab108319, Abcam, 1:1000), anti-ATPB (ATP synthase, beta subunit) (17247-1-AP, Proteintech, 1:3000), anti-EEA1 (28347-1-AP, Proteintech, 1:3000), anti-tau antibody (10274-1-AP, Proteintech, 1:3000), anti–phospho-tau (Ser^202^ and Thr^205^) (Thermo Fisher Scientific, clone AT8, 1:1000), anti-GSK3 (05-412, MilliporeSigma, 1:1000), anti-Htt protein (MAB5374, clone EM48, MilliporeSigma, 1:1000), anti–β-actin (A1978, MilliporeSigma, 1:10000), anti-DLP1 (611113, BD Bioscience, Franklin Lakes, NJ, USA, 1:2000), anti-PSD95 (2507, Cell Signaling Technology, Danvers, MA, USA, 1:5000), anti–Drp1-pS616 (3455S, Cell Signaling Technology, 1:1000), anti-p35/25 (2680, Cell Signaling Technology, 1:1000), anti-CAST (4146, Cell Signaling Technology, 1:2000), anti–calpain-2 (2539, Cell Signaling Technology, 1:1000), anti–calpain-1 (2556, Cell Signaling Technology, 1:1000), anti–α-spectrin-II (sc-48382, Santa Cruz Biotechnology, Dallas, TX, USA, 1:500), and anti-enolase (sc-15343, Santa Cruz Biotechnology, 1:2000), and horseradish peroxidase (HRP)–conjugated anti-rabbit or anti-mouse IgG (31430/31460, Thermo Fisher Scientific, 1:5000).

### Cell culture

Striatal neuron–like WT HdhQ7 and HD mutant HdhQ111 cells were obtained from the Cure Huntington’s Disease Initiative Foundation and were cultured in Dulbecco’s modified Eagle’s medium (DMEM) supplemented with 10% fetal bovine serum, penicillin (100 mg/ml), streptomycin (100 mg/ml), and G418 (400 μg/ml). Cells were grown at 33°C in a 5% CO_2_ environment and were used within 14 passages for studies.

iPSCs from normal subjects and patients with HD (ND42224*B, ND42228*E, and ND41656*C) were requested from NINDS Human Genetics DNA and Cell Line Repository. iPSCs were differentiated into neurons using a protocol from our previous study (*24*, *25*). Briefly, iPSCs were plated onto six-well plates precoated with 2.5% Matrigel (Corning) and allowed to reach 90% confluence in feeder-free medium (mTeSR Plus, 100-0276, STEMCELL Technologies, Vancouver, Canada). For the first 10 days, the cells were treated with SB431542 (10 mM; Tocris Bioscience, Bristol, UK) and Noggin (100 ng/ml; R&D systems, Minneapolis, MI, USA) in neural medium containing Neurobasal-A medium (Thermo Fisher Scientific, Waltham, MA) and DMEM/F12 GlutaMAX (Thermo Fisher Scientific) (1:1), B27 supplement minus vitamin A (50×, Invitrogen, Carlsbad, CA, USA), N2 supplement (100×, Invitrogen), GlutaMax (100×, Invitrogen), human recombinant fibroblast growth factor-basic (20 ng/ml; PEPROTECH, Rocky Hill, NJ, USA), and human recombinant epidermal growth factor (20 ng/ml; MilliporeSigma), penicillin (100 U/ml), and streptomycin (100 μg/ml). For the next 10 days, the cells were treated with human recombinant Sonic hedgehog (200 ng/ml; PEPROTECH), human recombinant DKK1 (100 ng/ml;, PEPROTECH) and human recombinant BDNF (20 ng/ml; PEPROTECH), and 10 mM Y27632 (MilliporeSigma) in neuronal differentiation medium containing neurobasal-A medium and DMEM/F12 GlutaMax medium (1:3), B27, N2, GlutaMax, penicillin (50 U/ml), and streptomycin (50 μg/ml). The cells were then switched to treatment with BDNF (20 ng/ml), ascorbic acid (200 mM, MilliporeSigma), dibutyryl cyclic adenosine monophosphate (0.5 mM, MilliporeSigma), and Y27632 (10 μM) in neuronal differentiation medium. Twenty days after differentiation initiation, neurons (approximately 5000 cells) were plated onto 12-mm poly-d-lysine (MilliporeSigma)/laminine (Thermo Fisher Scientific)–coated coverslips and grown in 24-well plates in neuronal differentiation medium.

### Measurement of MMP

Cells seeded on 96-well plate (8000 cells per well) were washed in phosphate-buffered saline (PBS) (pH 7.4) and incubated with 0.25 μM TMRM and Hoechst (5 μg/ml) for 20 min at 33°C for mouse striatal cells. The cells were then washed in PBS for three times and directly imaged by Keyence fluorescence microscope (BZX-710). At least three images were taken for each well. Image quantification was performed using ImageJ software. TMRM fluorescence density was normalized to the total number of cells.

Cells cultured on coverslips were washed in PBS (pH 7.4) and incubated with 0.25 μM TMRM and Hoechst (5 μg/ml) for 20 min at 33°C for mouse striatal cells and 37°C for other cells. Images were visualized by confocal microscopy (Olympus, Tokyo, Japan; Fluoview FV3000), and image quantification was performed using ImageJ software. At least 100 cells per group were counted for analysis. TMRM fluorescence density was normalized to the total number of cells.

### Measurement of mitochondrial morphology

Mitochondrial morphology was captured by immunofluorescence staining using anti-TOM20 antibody. To quantify mitochondrial morphology, Fiji ImageJ plugin “Mitochondrial Analyzer” (https://github.com/AhsenChaudhry/Mitochondria-Analyzer) (*73*) was used to assess the mean aspect ratio of mitochondria in individual cells. Elongated, intermediate, and fragmented mitochondria are defined by the mean aspect ratio when it is >4, <4 and >2, or <2, respectively. The percentage of cells with mitochondria in either form is presented as histograms.

### Measurement of cell viability

Striatal HdhQ7/111 cells were plated on 96-well plate (8000 cells per well) and treated with A36 for 48 hours. The cells were then washed once using serum-free DMEM and cultured in the serum-free DMEM with A36 for 12 hours before assessment of cell survival using CCK8 (HY-K0301, MedChemExpress) or MTT (11 465 007 001, Roche) assay following the manufacturer’s procedures.

### Measurement of in vitro GSK3 activity

Briefly, the test compounds are screened in 1% dimethyl sulfoxide (DMSO) (final) in the well. For 10-point titrations, threefold serial dilutions are conducted from the starting concentration. In each well of the 384-well plate, 100 nl of 100× test compound in 100% DMSO, 2.4 μl of kinase buffer [50 mM Hepes (pH 7.5), 0.01% BRIJ-35, 10 mM MgCl_2_, and 1 mM EGTA], 5 μl of 2× peptide/kinase mixture, and 2.5 μl of 4× ATP solution were added. After 30 s of shaking, the kinase reaction was processed for 60 min at room temperature. Then, 5 μl of development reagent solution was added, shaken for 30 s, and incubated for 60 min at room temperature. It was then read on a fluorescence plate reader.

### MDCK permeability measurement

MDR1-MDCK cell monolayers were grown to confluence on collagen-coated, microporous membranes in 12-well assay plates. Details of the plates and their certification are shown below. The permeability assay buffer was Hanks’ balanced salt solution containing 10 mM Hepes and 15 mM glucose at a pH of 7.4. The buffer in the receiver chamber also contained 1% bovine serum albumin. The dosing solution concentration was 5 μM of test article in the assay buffer. Cell monolayers were dosed on the apical side (A-to-B) or basolateral side (B-to-A) and incubated at 37°C with 5% CO_2_ in a humidified incubator. Samples were taken from the donor and receiver chambers at 120 min. Each determination was performed in duplicate. The flux of lucifer yellow was also measured postexperimentally for each monolayer to ensure that no damage was inflicted on the cell monolayers during the flux period. All samples were assayed by LC-MS/MS using electrospray ionization. Analytical conditions are outlined in Appendix 1. The apparent permeability (*P*_app_) and percent recovery were calculated as follows

$$P_{\text{app}}=\left(d\text{Cr}/dt\right)\;\times \;V_{r}/\left(A\;\times \;\text{CA}\right)$$ (1)

$$\begin{array}{c} \text{Percent recovery}=100\;\times \\ \left[\left(V_{r}\;\times \;{C_{r}}^{\text{final}}\right)+\left(V_{d}\;\times \;{C_{d}}^{\text{final}}\right)\right]/\left(V_{d}\;\times \;\text{CN}\right) \end{array}$$ (2)

where *d*Cr/*d*t is the slope of the cumulative concentration in the receiver compartment versus time in micromolars per second; *V*_r_ is the volume of the receiver compartment in cubic centimeters; *V*_d_ is the volume of the donor compartment in cubic centimeters; *A* is the area of the insert (1.13 cm^2^ for 12 wells); CA is the average of the nominal dosing concentration and the measured 120-min donor concentration in micromolars; CN is the nominal concentration of the dosing solution in micromolars; *C*_r_ final is the cumulative receiver concentration in micromolars at the end of the incubation period; and *C*_d_ final is the concentration of the donor in micromolars at the end of the incubation period. Efflux ratio is defined as *P*_app_ (B-to-A) / *P*_app_ (A-to-B).

### Drug affinity–responsive target stability

HdhQ111 cells were cultured in 10-cm dish till reaching 80 to 90% confluence. The cells were washed twice with cold DPBS and scrapped off in 650 μl of lysis buffer [0.4% Triton X-100, 400 mM NaCl, 100 mM tris-HCl (pH 7.5), and 20% glycerol] supplemented with protease and phosphatase inhibitors and transferred into a 1.5-ml Eppendorf tube. The tube was incubated on ice for 10 min and centrifuged at 13,000*g* for 15 min at 4°C. The supernatant was transferred into a new 1.5-ml tube and was kept chilled on ice. Ninety-nine microliters of the lysates was placed into two 1.5-ml tubes. DMSO (1 μl) or 10 mM A36 (1 μl) was added into the tube and incubated at room temperature for 40 min with gentle shaking. After incubation, the solution was divided into 20-μl aliquot in different tubes, and digestion was started by adding 2 μl of Pronase solutions (1:800 = 0.0625 μg/μl). TNC solution (1×) was made [for 1 ml of 10× TNC buffer, mixed were 300 μl of ultrapure water with 100 μl of 5 M sodium chloride, 100 μl of 1 M calcium chloride, and 500 μl of 1 M tris-HCl (pH 8.0)], and Pronase solution (0.5 μg/μl) (mg/ml) (1:100) in the 1× TNC buffer was made. After 5 min, the digestion was halted by adding 2 μl of cold 20× protease inhibitor cocktail. It was mixed well and incubated on ice for 10 min. Samples are ready for Western blot.

### Measurement of mitochondrial respiratory capacity

Mouse striatal HdhQ7/Q111 cells were seeded in XFp eight-well miniplates (103025-100, Agilent, Santa Clara, CA, USA) at 3000 cells per well in 100 μl of growth medium. Two days after treatment with A36, mitochondrial respiration activity in intact cells was analyzed using a Seahorse Bioscience XFp Extracellular Flux analyzer (Agilent). Briefly, 1 hour before measuring oxygen consumption, cell culture medium was replaced with XF assay medium and maintained in a non-CO_2_ incubator for 1 hour at 33°C. Sensor cartridges were placed in the XFp analyzer according to the manufacturer’s instructions from the Mito Stress Test kit (Agilent, 103010-100). Mitochondrial function was determined by the sequential injection of oligomycin A (1 μM), carbonyl cyanide *p-*trifluoromethoxyphenylhydrazone (1 μM), and rotenone/antimycin A (0.5 μM). The total protein content in each well was determined after respiration measurement, and all results were normalized to the total protein content.

### Mitochondrial ROS measurement

Mouse striatal cells cultured on coverslips were washed in PBS (pH 7.4) and incubated with 5 μM MitoSOX Red (Invitrogen, M36008), a mitochondrial superoxide indicator, and Hoechst (5 μg/ml) for 10 min at 33°C. Images were visualized by confocal microscopy (Olympus, Fluoview FV3000) and image quantification performed using ImageJ software. At least 100 cells per group were counted for analysis. MitoSOX fluorescence density was normalized to the total number of cells.

### Mouse models

All animal studies were conducted in accordance with protocols (2015-0024 and 2017-0153) approved by the Institutional Animal Care and Use Committee of Case Western Reserve University and were performed on the basis of the National Institutes of Health Guide for the Care and Use of Laboratory Animals. Sufficient procedures were used for reducing pain or discomfort of mice during the experiments. All mice were maintained with a 12-hour light/dark cycle (on at 06:00 hours and off at 18:00 hours).

Male R6/2 mice and their WT littermates (4 weeks old) were purchased from the Jackson Laboratory [Bar Harbor, ME, USA; B6CBA-TgN (HD exon 1); JAX stock number: 006494]. R6/2 mice (C57BL/6 and CBA genetic background) are transgenic for the 5′ end of the human *HD* gene carrying 100 to 150 glutamine (CAG) repeats. Male R6/2 mice at 6 to 12 weeks were used in the study.

Male tau P301S transgenic mice (PS19) and their WT litter mates (8 weeks old) were purchased from the Jackson Laboratory [Bar Harbor, ME, USA; B6; C3Tg (Prnp-MAPT*P301S) PS19Vle/J, JAX: 008169]. Male tau P301S mice at 4 to 9 months were used in the study.

### A36 treatment in vivo

All randomization and compound treatments were prepared by a researcher not associated with behavioral and neuropathological analyses. For R6/2 mice, male heterozygous HD R6/2 mice and their age-matched WT littermates were given an intraperitoneal injection of A36 [10 mg/kg dissolved in 10% Solutol (MedChemExpress, NJ, USA)] or vehicle once a day for 5 days/week starting at 6 weeks old. At 12 weeks, the treatment was terminated. For tau P301S mice, male heterozygous PS19 tau P301S mice and their age-matched WT littermates were given an intraperitoneal injection of A36 (10 mg/kg dissolved in 10% Solutol or vehicle once a day for 5 days/week starting at 4 months old till 9 months old.

### Mouse behavioral analysis

All behavioral analyses were conducted by a researcher blinded to genotypes and treatment groups, as we previously described. Gross locomotor activity was assessed in R6/2 mice and age-matched WT littermates at 6, 9 and, 12 weeks old. In an open-field activity chamber (Omnitech Electronics Inc., Columbus, OH, USA), mice were placed in the center of the chamber and allowed to explore while being tracked by an automated beam system (Vertax, Omnitech Electronics Inc.,). Horizontal and vertical distances and rearing activities were all recorded. Because R6/2 mice were sensitive to changes in the environment and handling, we only conducted a 1-hour locomotor activity analysis for these mice and the WT littermates.

Hindlimb clasping was assessed by a tail suspension test once a week from 9 to 12 weeks old. Briefly, the mice were suspended for 20 s and hindlimb latency or four paw clasping was recorded using a scoring system: clasping over 10 s, score 3; 5 to 10 s, score 2; 0 to 5 s, score 1; and 0 s, score 0. Body weights and survival rates of R6/2 mice and WT littermates were recorded throughout the study period.

For Barnes maze, on the test day, the mice were brought to the testing room 30 min before performing the Barnes maze test to allow habituation. Briefly, all the testing mice received three consecutive days of trials, with three trials each day. After being placed in the center of the platform at the beginning of each trial, the mice were allowed to explore for 3 min to find the target escape box. Mice that failed to enter the target escape hole in the given time were led to it by the operator. The mice were allowed to remain in the target hole for 2 min before returning to the home cage. After completing the 3-day trials, the mice were examined on days 5 and 12 with one test to monitor the long-term spatial learning and memory activities. The maze and the escape box were cleaned carefully after each trial to avoid odor disturbance. All the trials and tests were recorded with a video system. The total time to enter the target escape box (latency to the target box) and the number of times the wrong holes were explored (the total errors) were recorded.

For Y maze, on the test day, the mice were brought to the testing room 1 hour before performing the Y-maze test to allow habituation. The mice were placed in the middle of the Y maze and allowed to explore the three arms for 4 min. During exploration, the arm entries were recorded. The equipment was cleaned after every test to avoid odor disturbance. Spontaneous alternation was defined as a successive entry into three different arms on overlapping triplet sets.

### RNA interference

CAST shRNA (TRCN0000080114), CAPN1 shRNA (TRCN0000030706 and TRCN0000030707), CAPN2 shRNA (TRCN0000030669 and TRCN0000030670), and control shRNA were purchased from MilliporeSigma. Cells were transduced with lentiviral particles containing CAST shRNA or CAPN1/2 shRNA for 2 days and selected using puromycin (1 μg/ml) (Corning) to generate a stable CAST or calpain knock-down line.

### Immunohistochemistry

Mice were deeply anesthetized and transcardially perfused with 4% paraformaldehyde in PBS. Brain sections (14 μm, coronal) were subjected to antigen retrieval in 0.01 M sodium citrate buffer plus 0.05% Tween 20 (pH 6.0), and slides were incubated with 3% hydrogen peroxide (H_2_O_2_) in methanol to quench endogenous peroxidase. They were then treated with 5% normal goat serum (Invitrogen) in TBST for 1 hour at room temperature. Sections were then incubated with anti–DARPP-32 (1710-1, Epitomics, Burlingame, CA, USA; 1:500), anti-Htt protein (MAB5374, clone EM48, MilliporeSigma, 1:500), or anti–phospho-tau (Ser^202^, Thr^205^) (Thermo Fisher Scientific, clone AT8, 1:1000) antibody in a humidified chamber overnight at 4°C. The next day, slides were incubated with biotin-conjugated secondary antibody (goat anti-mouse/rabbit) and streptavidin-conjugated HRP using an immunohistochemistry select HRP/DAB kit (MilliporeSigma, DAB150) and DAB solution following the manufacturer’s instructions. Images were captured using a digital microscope (VHX-7000, Keyence, Osaka, Japan). Quantitation was conducted using Fiji imageJ software. The same image exposure times and threshold settings were used for all group sections. A researcher blinded to experimental groups conducted quantification analyses.

### Immunocytochemistry

Cells cultured on coverslips were washed in PBS (pH 7.4), fixed in 4% paraformaldehyde, and permeabilized in 0.1% Triton X-100. After incubation with 2% normal goat serum, fixed cells were incubated overnight at 4°C with the following primary antibodies: anti–DARPP-32 (ab40801, Abcam, 1:500), anti-MAP2 (4542, Cell Signaling Technology, 1:500), anti–Neurofilament L (AB9568, MilliporeSigma, 1:1000), anti-GAD65/67 (ab183999, Abcam, 1:500), anti-tubulin β 3 (Tuj1) (801201, BioLegend, San Diego, CA, USA, 1:500), and anti-CAST (ab28252, Abcam, 1:1000). Cells were washed in PBS (pH 7.4) and incubated with Alexa Fluor goat anti-mouse/rabbit 568 or 488 secondary antibodies (Thermo Fisher Scientific, 1:1000), followed by incubation with Hoechst dye (1:10,000). Coverslips were mounted, and slides were imaged by confocal microscopy (Olympus). MAP2^+^ neurite length, NFL^+^ axonal length were quantified using ImageJ software.

Frozen brain sections (14 μm, coronal) were subjected to antigen retrieval in 0.01 M sodium citrate buffer plus 0.05% Tween 20 (pH 6.0). They were then treated with 10% normal goat serum (Invitrogen) in PBST for 1 hour at room temperature. Sections were then incubated with anti–DARPP-32 (1710-1, Epitomics, Burlingame, CA, USA; 1:500), anti-GFAP (ab7260, Abcam, 1:1000), anti-IBA1 (019-19741, Wako, 1:1000), anti-NeuN (MAB377, MilliporeSigma, 1:500), anti-CAST (ab28252, Abcam, 1:1000), anti–phospho-tau (Ser^202^, Thr^205^) (Thermo Fisher Scientific, clone AT8, 1:1000), anti-Oligo2 (AB9610, MilliporeSigma, 1:1000), anti-CC1 (OP80, MilliporeSigma, 1:1000), and anti-BDNF (ab108319, Abcam, 1:500) in a humidified chamber overnight at 4°C. The next day, slides were incubated Alexa Fluor goat anti-mouse/rabbit 568 or 488 secondary antibodies (Thermo Fisher Scientific, 1:1000), followed by incubation with Hoechst dye (1:10,000). Coverslips were mounted, and slides were imaged by confocal microscopy (Olympus, FV3000).

### Preparation of total lysates

Cells were washed in cold PBS (pH 7.4) and incubated on ice for 30 min in total lysis buffer [50 mM tris-HCl (pH 7.5), 150 mM NaCl, 1% Triton X-100, protease inhibitors cocktail, and phosphatase inhibitors cocktail (MilliporeSigma)]. Mouse brains were minced and homogenized in lysis buffer and placed on ice for 30 min. Cells or tissues were centrifuged at 12,000*g* for 10 min at 4°C to generate total lysate supernatants.

### Immunoprecipitation

Cells were harvested and lysed in total lysis buffer for 30 min on ice and centrifuged at 12,000*g* for 10 min at 4°C. The supernatants were incubated with anti-CAST antibody overnight at 4°C, followed by incubation with protein A/G beads (sc-2003, Santa Cruz Biotechnology) for 2 hours at 4°C. The immunoprecipitates were washed with lysis buffer three times for a total of 30 min and then subjected to Western blot.

### Label-free proteomics

The samples were processed using a filter-aided sample preparation clean-up protocol (*74*) with Amicon Ultra molecular weight cutoff 3 K filters (Millipore, Billerica, MA, USA). Samples were reduced and alkylated on filters using 10 mM dithiothreitol (Acros, Fair Lawn, NJ, USA) and 25 mM iodoacetamide (Acros), respectively, and then concentrated to a final volume of 40 μl in 8 M urea. Protein concentrations were measured using the Bradford method according to the manufacturer’s instructions (Bio-Rad).

Following reduction and alkylation, total protein (10 μg) was subjected to enzymatic digestion. The urea concentration was adjusted to 4 M using 50 mM tris (pH 8) and proteins digested using mass spectrometry–grade lysyl endopeptidase (Wako Chemicals, Richmond, VA, USA) at an enzyme/substrate ratio of 1:40 for 2 hours at 37°C. Then, the urea concentration was further adjusted to 2 M using 50 mM tris (pH 8) and lysyl peptides digested overnight 37°C in sequencing-grade trypsin (Promega, Madison, WI, USA) at an enzyme/substrate ratio of 1:40. Last, samples were diluted in 0.1% formic acid (Thermo Fisher Scientific, Rockford, IL, USA) before LC-MS/MS analysis.

The 300 ng of lysed protein from each of group were loaded onto a column in a 3-μl injection volume with blanks in between for a total of four LC/MS/MS runs. Data were acquired with an Orbitrap Velos Elite mass spectrometer (Thermo Electron, San Jose, CA, USA) equipped with a Waters nanoACQUITY LC system (Waters, Taunton, MA, USA). Peptides were desalted on a trap column (180 μm by 20 mm, packed with C18 Symmetry, 5 μm, 100 Å, Waters) and subsequently resolved on a reversed-phase (RP) column [75 μm–by–250 mm nano column, packed with C18 BEH130, 1.7 μm, 130 Å (Waters)]. LC was conducted at an ambient temperature at a flow rate of 300 nl/min using a gradient mixture of 0.1% formic acid in water (solvent A) and 0.1% formic acid in acetonitrile (solvent B). The gradient ranged from 4 to 44% solvent B over 210 min. Peptides eluting from the capillary tip were introduced into the nanospray mode at a capillary voltage of 2.4 kV. A full scan was obtained for eluted peptides in the range of 38 to 1800 atomic mass units, followed by 25 data-dependent MS/MS scans. MS/MS spectra were generated by collision-induced dissociation of peptide ions at a normalized collision energy of 35% to generate a series of b- and y-ions as major fragments. In addition, a 1-hour wash was included between samples. The proteins were identified with Mascot (Matrix Sciences, London, UK). Key search parameters were trypsin for enzyme, a maximum of 1 missed cleavage, peptide charge states of +2 to +3; peptide tolerance of 10 parts per million, and MS/MS tolerance of 0.8 Da. Oxidation of methoinine was a variable modification. Identifications were merged, the spectra summed and then quantified using spectral counting (selecting normalized total spectra) in Scaffold version 4.4.0 (Proteome Software Inc., Portland, Oregon). The protein probability was determined using a protein threshold of 99%, two peptides, and a peptide threshold of 95%. A false discovery rate of 0.86% was calculated using ProteinProphet algorithm for the 56,902 spectra examined. Both MASCOT and Scaffold strategies used the UniProt human database from June 2016 (20,199 sequences).

### Affinity selection mass spectrometry

Affinity mass spectrometry screening was performed by Momentum Biotechnologies (Boston, MA). Recombinant protein CAST, calpain-1, and calpain-2 (final concentration 1 μM, each) were mixed with A36 (10 μM) in the buffer containing 50 mM tris 8, 150 mM NaCl, 2 mM EDTA, and 10 mM CaCl_2_. Protein samples were dispensed into compound plates at 10 μl per well using a multichannel pipette. Plates were sealed, briefly centrifuged, mixed on a plate shaker, and centrifuged again before placement in the autosampler, which was maintained at 4°C throughout the experiment. The ALIS platform consisted of an Agilent 1260 HPLC pump for size-exclusion chromatography (SEC) coupled to an Agilent 1290 UHPLC pump for RP chromatography, with a high-pressure switching valve interfaced to an Agilent 6230B time-of-flight mass spectrometer. All mass spectrometry data were acquired in positive ion mode. SEC separation was conducted using a polyhydroxyethyl A column (50 mm by 2.1 mm, 3 μm, 60 Å; PolyLC) with buffer A (700 mM ammonium acetate) and buffer B (70% acetonitrile). RP separation was performed using a Kinetex C18 column (50 mm by 2.1 mm, 2.6 μm, 100 Å; Phenomenex) with buffer A (water +0.1% formic acid) and buffer B (90% acetonitrile +0.1% formic acid). Data processing and analysis were carried out using Agilent MassHunter software supplemented with custom in-house analytical tools.

### Quantification and statistical analysis

Sample sizes were determined by a power analysis based on pilot data collected in our laboratory or from published studies. For animal studies, we used *n* = 10 to 15 mice per group for behavioral tests, *n* = 3 to 6 mice per group for biochemical analyses, and *n* = 3 to 10 mice per group for pathology studies. In cell culture studies, each experiment was independently conducted at least three times. For animal studies, we ensured randomization and blinded evaluations. For imaging studies, a blinded observer performed quantification analyses. No samples or animals were excluded from our analysis.

Data were analyzed using GraphPad Prism 9 (GraphPad Software, San Diego, CA, USA). The unpaired Student’s *t* test was used for comparisons between two groups. Comparisons between three or more independent groups were performed using one-way analysis of variance (ANOVA), followed by Tukey’s post hoc test. Comparisons of the effect of independent variables on a response variable were performed using two-way ANOVA. Survival rate was analyzed by log-rank (Mantel-Cox) test. All values are reported as the means ± SEM. Data are representative of at least three independent experiments. Statistical parameters were presented in each figure legend. We considered *P* < 0.05 as statistically significant.
