# Supplementary material for: A small-molecule stabilizer of the calpastatin–calpain-2 complex restores mitochondrial function and mitigates neurodegeneration
Source: Sci Adv. 2026 Mar 27;12(13):eaeb1174. doi: 10.1126/sciadv.aeb1174 (PMC13025052; doi:10.1126/sciadv.aeb1174)
Supplement: Supplementary file 1 — Supplementary Text Figs. S1 to S7 Tables S1 to S8 Supporting Information 1 to 4 [file sciadv.aeb1174_sm.pdf]

Science Advances
AAAS

Supplementary Materials for

A small-molecule stabilizer of the calpastatin–calpain-2 complex restores mitochondrial function and mitigates neurodegeneration

Di Hu *et al.*

Corresponding author: Drew J. Adams, dja59@case.edu; Xin Qi, xxq38@case.edu

Sci. Adv. **12**, eaeb1174 (2026)
DOI: 10.1126/sciadv.aeb1174

This PDF file includes:

Supplementary Text
Figs. S1 to S7
Tables S1 to S8
Supporting Information 1 to 4

Supplementary Text

Supplementary Material and Methods

Thermo-shift profiling

HdhQ111 striatal cells cultured in 10 cm dish were treated with compound for 30 h. After wash with DPBS, cells were dissociated with Trypsin and pipetted down using 5ml DPBS and transferred to 1.5ml Eppendorf tube subjected for centrifuge at 1500 rpm for 3 minutes. The cell pellet was washed with DPBS once and centrifuges again at 1500 rpm. Then, cells were suspended in 600ul DPBS with protease and phosphatase inhibitors and transferred to three 1.5ml Eppendorf tubes (200ul cell suspension in each). Cells were then incubated at 52 °C, 58 °C and 64 °C, for 3min, followed by incubation at room temperature for 3 min. Cells were then lysed through three cycles of freezing in liquid nitrogen and thawing at 25C (Vortex is recommended after thawing). The cell lysates were then subjected to centrifuge at 4C for 15 min under 16000 g. Supernatant were collected and subjected for measurement of protein concentration and western blot to detect the protein level of GSK3.

Measurement of liver microsome stability

Male CD-1 mouse liver microsomes (Lot# 2010017) were purchased from XenoTech. The reaction mixture, minus NADPH, was prepared as described below. The test article was added into the reaction mixture at a final concentration of 1 μM. The control compound, testosterone, was run simultaneously with the test article in a separate reaction. The reaction mixture (without cofactor) was equilibrated in a shaking water bath at 37°C for 5 minutes. The reaction was initiated by the addition of the cofactor, and the mixture was incubated in a shaking water bath at 37°C. Aliquots (100 μL) were withdrawn at 0, 10, 20, 30, and 60 minutes. Test article and testosterone samples were immediately combined with 400 μL of ice-cold 50/50 acetonitrile (ACN)/H2O containing 0.1% formic acid and internal standard to terminate the reaction. The samples were then mixed and centrifuged to precipitate proteins. All samples were assayed by LC-MS/MS using electrospray ionization. Analytical conditions are outlined in Appendix 1. The peak area response ratio (PARR) to internal standard was compared to the PARR at time 0 to determine the percent remaining at each time point. Half-lives and clearance were calculated using GraphPad software, fitting to a single-phase exponential decay equation.

Isolation of mitochondrial fraction

Cells were washed in cold PBS and incubated on ice for 30 min in subcellular lysis buffer (250mM sucrose, 20mM HEPES-NaOH, pH 7.5, 10mM KCl, 1.5mM MgCl2, 1mM EDTA, protease and phosphatase inhibitor cocktails (MilliporeSigma)). Cells were disrupted 20 times by repeated aspiration through a 25-gauge needle, followed by a 30-gauge needle. Homogenates were centrifuged at 800 × g for 10 min at 4 °C and resulting supernatants further centrifuged at 10,000 × g for 20 min at 4 °C. Pellets were washed in lysis buffer and re-centrifuged at 10,000 × g for 20 min at 4 °C. Final pellets were suspended in lysis buffer containing 1% Triton X-100 and were assigned as mitochondrial-rich lysate fractions. Supernatants were designated as cytosolic fractions.

Real-time PCR

Total RNA was isolated using RNeasy Mini Kit (QIAGEN, Hilden, Germany), and 0.5–1 μg of total RNA was used to synthesize cDNA using QuantiTect Reverse Transcription Kit (QIAGEN). qRT-PCR was performed using QuantiTect SYBR Green (QIAGEN) and analyzed with the StepOnePlus Real-Time PCR System (ThermoFisher Scientific). Three replicates were performed for each biological sample, and the expression values of each replicate were normalized against GAPDH cDNA using the 2-ΔΔCT method. The primers used were as follows (5’−3’):

mGAPDH-s: GACTTCAACAGCAACTCCCAC;
mGAPDH-as: TCCACCACCCTGTTGCTGTA.

mCAST1-s: GGAAGGACAAACCAGAGAAGC;
mCAST1-as: AGGGGCAGCTATCCAAATCTT.

mCAST2-s: CAATCAAGTGAGCAACCTGTGG;
mCAST2-as: CGGAAGATTTTTGGGCTCTGA.

Figure S1

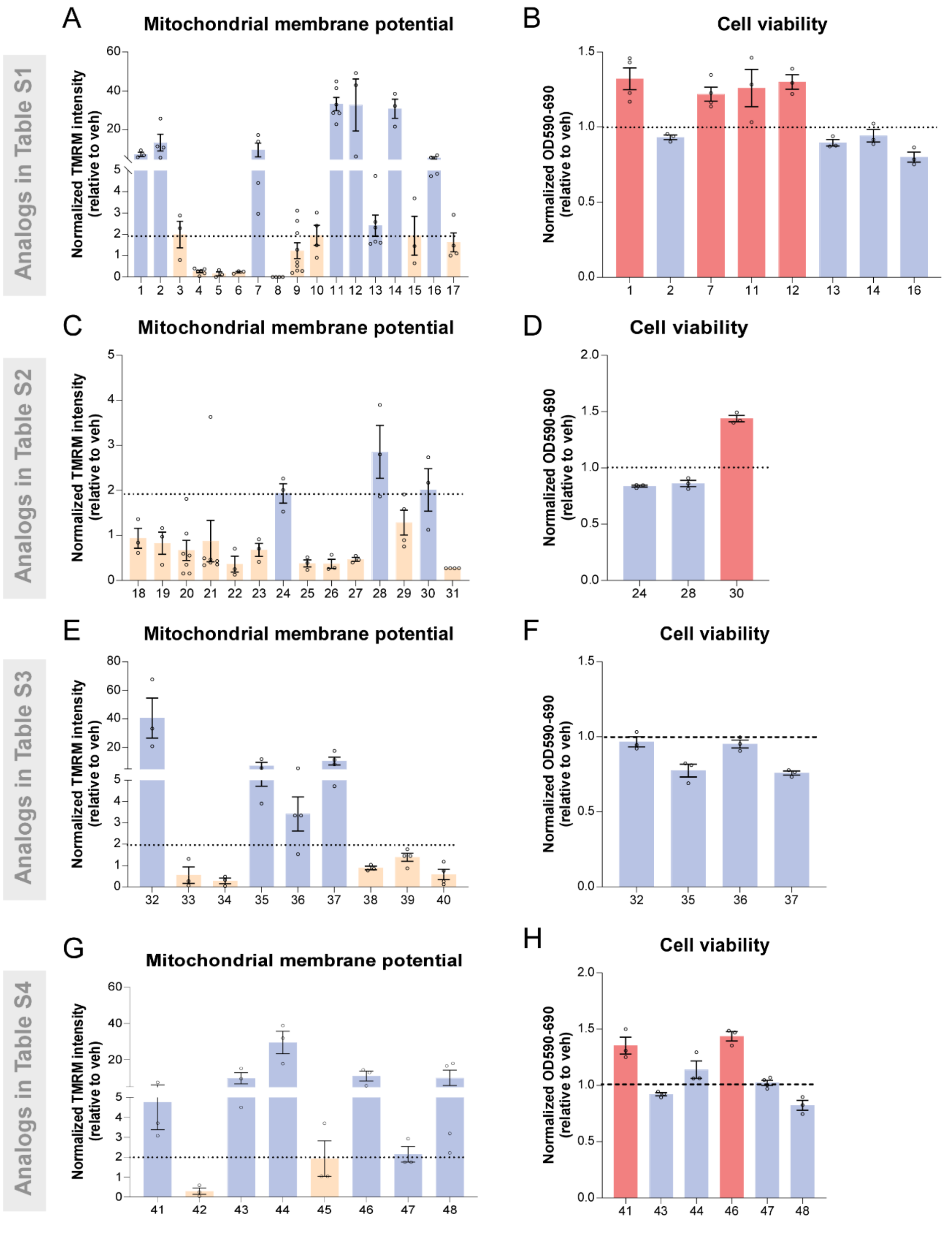

Fig. S1. Measurement of bioactivity of CHIR99021 analogs as shown in Tables S1-S4.
HdhQ111 mouse striatal cells were treated with CHIR99021 analogs at 5 µM for 48 hours. (**A**) (**C**) (**E**) (**G**) Fold change of TMRM fluorescence intensity in HdhQ111 cells treated with indicated analogs. Histograms shown in purple are positive on mitochondrial membrane potential (MMP). n = 4. (**B**) (**D**) (**F**) (**H**) Quantification of relative OD590-690 in MTT assays. Histograms shown in red are positive on cell viability. n = 3. Quantitative data for all panels are presented as means ± SEM. Data are compared by unpaired student's t-test.

Figure S2

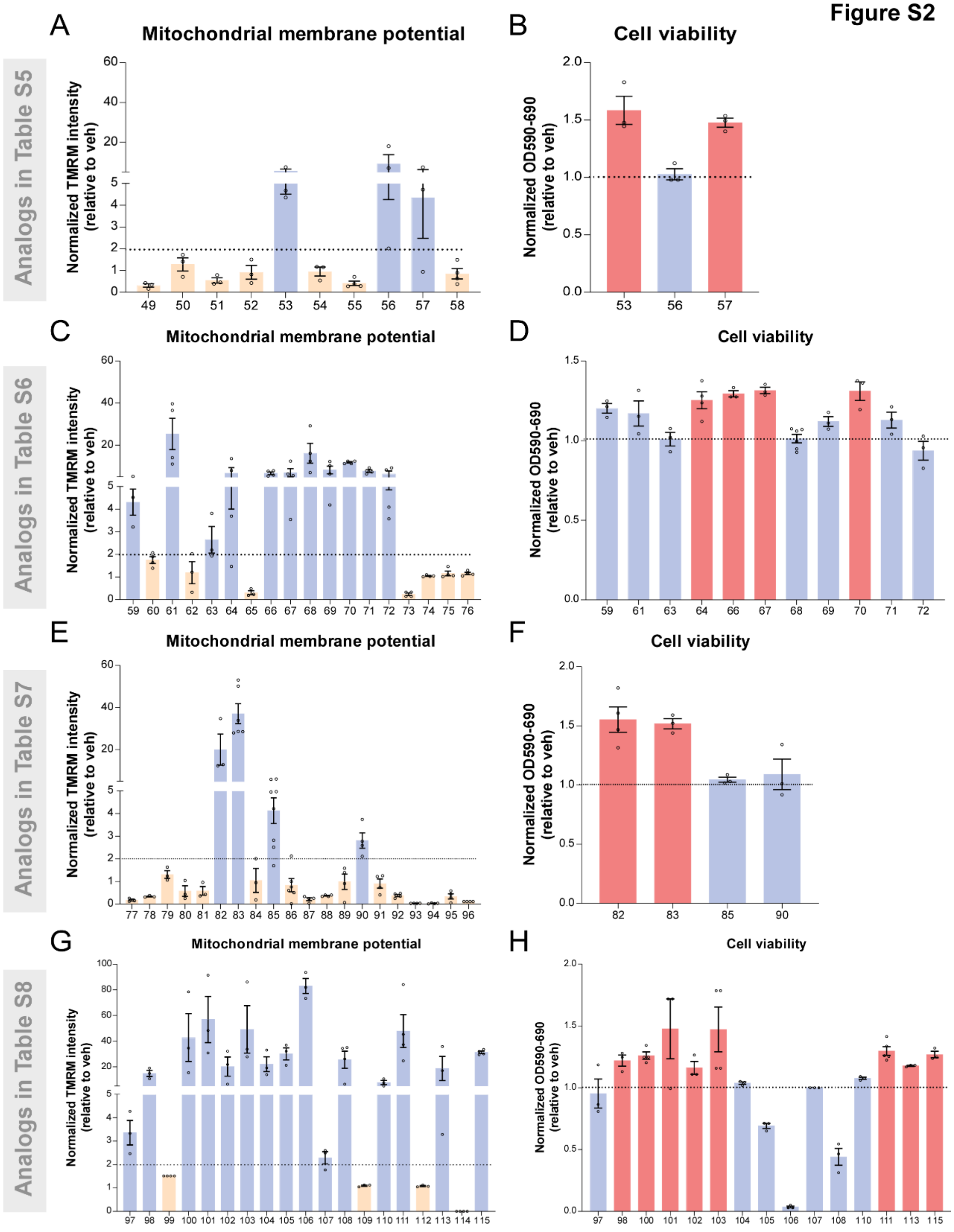

Fig. S2. Measurement of bioactivity of CHIR99021 analogs as shown in Tables S5-S8.
HdhQ111 mouse striatal cells were treated with CHIR99021 analogs at 5 µM for 48 hours. (**A**) (**C**) (**E**) (**G**) Fold change of TMRM intensity in HdhQ111 cells treated with indicated analogs. Histograms shown in purple are positive on MMP. n = 4. (**B**) (**D**) (**F**) (**H**) Quantification of relative OD590-690 in MTT assays. Histograms shown in red are positive on cell viability. n = 3. Quantitative data for all panels are presented as means ± SEM. Data are compared by unpaired student's t-test.

Figure S3

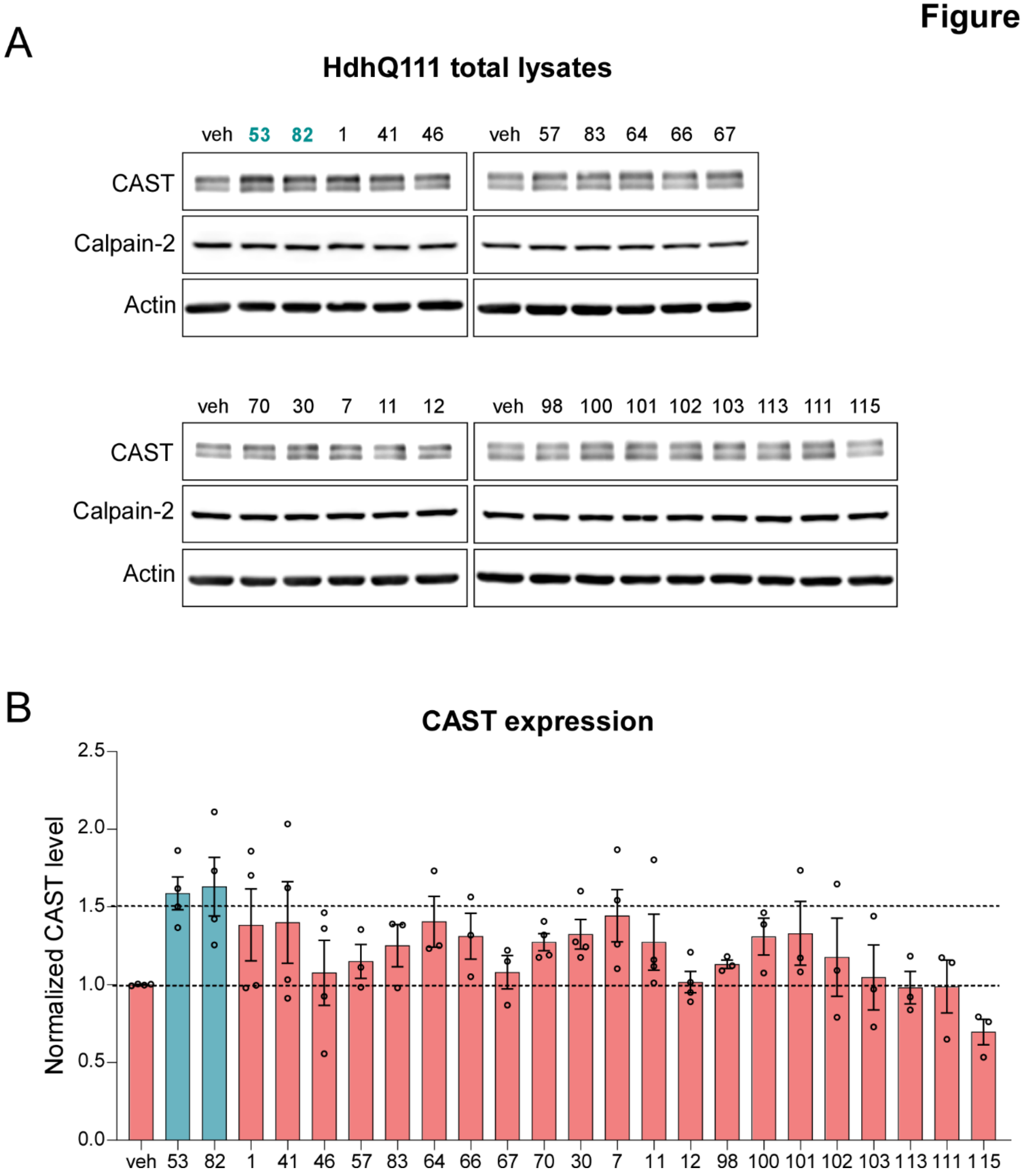

Fig. S3. Effect of CHIR99021 analogs on CAST expression.
HdhQ111 mouse striatal cells were treated with CHIR99021 analogs at 5 µM for 36 hours, followed by 2-hour serum starvation before harvest. (**A**) Representative blots of CAST and calpain-2 in HdhQ111 cells treated with DMSO (veh) or CHIR99021 analogs. (**B**) Quantification of relative density of CAST is shown in histogram. n = 3. Compounds 53 and 82 are prominent to restore CAST level. Quantitative data for all panels are presented as means ± SEM. Data are compared by unpaired student's t-test.

Figure S4

A

Thermoshift profiling

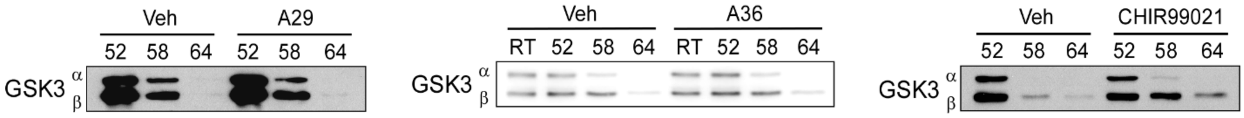

B

Liver microsome stability

Test Article	Species	% Remaining of Initial (n=1)					Half-lifea (min)	CLintb (mL/min/ mg protein)
		0 min	10 min	20 min	30 min	60 min		
A36	Mouse	100	<1.00	<1.00	<1.00	<1.00	<10 (1.28)	>0.139 (1.08)
A29	Mouse	100	<1.00	<1.00	<1.00	<1.00	<10(0.972)	>0.139(1.43)

Fig. S4. Characterization of compound 53 and 82.
(**A**) Inhibitory effect of CHIR99021, compound 53 (designed as A29) and 82 (designed as A36) on GSK3 was examined by cell-based thermos-shift profiling assay followed by western blot analysis. Shown are the representative blots. n = 3. (B) Summary of liver microsome stability of A29 and A36. Quantitative data for all panels are presented as means ± SEM. Data are compared by unpaired student's t-test.

Figure S5

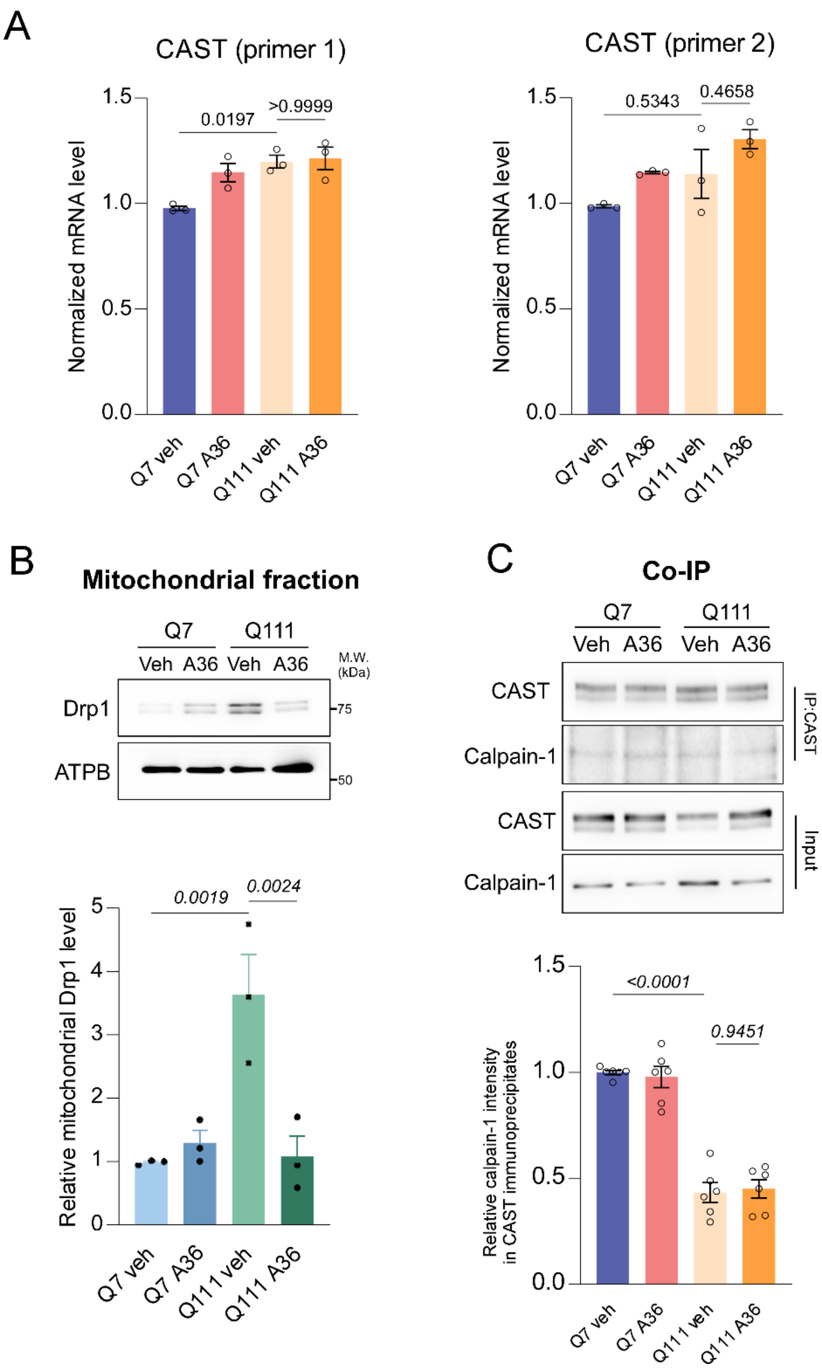

Fig. S5. Effect of A36 treatment in cell culture.
(A) The mRNA level of CAST was examined by RT-qPCR using different primers in Q7 (HdhQ7) and Q111 (HdhQ111) cells treated with Veh or A36. n = 3. (**B**) Mitochondrial fraction was isolated from Q7 and Q111 cells treated with Veh or A36 and subjected for western blot analysis. Shown are the representative blots. ATPB is a mitochondrial loading control. Quantification of relative density of Drp1 is shown in histogram. n = 3. **(C)** Total cell lysates were harvested from Q7 and Q111 cells treated with A36, subjected for co-immunoprecipitation with anti-CAST antibody, and followed by western blot analysis. Shown are the representative blots. Quantification of relative calpain-1 intensity in CAST immunoprecipitates is shown in histogram. n = 6. All data are expressed as the mean ± SEM from three independent experiments. Data are compared by one-way ANOVA with Tukey's *post-hoc* test.

Figure S6

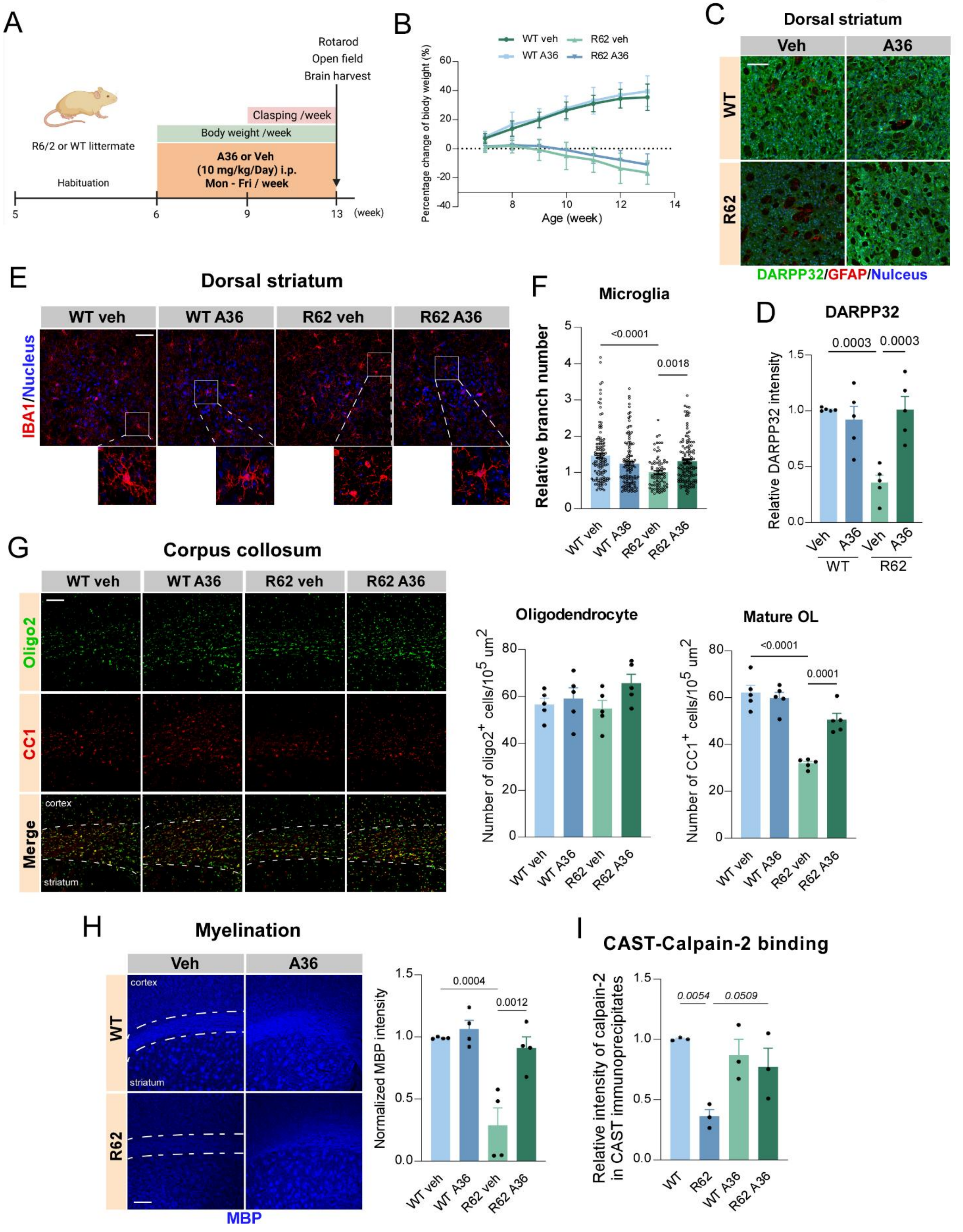
A
Rotarod
Open field
Brain harvest
Clasping /week
Body weight /week
R6/2 or WT littermate
A36 or Veh
(10 mg/kg/Day) i.p.
Mon - Fri / week
Habituation
5
6
9
13
(week)
B
WT veh
WT A36
R62 veh
R62 A36
Percentage change of biody weight (%)
Age (week)
C
Dorsal striatum
Veh
A36
WT
R62
DARPP32/GFAP/Nulceus
E
Dorsal striatum
WT veh
WT A36
R62 veh
R62 A36
IBA1/Nucleus
F
Microglia
<0.0001
0.0018
Relative branch number
D
DARPP32
0.0003
0.0003
Relative DARPP32 intensity
G
Corpus collosum
Oligo2
CC1
Merge
cortex
striatum
Oligodendrocyte
Number of oligo2+ cells/10^5 um^2
Mature OL
<0.0001
0.0001
Number of CC1+ cells/10^5 um^2
H
Myelination
MBP
0.0004
0.0012
Normalized MBP intensity
I
CAST-Calpain-2 binding
0.0054
0.0509
Relative intensity of calpain-2 in CAST immunoprecipitates
WT
R62
WT A36
R62 A36

Fig. S6. A36 treatment mitigates neuropathology in HD R6/2 mouse model.
(**A**) Experimental scheme of A36 administration in R6/2 mouse model of HD. (**B**) Body weight of Veh- or A36-treated R6/2 mice and their wild-type littermates were measured every week. Graph shows the percentage of change on body weight (compared to age of 6-week). n = 10 -15 mice per group. (**C**) Representative images of DARRP32 and GFAP staining of the brain sections from Veh- or A36-treated WT and R6/2 mice. scale bar: 20 µm. (**D**) Quantification of DARRP32 intensity per area is shown in histogram. n = 5 mice per group. (**E**) Representative images of IBA1 staining of the brain sections from Veh- or A36-treated WT and R6/2 mice. Scale bar: 20 µm. (**F**) Quantification of number of branches per microglia. At least 50 cells were counted from 3 mice per group. (**G**) Representative images of Oligo2 (a marker of oligodendrocyte) and CC1 (a marker of mature oligodendrocyte) staining of the brain sections from Veh- or A36-treated WT and R6/2 mice. Scale bar: 100 µm. Quantification of the number of Oligo2+ oligodendrocytes and CC1+ mature oligodendrocytes per area is shown in histogram. n = 4 mice each group. (**H**) Representative images of MBP (a marker of myelin) staining of the brain sections from Veh- or A36-treated WT and R6/2 mice. Scale bar: 200 µm. Quantification of MBP intensity per area is shown in histogram. n = 4 mice per group. (**I**) Quantification of the calpain-2 density in CAST immunoprecipitates is shown in histogram. n = 3 mice per group. The representative immunoprecipitation blots were shown in Fig. 5J. Quantitative data for all panels are presented as means ± SEM. Data are compared by one-way ANOVA with Tukey's *post-hoc* test.

Figure S7

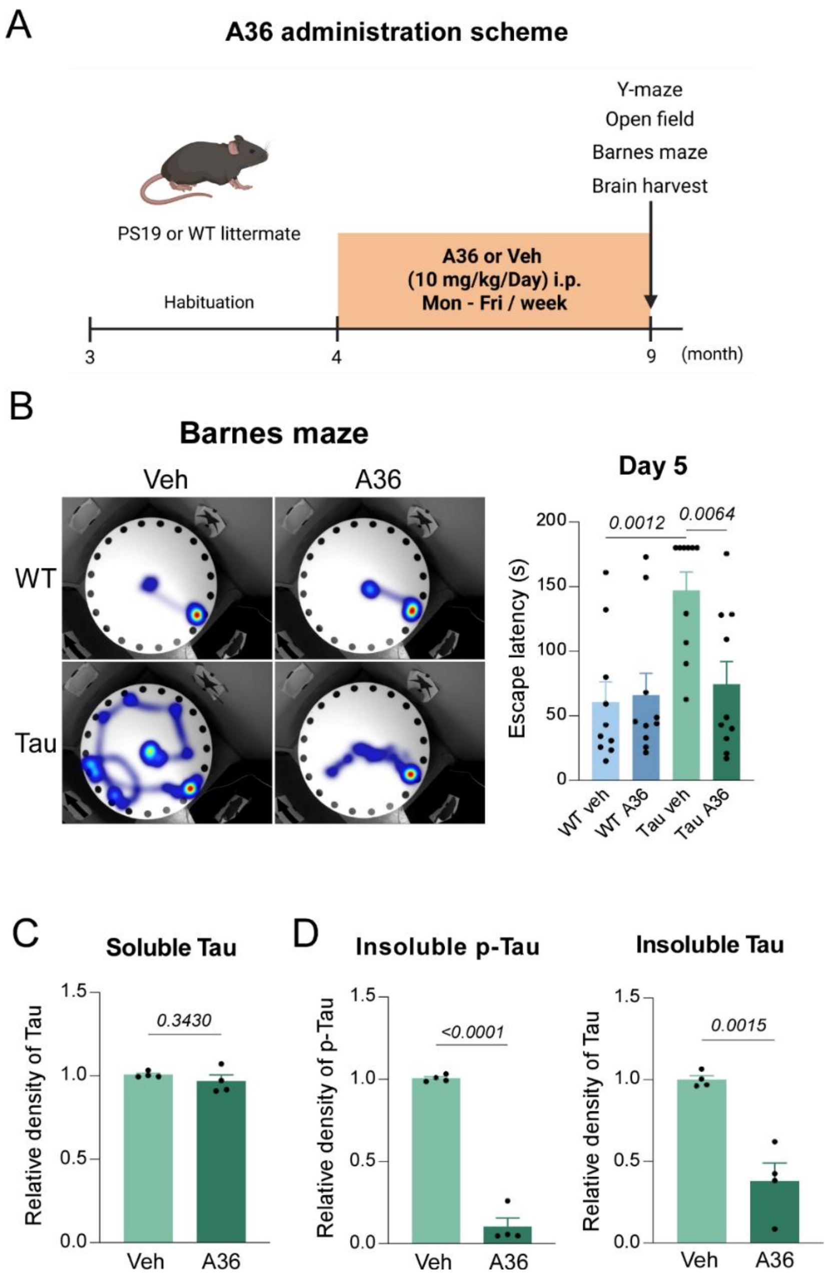

Fig. S7. A36 treatment mitigates tauopathy and cognitive deficits in the PS19 mouse model. (**A**) Experimental scheme of A36 administration in PS19 (Tau) mouse model. (**B**) Results of Barnes maze test on Day 5. Shown are the representative heat-map tracking the movements of the mice at age of 9-month. Histograms summarize the escape latency. n = 10-12 mice per group. (**C**) Quantification of the level of soluble Tau protein in hippocampal fractions from PS19 mice treated with Veh or A36. n = 4 mice per group. Representative blots are shown in Fig. 6J. (**D**) Quantification of the level of insoluble Tau and phosphorylated Tau (AT8) (p-Tau) in hippocampal fractions from PS19 mice treated with Veh or A36. n = 4 mice per group. Representative blots are shown in Fig. 6L. All data are expressed as the mean ± SEM from three independent experiments. Data are compared by one-way ANOVA with Tukey's *post-hoc* test in B, and unpaired student's t-test in C and D.

Table S1

#	Structure	TMRM Fold-change[a]	MTT Fold-change[b]	GSK3 binding	#	Structure	TMRM Fold-change[a]	MTT Fold-change[b]	GSK3 binding
1	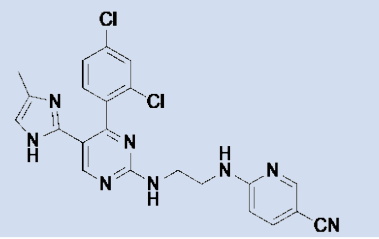	7.4	1.3	X	9	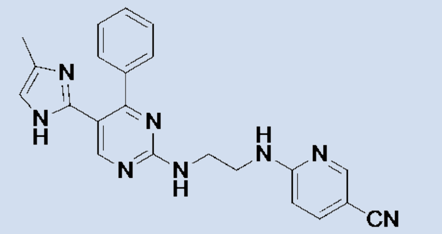	0.7	n.t.	n.t.
2	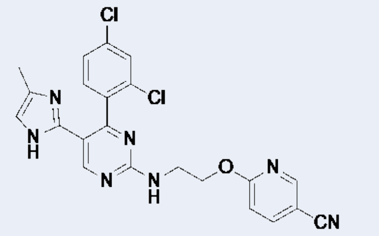	13	0.9	n.t.	10	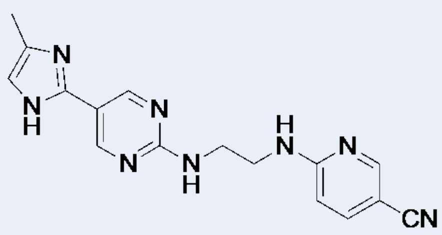	1.7	n.t.	n.t.
3	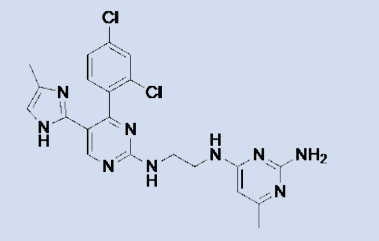	1.7	n.t.	n.t.	11	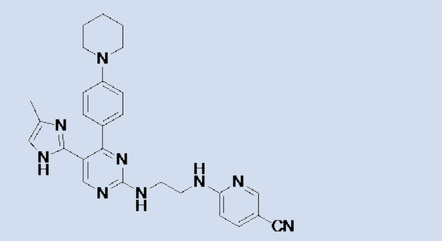	33	1.3	yes[c,d]
4	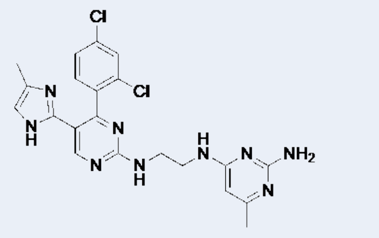	0.3	n.t.	n.t.	12	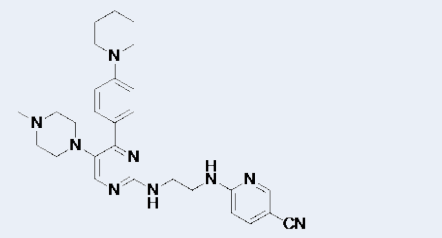	35	1.3	yes[c]
5	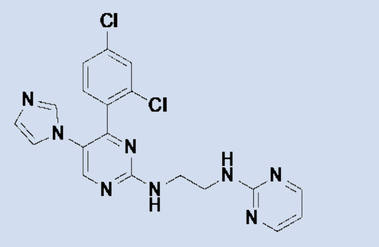	0.2	n.t.	n.t.	13	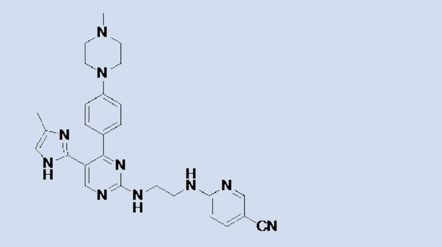	2.8	0.9	n.t.
6	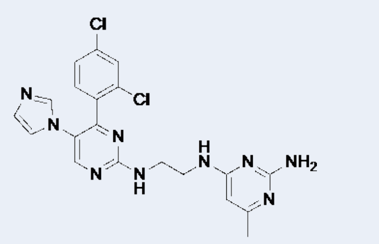	0.2	n.t.	n.t.	14	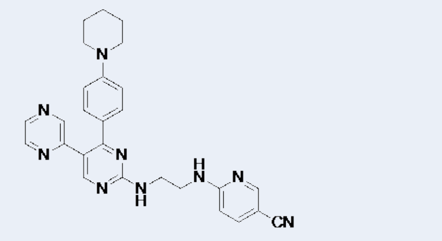	31	0.9	n.t.
7	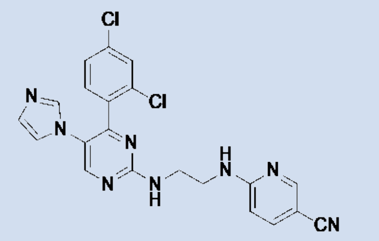	10	1.2	Yes[c,d]	15	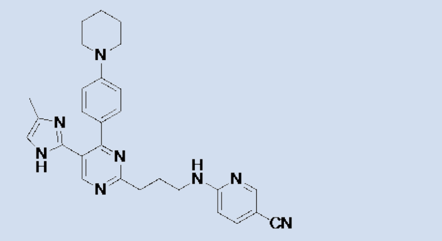	1.7	n.t.	n.t.
8	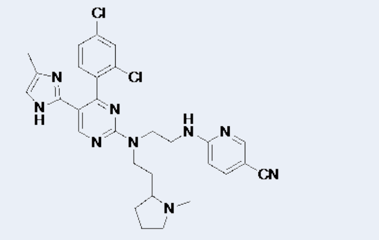	0	n.t.	n.t.	16	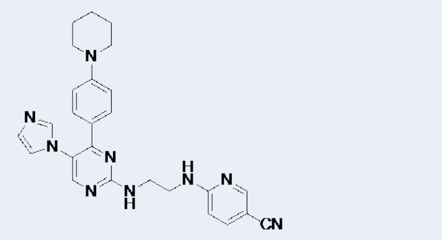	5.6	0.8	n.t.
					17	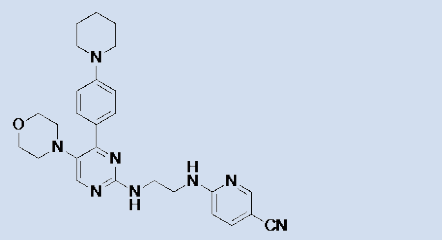	1.6	n.t.	n.t.

[a]See Figure S1A for compiled data for all analogs. [b]See Figure S1B for compiled data for all analogs. [c]CETSA assay used to monitor cellular stabilization of GSK3; See supporting file 3 for compiled data for all analogs. [d]Enzyme inhibition assay for GSK3β; see supporting file 4.

Table S2

#	Structure	TMRM Fold-change[a]	MTT Fold-change[b]	GSK3 binding	#	Structure	TMRM Fold-change[a]	MTT Fold-change[b]	GSK3 binding
**18**	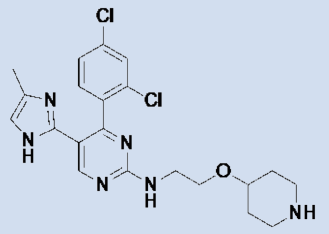	0.9	n.t.	n.t.	**25**	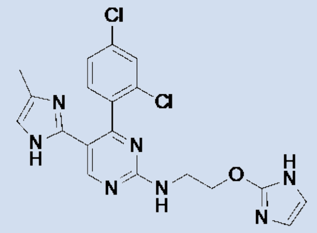	0.4	n.t.	n.t.
**19**	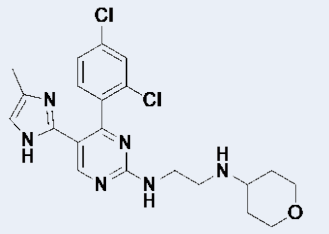	1.7	n.t.	n.t.	**26**	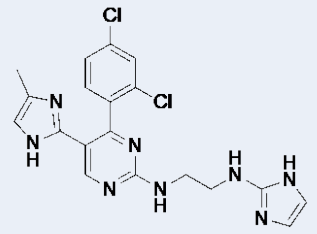	0.4	n.t.	n.t.
**20**	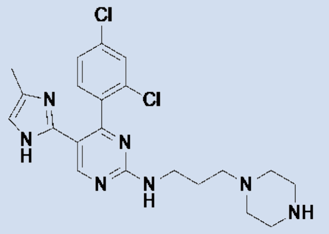	1.0	n.t.	n.t.	**27**	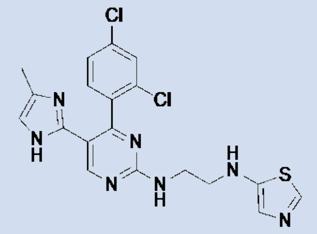	0.5	n.t.	n.t.
**21**	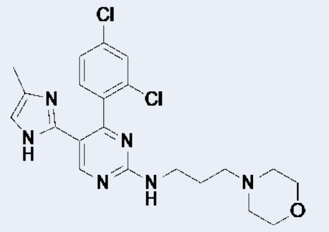	0.4	n.t.	n.t.	**28**	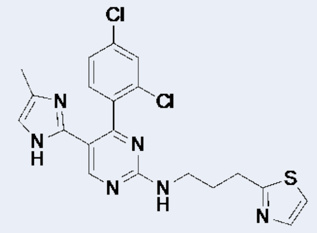	2.4	0.9	n.t.
**22**	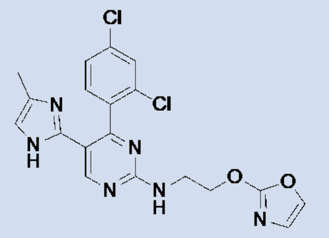	0.3	n.t.	n.t.	**29**	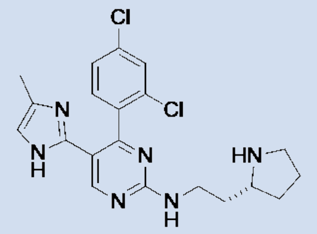	1.3	1.3	n.t.
**23**	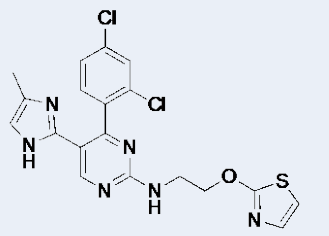	0.7	n.t.	n.t.	**30**	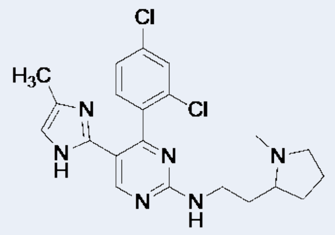	1.7	1.4	n.t.
**24**	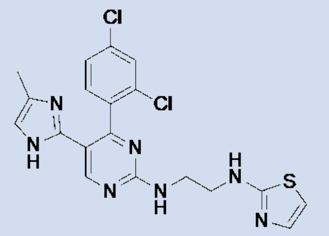	1.8	0.8	n.t.	**31**	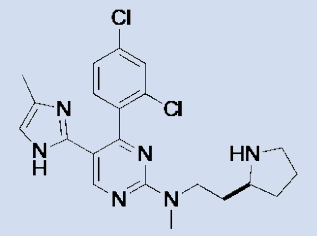	0.3	n.t.	n.t.

[a]See Figure S1C for compiled data for all analogs. [b]See Figure S1D for compiled data for all analogs.

Table S3

#	Structure	TMRM Fold-change[a]	MTT Fold-change[b]	GSK3 binding	#	Structure	TMRM Fold-change[a]	MTT Fold-change[b]	GSK3 binding
32	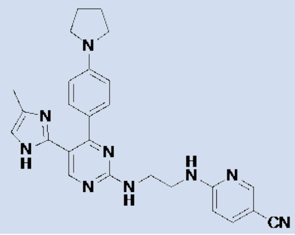	39	1.0	n.t.	36	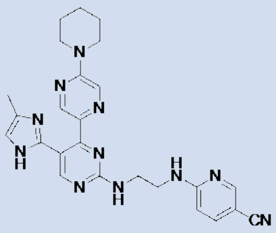	3.4	1.0	n.t.
33	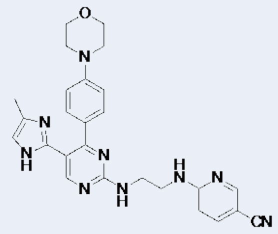	0.5	n.t.	n.t.	37	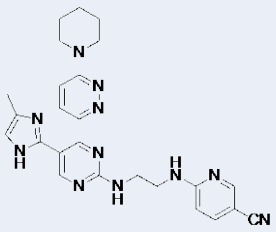	11	0.8	n.t.
34	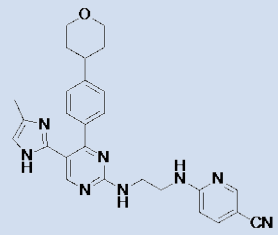	0.3	n.t.	n.t.	38	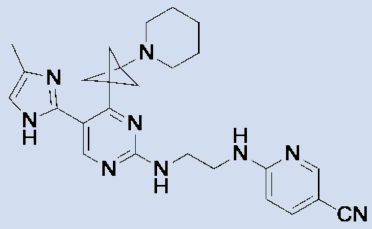	0.9	n.t.	n.t.
35	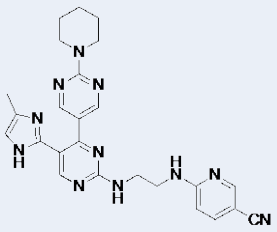	6.8	0.8	n.t.	39	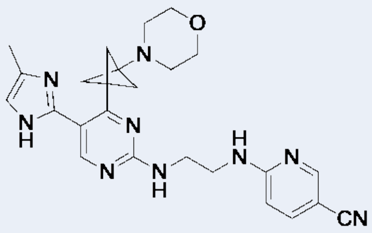	1.4	n.t.	n.t.
					40	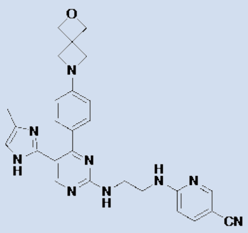	0.6	n.t.	n.t.

[a]See Figure S1E for compiled data for all analogs. [b]See Figure S1F for compiled data for all analogs.

Table S4

#	Structure	TMRM Fold-change[a]	MTT Fold-change[b]	GSK3 binding	#	Structure	TMRM Fold-change[a]	MTT Fold-change[b]	GSK3 binding
**41**	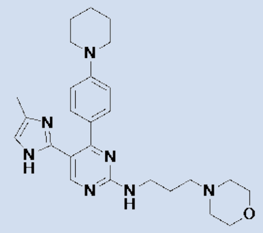	4.5	1.3	No[c]	**45**	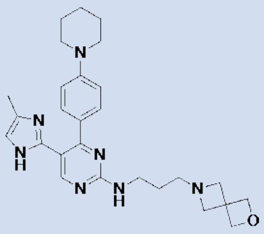	1.7	n.t.	n.t.
**42**	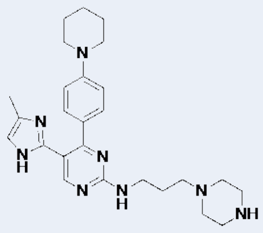	0.4	n.t.	n.t.	**46**	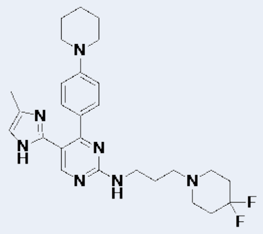	11	1.4	No[c]
**43**	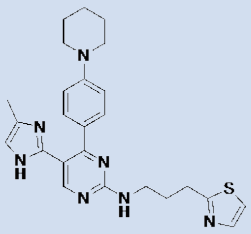	9.7	0.9	n.t.	**47**	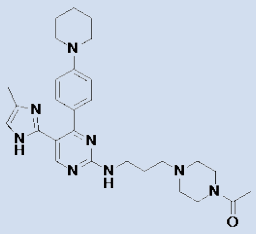	2.2	1.0	n.t.
**44**	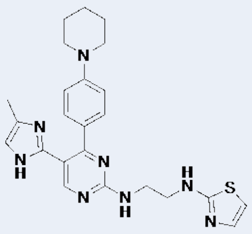	30	1.1	n.t.	**48**	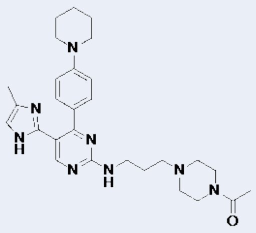	10	0.8	n.t.

[a]See Figure S1G for compiled data for all analogs. [b]See Figure S1H for compiled data for all analogs. [c]CETSA assay used to monitor cellular stabilization of GSK3; See supporting file 3 for compiled data for all analogs.

Table S5

#	Structure	TMRM Fold-change[a]	MTT Fold-change[b]	GSK3 binding	#	Structure	TMRM Fold-change[a]	MTT Fold-change[b]	GSK3 binding
49	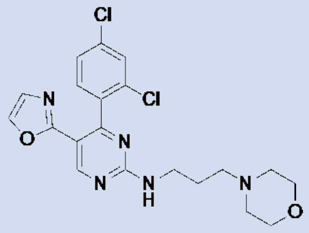	0.3	n.t.	n.t.	54	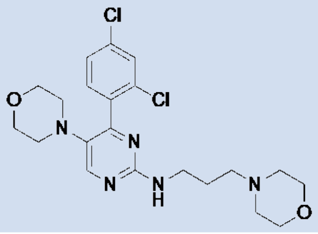	1.0	n.t.	No[d]
50	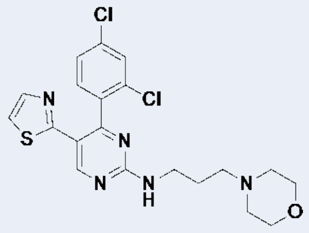	1.1	n.t.	n.t.	55	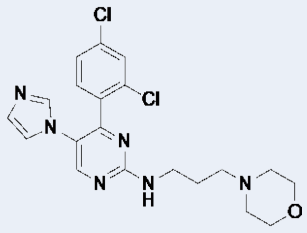	0.4	n.t.	n.t.
51	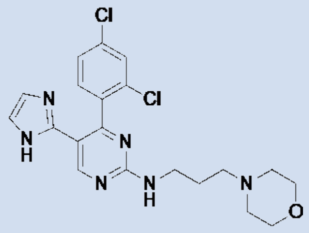	0.6	n.t.	n.t.	56	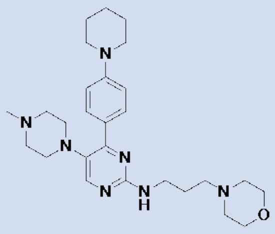	8.5	1.0	n.t.
52	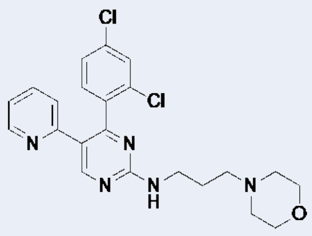	0.9	n.t.	n.t.	57	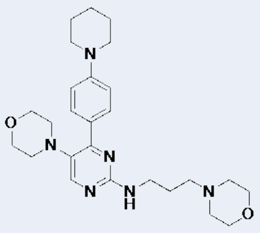	5.1	1.5	No[c]
53	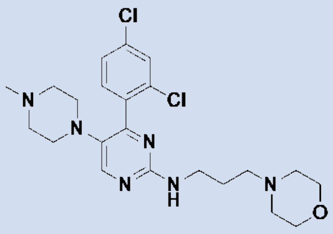	5.2	1.6	No[c,d]	58	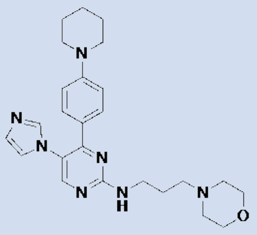	0.9	n.t.	n.t.

[a]See Figure S2A for compiled data for all analogs. [b]See Figure S2B for compiled data for all analogs. [c]CETSA assay used to monitor cellular stabilization of GSK3; See supporting file 3 for compiled data for all analogs. [d]Enzyme inhibition assay for GSK3β; see supporting file 4 for compiled data for all analogs.

Table S6

#	Structure	TMRM Fold-change[a]	MTT Fold-change[b]	GSK3 binding	#	Structure	TMRM Fold-change[a]	MTT Fold-change[b]	GSK3 binding
59	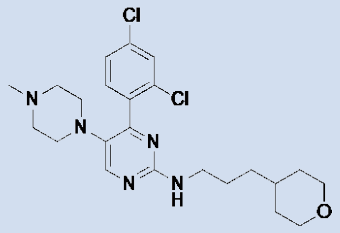	4.5	1.2	n.t.	68	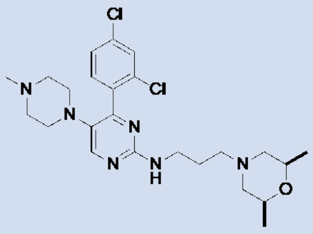	16	1.0	n.t.
60	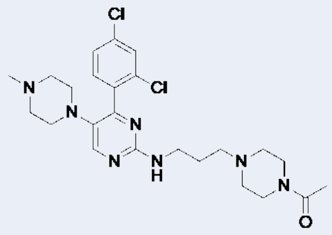	4.0	n.t.	n.t.	69	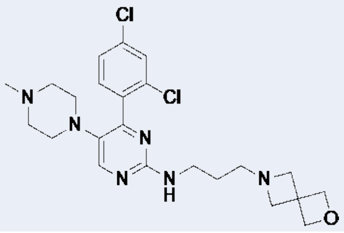	8.3	1.1	n.t.
61	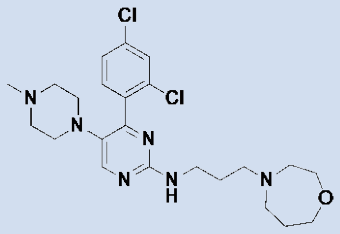	23	1.1	n.t.	70	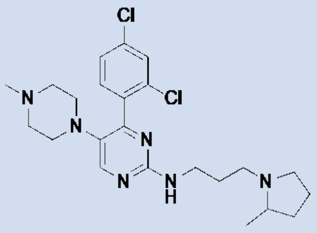	12	1.3	No[c]
62	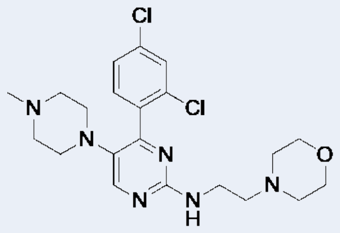	1.5	n.t.	n.t.	71	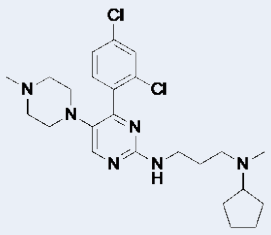	8	1.1	n.t.
63	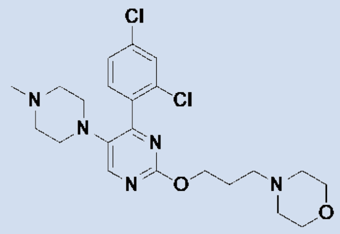	2.5	1.0	n.t.	72	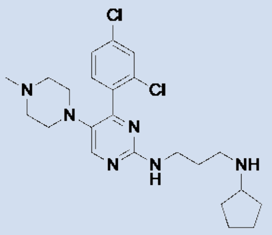	6	0.6	n.t.
64	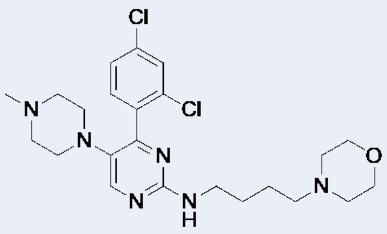	6.7	1.3	No[c]	73	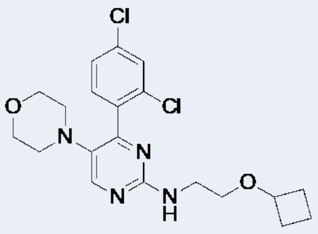	0.24	n.t.	n.t.
65	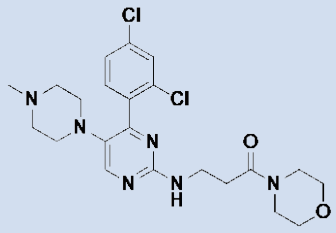	0.4	n.t.	n.t.	74	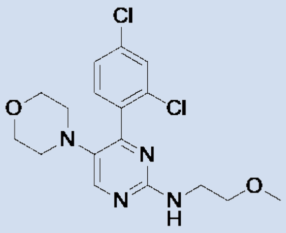	101	n.t.	n.t.
66	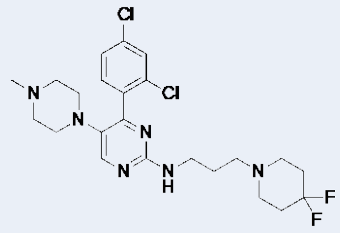	6.7	1.3	No[c]	75	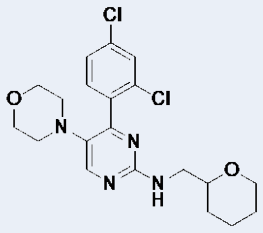	1.01	n.t.	n.t.
67	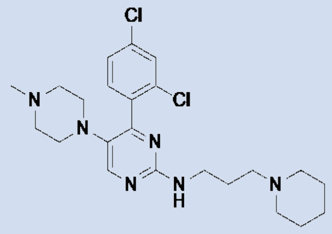	7	1.3	n.t.	76	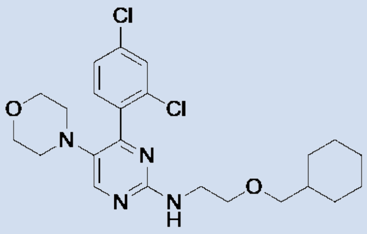	1.01	n.t.	n.t.

[a]See Figure S2C for compiled data for all analogs. [b]See Figure S2D for compiled data for all analogs. [c]CETSA assay used to monitor cellular stabilization of GSK3; See supporting file 3 for compiled data for all analogs.

Table S7

#	Structure	TMRM Fold-change[a]	MTT Fold-change[b]	GSK3 binding	#	Structure	TMRM Fold-change[a]	MTT Fold-change[b]	GSK3 binding
77	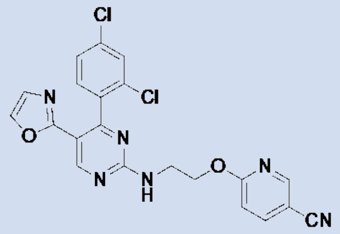	0.2	n.t.	n.t.	87	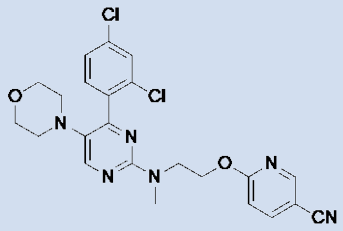	0.2	n.t.	n.t.
78	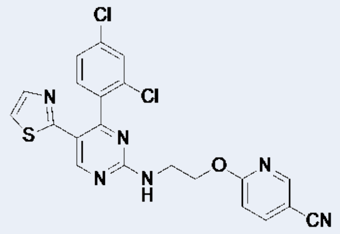	0.3	n.t.	n.t.	88	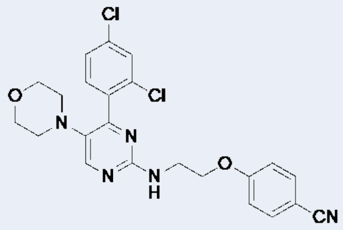	0.4	n.t.	n.t.
79	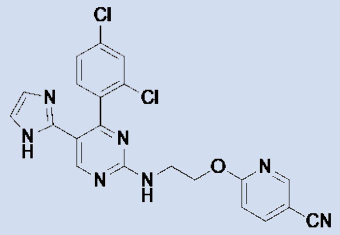	1.3	n.t.	n.t.	89	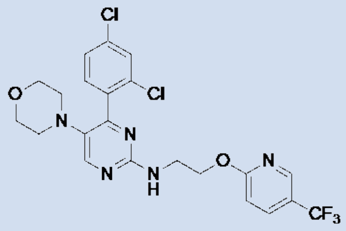	1.0	n.t.	n.t.
80	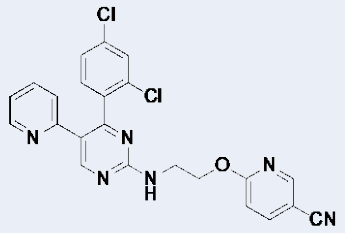	0.5	n.t.	n.t.	90	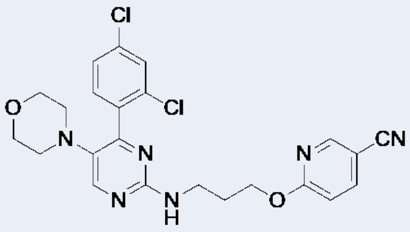	2.8	1.1	n.t.
81	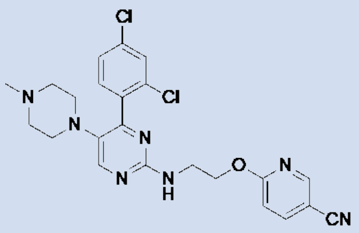	0.6	n.t.	n.t.	91	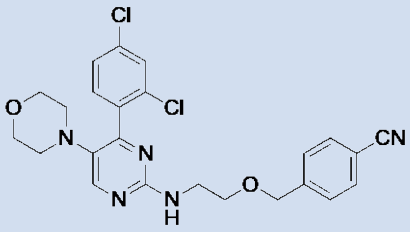	0.9	n.t.	n.t.
82	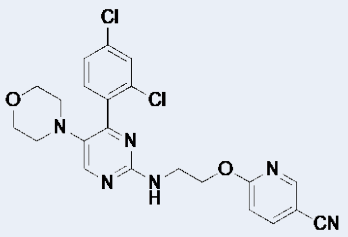	24	1.32	No[c,d]	92	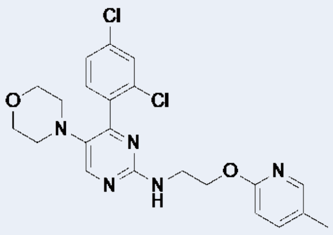	0.4	n.t.	n.t.
83	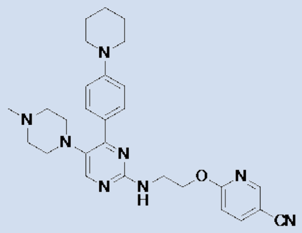	40	1.5	No[c]	93	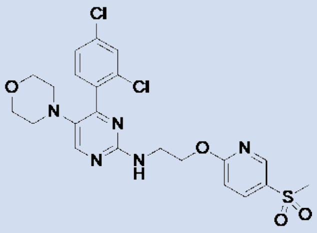	0.03	n.t.	n.t.
84	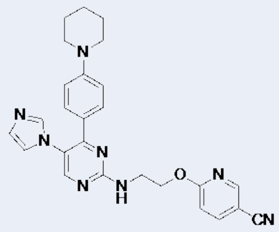	1.0	n.t.	n.t.	94	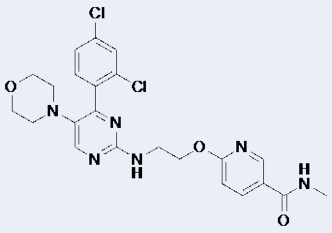	0.03	n.t.	n.t.
85	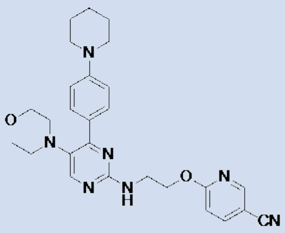	5.0	1.06	n.t.	95	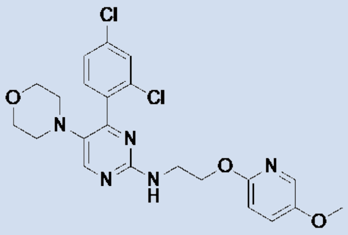	0.3	0.8	n.t.
86	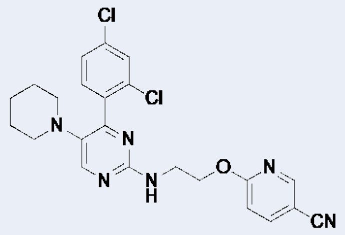	0.7	n.t.	n.t.	96	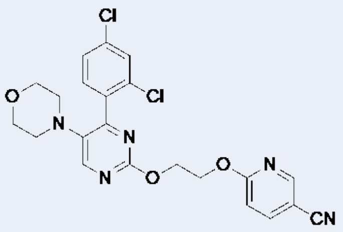	0.1	n.t.	n.t.

[a]See Figure S2E for compiled data for all analogs. [b]See Figure S2F for compiled data for all analogs. [c]CETSA assay used to monitor cellular stabilization of GSK3; See supporting file 3 for compiled data for all analogs. [d]Enzyme inhibition assay for GSK3β; see supporting file 4 for compiled data for all analogs.

Table S8

#	Structure	TMRM Fold-change[a]	MTT Fold-change[b]	GSK3 binding	#	Structure	TMRM Fold-change[a]	MTT Fold-change[b]	GSK3 binding
97	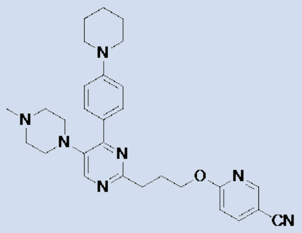	3.4	1.0	n.t.	106	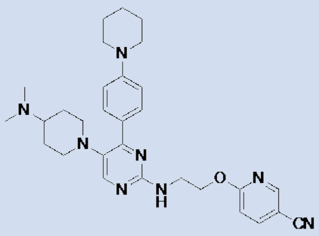	81	0.04	n.t.
98	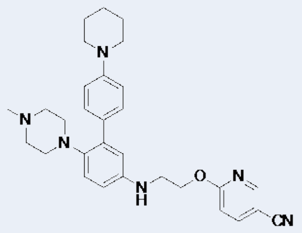	15	1.2	No[c]	107	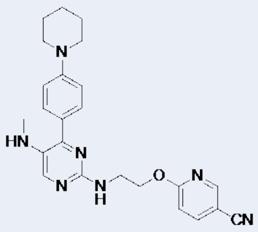	2.4	0	n.t.
99	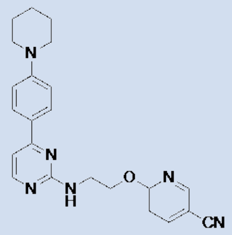	1.5	n.t.	n.t.	108	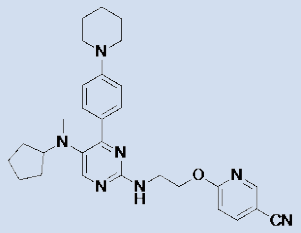	24	0.5	n.t.
100	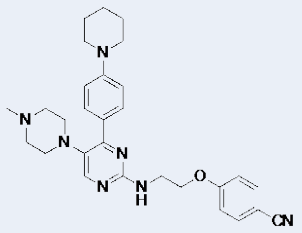	41	1.3	No[c]	109	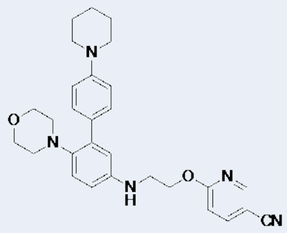	1.0	n.t.	n.t.
101	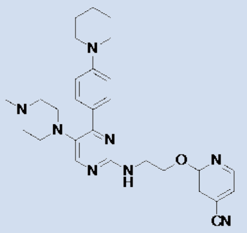	58	1.4	No[c]	110	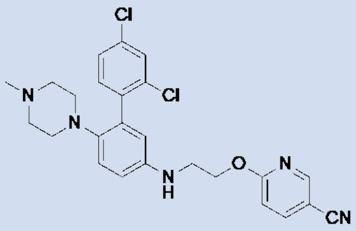	7.7	1.1	n.t.
102	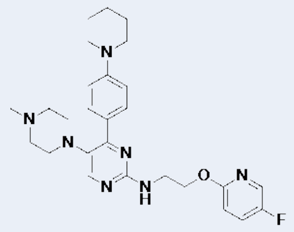	21	1.4	No[c]	111	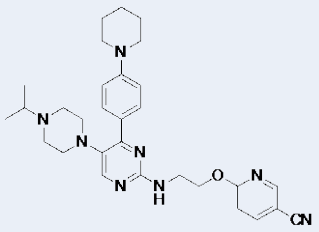	48	1.3	n.t.
103	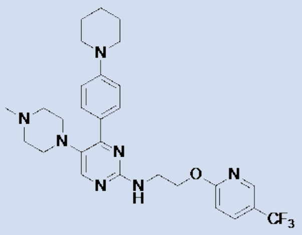	45	1.5	No[c]	112	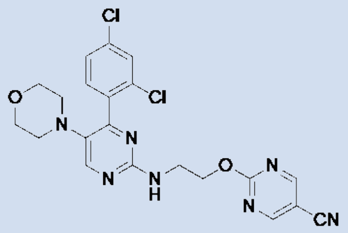	1.0	n.t.	n.t.
104	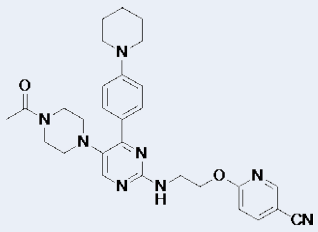	21	1.0	n.t.	113	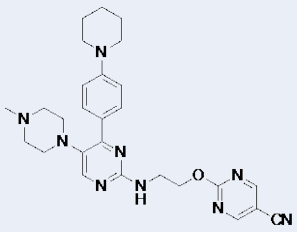	18	1.2	n.t.
105	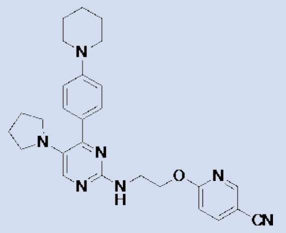	31	1.0	n.t.	114	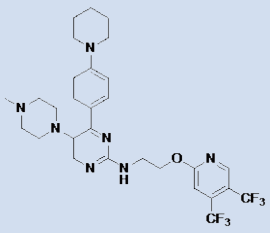	0	n.t.	n.t.
					115	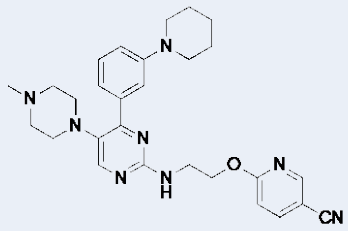	1.0	n.t.	n.t.

[a]See Figure S2G for compiled data for all analogs. [b]See Figure S2H for compiled data for all analogs. [c]CETSA assay used to monitor cellular stabilization of GSK3; See supporting file 3 for compiled data for all analogs.

Supporting information 1 – Analog synthesis procedues

Analog 2

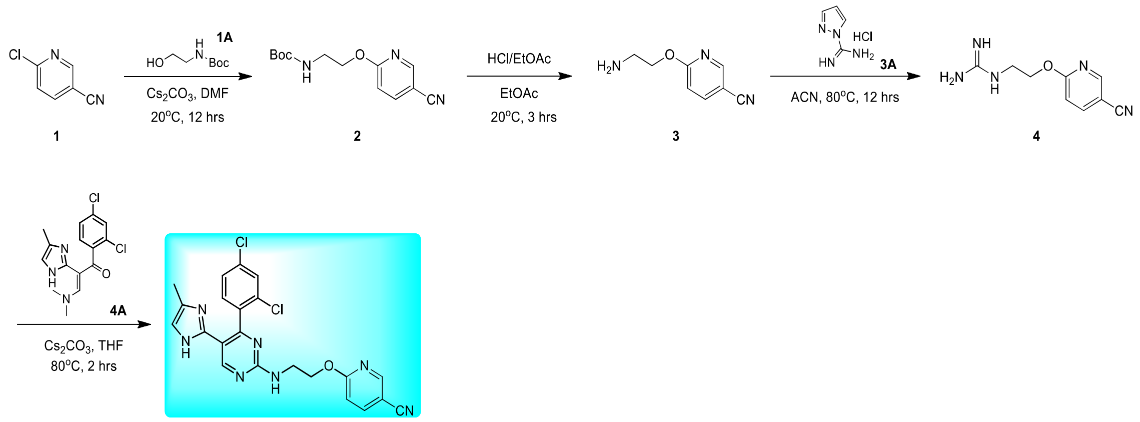

Analog 2

6-(2-((4-(2,4-dichlorophenyl)-5-(4-methyl-1*H*-imidazol-2-yl)pyrimidin-2-yl)amino)ethoxy)nicotinonitrile (Analog 2). To a solution of **Intermediate 1A** (4.07 g, 25.26 mmol, 3.92 mL) and Cs_2CO_3 (5.88 g, 18.04 mmol) in DMF (10 mL) was added **Intermediate 1** (1 g, 7.22 mmol). Then the mixture was stirred at 20°C for 12 hrs. LC-MS showed **Intermediate 1** was consumed completely and one main peak with desired mass was detected. The reaction was diluted with 20 mL H_2O and extracted with EtOAc 10 mL × 3. The combined organic layers were washed with brine 20 mL, dried over Na_2SO_4, filtered and concentrated under reduced pressure to give a residue. The residue was purified by flash silica gel chromatography (ISCO®; 12 g SepaFlash® Silica Flash Column, Eluent of 0~10% Ethyl acetate/Petroleum ether gradient @ 100 mL/min) (Petroleum ether: Ethyl acetate= 1:1, P1Rf=0.63) to afford ***Intermediate 2*** (1.1 g, 4.18 mmol, 57.89% yield) as a white solid. MS-ESI (m/z) calcd for $C_{13}H_{17}N_3O_3$ $[M+H]^+$: 264.1. Found 264.2. To a solution of **Intermediate 2** (600 mg, 2.28 mmol) in EtOAc (5 mL) was added HCl/EtOAc (4 M, 8.40 mL). The mixture was stirred at 20°C for 3 hrs. LC-MS showed **Intermediate 2** was consumed and one main peak with mass was detected. The reaction mixture was filtered and the filtered cake was dried under vacuum to afford ***Intermediate 3***(450 mg, crude, HCl salt) as a white solid. MS-ESI (m/z) calcd for $C_8H_9N_3O$ $[M+H]^+$: 164.1. Found 164.2. To a solution of **Intermediate 3** (300 mg, 1.50 mmol, HCl salt) in ACN (2 mL) was added **Intermediate 3A** (220.27 mg, 1.50 mmol, HCl salt) at 20°C. Then the mixture was stirred at 80°C for 12 hrs. LC-MS showed **Intermediate 3** was consumed completely and one main peak with desired mass was detected. The reaction mixture was concentrated under reduced pressure to remove solvent to afford ***Intermediate 4*** (500 mg, crude, HCl salt) as a pale yellow solid. MS-ESI (m/z) calcd for $C_9H_{11}N_5O$ $[M+H]^+$: 206.1. Found 206.1. To a solution of **Intermediate 4** (90 mg, 372.40 umol, HCl salt) in THF (2 mL) was added Cs_2CO_3 (364.00 mg, 1.12 mmol), **Intermediate 4A** (120.73 mg, 372.40 umol) at 20°C. Then the mixture was stirred at 80°C for 2 hrs. LC-MS showed **Intermediate 4A** was consumed completely and one main peak with desired mass was detected. The reaction mixture was filtered to remove the insoluble and the filtrate was concentrated under reduced pressure to remove solvent. The residue was purified by prep-HPLC (neutral condition) (column: Waters Xbridge Prep OBD C18 150*40mm*10um; mobile phase: [water (10mM NH_4HCO_3)-ACN]; B%: 30%-60%, 8min) to afford **Analog 2** (22.81 mg, 97.18% purity) as a light yellow solid. ^1H NMR (400 MHz, CD_3OD): δ

ppm 8.51 (br d, *J*=6.60 Hz, 2 H) 7.91 (br s, 1 H) 7.30 - 7.56 (m, 3 H) 6.90 (br s, 1 H) 6.66 (br s, 1 H) 4.62 (t, *J*=5.32 Hz, 2 H) 3.88 (t, *J*=5.32 Hz, 2 H) 2.16 (s, 3 H). **LCMS** (ESI+): *m/z* 466.1/468.1 (M+H)

Analog 3

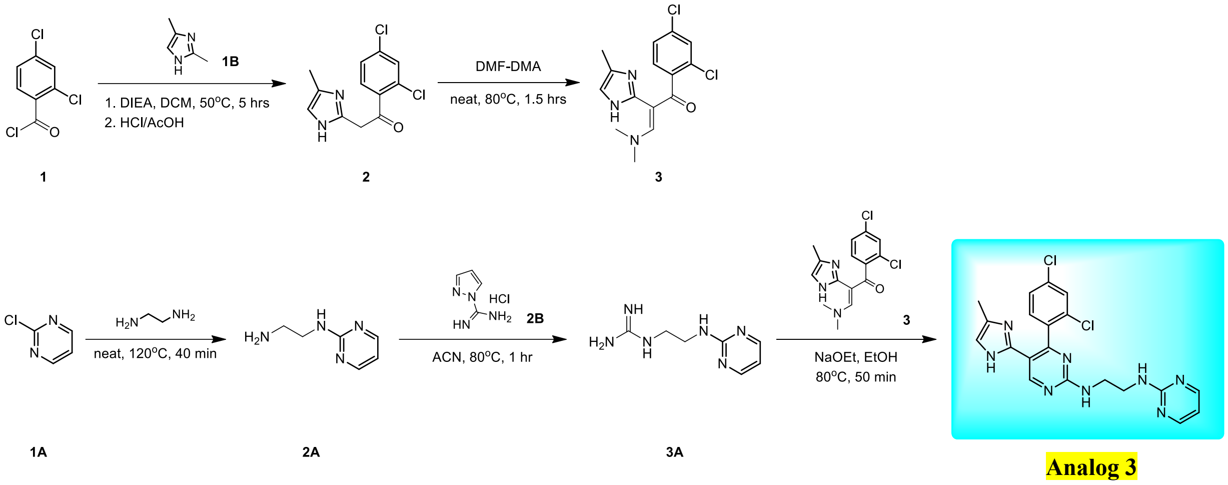

Analog 3

N*[1]-(4-(2,4-dichlorophenyl)-5-(4-methyl-1*H*-imidazol-2-yl)pyrimidin-2-yl)-*N*[2]-(pyrimidin-2-yl)ethane-1,2-diamine (Analog 3).** To a solution of **Intermediate 1B** (2.8 g, 29.13 mmol) in DCM (45 mL) was heated to 40°C until the solution was clear, then DIEA (12.05 g, 93.21 mmol, 16.23 mL) was added in one portion at 30 °C, another solution of **Intermediate 1** (18.42 g, 87.96 mmol, 12.28 mL) in DCM (8.5 mL) was added drop wised under water bath (keep the temperature below 35°C), the reaction was stirred at 50°C for 5 hrs. Then the reaction was concentrated under vacuum until the solid was dry. The dry solid was added to a solution of AcOH (38.6 mL) and HCl (19.3 mL) (12 M), and then the mixture was stirred at 125°C for 3 hrs. The LCMS showed the **Intermediate 1B** was consumed and there was 41% of desired product was detected. The reaction was concentrated under vacuum. Then water (96.6 mL) and toluene (48.1 mL) were added to the liquid, and then the mixture was vigorously stirred for 30 min at 20°C. Solid appeared and was filtered, rinsed with 23.8 mL of water and the solid was discared. The filtrate was transferred into a separatory funnel, the organic layer was discarded and the aqueous layer was washed with toluene (47.6 mL×2), the aqueous layer was transfered into a large breaker and diluted with MTBE (23.8 mL), then the stirred mixture was basified with saturated $NaHCO_3$ solution to pH=8. DCM (96.6 mL) was added and stirred for 10 min at 20°C, the organic phase was separated and the aqueous layer was extracted with DCM (47.6 mL×1), the organic layers were combined and washed with saturated $NaHCO_3$ solution (47.6 mL), water (47.6 mL) and brine (47.6 mL), dried over Na_2SO_4, filtered and the filtrate was concentrated under vacuum to afford ***Intermediate 2 (3.37 g, 9.02 mmol, 30.95% yield, 72% purity) as a yellow solid. MS-ESI (m/z) calcd for $C_{12}H_{10}$ Cl_2N_2O $[M+H]^+$: 269.0/271.0. Found 269.2/271.2. A mixture of **Intermediate 2** (1.87 g, 6.95 mmol) in DMF-DMA (20 mL) was stirred at 80°C for 1.5 hrs. LCMS showed the **Intermediate 2** was consumed and there was 33% of desired product was detected. The reaction was concentrated under vacuum. Then the residue was diluted with EtOAc (10 mL × 2) and ACN (10 mL), the mixture was concentrated under vacuum to afford ***Intermediate 3*** (2.6 g, crude) as a brown liquid and it was used directly. MS-ESI (m/z) calcd for $C_{15}H_{15}Cl_2N_3O$ $[M+H]^+$: 324.1/326.1. Found 324.2/326.2. A mixture of **Intermediate 1A** (4 g, 34.92 mmol) in ethane-1,2-diamine (35.92 g, 597.69 mmol, 40.00 mL) was stirred at 120°C for 40 min. LCMS

showed the **Intermediate 1A** was consumed and there was 45% of desired product was detected (Petroleum ether: Ethyl acetate=1:1) (P1 Rf=0.00). The reaction was diluted with brine (50 mL) and extracted with EtOAc (50 mL × 3), the organic layer was washed with brine (30 mL), dried over anhydrous Na_2SO_4, filtered and the filtrate was concentrated under vacuum to afford ***Intermediate 2A*** (722 mg, crude)(87% purity) as a yellow oil. MS-ESI (m/z) calcd for $C_6H_{10}N_4$ $[M+H]^+$: 139.1. Found 138.9. To a solution of **Intermediate 2A** (722 mg, 5.23 mmol) in ACN (8 mL) was added **Intermediate 2B** (765.94 mg, 5.23 mmol), then the mixture was stirred at 80°C for 1 hr. LCMS showed the **Intermediate 2A** was consumed and there was 61% of desired product was detected. The reaction was concentrated under vacuum to afford ***Intermediate 3A*** (1.2 g, crude, HCl salt) as a yellow solid, which was used directly. MS-ESI (m/z) calcd for $C_7H_{12}N_6$ $[M+H]^+$: 181.1. Found 181.2 To a solution of **Intermediate 3A** (195.82 mg, 903.75 umol, HCl salt) in EtOH (2 mL) was added EtONa (61.50 mg, 903.75 umol) at 20°C, then the mixture was stirred at 80°C for 10 min, then another solution of **Intermediate 3** (293 mg, 903.75 umol) in EtOH (1 mL) was added at 80°C, then the mixture was stirred at 80°C for 40 min. LCMS showed the **Intermediate 3** was consumed and there was 39% of desired product was detected. The reaction was concentrated under vacuum and purified by Prep-HPLC (HCl condition) (column: Phenomenex luna C18 80*40mm*3 um; mobile phase: [water (0.04%HCl)-ACN]; B%: 8%-30%, 7 min), the HNMR was not clean, so the residue was further purified by Prep-HPLC (basic condition)(column: Phenomenex Gemini-NX C18 75*30mm*3um; mobile phase: [water (0.05%$NH_3.H_2O$+10mM NH_4HCO_3)-ACN]; B%: 20%-60%, 8 min) to afford **Analog 3** (16.98 mg, 38.33 umol, 4.24% yield, 99.61% purity) as a white solid. **^{1}H NMR** (METHANOL-d_4 400MHz) δ ppm 8.49 (br s, 1 H) 8.22 (br d, J=4.52 Hz, 2 H) 7.34 - 7.44 (m, 3 H) 6.63 (s, 1 H) 6.56 (br s, 1 H) 3.65 - 3.72 (m, 2 H) 3.62 (br d, J=5.38 Hz, 2 H) 2.13 (s, 3 H). **LCMS** (ESI+): *m/z* 441.1/443.1 (M+H)

Analog 4

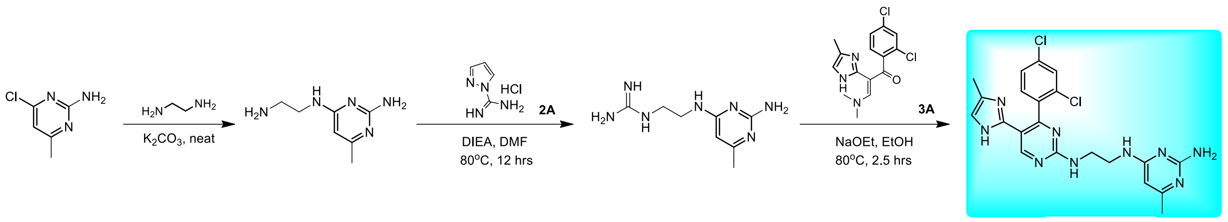

Analog 4

N*[4]-(2-((4-(2,4-dichlorophenyl)-5-(4-methyl-1*H*-imidazol-2-yl)pyrimidin-2-yl)amino)ethyl)-6-methylpyrimidine-2,4-diamine (Analog 4).** A mixture of **Intermediate 1** (1.5 g, 10.45 mmol) in ethane-1,2-diamine (13.47 g, 224.14 mmol, 15.00 mL) was stirred at 20°C for 1 hr, then K_2CO_3 (4.33 g, 31.34 mmol) was added, and the resulting mixture was stirred at 50°C for 12 hrs. LC-MS showed **Intermediate 1** was consumed completely and one main peak with desired mass was detected. The reaction mixture was filtered and the filtered cake was washed with DMF (10 mL × 3), then the filtrate was concentrated under reduced pressure to remove **solvent**. The residue was triturated in 20 mL MTBE for 10 min, then the mixture was filtered and the cake was dried under reduced pressure to afford ***Intermediate 2 (2 g, crude) as a white solid. MS-ESI (m/z) calcd for $C_7H_{13}N_5$ $[M+H]^+$: 168.1. Found 168.2. To a solution of **Intermediate 2** (200 mg, 1.20 mmol) and **Intermediate 2A** (175.32 mg, 1.20 mmol) in DMF (2 mL) was added DIEA (463.76 mg, 3.59 mmol, 625.01 uL) at 20°C. Then the mixture was stirred at 80°C for 12 hrs. LC-MS showed **Intermediate 2** was consumed completely and one main peak with desired mass was detected. The reaction mixture was concentrated under reduced pressure to afford ***Intermediate 3*** (300 mg, crude) as a yellow oil. MS-ESI (m/z) calcd for $C_8H_{15}N_7$ $[M+H]^+$: 210.1. Found 210.0. To a solution of **Intermediate 3** (250 mg, 1.19 mmol) in EtOH (2 mL) was added NaOEt (81.30 mg, 1.19 mmol) and **Intermediate 3A** (387.34 mg, 1.19 mmol). The mixture was stirred at 80°C for 1 hr. LC-MS showed Reactant 1 was consumed completely and 21% desired product was detected. The reaction mixture was concentrated under reduced pressure to remove solvent. The residue was purified by Prep-HPLC (neutral condition, column: Phenomenex Gemini-NX C18 75*30mm*3um; mobile phase: [water (10mM NH_4HCO_3)-ACN]; B%: 25%-45%, 8min). But LCMS showed that the purity was not high enough, so the residue was purified further together with another batch by Prep-HPLC (HCl condition) to afford **Analog 4** (30 mg, 98.18% purity, HCl salt) as a yellow solid. **^{1}H NMR** (METHANOL-d_4 400MHz) δ ppm 8.67 (br s, 1 H) 7.47 - 7.65 (m, 3 H) 7.22 (s, 1 H) 5.72 - 6.02 (m, 1 H) 3.55 - 3.90 (m, 4 H) 2.10 - 2.34 (m, 6 H). **LCMS** (ESI+): *m/z* 470.1/472.1 (M+H)

Analogs 5, 6, 7

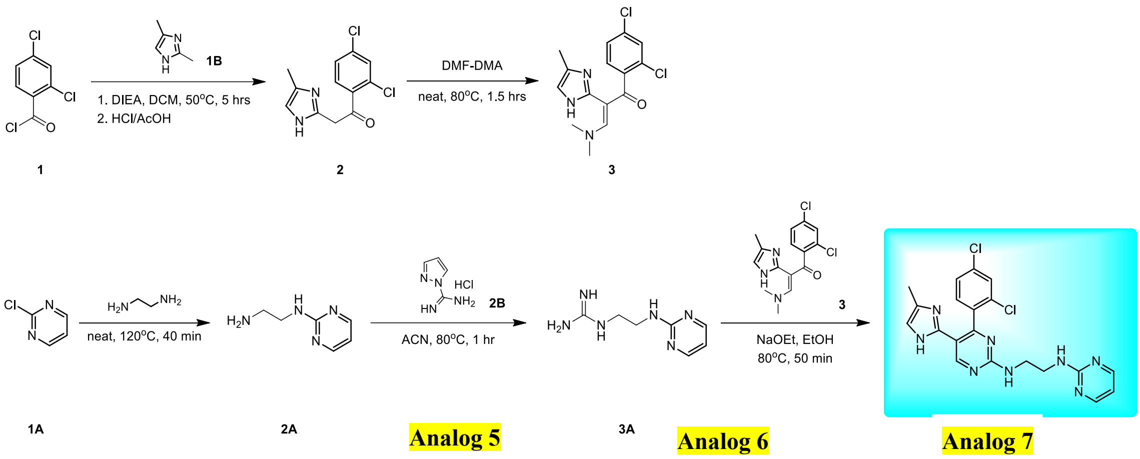

N*[1]-(4-(2,4-dichlorophenyl)-5-(1*H*-imidazol-1-yl)pyrimidin-2-yl)-*N*[2]-(pyrimidin-2-yl)ethane-1,2-diamine (Analog 5); *N*[4]-(2-((4-(2,4-dichlorophenyl)-5-(1*H*-imidazol-1-yl)pyrimidin-2-yl)amino)ethyl)-6-methylpyrimidine-2,4-diamine (Analog 6); 6-((2-((4-(2,4-dichlorophenyl)-5-(1*H*-imidazol-1-yl)pyrimidin-2-yl)amino)ethyl)amino)nicotinonitrile (Analog 7).** A mixture of **Intermediate 1** (3 g, 11.20 mmol), **Intermediate 1A** (2.29 g, 33.59 mmol) and K_2CO_3 (4.64 g, 33.59 mmol) in DMF (30 mL) was stirred at 20°C for 2 hrs under N_2 atmosphere. TLC indicated one major new spot with larger polarity was detected (SiO_2, Petroleum ether: Ethyl acetate=0:1, P1Rf=0.15). The reaction mixture was diluted with H_2O 40 mL and extracted with EtOAc 10 mL×3. The combined organic layers were washed with brine 20 mL, dried over Na_2SO_4, filtered and concentrated under reduced pressure to give a residue. The residue was purified by flash silica gel chromatography (ISCO®; 20 g SepaFlash® Silica Flash Column, Eluent of 0~100% Ethyl acetate/Petroleum ether gradient @ 100 mL/min) (SiO_2, Petroleum ether: Ethyl acetate=0:1, P1Rf=0.15) to afford ***Intermediate 2 (2.2 g, 8.62 mmol, 77.02% yield) as a brown gum. A mixture of **Intermediate 2** (2.8 g, 10.98 mmol) in DMFDMA (26.91 g, 225.83 mmol, 30.00 mL) was stirred at 80°C for 12 hrs. LC-MS showed **Intermediate 2** was consumed completely and one main peak with desired mass was detected. The reaction mixture was concentrated under reduced pressure to remove DMFDMA to afford ***Intermediate 3*** (4 g, crude) as a brown oil. MS-ESI (m/z) calcd for $C_{14}H_{13}Cl_2ON_3$ $[M+H]^+$: 310.0/312.0. Found 310.0/312.0. To a solution of **Intermediate 3A** (2.87 g, 14.19 mmol) and Cs_2CO_3 (13.87 g, 42.56 mmol) in THF (40 mL) was added **Intermediate 3** (4.4 g, 14.19 mmol) in THF (10 mL) at 20°C. Then the mixture was stirred at 80°C for 12 hrs. LC-MS showed **Intermediate 3** was consumed completely and one main peak with desired mass was detected. The reaction mixture was concentrated under reduced pressure to remove solvent. The residue was diluted with H_2O 50 mL and extracted with EtOAc 10 mL × 3. The combined organic layers were washed with brine 20 mL, dried over Na_2SO_4, filtered and concentrated under reduced pressure to afford ***Intermediate 4*** (4.4 g, crude) as a brown solid. MS-ESI (m/z) calcd for $C_{20}H_{22}Cl_2N_6O_2$ $[M+H]^+$: 449.1/451.1. Found 449.1/451.1. To a solution of **Intermediate 4** (1 g, 2.23 mmol) in ACN (10 mL) was added HCl (3 M, 10.00 mL) at 20°C. The mixture was stirred at 30°C for 12 hrs. LC-MS showed **Intermediate 4** was consumed completely and one main peak with desired mass was detected. The reaction mixture was concentrated under reduced pressure to remove solvent to afford ***Intermediate 5*** (900 mg, crude, HCl salt) as a yellow solid. MS-ESI (m/z) calcd for $C_{15}H_{14}Cl_2N_6$ $[M+H]^+$: 349.1/351.1. Found 349.0/351.0. To a solution of **Intermediate 5** (150 mg, 388.93 umol, HCl salt) in DMF (2 mL) was added DIEA (150.80 mg, 1.17 mmol, 203.23 uL) and **Intermediate 2A** (44.54 mg, 388.93 umol) at 20°C. The

mixture was stirred at 80°C for 12 hrs. LC-MS showed **Intermediate 5** was consumed and one main peak with desired mass was detected. The reaction mixture was filtered to remove the insoluble. The filtrate was purified by prep-HPLC (neutral condition, column: Phenomenex Gemini-NX C18 75*30mm*3um; mobile phase: [water (10mM NH_4HCO_3)-ACN]; B%: 30%-50%, 8min) to afford ***analog 5*** (10.93 mg, 99.47% purity) as a pale yellow solid.

The procedure of making ***analog 6*** & ***analog 7*** was similar with that of ***analog 5*** but used distinct chloro-arenes.

***Analog 5*: ^{1}H NMR** (METHANOL-d_4 400MHz) δ ppm 8.40 (br s, 1 H) 8.24 (br s, 2 H) 7.56 (s, 1 H) 7.46 (s, 1 H) 7.36 (d, *J*=0.86 Hz, 2 H) 6.95 (d, *J*=17.12 Hz, 2 H) 6.57 (br s, 1 H) 3.65 (br d, *J*=12.72 Hz, 4 H). **LCMS** (ESI+): *m*/*z* 427.1/429.1 (M+H)

***Analog 6*: ^{1}H NMR** (METHANOL-d_4 400MHz) δ ppm 8.42 (br s, 1 H) 7.56 (br s, 1 H) 7.47 (s, 1 H) 7.37 (d, *J*=0.86 Hz, 2 H) 6.97 (s, 1 H) 6.93 (s, 1 H) 6.57 (br s, 1 H) 5.70 (br s, 1 H) 3.65 (br d, *J*=12.72 Hz, 4 H) 2.06 (s, 3 H). **LCMS** (ESI+): *m*/*z* 456.1/458.1 (M+H)

***Analog 7*: ^{1}H NMR** (METHANOL-d_4 400MHz) δ ppm 8.15 - 8.54 (m, 2 H) 7.59 (s, 1 H) 7.50 (s, 1 H) 7.33 - 7.42 (m, 2 H) 6.97 (d, *J*=15.85 Hz, 2 H) 6.56 (br s, 1 H) 3.67 (br s, 4 H). **LCMS** (ESI+): *m*/*z* 451.1/453.1 (M+H).

Analog 8

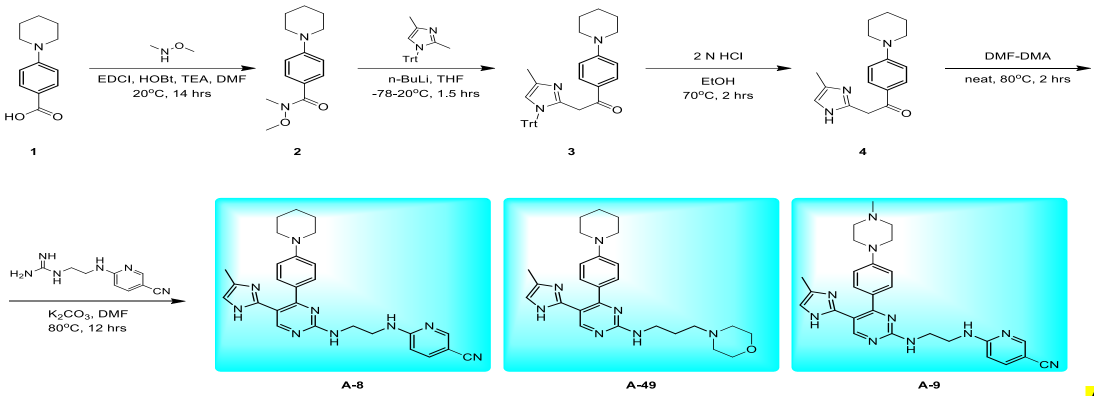

Analog 8

6-((2-((4-(2,4-dichlorophenyl)-5-(4-methyl-1*H*-imidazol-2-yl)pyrimidin-2-yl)(2-(1-methylpyrrolidin-2-yl)ethyl)amino)ethyl)amino)nicotinonitrile (Analog 8). To a solution of **Compound 1A** (5 g, 36.09 mmol) and **Compound 1** (9.25 g, 57.74 mmol, 9.07 mL) in DMF (50 mL) was added DIEA (25 mL) at 20°C. The mixture was stirred at 100°C for 2hrs. TLC (Petroleum ether / Ethyl acetate=1/1, Rf =0.42) indicated **Compound 1A** was consumed completely and two new spots was detected. The mixture was diluted with H_2O 200 mL and extracted with EtOAc (100 mL × 3). The combined organic layers were washed with brine (50 mL × 2), dried over Na_2SO_4, filtered and concentrated under reduced pressure to give a residue. The residue was purified by flash silica gel chromatography (ISCO®; 40 g SepaFlash® Silica Flash Column, Eluent of 0~23% Ethyl acetate/Petroleum ether gradient @ 100mL/min) (Petroleum ether / Ethyl acetate=1/1, Rf =0.42) to afford ***Compound 2*** (6.3 g, 24.02 mmol, 66.56% yield) as a white solid. To a solution of **Compound 2** (3 g, 11.44 mmol) in DMF (30 mL) was added NaH (503.18 mg, 12.58 mmol, 60% purity), the mixture was stirred at 20 °C for 1hr. PMB-Cl (2.15 g, 13.72 mmol, 1.87 mL) was added into the mixture, the mixture was stirred at 20°C for 1hr. LC-MS showed trace **Compound 2** was remained 14% desired compound was detected. The reaction mixture was quenched by addition H_2O 10 mL at 20°C, and then extracted with EtOAc (30 mL × 3). The combined organic layers were dried over Na_2SO_4, filtered and concentrated under reduced pressure to give a residue. The residue was purified by flash silica gel chromatography (ISCO®; 25 g SepaFlash® Silica Flash Column, Eluent of 0~15% Ethyl acetate/Petroleum ether gradient @ 150 mL/min) (Petroleum ether/Ethyl acetate=1/1, Rf =0.50) to afford ***Compound 3*** (2.5 g, 6.54 mmol, 57.15% yield) as a white solid. MS-ESI (m/z) calcd for $C_{21}H_{26}N_4O_3$ $[M+H]^+$: 383.2. Found 383.2. A solution of **Compound 3** (2.5 g, 6.54 mmol) in DCM (40 mL) and TFA (10 mL) was stirred at 20°C for 3 hrs. LC-MS showed trace **Compound 3** was remained and 58% desired product was detected. The reaction mixture was treated with DIEA to adjust the pH to 8; the mixture was concentrated under reduced pressure to afford ***Compound 4*** (1.5 g, crude) as a white solid. MS-ESI (m/z) calcd for $C_{16}H_{18}N_4O$ $[M+H]^+$: 283.2. Found 283.1 To a solution of **Compound 4** (1.5 g, 5.31 mmol) in DCM (30 mL) was added DIEA (2.06 g, 15.94 mmol, 2.78 mL) and **Compound 4A** (1.41 g, 6.38 mmol). The mixture was stirred at 20°C for 12 hrs. LC-MS showed **Compound 4** was consumed completely and 27% desired product was detected. The reaction mixture was concentrated under reduced pressure to remove solvent. The residue was purified by flash silica gel chromatography (ISCO®; 25 g SepaFlash® Silica Flash Column, Eluent of 0~50% Ethyl acetate/Petroleum ether gradient @ 100 mL/min) (Petroleum ether /Ethyl acetate=1/1, Rf=0.47) to afford ***Compound 5*** (850 mg, 1.82 mmol, 34.22% yield) as a yellow solid. MS-ESI (m/z) calcd for $C_{22}H_{21}N_5O_5S$ $[M+H]^+$: 468.1. Found 468.1. A solution of DIAD (1.04 g, 5.13 mmol, 998.15 uL) and PPh_3 (1.35 g, 5.13 mmol) in THF (20 mL) was stirred at 15°C for 30 min under N_2 atomsphere, then **Compound 5A** (331.64 mg, 2.57 mmol, 348.72

uL) was added into the mixture, and the mixture was stirred at 15°C for 5 min under N_2 atomsphere. After that **Compound 5** (1.2 g, 2.57 mmol) was added into the mixture. The resulting mixture was stirred at 80°C for 12 hrs N_2 atmosphere. LC-MS showed 8% **Compound 5** was remained and 33% desired product was detected. The reaction mixture was concentrated under reduced pressure to remove solvent to give a residue. The residue was purified by flash silica gel chromatography (ISCO®; 4 g SepaFlash® Silica Flash Column, Eluent of 0~100% Ethyl acetate/Petroleum ether to 0-10% Methanol/ Ethyl acetate gradient @ 100 mL/min) (Dichloromethane / Methanol=10/1, Rf =0.36) to afford ***Compound 6*** (1.2 g, 2.07 mmol, 80.79% yield) as a yellow gum. MS-ESI (m/z) calcd for $C_{29}H_{34}N_6O_5S$ $[M+H]^+$: 579.2. Found 579.3. To a solution of **Compound 6** (1.15 g, 1.99 mmol) in ACN (15 mL) was added Cs_2CO_3 (1.29 g, 3.97 mmol) and PhSH (1.09 g, 9.94 mmol, 1.01 mL) at 15°C. The mixture was stirred at 50°C for 12 hrs. LC-MS showed **Compound 6** was consumed completely and 22% desired product was detected. The mixture was diluted with 1N HCl to adjust the ph to 2 and extracted with EtOAc (30 mL × 3), the organic phase was discarded, the aqueous phase was basified with aq. $NaHCO_3$ to PH=8 and extracted with EtOAc (50 mL × 3). The combined organic phase was dried with anhydrous Na_2SO_4, the mixture was filtered and the filtrate was concentrated under vacuum to afford ***Compound 7*** (560 mg, crude) as a yellow oil. MS-ESI (m/z) calcd for $C_{23}H_{31}N_5O$ $[M+H]^+$: 394.3. Found 394.2. To a solution of **Compound 7** (300 mg, 762.34 umol) in DMF (3 mL) was added TEA (154.28 mg, 1.52 mmol, 212.22 uL) and **Compound 7A** (111.74 mg, 762.34 umol) at 15°C. The mixture was stirred at 80°C for 12hrs. LC-MS showed **Compound 7** was consumed completely and 28% desired product was detected. The reaction mixture was concentrated under reduced pressure to remove solvent to afford ***Compound 8*** (330 mg, crude) as a yellow oil. MS-ESI (m/z) calcd for $C_{24}H_{33}N_7O$ $[M+H]^+$: 436.3. Found 436.3. A solution of **Compound 8** (330 mg, 757.64 umol) in DCM (2 mL) and TFA (2 mL) was stirred at 40°C for 2hrs. LC-MS showed 3% **Compound 8** was remained and 14% desired product was detected. The reaction mixture was concentrated under reduced pressure to remove solvent to give a residue. The residue was purified by prep-HPLC (column: Phenomenex Luna C18 150*30mm*5um; mobile phase: [water (0.1%TFA)-ACN]; B%: 1%-30%, 8min) to afford ***Compound 9*** (130 mg, 302.72 umol, 39.96% yield, TFA salt) as a pale yellow oil. MS-ESI (m/z) calcd for $C_{16}H_{25}N_7$ $[M+H]^+$: 316.2. Found 316.3. To a solution of **Compound 9** (20 mg, 46.57 umol, TFA salt) in EtOH (1 mL) was added NaOEt (6.34 mg, 93.14 umol) at 15°C, the mixture was stirred at 80°C for 0.5 hr, and **Compound 9A** (15.10 mg, 46.57 umol) was added into the mixture. The mixture was stirred at 80°C for 1 hr. NaOEt (6.34 mg, 93.14 umol) was added into the mixture, and the mixture was stirred at 80°C for another 1 hr. LC-MS showed **Compound 9** was consumed completely and 48% desired product was detected. The reaction mixture was concentrated under reduced pressure to remove solvent. The residue was purified by prep-HPLC (column: Waters Xbridge Prep OBD C18 150*40mm*10um; mobile phase: [water (10mM NH_4HCO_3)-ACN]; B%: 25%-55%, 8min). The residue was purified twice by Prep-HPLC (column: Phenomenex Gemini-NX C18 75*30mm*3um; mobile phase: [water (0.05%NH_3H_2O+10mM NH_4HCO_3)-ACN]; B%: 30%-60%, 8min) to afford ***analog 8*** (8 mg, 13.29 umol, 95.77% purity) as a gray solid. **^{1}H NMR** (DMSO-d_6 400MHz) δ ppm 11.81 (br s, 1 H) 11.36 (br s, 1 H) 8.65 (br s, 1 H) 8.27 - 8.42 (m, 1 H) 7.54 - 7.81 (m, 2 H) 7.39 - 7.48 (m, 1 H) 7.30 (br s, 1 H) 6.39 - 6.77 (m, 2 H) 3.45 - 3.86 (m, 6 H) 2.91 (br s, 1 H) 2.07 - 2.25 (m, 5 H) 1.83 - 2.06 (m, 5 H) 1.39 - 1.70 (m, 4 H). **LCMS** (ESI+): *m/z* 576.2 (M+H)

Analog 9

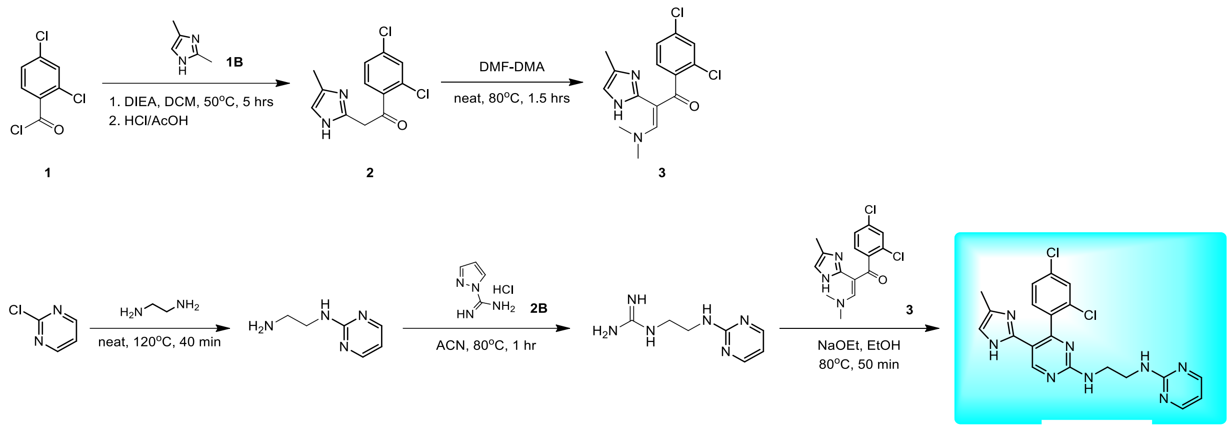

6-((2-((5-(4-methyl-1*H*-imidazol-2-yl)-4-phenylpyrimidin-2-yl)amino)ethyl)amino)- nicotinonitrile (Analog 9). A mixture of **Intermediate 1** (1 g, 7.22 mmol) and **Intermediate 1A** (13.01 g, 216.52 mmol, 14.49 mL) was stirred at 20°C for 2 hrs, then K_2CO_3 (4 g, 28.94 mmol) was added, and then the mixture was stirred at 20°C for 1 hr. LCMS showed **Intermediate 1** was consumed and 18% of desired product was detected. The reaction was filtered and the filter-cake was washed with MeOH (10 mL), the filtrate was concentrated under vacuum to afford ***Intermediate 2*** (1.79 g, crude) as a light pink solid. MS-ESI (m/z) calcd for $C_8H_{10}N_4$ $[M+H]^+$: 163.1. Found 163.1 To a solution of **Intermediate 2A** (200 mg, 1.36 mmol) in ACN (10 mL) was added **Intermediate 2** (331.96 mg, 2.05 mmol) at 20°C, then the mixture was stirred at 80°C for 12 hrs. LCMS showed **Intermediate 2** was consumed and 84% of desired product was detected. The reaction was concentrated under vacuum to afford ***Intermediate 3*** (300 mg, crude) as a yellow solid and it was used directly. MS-ESI (m/z) calcd for $C_9H_{12}N_6$ $[M+H]^+$: 205.1. Found 205.4. To a solution of **Intermediate 4A** (1 g, 10.40 mmol) in DCM (16 mL) was heated to 40°C until the solution was clear, then DIEA (4.30 g, 33.29 mmol, 5.80 mL) was added in one portion at 30°C, another solution of **Intermediate 4** (4.39 g, 31.21 mmol, 3.63 mL) in DCM (3 mL) was added dropwise under water bath (keep the temperature below 35°C), the reaction was stirred at 50°C for 5 hrs. Then the reaction was concentrated under vacuum until the solid was dry. The dry solid was added a solution of AcOH (13.8 mL) and HCl (6.9 mL) (12 M), then the mixture was stirred at 125°C for 3 hrs. LCMS showed **Intermediate 4A** was consumed and there was 47% of desired product was detected. The reaction was concentrated under vacuum. Then water (34.5 mL) and toluene (17.2 mL) were added to the liquid, then the mixture was vigorously stirred for 30 min at 20°C. Solid was appeared and was filtered, rinsed with 8.5 mL of water and the solid was discared. The filtrate was transfered into a separatory funnel, the organic layer was discarded and the aqueous layer was washed with toluene (17 mL × 2), The aqueous layer was transfered into a large breaker and diluted with MTBE (8.5 mL), then the stirred mixture was basified with saturated $NaHCO_3$ solution to pH=8. DCM (34.5 mL) was added and stirred for 10 min at 20°C, and the aqueous layer was extracted with DCM (17 mL × 1), the organic layers were combined and washed with saturated $NaHCO_3$ solution (17 mL), water (17 mL) and brine (17 mL), dried over Na_2SO_4, filtered and the filtrate was concentrated under vacuum to afford ***Intermediate 5*** (1.32 g, 6.13 mmol, 58.94% yield, 93% purity) as a yellow solid. MS-ESI (m/z) calcd for $C_{12}H_{12}N_2O$ $[M+H]^+$: 201.1. Found 201.0. A solution of **Intermediate 5** (0.3 g, 1.50 mmol) in DMF-DMA (5 mL) was stirred at 80°C for 2 hrs. LCMS showed **Intermediate 5** was consumed and 46% of desired product was detected. The reaction was concentrated under vacuum to afford ***Intermediate 6*** (400 mg, crude) as a brown liquid and it was used directly. MS-ESI (m/z) calcd for $C_{15}H_{17}N_3O$ $[M+H]^+$: 256.1. Found 256.0. A solution of **Intermediate 3** (122.39 mg, 599.26 umol) and EtONa (39.98 mg,

587.51 umol) in EtOH (2 mL) was stirred at 20°C for 5 min, then another solution of **Intermediate 6** (150 mg, 587.51 umol) in EtOH (2 mL) was added at 20°C, then the mixture was stirred at 80°C for 2.5 hrs. LCMS showed 20% of **Intermediate 6** was remained and 18% of desired product was detected. The reaction was filtered. The filtrate was purified by Prep-HPLC (basic condition) (column: Phenomenex Gemini-NX C18 75*30mm*3um; mobile phase: [water(0.05% NH_3H_2O+10mM NH_4HCO_3)-ACN]; B%: 15%-45%, 8min), the HNMR was not clean, so the residue was further purified by Prep-HPLC (HCl condition) (column: Welch Xtimate C18 150*25mm*5um; mobile phase: [water(0.04%HCl)-ACN]; B%: 3%-20%, 8 min) to afford **Analog 9** (9.96 mg, 22.60 umol, 25.60% yield, 98.24% purity, HCl salt) as a white solid. **^{1}H NMR** (METHANOL-d_4 400MHz) δ ppm 8.65 (br s, 1 H) 7.99 - 8.59 (m, 2 H) 7.57 (br s, 1 H) 7.43 (br t, *J*=7.61 Hz, 2 H) 7.28 (s, 3 H) 6.83 (br s, 1 H) 3.87 - 4.07 (m, 2 H) 3.80 (br s, 2 H) 2.32 (s, 3 H). (DMSO-d_6 400MHz) δ = ppm 14.45 (br s, 1 H) 14.06 (br s, 1 H) 8.52 - 8.64 (m, 1 H) 8.42 (br d, *J*=7.06 Hz, 1 H) 8.11 - 8.20 (m, 1 H) 7.61 - 7.78 (m, 1 H) 7.44 - 7.52 (m, 1 H) 7.35 - 7.44 (m, 3 H) 7.29 (br t, *J*=6.50 Hz, 2 H) 6.59 - 6.71 (m, 1 H) 3.53 - 3.68 (m, 4 H) 2.25 (s, 3 H). **LCMS** (ESI+): *m/z* 397.2 (M+H)

Analog 10

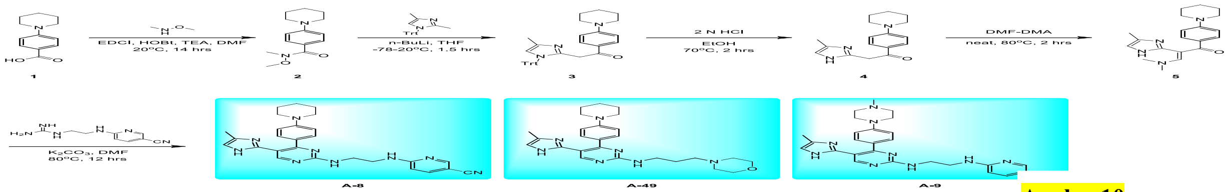

Analog 10

6-((2-((5-(4-methyl-1*H*-imidazol-2-yl)pyrimidin-2-yl)amino)ethyl)amino)nicotinonitrile (Analog 10). A mixture of **Compound 1** (200 mg, 1.26 mmol), **Compound 1A** (245.82 mg, 1.52 mmol), K_2CO_3 (523.67 mg, 3.79 mmol) in dioxane (4 mL) was stirred at 100°C for 12 hrs. LC-MS showed **Compound 1** was consumed completely and one main peak with desired compound was detected. ***Compound 2*** (360 mg, crude) in 4 mL dioxane as a yellow liquid (suspension) was used to the next step directly. MS-ESI (m/z) calcd for $C_{12}H_{13}N_6BO_2$ $[M+H]^+$: 285.1. Found 285.2. To a solution of **Compound 2** (360 mg, 1.27 mmol) in dioxane (2 mL) and H_2O (0.5 mL) was added **Compound 2A** (204.03 mg, 1.27 mmol) and K_2CO_3 (350.28 mg, 2.53 mmol) and Pd(dppf)Cl_2 (92.72 mg, 126.72 umol). The mixture was stirred at 100°C for 12 hrs under N_2. LC-MS showed **Compound 2** was consumed completely and one main peak with desired mass was detected. The reaction mixture was concentrated under reduced pressure to remove solvent. The residue was purified by prep-HPLC (HCl condition) (Column: Welch Xtimate C18 150*25mm*5um; mobile phase: [water (0.04%HCl)-ACN]; B%: 8%-20%, 8min). But HNMR showed that the residue was not pure enough to be delivered, so the residue was purified further using prep-HPLC (neutral condition) (column: Waters Xbridge Prep OBD C18 150*40mm*10um; mobile phase: [water (10mM NH_4HCO_3)-ACN];B%: 20%-50%,8min) to afford ***Analog 10*** (15.8 mg, 49.32 umol, 3.89% yield, 100% purity) as a white solid. **^{1}H NMR** (METHANOL-d_4 400MHz) δ ppm 8.69 (s, 2 H) 8.31 (d, *J*=1.98 Hz, 1 H) 7.58 (br d, *J*=9.26 Hz, 1 H) 6.79 (br s, 1 H) 6.56 (br d, *J*=8.60 Hz, 1 H) 3.63 (s, 4 H) 2.25 (s, 3 H). **LCMS** (ESI+): *m/z* 321.2 (M+H)

Analog 11, 13, 41

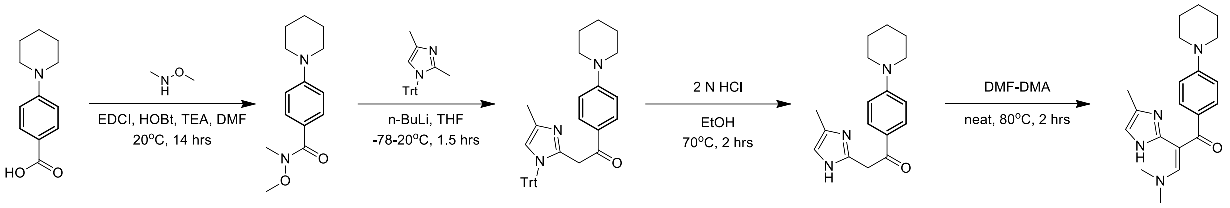

1 2 3 4 5

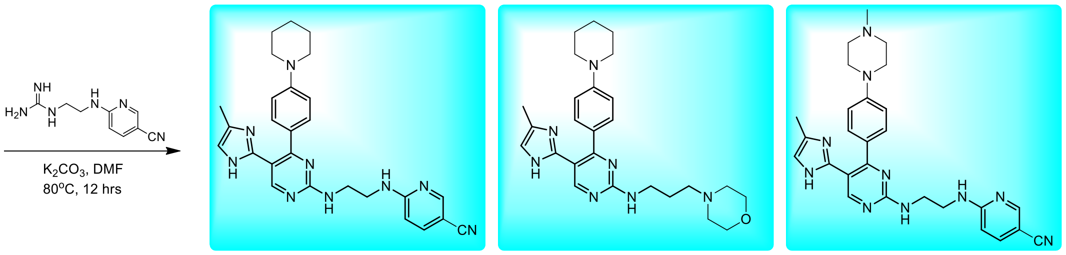

Analog 11 Analog 41 Analog 13

6-((2-((5-(4-methyl-1*H*-imidazol-2-yl)-4-(4-(piperidin-1-yl)phenyl)pyrimidin-2-yl)amino)ethyl)amino)nicotinonitrile (Analog 11); 6-((2-((5-(4-methyl-1*H*-imidazol-2-yl)-4-(4-(4-methylpiperazin-1-yl)phenyl)pyrimidin-2-yl)amino)ethyl)amino)nicotinonitrile (Analog 13); To a solution of **Compound 1** (1 g, 4.87 mmol) and **Compound 1A** (570.29 mg, 5.85 mmol, 1.2 eq, HCl salt) in DMF (5 mL) was added EDCI (1.40 g, 7.31 mmol), HOBt (987.49 mg, 7.31 mmol) and TEA (1.48 g, 14.62 mmol, 2.03 mL). The mixture was stirred at 20°C for 14 hrs. TLC indicated that **Compound 1** was consumed, and one main spot was formed (SiO_2, Petroleum ether/Ethyl acetate=1/1, P1Rf=0.59). The reaction mixture was concentrated under reduced pressure to remove solvent. The residue was purified by flash silica gel chromatography (ISCO®; 4 g SepaFlash® Silica Flash Column, Eluent of 0~30% Ethyl acetate/Petroleum ether gradient @ 50 mL/min) to afford ***Compound 2*** (1 g, 4.03 mmol, 82.66% yield) as a yellow oil. To a solution of **Compound 2** (1 g, 4.03 mmol) in THF (5 mL) was added n-BuLi (2.5 M, 4.83 mL) at -78°C, the mixture was stirred at -78°C for 0.5 hr, then Compound 2A (1.36 g, 4.03 mmol) was added, and the mixture was allowed to warm up to 20°C, and stirred at 20°C for 1 hr. LC-MS showed **Compound 2** was consumed, and 48% of desired product was detected. The reaction mixture was quenched with 100 mL sat. NH_4Cl, the mixture was extracted with ethyl acetate (20 mL × 3). The combined organic layers were washed with brine (20 mL × 1), dried over Na_2SO_4, filtered and concentrated under reduced pressure to afford ***Compound 3*** (2 g, crude) as a yellow solid. MS-ESI (m/z) calcd for $C_{36}H_{35}N_3O$ $[M+H]^+$: 526.3. Found 526.2. To a solution of **Compound 3** (2 g, 3.80 mmol) in EtOH (2 mL) was added HCl (2 M, 21.05 mL) and the mixture was stirred at 70°C for 2 hrs. LC-MS showed **Compound 3** was consumed completely and 37% desired product was detected. The reaction mixture was concentrated under reduced pressure to remove solvent. The residue was diluted with H_2O 60 mL and extracted with ethyl acetate (10 mL × 3). The combined organic layers were discarded and the water phase was adjusted the pH to about 9 with sat. $NaHCO_3$, extracted with ethyl acetate (10 mL × 3). The combined organic layers were dried over Na_2SO_4, filtered and concentrated under reduced pressure to afford ***Compound 4*** (1 g, crude) (85% purity) as a brown solid. MS-ESI (m/z) calcd for

$C_{17}H_{21}N_3O$ [M+H]$^+$: 284.2. Found 284.2. A mixture of **Compound 4** (150 mg, 529.35 umol) in DMFDMA (2 mL) was stirred at 80°C for 2 hrs under N_2 atmosphere. LC-MS showed **Compound 4** was consumed completely and one main peak with desired mass was detected. The reaction mixture was concentrated under reduced pressure to remove solvent to afford ***Compound 5*** (180 mg, crude) as a brown oil. MS-ESI (m/z) calcd for $C_{20}H_{26}N_4O$ [M+H]$^+$: 339.2. Found 339.2. To a solution of **Compound 5A** (108.62 mg, 531.84 umol) and **Compound 5**(180 mg, 531.84 umol) in DMF (2 mL) was added K_2CO_3 (220.51 mg, 1.60 mmol) and the mixture was stirred at 80°C for 12 hrs. LC-MS showed **Compound 5** was consumed and 6% desired product was detected. The reaction mixture was concentrated under reduced pressure to remove solvent. The residue was purified by prep-HPLC (basic condition) (column: Phenomenex Gemini-NX C18 75*30mm*3um; mobile phase: [water (0.05%NH_3H_2O+10mM NH_4HCO_3)-ACN]; B%: 15%-45%, 8min). But HNMR showed that there was some impurity in the product, so the residue was purified by prep-HPLC (TFA condition) (column: Phenomenex luna C18 80*40mm*3 um; mobile phase: [water (0.1%TFA)-ACN]; B%: 1%-30%, 12min). But HNMR still looked a little messy, so the residue was basified with 10 ml sat. $NaHCO_3$ and extracted with EtOAc (3ml × 3), then the organic phase was concentrated and the residue was purified third time by prep-HPLC (basic condition) (column: Phenomenex Gemini-NX C18 75*30mm*3um;mobile phase: [water(0.05%NH_3H_2O+10mM NH_4HCO_3)-ACN];B%: 15%-70%,8min) to afford **analog 11** (17.30 mg, 36.02 umol, 6.77% yield, 99.85% purity) as a pale yellow solid. **The procedure to make 13 is similar with that of 11.**

Analog 11: ^{1}H NMR (METHANOL-d_4 400MHz) δ ppm. 8.20 - 8.35 (m, 2 H) 7.48 (br d, *J*=6.24 Hz, 1 H) 7.25 (d, *J*=8.93 Hz, 2 H) 6.82 (d, *J*=9.05 Hz, 2 H) 6.76 (d, *J*=0.86 Hz, 1 H) 6.48 (br s, 1 H) 3.57 - 3.76 (m, 4 H) 3.21 - 3.28 (m, 4 H) 2.20 (d, *J*=0.73 Hz, 3 H) 1.57 - 1.71 (m, 6 H)**. LCMS** (ESI+): *m/z* 480.3 (M+H).

Analog 13: ^{1}H NMR (METHANOL-d_4 400MHz) δ ppm. 8.20 - 8.37 (m, 2 H) 7.48 (br s, 1 H) 7.27 (d, *J*=8.93 Hz, 2 H) 6.86 (d, *J*=8.93 Hz, 2 H) 6.73 (d, *J*=0.86 Hz, 1 H) 6.47 (br s, 1 H) 3.72 (br s, 2 H) 3.64 (br d, *J*=5.26 Hz, 2 H) 3.25 - 3.30 (m, 4 H) 2.55 - 2.62 (m, 4 H) 2.34 (s, 3 H) 2.19 (s, 3 H). **LCMS** (ESI+): *m/z* 495.3 (M+H)

Analog 12

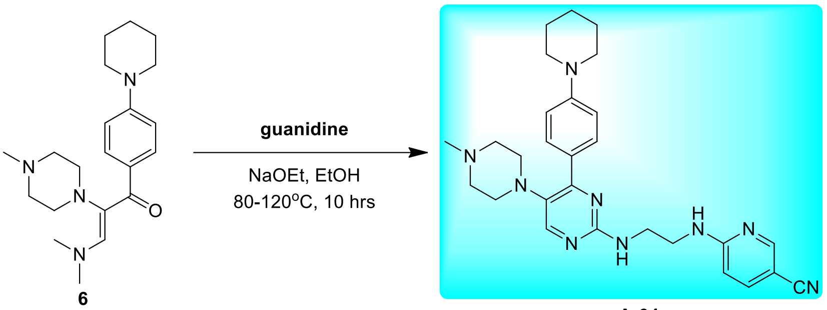

A-64

Analog 12

6-((2-((5-(4-methylpiperazin-1-yl)-4-(4-(piperidin-1-yl)phenyl)pyrimidin-2-yl)amino)ethyl)amino)nicotinonitrile (Analog 12). To a solution of **guanidine** (114.57 mg, 561.00 umol) in EtOH (3 mL) was added EtONa (76.35 mg, 1.12 mmol) at 20°C, then the mixture was stirred at 80°C for 15 min, then another solution of **Compound 6** (200 mg, 561.00 umol) in EtOH (2 mL) was added at 80°C, then the mixture was stirred at 120°C for 5 hrs under microwave. LCMS showed no reaction. Then the mixture was stirred at 120°C for 5 hrs under microwave. LCMS showed **Compound 6** was consumed and 24% of desired product was detected. The reaction was concentrated under vacuum. The residue was purified by Prep-HPLC (basic condition) (column: Waters Xbridge Prep OBD C18 150*40mm*10um; mobile phase: [water (0.05% NH_3H_2O+10 mM NH_4HCO_3)-ACN]; B%: 30%-60%, 8 min) to afford ***12*** (27.1 mg, 53.10 umol, 9.46% yield, 97.50% purity) as a pale yellow solid. **^{1}H NMR** (METHANOL-d_4 400MHz) δ ppm 8.28 (d, J=1.67 Hz, 1 H) 8.00 - 8.09 (m, 3 H) 7.48 (dd, J=8.88, 1.97 Hz, 1 H) 6.98 (d, J=8.94 Hz, 2 H) 6.46 (br s, 1 H) 3.54 - 3.67 (m, 4 H) 3.27 - 3.30 (m, 4 H) 2.86 (br s, 4 H) 2.52 (br s, 4 H) 2.31 (s, 3 H) 1.62 - 1.76 (m, 6 H). **LCMS** (ESI+): *m/z* 498.3 (M+H)

Analog 14

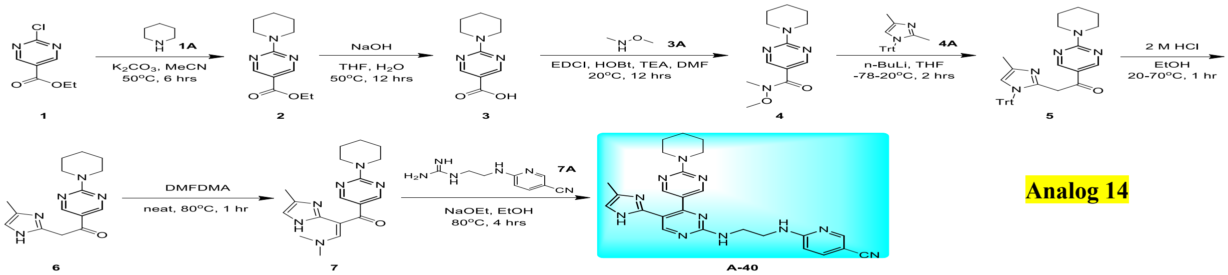

Analog 14

6-((2-((4-(4-(piperidin-1-yl)phenyl)-5-(pyrazin-2-yl)pyrimidin-2-yl)amino)ethyl)amino)-nicotinonitrile (Analog 14). To a solution of 2-methylpyrazine (75.80 mg, 805.41 umol, 73.59 uL) in THF (1 mL) was added LDA (2 M, 483.25 uL, 1.2 eq) at 0°C under N_2 atmosphere. After 10 min, to the reaction mixture was added a solution of **Compound 1** (200 mg, 805.41 umol) in THF (2 mL). Then the resulting mixture was stirred at 20°C for 17 hrs under N_2 atmosphere. LC-MS showed **Compound 1** was remained and 37% desired product was detected. The reaction mixture was diluted with sat. NH_4Cl

10 mL and was extracted with ethyl acetate (5 mL × 3). The combined organic layers were dried over Na_2SO_4, filtered and concentrated under reduced pressure to give a residue. The residue was purified by flash silica gel chromatography (ISCO®; 4 g SepaFlash® Silica Flash Column, Eluent of 0~25% Ethyl acetate/Petroleum ether gradient @ 50 mL/min) (Petroleum ether/Ethyl acetate=1/1, P1Rf=0.3) to afford **Compound 2** (100 mg, 355.43 umol, 44.13% yield) as a yellow solid. MS-ESI (m/z) calcd for $C_{17}H_{19}N_3O$ $[M+H]^+$: 282.2. Found 282.0. A mixture of **Compound 2** (50 mg, 177.71 umol) in DMFDMA (1 mL) was stirred at 80 °C for 1 hr under N_2 atmosphere. LC-MS showed **Compound 2** was consumed completely and one main peak with desired mass was detected. The reaction mixture was concentrated under reduced pressure to afford **Compound 3** (60 mg, crude) as a brown gum. MS-ESI (m/z) calcd for $C_{20}H_{24}N_4O$ $[M+H]^+$: 337.2. Found 377.3. To a solution of **Compound 3A** (36.42 mg, 178.34 umol) in EtOH(1 mL) was added NaOEt (24.27 mg, 356.69 umol) and the mixture was stirred at 80°C for 0.5 hr, then **Compound 3** (60 mg, 178.34 umol, 1 eq) in EtOH(1 mL) was added, and the resulting mixture was stirred at 80°C for 2 hrs. LC-MS showed **Compound 3** was consumed completely and one main peak with desired mass was detected. The reaction mixture was concentrated under reduced pressure to remove solvent. The residue was purified by prep-HPLC (basic condition) (column: Phenomenex Gemini-NX C18 75*30mm*3um; mobile phase: [water (0.05%NH_3H_2O+10mM NH_4HCO_3)-ACN]; B%: 35%-60%, 8min) to afford ***14*** (30.7 mg, 62.81 umol, 35.22% yield, 97.7% purity) as a yellow solid. **^{1}H NMR** (METHANOL-*d*$_4$ 400MHz) δ ppm 8.59 - 8.65 (m, 1 H) 8.47 (br s, 1 H) 8.39 (d, J=2.43 Hz, 1 H) 8.30 (s, 1 H) 8.17 (s, 1 H) 7.44 - 7.57 (m, 1 H) 7.17 (d, J=8.82 Hz, 2 H) 6.85 (d, J=8.82 Hz, 2 H) 6.50 (br s, 1 H) 3.74 (br s, 2 H) 3.65 (br s, 2 H) 3.24 (br d, J=5.07 Hz, 4 H) 1.66 (br d, J=4.85 Hz, 6 H). **LCMS** (ESI+): *m/z* 478.2 (M+H)

Analog 15

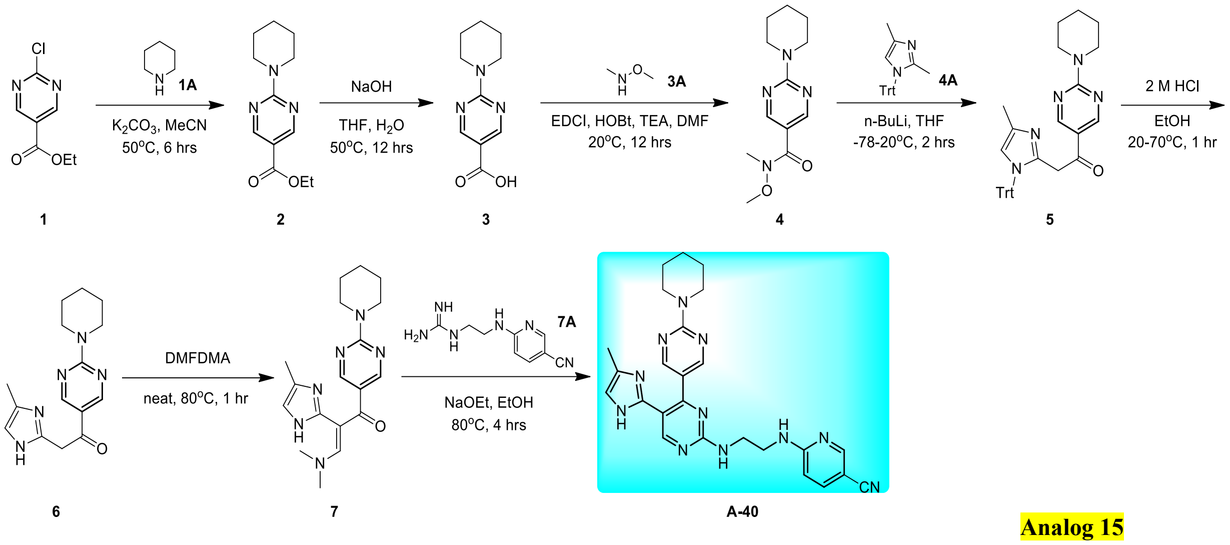

Analog 15

6-((3-(5-(4-methyl-1*H*-imidazol-2-yl)-4-(4-(piperidin-1-yl)phenyl)pyrimidin-2-yl)propyl)amino)nicotinonitrile (Analog 15). To a solution of **Compound 1** (5 g, 33.78 mmol) in DMF (100 mL) was added **Compound 1A** (6.26 g, 33.78 mmol). The mixture was stirred at 100°C for 12 hrs. LCMS showed **Compound 1** was consumed completely and one main peak with desired mass was detected. The reaction mixture was cooled to 20°C and diluted with H_2O (500 ml) and extracted with ethyl acetate (200 ml × 5), the combined organic layer was washed with saturated brine (200 ml × 2), dried over Na_2SO_4, filtered and the filtrate was concentrated to give a residue. The residue was purified by flash silica gel chromatography (ISCO®; 40 g SepaFlash® Silica Flash Column, Eluent of 0~30% Ethyl acetate/Petroleum ether gradient @ 100 mL/min) (petroleum ether: ethyl acetate=2:1, Rf = 0.43) to afford ***Compound 2*** (4.3 g, 20.07 mmol, 59.42% yield) was obtained as a white solid. MS-ESI (m/z) calcd for $C_{12}H_{10}N_2O_2$ $[M+H]^+$: 215.1. Found 215.2. HCl (gas) was bubbled into a solution of **Compound 2** (0.5 g, 2.33 mmol) in EtOH (12 mL) and $CHCl_3$ (12 mL) at 0°C for 30 minutes. Then the reaction was stirred at 20°C for 12 hrs. LCMS showed **Compound 2** was consumed and 71% of desired product was detected. The reaction was concentrated under vacuum to afford ***Compound 3*** (685 mg, crude) was obtained as a white solid and it was used directly. MS-ESI (m/z) calcd for $C_{14}H_{16}N_2O_3$ $[M+H]^+$: 261.1. Found 261.1. To a solution of **Compound 3** (685 mg, 2.63 mmol) in MeOH (10 mL) were added NaOMe (142.17 mg, 2.63 mmol) and NH_4Cl (140.77 mg, 2.63 mmol) at 20°C, then the mixture was stirred at 20°C for 4 hrs and the reaction was stirred for 12 hrs. LCMS showed **Compound 3** was consumed and 84% of desired product was detected. The reaction was concentrated under vacuum, the filtrate was purified by Prep-HPLC (basic condition) (column: Waters Xbridge Prep OBD C18 150*40mm*10um; mobile phase: [water (0.05%NH_3H_2O+10mM NH_4HCO_3)-ACN]; B%: 1%-30%, 8 min) to afford ***Compound 4*** (360 mg, 1.56 mmol, 52.71% yield) as a white solid. MS-ESI (m/z) calcd for $C_{12}H_{13}N_3O_2$ $[M+H]^+$: 232.1. Found 231.9. 1H NMR (400 MHz, METHANOL-d4) δ ppm 7.69 (d, J=7.09 Hz, 1 H) 7.37 - 7.46 (m, 3 H) 3.38 - 3.44 (m, 2 H) 2.62 (t, J=7.76 Hz, 2 H) 1.92 - 2.02 (m, 2 H). To a solution of **Compound 4** (146.90 mg, 635.26 umol) and EtONa (86.46 mg, 1.27 mmol) in EtOH (2 mL) was stirred at 80°C for 15 min, then another solution of **Compound 4A** (215 mg, 635.26 umol) in EtOH (2 mL) was stirred at 80°C for 12 hrs. LCMS showed **Compound 4A** was consumed and 22% of desired product was detected. The reaction was concentrated under vacuum. The residue was purified by Prep-TLC (Petroleum ether: Ethyl acetate=0:1) (P1 Rf=0.16) to afford ***Compound 5*** (53 mg, 104.62 umol, 16.47% yield) as a yellow liquid. MS-ESI (m/z) calcd for $C_{30}H_{30}N_6O_2$ $[M+H]^+$: 507.2. Found 507.2 To a solution of **Compound 5** (53 mg, 104.21 umol) in EtOH (2 mL) was added $NH_2NH_2.H_2O$ (122.74 mg,

2.08 mmol, 119.17 uL, 85% purity) at 20°C, then the mixture was stirred at 80°C for 1 hr. LCMS showed **Compound 5** was consumed and 87% of desired product was detected. The reaction was cooled to room temperature, then solid was appeared and the reaction was filtered and the filtrate was concentrated under vacuum to afford ***Compound 6*** (50 mg, crude) as a yellow liquid and it was used directly. MS-ESI (m/z) calcd for $C_{22}H_{28}N_6$ $[M+H]^+$: 377.2. Found 377.2 To a solution of **Compound 6** (45 mg, 119.52 umol) and **Compound 6A** (21.89 mg, 179.28 umol) in DMF (1.5 mL) was added DIEA (46.34 mg, 358.57 umol, 62.46 uL), then the mixture was stirred at 80°C for 2 hrs. LCMS showed **Compound 6** was consumed and 13.9% of desired product was detected. The reaction was filtered. The filtrate was purified by Prep-HPLC (basic condition) (column: Phenomenex Gemini-NX C18 75*30mm*3um; mobile phase: [water (0.05%NH_3H_2O+10mM NH_4HCO_3)-ACN]; B%: 40%-65%, 8 min) to afford ***15*** (11.00 mg, 22.65 umol, 18.95% yield, 98.53% purity) as a pale yellow gum. **^{1}H NMR** (METHANOL-d_4 400MHz) δ ppm 8.65 (s, 1 H) 8.25 (d, *J*=1.79 Hz, 1 H) 7.51 (dd, *J*=8.88, 2.21 Hz, 1 H) 7.34 (d, *J*=8.94 Hz, 2 H) 6.80 - 6.91 (m, 3 H) 6.50 (d, *J*=8.94 Hz, 1 H) 3.49 (br t, *J*=6.74 Hz, 2 H) 3.26 - 3.31 (m, 4 H) 3.07 (t, *J*=7.33 Hz, 2 H) 2.15 - 2.30 (m, 5 H) 1.66 (br d, *J*=7.63 Hz, 6 H). **LCMS** (ESI+): *m/z* 479.3 (M+H)

Analog 58, 16, 84

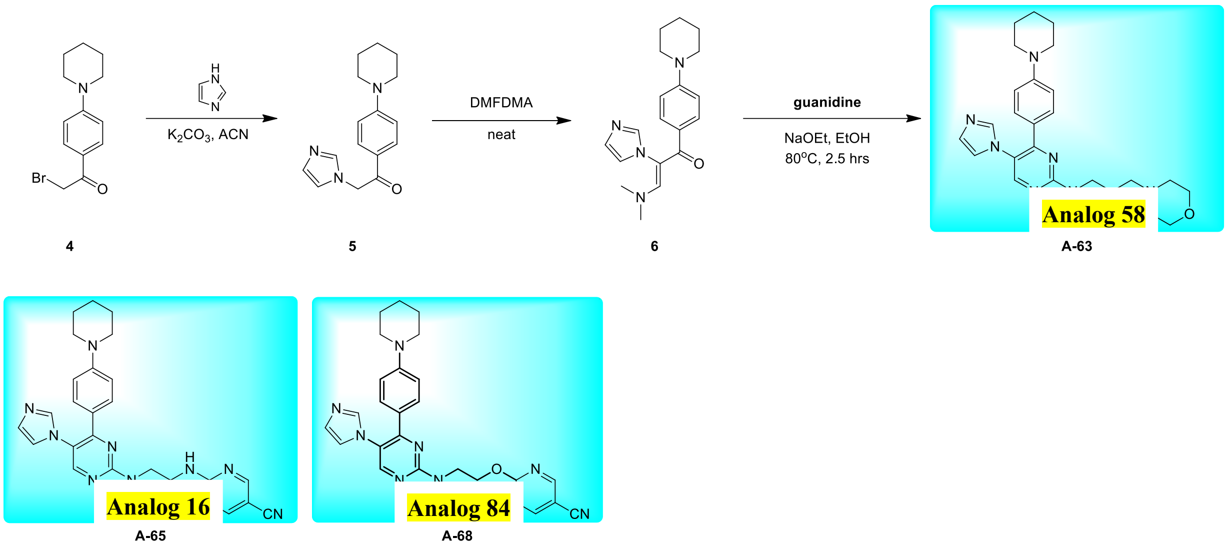

5-(1*H*-imidazol-1-yl)-*N*-(3-morpholinopropyl)-4-(4-(piperidin-1-yl)phenyl)pyrimidin-2-amine (Analog 58). To a solution of **Compound 4** (2 g, 7.09 mmol) and imidazole (579.02 mg, 8.51 mmol) in ACN (20 mL) was added K_2CO_3 (2.45 g, 17.72 mmol) at 20°C, then the mixture was stirred at 80°C for 1 hr. LCMS showed **Compound 4** was consumed and 56% of desired product was detected. The reaction was concentrated under vacuum. The residue was diluted with water (10 mL) and EtOAc (10 mL), solid was appeared and the mixture was filtered and the filter-cake was dried under vacuum to give ***Compound 5*** (400 mg, 1.49 mmol, 20.95% yield) as a yellow solid and it was used directly. MS-ESI (m/z) calcd for $C_{16}H_{19}N_3O$ $[M+H]^+$: 270.2. Found 270.1. A solution of **Compound 5** (200 mg, 742.55 umol) in DMFDMA (5 mL) was stirred at 80°C for 1 hr. LCMS showed 2% of **Compound 5** was remained and 24% of desired product was detected. The reaction was concentrated under vacuum to afford ***Compound 6*** (250 mg, crude) as a brown liquid and it was used directly. A solution of **guanidine** (71.76 mg, 385.30 umol) and EtONa (78.66 mg, 1.16 mmol) in EtOH (2 mL) was stirred at 80°C for 15

min. Then another solution of **Compound 6** (125 mg, 385.30 umol) in EtOH (2 mL) was added at 80°C, then the mixture was stirred at 80°C for 2 hrs. LCMS showed 2% of **Compound 6** was remained and 63% of desired product was detected. The reaction was filtered. The filtrate was purified by Prep-HPLC (neutral condition) (column: Waters Xbridge Prep OBD C18 150*40mm*10um; mobile phase: [water (10 mM NH_4HCO_3)-ACN]; B%: 30%-60%, 8 min) to afford ***58*** (24.7 mg, 53.86 umol, 13.98% yield, 97.59% purity) as a yellow solid. **^{1}H NMR** (METHANOL-*d*$_4$ 400MHz) δ ppm 8.17 (s, 1 H) 7.62 (s, 1 H) 7.17 (br d, J=8.76 Hz, 2 H) 7.09 - 7.14 (m, 2 H) 6.80 (d, J=9.13 Hz, 2 H) 3.70 (t, J=4.69 Hz, 4 H) 3.52 (br t, J=6.44 Hz, 2 H) 3.23 (br d, J=5.50 Hz, 4 H) 2.43 - 2.53 (m, 6 H) 1.87 (quin, J=7.10 Hz, 2 H) 1.60 - 1.69 (m, 6 H). **LCMS** (ESI+): *m/z* 448.3 (M+H)

General procedure for preparation of Analog 16

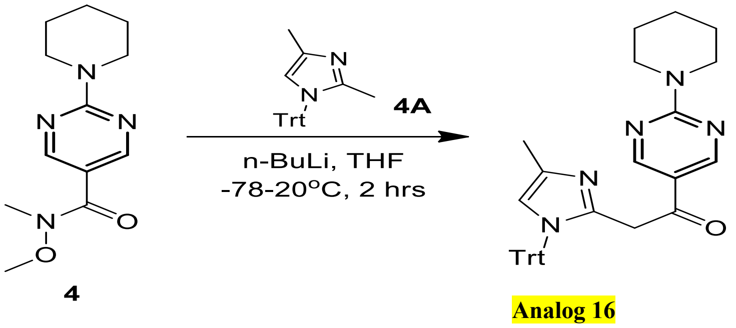

Analog 16

6-((2-((5-(1*H*-imidazol-1-yl)-4-(4-(piperidin-1-yl)phenyl)pyrimidin-2-yl)amino)ethyl)amino)-nicotinonitrile (16). A solution of **guanidine** (78.69 mg, 385.30 umol) and EtONa (78.66 mg, 1.16 mmol) in ETOH (2 mL) was stirred at 80°C for 15 min. Then another solution of **Compound 6** (125 mg, 385.30 umol) in EtOH (2 mL) was added at 80°C, then the mixture was stirred at 80°C for 2 hrs. LCMS showed 11% of **Compound 6** was remained and 60% of desired product was detected. The reaction was filtered. The filtrate was purified by prep-HPLC (neutral condition) (column: Waters Xbridge Prep OBD C18 150*40mm*10um; mobile phase: [water (10 mM NH_4HCO_3)-ACN]; B%: 40%-70%, 8 min) to afford ***16*** (34.2 mg, 73.46 umol, 19.07% yield, 100% purity) as a pale yellow solid. **^{1}H NMR** (METHANOL-*d*$_4$ 400MHz) δ ppm 8.30 (d, J=1.75 Hz, 1 H) 8.17 (br s, 1 H) 7.61 (s, 1 H) 7.50 (br s, 1 H) 7.08 - 7.15 (m, 4 H) 6.78 (d, J=9.01 Hz, 2 H) 6.51 (br s, 1 H) 3.72 (br s, 2 H) 3.64 (br d, J=5.25 Hz, 2 H) 3.25 (br d, J=5.38 Hz, 4 H) 1.59 - 1.69 (m, 6 H). **LCMS** (ESI+): *m/z* 466.3 (M+H). A solution of **guanidine** (126.51 mg, 616.49 umol) and EtONa (125.86 mg, 1.85 mmol, 3 eq) in EtOH (2 mL) was stirred at 80°C for 15 min. Then another solution of **Compound 6** (200 mg, 616.49 umol, 1 eq) in EtOH (2 mL) was added at 80°C, then the mixture was stirred at 80°C for 12 hrs. LCMS showed 34% of **Compound 6** was remained and 25% of desired product was detected. The reaction was filtered. The filtrate was purified by Prep-HPLC (basic condition) (column: Phenomenex Gemini-NX C18 75*30mm*3um; mobile phase: [water (0.05%NH_3H_2O+10mM NH_4HCO_3)-ACN]; B%: 25%-55%, 8 min) to afford ***84***(19.3 mg, 39.14 umol, 6.35% yield, 94.62% purity) as a yellow gum. **^{1}H NMR** (METHANOL-*d*$_4$ 400MHz) δ ppm 8.51 (d, *J*=1.75 Hz, 1 H) 8.17 (s, 1 H) 7.88 (br s, 1 H) 7.62 (t, *J*=1.10 Hz, 1 H) 7.05 - 7.18 (m, 4 H) 6.87 (br d, *J*=7.02 Hz, 1 H) 6.77 (d, *J*=8.99 Hz, 2 H) 4.62 (t, *J*=5.37 Hz, 2 H) 3.90 (br s, 2 H) 3.24 (br d, *J*=5.48 Hz, 4 H) 1.54 - 1.72 (m, 6 H). **LCMS** (ESI+): *m/z* 467.2 (M+H)

Analog 57, 17

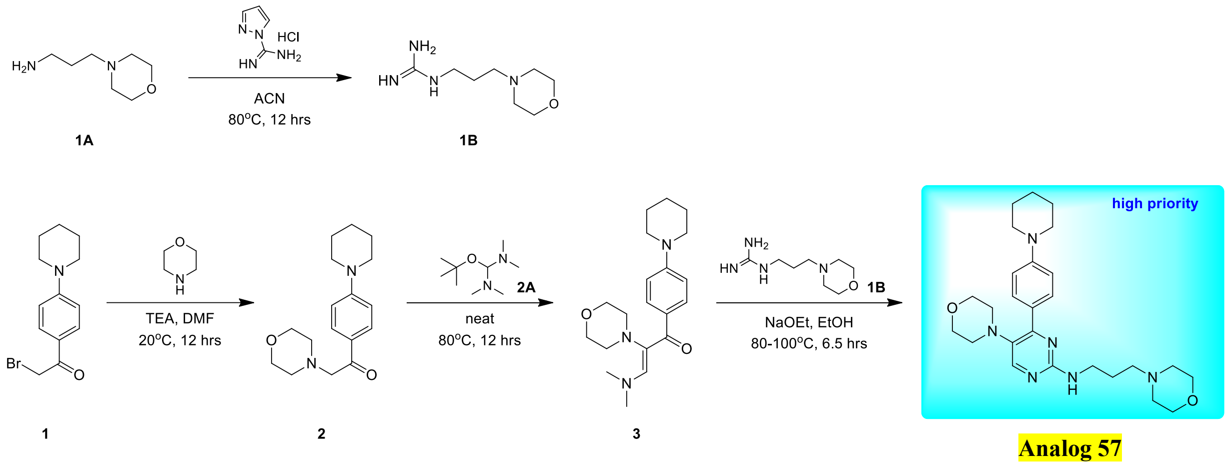

Analog 57

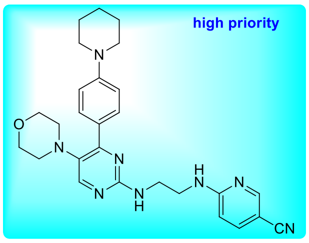

Analog 17

5-morpholino-*N*-(3-morpholinopropyl)-4-(4-(piperidin-1-yl)phenyl)pyrimidin-2-amine (Analog 57). To a solution of **Compound 1A** (100 mg, 693.41 umol, 101.32 uL) in ACN (2 mL) was added pyrazole-1-carboxamidine (101.64 mg, 693.41 umol, HCl). The mixture was stirred at 80 °C for 12 hrs. LC-MS showed **Compound 1A** was consumed completely and one main peak with desired mass was detected. The reaction mixture was concentrated under reduced pressure to afford ***Compound 1B*** (130 mg, crude) as a colorless oil. MS-ESI (m/z) calcd for $C_8H_{18}N_4O$ $[M+H]^+$: 187.1. Found 187.0. To a solution of **Compound 1**(2 g, 7.09 mmol) and TEA (2.15 g, 21.26 mmol, 2.96 mL) in DMF (10 mL) was added MORPHOLINE (1.23 g, 14.18 mmol, 1.25 mL). The mixture was stirred at 20°C for 12 hrs. LC-MS showed Reactant 1 was consumed completely and 7% desired product was detected. The reaction was worked up together with another batch. The reaction mixture was diluted with H_2O 20 mL and extracted with ethyl acetate (5 mL × 3). The combined organic layers were dried over Na_2SO_4, filtered and concentrated under reduced pressure to give a residue. The residue was purified by flash silica gel chromatography (ISCO®; 12 g SepaFlash® Silica Flash Column, Eluent of 0~20% Ethyl acetate/Petroleum ether gradient @ 75 mL/min) (Petroleum ether/Ethyl acetate=1/1, P1Rf=0.3) to afford ***Compound 2*** (1.3 g, 4.51 mmol, 63.60% yield) as a yellow solid. MS-ESI (m/z) calcd for $C_{17}H_{24}N_2O_2$ $[M+H]^+$: 289.2. Found 289.0. A mixture of **Compound 2**(200 mg, 693.52 umol) in **Compound 2A** (1.69 g, 9.69 mmol, 2.00 mL) was stirred at 80°C for 12 hrs. LC-MS showed Reactant 1 was consumed completely and one main peak with desired mass was detected. The reaction mixture was concentrated under reduced pressure to afford ***Compound 3*** (240 mg, crude) as a yellow solid. MS-ESI (m/z) calcd for $C_{20}H_{29}N_3O_2$ $[M+H]^+$: 344.2. Found 344.4. To a solution of **Compound 1B** (129.61 mg, 695.86 umol) in EtOH (1 mL) was added NaOEt (142.06 mg, 2.09 mmol) and the mixture was stirred at 80°C for 0.5 hr, then **Compound 3** (239 mg, 695.86 umol) in EtOH (2 mL) was added at 80°C, and then the resulting mixture was stirred at 100°C for 6 hrs under microwave. LC-MS showed 22% Reactant 1 was remained and 14% desired product was detected. The reaction mixture was concentrated under reduced pressure to remove solvent. The residue was purified by prep-HPLC (HCl condition) (column: Welch Xtimate C18 150*25mm*5um;mobile phase: [water(0.04%HCl)-ACN];B%: 10%-30%,8min), but LCMS showed

that the purity was not high enough, so the residue was purified further by Prep-HPLC(basic condition)(column: Waters Xbridge Prep OBD C18 150*40mm*10um;mobile phase: [water(0.05%NH_3H_2O+10mM NH_4HCO_3)-ACN];B%: 25%-55%,8min) to afford ***A-62*** (16 mg, 34.29 umol, 4.93% yield, 100% purity) as a green solid. MS-ESI (m/z) calcd for $C_{26}H_{38}N_6O_2$ $[M+H]^+$: 467.3. Found 467.2. To a solution of **Compound 3A** (89.19 mg, 436.73 umol) in EtOH(1 mL) was added NaOEt (89.16 mg, 1.31 mmol) and the mixture was stirred at 80°C for 0.5 hr, then **Compound 3** (150 mg, 436.73 umol) in EtOH(1 mL) was added at 80°C, then the mixture was stirred at 100°C under microwave for 6 hrs. LC-MS showed 13% Reactant 1 was remained and 40% desired product was detected. The reaction mixture was concentrated under reduced pressure to remove solvent. The residue was purified together with another batch by prep-HPLC (basic condition) (column: Phenomenex Gemini-NX C18 75*30mm*3um; mobile phase: [water (0.05%NH_3H_2O+10mM NH_4HCO_3)-ACN]; B%: 45%-70%, 8min) to afford ***57*** (30.9 mg, 63.42 umol, 14.52% yield, 99.46% purity) as a yellow solid. **^{1}H NMR** (METHANOL-d_4 400MHz) δ ppm 8.11 (d, J=9.05 Hz, 2 H) 8.01 (s, 1 H) 6.99 (d, J=9.05 Hz, 2 H) 3.65 - 3.74 (m, 8 H) 3.43 (t, J=6.79 Hz, 2 H) 3.28 (br d, J=5.62 Hz, 4 H) 2.75 - 2.86 (m, 4 H) 2.48 (br t, J=7.40 Hz, 6 H) 1.83 (quin, J=7.15 Hz, 2 H) 1.60 - 1.75 (m, 6 H). **LCMS** (ESI+): *m/z* 467.2 (M+H)

A similar route was used to synthesize **6-((2-((5-morpholino-4-(4-(piperidin-1-yl)phenyl)pyrimidin-2-yl)amino)ethyl)amino)-nicotinonitrile (Analog 17)**. **^{1}H NMR** (METHANOL-d_4 400MHz) δ ppm 8.27 (d, J=1.96 Hz, 1 H) 8.07 (d, J=9.05 Hz, 2 H) 8.01 (s, 1 H) 7.48 (dd, J=8.86, 2.26 Hz, 1 H) 6.98 (d, J=9.05 Hz, 2 H) 6.46 (br d, J=8.56 Hz, 1 H) 3.68 - 3.74 (m, 4 H) 3.61 (br dd, J=12.41, 4.71 Hz, 4 H) 3.27 - 3.30 (m, 4 H) 2.77 - 2.86 (m, 4 H) 1.61 - 1.76 (m, 6 H). **LCMS** (ESI+): *m/z* 485.3 (M+H)

Analog 18

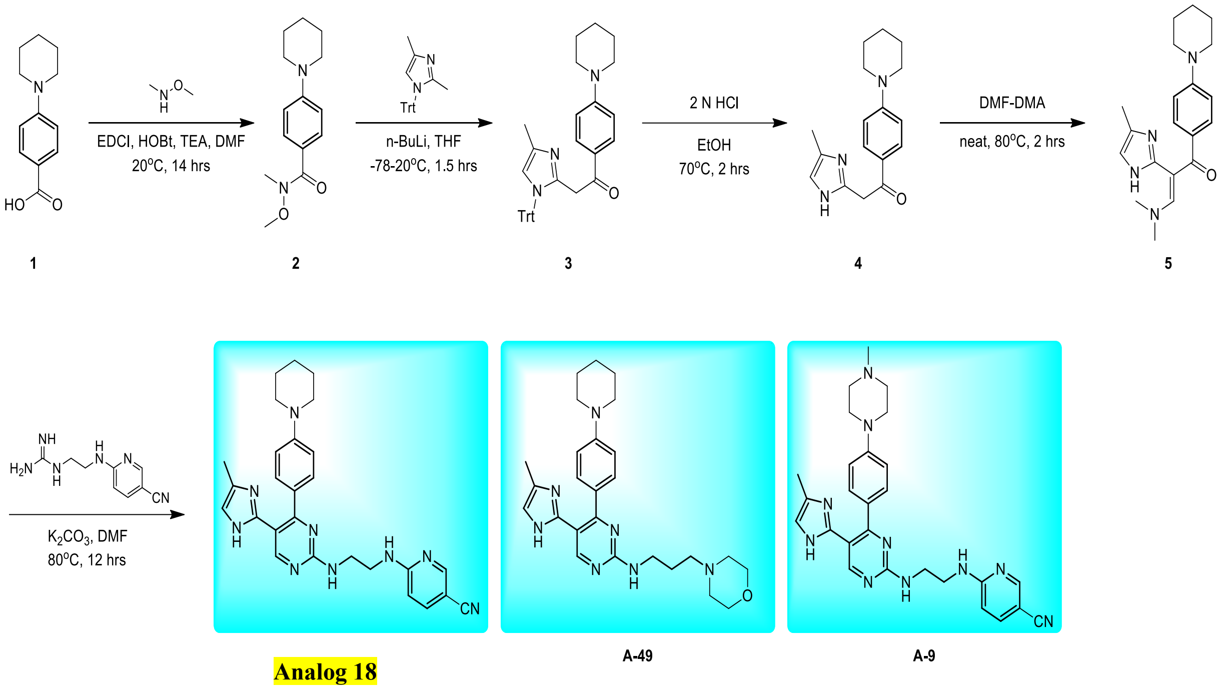

4-(2,4-dichlorophenyl)-5-(4-methyl-1*H*-imidazol-2-yl)-*N*-(2-(piperidin-4-yloxy)ethyl)pyrimidin-2-amine (analog 18). To a solution of **Compound 1** (4.39 g, 46.16 mmol) in THF (330 mL) were added PPh_3 (13.32 g, 50.78 mmol) and DIAD (10.27 g, 50.78 mmol, 9.87 mL) at 20 °C, then the mixture was stirred at 20°C for 0.5 hr, then **Compound 1A** (7.44 g, 46.16 mmol, 7.16 mL) was added and the mixture was stirred at 70°C for 12 hrs. LCMS showed **Compound 1** was consumed and 5% of desired product was detected. The reaction was concentrated under vacuum. The residue was diluted with EtOAc(100 mL) and 1 N HCl (100 mL), the organic layer was separated and discarded and the aqueous layer was basified with Na_2CO_3 solid to pH 8, then the aqueous layer was extracted with EtOAc (100 mL×3), the organic layer was combined and dried over anhydrous Na_2SO_4, filtered and the filtrate was concentrated under vacuum to afford ***Compound 2*** (5.14 g, 21.57 mmol, 46.73% yield) as a yellow oil and it was used directly. MS-ESI (m/z) calcd for $C_{12}H_{18}O_3N_2$ $[M+H]^+$: 239.1. Found 239.2. To a solution of **Compound 2** (0.5 g, 2.10 mmol) in AcOH (20 mL) was added PtO_2 (285.90 mg, 1.26 mmol) at 20 °C, then the mixture was stirred at 60 °C under H_2 for 12 hrs at 15 Psi. TLC showed **Compound 2** was consumed and a new spot was formed (Petroleum ether: Ethyl acetate=1:1) (P1 RF=0.00). The reaction was filtered and the filtrate was concentrated under vacuum to afford ***Compound 3*** (1 g, crude) as a yellow liquid and it was used directly. MS-ESI (m/z) calcd for $C_{12}H_{24}O_3N_2$ $[M+H]^+$: 245.2. Found 245.1. To a solution of **Compound 3** (800 mg, 3.27 mmol) and $NaHCO_3$ (550.12 mg, 6.55 mmol, 254.69 uL) in ACN (1 mL) and H_2O (1 mL) was added CbzCl (558.57 mg, 3.27 mmol, 465.47 uL) drop wised at 0°C. Then the mixture was stirred at 20°C for 1 hr. LC-MS showed **Compound 3** was consumed completely and 43% desired mass was detected. The reaction mixture was diluted with H_2O 10 mL and extracted with EtOAc (5 mL×3). The combined organic layers were dried over Na_2SO_4, filtered and concentrated under reduced pressure to give a residue. The residue was purified by flash silica gel chromatography (ISCO®; 4 g SepaFlash® Silica Flash Column, Eluent of 0~50% Ethyl acetate/Petroleum ether gradient @ 50 mL/min) (Petroleum ether/Ethyl acetate=3/1, P1Rf=0.1) to afford ***Compound 4*** (630 mg, 1.66 mmol, 50.84% yield) as a light yellow oil. MS-ESI (m/z) calcd for $C_{20}H_{30}O_5N_2$ $[M+H]^+$: 379.2. Found 379.2. To a solution of **Compound 4** (200 mg, 528.46 umol) in DCM (2 mL) was added TFA (1.54 g, 13.51 mmol, 1 mL) and the mixture was stirred at 20°C for 0.5 hr. LC-MS showed **Compound 4** was

consumed completely and one main peak with desired mass was detected. The reaction mixture was concentrated under reduced pressure to remove solvent. The residue was diluted with sat.$NaHCO_3$ 10 mL and extracted with DCM (5 mL×3). The combined organic layers were dried over Na_2SO_4, filtered and concentrated under reduced pressure to afford ***Compound 5*** (150 mg, crude) as a light yellow gum. MS-ESI (m/z) calcd for $C_{15}H_{22}O_3N_2$ $[M+H]^+$: 279.2. Found 279.2.

A mixture of **Compound 5** (150 mg, 538.90 umol), **Compound 5A** (78.99 mg, 538.90 umol, HCl salt) and TEA (109.06 mg, 1.08 mmol, 150.02 uL) in ACN (2 mL) was stirred at 80°C for 1 hr under N_2 atmosphere. LC-MS showed **Compound 5** was consumed completely and 21% desired product was detected. The reaction mixture was concentrated under reduced pressure to remove solvent to afford ***Compound 6*** (175 mg, crude) as a colorless oil. MS-ESI (m/z) calcd for $C_{16}H_{24}O_3N_4$ $[M+H]^+$: 321.2. Found 321.2. To a solution of **Compound 6** (175 mg, 546.22 umol) in EtOH (2 mL) was added NaOEt (74.34 mg, 1.09 mmol) and the mixture was stirred at 80°C for 0.5 hr, then **Compound 6A** (177.09 mg, 546.22 umol) was added, and the resulting mixture was stirred at 80°C for 1 hr. LC-MS showed **Compound 6** was consumed completely and one main peak with desired mass was detected. The reaction mixture was concentrated under reduced pressure to remove solvent. The residue was purified by flash silica gel chromatography (ISCO®; 12 g SepaFlash® Silica Flash Column, Eluent of 0~100% Ethyl acetate/Petroleum ether gradient @ 75 mL/min) (Petroleum ether/Ethyl acetate=0/1, P1Rf=0.2) to afford ***Compound 7*** (200 mg, 343.94 umol, 62.97% yield) as a brown solid. MS-ESI (m/z) calcd for $C_{29}H_{30}Cl_2N_6O_3$ $[M+H]^+$: 581.2/583.2. Found 581.2/583.2. To a solution of **Compound 7** (70 mg, 120.38 umol) in DCM (1 mL) was added TMSI (48.17 mg, 240.76 umol, 32.77 uL) at 0°C, and the mixture was stirred at 20°C for 1hr. LC-MS showed **Compound 7** was consumed completely and one main peak with desired mass was detected. The reaction mixture was concentrated under reduced pressure to remove solvent. The residue was purified by Prep-HPLC (HCl condition) (column: Phenomenex luna C18 80*40mm*3um; mobile phase: [water(0.04%HCl)-ACN];B%: 1%-20%,7min) to afford ***analog 18*** (40.10 mg, 82.88 umol, 68.85% yield, 100% purity, HCl salt) as a yellow solid. **^{1}H NMR** (METHANOL-d_4 400MHz) δ ppm 8.71 (s, 1 H) 7.62 - 7.66 (m, 1 H) 7.52 - 7.59 (m, 2 H) 7.24 (d, J=0.98 Hz, 1 H) 3.74 (s, 5 H) 3.32 - 3.37 (m, 1 H) 3.32 - 3.37 (m, 1 H) 3.27 - 3.30 (m, 1 H) 3.04 - 3.16 (m, 2 H) 2.32 (d, J=0.98 Hz, 3 H) 1.97 - 2.08 (m, 2 H) 1.84 - 1.95 (m, 2 H). **LCMS** (ESI+): *m/z* 447.1/449.1 (M+H)

Analog 19

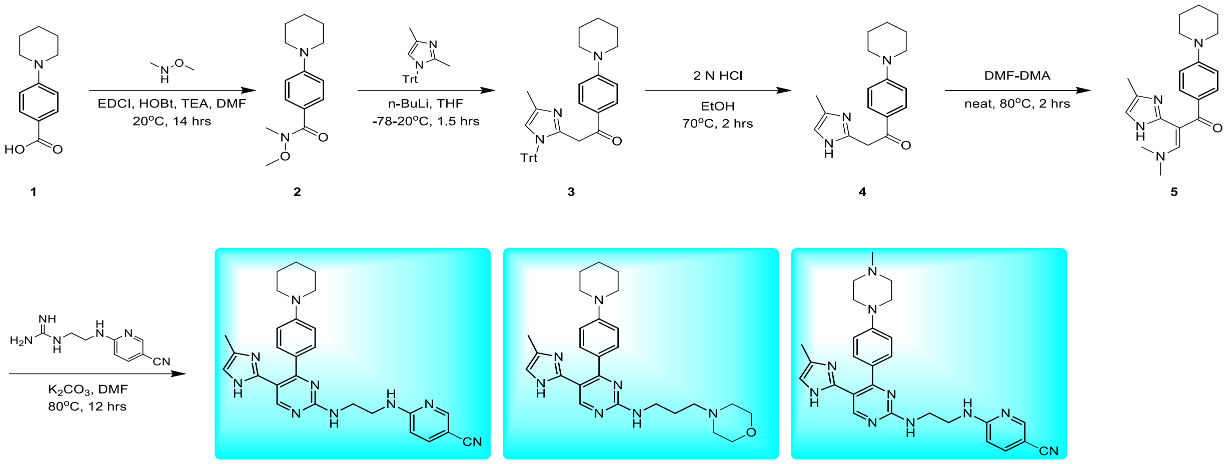

Analog 19 A-49 A-9

N^1-(4-(2,4-dichlorophenyl)-5-(4-methyl-1*H*-imidazol-2-yl)pyrimidin-2-yl)-N^2-(tetrahydro-2*H*-pyran-4-yl)ethane-1,2-diamine (Analog 19). A mixture of **Compound 1** (1 g, 9.99 mmol, 917.43 uL) and **Compound 1A** (1.84 g, 11.49 mmol, 1.80 mL) in DCE (20 mL) was stirred at 20°C for 1 hr, then AcOH (59.98 mg, 998.84 umol, 57.13 uL) was added, and the resulting mixture was stirred at 20°C for 1 hr, then $NaBH(OAc)_3$ (4.23 g, 19.98 mmol) was added and the resulting mixture was stirred at 20°C for 12 hrs. LC-MS showed **Compound 1** was consumed completely and 21% of desired compound was detected. The reaction mixture was diluted with NH_4OH(10% in H_2O) 30 mL and extracted with DCM (10 mL × 3), dried over Na_2SO_4, filtered and concentrated under reduced pressure to afford ***Compound 2*** (2.9 g, crude) as a yellow oil. MS-ESI (m/z) calcd for $C_{12}H_{24}N_2O_3$ $[M+H]^+$: 245.2. Found 245.1. A mixture of **Compound 2** (1 g, 4.09 mmol) in HCl/EtOAc (10 mL, 4M) and EtOAc (2 mL) was stirred at 20°C for 1 hr. LC-MS showed 57% of **Compound 2** was remained and 24% of desired compound was detected. Then the mixture was stirred at 20°C for another 24hrs. LC-MS showed **Compound 2** was consumed completely and 80% of desired compound was detected. The reaction mixture was filtered, the filter cake was washed with ethyl acetate(10 mL × 2), then the filtered cake was dried under reduced pressure to afford ***Compound 3*** (550 mg, crude, HCl salt) as a white solid. MS-ESI (m/z) calcd for $C_7H_{16}N_2O$ $[M+H]^+$: 145.1. Found 145.2. To a solution of **Compound 3** (50 mg, 276.74 umol, HCl salt) in ACN (2 mL) was added TEA (84.01 mg, 830.22 umol, 115.56 uL) and **Compound 3A** (48.68 mg, 332.09 umol) at 20°C. The mixture was stirred at 80°C for 12hrs. LC-MS showed **Compound 3** was consumed completely and one main peak with desired mass was detected. The reaction mixture was concentrated under reduced pressure to remove solvent to afford ***Compound 4*** (110 mg, crude) as a brown solid. MS-ESI (m/z) calcd for $C_8H_{18}N_4O$ $[M+H]^+$: 187.2. Found 187.5 To a solution of **Compound 4** (55 mg, 295.30 umol) in THF (2 mL) was added Cs_2CO_3 (288.64 mg, 885.89 umol) and **Compound 4A** (95.74 mg, 295.30 umol) at 20°C. The mixture was stirred at 80°C for 2hrs. LC-MS showed **Compound 4A** was consumed completely and one main peak with desired mass was detected. The reaction mixture was filtered to remove the insoluble, and the filtrate was concentrated under reduced pressure to give a residue. The residue was purified by prep-HPLC (HCl condition; column: Welch Xtimate C18 150*25mm*5um; mobile phase: [water (0.04%HCl)-ACN]; B%: 1%-25%, 8min) to afford ***analog 19*** (26.39 mg, 58.08 umol, 19.67% yield, 98.45% purity) as a pale yellow solid. **^{1}H NMR** (METHANOL-d_4 400MHz) δ ppm 8.73 (s, 1 H) 7.49 - 7.75 (m, 3 H) 7.24 (br s, 1 H) 3.87 - 4.06 (m, 4 H) 3.42 - 3.54 (m, 2 H) 3.37 (br s, 3 H) 2.32 (s, 3 H) 1.94 - 2.13 (m, 2 H) 1.61 - 1.82 (m, 2 H). **LCMS** (ESI+): *m*/*z* 447.1 (M+H).

Analog 20

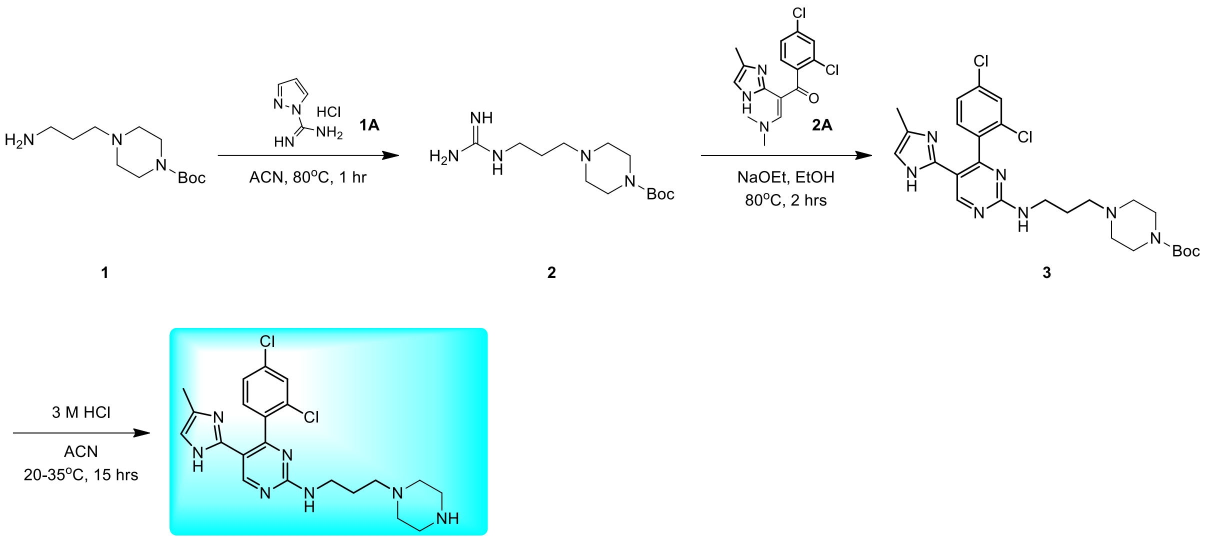

Analog 20

4-(2,4-dichlorophenyl)-5-(4-methyl-1*H*-imidazol-2-yl)-*N*-(3-(piperazin-1-yl)propyl)-pyrimidin-2-amine (Analog 20). A solution of **Compound 1** (200 mg, 821.88 umol) and **Compound 1A** (120.47 mg, 821.88 umol) in ACN (4 mL) was stirred at 80°C for 1 hr. LCMS showed the **Compound 1** was consumed and 99% of desired product was detected. The reaction was concentrated under vacuum to afford ***Compound 2*** (270 mg, crude, HCl salt) as a colorless liquid. MS-ESI (m/z) calcd for $C_{13}H_{27}N_5O_2$ $[M+H]^+$: 286.2. Found 286.4 To a solution of **Compound 2** (135 mg, 419.46 umol, HCl salt) in EtOH (3 mL) was added EtONa (28.54 mg, 419.46 umol) at 20°C, then the mixture was stirred at 80°C for 10 min, then another solution of **Compound 2A** (135.99 mg, 419.46 umol) in EtOH (3 mL) was added at 80°C, then the mixture was stirred at 80°C for 2 hrs. LCMS showed the **Compound 2** was consumed and 38% of desired product was detected. The reaction was concentrated under vacuum. The residue was diluted with water (8 mL), then the aqueous layer was extracted with EtOAc (8 mL × 3), the organic layer was dried over anhydrous Na_2SO_4, filtered and the filtrate was concentrated under vacuum to afford ***Compound 3*** (173 mg, crude) as a brown liquid and it was used directly. MS-ESI (m/z) calcd for $C_{26}H_{33}Cl_2N_7O_2$ $[M+H]^+$: 546.2. Found 546.0 To a solution of **Compound 3** (271 mg, 495.89 umol) in ACN (6 mL) was added HCl (3 mL) (3 M) at 20°C, then the mixture was stirred at 20°C for 12 hrs. LCMS showed 17% of **Compound 3** was remained, then the reaction was stirred at 35°C for 3 hrs. HPLC showed **Compound 3** was consumed and 70% of desired product was detected. The reaction was filtered. The filtrate was purified by Prep-HPLC (HCl condition) (column: Phenomenex luna C18 80*40mm*3 um; mobile phase: [water(0.04%HCl)-ACN]; B%: 1%-25%, 7 min) to afford ***analog 20*** (27.37 mg, 55.98 umol, 11.29% yield, 98.75% purity, HCl salt) as a pale yellow solid. **^{1}H NMR** (METHANOL-d_4 400MHz) δ ppm 8.60 - 8.71 (m, 1 H) 7.60 - 7.77 (m, 1 H) 7.56 (brs, 1 H) 7.52 (brs, 1 H) 7.21 (d, *J*=0.88 Hz, 1 H) 3.85 (brs, 1 H) 3.66 (brs, 7 H) 3.34 - 3.55 (m, 4 H) 2.31 (d, *J*=0.88 Hz, 3 H) 2.17 - 2.26 (m, 2 H). **LCMS** (ESI+): *m*/*z* 446.2/448.2 (M+H).

Analog 21

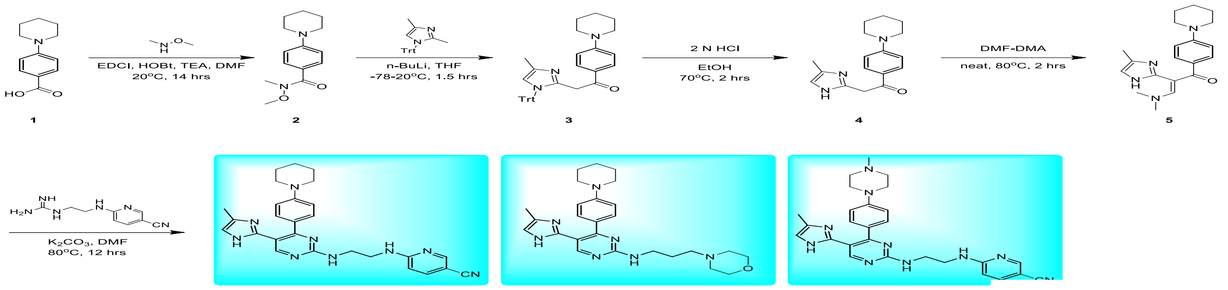

A-8 A-49 A-9

Analog 21

4-(2,4-dichlorophenyl)-5-(4-methyl-1*H*-imidazol-2-yl)-*N*-(3-morpholinopropyl)pyrimidin-2-amine (Analog 21). A mixture of **Compound 1** (200 mg, 1.39 mmol, 202.63 uL) and **Compound 1A** (203.28 mg, 1.39 mmol) in ACN (3 mL) was stirred at 80°C for 12 hrs. LC-MS showed **Compound 1** was consumed completely and one main peak with desired mass was detected. The reaction mixture was concentrated under reduced pressure to remove solvent to afford ***Compound 2*** (270 mg, crude) as a yellow oil. MS-ESI (m/z) calcd for $C_8H_{18}N_4O$ $[M+H]^+$: 187.2. Found 187.4. To a solution of **Compound 2** (210 mg, 1.13 mmol) in EtOH (2 mL) was added EtONa (76.73 mg, 1.13 mmol) at 20°C, and the mixture was stirred at 80°C for 10 min, then **Compound 2A**(365.54 mg, 1.13 mmol) in EtOH (2 mL) was added at 80°C. The mixture was stirred at 80°C for 2 hrs. LC-MS showed **Compound 2A** was consumed completely and one main peak with desired mass was detected. The reaction was filtered to remove the insoluble, and the filtrate was concentrated under reduced pressure to give a residue. The residue was purified together with another batch by prep-HPLC (neutral condition; column: Waters Xbridge Prep OBD C18 150*40mm*10um; mobile phase: [water (10mM NH_4HCO_3)-ACN]; B%: 25%-55%, 8min) to afford ***analog 21*** (111.30 mg, 100% purity) as a pale yellow solid. **^{1}H NMR** (METHANOL-d_4 400MHz) δ ppm 8.50 (s, 1 H) 7.40-7.42 (m, 3 H) 6.63 (s, 1 H) 3.67 (br s, 4 H) 3.47 - 3.50 (m, 2 H) 2.47 (s, 6 H) 1.83 - 1.87 (m, 2 H). **LCMS** (ESI+): *m/z* 447.2 (M+H)

Analog 22

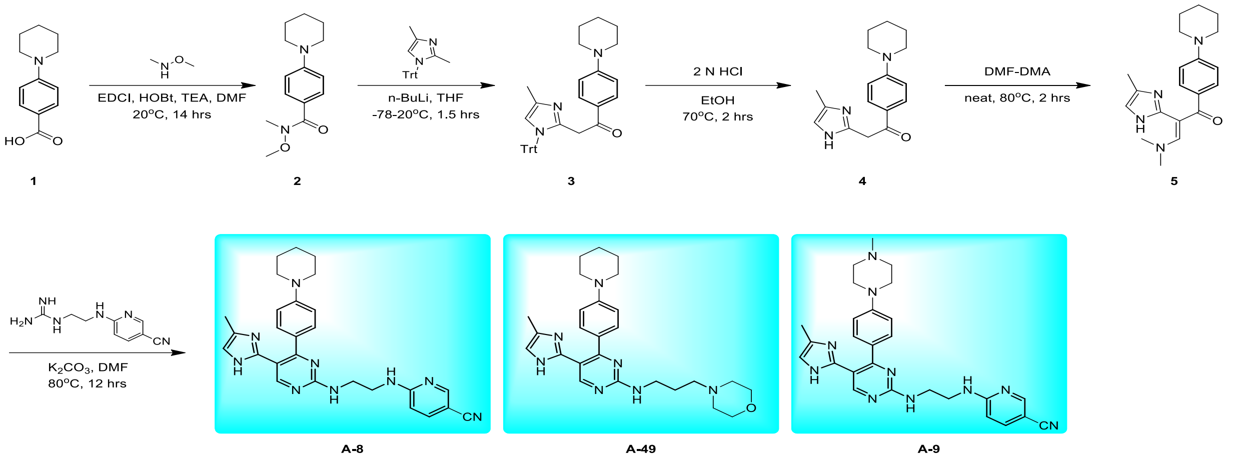

Analog 22

4-(2,4-dichlorophenyl)-5-(4-methyl-1*H*-imidazol-2-yl)-*N*-(2-(oxazol-2-yloxy)ethyl)-pyrimidin-2-amine (analog 22). To a solution of **Compound 1** (5 g, 37.27 mmol) and KSCN (5.43 g, 55.90 mmol, 5.43 mL) in EtOH (50 mL) was added HCl (11.02 g, 111.80 mmol, 10.80 mL, 37% purity) at -5°C. Then the mixture was stirred at 80°C for 12 hrs. TLC indicated that one main spot, which was P1, was formed (Petroleum ether/Ethyl acetate=1/1, P1Rf=0.3). The reaction mixture was concentrated under reduced pressure to remove solvent. The residue was purified by flash silica gel chromatography (ISCO®; 20 g SepaFlash® Silica Flash Column, Eluent of 0~30% Ethyl acetate/Petroleum ether gradient @ 75 mL/min) (Petroleum ether/Ethyl acetate=1/1, P1Rf=0.3) to afford ***Compound 2*** (3.2 g, 31.64 mmol, 84.91% yield) as a light yellow solid. To a solution of **Compound 2** (1 g, 9.89 mmol) in ACN (20 mL) was added K_2CO_3 (4.10 g, 29.67 mmol) and MeI (4.21 g, 29.67 mmol, 1.85 mL). The mixture was stirred at 20°C for 1 hrs. TLC indicated that **Compound 2** was consumed, and one main spot was formed (Petroleum ether/Ethyl acetate=1/1, P1Rf=0.8). The reaction mixture was filtered and the filtrate was concentrated under reduced pressure under at low temperature to remove solvent. Then the residue was diluted with H_2O 30 mL and extracted with DCM (10 mL × 3). The combined organic layers were dried over Na_2SO_4, filtered and the filtrate was used to the next step without further purification. ***Compound 3*** (1.2 g, crude) was obtained as a light yellow liquid in DCM (30 mL). To a solution of **Compound 3** (1 g, 8.68 mmol) in DCM (30 mL) was added m-CPBA (4.41 g, 21.71 mmol, 85% purity) at 0°C, then the mixture was stirred at 20°C for 12 hrs. TLC indicated that **Compound 3** was consumed, and two new spots were formed (Petroleum ether/Ethyl acetate=1/1, P1Rf=0.4). The reaction mixture was quenched with 50 ml 10% Na_2SO_3 solution at 20 °C, and the mixture was stirred for 10 min, and then separated, and the aqueous phase was extracted with DCM (10 mL × 3). The combined organic layers were washed with sat. $NaHCO_3$ (30 mL × 1), dried over Na_2SO_4, filtered and concentrated under reduced pressure to give a residue. The residue was purified by flash silica gel chromatography (ISCO®; 4 g SepaFlash® Silica Flash Column, Eluent of 0~40% Ethyl acetate/Petroleum ether gradient @ 75 mL/min) (Petroleum ether/Ethyl acetate=1/1, P1Rf=0.4) to afford ***Compound 4*** (750 mg, 5.10 mmol, 58.69% yield) as a colorless oil. A mixture of Compound 4 (700 mg, 4.76 mmol) in ethane-1,2-diamine (6.29 g, 104.60 mmol, 7 mL) was stirred at 70°C for 2 hrs under N_2 atmosphere. LC-MS showed **Compound 4** was consumed completely and one main peak with desired MS was detected. The reaction mixture was concentrated under reduced pressure to remove solvent. The residue was purified by prep-HPLC (basic condition) (column: Waters Xbridge Prep OBD

C18 150*40mm*10um; mobile phase: [water (0.05%NH_3H_2O+10mM NH_4HCO_3)-ACN]; B%: 1%-5%, 8min) to afford ***Compound 5*** (300 mg, 2.36 mmol, 49.60% yield) as a brown oil. MS-ESI (m/z) calcd for $C_5H_9N_3O$ $[M+H]^+$: 128.1. Found 128.3. To a solution of **Compound 5** (100 mg, 786.51 umol) in DMF (2 mL) was added **Compound 5A** (115.28 mg, 786.51 umol). The mixture was stirred at 80°C for 1 hr. LC-MS showed **Compound 5** was consumed completely and one main peak with desired mass was detected. The reaction mixture was concentrated under reduced pressure to remove solvent. The residue was purified by prep-HPLC (basic condition) (column: Waters Xbridge Prep OBD C18 150*40mm*10um; mobile phase: [water (0.04%NH_3H_2O+10mM NH_4HCO_3)-ACN]; B%: 1%-20%, 8min) to afford ***Compound 6*** (40 mg, 236.43 umol, 30.06% yield) as a white solid. MS-ESI (m/z) calcd for $C_6H_{11}N_5O$ $[M+H]^+$: 170.1. Found 170.2. To a solution of **Compound 6** (10 mg, 59.11 umol) in EtOH (1 mL) was added NaOEt (8.04 mg, 118.21 umol) and the mixture was stirred at 80°C for 0.5 hr, then **Compound 6A** (19.16 mg, 59.11 umol) was added, and the resulting mixture was stirred at 80°C for 1 hr. LC-MS showed **Compound 6** was consumed completely and one main peak with desired mass was detected. The reaction mixture was concentrated under reduced pressure to remove solvent. The residue was purified by prep-HPLC (basic condition) (column: Waters Xbridge BEH C18 100*30mm*10um; mobile phase: [water (0.04%NH_3H_2O+10mM NH_4HCO_3)-ACN]; B%: 15%-45%, 6min). But LCMS showed that the purity of the residue was not high enough, so the mixture was purified further by Prep-HPLC (TFA condition) (column: Phenomenex Luna C18 100*30mm*5um; mobile phase: [water (0.1%TFA)-ACN]; B%: 1%-30%, 10min) to afford ***analog 22*** (16.8 mg, 29.79 umol, 25.20% yield, 96.51% purity, TFA salt) as a colorless gum. **^{1}H NMR** (METHANOL-d_4 400MHz) δ ppm 8.64 (d, J=15.21 Hz, 1 H) 8.17 - 8.57 (m, 2 H) 7.65 (s, 1 H) 7.47 - 7.61 (m, 3 H) 7.35 (d, J=0.88 Hz, 1 H) 7.09 (br d, J=7.28 Hz, 1 H) 3.51 - 3.63 (m, 2 H) 3.40 - 3.50 (m, 2 H) 2.22 (s, 3 H). **LCMS** (ESI+): m/z 430.0/432.0 (M+H)

Analog 23

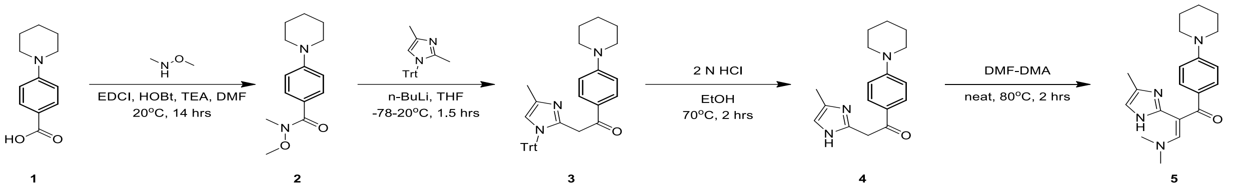

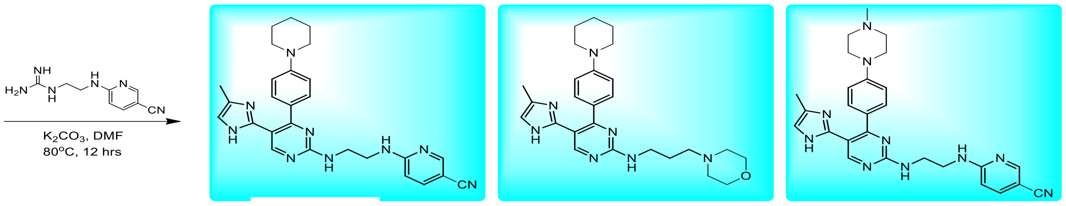

Analog 23 A-49 A-9

4-(2,4-dichlorophenyl)-5-(4-methyl-1*H*-imidazol-2-yl)-*N*-(2-(thiazol-2-yloxy)ethyl)-pyrimidin-2-amine (Analog 23). NaH (6.10 g, 152.42 mmol, 60% purity) was added to a solution of **Compound 1A** (49.14 g, 304.83 mmol, 47.25 mL) in DMSO (5 mL) at 20°C. The mixture was degassed and purged with N_2 for 3 times, it was stirred at 20°C for 30 min, then **Compound 1** (5 g, 30.48 mmol, 2.75 mL) was added, the resulted mixture was stirred at 40°C for 12 hrs. LCMS showed 34% **Compound 1** was remained and 32% desired compound was detected, and then it was stirred at 40°C for another 12 hrs. LCMS showed 34% **Compound 1** was remained and 37% desired compound was detected. The reaction was poured into saturated NH_4Cl solution (100 mL), then the mixture was extracted with EtOAc (80 mL × 3), the organic layer was dried over anhydrous Na_2SO_4, filtered and the filtrate was concentrated under vacuum. The residue was purified by flash silica gel chromatography (ISCO®; 80 g SepaFlash® Silica Flash Column, Eluent of 0~20% Ethyl acetate/Petroleum ether gradient @ 100 mL/min) (petroleum ether: ethyl acetate=3:1, P1 Rf=0.18) to afford ***Compound 2*** (4.2 g, 17.19 mmol, 56.40% yield) as a light yellow solid. MS-ESI (m/z) calcd for $C_{10}H_{16}N_2O_3S$ $[M+H]^+$: 245.1. Found 245.4. To a solution of **Compound 2** (2 g, 8.19 mmol) in DCM (40 mL) was added TFA (9.33 g, 81.86 mmol, 6.06 mL). The mixture was stirred at 20°C for 12 hrs. LCMS showed **Compound 2** was consumed completely and 83.9% of desired compound was detected. The reaction mixture was concentrated under reduced pressure to remove solvent to afford ***Compound 3*** (2 g, crude, TFA salt) as a yellow oil. MS-ESI (m/z) calcd for $C_5H_8N_2OS$ $[M+H]^+$: 145.0. Found 145.1. To a solution of **Compound 3** (200 mg, 774.54 umol, TFA salt) in ACN (5 mL) was added DIEA (200.21 mg, 1.55 mmol, 269.82 uL) and **Compound 3A** (113.53 mg, 774.54 umol) at 20°C. The mixture was stirred at 80°C for 13 hrs. TLC (ethyl acetate: methanol=10:1, P1 Rf=0.00) showed the **Compound 3** was consumed completely and there was a new spot formed. The reaction mixture was concentrated to afford ***Compound 4*** (280 mg, crude) as a yellow oil. The reaction was carried out in 5 parallel batches. To a solution of **Compound 4** (30 mg, 161.09 umol) in THF (2 mL) was added Cs_2CO_3 (157.46 mg, 483.26 umol) and **Compound 4A** (52.23 mg, 161.09 umol) at 20°C. The mixture was stirred at 80°C for 2 hrs. LCMS showed trace **Compound 4** was remained and one main peak with desired mass was detected. The reaction mixture was filtered; the filtrate was concentrated to give a residue. The residue was purified by Prep-HPLC (neutral condition) (column: Phenomenex Gemini-NX 150*30mm*5um; mobile phase: [water (10mM NH_4HCO_3)-ACN]; B%: 25%-45%, 8 min) to afford ***analog 23*** (60.30 mg, 133.68 umol, 16.60% yield, 99.17% purity) as a yellow solid. **^{1}H NMR** (METHANOL-d_4 400MHz) δ ppm 8.53 (s, 1 H) 7.38 - 7.45 (m, 3 H) 7.11 (d, *J*=3.79 Hz, 1 H) 6.87 (d, *J*=3.79 Hz, 1 H) 6.65 (brs, 1 H) 4.59 (t, *J*=5.38 Hz, 2 H) 3.88 (t, *J*=5.20 Hz, 2 H) 2.15 (s, 3 H). **LCMS** (ESI+): *m/z* 447.1/449.1 (M+H)

Analog 24, 44

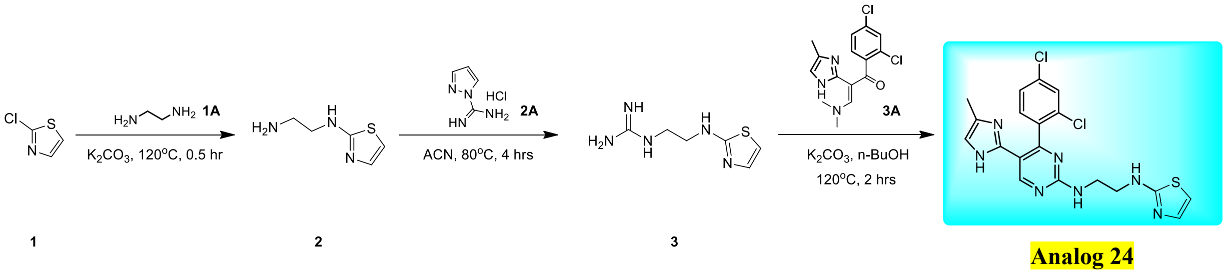

Analog 24

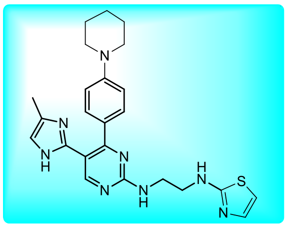

Analog 44

N^1-(4-(2,4-dichlorophenyl)-5-(4-methyl-1*H*-imidazol-2-yl)pyrimidin-2-yl)-N^2-(thiazol-2-yl)ethane-1,2-diamine (Analog 24): To a solution of **Compound 1** (100 mg, 836.31 umol, 274.73 uL) in **Compound 1A** (3.70 g, 61.49 mmol, 4.12 mL) was added K_2CO_3 (231.17 mg, 1.67 mmol) at 20°C, then the mixture was stirred at 120°C for 0.5 hr under microwave. LCMS showed the **Compound 1** was consumed and 72% of desired product was detected. The reaction was combined with another batch. The reaction was filtered and the filter-cake was washed with DMF (5 mL), the filtrate was concentrated under vacuum. The residue was diluted with DMF (5 mL) and concentrated under vacuum to remove excess **Compound 1A** to afford ***Compound 2*** (309 mg, crude) as a yellow liquid. MS-ESI (m/z) calcd for $C_5H_9N_3S$ $[M+H]^+$: 144.1. Found 144.5**.** A solution of **Compound 2** (219 mg, 1.53 mmol) and **Compound 2A** (224.15 mg, 1.53 mmol) in ACN (6 mL) was stirred at 80°C for 4 hrs. LCMS showed **Compound 2** was consumed and 88% of desired product was detected. The reaction was concentrated under vacuum to afford ***Compound 3*** (350 mg, crude, HCl salt) as a brown liquid and it was used directly. MS-ESI (m/z) calcd for $C_6H_{11}N_5S$ $[M+H]^+$: 186.1. Found 186.4. To a solution of **Compound 3** (200 mg, 902.08 umol, HCl salt) in n-BuOH (6 mL) was added K_2CO_3 (374.02 mg, 2.71 mmol,) and **Compound 3A** (292.46 mg, 902.08 umol) at 20°C, then the mixture was stirred at 120°C for 2 hrs. LCMS showed the **Compound 3** was consumed and 11% of desired product was detected. The reaction was filtered. The filtrate was purified by Prep-HPLC (basic condition) (column: Waters Xbridge Prep OBD C18 150*40mm*10um; mobile phase: [water(0.05%NH_3H_2O+10mM NH_4HCO_3)-ACN]; B%: 20%-50%, 8 min), the HNMR was not clean, so the residue was further purified by Prep-HPLC (HCl condition) (column: Phenomenex luna C18 80*40mm*3 um; mobile phase: [water (0.04%HCl)-ACN]; B%: 10%-30%, 7 min) to afford ***analog 24*** (20.33 mg, 42.11 umol, 4.67% yield, 100% purity, HCl salt) as a pale yellow solid. **^{1}H NMR** (METHANOL-d_4 400MHz) δ ppm 8.71 (s, 1 H) 8.11 (br s, 1 H) 7.51 - 7.61 (m, 3 H) 7.26 (s, 1 H) 7.18 (br d, *J*=3.97 Hz, 1 H) 6.80 (br d, *J*=3.31 Hz, 1 H) 3.67 (br s, 2 H) 3.62 (br d, *J*=5.07 Hz, 2 H) 2.24 (s, 3 H). **LCMS** (ESI+): *m/z* 446.1/448.1 (M+H)

Analog 25

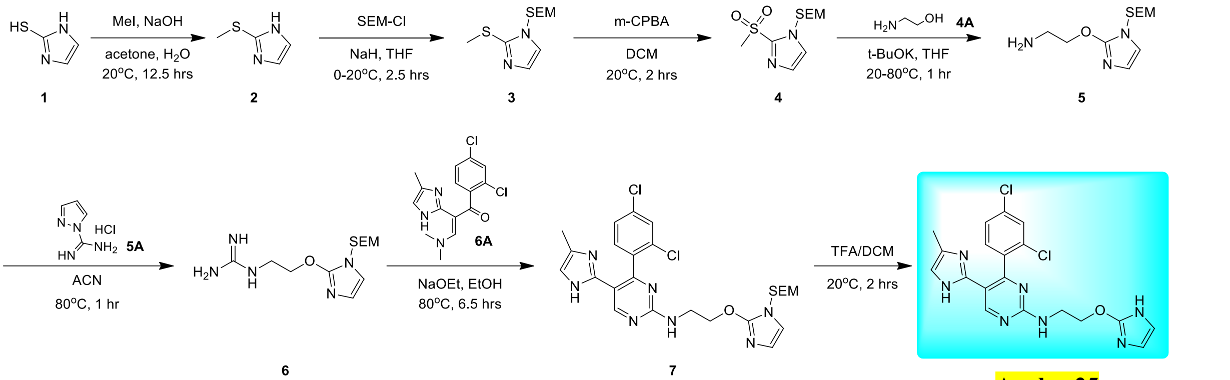

Analog 25

N*-(2-((1*H*-imidazol-2-yl)oxy)ethyl)-4-(2,4-dichlorophenyl)-5-(4-methyl-1*H*-imidazol-2-yl)pyrimidin-2-amine (Analog 25).** To a solution of **Compound 1** (4.5 g, 44.94 mmol) in Water (180 mL) was added NaOH (2.16 g, 54.00 mmol), the mixture was stirred at 20°C for 0.5 hr, then another solution of MeI (6.98 g, 49.15 mmol, 3.06 mL) in acetone (180 mL) was added, then the mixture was stirred at 20°C for 12 hrs. LCMS showed **Compound 1** was consumed and 99% of desired product was detected. The acetone was removed under vacuum, the aqueous layer was extracted with EtOAc (150 mL × 3), the combined organic layer was dried over anhydrous Na_2SO_4, filtered and the filtrate was concentrated under vacuum. The residue was washed with Petroleum ether, the mixture was filtered and the filter-cake was dried under vacuum to afford ***Compound 2 (3.12 g, 27.33 mmol, 60.82% yield) as a light yellow solid and it was used to next step directly. MS-ESI (m/z) calcd for $C_4H_6N_2S$ $[M+H]^+$: 115.0. Found 115.0. To a solution of **Compound 2** (1 g, 8.76 mmol) in THF (20 mL) was added NaH (385.36 mg, 9.63 mmol, 60% purity) at 0°C, then the mixture was stirred at 20°C for 0.5 hr, then SEM-Cl (1.46 g, 8.76 mmol, 1.55 mL) was added dropwise at 0°C, then the mixture was stirred at 20°C for 2 hrs. TLC (Petroleum ether: Ethyl acetate=1:1) (P1 RF=0.41) showed **Compound 2** was consumed and a new spot was formed. Saturated NH_4Cl solution (15 mL) was added dropwised to the reaction at 0°C, then the mixture was extracted with EtOAc (10 mL × 3), the organic layer was dried over anhydrous Na_2SO_4, filtered and the filtrate was concentrated under vacuum. The residue was purified by flash silica gel chromatography (ISCO®; 4 g SepaFlash® Silica Flash Column, Eluent of 0~25% Ethyl acetate/Petroleum ether gradient @ 36 mL/min) to afford ***Compound 3*** (1.89 g, 7.73 mmol, 88.28% yield) as a pale yellow oil. To a solution of **Compound 3** (1.89 g, 7.73 mmol) in DCM (50 mL) was added m-CPBA (3.45 g, 17.01 mmol, 85% purity) at 0°C, then the mixture was stirred at 20°C for 2 hrs. LCMS showed **Compound 3** was consumed and 30% of desired product was detected. The reaction was cooled to 0°C and 16 mL 10% Na_2SO_3 solution was added drop wised, then the mixture was stirred at 20°C for 10 min, then the mixture was added saturated Na_2CO_3 solution (15 mL), the organic layer was separated, and the aqueous layer was extracted with EtOAc (20 mL ×2), the organic layer was dried over anhydrous Na_2SO_4, filtered and the filtrate was concentrated under vacuum. The residue was purified by flash silica gel chromatography (ISCO®; 40 g SepaFlash® Silica Flash Column, Eluent of 0~25% Ethyl acetate/Petroleum ether gradient @ 75 mL/min) to afford ***Compound 4*** (1.72 g, 6.22 mmol, 80.47% yield) as a colorless oil. MS-ESI (m/z) calcd for $C_{10}H_{20}N_2O_3SSi$ $[M+H]^+$: 277.1. Found 277.1 To a solution of **Compound 4** (100 mg, 361.76 umol) and t-BuOK (162.37 mg, 1.45 mmol) in THF (4 mL) was added **Compound 4A** (88.39 mg, 1.45 mmol, 87.51 uL) at 20°C, then the mixture was stirred at 80°C for 1 hr. LCMS showed **Compound 4** was consumed and 60% of desired product was detected. The mixture was diluted with water (15 mL), the aqueous layer was extracted with EtOAc（15 mL × 3）, the organic layer was dried over anhydrous Na_2SO_4, filtered and the filtrate was concentrated under

vacuum to afford ***Compound 5*** (379 mg, crude) as a yellow liquid and it was used directly. MS-ESI (m/z) calcd for $C_{11}H_{23}N_3O_2Si$ $[M+H]^+$: 258.2. Found 258.1. To a solution of **Compound 5** (75.8 mg, 294.48 umol) in ACN (2 mL) was added **Compound 5A** (43.16 mg, 294.48 umol) at 20°C, then the mixture was stirred at 80°C for 1 hr. LCMS showed **Compound 5** was consumed and 41% of desired product was detected. The reaction was concentrated under vacuum. The residue was purified by Prep-HPLC (neutral condition) (column: Waters Xbridge Prep OBD C18 150*40mm*10um; mobile phase: [water (10 mM NH_4HCO_3)-ACN]; B%: 15%-45%, 8 min) to afford ***Compound 6*** (363 mg, 1.21 mmol, 82.33% yield) as a white solid. MS-ESI (m/z) calcd for $C_{12}H_{25}N_5O_2Si$ $[M+H]^+$: 300.2. Found 300.2. To a solution of **Compound 6** (100 mg, 333.95 umol) in EtOH (3 mL) was added EtONa (45.45 mg, 667.91 umol) at 20°C, then the mixture was stirred at 80°C for 15 min, then another solution of **Compound 6A** (108.27 mg, 333.95 umol) in EtOH (3 mL) was added at 80°C, then the mixture was stirred at 80°C for 6.5 hrs. LCMS showed 8% of **Compound 6A** remained and 60% of desired product was detected. The reaction was concentrated under vacuum. The residue was diluted with water (6 mL), the aqueous layer was extracted with EtOAc (6 mL × 3), the organic layer was dried over anhydrous Na_2SO_4, filtered and the filtrate was concentrated under vacuum to afford ***Compound 7*** (132 mg, 235.48 umol, 70.51% yield) as a brown liquid. MS-ESI (m/z) calcd for $C_{25}H_{31}Cl_2N_7O_2Si$ $[M+H]^+$: 560.2/562.2. Found 560.2/562.2. A solution of **Compound 7** (132 mg, 235.48 umol) in DCM (3 mL) and TFA (3 mL) was stirred at 20°C for 2 hrs. LCMS showed 10% of **Compound 7** was remained and 60% of desired product was detected. The reaction was concentrated under vacuum. The residue was purified by Prep-HPLC (basic condition) (column: Waters Xbridge Prep OBD C18 150*40mm*10um; mobile phase: [water (0.05%NH_3H_2O+10mM NH_4HCO_3)-ACN]; B%: 20%-50%, 8 min) to afford ***analog 25*** (27.1 mg, 61.74 umol, 26.22% yield, 98.03% purity) as white solid. ^{1}**H NMR** (METHANOL-d_4 400MHz) δ ppm 8.52 (br s, 1 H) 7.36 - 7.44 (m, 3 H) 6.64 (s, 1 H) 6.57 (br s, 2 H) 4.43 (t, J=5.40 Hz, 2 H) 3.83 (br t, J=5.18 Hz, 2 H) 2.14 (s, 3 H). **LCMS** (ESI+): m/z 430.1/432.1 (M+H)

Analog 26

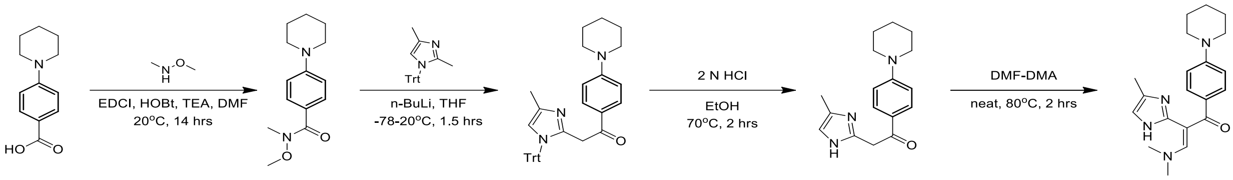

1 2 3 4 5

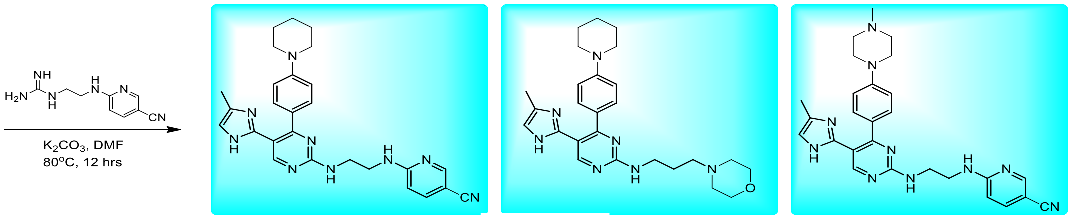

A-8 Analog 26 A-49 A-9

N*[1]-(4-(2,4-dichlorophenyl)-5-(4-methyl-1*H*-imidazol-2-yl)pyrimidin-2-yl)-*N*[2]-(1*H*-imidazol-2-yl)ethane-1,2-diamine (Analog 26).**To a solution of **Compound 1** (200 mg, 1.36 mmol) in THF (8 mL) was added NaH (59.87 mg, 1.50 mmol, 60% purity) at 0°C, then the mixture was stirred at 20°C for 0.5 hr, then SEM-Cl (226.87 mg, 1.36 mmol, 240.84 uL) was added drop wised at 0°C, then the mixture was stirred at 20°C for 2 hrs. LCMS showed 9% of **Compound 1** was remained and 86% of desired product was detected. Saturated NH_4Cl solution (5 mL) was added drop wised to the reaction at 0°C, then the mixture was extracted with EtOAc (5 mL × 3), the organic layer was dried over anhydrous Na_2SO_4 filtered and the filtrate was concentrated under vacuum. The residue was purified by Prep-TLC (Petroleum ether: Ethyl acetate=1:1) (P1 Rf=0.41) to afford ***Compound 2 (256 mg, 923.41 umol, 67.86% yield) as a colorless oil. MS-ESI (m/z) calcd for $C_9H_{17}BrN_2OSi$ $[M+H]^+$: 277.0/279.0. Found 277.1/279.1. To a solution of **Compound 2** (156 mg, 562.70 umol) in ethane-1,2-diamine (2.69 g, 44.83 mmol, 3 mL) was added K_2CO_3 (155.54 mg, 1.13 mmol) at 20°C, then the mixture was stirred at 180°C for 8 hrs under microwave. HPLC showed **Compound 2** was consumed and 72% of desired product was detected. The reaction was filtered and the filter-cake was washed with DMF (10 mL), the filtrate was concentrated under vacuum to afford ***Compound 3*** (172 mg, crude) as a yellow liquid. To a solution of **Compound 3** (172 mg, 670.78 umol) in ACN (4 mL) was added **Compound 3A** (98.32 mg, 670.78 umol) at 20°C, then the mixture was stirred at 80°C for 1 hr. LCMS showed **Compound 3** was consumed and 56% of desired product was detected. The reaction was concentrated under vacuum to afford ***Compound 4*** (230 mg, crude, HCl salt) as a brown liquid and it was used directly. MS-ESI (m/z) calcd for $C_{12}H_{26}N_6OSi$ $[M+H]^+$: 299.2. Found 299.3. To a solution of **Compound 4** (230 mg, 686.73 umol, HCl salt) in EtOH (4 mL) was added EtONa (140.20 mg, 2.06 mmol, 3 eq) at 20°C, then the mixture was stirred at 80°C for 15 min, then another solution of **Compound 4A** (222.64 mg, 686.73 umol) in EtOH (4 mL) was added at 80 °C, then the mixture was stirred at 80°C for 2 hrs. LCMS showed 16% of **Compound 4** was remained and 15% of desired product was detected. Then the reaction was stirred at 80°C for another 2.5 hrs. LCMS showed 17% of **Compound 4** was remained and 15% of desired product was detected. The mixture was concentrated under vacuum. The reaction was purified by Prep-TLC (Dichloromethane: Methanol=10:1) (P1 Rf=0.09) to afford ***Compound 5*** (128 mg, 128.10 umol, 18.65% yield, 56% purity) as a yellow liquid. MS-ESI (m/z) calcd for $C_{25}H_{32}Cl_2N_8OSi$ $[M+H]^+$: 559.2/561.2. Found 559.1/561.1. To a solution of **Compound 5** (128 mg, 228.75 umol) in DCM (4 mL) was added TFA (6.16 g, 54.02 mmol, 4 mL) at 20°C, then the mixture was stirred at 20°C for 2 hrs. LCMS showed **Compound 5** was consumed and 39% of desired product was detected. The reaction was concentrated under vacuum. The residue was purified by Prep-HPLC (HCl condition)(column: Welch Xtimate C18

150*25mm*5um; mobile phase: [water (0.04% HCl)-ACN]; B%: 5%-20%, 8 min) to afford ***analog 26*** (13.7 mg, 28.26 umol, 12.35% yield, 96.08% purity, HCl salt) as a pale yellow gum. **^{1}H NMR** (METHANOL-d_4 400MHz) δ ppm 8.57 - 8.70 (m, 1 H) 7.47 - 7.62 (m, 3 H) 7.21 (s, 1 H) 6.88 (br s, 1 H) 6.68 (br s, 1 H) 3.72 - 3.83 (m, 2 H) 3.51 - 3.65 (m, 2 H) 2.31 (s, 3 H). **LCMS** (ESI+): *m/z* 429.0/431.0 (M+H)

Analog 27

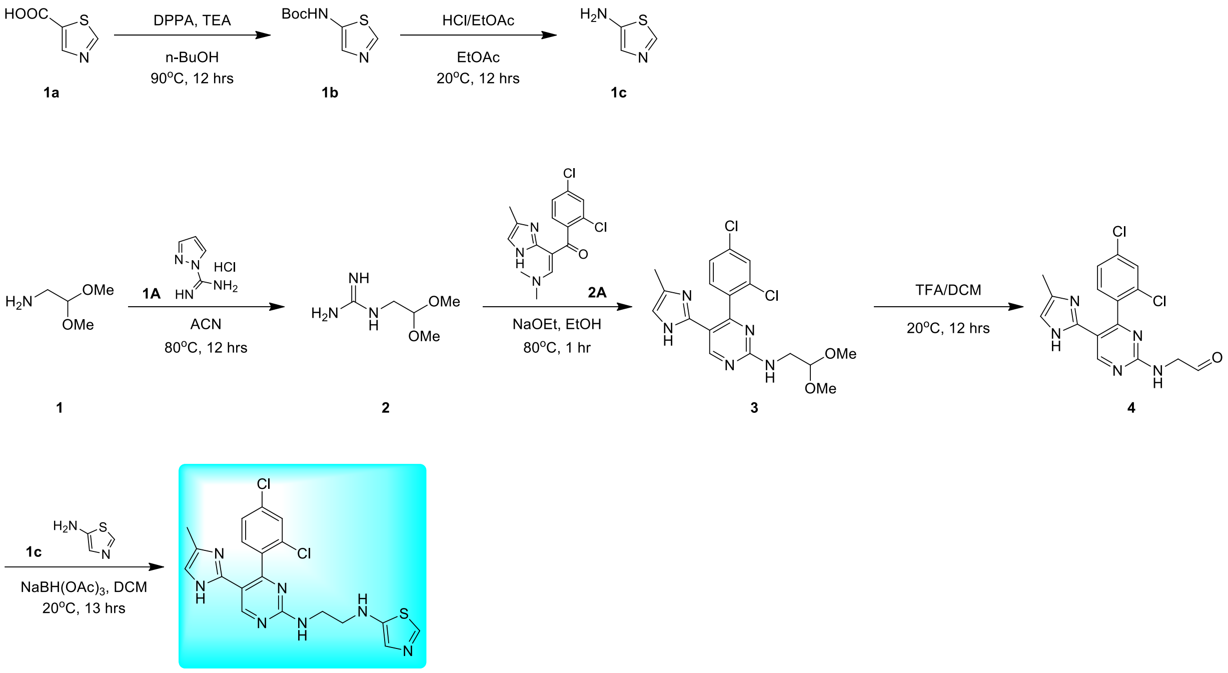

Analog 27

N^1-(4-(2,4-dichlorophenyl)-5-(4-methyl-1*H*-imidazol-2-yl)pyrimidin-2-yl)-N^2-(thiazol-5-yl)ethane-1,2-diamine (Analog 27). To a solution of **Compound 1a** (4 g, 30.97 mmol) in t-BuOH (50 mL) was added DPPA (17.05 g, 61.95 mmol, 13.42 mL) and TEA (6.27 g, 61.95 mmol, 8.62 mL). The mixture was stirred at 100°C for 12 hrs. TLC indicated that **Compound 1a** was consumed, and one main spot was formed (Petroleum ether/Ethyl acetate=3/1, P1Rf=0.4). The reaction mixture was concentrated under reduced pressure to remove solvent. The residue was purified by flash silica gel chromatography (ISCO®; 80 g SepaFlash® Silica Flash Column, Eluent of 0~50% Ethyl acetate/Petroleum ether gradient @100 mL/min) (Petroleum ether/Ethyl acetate=3/1, P1Rf=0.4) to afford ***Compound 1b*** (4.5 g, 22.47 mmol, 72.55% yield) as a white solid. To a solution of **Compound 1b** (500 mg, 2.50 mmol) in EtOAc (5 mL) was added HCl/EtOAc (4 M, 5 mL) and the mixture was stirred at 20°C for 1 hr. LC-MS showed 21% **Compound 1b** was remained and 48% desired product was detected, so the reaction was stirred at 20°C for another 12 hrs. TLC indicated that **Compound 1b** was consumed and one main spot was formed (Petroleum ether/Ethyl acetate=1/1, P1Rf=0.0). The reaction mixture was filtered and the cake was dried under reduced pressure to afford ***Compound 1c*** (320 mg, crude, HCl salt) as a white solid. MS-ESI (m/z) calcd for $C_3H_4N_2S$ $[M+H]^+$: 101.0. Found 101.1. A mixture of **Compound 1** (200 mg, 1.90 mmol, 207.25 uL) and **Compound 1A** (278.84 mg, 1.90 mmol) in ACN (2 mL) was was stirred at 80°C for 12 hrs under N_2 atmosphere. LC-MS showed **Compound 1** was consumed completely and one main peak with desired mass was detected. The reaction mixture was concentrated under reduced pressure to remove solvent to afford ***Compound 2*** (350 mg, crude, HCl salt) as a light yellow oil. MS-ESI (m/z) calcd for $C_5H_{13}N_3O_2$ $[M+H]^+$: 148.1. Found 148.1. To a solution of **Compound 2** (350 mg, 1.91 mmol, HCl salt) in EtOH (5 mL) was added NaOEt (259.40 mg, 3.81 mmol) and the mixture was stirred at 80°C for 0.5 hr, then **Compound 2A** (617.92 mg, 1.91 mmol) was added, and the resulting mixture was stirred at 80°C for 1 hr. LC-MS showed **Compound 2A** was consumed completely and one main peak with desired mass was detected. The reaction mixture was concentrated under reduced pressure to remove solvent. The residue was purified by flash silica gel chromatography (ISCO®; 12 g SepaFlash® Silica Flash Column, Eluent of 0~100% Ethyl acetate/Petroleum ether gradient @ 75 mL/min) (Petroleum ether/Ethyl acetate=1/1, P1 Rf=0.3) to afford ***Compound 3*** (400 mg, 979.72 umol, 51.40% yield) as a

brown solid. MS-ESI (m/z) calcd for $C_{18}H_{19}Cl_2N_5O_2$ $[M+H]^+$: 408.1/410.1. Found 408.1/410.1. To a solution of **Compound 3** (80 mg, 195.94 umol) in DCM (2 mL) was added TFA (352.00 mg, 3.09 mmol, 228.57 uL) and H_2O (114.29 mg, 6.34 mmol, 114.29 uL) and the mixture was stirred at 20°C for 12 hrs. LC-MS showed **Compound 3** was consumed completely and one main peak with desired MS was detected. The reaction mixture was diluted with sat. $NaHCO_3$ 10 mL and was extracted with DCM (5 mL × 3). The combined organic layers were dried over Na_2SO_4, filtered and the filtrate was concentrated under reduced pressure at low temperature to remove DCM to about 3 mL. ***Compound 4*** (72 mg, crude) in 3 mL DCM was obtained as a brown liquid and the solution was used to the next step directly. MS-ESI (m/z) calcd for $C_{16}H_{13}Cl_2N_5O$ $[M+H]^+$: 362.1/364.1. Found 362.0/364.0. To a solution of **Compound 4** (71 mg, 196.02 umol) in DCM (3 mL) was added Compound **1c** (26.78 mg, 196.02 umol, HCl salt) and the mixture was neutralized with TEA (39.67 mg, 392.03 umol, 54.57 uL) and then HOAc (23.54 mg, 392.03 umol, 22.42 uL) was added to make sure the ph of the reaction mixture was about 6, the mixture was stirred at 20°C for 1 hr, then $NaBH(OAc)_3$ (124.63 mg, 588.05 umol) was added, and the resulting mixture was stirred at 20°C for 12 hrs. LC-MS showed **Compound 4** was consumed completely and one main peak with desired mass was detected. The reaction mixture was diluted with sat. $NaHCO_3$ 10 mL and was extracted with DCM (5 mL × 3). The combined organic layers were dried over Na_2SO_4, filtered and concentrated under reduced pressure to give a residue. The residue was purified together with another batch by prep-HPLC (basic condition) (column: Phenomenex Gemini-NX C18 75*30mm*3um; mobile phase: [water (0.05%NH_3H_2O+10mM NH_4HCO_3)-ACN];B%: 25%-55%, 8 min), but LCMS showed that the purity was not high enough, so the residue was purified further by Prep-HPLC (basic condition) (column: Phenomenex Gemini-NX C18 75*30mm*3um;mobile phase: [water (0.05%NH_3H_2O+10mM NH_4HCO_3)-ACN]; B%: 20%-50%, 8 min) to afford ***analog 27*** (4.8 mg, 92.34% purity) as a white solid. **^{1}H NMR** (METHANOL-d_4 400MHz) δ ppm 8.52 (br s, 1 H) 8.05 (s, 1 H) 7.35 - 7.44 (m, 3 H) 6.92 (br s, 1 H) 6.64 (s, 1 H) 3.68 (t, J=6.06 Hz, 2 H) 3.37 (t, J=6.28 Hz, 2 H) 2.14 (s, 3 H). **LCMS** (ESI+): m/z 445.9/448.0 (M+H).

Analog 28

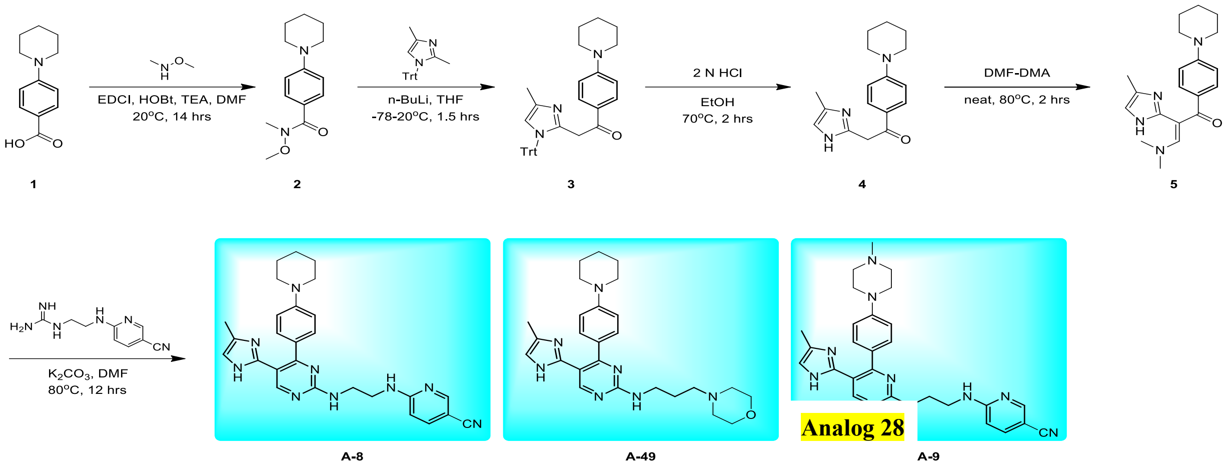

4-(2,4-dichlorophenyl)-5-(4-methyl-1*H*-imidazol-2-yl)-*N*-(3-(thiazol-2-yl)propyl)-pyrimidin-2-amine (Analog 28). To a solution of **Compound 1** (5 g, 33.78 mmol) in DMF (100 mL) was added **Compound 1A** (6.26 g, 33.78 mmol). The mixture was stirred at 100°C for 12 hrs. LCMS showed **Compound 1** was consumed completely and one main peak with desired mass was detected. The reaction mixture was cooled to 20°C and diluted with H_2O (500 ml) and extracted with ethyl acetate (200 ml × 5), the combined organic layer was washed with saturated brine (200 ml × 2), dried over Na_2SO_4, filtered and the filtrate was concentrated to give a residue. The residue was purified by flash silica gel chromatography (ISCO®; 40 g SepaFlash® Silica Flash Column, Eluent of 0~30% Ethyl acetate/Petroleum ether gradient @ 100 mL/min) (petroleum ether: ethyl acetate=2:1, Rf = 0.43) to afford ***Compound 2*** (4.3 g, 20.07 mmol, 59.42% yield) as a white solid. MS-ESI (m/z) calcd for $C_{12}H_{10}N_2O_2$ $[M+H]^+$: 215.1. Found 215.2. A mixture of **Compound 2** (3.27 g, 15.26 mmol) and **Compound 2A** (2.84 g, 15.26 mmol) in HCl/EtOAc (60 mL, 4 M) was stirred at 20°C for 12 hrs. LCMS showed **Compound 2** was consumed completely and 34% of desired compound was detected. The reaction mixture was filtered, the filter cake was washed with ethyl acetate, then the filtered cake was dried under vacuum to afford ***Compound 3*** (4.1 g, crude, HCl salt) as a white solid. MS-ESI (m/z) calcd for $C_{12}H_{12}N_2O_2S$ $[M+H]^+$: 249.1. Found 249.0. To a solution of **Compound 3** (4.1 g, 14.40 mmol, HCl salt) in acetone (80 mL) was added **Compound 3A** (3.12 g, 15.84 mmol, 2.38 mL) at 20°C. The mixture was stirred at 70°C for 12 hrs. LCMS showed **Compound 3** was consumed completely and 30% of desired compound was detected. The reaction mixture was filtered, the filter cake was washed with acetone, then the filter cake was dried under vacuum to afford ***Compound 4*** (2.71 g, crude) as a white solid. MS-ESI (m/z) calcd for $C_{14}H_{12}N_2O_2S$ $[M+H]^+$: 273.1. Found 273.0. To a solution of **Compound 4** (1 g, 3.67 mmol) in EtOH (20 mL) was added $NH_2NH_2.H_2O$ (750.32 mg, 14.69 mmol, 728.46 uL) (98% purity), the mixture was stirred at 80°C for 12 hrs. LCMS showed **Compound 4** was consumed completely and one peak with desired mass was detected. The reaction mixture was cooled to 20°C, filtered and the filtrate was concentrated to afford ***Compound 5*** (500 mg, crude) as a yellow solid. MS-ESI (m/z) calcd for $C_6H_{10}N_2S$ $[M+H]^+$: 143.1. Found 143.0. To a solution of **Compound 5** (500 mg, 3.52 mmol) and **Compound 5A** (515.32 mg, 3.52 mmol) in DMF (20 mL) was added DIEA (1.36 g, 10.55 mmol, 1.84 mL), the mixture was stirred at 80°C for 12 hrs. LCMS showed the **Compound 5** was consumed and desired mass was detected. The reaction was concentrated under vacuum to afford ***Compound 6*** (640 mg, crude) as a yellow gum and it was used directly. MS-ESI (m/z) calcd for $C_7H_{12}N_4S$ $[M+H]^+$: 185.1. Found 185.0. To a solution of **Compound 6** (60 mg, 325.62 umol) in THF (6 mL) was added Cs_2CO_3 (318.28 mg, 976.87 umol) and **Compound 6A** (105.57 mg, 325.62 umol) at 20°C. The mixture was

stirred at 80°C for 3 hrs. LCMS showed most **Compound 6** was remained, then it was stirred at 80°C for another 12 hrs. The LCMS showed the **Compound 6** was consumed completely and one main peak with desire mass was detected. The reaction mixture was filtered and the filtrate was concentrated to give a residue. The residue was purified by Prep-HPLC (neutral condition) (column: Waters Xbridge Prep OBD C18 150*40mm*10um; mobile phase: [water (10mM NH_4HCO_3)-ACN]; B%: 15%-45%, 8 min) to afford ***analog 28*** (25.5 mg, 56.88 umol, 8.73% yield, 99.34% purity) as a yellow solid. **^{1}H NMR** (METHANOL-d_4 400MHz) δ ppm 8.50 (s, 1 H) 7.66 (d, J=3.42 Hz, 1 H) 7.37 - 7.45 (m, 4 H) 6.63 (s, 1 H) 3.53 (t, J=6.79 Hz, 2 H) 3.13 (t, J=7.58 Hz, 2 H) 2.08 - 2.17 (m, 5 H). **LCMS** (ESI+): m/z 445.1/447.1 (M+H)

Analog 29

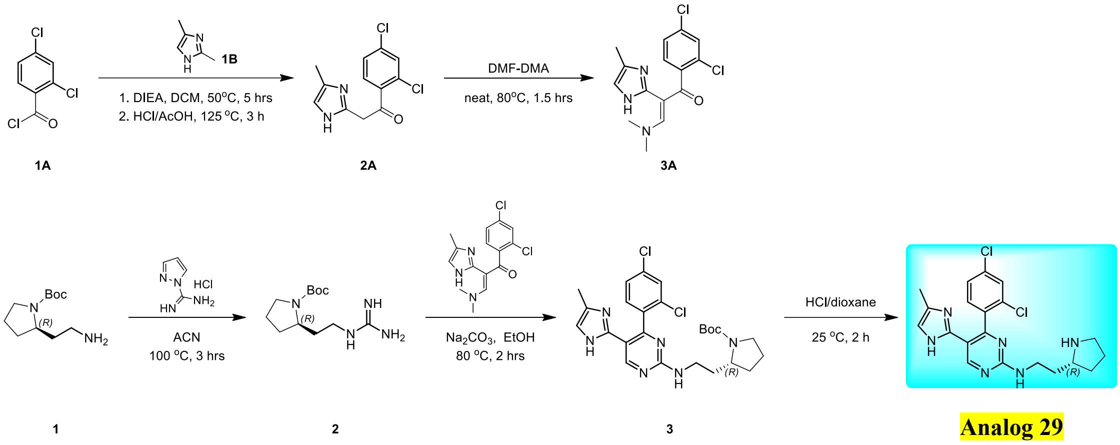

Analog 29

(*R*)-4-(2,4-dichlorophenyl)-5-(4-methyl-1*H*-imidazol-2-yl)-*N*-(2-(pyrrolidin-2-yl)ethyl)pyrimidin-2-amine (Analog 29). To a mixture of **compound 1B** (2.8 g, 29.13 mmol, 1 *eq*) in DCM (40 mL) was added DIEA (12.04 g, 93.18 mmol, 16.23 mL, 3.20 *eq*) and **compound 1A** (18.30 g, 87.39 mmol, 12.20 mL, 3 *eq*) was added below 35 °C, the mixture was stirred at 50 °C for 5 h. Then the mixture was concentrated to remove DCM, to the residue was added AcOH (40 mL) HCl (20 mL) at 25 °C, the mixture was stirred at 125 °C for 3 h. LCMS showed the mixture was completed. The mixture was concentrated to give residue, to the residue was added toluene (20 mL) and H2O (10 mL) and stirred at 25 °C for 0.5 h, filtered, the filtrate was extracted with toluene (50 mL*2), to aqueous phase was added sat.NaHCO3 till pH=8, extracted with DCM (100 mL*2). The combined organic phase was washed with brine (100 mL), dried with anhydrous Na_2SO_4, filtered and concentrated in vacuum. The residue was washed by MTBE (10 mL), filtered the filter cake was concentrated to give **compound 2A** (3.3 g, 12.26 mmol, 42.09% yield) as a yellow solid. **^{1}H NMR (400 MHz, DMSO-d_6)** δ ppm 7.53 - 7.65 (m, 2 H) 7.43 (br d, J=7.94 Hz, 1 H) 6.72 (s, 1 H) 5.62 - 5.72 (m, 1 H) 2.17 (s, 3 H). MS-ESI (m/z) calcd forC12H10Cl2N2O $[M+H]^+$: 269.0/271.0. A mixture of **compound 2A** (1 g, 3.72 mmol, 1 *eq*) in DMFDMA (8 mL) was stirred at 80 °C for 1.5 h. LCMS showed the mixture was completed. The

reaction was concentrated to give residue. The residue was used next step directly. **Compound 3A** (1.3 g, crude) as black oil. MS-ESI (m/z) calcd for C15H15Cl2N3O $[M+H]^+$: 324.0/326.0 A mixture of **compound 1** (200.00 mg, 933.25 umol, 1 *eq*) pyrazole-1-carboxamidine (164.15 mg, 1.12 mmol, 1.2 *eq*, HCl) in MeCN (5 mL) was stirred at 100 °C for 3 h. LCMS showed the mixture was completed. The reaction was concentrated to give residue. The product was used next step directly. **Compound 2** (0.2 g, crude) as colour less oil. MS-ESI (m/z) calcd for C12H24N4O2 $[M+H]^+$: 257.2. To a mixture of **compound 2** (0.15 g, 585.15 umol, 1 *eq*) (E)-1-(2,4-dichlorophenyl)-3-(dimethylamino)-2-(4-methyl-1H-imidazol-2-yl)prop-2-en-1-one (379.42 mg, 1.17 mmol, 2 *eq*) in EtOH (5 mL) was added Na2CO3 (186.06 mg, 1.76 mmol, 3 *eq*) at 25 °C, the mixture was stirred at 80 °C for 2 h. LCMS showed desired peak with desired MS. The mixture was concentrated to remove EtOH. To the residue was added H2O (20 mL) and aqueous phase was extracted with DCM (20 mL*2). The combined organic phase was washed with brine (20 mL*2), dried with anhydrous Na_2SO_4, filtered and concentrated in vacuum. The residue was purified by prep-TLC (petroleum ether: ethyl acetate=0:1, R_f=0.26) to give **compound 3** (0.15 g, 289.88 umol, 49.54% yield) as a yellow solid. MS-ESI (m/z) calcd for C25H30Cl2N6O2 $[M+H]^+$: 517.2/519.2. A mixture of **compound 3** (0.12 g, 231.91 umol, 1 *eq*) in HCl/dioxane (4 M, 20.00 mL, 344.97 *eq*) was stirred at 25 °C for 2 h. LCMS showed the mixture was completed. The mixture was concentrated to give residue. The residue was purified by prep-HPLC: column: Welch Xtimate C18 100*25mm*3um;mobile phase: [water(0.05%HCl)-ACN];B%: 1%-15%,8min to give **29** (61.05 mg, 146.29 umol, 63.08% yield, 100% purity) as a yellow solid. **^{1}H NMR** (METHANOL-d_4 400MHz) δ ppm 8.55 (br s, 1 H) 7.43 - 7.60 (m, 3 H) 7.02 (d, J=1.10 Hz, 1 H) 3.49 - 3.68 (m, 3 H) 3.24 (br s, 2 H) 2.19 (d, J=0.98 Hz, 3 H) 2.15 - 2.27 (m, 1 H) 1.81 - 2.13 (m, 4 H) 1.68 (br d, J=10.15 Hz, 1 H). **LCMS** (ESI+): *m/z* 417.2/419.2 (M+H)

Analog 30

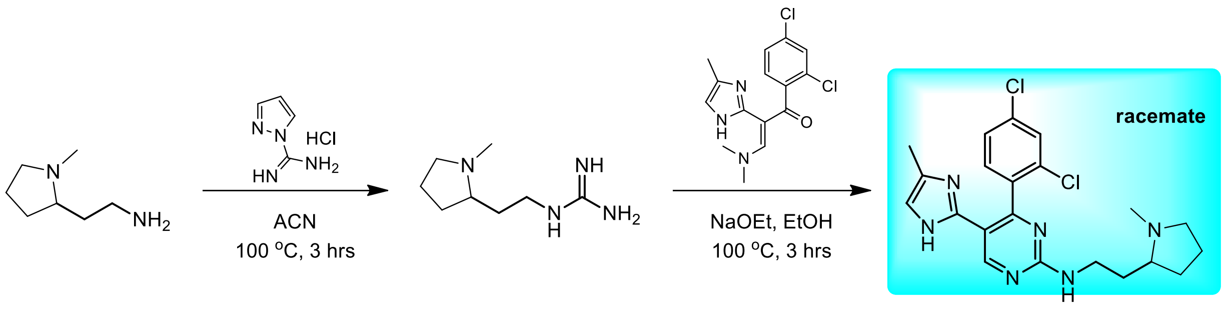

1 2 **Analog 30**

4-(2,4-dichlorophenyl)-5-(4-methyl-1*H*-imidazol-2-yl)-*N*-(2-(1-methylpyrrolidin-2-yl)ethyl)pyrimidin-2-amine (Analog 30). pyrazole-1-carboxamidine (228.64 mg, 1.56 mmol, 2 *eq*, HCl), 2-(1-methylpyrrolidin-2-yl)ethanamine (0.1 g, 779.94 umol, 112.99 uL, 1 *eq*) were taken up into a microwave tube in MeCN (5 mL). The sealed tube was heated at 100 °C for 3 h under microwave. LCMS showed the mixture was completed. The mixture was concentrated to give residue. The residue was used next step directly. **Compound 2** (0.15 g, crude) as a white solid. MS-ESI (m/z) calcd for C8H18N4 $[M+H]^+$: 171.2 **Compound 2** (0.08 g, 469.88 umol, 1 *eq*), (E)-1-(2,4-dichlorophenyl)-3-(dimethylamino)-2-(4-methyl-1H-imidazol-2-yl)prop-2-en-1-one (152.34 mg, 469.88 umol, 1 *eq*) and NaOEt (95.93 mg, 1.41 mmol, 3 *eq*) were taken up into a microwave tube in EtOH (2 mL). The sealed tube was heated at 100 °C for 3 h under microwave. LCMS showed the mixture was completed. The mixture was concentrated to give residue, to the residue was added H2O (10 mL) and DCM (10 mL), aqueous phase was extracted with DCM (20 mL*2). The combined organic phase was washed with brine (20 mL), dried with anhydrous Na_2SO_4, filtered and concentrated in vacuum. The residue was purified by prep-HPLC:column: Waters Xbridge BEH C18 100*30mm*10um;mobile phase: [water(10mM NH4HCO3)-ACN];B%: 22%-52%,10min to give **30** (14.2 mg, 31.21 umol, 6.64% yield, 94.8% purity) as yellow solid. ^{1}**H NMR** (METHANOL-d_4 400MHz) δ ppm 8.50 (br s, 1 H) 7.39 - 7.43 (m, 3 H) 6.63 (br s, 1 H) 3.44 - 3.51 (m, 2 H) 3.01 - 3.09 (m, 1 H) 2.33 (s, 3 H) 2.19 - 2.25 (m, 2 H) 2.14 (s, 3 H) 2.04 - 2.12 (m, 2 H) 1.76 (br s, 2 H) 1.50 - 1.61 (m, 2 H). **LCMS** (ESI+): *m/z* 431.0/433.1 (M+H)

Analog 31

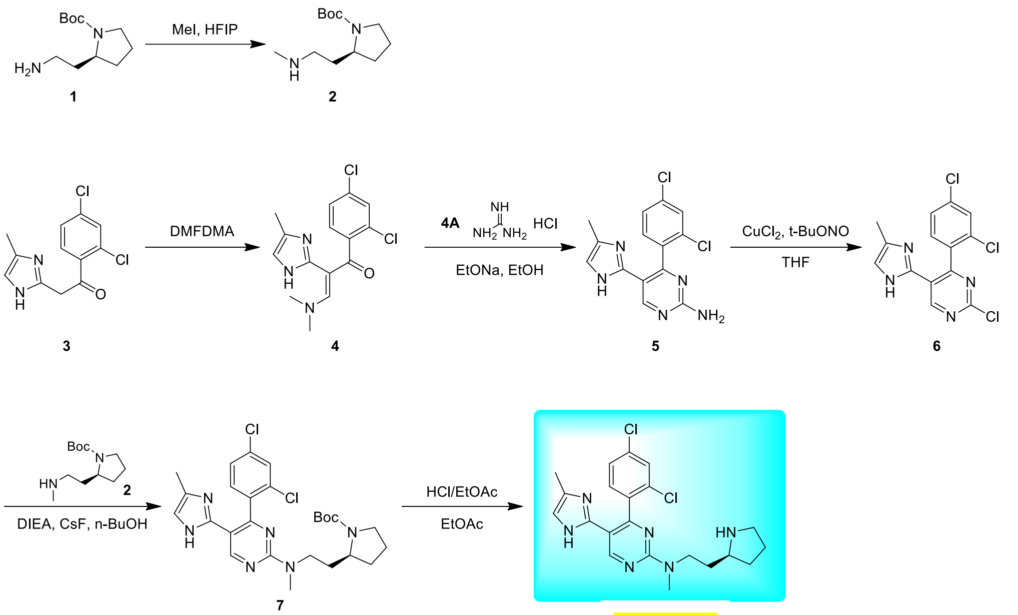

Analog 31

(*S*)-4-(2,4-dichlorophenyl)-*N*-methyl-5-(4-methyl-1*H*-imidazol-2-yl)-*N*-(2-(pyrrolidin-2-yl)ethyl)pyrimidin-2-amine (Analog 31). To a stirred solution of **Compound 1** (0.4 g, 1.87 mmol) in HFIP (0.8 mL) was added MeI (397.39 mg, 2.80 mmol, 174.30 uL). The mixture was stirred at 60°C for 12 hrs. LCMS showed **Compound 1** was consumed completely and desired mass was detected. The residue was poured into H_2O 1 mL, then extracted with EtOAc (1 mL * 3). The combined organic layers were dried over Na_2SO_4, filtered and concentrated under reduced pressure to give a residue. The residue was purified by prep-HPLC (column: Phenomenex Luna C18 100*30mm*5um; mobile phase: [water (10mM NH_4HCO_3) - ACN]; B%: 5%-35%, 10 min) to afford ***Compound 2*** (50 mg, 218.98 umol, 11.73% yield) as a Brown oil. MS-ESI (m/z) calcd for $C_{12}H_{24}N_2O_2$ $[M+H]^+$: 229.2 Found 229.3. The mixture of **Compound 3** (1.5 g, 5.57 mmol) in DMFDMA (20 mL) stirred at 80°C for 5 hrs. LCMS showed **Compound 3** was consumed completely and desired mass was detected. The combined organic phase was washed with brine (20 mL * 2), dried with anhydrous Na_2SO_4, filtered and concentrated in vacuum to afford ***Compound 4*** (1.7 g, crude) as a brown oil. MS-ESI (m/z) calcd for $C_{15}H_{15}Cl_2N_3O$ $[M+H]^+$: 324.1/326.1 Found 324.1/326.0. To a solution of **Compound 4A** (865.14 mg, 6.55 mmol, 727.01 uL, HCl) in EtOH (30 mL) was added EtONa (1.07 g, 15.73 mmol) and **Compound 4** (1.7 g, 5.24 mmol) at 0°C. Then the reaction mixture was stirred at 70°C for 12 hrs. LCMS showed **Compound 4** was consumed completely and desired mass was detected. The residue was diluted by H_2O 70 mL, then extracted with EtOAc (30 mL * 3). The combined organic layers were dried over Na_2SO_4, filtered and concentrated under reduced pressure to give a residue to afford ***Compound 5*** (1.6 g, crude) as a brown solid. MS-ESI (m/z) calcd for $C_{14}H_{11}Cl_2N_5$ $[M+H]^+$: 320.0/322.0 Found 320.2/322.2. To a solution of $CuCl_2$ (1.34 g, 9.99 mmol) and t-BuONO (1.03 g, 9.99 mmol, 1.19 mL) in THF (20 mL) was added **Compound 5** (1.6 g, 5.00 mmol) at 65°C, then the mixture was stirred at 65°C for 2.5 hrs. LCMS showed **Compound 5** was consumed completely and desired mass was detected. The reaction mixture was quenched by pouring into water 20 mL and extracted with EtOAc (10 mL * 3), dried over Na_2SO_4, filtered and concentrated under reduced pressure to give a residue. The residue was purified by flash silica gel chromatography (ISCO®; 40 g SepaFlash® Silica Flash Column, Eluent of 0~13% Ethyl acetate/Petroleum ether gradient @100 mL/min) to afford ***Compound 6*** (0.2 g, 588.92 umol, 11.78%

yield) as a yellow solid. MS-ESI (m/z) calcd for $C_{14}H_9Cl_3N_4$ $[M+H]^+$: 339.1/341.1 Found 339.1/341.0. To a solution of **Compound 2** (13.45 mg, 58.89 umol) in t-BuOH (0.3 mL) were added DIEA (7.61 mg, 58.89 umol, 10.26 uL), CsF (4.47 mg, 29.45 umol, 1.09 uL) and **Compound 6** (10 mg, 29.45 umol). The reaction vessel was sealed and heated in microwave at 140°C for 2 hrs. LCMS showed **Compound 6** was consumed completely and desired mass was detected. The reaction mixture was quenched by pouring into water 2 mL and extracted with EtOAc (2 mL * 3), dried over Na_2SO_4, filtered and concentrated under reduced pressure to afford ***Compound 7*** (20 mg, crude) as a brown solid. MS-ESI (m/z) calcd for $C_{26}H_{32}Cl_2N_6O_2$ $[M+H]^+$: 531.2/533.2 Found 531.1/533.1. To a stirred solution of **Compound 7** (20 mg, 37.63 umol) inEtOAc (1 mL) was added HCl/EtOAc (4 M, 112.89 uL). The resulting mixture was stirred at 20°C for 1 hr. LCMS showed **Compound 7** was consumed completely and desired mass was detected. The mixture was concentrated to give residue. The residue was purified by prep-HPLC (column: Phenomenex Luna C18 200*40mm*10um; mobile phase: [water (0.2%FA) - ACN]; B%: 1%-30%, 8 min) to afford ***31*** (3 mg, 6.95 umol, 18.48% yield, 100% purity) as a pale yellow solid. **^{1}H NMR** (METHANOL-d_4 400MHz) δ ppm 8.61 (s, 1H), 7.46 (s, 1H), 7.42 (s, 2H), 6.71 (s, 1H), 3.98 - 3.70 (m, 2H), 3.57 - 3.43 (m, 1H), 3.26 (s, 4H), 2.24 (br s, 1H), 2.16 (s, 3H), 2.08 (br d, J=6.5 Hz, 2H), 2.03 - 1.61 (m, 2H). **LCMS** (ESI+): *m/z* 431.0/433.1 (M+H)

Analog 32, 33

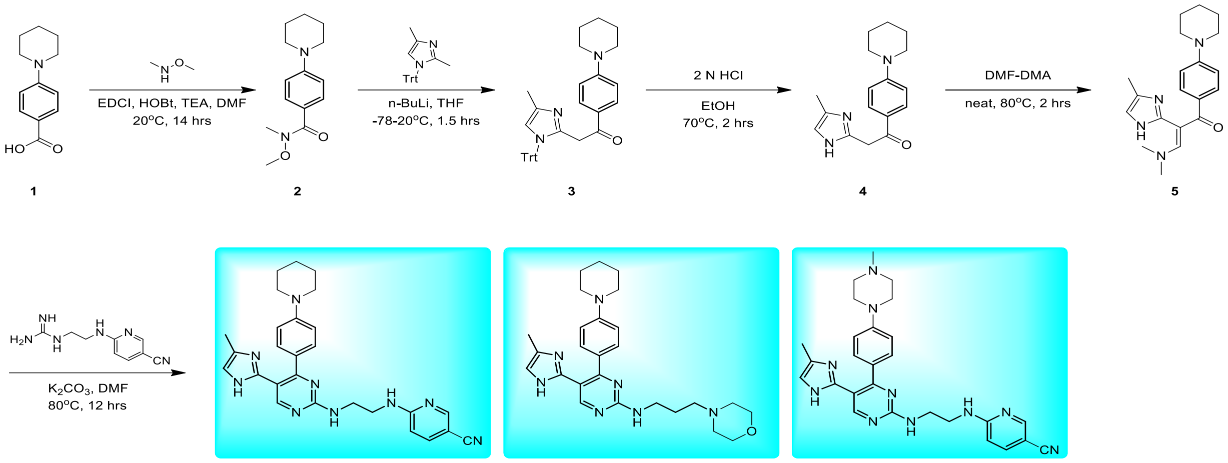

Analog 32 **Analog 33**

6-((2-((5-(4-methyl-1*H*-imidazol-2-yl)-4-(4-(pyrrolidin-1-yl)phenyl)pyrimidin-2-yl)amino)ethyl)amino)nicotinonitrile (Analog 32). To a solution of **Compound 1** (1 g, 5.23 mmol) and N-methoxymethanamine (612.12 mg, 6.28 mmol, HCl salt) in DMF (10 mL) was added EDCI (1.50 g, 7.84 mmol) and HOBt (1.06 g, 7.84 mmol) and TEA (1.59 g, 15.69 mmol, 2.18 mL). The mixture was stirred at 20°C for 12 hrs. LC-MS showed **Compound 1** was consumed completely and one main peak with desired mass was detected. The reaction mixture was diluted with H_2O 20 mL and extracted with ethyl acetate (10 mL × 3). The combined organic layers were dried over Na_2SO_4, filtered and concentrated under reduced pressure to give a residue. The residue was purified by flash silica gel chromatography (ISCO®; 12 g SepaFlash® Silica Flash Column, Eluent of 0~25% Ethyl acetate/Petroleum ether gradient @ 75 mL/min) (Petroleum ether/Ethyl acetate=3/1, P1Rf=0.2) to afford **Compound 2** (1.2 g, 5.12 mmol, 97.94% yield) as a white solid. MS-ESI (m/z) calcd for $C_{13}H_{18}N_2O_2$ $[M+H]^+$: 235.1. Found 235.1. To a solution of n-BuLi (2.5 M, 2.56 mL) in THF(5 ml) was added 2,4-dimethyl-1-trityl-imidazole (722.27 mg, 2.13 mmol) in THF(5 ml) at -78°C drop wised, the mixture was stirred at -78°C for 1 hr, then **Compound 2** (500 mg, 2.13 mmol) in THF(1 ml) was added at -78°C. The mixture was warmed up to 20°C and stirred at 20°C for 1 hr. TLC indicated that **Compound 2** was consumed, and one main spot was formed (Petroleum ether/Ethyl acetate=1/1, P1Rf=0.3). The reaction mixture was quenched by adding the solution to sat. NH_4Cl 20 ml, then the mixture was diluted with 10 ml EtOAc, then solid was formed, and the mixture was filtered and the filtered cake was dried under reduced pressure to afford **Compound 3** (600 mg, 85% purity) as a light green solid. MS-ESI (m/z) calcd for $C_{35}H_{33}N_3O$ $[M+H]^+$: 512.3. Found 512.2. To a solution of **Compound 3** (500 mg, 977.22 umol) in EtOH (5 mL) was added HCl (2 M, 4.00 mL) and the mixture was stirred at 70°C for 1 hr. LC-MS showed **Compound 3** was consumed completely and 57% desired product was detected. The reaction mixture was concentrated under reduced pressure to remove solvent. The residue was diluted with H_2O 10 mL and extracted with ethyl acetate (3 mL × 3). The organic phase was discarded and the water phase was treated with sat.$NaHCO_3$ to adjust the ph to about 9, then the mixture was extracted with ethyl acetate (3 mL × 3), the combined organic layers were dried over Na_2SO_4, filtered and concentrated under reduced pressure to afford **Compound 4** (200 mg, crude) as a yellow solid. MS-ESI (m/z) calcd for $C_{16}H_{19}N_3O$ $[M+H]^+$: 270.2. Found 270.1. A mixture of **Compound 4** (50 mg, 185.64 umol) in DMFDMA (1 mL) was stirred at 80°C for 1.5 hrs under N_2 atmosphere. LC-MS showed **Compound 4** was consumed completely and 35% desired product was detected. The reaction mixture was concentrated under reduced pressure to remove solvent to afford **Compound 5** (60 mg, crude) as a brown oil. MS-ESI (m/z) calcd for $C_{19}H_{24}N_4O$ $[M+H]^+$: 325.2. Found 325.1. To a solution of **Compound**

5A (37.77 mg, 184.95 umol) in EtOH (1 mL) was added NaOEt (25.17 mg, 369.89 umol) and the resulting mixture was stirred at 80°C for 0.5 hr, then **Compound 5** (30 mg, 92.47 umol) was added, and the resulting mixture was stirred at 80°C for 1 hr. LC-MS showed **Compound 5** was consumed completely and one main peak with desired mass was detected. The reaction mixture was concentrated under reduced pressure to remove solvent. The residue was purified by prep-HPLC (basic condition) (column: Waters Xbridge Prep OBD C18 150*40mm*10um; mobile phase: [water (0.05%NH_3H_2O+10mM NH_4HCO_3)-ACN]; B%: 20%-50%, 8min) to afford ***32*** (28.2 mg, 60.57 umol, 32.75% yield, 100% purity) as a light yellow solid. **^{1}H NMR** (METHANOL-*d*$_4$ 400MHz) *δ* ppm 8.14 - 8.36 (m, 2 H) 7.46 (br s, 1 H) 7.23 (br d, J=8.82 Hz, 2 H) 6.76 (s, 1 H) 6.45 (br d, J=8.60 Hz, 3 H) 3.73 (br s, 2 H) 3.63 (br s, 2 H) 3.28 - 3.31 (m, 4 H) 2.21 (s, 3 H) 2.02 (br t, J=6.39 Hz, 4 H). **LCMS** (ESI+): *m*/*z* 466.2 (M+H).

The procedure of preparation of **Analog 33** is similar with that of **32**. **^{1}H NMR** (METHANOL-*d*$_4$ 400MHz) *δ* ppm 8.15 - 8.37 (m, 2 H) 7.49 (br s, 1 H) 7.28 (br d, J=8.60 Hz, 2 H) 6.85 (br d, J=8.60 Hz, 2 H) 6.73 (s, 1 H) 6.48 (br s, 1 H) 3.78 - 3.84 (m, 4 H) 3.72 (br s, 2 H) 3.64 (br d, J=5.07 Hz, 2 H) 3.16 - 3.24 (m, 4 H) 2.13 - 2.23 (m, 3 H). **LCMS** (ESI+): *m*/*z* 482.3 (M+H)

Analog 34

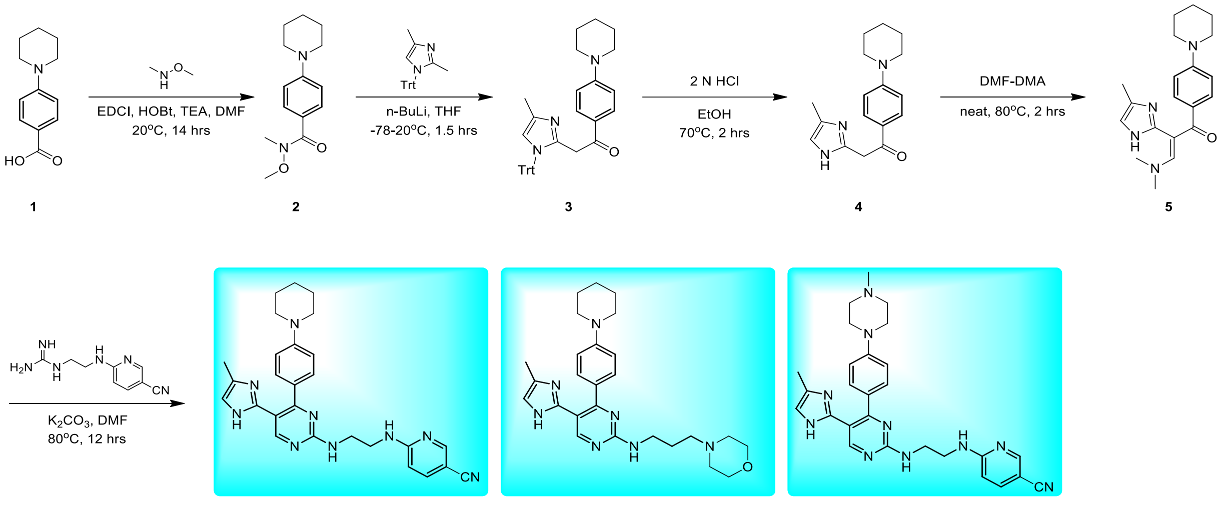

Analog 34

6-((2-((5-(4-methyl-1*H*-imidazol-2-yl)-4-(4-(tetrahydro-2*H*-pyran-4-yl)phenyl)pyrimidin-2-yl)amino)ethyl)amino)nicotinonitrile (Analog 34). To a solution of **Compound 1** (3 g, 13.95 mmol) and **Compound 1A** (2.99 g, 14.23 mmol) in dioxane (30 mL) and H_2O (3 mL) was added Pd(dppf)Cl_2 (1.02 g, 1.40 mmol) and K_2CO_3 (3.86 g, 27.90 mmol). The mixture was stirred at 90°C for 12 hrs under N_2. LC-MS showed **Compound 1** was consumed completely and one main peak with desired mass was detected. The reaction mixture was concentrated under reduced pressure to remove solvent. The residue was purified by flash silica gel chromatography (ISCO®; 40 g SepaFlash® Silica Flash Column, Eluent of 0~10% Ethyl acetate/Petroleum ether gradient @ 100 mL/min) (Petroleum ether/Ethyl acetate=10/1, P1Rf=0.3) to afford ***Compound 2*** (1.6 g, 7.33 mmol, 52.55% yield) as an off-white solid. MS-ESI (m/z) calcd for $C_{13}H_{14}O_3$ $[M+H]^+$: 219.1. Found 219.0. To a solution of **Compound 2** (1 g, 4.58 mmol) in EtOAc (10 mL) was added Pd/C (500 mg, 10% purity). The mixture was stirred at 20°C for 2 hrs under H_2 atmosphere at 15 PSI. LC-MS showed **Compound 2** was consumed completely and one main peak with desired mass was detected. The reaction was worked up together with another batch and the reaction mixture was filtered and the filtrate was concentrated under reduced pressure to afford

Compound 3 (1.8 g, crude) as a light yellow solid. MS-ESI (m/z) calcd for $C_{13}H_{16}O_3$ $[M+H]^+$: 221.1. Found 221.2. To a solution of **Compound 3** (648 mg, 2.94 mmol) in THF (10 mL), MeOH (2 mL) and H_2O (2 mL) was added LiOH.H_2O (617.27 mg, 14.71 mmol) and the mixture was stirred at 20 °C for 12 hrs. LC-MS showed **Compound 3** was consumed and 97% of desired compound was detected. The reaction mixture was diluted with H_2O (10 mL) and extracted with EtOAc (3 mL × 1), the organic phase was discarded, then the water phase was acidified with 1 N HCl to pH=3 and filtered, the filtered cake was dried under reduced pressure to afford ***Compound 4*** (517 mg, crude) as a white solid. MS-ESI (m/z) calcd for $C_{12}H_{14}O_3$ $[M+H]^+$: 207.1. Found 207.2. To a solution of **Compound 4** (500 mg, 2.42 mmol) in DMF (7 mL) was added EDCI (697.14 mg, 3.64 mmol), HOBt (491.39 mg, 3.64 mmol) and Et_3N (735.97 mg, 7.27 mmol, 1.01 mL), then N-methoxymethanamine (177.71 mg, 2.91 mmol) was added to the mixture. The mixture was stirred at 20°C for 12 hrs. TLC (Petroleum ether/Ethyl acetate=1/1, P1Rf=0.3) indicated **Compound 4** was consumed completely and new spot formed. The reaction was worked up together with another batch and the reaction mixture was concentrated under reduced pressure to give a residue. The residue was purified by flash silica gel chromatography (ISCO®; 12 g SepaFlash® Silica Flash Column, Eluent of 0~11% Ethyl acetate/Petroleum ether gradient @ 100 mL/min) (Petroleum ether/Ethyl acetate=1/1, P1 Rf=0.3) to afford ***Compound 5*** (700 mg, 97% purity) as a white solid. MS-ESI (m/z) calcd for $C_{14}H_{19}NO_3$ $[M+H]^+$: 250.1. Found 250.2. To a solution of n-BuLi (2.5 M, 2.89 mL) in THF (20 mL) was added 2,4-dimethyl-1-trityl-imidazole (814.53 mg, 2.41 mmol) in THF (22 mL) drop wised at -78 °C under N_2 atmosphere. The mixture was stirred at -78 °C for 1 hr under N_2 atmosphere. Then **Compound 5** (600 mg, 2.41 mmol) in THF (8 mL) was added to the mixture at -78 °C under N_2 atmosphere, the mixture was allowed to warm up to 20°C and stirred at 20 °C for 1 hr under N_2 atmosphere. LC-MS showed **Compound 5** was consumed completely and 56% of desired compound was detected. The reaction mixture was quenched by addition to saturated NH_4Cl solution 20 mL at 15°C, and then diluted with H_2O 10 mL and extracted with EtOAc (20 mL × 3), the combined organic layers were dried over Na_2SO_4, then filtered and concentrated under reduced pressure to afford ***Compound 6*** (820 mg, crude) as a yellow oil. MS-ESI (m/z) calcd for $C_{36}H_{34}N_2O_2$ $[M+H]^+$: 527.3. Found 527.2. To a solution of **Compound 6** (400 mg, 759.49 umol) in EtOH (10 mL) was added HCl (1 M, 10 mL) and the mixture was stirred at 70 °C for 1 hr. LC-MS showed **Compound 6** was consumed completely and 51% of desired compound was detected. The reaction mixture was concentrated under reduced pressure to remove EtOH, then the residue was diluted with H_2O (10 mL) and extracted with EtOAc (10 mL × 2), the organic phase was discarded, then the water phase was basified with saturated Na_2CO_3 solution to pH=8 and extracted with EtOAc (10 mL × 3), the combined organic layers were dried over Na_2SO_4, filtered and concentrated under reduced pressure to afford ***Compound 7*** (400 mg, 1.35 mmol, 88.81% yield, 95.9% purity) as a white solid. MS-ESI (m/z) calcd for $C_{17}H_{20}N_2O_2$ $[M+H]^+$: 285.2. Found 285.2. A solution of **Compound 7** (50 mg, 175.84 umol) in DMFDMA (1 mL) was stirred at 80 °C for 1 hr. LC-MS showed **Compound 7** was consumed completely and one main peak with desired mass was detected. The reaction mixture was concentrated under reduced pressure to remove solvent to afford ***Compound 8*** (60 mg, crude) as a brown oil. MS-ESI (m/z) calcd for $C_{20}H_{25}N_3O_2$ $[M+H]^+$: 340.2. Found 340.3. To a solution of **Compound 8A** (72.20 mg, 353.53 umol) in EtOH (3 mL) was added NaOEt (48.12 mg, 707.07 umol) and the mixture was stirred at 80°C for 0.5 hr, then **Compound 8** (60 mg, 176.77 umol) was added at 80°C, and the resulting mixture was stirred at 80°C for 1 hr. LC-MS showed **Compound 8** was consumed completely and one main peak with desired mass was detected. The reaction mixture was concentrated under reduced pressure to remove solvent. The residue was purified by prep-HPLC (basic condition) (column: Phenomenex Gemini-NX C18 75*30mm*3um; mobile phase: [water (0.05%NH_3H_2O+10mM NH_4HCO_3)-ACN]; B%: 25%-55%, 8min)

to afford ***34*** (9.3 mg, 18.85 umol, 10.66% yield, 97.40% purity) as a light yellow solid. **^{1}H NMR** (METHANOL-d_4 400MHz) δ ppm 8.20 - 8.39 (m, 2 H) 7.36 - 7.53 (m, 1 H) 7.32 (d, J=8.38 Hz, 2 H) 7.21 (d, J=8.38 Hz, 2 H) 6.72 (s, 1 H) 6.37 - 6.58 (m, 1 H) 4.00 - 4.07 (m, 2 H) 3.71 (br s, 2 H) 3.63 (br d, J=5.07 Hz, 2 H) 3.51 - 3.59 (m, 2 H) 2.74 - 2.89 (m, 1 H) 2.11 - 2.23 (m, 3 H) 1.72 - 1.81 (m, 4 H). **LCMS** (ESI+): m/z 481.2 (M+H)

Analog 35

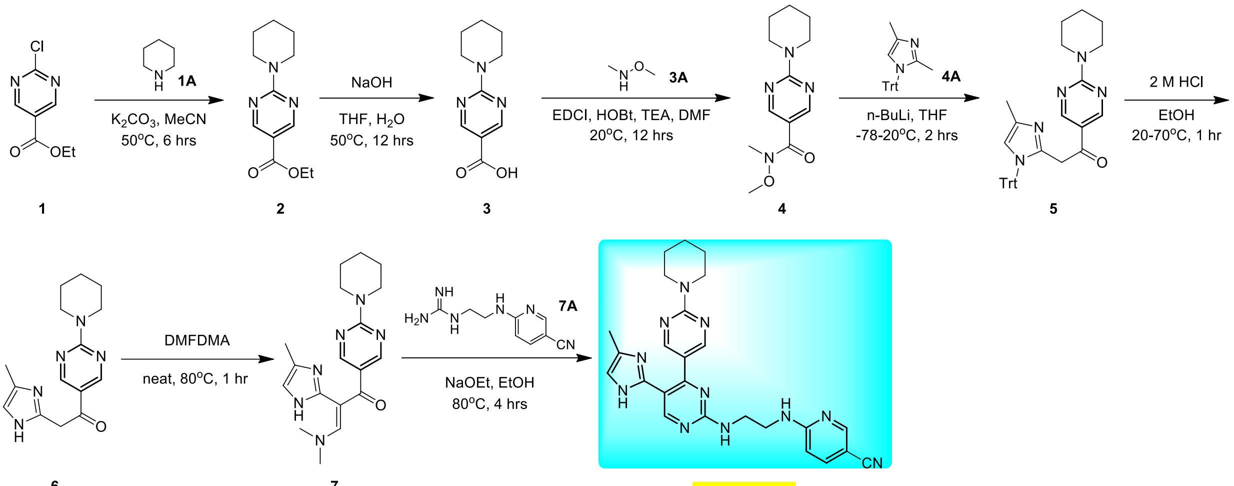

6-((2-((5-(4-methyl-1*H*-imidazol-2-yl)-2'-(piperidin-1-yl)-[4,5'-bipyrimidin]-2-yl)amino)ethyl)amino)nicotinonitrile (Analog 35). To a solution of **Compound 1** (1 g, 5.36 mmol) and **Compound 1A** (1.37 g, 16.08 mmol, 1.59 mL) in MeCN (10 mL) was added K_2CO_3 (1.48 g, 10.72 mmol) at 20°C, then the mixture was stirred at 50°C for 6 hrs. TLC (Petroleum ether: Ethyl acetate=5:1) (P1 Rf=0.38) showed **Compound 1** was consumed completely and a new spot was formed. The reaction was concentrated under vacuum. The residue was diluted with H_2O (20 mL), the aqueous layer was extracted with EtOAc (20 mL × 3), the organic layer was dried over anhydrous Na_2SO_4, filtered and the filtrate was concentrated under vacuum to afford ***Compound 2*** (1.26 g, crude) as a yellow solid and it was used directly. To a solution of **Compound 2** (1.26 g, 5.36 mmol) in THF (20 mL) was added NaOH (1 M, 20 mL) at 20°C, then the mixture was stirred at 50°C for 12 hrs. TLC (Petroleum ether: Ethyl acetate=3:1) (P1 RF=0.00) showed **Compound 2** was consumed completely and a new spot was formed. THF was removed under vacuum. The aqueous layer was acified with 1 M HCl to pH=5, solid was appeared and the mixture was filered and the filter-cake was dried under vacuum to afford ***Compound 3*** (1.14 g, crude) as a white solid. To a solution of **Compound 3** (1.14 g, 5.50 mmol) in DMF (12 mL) were added EDCI (1.58 g, 8.25 mmol), HOBt (1.12 g, 8.25 mmol), TEA (1.67 g, 16.50 mmol, 2.30 mL) and **Compound 3A** (643.93 mg, 6.60 mmol , HCl salt) at 20°C, then the mixture was stirred at 20°C for 12 hrs. LCMS showed the **Compound 3** was consumed and 17% of desired product was detected. The reaction was concentrated under vacuum. The residue was diluted with water (8 mL), the aqueous layer was extracted with EtOAc (10 mL × 3), the organic layer was dried over anhydrous Na_2SO_4, filtered and the filtrate was concentrated under vacuum. The residue was purified by flash silica gel

chromatography (ISCO®; 4 g SepaFlash® Silica Flash Column, Eluent of 0~30% Ethyl acetate/Petroleum ether gradient @ 36 mL/min) to afford ***Compound 4*** (1.11 g, 4.43 mmol, 80.61% yield) as a colourless oil. MS-ESI (m/z) calcd for $C_{12}H_{18}N_4O_2$ $[M+H]^+$: 251.1. Found 251.1. To a solution of n-BuLi (2.5 M, 479.43 uL) in THF(4mL) was added **Compound 4A** (135.22 mg, 399.53 umol) at -78°C, then the mixture was stirred at -78°C for 30 min, then another solution of **Compound 4** (100 mg, 399.53 umol) in THF(2mL) was added at -78°C, the mixture was stirred at 20°C for 1.5 hrs. LCMS showed **Compound 4** was consumed and 42% of desired product was detected. The reaction was added H_2O (5 mL) slowly, then the mixture was extracted with EtOAc (5 mL×3), the organic layer was dried over anhydrous Na_2SO_4, filtered and the filtrate was concentrated under vacuum to afford ***Compound 5*** (223 mg, crude) as a yellow liquid and it was used directly. MS-ESI (m/z) calcd for $C_{34}H_{33}N_5O$ $[M+H]^+$: 528.3. Found 528.5. To a solution of **Compound 5** (223 mg, 422.62 umol) in EtOH (3 mL) was added HCl (2 M, 3 mL) at 20°C, then the mixture was stirred at 70°C for 1 hr. LCMS showed the **Compound 5** was consumed and 23% of desired product was detected. The reaction mixture was concentrated under reduced pressure to remove solvent. The residue was diluted with H_2O 6 mL and extracted with ethyl acetate (3 mL × 3). The combined organic layers were discarded and the water phase was adjust the pH=9 with sat. $NaHCO_3$, extracted with ethyl acetate (5 mL × 3). The combined organic layers were dried over Na_2SO_4, filtered and concentrated under reduced pressure to give a residue to afford ***Compound 6*** (72 mg, 169.06 umol, 40.00% yield, 67% purity) as a purple liquid. MS-ESI (m/z) calcd for $C_{15}H_{19}N_5O$ $[M+H]^+$: 286.2. Found 286.1. A mixture of **Compound 6** (200 mg, 700.91 umol) in DMFDMA (5 mL) was stirred at 80°C for 1 hr. LCMS showed **Compound 6** was consumed and 23% of desired product was detected. The reaction was concentrated under vacuum to afford ***Compound 7*** (250 mg, crude) as a brown liquid and it was used directly. MS-ESI (m/z) calcd for $C_{18}H_{24}N_6O$ $[M+H]^+$: 341.2. Found 341.2. To a solution of **Compound 7A** (10.80 mg, 52.88 umol) and EtONa (10.79 mg, 158.63 umol) in EtOH (1 mL) was stirred at 80°C for 15 min, then another solution of **Compound 7** (18 mg, 52.88 umol) in EtOH (1 mL) was added at 80°C , then the mixture was stirred at 80°C for 4 hrs. LCMS showed 16% of **Compound 7** was remained and 52% of desired product was detected. The reaction was filtered. The filtrate was purified by Prep-HPLC (basic condition) (column: Phenomenex Gemini-NX C18 75*30mm*3um; mobile phase: [water (0.05% NH_3H_2O+10 mM NH_4HCO_3)-ACN]; B%: 25%-55%, 8 min), the fraction was combined with another batch to afford ***35*** (12.50 mg, 25.95 umol, 7.5% yield, 98.67% purity) as a pale yellow solid. **^{1}H NMR** (METHANOL-d_4 400MHz) δ ppm 8.36 (br s, 1 H) 8.22 - 8.32 (m, 3 H) 7.52 (br s, 1 H) 6.82 (br s, 1 H) 6.51 (br s, 1 H) 3.80 - 3.88 (m, 4 H) 3.71 (br s, 2 H) 3.65 (br d, *J*=5.73 Hz, 2 H) 2.24 (s, 3 H) 1.72 (br d, *J*=4.19 Hz, 2 H) 1.61 (br d, *J*=4.41 Hz, 4 H). **LCMS** (ESI+): *m/z* 482.1 (M+H)

Analog 36, 37

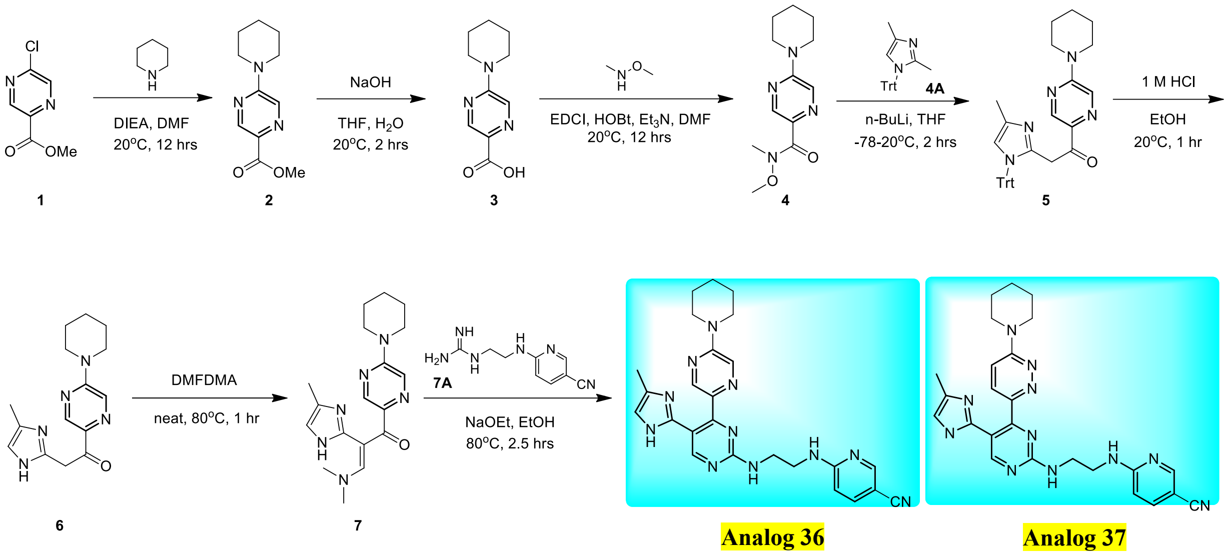

6-((2-((5-(4-methyl-1*H*-imidazol-2-yl)-4-(4-(piperidin-1-yl)phenyl)pyrimidin-2-yl)amino)ethyl)amino)nicotinonitrile (Analog 36)

6-((2-((5-(4-methyl-1*H*-imidazol-2-yl)-4-(6-(piperidin-1-yl)pyridazin-3-yl)pyrimidin-2-yl)amino)ethyl)amino)nicotinonitrile (Analog 37)

To a solution of **Compound 1** (3 g, 17.38 mmol) in DMF (15 mL) was added DIEA (3.37 g, 26.08 mmol, 4.54 mL) and piperidine (1.48 g, 17.38 mmol, 1.72 mL). The mixture was stirred at 20 °C for 12 hrs. LC-MS showed **Compound 1** was consumed completely and ~32.9% of desired compound was detected. H_2O (15 mL) was added to the reaction mixture, then the mixture was filtered and the cake was dried under vacuum to afford **Compound 2** (2.65 g, crude) as a white solid. MS-ESI (m/z) calcd for $C_{11}H_{15}N_3O_2$ $[M+H]^+$: 222.1. Found 222.1. To a solution of **Compound 2** (2.65 g, 11.98 mmol) in H_2O (24 mL) was added NaOH (1.92 g, 47.91 mmol). The mixture was stirred at 20°C for 1 hr. Then THF (9 mL) was added, the mixture was stirred at 20°C for 1 hr. TLC (Petroleum ether/Ethyl acetate=3/1, P1Rf=0.00) indicated **Compound 2** was consumed completely and one new spot formed. The reaction mixture was concentrated under reduced pressure to remove solvent. Then the mixture was adjusted to pH 4 with 1N HCl. Then the mixture was cooled to 0°C. Then the mixture was filtered and the cake was dried under vacuum to afford **Compound 3** (2.4 g, crude) was obtained as a white solid. To a solution of **Compound 3** (1.1 g, 5.31 mmol) in DMF (12 mL) was added EDCI (1.53 g, 7.96 mmol), HOBt (1.08 g, 7.96 mmol,) and Et_3N (1.61 g, 15.92 mmol, 2.22 mL). Then N-methoxymethanamine (621.33 mg, 6.37 mmol, HCl salt) was added to the mixture, the mixture was stirred at 20°C for 12 hrs. LC-MS showed **Compound 3** was consumed completely and 57% of desired compound was detected. The reaction mixture was concentrated under reduced pressure to give a residue. The residue was purified by flash silica gel chromatography (ISCO®; 4 g SepaFlash® Silica Flash Column, Eluent of 0~10% Ethyl acetate/Petroleum ether gradient @ 100 mL/min) (Petroleum ether/Ethyl acetate=1/1, P1 Rf=0.2) to afford **Compound 4** (900 mg, 3.60 mmol, 67.74% yield) as a yellow solid. MS-ESI (m/z) calcd for $C_{12}H_{18}N_4O_2$ $[M+H]^+$: 251.1. Found 251.1. To a solution of n-BuLi (2.5 M, 1.44 mL) in THF (5 mL) was added **Compound 4A** (405.65 mg, 1.20 mmol) in THF (15 mL) drop wised at -78 °C under N_2 atmosphere. The mixture was stirred at -78 °C for 1 hr under N_2 atmosphere. Then **Compound 4** (300 mg, 1.20 mmol) in THF (5 mL) was added to the mixture at -78 °C, the mixture was allowed to warm up to 20°C and

stirred at 20°C for 1 hr under N_2 atmosphere. LC-MS showed **Compound 4** was consumed completely and 23% of desired compound was detected. The reaction mixture was quenched by addition to saturated NH_4Cl solution 20 mL at 15°C, and then diluted with H_2O 10 mL and extracted with EtOAc (20 mL × 3); the combined organic layers were dried over Na_2SO_4, then filtered and concentrated under reduced pressure to give a residue. MS-ESI (m/z) calcd for $C_{34}H_{33}N_5O$ $[M+H]^+$: 528.3. Found 528.3. To a solution of **Compound 5** (700 mg, 1.33 mmol) in EtOH (7 mL) was added HCl (1 M, 7 mL) and the mixture was stirred at 70°C for 1 hr. LC-MS showed **Compound 5** was consumed and 24% of desired compound was detected. The reaction mixture was concentrated under reduced pressure to remove EtOH, then the residue was diluted with H_2O (5 mL) and extracted with EtOAc (5 mL × 2), the organic phase was discarded, then the water phase was basified with saturated Na_2CO_3 solution to pH=8 and extracted with EtOAc (5 mL × 3), the combined organic layers were dried over Na_2SO_4, filtered and concentrated under reduced pressure to give a residue. The residue was purified by prep-HPLC (basic condition) (column: Waters Xbridge Prep OBD C18 150*40mm*10um; mobile phase: [water (0.05%NH_3H_2O+10mM NH_4HCO_3)-ACN]; B%: 10%-40%, 8min) to afford **Compound 6** (30 mg, 84.11 umol, 6.34% yield, 80% purity) as a yellow solid. MS-ESI (m/z) calcd for $C_{15}H_{19}N_5O$ $[M+H]^+$: 286.2. Found 286.0. A solution of **Compound 6** (20 mg, 70.09 umol) in DMFDMA (1 mL) was stirred at 80°C for 1 hr. LC-MS showed **Compound 6** was consumed completely and one main peak with desired mass was detected. The reaction mixture was concentrated under reduced pressure to remove solvent to afford **Compound 7** (25 mg, crude) as a brown oil. MS-ESI (m/z) calcd for $C_{18}H_{24}N_6O$ $[M+H]^+$: 341.2. Found 341.1 To a solution of **Compound 7A** (22.50 mg, 110.16 umol) in EtOH (2 mL) was added NaOEt (14.99 mg, 220.31 umol) and the mixture was stirred at 80°C for 0.5 hr, then **Compound 7** (25 mg, 73.44 umol) was added at 80°C, and the resulting mixture was stirred at 80°C for 1 hr. LC-MS showed **Compound 7** was consumed completely and 23% desired product was detected. The reaction mixture was concentrated under reduced pressure to remove solvent. The residue was purified by prep-HPLC (basic condition) (column: Phenomenex Gemini-NX C18 75*30mm*3um; mobile phase: [water (0.05%NH_3H_2O+10mM NH_4HCO_3)-ACN]; B%: 20%-50%, 8min) to afford ***36*** (3.1 mg, 6.44 umol, 8.77% yield, 100% purity) as a yellow gum.

The procedure of making 37 is similar with that of 36.

^{1}H NMR (METHANOL-d_4 400MHz) δ ppm 8.52 - 8.74 (m, 1 H) 8.47 (s, 1 H) 8.34 (br s, 1 H) 8.07 (d, J=1.00 Hz, 1 H) 7.50 (br s, 1 H) 6.74 (s, 1 H) 6.51 (br s, 1 H) 3.61 - 3.77 (m, 8 H) 2.22 (d, J=0.75 Hz, 3 H) 1.62 - 1.77 (m, 6 H). **LCMS** (ESI+): *m/z* 482.3 (M+H)

Analog 38, 39

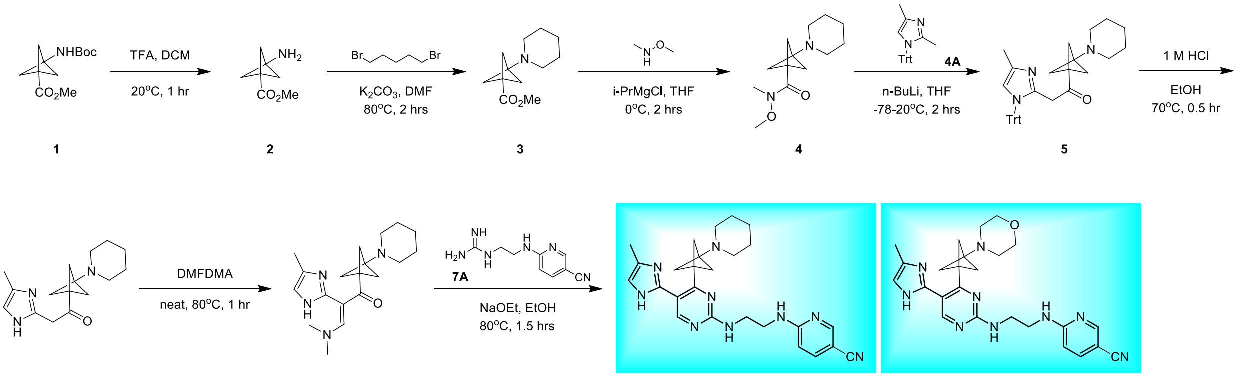

Analog 38 Analog 39

6-((2-((5-(4-methyl-1*H*-imidazol-2-yl)-4-(3-(piperidin-1-yl)bicyclo[1.1.1]pentan-1-yl)pyrimidin-2-yl)amino)ethyl)amino)nicotinonitrile (Analog 38). To a solution of **Compound 1** (450 mg, 1.87 mmol) in DCM (5 mL) was added TFA (6.93 g, 60.78 mmol, 4.50 mL) and the mixture was stirred at 20°C for 1 hr. TLC indicated that **Compound 1** was consumed, and one main spot was formed. (SiO_2, Petroleum ether/Ethyl acetate=3/1, P1Rf=0.2). The reaction mixture was concentrated under reduced pressure to afford **Compound 2** (480 mg, crude, TFA salt) as a light yellow oil. To a solution of **Compound 2** (480 mg, 1.88 mmol, TFA salt) and 1,5-dibromopentane (1.30 g, 5.64 mmol, 763.25 uL) in ACN (10 mL) was added K_2CO_3 (1.56 g, 11.29 mmol) and the mixture was stirred at 80°C for 2 hrs. TLC indicated that **Compound 2** was consumed, and one main spot was formed (Petroleum ether/Ethyl acetate=3/1, P1Rf=0.3). The reaction mixture was concentrated under reduced pressure to remove solvent. The residue was diluted with H_2O 10 mL and extracted with ethyl acetate (3 mL × 3). The combined organic layers were dried over Na_2SO_4, filtered and concentrated under reduced pressure to give a residue. The residue was purified by flash silica gel chromatography (ISCO®; 4 g SepaFlash® Silica Flash Column, Eluent of 0~20% Ethyl acetate/Petroleum ether gradient @ 50 mL/min) (Petroleum ether/Ethyl acetate=3/1, P1Rf=0.3) to afford **Compound 3** (390 mg, 1.86 mmol, 99.07% yield) as a light yellow oil. To a solution of **Compound 3** (260 mg, 1.24 mmol) and N-methoxymethanamine (181.77 mg, 1.86 mmol, HCl salt) in THF (3 mL) was added i-PrMgCl (2 M, 2.48 mL) at 0°C under N_2 atmosphere. The mixture was stirred at 0°C for 2 hrs. TLC indicated that **Compound 3** was consumed, and one main spot was formed (Petroleum ether/Ethyl acetate=0/1, P1Rf=0.1). The reaction mixture was quenched with sat. NH_4Cl 10 mL and then was extracted with ethyl acetate (3 mL × 3). The combined organic layers were dried over Na_2SO_4, filtered and concentrated under reduced pressure to give a residue. The residue was purified by flash silica gel chromatography (ISCO®; 4 g SepaFlash® Silica Flash Column, Eluent of 0~60% Ethyl acetate/Petroleum ether gradient @ 50 mL/min) (Petroleum ether/Ethyl acetate=0/1, P1Rf=0.1) to afford **Compound 4** (220 mg, 923.11 umol, 74.30% yield) as a yellow oil. To a solution of n-BuLi (2 M, 849.68 uL) in THF (2 mL) was added **Compound 4A** (191.71 mg, 566.45 umol) in THF (2 mL) at -78°C drop wised under N_2. The mixture was stirred at -78°C for 1 hr, then **Compound 4**(150 mg, 629.39 umol) in THF (1 ml) was added drop wised at -78°C. Then the mixture was allowed to warm to 20°C and stirred at 20°C for 1hr. LC-MS showed **Compound 4** was consumed completely and 36% desired product was detected. The reaction mixture was diluted with sat. NH_4Cl 10 mL and was extracted with ethyl acetate (5 mL × 3). The combined organic layers were dried over Na_2SO_4, filtered and concentrated under reduced pressure to give a residue. The residue was dilute with 5 ml EtOAc, there was solid formed in the mixture, then the solid was filtered and the cake was dried under reduced pressure to afford **Compound 5** (60 mg, 116.35 umol, 18.49% yield) as a light yellow solid. The filtrate was concentrated under reduced pressure to afford **Compound 5A** (200 mg, crude) as a brown gum.

Compound 5 was used to the next step. MS-ESI (m/z) calcd for $C_{35}H_{37}N_3O$ $[M+H]^+$: 516.3. Found 516.5. To a solution of **Compound 5** (60 mg, 116.35 umol) in EtOH (1 mL) was added HCl (1 M, 1.20 mL,) and the mixture was stirred at 70°C for 0.5 hr. LC-MS showed **Compound 5** was consumed completely and 34% desired product was detected. The reaction mixture was concentrated under reduced pressure to remove solvent. The residue was diluted with H_2O 10 mL and extracted with EtOAc (3 mL × 3), the organic phase was discarded and the water phase was treated with sat. $NaHCO_3$ to adjust the ph to about 9 then was extracted with EtOAc (3 mL × 3). The combined organic layers were dried over Na_2SO_4, filtered and concentrated under reduced pressure to afford **Compound 6** (32 mg, crude) as yellow oil. MS-ESI (m/z) calcd for $C_{16}H_{23}N_3O$ $[M+H]^+$: 274.2. Found 274.1. A mixture of Comound 6 (30 mg, 109.74 umol) in DMFDMA (1 mL) was stirred at 80°C for 1 hr under N_2 atmosphere. LC-MS showed **Compound 6** was consumed completely and 69% desired product was detected. The reaction mixture was concentrated under reduced pressure to afford **Compound 7** (40 mg, crude) as a light brown oil. MS-ESI (m/z) calcd for $C_{19}H_{28}N_4O$ $[M+H]^+$: 329.2. Found 329.2. To a solution of **Compound 7A** (21.76 mg, 106.56 umol) in EtOH(1 mL) was added NaOEt (14.50 mg, 213.12 umol) and the mixture was stirred at 80°C for 0.5 hr, then **Compound 7** (35 mg, 106.56 umol) in EtOH(1 mL) was added, and the resulting mixture was stirred at 80°C for 1 hr. LC-MS showed Reactant 1 was consumed completely and 35% desired product was detected. The reaction mixture was concentrated under reduced pressure to remove solvent. The residue was purified by prep-HPLC (basic condition) (column: Waters Xbridge Prep OBD C18 150*40mm*10um; mobile phase: [water (0.05%NH_3H_2O+10mM NH_4HCO_3)-ACN]; B%: 27%-47%, 8min) to afford ***38*** (7.2 mg, 14.88 umol, 13.96% yield, 97.05% purity) as a colorless gum. **^{1}H NMR** (METHANOL-d_4 400MHz) δ ppm 8.31 (s, 1 H) 8.09 (br s, 1 H) 7.58 (br d, J=7.94 Hz, 1 H) 6.82 (s, 1 H) 6.54 (d, J=9.04 Hz, 1 H) 3.58 - 3.69 (m, 4 H) 2.37 (br s, 4 H) 2.25 (s, 3 H) 1.81 (s, 6 H) 1.58 (quin, J=5.46 Hz, 4 H) 1.44 (br d, J=5.29 Hz, 2 H). **LCMS** (ESI+): *m/z* 470.3 (M+H).

The procedure to make 39 is similar with that of 38, except for the second step as shown below.

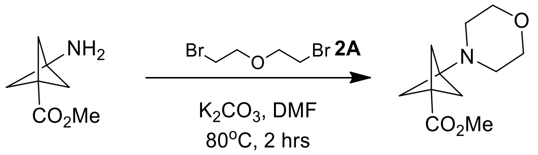

2 3

6-((2-((5-(4-methyl-1*H*-imidazol-2-yl)-4-(3-morpholinobicyclo[1.1.1]pentan-1-yl)pyrimidin-2-yl)amino)ethyl)amino)nicotinonitrile (Analog 39). To a solution of **Compound 2** (600 mg, 2.35 mmol TFA salt) and **Compound 2A** (1.64 g, 7.05 mmol, 884.22 uL) in ACN (10 mL) was added K_2CO_3 (1.95 g, 14.11 mmol) and the mixture was stirred at 80 °C for 2 hrs. TLC indicated that **Compound 2** was consumed, and one main spot was formed (Plate 1, Petroleum ether/Ethyl acetate=3/1, P1Rf=0.3). LC-MS showed **Compound 2** was consumed completely and one main peak with desired mass was detected. The reaction mixture was concentrated under reduced pressure to remove solvent. The residue was diluted with H_2O 30 mL and extracted with ethyl aceate (10 mL × 3). The combined organic layers were dried over Na_2SO_4, filtered and concentrated under reduced pressure to give a residue. The residue was purified by flash silica gel chromatography (ISCO®; 4 g SepaFlash® Silica Flash Column, Eluent of 0~20% Ethyl acetate/Petroleum ether gradient @ 50 mL/min) to obtain ***Compound 3*** (800 mg, 3.79 mmol, 80.53% yield) as a brown oil.

Analog 40

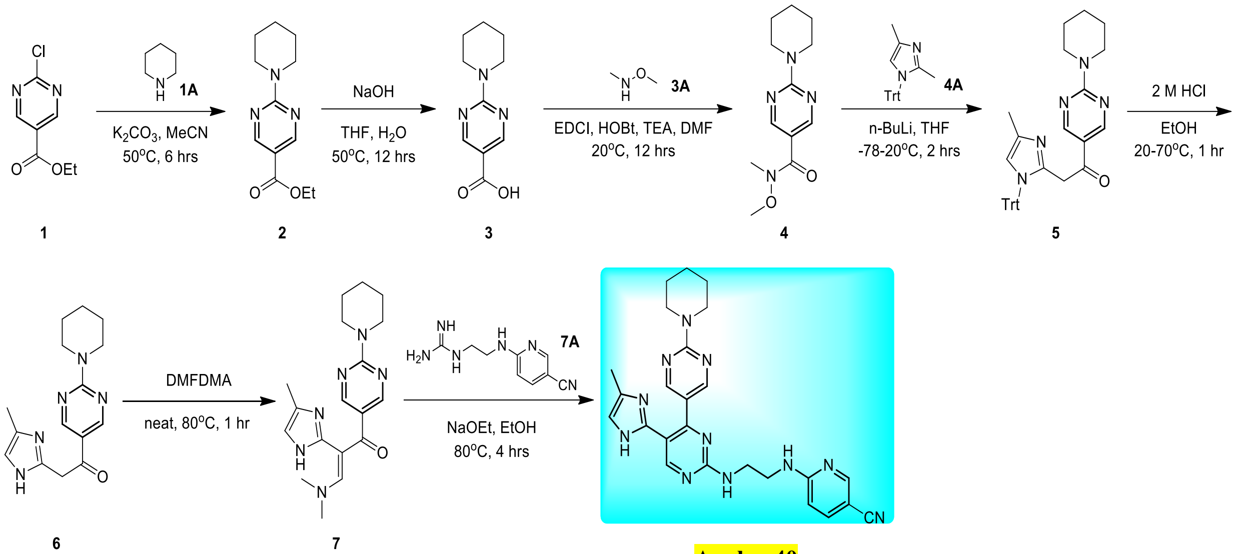

Analog 40

6-((2-((4-(4-(2-oxa-6-azaspiro[3.3]heptan-6-yl)phenyl)-5-(4-methyl-1*H*-imidazol-2-yl)pyrimidin-2-yl)amino)ethyl)amino)nicotinonitrile (Analog 40). To a solution of **Compound 1** (1.5 g, 9.73 mmol, 1.26 mL) and **Compound 1A** (1.84 g, 9.73 mmol, oxalic acid) in DMF (15 mL) was added K_2CO_3 (2.69 g, 19.46 mmol) at 20°C, then the mixture was stirred at 130°C for 12 hrs. TLC (Petroleum ether:Ethyl acetate=10:1)(P1 Rf=0.00) showed little of **Compound 1** was remained and a new spot was formed. The reaction was concentrated under vacuum. The residue was purfied by flash silica gel chromatography (ISCO®; 4 g SepaFlash® Silica Flash Column, Eluent of 0~30% Ethyl acetate/Petroleum ether gradient @ 18 mL/min) (Petroleum ether: Ethyl acetate=3:1) (P1 Rf=0.38) to afford ***Compound 2*** (492 mg, 2.11 mmol, 21.67% yield) as a white solid. 1H NMR (400 MHz, CHLOROFORM-d) δ ppm 7.90 (d, J=8.60 Hz, 2 H) 6.39 (d, J=8.60 Hz, 2 H) 4.86 (s, 4 H) 4.12 (s, 4 H) 3.86 (s, 3 H). To a solution of **Compound 2** (1.3 g, 5.57 mmol) in THF (30 mL) and MeOH (30 mL) was added NaOH (1 M, 30.00 mL) at 20°C, then the mixture was stirred at 50°C for 3 hrs. LCMS showed 7% of reactant 1 was remained and 87% of desired product was detected, then the mixture was stirred at 50°C for another 1 hr. TLC (Petroleum ether : Ethyl acetate=3:1)(P1 Rf=0.00) showed **Compound 2** was consumed and a new spot was formed. The MeOH and THF were removed under vacuum. The aqueous layer was acidized with 1 M HCl to pH=5. Solid was appeared, the mixture was filtered and the filter-cake was dried under vacuum to afford ***Compound 3*** (1.14 g, crude) as a white solid and it was used directly. MS-ESI (m/z) calcd for $C_{12}H_{13}NO_3$ $[M+H]^+$: 220.1. Found 220.0. To a solution of **Compound 3** (1.14 g, 5.20 mmol) and **Compound 3A** (608.66 mg, 6.24 mmol, HCl salt) in DMF (10 mL) were added EDCI (1.50 g, 7.80 mmol), HOBt (1.05 g, 7.80 mmol) and TEA (1.58 g, 15.60 mmol, 2.17 mL) at 20°C, then the mixture was stirred at 20°C for 12 hrs. LCMS showed **Compound 3** was consumed completely and 32% of desired product was detected. The reaction was concentrated under vacuum. The resiude was purified by flash silica gel chromatography (ISCO®; 4 g SepaFlash® Silica Flash Column, Eluent of 0~40% Ethyl acetate/Petroleum ether gradient @ 18 mL/min) (Petroleum ether:Ethyl acetate=0:1) (P1 Rf=0.60) to afford ***Compound 4*** (1.22 g, 4.65 mmol, 89.45% yield) as a white solid. MS-ESI (m/z) calcd for $C_{14}H_{18}N_2O_3$ $[M+H]^+$: 263.1. Found 263.1. n-BuLi (2.5 M, 2.29 mL) was added into THF (10 mL), then another solution of **Compound 4A** (645.14 mg, 1.91 mmol) in THF (15 mL) was added at -78°C, then the mixture was stirred at -78°C for 1 hr, then a solution of **Compound 4** (500 mg, 1.91 mmol) in THF (5 mL) was added at -78°C, then the mixture was stirred at 20°C for 1.5 hrs. LCMS showed 3% of **Compound 4** was remained and 45% of desired product was detected.

Water (10 mL) was added into the reaction, then the mixture was extracted with EtOAc (10 mL × 3), the organic layer was dried over anhydrous Na_2SO_4, filtered and the filtrate was concentrated under vacuum to afford ***Compound 5*** (1 g, crude) as a yellow solid and it was used directly. MS-ESI (m/z) calcd for $C_{36}H_{33}N_3O_2$ $[M+H]^+$: 540.3. Found 540.3 To a solution of **Compound 5** (585 mg, 1.08 mmol) in DCM (10 mL) was added TFA (1 mL) at 20°C, then the mixture was stirred at 20°C for 12 hrs. LCMS showed 3% of **Compound 5** was remained and 38% of desired product was detected. The reaction was concentrated under vacuum. 7.5 mL 1 M HCl was added and the aqueous layer was extracted with EtOAc (5 mL × 3), the organic layer was discarded and the aqueous layer was basified with saturated Na_2CO_3 solution to pH=8, then solid was appeared and the mixture was filtered and the filter-cake was dried under vacuum to afford ***Compound 6*** (133 mg, 447.28 umol, 41.26% yield) as a yellow solid and it was used directly. MS-ESI (m/z) calcd for $C_{17}H_{19}N_3O_2$ $[M+H]^+$: 298.2. Found 298.2. A solution of **Compound 6** (50 mg, 168.15 umol) in DMFDMA (2 mL) was stirred at 80°C for 1 hr. LCMS showed **Compound 6** was consumed and 23% of desired product was detected. The reaction was concentrated under vacuum to afford ***Compound 7*** (60 mg, crude) as a yellow liquid and it was used directly. MS-ESI (m/z) calcd for $C_{20}H_{24}N_4O_2$ $[M+H]^+$: 353.2. Found 353.3. A solution of **Compound 7A** (34.77 mg, 170.25 umol) and EtONa (34.76 mg, 510.74 umol) in EtOH (1.5 mL) was stirred at 80°C for 15 min, then another solution of **Compound 7** (60 mg, 170.25 umol) in EtOH (1.5 mL) was added at 80°C, then the mixture was stirred at 80°C for 12 hrs. LCMS showed 8% of **Compound 7** was remained and 60% of desired product was detected. The reaction was filtered. The filtrate was purified by Prep-HPLC (neutral condition) (column: Waters Xbridge BEH C18 100*25mm*5um; mobile phase: [water (10 mM NH_4HCO_3)-ACN]; B%: 20%-50%, 10 min) to afford ***40*** (22.9 mg, 44.68 umol, 26.24% yield, 96.29% purity) as a white solid. **^{1}H NMR** (METHANOL-d_4 400MHz) δ ppm 8.25 (br d, J=8.82 Hz, 2 H) 7.42 (br s, 1 H) 7.21 (d, J=8.60 Hz, 2 H) 6.72 (s, 1 H) 6.43 (br s, 1 H) 6.35 (d, J=8.60 Hz, 2 H) 4.83 (br s, 4 H) 4.06 (s, 4 H) 3.72 (br s, 2 H) 3.62 (br d, J=5.51 Hz, 2 H) 2.19 (s, 3 H). **LCMS** (ESI+): *m/z* 494.3 (M+H).

Analog 41

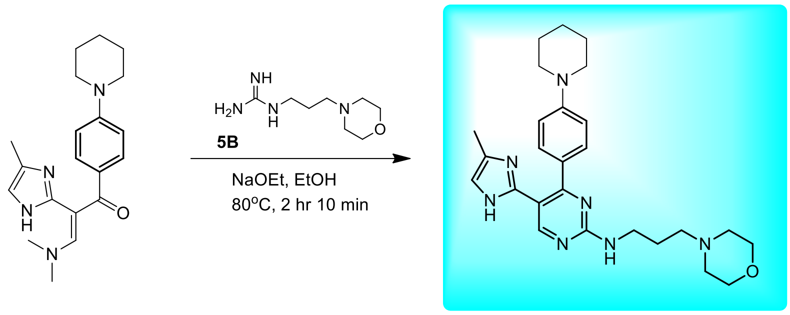

5   **Analog 41**

5-(4-methyl-1*H*-imidazol-2-yl)-*N*-(3-morpholinopropyl)-4-(4-(piperidin-1-yl)phenyl)pyrimidin-2-amine (Analog 41). To a solution of **Compound 5B** used above (65.81 mg, 295.47 umol, HCl salt) in EtOH (1.5 mL) was added EtONa (40.21 mg, 590.94 umol) at 20°C. The mixture was stirred at 80°C for 10 min. Then **Compound 5** (100 mg, 295.47 umol) in EtOH (1.5 mL) was added, and the mixture was stirred at 80°C for 2 hrs. LC-MS showed **Compound 5** was consumed, and one main peak with desired MS was detected. The reaction mixture was concentrated under reduced pressure to remove solvent. The residue was purified by prep-HPLC (neutral condition) (column: Phenomenex Gemini-NX C18 75*30mm*3um; mobile phase: [water (0.05%NH_3H_2O+10mM NH_4HCO_3)-ACN]; B%: 30%-60%, 8min) to afford ***analog 41*** (21.1 mg, 45.71 umol, 15.47% yield, 100% purity) as a light yellow solid. **^{1}H NMR** (METHANOL-*d*$_4$ 400MHz) δ ppm. 8.26 (br s, 1 H) 7.31 (br d, *J*=8.34 Hz, 2 H) 6.85 (d, *J*=8.94 Hz, 2 H) 6.74 (s, 1 H) 3.70 (t, *J*=4.59 Hz, 4 H) 3.52 (br s, 2 H) 3.21 - 3.28 (m, 4 H) 2.42 - 2.57 (m, 6 H) 2.20 (s, 3 H) 1.87 (quin, *J*=7.06 Hz, 2 H) 1.53 - 1.75 (m, 6 H). **LCMS** (ESI+): *m/z* 462.3 (M+H)

Analog 42

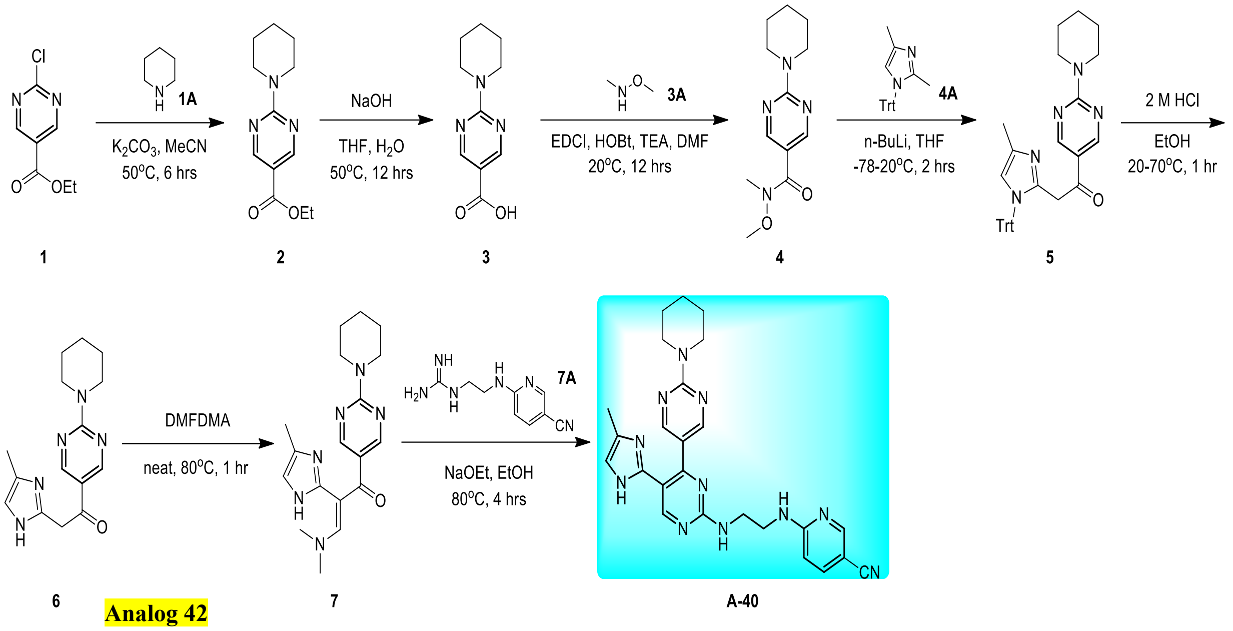

Analog 42

5-(4-methyl-1*H*-imidazol-2-yl)-*N*-(3-(piperazin-1-yl)propyl)-4-(4-(piperidin-1-yl)phenyl)pyrimidin-2-amine (Analog 42). A solution of **Compound 1** (86 mg, 353.41 umol) and **Compound 1A** (51.80 mg, 353.41 umol) in ACN (2 mL) was stirred at 80°C for 1 hr. LC-MS showed **Compound 1** was consumed completely and one main peak with desired mass was detected. The reaction mixture was concentrated under reduced pressure to remove solvent to afford ***Compound 2*** (120 mg, crude, HCl salt) as a colorless oil. MS-ESI (m/z) calcd for $C_{13}H_{27}N_5O_2$ $[M+H]^+$: 286.2. Found 286.2. The next reaction was carried out in 3 parallel batches. To a solution of **Compound 2** (38.04 mg, 118.19 umol, HCl salt) in EtOH (2 mL) was added EtONa (8.04 mg, 118.19 umol) at 20°C. The mixture was stirred at 80°C for 10 min. Then **Compound 2A** (40 mg, 118.19 umol) in EtOH (2 mL) was added at 80°C. The mixture was stirred at 80°C for 2 hrs. LC-MS showed 42% of **Compound 2A** was remained and 15% of desired compound was detected. The reaction was concentrated under vacuum. The reaction mixture was diluted with H_2O 3 mL and extracted with ethyl acetate (5 mL × 3). The combined organic layers were dried over Na_2SO_4, filtered and concentrated under reduced pressure to afford ***Compound 3*** (194 mg, crude) as a yellow liquid and it was used directly. MS-ESI (m/z) calcd for $C_{31}H_{44}N_8O_2$ $[M+H]^+$: 561.4. Found 561.3. To a solution of **Compound 3** (194 mg, 345.98 umol) in ACN (4 mL) was added HCl (2 mL) (3 M) at 20°C, then the mixture was stirred at 20°C for 12 hrs. LCMS showed **Compound 3** was consumed and 20% of desired product was detected. The reaction was filtered. The filtrate was purified by Prep-HPLC (HCl condition)(column: Phenomenex Luna C18 100*30mm*5um; mobile phase: [water (0.04% HCl)-ACN]; B%: 1%-5%, 10 min) to afford ***42*** (26 mg, 52.31 umol, 15.12% yield, 100% purity, HCl salt) as a yellow gum. **^{1}H NMR** (D_2O 400MHz) δ ppm 8.56 (br s, 1 H) 7.64 - 7.72 (m, 2 H) 7.60 (br s, 2 H) 7.10 (s, 1 H) 3.49 - 3.80 (m, 14 H) 3.37 - 3.44 (m, 2 H) 2.23 (s, 3 H) 2.14 (br d, *J*=7.09 Hz, 2 H) 1.96 - 2.05 (m, 4 H) 1.75 (br s, 2 H). **LCMS** (ESI+): *m/z* 461.2 (M+H)

Analog 43

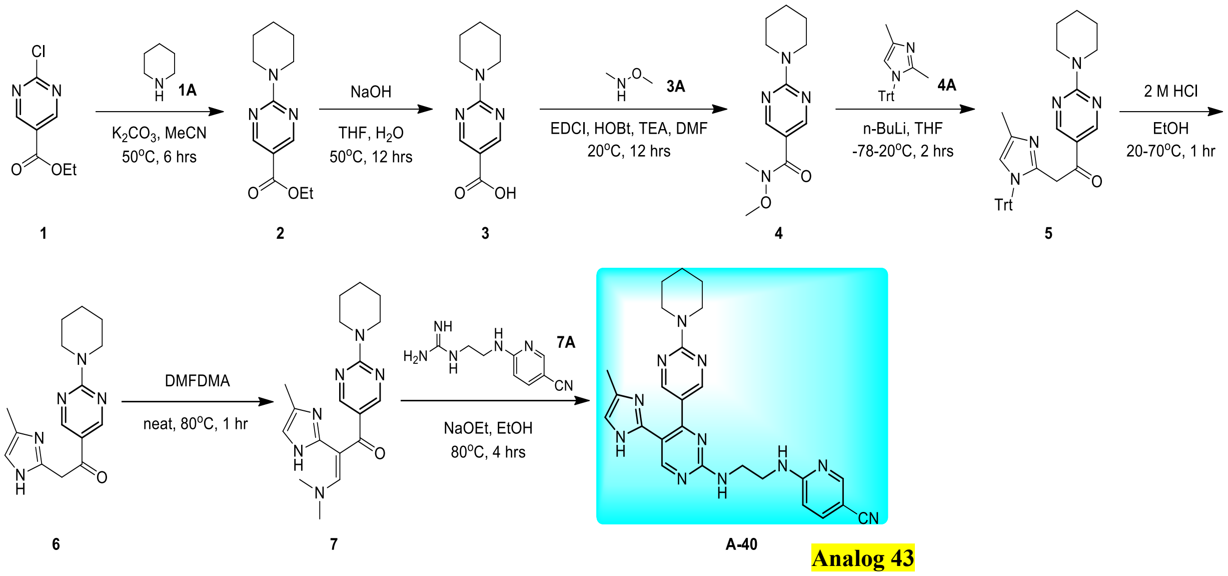

Analog 43

5-(4-methyl-1*H*-imidazol-2-yl)-4-(4-(piperidin-1-yl)phenyl)-*N*-(3-(thiazol-2-yl)propyl)pyrimidin-2-amine (Analog 43). To a solution of **Compound 1** (5 g, 33.78 mmol) in DMF (100 mL) was added **Compound 1A** (6.26 g, 33.78 mmol) at 20°C. The mixture was stirred at 100°C for 12 hrs. LC-MS showed **Compound 1** was consumed completely and one main peak with desired mass was detected. The reaction mixture was diluted with H_2O 400 mL and extracted with EtOAc (200 mL × 3). The combined organic layers were washed with brine (200 mL× 2), dried over Na_2SO_4, filtered and the filtrate was concentrated under reduced pressure to give a residue. The residue was purified by flash silica gel chromatography (ISCO®; 40 g SepaFlash® Silica Flash Column, Eluent of 0~30% Ethyl acetate/Petroleum ether gradient @ 100 mL/min) (SiO_2, Petroleum ether: Ethyl acetate=2:1, P1Rf=0.4) to afford ***Compound 2*** (5.5 g, 25.67 mmol, 76.00% yield) as a white solid. MS-ESI (m/z) calcd for $C_{12}H_{10}N_2O_2$ $[M+H]^+$: 215.1. Found 215.0. To a solution of **Compound 2** (500 mg, 2.33 mmol) in HCl/EtOAc (10 mL) was added **Compound 2A** (432.32 mg, 2.33 mmol). The mixture was stirred at 20°C for 12 hrs. LC-MS showed **Compound 2** was consumed completely and one main peak with desired mass was detected. The reaction mixture was filtered and the cake was washed with EtOAc (10 mL×2), then the cake was dried under reduced pressure to afford ***Compound 3*** (310 mg, crude) as a white solid. MS-ESI (m/z) calcd for $C_{12}H_{12}N_2O_2S$ $[M+H]^+$: 249.1. Found 249.0. To a solution of **Compound 3** (310 mg, 1.25 mmol) in ACETONE (3 mL) was added **Compound 3A** (270.64 mg, 1.37 mmol, 206.60 uL) at 20°C. The mixture was stirred at 60°C for 4 hrs. LC-MS showed **Compound 3** was consumed completely and one main peak with desired mass was detected. The reaction mixture was concentrated under reduced pressure to afford ***Compound 4*** (340 mg, crude) as a white solid. MS-ESI (m/z) calcd for $C_{14}H_{12}N_2O_2S$ $[M+H]^+$: 273.1. Found 273.0. To a solution of **Compound 4** (340 mg, 1.25 mmol) in EtOH (5 mL) was added hydrazine hydrate (294.12 mg, 4.99 mmol, 285.56 uL, 85% purity) at 20°C. The mixture was stirred at 80°C for 12 hrs. LC-MS showed **Compound 4** was consumed completely and one main peak with desired mass was detected. The reaction mixture was filtered to remove the insoluble, and the filtrate was concentrated under reduced pressure to afford ***Compound 5*** (180 mg, crude) as a yellow oil. MS-ESI (m/z) calcd for $C_6H_{10}N_2S$ $[M+H]^+$: 143.1. Found 143.0. To a solution of **Compound 5** (20 mg, 140.63 umol) in DMF (1 mL) was added DIEA (54.52 mg, 421.88 umol, 73.48 uL) and **Compound 5A** (20.61 mg, 140.63 umol) at 20°C. The mixture was stirred at 80°C for 12 hrs. LC-MS showed **Compound 5** was consumed completely and one main peak with desired mass was detected. ***Compound 6*** (25 mg, crude) in DMF was obtained as a yellow liquid, and the solution was

used to the next step directly. MS-ESI (m/z) calcd for $C_7H_{12}N_4S$ $[M+H]^+$: 185.1. Found 185.2. To a solution of **Compound 6A** (60 mg, 177.28 umol) in DMF (2 mL) was added Cs_2CO_3 (173.28 mg, 531.84 umol) and **Compound 6** (32.67 mg, 177.28 umol) at 20°C. The mixture was stirred at 80°C for 12 hrs. LC-MS showed **Compound 6A** was consumed and ~7% of desired product was detected. The reaction mixture was filtered to remove the insoluble, and the filtrate was concentrated under reduced pressure to give a residue. The residue was purified by prep-HPLC (basic condition) (column: Waters Xbridge Prep OBD C18 150*40mm*10um; mobile phase: [water (0.04%NH_3H_2O+10mM NH_4HCO_3)-ACN]; B%: 25%-55%, 8min) to afford ***43*** (17.6 mg, 37.11 umol, 34.11% yield, 96.9% purity) as a yellow solid. **^{1}H NMR** (METHANOL-d_4 400MHz) δ ppm 8.26 (s, 1 H) 7.68 (d, *J*=3.34 Hz, 1 H) 7.45 (d, *J*=3.46 Hz, 1 H) 7.30 (d, *J*=8.94 Hz, 2 H) 6.84 (d, *J*=9.06 Hz, 2 H) 6.74 (d, *J*=0.83 Hz, 1 H) 3.57 (br s, 2 H) 3.21 - 3.28 (m, 4 H) 3.16 (t, *J*=7.63 Hz, 2 H) 2.20 (s, 3 H) 2.14 (quin, *J*=7.21 Hz, 2 H) 1.60 - 1.71 (m, 6 H). **LCMS** (ESI+): *m/z* 460.2 (M+H)

Analog 44

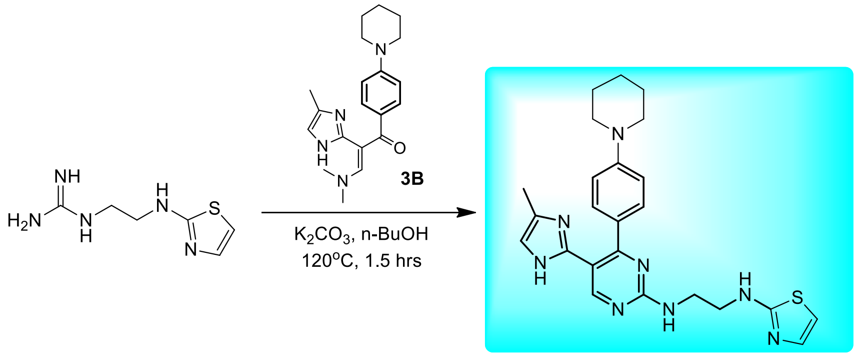

3

Analog 44

N*^{1}-(5-(4-methyl-1*H*-imidazol-2-yl)-4-(4-(piperidin-1-yl)phenyl)pyrimidin-2-yl)-*N*^{2}-(thiazol-2-yl)ethane-1,2-diamine (Analog 44).** To a solution of **Compound 3B** (120 mg, 354.56 umol) and **Compound 3** (65.68 mg, 354.56 umol) in n-BuOH (2 mL) was added K_2CO_3 (147.01 mg, 1.06 mmol) at 20°C, then the mixture was stirred at 120°C for 1.5 hrs. LCMS showed **Compound 3** was consumed and 27% of desired product was detected. The reaction was concentrated under vacuum. The residue was purified by Prep-HPLC (HCl condition)(column: Phenomenex luna C18 80*40mm*3 um; mobile phase: [water (0.04% HCl)-ACN]; B%: 5%-25%, 7 min), LCMS showed purity was 93%, then the residue was purified by Prep-HPLC (basic condition)(column: Phenomenex Gemini-NX C18 75*30mm*3um; mobile phase: [water(0.05% NH_3H_2O+10mM NH_4HCO_3)-ACN]; B%: 25%-55%, 8 min) to afford ***analog 44 (8.60 mg, 18.51 umol, 5.22% yield, 99.12% purity) as a pale yellow gum. **^{1}H NMR** (METHANOL-d_4 400MHz) δ ppm 8.28 (s, 1 H) 7.31 (br d, *J*=8.68 Hz, 2 H) 7.00 (d, *J*=3.67 Hz, 1 H) 6.84 (d, *J*=8.93 Hz, 2 H) 6.74 (s, 1 H) 6.53 (d, *J*=3.67 Hz, 1 H) 3.73 (br t, *J*=5.69 Hz, 2 H) 3.51 - 3.61 (m, 2 H) 3.21 - 3.27 (m, 4 H) 2.20 (s, 3 H) 1.57 - 1.72 (m, 6 H). **LCMS** (ESI+): *m/z* 461.2 (M+H)

Analog 45, 46, 47

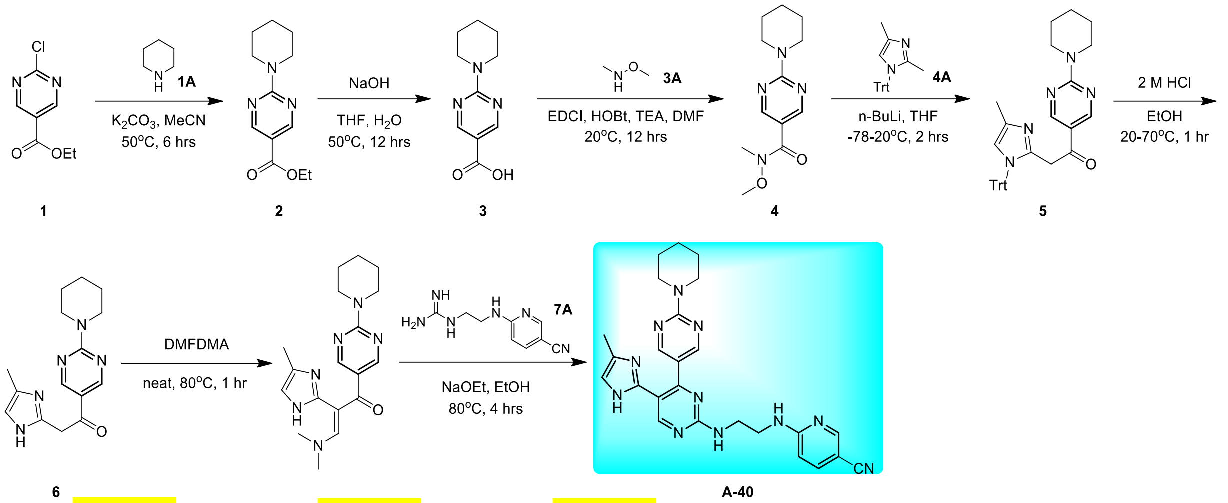

Analog 47 Analog 45 Analog 46

1-(4-(3-((5-(4-methyl-1*H*-imidazol-2-yl)-4-(4-(piperidin-1-yl)phenyl)pyrimidin-2-yl)amino)propyl)piperazin-1-yl)ethan-1-one (Analog 47). To a solution of **Compound 1** (0.5 g, 3.40 mmol, 549.45 uL) in ACN (10 mL) was added **Compound 1A** (497.84 mg, 3.40 mmol) at 20°C, then the mixture was stirred at 80°C for 12 hrs. LCMS showed **Compound 1** was consumed and a main peak with desired product was detected. The reaction was concentrated under vacuum to afford ***Compound 2*** (1.35 g, crude, HCl salt) was obtained as a pale yellow liquid and it was used directly. MS-ESI (m/z) calcd for $C_8H_{19}N_3O_2$ $[M+H]^+$: 190.2. Found 190.1. To a solution of **Compound 2** (240.09 mg, 1.06 mmol, HCl salt) in EtOH (3 mL) was added NaOEt (168.90 mg, 2.48 mmol) and the mixture was stirred at 80°C for 0.5 hr, then **Compound 2A** (240 mg, 709.12 umol) was added, and the resulting mixture was stirred at 80°C for 1 hr. LC-MS showed **Compound 2A** was consumed completely and one main peak with desired mass was detected. The reaction mixture was concentrated under reduced pressure to remove solvent. The residue was purified by flash silica gel chromatography (ISCO®; 12 g SepaFlash® Silica Flash Column, Eluent of 0~100% Ethyl acetate/Petroleum ether gradient @ 50 mL/min) (Petroleum ether/Ethyl acetate=0/1, P1Rf=0.2) to afford ***Compound 3*** (150 mg, 322.86 umol, 45.53% yield) as a yellow solid. MS-ESI (m/z) calcd for $C_{26}H_{36}N_6O_2$ $[M+H]^+$: 465.3. Found 465. To a solution of **Compound 3** (70 mg, 150.67 umol) in DCM (2 mL) was added TFA (718.67 mg, 6.30 mmol, 466.67 uL) and H_2O (233.33 mg, 12.95 mmol, 233.33 uL). The mixture was stirred at 20°C for 12 hrs. LC-MS showed **Compound 3** was consumed completely and one main peak with desired mass was detected. The reaction mixture was diluted with sat. $NaHCO_3$ 10 mL and then extracted with ethyl acetate (3 mL × 3). The combined organic layers were dried over Na_2SO_4, filtered and concentrated under reduced pressure to afford ***Compound 4*** (60 mg, crude) as a yellow solid. MS-ESI (m/z) calcd for $C_{12}H_{18}N_4O_2$ $[M+H]^+$: 391.2. Found 391.1. To a solution of **Compound 4** (60 mg, 153.66 umol) in DCM (2 mL) was added HOAc (922.71 ug, 15.37 umol) and **Compound 4A** (23.63 mg, 184.39 umol). The mixture was stirred at 20°C for 1 hr, then $NaBH(OAc)_3$ (97.70 mg, 460.97 umol) was added, and the resulting mixture was stirred at 20°C for 1 hr. LC-MS showed **Compound 4** was consumed completely and one main peak with desired mass was detected. The reaction mixture was concentrated under reduced pressure to remove solvent. The residue was purified by prep-HPLC (basic condition) (column: Waters Xbridge Prep OBD C18 150*40mm*10um; mobile phase: [water (0.05%NH_3H_2O+10mM NH_4HCO_3)-ACN]; B%: 20%-50%, 8min) to afford ***47*** (40.8 mg, 80.84 umol, 52.61% yield, 99.59% purity) as a white solid. **^{1}H NMR** (METHANOL-d_4 400MHz) δ ppm 8.26 (s, 1 H) 7.31 (br d, J=8.56 Hz, 2 H) 6.85 (d, J=9.05 Hz, 2 H) 6.73 (d, J=0.86 Hz, 1 H) 3.57 - 3.63 (m, 2 H) 3.54 (br t, J=5.07 Hz, 4 H) 3.21 - 3.27 (m, 4 H) 2.48 -

2.56 (m, 4 H) 2.43 - 2.48 (m, 2 H) 2.20 (s, 3 H) 2.08 (s, 3 H) 1.83 - 1.92 (m, 2 H) 1.59 - 1.70 (m, 6 H). **LCMS** (ESI+): *m/z* 503.3 (M+H)

Analog 45

General procedure for preparation of Compound 45

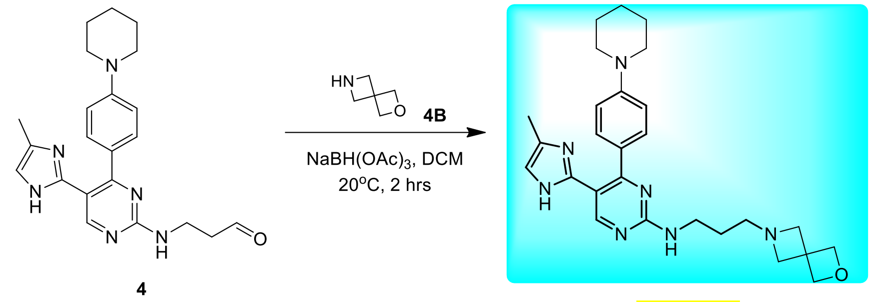

Analog 45

N*-(3-(2-oxa-6-azaspiro[3.3]heptan-6-yl)propyl)-5-(4-methyl-1*H*-imidazol-2-yl)-4-(4-(piperidin-1-yl)phenyl)pyrimidin-2-amine (45)**. To a solution of **Compound 4** (45 mg, 115.24 umol) and **Compound 4B** (26.16 mg, 138.29 umol, oxalic acid) in DCM (2 mL) was added TEA (13.99 mg, 138.29 umol, 19.25 uL) to make sure that the ph of the reaction mixture was about 7-8, then HOAc (692.06 ug, 11.52 umol) was added to adjust the ph to about 5-6, and the mixture was stirred at 20°C for 1 hr, then $NaBH(OAc)_3$ (122.12 mg, 576.21 umol) was added, and the resulting mixture was stirred at 20°C for 1 hr. LC-MS showed **Compound 4** was consumed completely and one main peak with desired mass was detected. The reaction mixture was diluted with $NaHCO_3$ 10 mL and extracted with DCM (3 mL × 3). The combined organic layers were dried over Na_2SO_4, filtered and concentrated under reduced pressure to give a residue. The residue was purified by prep-HPLC (basic condition) (column: Waters Xbridge Prep OBD C18 150*40mm*10um; mobile phase: [water (0.05%NH_3H_2O+10mM NH_4HCO_3)-ACN]; B%: 20%-60%, 8min) to afford ***A-53 (21.5 mg, 44.93 umol, 38.99% yield, 98.97% purity) as a pale yellow solid.

The procedure of making *46* is similar with that of *45*.

^{1}H NMR (METHANOL-d_4 400MHz) δ ppm 8.26 (s, 1 H) 7.32 (br d, J=8.60 Hz, 2 H) 6.85 (d, J=8.82 Hz, 2 H) 6.74 (s, 1 H) 4.72 (s, 4 H) 3.47 (br t, J=6.73 Hz, 2 H) 3.40 (s, 4 H) 3.20 - 3.27 (m, 4 H) 2.53 (t, J=7.50 Hz, 2 H) 2.20 (s, 3 H) 1.60 - 1.75 (m, 8 H). **LCMS** (ESI+): *m/z* 503.3 (M+H)

Analog 48

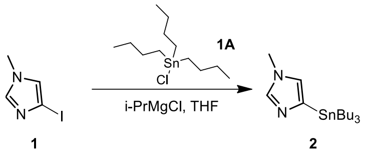

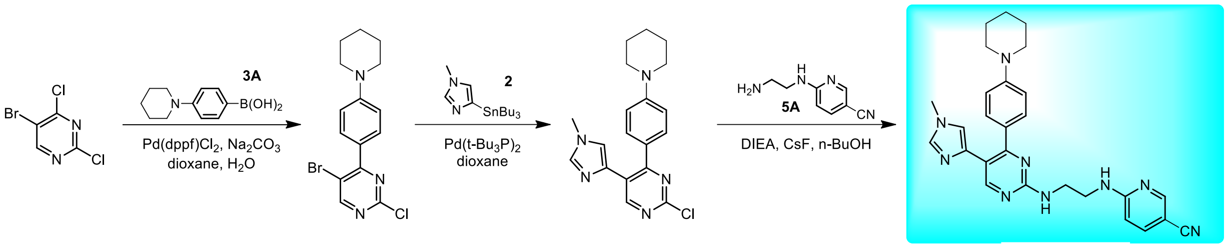

3 4 5

Analog 48

6-((2-((5-(1-methyl-1*H*-imidazol-4-yl)-4-(4-(piperidin-1-yl)phenyl)pyrimidin-2-yl)amino)ethyl)amino)nicotinonitrile (Analog 48). To a solution of **Compound 1** (1 g, 4.81 mmol) in THF (20 mL) at -10 °C was added i-PrMgCl (2 M, 3.61 mL) dropwise under N_2. The mixture was stirred for 1 hr at this temperature, and **Compound 1A** (1.64 g, 5.05 mmol, 1.36 mL) was added drop-wise. The reaction was stirred at 20°C for 12 hrs under N_2. LCMS showed **Compound 1** was consumed completely and desired mass was detected. The reaction mixture was diluted with saturated aqueous NH_4Cl (20 mL) and extracted with EtOAc (10 mL * 3). The combined organic phases were washed with water (10 mL * 2) and brine (20 mL), dried over Na_2SO_4, and concentrated to dryness to afford ***Compound 2*** (1.5 g, crude) as an orange oil. MS-ESI (m/z) calcd for $C_{16}H_{32}N_2Sn$ $[M+H]^+$: 373.2 Found 373.3. To a mixture of **Compound 3** (0.5 g, 2.19 mmol, 280.90 uL), **Compound 3A** (449.94 mg, 2.19 mmol) in dioxane (15 mL) and H_2O (2 mL) was added Na_2CO_3 (697.68 mg, 6.58 mmol) Pd(dppf)Cl_2 (16.06 mg, 21.94 umol) at 20 °C, the mixture was stirred at 50 °C for 12 hrs under N_2. LCMS showed **Compound 3** was consumed completely and desired mass was detected. The residue was diluted by H_2O 20 mL, then extracted with EtOAc (8 mL * 3). The combined organic layers were dried over Na_2SO_4, filtered and concentrated under reduced pressure to give a residue. The residue was purified by flash silica gel chromatography (ISCO®; 12 g SepaFlash® Silica Flash Column, Eluent of 0~10% Ethyl acetate/Petroleum ether gradient @ 80 mL/min) to afford ***Compound 4*** (0.3 g, 850.69 umol, 38.77% yield) as a yellow solid. MS-ESI (m/z) calcd for $C_{15}H_{15}BrClN_3$ $[M+H]^+$: 354.0/352.0 Found 354.1/352.1. To a solution of **Compound 4** (0.2 g, 567.12 umol) and **Compound 2** (210.49 mg, 567.12 umol) in dioxane (8 mL) was added Pd(t-$Bu_3P)_2$ (28.98 mg, 56.71 umol) under N_2. The resulting mixture was stirred at 110°C for 12 hrs. LCMS showed **Compound 4** was consumed completely and desired mass was detected. The reaction mixture was quenched by pouring into water 10 mL and extracted with EtOAc (4 mL * 3), dried over Na_2SO_4, filtered and concentrated under reduced pressure to give a residue. The residue was purified by prep-HPLC (column: Phenomenex Luna C18 75*30mm*3um; mobile phase: [water (0.2%FA) - ACN]; B%: 1%-35%, 8 min) to afford ***Compound 5*** (80 mg, 226.09 umol, 39.87% yield) as a yellow solid. MS-ESI (m/z) calcd for $C_{19}H_{20}ClN_5$ $[M+H]^+$: 354.1/356.1 Found 354.3/356.3. To a solution of **Compound 5A** (42.11 mg, 211.96 umol, HCl) in n-BuOH (1.2 mL) was added DIEA (54.79 mg, 423.91 umol, 73.84 uL), CsF (12.88 mg, 84.78 umol, 3.13 uL) and **Compound 5** (30 mg, 84.78 umol). The reaction vessel was sealed and heated in microwave at 140°C for 8 hrs. LCMS showed **Compound 5** was remained and desired mass was detected. The reaction mixture was quenched by pouring into water 4 mL and extracted with EtOAc (2 mL * 3), dried over Na_2SO_4, filtered and concentrated under reduced pressure to give a residue. The residue was

purified by prep-HPLC (column: Phenomenex Gemini-NX C18 75*30mm*3um; mobile phase: [water (0.05% NH_3H_2O + 10mM NH_4HCO_3) - ACN]; B%: 25%-65%, 8 min) to afford ***48*** (12.8 mg, 26.69 umol, 31.48% yield, 100% purity) as a pale yellow solid. **^{1}H NMR** (DMSO-d_6 400MHz) δ ppm 8.45 - 8.30 (m, 2H), 7.82 - 7.59 (m, 2H), 7.56 (s, 1H), 7.33 (d, J=8.8 Hz, 2H), 7.17 (br s, 1H), 6.83 (br d, J=8.8 Hz, 2H), 6.67 (s, 1H), 6.55 (br s, 1H), 3.57 (s, 3H), 3.51 (br s, 4H), 3.20 (br d, J=4.9 Hz, 4H), 1.57 (br s, 6H). **LCMS** (ESI+): *m/z* 480.3 (M+H)

Analog 49, 77

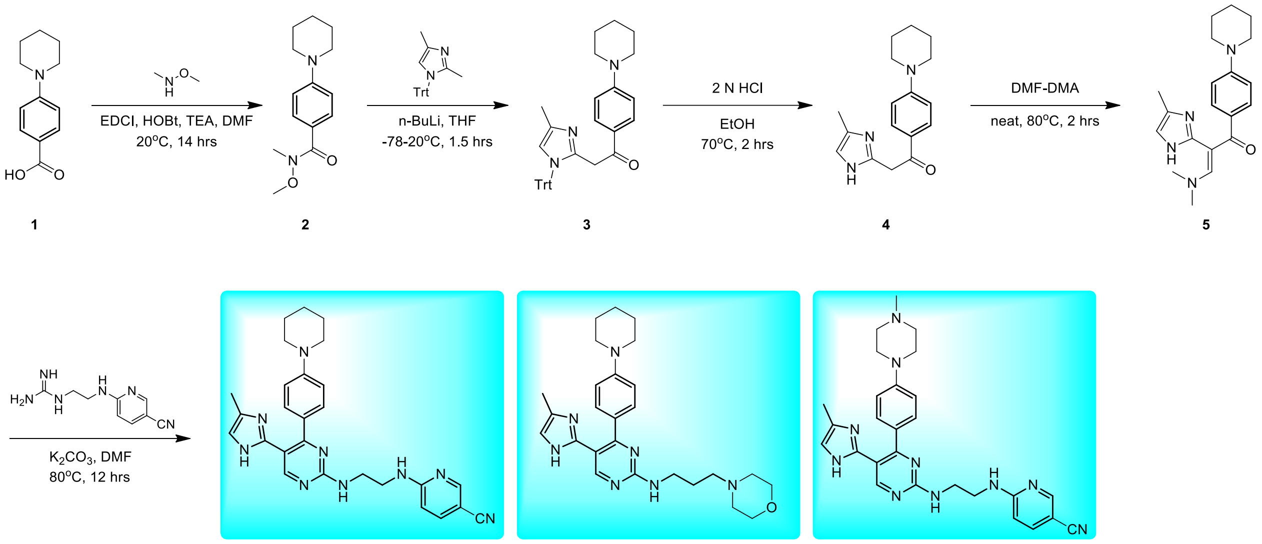

Analog 49 A-8 **Analog 77** A-49 A-9

4-(2,4-dichlorophenyl)-*N*-(3-morpholinopropyl)-5-(oxazol-2-yl)pyrimidin-2-amine (Analog 49). To a solution of **Compound 1** (100 mg, 1.20 mmol) in ACN (5 mL) was added TEA (450.60 mg, 4.45 mmol, 619.81 uL) and **Compound 1A** (756.27 mg, 3.61 mmol, 504.18 uL) at 20°C. The mixture was stirred at 80°C for 12 hrs. TLC indicated that **Compound 1** was consumed, and one main spot was formed (Petroleum ether/Ethyl acetate=5/1, P1Rf=0.3). The reaction mixture was filtered and the cake was washed with EtOAc (5 mL×2), then the cake was discarded, and the filtrate was concentrated under reduced pressure to give a residue. The residue was purified by flash silica gel chromatography (ISCO®; 12 g SepaFlash® Silica Flash Column, Eluent of 0~15% Ethyl acetate/Petroleum ether gradient @ 75 mL/min) (Petroleum ether/Ethyl acetate=5/1, P1Rf=0.3) to afford ***Compound 2*** (250 mg, 582.64 umol, 48.41% yield) as a yellow oil. To a solution of **Compound 2** (250 mg, 582.64 umol) in MeOH (3 mL) was added KOH (65.38 mg, 1.17 mmol) and the mixture was stirred at 20°C for 12 hrs. LC-MS showed **Compound 2** was consumed completely and one main peak with desired mass was detected. The

reaction mixture was concentrated under reduced pressure to remove solvent. The residue was diluted with H_2O 10 mL and the aqueous phase was treated with 1M HCl to adjust the pH to about 5, then the aqueous phase was extracted with DCM (5 mL×3). The combined organic layers were dried over Na_2SO_4, filtered and concentrated under reduced pressure to give a residue. The residue was purified by flash silica gel chromatography (ISCO®; 4 g SepaFlash® Silica Flash Column, Eluent of 0~15% Ethyl acetate/Petroleum ether gradient @ 75 mL/min) (Petroleum ether/Ethyl acetate=5/1, P1Rf=0.4) to afford ***Compound 3*** (80 mg, 312.40 umol, 53.62% yield) as a purple solid. MS-ESI (m/z) calcd for $C_{11}H_7O_2Cl_2N$ $[M+H]^+$: 256.0/258.0. Found 255.9/257.9. A mixture of **Compound 3** (80 mg, 312.40 umol) in DMFDMA (1 mL) was stirred at 80°C for 1 hr under N_2 atmosphere. LC-MS showed **Compound 3** was consumed completely and one main peak with desired mass was detected. The reaction mixture was concentrated under reduced pressure to remove solvent to afford ***Compound 4*** (98 mg, crude) as a dark green oil. MS-ESI (m/z) calcd for $C_{14}H_{12}O_2Cl_2N_2$ $[M+H]^+$: 311.0/313.0. Found 311.0/313.0. To a solution of **Compound 4A** (70.14 mg, 314.95 umol, HCl salt) in EtOH (2 mL) was added NaOEt (42.86 mg, 629.90 umol) and the mixture was stirred at 80°C for 0.5 hr, then **Compound 4** (98 mg, 314.95 umol) was added, and the resulting mixture was stirred at 80°C for 1 hr. LC-MS showed **Compound 4** was consumed completely and one main peak with desired mass was detected. The reaction mixture was filtered and the filtrate was collected. The filtrate was purified by prep-HPLC (neutral condition) (column: mobile phase: [water (10mM NH_4HCO_3)-ACN]; B%: 40%-70%, 6min) to afford ***49*** (21.90 mg, 50.22 umol, 15.95% yield, 99.60% purity) as a white solid. **^{1}H NMR** (METHANOL-d_4 400MHz) δ ppm 8.78 - 8.97 (m, 1 H) 7.72 (s, 1 H) 7.50 (s, 1 H) 7.38 - 7.47 (m, 2 H) 7.13 (s, 1 H) 3.65 (br s, 4 H) 3.49 (br s, 2 H) 2.46 (br s, 6 H) 1.85 (br s, 2 H). **LCMS** (ESI+): *m/z* 434.1/436.1 (M+H)

Analog 50, 78

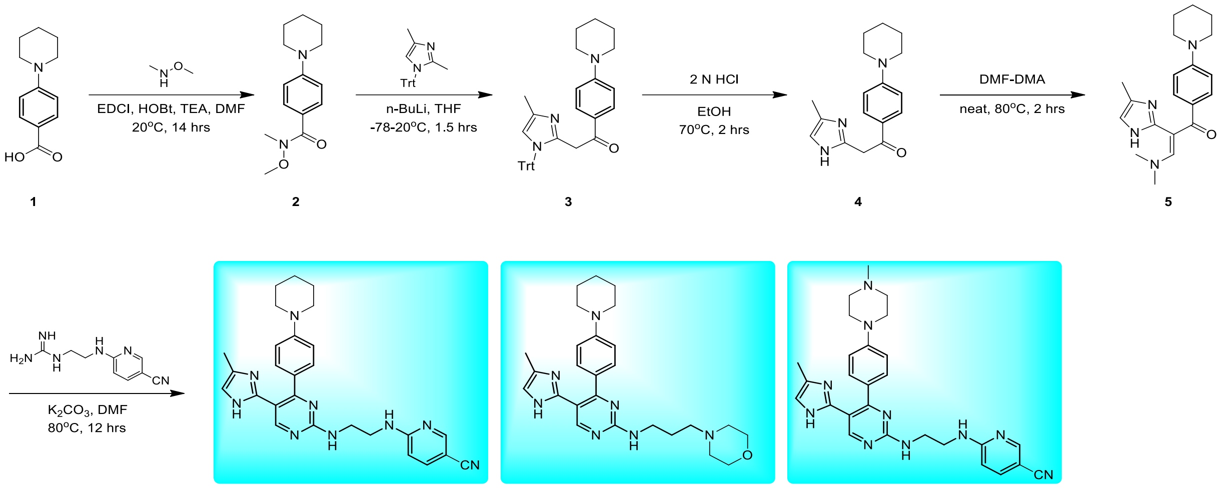

Analog 50 A-8 Analog 78 A-49 A-9

4-(2,4-dichlorophenyl)-*N*-(3-morpholinopropyl)-5-(thiazol-2-yl)pyrimidin-2-amine (Analog 50). To a solution of **Compound 1** (0.5 g, 5.04 mmol) and TEA (1.84 g, 18.15 mmol, 2.53 mL) in MeCN (6 mL) was added **Compound 1A** (3.17 g, 15.13 mmol, 2.11 mL) at 20°C, then the mixture was stirred at 80°C for 12 hrs. LCMS showed the **Compound 1** was consumed and 39% of desired product was detected. The reaction was concentrated under vacuum. The residue was diluted with DCM (50 mL), the organic layer was washed with saturated $NaHCO_3$ solution (10 mL), the organic layer was dried over anhydrous Na_2SO_4, filtered and the filtrate was concentrated under vacuum. The residue was purified by flash silica gel chromatography (ISCO®; 12 g SepaFlash® Silica Flash Column, Eluent of 0~10% Ethyl acetate/Petroleum ether gradient @ 75 mL/min) (Petroleum ether: Ethyl acetate=5:1) (P1 Rf=0.39) to afford ***Compound 2*** (2.2 g, 4.94 mmol, 98.01% yield) as a brown liquid. MS-ESI (m/z) calcd for $C_{18}H_9Cl_4NO_2S$ $[M+H]^+$: 443.9/445.9/447.9. Found 443.9/445.9/447.9. To a solution of **Compound 2** (1 g, 2.25 mmol) in MeOH (15 mL) was added another solution of KOH (252.08 mg, 4.49 mmol) in MeOH (10 mL) at 20°C, then the mixture was stirred at 20°C for 12 hrs. LCMS showed the **Compound 2** was consumed and 87% of desired product was detected. The reaction was concentrated under vacuum. The residue was dissolved in H_2O (30 mL), the aqueous layer was neutralized with 1 M HCl to pH=7, then the aqueous layer was extracted with DCM (20 mL × 3), the organic layer was dried over anhydrous Na_2SO_4, filtered and the filtrate was concentrated under vacuum. The residue was purified by flash silica gel chromatography (ISCO®; 12g SepaFlash® Silica Flash Column, Eluent of 0~10% Ethyl acetate/Petroleum ether gradient @ 36 mL/min) (Petroleum ether: Ethyl acetate=5:1) (P1 RF=0.49) to afford ***Compound 3*** (444 mg, 1.63 mmol, 72.62% yield) as a gray solid. MS-ESI (m/z) calcd for $C_{11}H_7Cl_2NOS$ $[M+H]^+$: 272.0/274.0. Found 271.9/273.9. A solution of **Compound 3** (444 mg, 1.63 mmol) in DMFDMA (8 mL) was stirred at 80°C for 45 min. LCMS showed the **Compound 3** was consumed and 34% of desired product was detected. The reaction was concentrated under vacuum to afford ***Compound 4*** (600 mg, crude) as a brown liquid and it was used directly. MS-ESI (m/z) calcd for $C_{14}H_{12}Cl_2N_2OS$ $[M+H]^+$: 327.0/329.0. Found 327.0/329.0. To a solution of **Compound 4A** (156.54 mg, 702.87 umol, HCl salt) in EtOH (2 mL) was added EtONa (47.83 mg, 702.87 umol) at 20°C, then the mixture was stirred at 80°C for 10 min, then another solution of **Compound 4** (230 mg, 702.87 umol) in EtOH (2 mL) was added at 80°C, then the mixture was stirred at 80°C for 2 hrs. LCMS showed 28% of **Compound 4** was remained and 50% of desired product was detected. The reaction was filtered. The filtrate was purified by Prep-HPLC (neutral condition) (column: Waters Xbridge BEH C18 100*30mm*10um; mobile phase: [water (10mM NH_4HCO_3)-ACN]; B%: 35%-55%, 6 min) to afford ***50***

(60.2 mg, 133.12 umol, 18.94% yield, 99.59% purity) as a pale yellow solid. **^{1}H NMR** (METHANOL-d_4 400MHz) δ ppm 8.93 (br s, 1 H) 7.73 (d, J=3.30 Hz, 1 H) 7.57 (s, 1 H) 7.47 - 7.53 (m, 1 H) 7.39 - 7.45 (m, 2 H) 3.67 (br s, 4 H) 3.50 (br s, 2 H) 2.47 (br s, 6 H) 1.85 (br s, 2 H). **LCMS** (ESI+): m/z 450.1/452.1 (M+H)

Analog 51

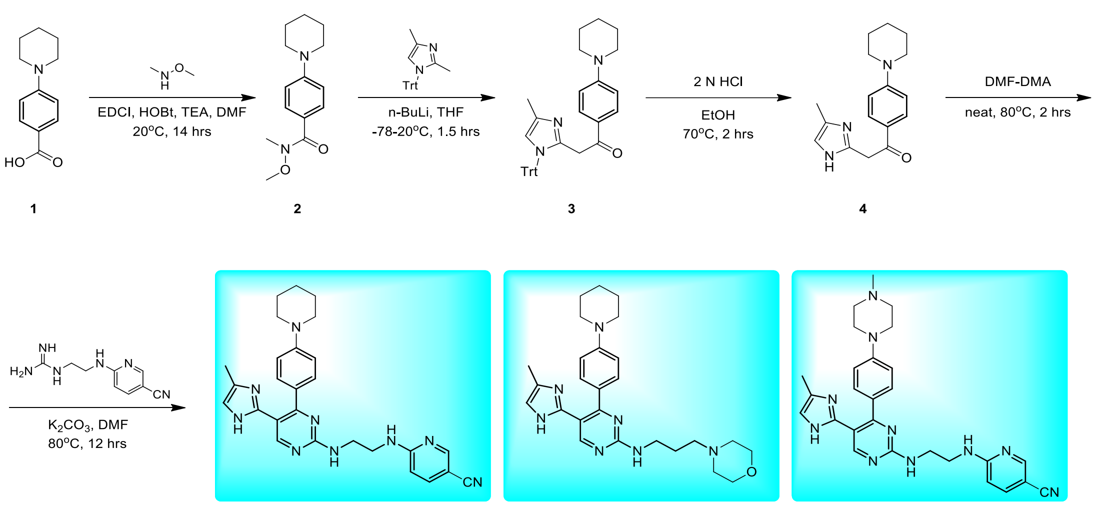

A-8 A-49 A-9 **Analog 51**

4-(2,4-dichlorophenyl)-5-(1*H*-imidazol-2-yl)-*N*-(3-morpholinopropyl)pyrimidin-2-amine (Analog 51). To a solution of **Compound 1b** (0.5 g, 6.09 mmol) in DCM (16 mL) was heated to 40°C until the solution was clear, then DIEA (2.52 g, 19.49 mmol, 3.39 mL) was added in one portion at 30°C, another solution of **Compound 1a** (3.85 g, 18.39 mmol, 2.57 mL) in DCM (3mL) was added drop wised under water bath (keep the temperature below 35°C), the reaction was stirred at 50°C for 12 hrs. Then the reaction was concentrated under vacuum until the solid was dry. The dry solid was added into a solution of AcOH (13.8 mL) and HCl (6.9 mL) (12 M), the mixture was stirred at 125°C for 3.5 hrs. LCMS showed the **Compound 1b** was consumed and 68% of desired product was detected. The reaction was concentrated under vacuum. Then water (34.5 mL) and toluene (17.2 mL) were added to the liquid, the mixture was vigorously stirred for 30 min at 20°C. Solid was appeared and was filtered, rinsed with 8.5 mL of water and the solid was discarded. The filtrate was transferred into a separatory funnel, the organic layer was discarded and the aqueous layer was washed with toluene (17 mL × 2), The aqueous

layer was transferred into a large breaker and diluted with MTBE (8.5 mL), then the stirred mixture was basified with saturated $NaHCO_3$ solution to pH=8. DCM (34.5 mL) was added and stirred for 10 min at 20°C, and the aqueous layer was extracted with DCM (17 mL × 1), the organic layers were combined and washed with saturated $NaHCO_3$ solution (17 mL), water (17 mL) and brine (17 mL), dried over Na_2SO_4, filtered and the filtrate was concentrated under vacuum to afford ***Compound 2a*** (2.09 g, 8.12 mmol, 66.69% yield, 99.14% purity) as an off-white solid. MS-ESI (m/z) calcd for $C_{11}H_8Cl_2N_2O$ $[M+H]^+$: 255.0/257.0. Found 254.9/256.9

A solution of **Compound 2a** (0.5 g, 1.96 mmol) in DMFDMA (10 mL) was stirred at 80°C for 1.5 hrs. LCMS showed the **Compound 2a** was consumed and 58% of desired product was detected. The reaction was concentrated under vacuum to afford ***Compound 3a*** (620 mg, crude) as a brown liquid and it was used directly. MS-ESI (m/z) calcd for $C_{14}H_{13}Cl_2N_3O$ $[M+H]^+$: 310.0/312.0. Found 310.0/312.0. To a solution of **Compound 1** (100 mg, 693.41 umol, 101.32 uL) in ACN (3 mL) was added **Compound 1c** (101.64 mg, 693.41 umol) at 20°C, then the mixture was stirred at 80°C for 6 hrs. LCMS showed trace of **Compound 1** was remained and 99% of desired product was detected. The reaction was concentrated under vacuum to afford ***Compound 2*** (160 mg, crude, HCl salt) as a colorless liquid and it was used directly. MS-ESI (m/z) calcd for $C_8H_{18}N_4O$ $[M+H]^+$: 187.2. Found 187.1. To a solution of **Compound 2** (160 mg, 718.41 umol, HCl salt) in EtOH (2 mL) was added EtONa (48.89 mg, 718.41 umol) at 20°C, then the mixture was stirred at 80°C for 10 min, then another solution of **Compound 3a** (222.83 mg, 718.41 umol) in EtOH (2 mL) was added at 80°C, then the mixture was stirred at 80°C for 2 hrs. LCMS showed the **Compound 3a** was consumed and 23% of desired product was detected. The reaction was filtered. The filtrate was purified by Prep-HPLC (neutral condition) (column: Phenomenex Gemini-NX 150*30mm*5um; mobile phase: [water (10mM NH_4HCO_3)-ACN]; B%: 18%-38%, 8 min) to afford ***51*** (21.20 mg, 47.96 umol, 6.68% yield, 98.03% purity) as a pale yellow solid. **^{1}H NMR** (METHANOL-d_4 400MHz) δ ppm 8.53 (br s, 1 H) 7.39 - 7.44 (m, 3 H) 6.97 (s, 2 H) 3.67 (br s, 4 H) 3.45 - 3.53 (m, 2 H) 2.47 (br s, 6 H) 1.85 (quin, *J*=7.12 Hz, 2 H). **LCMS** (ESI+): *m/z* 433.1/435.1 (M+H).

Analog 52, 80

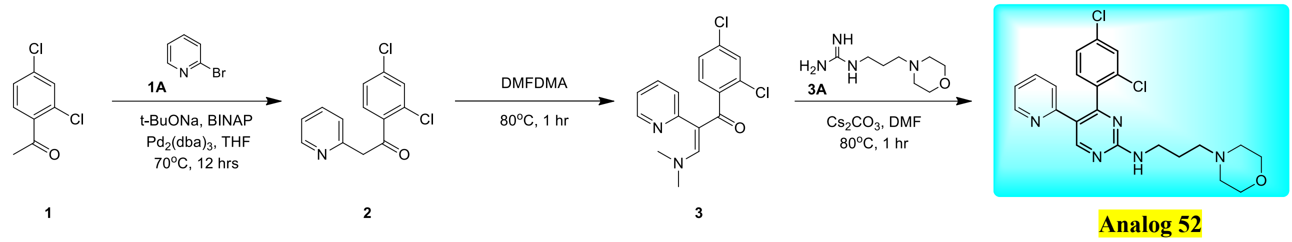

Analog 52

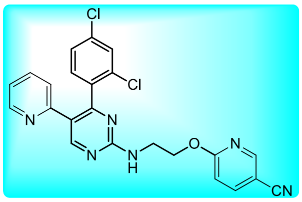

Analog 80

4-(2,4-dichlorophenyl)-*N*-(3-morpholinopropyl)-5-(pyridin-2-yl)pyrimidin-2-amine (Analog 52). To a solution of **Compound 1A** (2 g, 12.66 mmol, 1.20 mL) and **Compound 1** (2.87 g, 15.19 mmol) in THF (40 mL) was added t-BuONa (2.43 g, 25.32 mmol) and BINAP (394.11 mg, 632.93 umol) and $Pd_2(dba)_3$ (289.79 mg, 316.46 umol). The mixture was stirred at 70°C for 12 hrs under N_2 atmosphere. TLC indicated that **Compound 1A** was consumed and one main spot was formed (Petroleum ether/Ethyl acetate=3/1, P1Rf=0.7). The reaction mixture was concentrated under reduced pressure to remove solvent. The residue was purified by flash silica gel chromatography (ISCO®; 12 g SepaFlash® Silica Flash Column, Eluent of 0~15% Ethyl acetate/Petroleum ether gradient @ 50 mL/min) (Petroleum ether/Ethyl acetate=3/1, P1Rf=0.7) to afford ***Compound 2*** (2 g, 53% yield) as a yellow solid. A mixture of **Compound 2** (200 mg, 751.53 umol) in DMFDMA (2 mL) was stirred at 80°C for 1 hr under N_2 atmosphere. The LC-MS showed **Compound 2** was consumed completely and one main peak with desired mass was detected. The reaction mixture was concentrated under reduced pressure to remove solvent to afford ***Compound 3*** (240 mg, crude) as a brown oil. MS-ESI (m/z) calcd for $C_{16}H_{14}OCl_2N_2$ $[M+H]^+$: 321.1/323.1. Found 321.0/323.0. To a solution of **Compound 3** (200 mg, 622.66 umol) and **Compound 3A** (138.68 mg, 622.66 umol, HCl salt) in DMF (3 mL) was added Cs_2CO_3 (608.63 mg, 1.87 mmol) and the mixture was stirred at 80°C for 1 hr. The LC-MS showed **Compound 3** was consumed completely and one main peak with desired mass was detected. The reaction was filtered and the filtrate was collected. The filtrate was purified by Prep-HPLC (basic condition) (column: Phenomenex Gemini-NX C18 75*30mm*3um; mobile phase: [water (0.05%NH_3H_2O+10mM NH4HCO3)-ACN]; B%: 28%-58%, 8min) to afford **52** (58.90 mg, 132.55 umol, 21.29% yield, 100% purity) as a yellow solid. **^{1}H NMR** (METHANOL-d_4 400MHz) δ ppm 8.60 (s, 1 H) 8.46 - 8.51 (m, 1 H) 7.60 (td, *J*=7.79, 1.77 Hz, 1 H) 7.34 - 7.45 (m, 3 H) 7.23 (ddd, *J*=7.58, 4.95, 1.04 Hz, 1 H) 6.99 (d, *J*=7.95 Hz, 1 H) 3.68 (br s, 4 H) 3.51 (br t, *J*=6.72 Hz, 2 H) 2.48 (br s, 6 H) 1.86 (quin, *J*=7.12 Hz, 2 H). **LCMS** (ESI+): *m/z* 444.1/446.1 (M+H).

Analog 53, 81

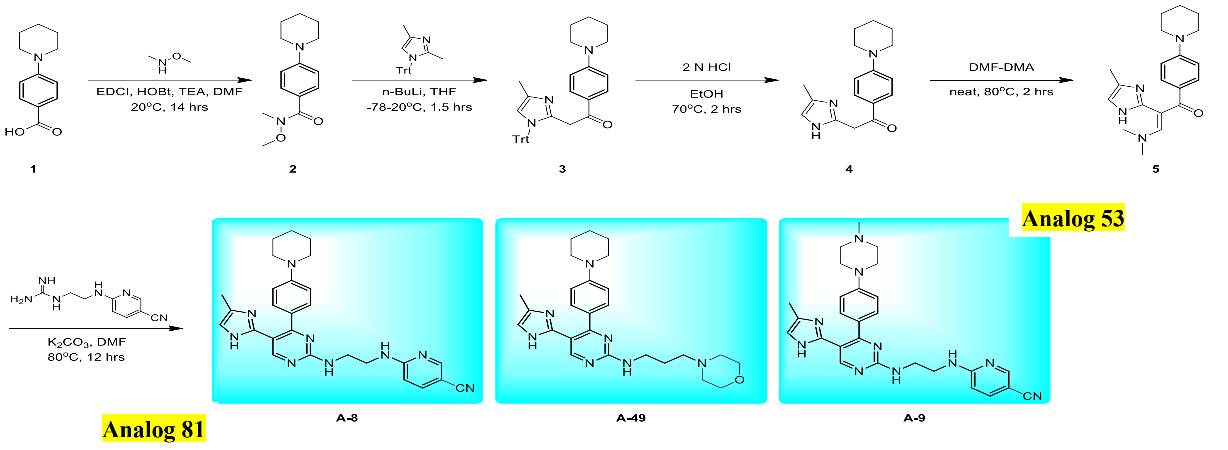

4-(2,4-dichlorophenyl)-5-(4-methylpiperazin-1-yl)-*N*-(3-morpholinopropyl)pyrimidin-2-amine (Analog 53). To a solution of **Compound 1A** (22.41 g, 223.73 mmol, 24.82 mL) in DMF (15 mL) was added drop wised another solution of **Compound 1** (5 g, 22.37 mmol) in DMF (10 mL) at 20°C, then the mixture was stirred at 20°C for 12 hrs. LCMS showed **Compound 1** was consumed and 61% of desired product was detected. The reaction was concentrated under vacuum. The residue was purified by flash silica gel chromatography (ISCO®; 40 g SepaFlash® Silica Flash Column, Eluent of 0~68% Ethyl acetate/Petroleum ether gradient @ 100 mL/min) (Dichloromethane: Methanol=10:1) (P1 Rf=0.19) to afford ***Compound 2*** (3.96 g, 13.79 mmol, 61.63% yield) as a brown liquid. MS-ESI (m/z) calcd for $C_{13}H_{16}Cl_2N_2O$ $[M+H]^+$: 287.1/289.1. Found 287.0/289.0. A solution of **Compound 2** (3.46 g, 12.05 mmol) in DMFDMA (60 mL) was at 80°C for 12 hrs. LCMS showed 9% of **Compound 2** was remained and 20% of desired product was detected. The reaction was concentrated under vacuum. The residue was diluted with MTBE (20 mL), the mixture was stirred at 20°C for 10 min, then the mixture was filtered and the filter-cake was dried under vacuum to afford ***Compound 3*** (1.08 g, 3.16 mmol, 26.19% yield) as a yellow solid. MS-ESI (m/z) calcd for $C_{16}H_{21}Cl_2N_3O$ $[M+H]^+$: 342.1/344.1. Found 342.1/344.1. To a solution of **Compound 3A** (108.84 mg, 584.35 umol) in EtOH (2 mL) was added EtONa (119.29 mg, 1.75 mmol) at 20°C, then the mixture was stirred at 80°C for 10 min, then another solution of **Compound 3** (200 mg, 584.35 umol) in EtOH (2 mL) was added at 80°C, then the mixture was stirred at 80 °C for 12 hrs. LCMS showed 4% of **Compound 3** was remained and there was 22% of desired product was detected. The reaction was filtered. The filtrate was purified by Prep-HPLC (neutral condition) (column: Phenomenex Gemini-NX 150*30mm*5um; mobile phase: [water (10 mM NH_4HCO_3)-ACN]; B%: 30%-50%, 8 min) to afford ***53*** (27.3 mg, 55.86 umol, 9.56% yield, 95.23% purity) as a yellow solid. **^{1}H NMR** (METHANOL-d_4 400MHz) δ ppm 8.23 (s, 1 H) 7.58 (d, *J*=1.34 Hz, 1 H) 7.39 - 7.46 (m, 2 H) 3.67 (t, *J*=4.71 Hz, 4 H) 3.40 (t, *J*=6.85 Hz, 2 H) 2.81 (t, *J*=4.71 Hz, 4 H) 2.24 - 2.53 (m, 10 H) 2.22 (s, 3 H) 1.81 (quin, *J*=7.15 Hz, 2 H). **LCMS** (ESI+): *m/z* 465.2/467.2 (M+H).

Analog 54, 82

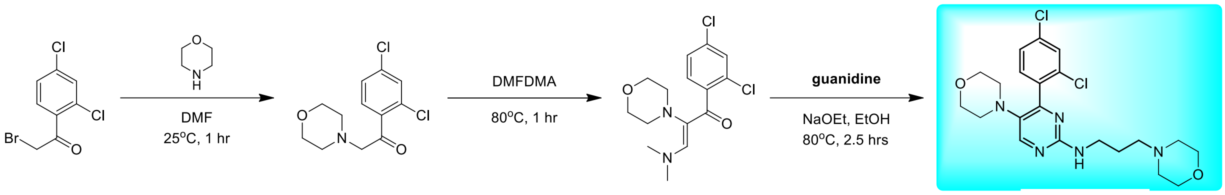

1 **2** **3**

Analog 54

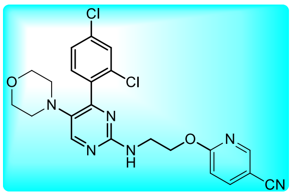

Analog 82

4-(2,4-dichlorophenyl)-5-morpholino-*N*-(3-morpholinopropyl)pyrimidin-2-amine (Analog 54). **Compound 1** (2 g, 8.95 mmol) in DMF (5 mL) was added drop wised to a stirred solution of morpholine (7.80 g, 89.49 mmol, 7.88 mL) in DMF (5 mL) at 20°C, then the mixture was stirred at 20°C for 14 hrs. LC-MS showed **Compound 1** was consumed completely and one main peak with desired mass was detected. The reaction mixture was concentrated under reduced pressure to remove solvent. The residue was diluted with H_2O 20 mL and extracted with EtOAc 10 mL × 3. The combined organic layers were washed with brine 20 mL, dried over Na_2SO_4, filtered and the filtrate was concentrated under reduced pressure to give a residue. The residue was purified by flash silica gel chromatography (ISCO®; 12 g SepaFlash® Silica Flash Column, Eluent of 0~40% Ethyl acetate/Petroleum ether gradient @ 75 mL/min) (SiO_2, Petroleum ether: Ethyl acetate=1:1, P1Rf=0.25) to afford ***Compound 2*** (1.5 g, 5.47 mmol, 61.14% yield) as a yellow oil. MS-ESI (m/z) calcd for $C_{12}H_{13}NO_2Cl_2$ $[M+H]^+$: 274.0/276.0. Found 274.0/276.0. **Compound 2** (200 mg, 729.55 umol) was dissolved in DMFDMA (2 mL) at 20°C, and the mixture was stirred at 80°C for 1 hr. LC-MS showed **Compound 2** was consumed completely and ~22% of desired product was detected. The reaction mixture was concentrated under reduced pressure to afford ***Compound 3*** (220 mg, crude) as a brown oil. MS-ESI (m/z) calcd for $C_{15}H_{18}N_2O_2Cl_2$ $[M+H]^+$: 329.1/331.1. Found 329.0/331.0. To a solution of **Compound 3A** (148.83 mg, 668.24 umol, HCl salt) in DMF (2 mL) was added Cs_2CO_3 (653.18 mg, 2.00 mmol) and **Compound 3** (220 mg, 668.24 umol) at 20°C. The mixture was stirred at 80°C for 1 hr. LC-MS showed ~8% of **Compound 3** was remained and ~5% of desired compound was detected. So the mixture was stirred at 80°C for another 12 hrs, and LC-MS showed Reactant 1 was consumed completely and ~13% of desired compound was detected. The reaction mixture was worked up together with another batch. The combined mixture was filtered to remove the insoluble, and the filtrate was concentrated under reduced pressure to give a residue. The residue was purified by prep-HPLC (HCl condition; column: Phenomenex luna C18 80*40mm*3 um; mobile phase: [water (0.04%HCl)-ACN]; B%: 22%-44%, 7min). But LC-MS show the purity of the product is only 94%, so the residue was purified by prep-HPLC twice (neutral condition, column: Phenomenex Gemini-NX C18 75*30mm*3um; mobile phase: [water (0.05%NH_3H_2O+10mM NH_4HCO_3)-ACN]; B%: 30%-60%, 8min) to afford ***54*** (16.5 mg, 99.06% purity) as a pale yellow solid. **^{1}H NMR** (METHANOL-d_4 400MHz) δ ppm 8.23 (s, 1 H) 7.58 (s, 1 H) 7.43 (s, 2 H) 3.66-3.68 (m, 4 H) 3.48-3.50 (m, 4 H) 3.30-3.32 (m, 2 H) 2.75 - 2.77 (m, 4 H) 2.43-2.47 (m, 6 H) 1.77 - 1.84 (m, 2 H). **LCMS** (ESI+): *m/z* 452.1/454.1 (M+H).

Analog 55

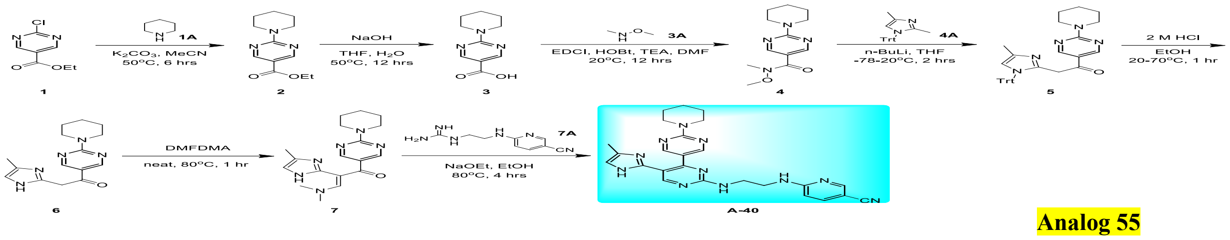

Analog 55

4-(2,4-dichlorophenyl)-5-(1*H*-imidazol-1-yl)-*N*-(3-morpholinopropyl)pyrimidin-2-amine (Analog 55). To a solution of **Compound 1** (2 g, 8.95 mmol) in ACN (20 mL) was added K_2CO_3 (1.48 g, 10.74 mmol) and imidazole (731.08 mg, 10.74 mmol). The mixture was stirred at 80°C for 4 hrs. LC-MS showed **Compound 1** was consumed completely and one main peak with desired mass was detected. The reaction mixture was concentrated under reduced pressure to remove solvent. The residue was diluted with H_2O 20 mL and extracted with ethyl acetate (5 mL × 3). The combined organic layers were dried over Na_2SO_4, filtered and concentrated under reduced pressure to give a residue. The residue was purified by flash silica gel chromatography (ISCO®; 20 g SepaFlash® Silica Flash Column, Eluent of 0~80% Ethyl acetate/Petroleum ethergradient @ 75 mL/min) (Petroleum ether/Ethyl acetate=0/1, P1Rf=0.15) to afford **Compound 2** (1.5 g, 5.47 mmol, 61.11% yield, 93% purity) as a brown gum. MS-ESI (m/z) calcd for $C_{11}H_8N_2OCl_2$ $[M+H]^+$: 255.0/257.0. Found 254.9/256.8. A mixture of **Compound 2** (150 mg, 588.01 umol) in DMFDMA (2 mL) was stirred at 80 °C for 1 hr under N_2 atmosphere. LC-MS showed **Compound 2** was consumed completely and one main peak with desired mass was detected. The reaction mixture was concentrated under reduced pressure to remove solvent to afford **Compound 3** (180 mg, crude) as a brown gum. MS-ESI (m/z) calcd for $C_{14}H_{13}N_3OCl_2$ $[M+H]^+$: 310.0/312.0. Found 309.9/311.8. To a solution of **Compound 3a** (108.09 mg, 580.31 umol) in EtOH (3 mL) was added NaOEt (78.98 mg, 1.16 mmol) and the mixture was stirred at 80°C for 0.5 hr, then **Compound 3** (180 mg, 580.31 umol) was added and the resulting mixture was stirred at 80°C for 1 hr. LC-MS showed **Compound 3** was consumed completely and one main peak with desired mass was detected. The reaction mixture was concentrated under reduced pressure to remove solvent. The residue was purified by prep-HPLC (basic condition) (column: Waters Xbridge Prep OBD C18 150*40mm*10um; mobile phase: [water (0.05%NH_3H_2O+10mM NH_4HCO_3)-ACN]; B%: 15%-50%, 8min) to afford ***55*** (36 mg, 83.08 umol, 14.32% yield, 100% purity) as a white solid. **^{1}H NMR** (METHANOL-d_4 400MHz) δ ppm 8.40 (br s, 1 H) 7.57 (s, 1 H) 7.48 (s, 1 H) 7.37 (d, J=1.00 Hz, 2 H) 6.98 (t, J=1.19 Hz, 1 H) 6.93 (s, 1 H) 3.67 (br s, 4 H) 3.48 (br s, 2 H) 2.47 (br s, 6 H) 1.85 (quin, J=6.91 Hz, 2 H). **LCMS** (ESI+): *m/z* 433.1/435.1 (M+H)

Analog 56, 12

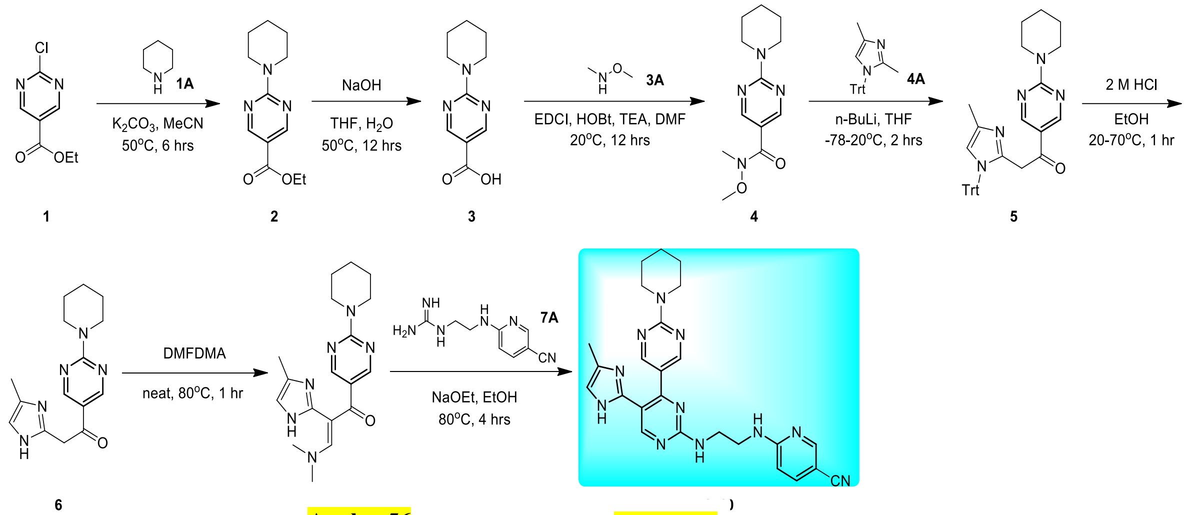

Analog 56 Analog 12

5-(4-methylpiperazin-1-yl)-*N*-(3-morpholinopropyl)-4-(4-(piperidin-1-yl)phenyl)-pyrimidin-2-amine (Analog 56). To a solution of **Compound 2** (2.5 g, 12.30 mmol) in conc.H_2SO_4 (12 mL, 98% purity) was added Br_2 (2.02 g, 12.61 mmol, 0.65 mL) at 0°C, then the temperature was warmed to 20°C slowly, then the mixture was stirred at 20°C for 12 hrs. TLC (Petroleum ether: Ethyl acetate= 5:1) (P1 Rf=0.51) showed **Compound 2** was remained and two new spots were formed, then Br_2 (465.00 mg, 2.91 mmol, 0.15 mL) was added at 0°C, then the mixture was stirred at 20°C for another 3.5 hrs. TLC (Petroleum ether: Ethyl acetate= 5:1) (P1 Rf=0.51) showed **Compound 2** was consumed and two new spots were formed. The reaction was poured into ice-water slowly and the temperature was maintained at 0°C, solid was appeared, the mixture was filtered and the filter-cake was dried under vacuum to afford ***Compound 3*** (3.41 g, 7.65 mmol, 62.20% yield, 81% purity) as a yellow solid. MS-ESI (m/z) calcd for $C_{13}H_{15}Br_2NO$ $[M+H]^+$: 360.0/362.0/364.0. Found 359.9/361.9/363.9. To a solution of **Compound 3** (3.41 g, 9.44 mmol) in THF (17 mL) was added another solution of **Compound 3A** (1.37 g, 9.92 mmol, 1.28 mL) and TEA (1.00 g, 9.92 mmol, 1.38 mL) in THF (9 mL) at 0°C, then the mixture was stirred at 20°C for 12 hrs. LCMS showed **Compound 3** was consumed and 57% of desired product was detected. The reaction was concentrated under vacuum. The residue was poured into ice-water, solid was appeared, then the mixture was filtered and the filter-cake was dried under vacuum to afford ***Compound 4*** (1.93 g, 6.84 mmol, 72.42% yield) as yellow solid. MS-ESI (m/z) calcd for $C_{13}H_{16}BrNO$ $[M+H]^+$: 282.1/284.1. Found 281.9/283.9. To a solution of **Compound 4** (1 g, 3.54 mmol) in DMF (5 mL) was added **Compound 4A** (3.55 g, 35.44 mmol, 3.93 mL) at 20°C, then the mixture was stirred at 20°C for 3 hrs. TLC (plate 1) (Petroleum ether: Ethyl acetate=5:1) (P1 Rf=0.00) showed **Compound 4** was consumed and two spots were formed. The reaction was concentrated under vacuum. The residue was purified by flash silica gel chromatography (ISCO®; 20 g SepaFlash® Silica Flash Column, Eluent of 0~70% Ethyl acetate/Petroleum ether gradient @ 75 mL/min) (plate 1) (Dichloromethane: Methanol=10:1) (P1 Rf=0.19) to afford ***Compound 5*** (0.64 g, 2.12 mmol, 59.91% yield) as a yellow solid. MS-ESI (m/z) calcd for $C_{18}H_{27}N_3O$ $[M+H]^+$: 302.2. Found 302.2. A solution of **Compound 5** (100 mg, 331.76 umol) in DMFDMA (2 mL) was stirred at 80°C for 6 hrs. LCMS showed 13% of reactant 1 was remained and 2% of desired product was detected. Then the reaction was stirred at 80°C for 12 hrs. LCMS showed 11% of **Compound 5** was remained and 7% of desired product was detected. Then the mixture was concentrated under vacuum. The residue was purified by Prep-TLC (Dichloromethane: Methanol=10:1) (P1 RF=0.19) to afford ***Compound 6*** (34 mg, 95.37 umol, 28.75% yield) as a yellow liquid. MS-ESI (m/z) calcd for $C_{21}H_{32}N_4O$ $[M+H]^+$: 357.3. Found 357.2. To a solution of **guanidine** (21.42 mg, 115.01

umol) and EtONa (46.96 mg, 690.03 umol, 6 eq) in EtOH (1 mL) was stirred at 80°C for 15 min, then another solution of **Compound 6** (41 mg, 115.01 umol) in EtOH(1 mL) was added at 80°C, then the mixture was stirred at 100°C for 12 hrs under microwave. LCMS showed 21% **Compound 6** was remained and 15% of desired product was detected. The reaction was combined with another batch. The mixture was filtered. The filtrate was purified by Prep-HPLC (TFA condition) (column: Phenomenex Luna C18 100*30mm*5um; mobile phase: [water (0.1%TFA)-ACN]; B%: 1%-18%, 10 min) to afford ***56*** (17.30 mg, 29.14 umol, 14.9% yield, 98.25% purity) as a yellow gum. **^{1}H NMR** (D_2O 400MHz) δ ppm 8.16 - 8.22 (m, 3 H) 7.75 (d, J=8.78 Hz, 2 H) 4.04 (br d, J=12.67 Hz, 2 H) 3.65 - 3.75 (m, 6 H) 3.58 (t, J=6.65 Hz, 2 H) 3.40 - 3.51 (m, 4 H) 3.21 - 3.28 (m, 2 H) 2.97 - 3.17 (m, 8 H) 2.86 (s, 3 H) 1.99 - 2.12 (m, 6 H) 1.77 (br d, J=5.40 Hz, 2 H). **LCMS** (ESI+): *m/z* 480.2 (M+H)

Analog 59

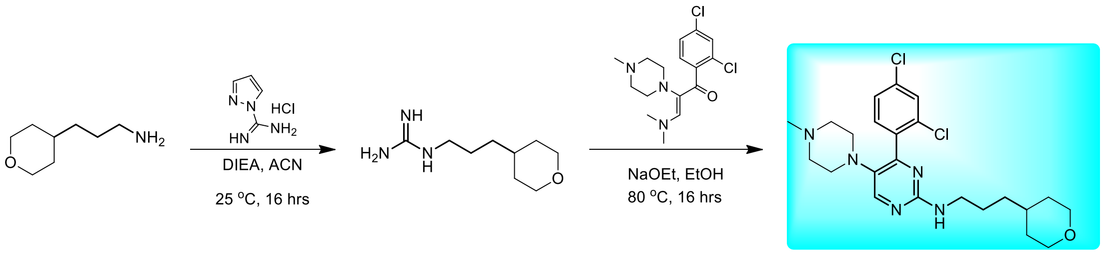

1 2 **Analog 59**

4-(2,4-dichlorophenyl)-5-(4-methylpiperazin-1-yl)-*N*-(3-(tetrahydro-2*H*-pyran-4-yl)propyl)pyrimidin-2-amine (Analog 59). To a mixture of **compound 1** (0.21 g, 1.47 mmol, 1 *eq*) in MeCN (3 mL) was added DIEA (378.99 mg, 2.93 mmol, 510.76 uL, 2 *eq*) pyrazole-1-carboxamidine (257.90 mg, 1.76 mmol, 1.2 *eq*, HCl) at 25 °C, the mixture was stirred at 25 °C for 16 h. LCMS showed the mixture was completed. The mixture was concentrated to give **compound 2** (0.2 g, crude) as colour less oil. MS-ESI (m/z) calcd for C9H19N3O $[M+H]^+$: 186.0. To a mixture of **compound 2** (135.32 mg, 730.43 umol, 2.5 *eq*) (Z)-1-(2,4-dichlorophenyl)-3-(dimethylamino)-2-(4-methylpiperazin-1-yl)prop-2-en-1-one (0.1 g, 292.17 umol, 1 *eq*) in EtOH (5 mL) was added NaOEt (59.65 mg, 876.52 umol, 3 *eq*) at 25 °C, the mixture was stirred at 80 °C for 16 h. LCMS showed the peak with desired MS. The mixture was concentrated to give residue, to the residue was added H2O (10 mL) and DCM (10 mL). The aqueous phase was extracted with DCM (10 mL*2). The combined organic phase was washed with brine (10 mL*2), dried with anhydrous Na_2SO_4, filtered and concentrated in vacuum. The residue was purified by prep-HPLC: column: Waters Xbridge Prep OBD C18 150*40mm*10um;mobile phase: [water(10mM NH4HCO3)-ACN];B%: 50%-70%,8min to give compound **59** (28.29 mg, 60.49 umol, 20.70% yield, 99.3% purity) as a yellow solid. **^{1}H NMR** (METHANOL-d_4 400MHz) δ ppm 8.22 (s, 1 H) 7.57 (d, *J*=1.47 Hz, 1 H) 7.38 - 7.47 (m, 2 H) 3.89 (dd, *J*=11.19, 3.97 Hz, 2 H) 3.33 - 3.40 (m, 4 H) 2.81 (t, *J*=4.77 Hz, 4 H) 2.17 - 2.41 (m, 7 H) 1.58 - 1.69 (m, 4 H) 1.47 - 1.57 (m, 1 H) 1.28 - 1.35 (m, 2 H) 1.16 - 1.27 (m, 2 H). **LCMS** (ESI+): *m/z* 464.2/466.2 (M+H)

Analog 63, 62

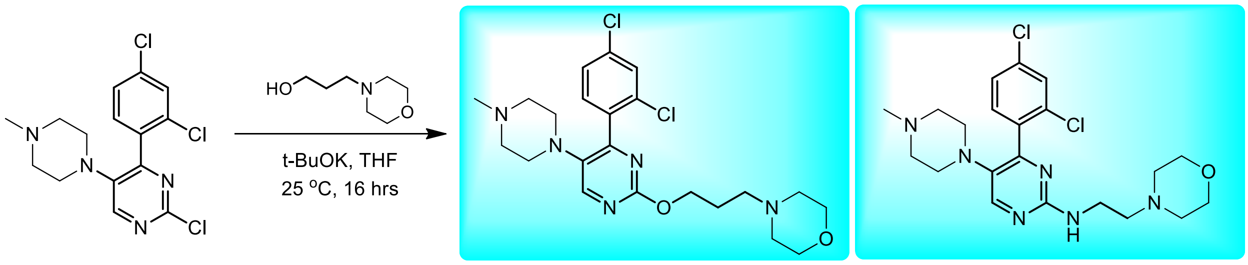

1

Analog 63 **Analog 62**

4-(3-((4-(2,4-dichlorophenyl)-5-(4-methylpiperazin-1-yl)pyrimidin-2-yl)oxy)propyl)-morpholine (Analog 63). To a mixture of **compound** 1 (0.03 g, 83.88 umol, 1 *eq*) 3-morpholinopropan-1-ol (24.36 mg, 167.75 umol, 2 *eq*) in THF (1 mL) was added t-BuOK (1 M, 251.63 uL, 3 *eq*) at 25 °C, the mixture was stirred at 25 °C for 16 h. LCMS showed the mixture was completed. The mixture was concentrated to give residue. The residue was purified by prep-HPLC: column: Phenomenex Gemini-NX C18 75*30mm*3um;mobile phase: [water(0.05%NH3H2O+10mM NH4HCO3)-ACN];B%: 30%-60%,8min to give **63** (22.05 mg, 47.16 umol, 56.23% yield, 99.76% purity) as a yellow solid.

A similar procedure was used to make **4-(2,4-dichlorophenyl)-5-(4-methylpiperazin-1-yl)-*N*-(2-morpholinoethyl)pyrimidin-2-amine (Analog 62)**. **^{1}H NMR** (METHANOL-d_4 400MHz) δ ppm 8.46 (s, 1 H) 7.63 (s, 1 H) 7.49 (s, 2 H) 4.42 (br t, *J*=6.28 Hz, 2 H) 3.69 (br t, *J*=4.30 Hz, 4 H) 2.90 (br t, *J*=4.30 Hz, 4 H) 2.56 - 2.62 (m, 2 H) 2.53 (br s, 4 H) 2.41 (br s, 4 H) 2.29 (s, 3 H) 1.96 - 2.09 (m, 2 H). **LCMS** (ESI+): *m/z* 466.2/468.2 (M+H)

Analog 65

3-((4-(2,4-dichlorophenyl)-5-(4-methylpiperazin-1-yl)pyrimidin-2-yl)amino)-1-

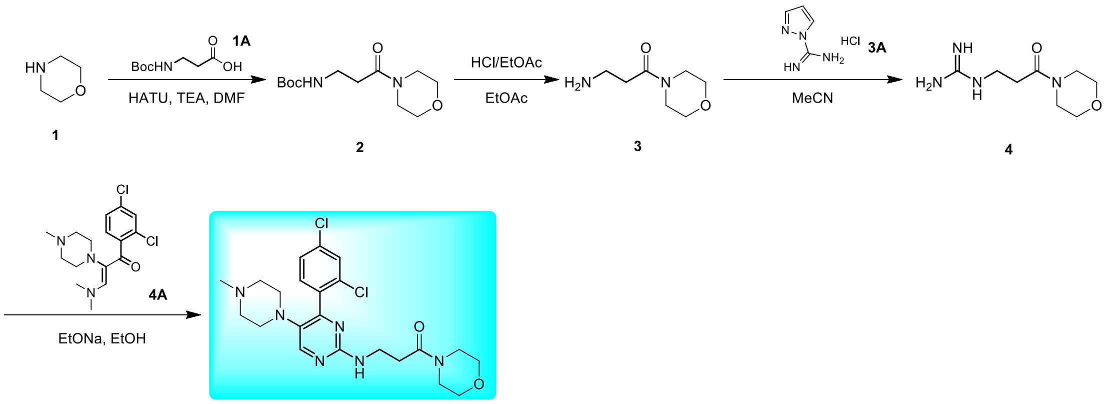

Analog 65

morpholinopropan-1-one (Analog 65). To a solution of **Compound 1A** (2.23 g, 11.79 mmol) and **Compound 1** (1.03 g, 11.79 mmol, 1.04 mL) in DMF (20 mL) were added HATU (4.48 g, 11.79 mmol) and TEA (1.25 g, 12.38 mmol, 1.72 mL) at 20°C, then the mixture was stirred at 20°C for 12 hrs. LCMS showed **Compound 1** was consumed and 16% of desired product was detected. The reaction was concentrated under vacuum to afford ***Compound 2*** (4 g, crude) as a yellow liquid and it was used directly. MS-ESI (m/z) calcd for $C_{12}H_{22}N_2O_4$ $[M+H]^+$: 259.2 Found 259.1. To a solution of **Compound 2** (100 mg, 387.35 umol) in EtOAc (3 mL) was added HCl/EtOAc (4 M, 3 mL). The mixture was stirred at 20 °C for 1 hr. TLC showed **Compound 2** was consumed completely and desired mass was detected. The mixture was concentrated under reduced pressure to afford ***Compound 3*** (80 mg, crude, HCl) as a white solid. A mixture of **Compound 3** (0.2 g, 1.26 mmol, HCl), **Compound 3A** (244.72 mg, 1.67 mmol, HCl) in MeCN (3 mL) was stirred at 100 °C for 3 hrs. LCMS showed the mixture was completed. The mixture was concentrated to give residue. The residue was purified by Prep-HPLC (HCl condition) (column: Phenomenex Luna C18 100*30mm*5um; mobile phase: [water (0.04% HCl) - ACN]; B%: 1%-30%, 10 min) to afford ***Compound 4*** (51 mg, 215.46 umol, 8.63% yield, HCl) as a colourless oil. MS-ESI (m/z) calcd for $C_8H_{16}N_4O_2$ $[M+H]^+$: 201.1 Found 201.2. To a solution of **Compound 4** (58.50 mg, 292.17 umol) in EtOH (3 mL) was added EtONa (59.65 mg, 876.52 umol) at 20°C, then the mixture was stirred at 80°C for 15 min, then another solution of **Compound 4A** (100 mg, 292.17 umol) in EtOH (2 mL) was added at 80°C, then the mixture was stirred at 80°C for 8 hrs under microwave. LCMS showed no reaction. The reaction was stirred at 80 °C for 5 hrs. LCMS showed 57% **Compound 4A** was remained, 14% of desired product was detected. The reaction was stirred at 80 °C for 10 hrs. LCMS showed 55% **Compound 4A** was remained, 15% of desired product was detected. The reaction was stirred at 100 °C for 2 hrs. LCMS showed 51% **Compound 4A** was remained, 16% of desired product was detected. The reaction was filtered. The crude product was purified by Prep-HPLC (TFA condition) (column: Phenomenex Gemini-NX 150*30mm*5um; mobile phase: [water (0.1% TFA) - ACN]; B%: 10%-40%, 9 min), the residue was purified by Prep-HPLC (neutral condition) (column: Waters Xbridge BEH C18 100*25mm*5um; mobile phase: [water (10mM NH_4HCO_3) - ACN]; B%: 20%-60%, 10 min) to afford ***65*** (4.47 mg, 7.53 umol, 12.04% yield, 100% purity, TFA) as a pale yellow gum. **^{1}H NMR**

(METHANOL-*d*4 400MHz) *δ* ppm 8.26 (s, 1 H) 7.59 (d, J=1.22 Hz, 1 H) 7.39 - 7.48 (m, 2 H) 3.58 - 3.64 (m, 4 H) 3.50 - 3.56 (m, 6 H) 2.81 (t, J=4.65 Hz, 4 H) 2.69 (t, J=6.91 Hz, 2 H) 2.23 - 2.37 (m, 4 H) 2.22 (s, 3 H) **LCMS** (ESI+): *m*/*z* 479.1/481.1(M+H)

Analog 67

THE END

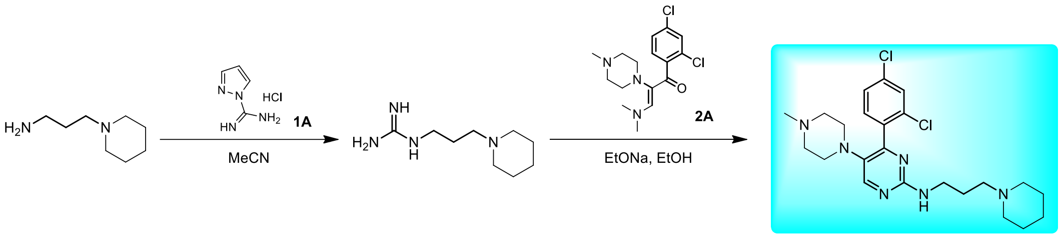

1 2 A-79

Analog 67

4-(2,4-dichlorophenyl)-5-(4-methylpiperazin-1-yl)-*N*-(3-(piperidin-1-yl)propyl)pyrimidin-2-amine (Analog 67). To a solution of **Compound 1** (100 mg, 703.03 umol) in MeCN (3 mL) was added **Compound 1A** (103.05 mg, 703.03 umol) at 20°C, then the mixture was stirred at 80°C for 12 hrs. LCMS showed **Compound 1** was consumed and a main peak with desired product was detected. The reaction was concentrated under vacuum to afford ***Compound 2*** (160 mg, crude, HCl) as a pale yellow liquid and it was used directly. MS-ESI (m/z) calcd for $C_9H_{20}N_4$ $[M+H]^+$: 185.2 Found 185.2. To a solution of **Compound 2** (77.39 mg, 350.61 umol, HCl) in EtOH (2 mL) was added EtONa (59.65 mg, 876.52 umol) at 20°C, then the mixture was stirred at 80°C for 15 min, then another solution of **Compound 2A** (100 mg, 292.17 umol) in EtOH (2 mL) was added at 80°C, then the mixture was stirred at 80°C for 12 hrs. LCMS showed 55% of **Compound 2A** was consumed and 16% of desired product was detected. The reaction was concentrated under vacuum. The residue was purified by Prep-HPLC (basic condition) (column: Phenomenex Gemini-NX C18 75*30mm*3um; mobile phase: [water (0.05% NH_3H_2O + 10mM NH_4HCO_3) - ACN]; B%: 40%-80%, 8 min) to afford ***67*** (35.08 mg, 73.09 umol, 25.02% yield, 96.56% purity) as a pale yellow solid. **^{1}H NMR** (METHANOL-*d*4 400MHz) *δ* ppm 8.22 (s, 1 H) 7.57 (s, 1 H) 7.40 - 7.46 (m, 2 H) 3.37 (t, J=6.84 Hz, 2 H) 2.81 (t, J=4.71 Hz, 4 H) 2.35 - 2.48 (m, 6 H) 2.23 - 2.35 (m, 4 H) 2.21 (s, 3 H) 1.81 (quin, J=7.37 Hz, 2 H) 1.59 (dt, J=11.17, 5.58 Hz, 4 H) 1.46 (br d, J=4.77 Hz, 2 H). **LCMS** (ESI+): *m*/*z* 463.2/465.2(M+H)

Analog 68

THE EN

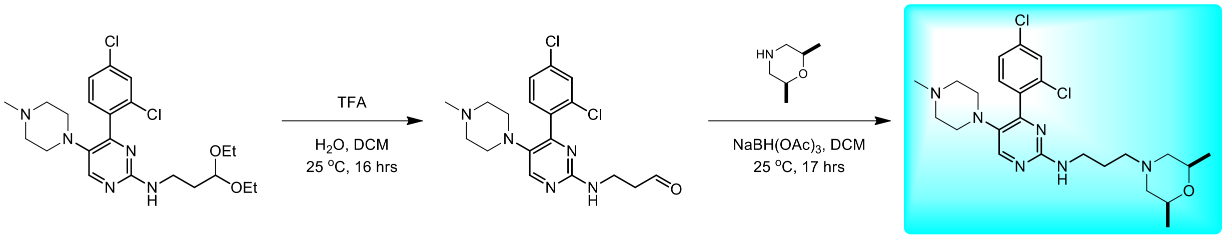

1 2

Analog 68

4-(2,4-dichlorophenyl)-*N*-(3-((2*S*,6*R*)-2,6-dimethylmorpholino)propyl)-5-(4-methylpiperazin-1-yl)pyrimidin-2-amine (Analog 68). To a mixture of **compound 1** (0.1 g, 213.48 umol, 1 *eq*) in DCM (5 mL) was added H2O (300.00 mg, 16.65 mmol, 0.3 mL, 77.98 *eq*) TFA (924.00 mg, 8.10 mmol, 0.6 mL, 37.96 *eq*) at 25 °C, the mixture was stirred at 25 °C for 16 h. LCMS showed the mixture was completed. The mixture was concentrated to give residue, the residue was in sat.$NaHCO_3$ (5 mL) and extracted with DCM (10 mL*3). The combined organic phase was washed with brine (10 mL*2), dried with anhydrous Na_2SO_4, filtered and concentrated in vacuum to give **compound 2** (0.08 g, crude) as yellow oil and used directly. MS-ESI (m/z) calcd for C18H21Cl2N5O $[M+H]^+$: 394.1/396.1. To a mixture of **compound 2** (0.08 g, 202.89 umol, 1 *eq*) in DCM (3 mL) was added AcOH (1.22 mg, 20.29 umol, 1.16 uL, 0.1 *eq*) and (2S,6R)-2,6-dimethylmorpholine (23.37 mg, 202.89 umol, 1 *eq*) at 25 °C, the mixture was stirred at 25 °C for 1 h, then $NaBH(OAc)_3$ (215.01 mg, 1.01 mmol, 5 *eq*) was added at 25 °C and stirred at 25 °C for another 16 h. LCMS showed desired MS. The mixture was concentrated to give residue, to the residue was added sat.$NaHCO_3$ (20 mL). The aqueous phase was extracted with DCM (20 mL*2). The combined organic phase was washed with brine (20 mL*2), dried with anhydrous Na_2SO_4, filtered and concentrated in vacuum. The residue was purified by prep-HPLC: column: Waters Xbridge BEH C18 100*30mm*10um;mobile phase: [water(10mM NH4HCO3)-ACN];B%:35%-65%,10min to give **68** (7.14 mg, 14.33 umol, 7.06% yield, 99.03% purity) as a white solid. **^{1}H NMR** (METHANOL-d_4 400MHz) δ ppm 8.24 (s, 1H) 7.58 (d, J=1.22 Hz, 1H) 7.36 - 7.50 (m, 2H) 3.59 - 3.74 (m, 2H) 3.40 (t, J=6.85 Hz, 2H) 2.79 - 2.85 (m, 6H) 2.41 - 2.48 (m, 2H) 2.33 (br d, J=10.64 Hz, 4H) 2.24 (s, 3H) 1.82 (quin, J=7.12 Hz, 2H) 1.69 (t, J=10.94 Hz, 2H) 1.11 (d, J=6.36 Hz, 6H) **LCMS** (ESI+): m/z 493.2/495.2 (M+H).

Analog 69

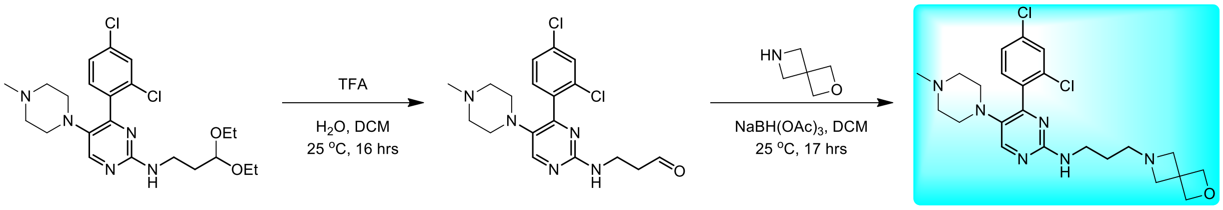

1 2

Analog 69

***N*-(3-(2-oxa-6-azaspiro[3.3]heptan-6-yl)propyl)-4-(2,4-dichlorophenyl)-5-(4-methylpiperazin-1-yl)pyrimidin-2-amine (Analog 69)** To a mixture of **compound 1** (0.3 g, 640.45 umol, 1 *eq*) in DCM (10 mL) was added H2O (900.00 mg, 49.94 mmol, 900.00 uL, 77.98 *eq*) TFA (2.77 g, 24.31 mmol, 1.80 mL, 37.96 *eq*) at 25 °C, the mixture was stirred at 25 °C for 16 h. TLC (dichloromethane:

methanol=10:1, R_f = 0.27) showed the mixture was completed. The mixture was concentrated to give residue. To the residue was added sat.NaHCO3 (50 mL) and DCM (50 mL), the aqueous phase was extracted with DCM (50 mL*2). The combined organic phase was washed with brine (50 mL*2), dried with anhydrous Na_2SO_4, filtered and concentrated in vacuum. The crude product was used next step directly. **Compound 2** (0.3 g, crude) as yellow oil. MS-ESI (m/z) calcd for C18H21Cl2N5O $[M+H]^+$: 394.1/396.1 To a mixture of **compound 2** (0.1 g, 253.62 umol, 1 *eq*) 2-oxa-6-azaspiro[3.3]heptane (125.71 mg, 1.27 mmol, 5 *eq*) in DCM (3 mL) was added AcOH (1.52 mg, 25.36 umol, 1.45 uL, 0.1 *eq*) at 25 °C, the mixture was stirred at 25 °C for 1 h, then $NaBH(OAc)_3$ (161.25 mg, 760.85 umol, 3 *eq*) was added and stirred at 25 °C for another 16 h. LCMS showed the mixture was completed. The mixture was added to sat.NaHCO3 (20 mL) and the aqueous phase was extracted with DCM (50 mL*2). The combined organic phase was washed with brine (50 mL*2), dried with anhydrous Na_2SO_4, filtered and concentrated in vacuum. The residue was purified by prep-HPLC: column: Waters Xbridge Prep OBD C18 150*40mm*10um;mobile phase: [water(10mM NH4HCO3)-ACN];B%: 25%-45%,8min to give **69** (15.3 mg, 29.45 umol, 11.61% yield, 91.9% purity) as a yellow solid. **^{1}H NMR** (METHANOL-*d_4* 400MHz) δ ppm 8.22 (s, 1 H) 7.58 (d, *J*=1.59 Hz, 1 H) 7.39 - 7.46 (m, 2 H) 4.71 (s, 4 H) 3.38 (s, 4 H) 3.35 (t, *J*=6.91 Hz, 2 H) 2.81 (t, *J*=4.71 Hz, 4 H) 2.43 - 2.54 (m, 2 H) 2.19 - 2.37 (m, 7 H) 1.63 (quin, *J*=7.18 Hz, 2 H). **LCMS** (ESI+): *m/z* 477.2/479.2 (M+H)

Analog 70

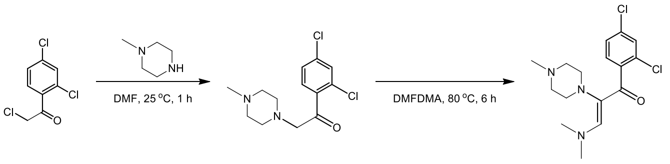

1A 2A 3A

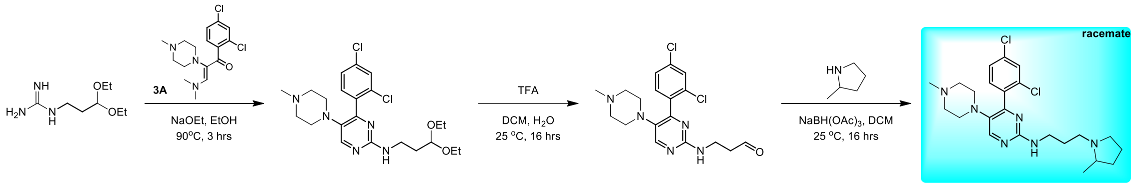

1 2 3 Analog 70

4-(2,4-dichlorophenyl)-5-(4-methylpiperazin-1-yl)-*N*-(3-(2-methylpyrrolidin-1-yl)propyl)pyrimidin-2-amine (Analog 70). To a mixture of **compound 1A** (8 g, 35.80 mmol, 1 *eq*) and 1-methylpiperazine (35.85 g, 357.97 mmol, 39.71 mL, 10 *eq*) in DMF (80 mL). The mixture was stirred at 25°C for 1 hour. TLC (petroleum ether: Ethyl acetate=10:1, R_f=0.6) indicated compound 1A was consumed completely and **one new spot** formed. The mixture was poured into water and extracted with ethyl acetate (200 mL*3). The combined organic phase was washed with brine (300 mL*2), dried with anhydrous Na_2SO_4, filtered and concentrated in vacuum. The residue was purified by column chromatography (SiO2, **Petroleum ether/Ethyl acetate=100/1** to **0/1**) to give **compound 2A** (9 g, 31.34 mmol, 87.55% yield) was obtained as a yellow oil. The structure was confirmed by HNMR (ET39965-6-P1A). 1H NMR (400MHz, CHLOROFORM-d) δ = 7.48 - 7.43 (m, 2H), 7.34 - 7.29 (m, 1H), 3.74 (s, 2H), 2.75 - 2.40 (m, 8H), 2.34 (s, 3H). The mixture of **compound 2A** (7.8 g, 27.16 mmol, 1 *eq*) in DMF-DMA (20 mL) was stirred at 80 °C for 6 hours. LCMS showed the desired ms was detected. The mixture was concentrated. The crude product was triturated with **MTBE, filtered. Compound 3A** (3.5 g, 10.23 mmol, 37.65% yield) was obtained as a yellow solid. The structure was confirmed by

HNMR (ET39965-19-P1A) **1H NMR (400MHz, CHLOROFORM-d)** δ = 7.35 (d, J=1.6 Hz, 1H), 7.25 - 7.20 (m, 1H), 7.19 - 7.14 (m, 1H), 6.33 (br s, 1H), 3.69 - 2.78 (m, 10H), 2.66 - 2.41 (m, 4H), 2.35 (br s, 3H)

MS-ESI (m/z) calcd for C16H21Cl2N3O $[M+H]^+$: 342.0/344.0. **Compound 3**A (1 g, 2.92 mmol, 1 *eq*), **compound 1** (1.11 g, 5.84 mmol, 2 *eq*) and NaOEt (596.47 mg, 8.77 mmol, 3 *eq*) were in EtOH (10 mL). The sealed tube was heated at 90 °C for 3 h. TLC (dichloromethane: methanol=10:1, R_f = 0.16) showed the mixture was completed. The mixture was concentrated to remove EtOH, to the residue was added H2O (50 mL) and DCM (50 mL), the aqueous phase was extracted with DCM (50 mL*2). The combined organic phase was washed with brine (50 mL*2), dried with anhydrous Na_2SO_4, filtered and concentrated in vacuum. The residue was purified by silica gel chromatography (column height: 250 mm, diameter: 100 mm, 100-200 mesh silica gel, EtOAc/EtOH = 1/0, 10/1) to give product compound 2 (0.68 g, 1.45 mmol, 49.69% yield) as yellow oil **^{1}H NMR (400 MHz, CHLOROFORM-*d*)** δ ppm 8.24 (s, 1 H) 7.48 (s, 1 H) 7.33 (s, 2 H) 5.36 (br s, 1 H) 4.64 (t, *J*=5.50 Hz, 1 H) 3.68 (dq, *J*=9.32, 7.04 Hz, 2 H) 3.48 - 3.55 (m, 4 H) 2.76 - 3.05 (m, 4 H) 2.27 - 2.54 (m, 6 H) 1.95 (q, *J*=6.44 Hz, 2 H) 1.21 (t, *J*=7.03 Hz, 6 H) To a mixture of **compound 2** (0.3 g, 640.45 umol, 1 *eq*) in DCM (5 mL) was added H2O (900.00 mg, 49.94 mmol, 900.00 uL, 77.98 *eq*) TFA (2.77 g, 24.31 mmol, 1.80 mL, 37.96 *eq*) at 25 °C, the mixture was stirred at 25 °C for 16 h. LCMS showed the mixture was completed. The mixture was concentrated to give residue, to the residue was added NaHCO3 (50 mL) and DCM (50 mL), the aqueous phase was extracted with DCM (20 mL*2). The combined organic phase was washed with brine (50 mL*2), dried with anhydrous Na_2SO_4, filtered and concentrated in vacuum. The residue was used next step directly. **Compound 3** (0.27 g, crude) as yellow oil. MS-ESI (m/z) calcd for C18H21Cl2N5O $[M+H]^+$: 394.05/396.05. To a mixture of **compound 3** (0.1 g, 253.62 umol, 1 *eq*) 2-methylpyrrolidine (107.97 mg, 1.27 mmol, 129.46 uL, 5 *eq*) in DCM (3 mL) was added AcOH (1.52 mg, 25.36 umol, 1.45 uL, 0.1 *eq*) NaBH(OAc)3 (161.25 mg, 760.85 umol, 3 *eq*) was added and stirred at 25 °C for 16 h. LCMS showed the mixture was completed. The mixture was added to sat.NaHCO3 (20 mL) and aqueous phase was extracted with DCM (20 mL*2). The combined organic phase was washed with brine (20 mL), dried with anhydrous Na_2SO_4, filtered and concentrated in vacuum. The residue was purified by prep-HPLC: column: Waters Xbridge BEH C18 100*30mm*10um;mobile phase: [water(10mM NH4HCO3)-ACN];B%: 25%-50%,10min to give **70** (44.15 mg, 94.22 umol, 37.15% yield, 98.9% purity) as a yellow solid. **^{1}H NMR** (METHANOL-d_4 400MHz) *δ* ppm 8.23 (br s, 1 H) 7.57 (dd, *J*=1.54, 0.66 Hz, 1 H) 7.38 - 7.47 (m, 2 H) 3.35 - 3.43 (m, 2 H) 3.11 - 3.19 (m, 1 H) 2.92 (ddd, *J*=11.69, 10.14, 6.39 Hz, 1 H) 2.81 (t, *J*=4.63 Hz, 4 H) 2.23 - 2.42 (m, 5 H) 2.21 (s, 3 H) 2.10 - 2.18 (m, 2 H) 1.91 - 2.02 (m, 1 H) 1.68 - 1.89 (m, 4 H) 1.35 - 1.48 (m, 1 H) 1.10 (d, *J*=6.17 Hz, 3 H)/. **LCMS** (ESI+): *m/z* 463.2/465.2 (M+H)

Analog 71

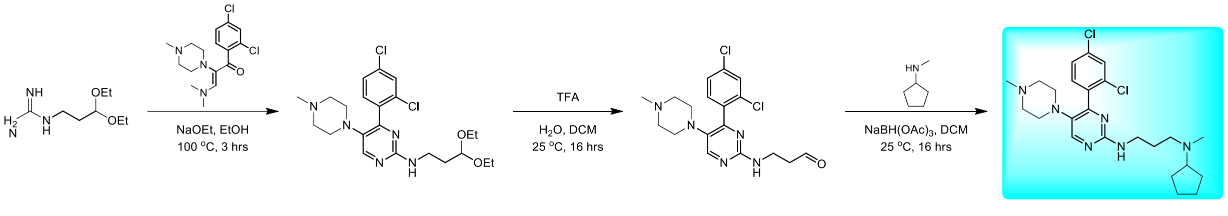

***N*[1]-cyclopentyl-*N*[3]-(4-(2,4-dichlorophenyl)-5-(4-methylpiperazin-1-yl)pyrimidin-2-yl)-*N*[1]-methylpropane-1,3-diamine (Analog 71).** (Z)-1-(2,4-dichlorophenyl)-3-(dimethylamino)-2-(4-methylpiperazin-1-yl)prop-2-en-1-one (0.3 g, 876.52 umol, 1 *eq*), **compound** 1 (165.89 mg, 876.52 umol, 1 *eq*) and NaOEt (178.94 mg, 2.63 mmol, 3 *eq*) were taken up into a microwave tube in EtOH (3 mL). The sealed tube was heated at 100 °C for 3h under microwave. TLC (dichloromethane: methanol=10:1, R_f = 0.15) showed the mixture was completed. The mixture was concentrated to remove EtOH, to the residue was added DCM (50 mL) and H_2O (50 mL), the aqueous phase was extracted with DCM (50 mL*2). The combined organic phase was washed with brine (50 mL*2), dried with anhydrous Na_2SO_4, filtered and concentrated in vacuum. The residue was purified by prep-TLC (DCM/MeOH=10/1, R_f= 0.2) to afford to give compound 2 (0.15 g, 320.23 umol, 36.53% yield) as yellow oil. To a mixture of **compound 2** (0.15 g, 320.23 umol, 1 *eq*) in DCM (10 mL) was added H2O (450.00 mg, 24.97 mmol, 450.00 uL, 77.98 *eq*) TFA (1.39 g, 12.16 mmol, 900.00 uL, 37.96 *eq*) at 25 °C, the mixture was stirred at 25 °C for 16 h. LCMS showed the mixture was completed. The mixture was concentrated to give residue, to the residue was added sat.NaHCO3 (20 mL) and DCM (20 mL), the aqueous phase was extracted with DCM (20 mL*2). The combined organic phase was washed with brine (20 mL*2), dried with anhydrous Na_2SO_4, filtered and concentrated in vacuum. The residue was used next step directly. **Compound 3** (0.15 g, crude) as yellow oil. MS-ESI (m/z) calcd for C18H21Cl2N5O $[M+H]^+$: 394.1/396.1. To a mixture of **compound 3** (0.08 g, 202.89 umol, 1 *eq*) in DCM (3 mL) was added AcOH (1.22 mg, 20.29 umol, 1.16 uL, 0.1 *eq*) N-methylcyclopentanamine (201.22 mg, 2.03 mmol, 10 *eq*) NaBH(OAc)3 (129.00 mg, 608.68 umol, 3 *eq*) at 25 °C, the mixture was stirred at 25 °C for 16 h. LCMS showed the mxture was completed. The mixture was added to sat.NaHCO3 (20 mL), aqueous phase was extracted with DCM (20 mL*2). The combined organic phase was washed with brine (50 mL), dried with anhydrous Na_2SO_4, filtered and concentrated in vacuu. The residue was purified by prep-HPLC:column: Waters Xbridge BEH C18 100*30mm*10um;mobile phase: [water(10mM NH4HCO3)-ACN];B%: 35%-65%,10min to give crude product, the crude was purified by another prep-HPLC:column: 3_Phenomenex Luna C18 75*30mm*3um;mobile phase: [water(0.1%TFA)-ACN];B%: 1%-40%,10min to give **71** (16.44 mg, 27.32 umol, 13.47% yield, 98.3% purity, TFA) as yellow oil. **^{1}H NMR** (METHANOL-d_4 400MHz) δ ppm 8.35 (s, 1 H) 7.64 (d, *J*=1.34 Hz, 1 H) 7.43 - 7.52 (m, 2 H) 3.57 - 3.67 (m, 1 H) 3.49 (t, *J*=6.60 Hz, 2 H) 3.40 (br d, *J*=11.49 Hz, 2 H) 3.36 (br d, *J*=5.62 Hz, 1 H) 3.02 - 3.16 (m, 5 H) 2.84 (d, *J*=11.98 Hz, 8 H) 1.94 - 2.16 (m, 4 H) 1.80 (br s, 2 H) 1.66 (br s, 4 H). **LCMS** (ESI+): *m/z* 477.2/479.2 (M+H)

Analog 72

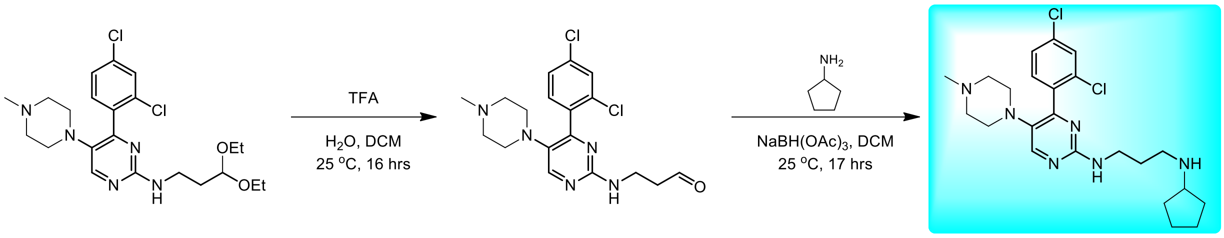

1 2 **Analog 72**

***N*[1]-cyclopentyl-*N*[3]-(4-(2,4-dichlorophenyl)-5-(4-methylpiperazin-1-yl)pyrimidin-2-yl)propane-1,3-diamine (Analog 72)**. To a mixture of **compound 1** (0.3 g, 640.45 umol, 1 *eq*) in DCM (10 mL) was added H2O (900.00 mg, 49.94 mmol, 900.00 uL, 77.98 *eq*) TFA (2.77 g, 24.31 mmol, 1.80 mL, 37.96 *eq*) at 25 °C, the mixture was stirred at 25 °C for 16 h. TLC (dichloromethane: methanol=10:1, R_f = 0.27) showed the mixture was completed. The mixture was concentrated to give residue. To the residue was added sat.NaHCO3 (50 mL) and DCM (50 mL), the aqueous phase was extracted with DCM (50 mL*2). The combined organic phase was washed with brine (50 mL*2), dried with anhydrous Na_2SO_4, filtered and concentrated in vacuum. The crude product was used next step directly. **Compound 2** (0.3 g, crude) as yellow oil. MS-ESI (m/z) calcd for C18H21Cl2N5O $[M+H]^+$: 394.1/396.1 To a mixture of **compound 2** (0.1. g, 253.62 umol, 1 *eq*) cyclopentanamine (107.97 mg, 1.27 mmol, 125.11 uL, 5 *eq*) in DCM (3 mL) was added AcOH (1.52 mg, 25.36 umol, 1.45 uL, 0.1 *eq*) at 25 °C, the mixture was stirred at 25 °C for 1 h, then $NaBH(OAc)_3$ (161.25 mg, 760.85 umol, 3 *eq*) was added and stirred at 25 °C for 16 h. LCMS showed the mixture was completed. The mixture was added to sat.NaHCO3 (20 mL), the aqueous phase was extracted with DCM (20 mL*2). The combined organic phase was washed with brine (20 mL*2), dried with anhydrous Na_2SO_4, filtered and concentrated in vacuum. The residue was purified by prep-HPLC: column: Phenomenex luna C18 100*40mm*5 um;mobile phase: [water(0.1%TFA)-ACN];B%: 15%-47%,8min to give **72** (72.78 mg, 149.31 umol, 58.87% yield, 95.08% purity) as yellow oil. **^{1}H NMR** (METHANOL-d_4 400MHz) δ ppm 8.34 (s, 1 H) 7.64 (d, *J*=1.71 Hz, 1 H) 7.43 - 7.51 (m, 2 H) 3.49 (t, *J*=6.60 Hz, 3 H) 3.40 (br d, *J*=11.49 Hz, 2 H) 3.04 - 3.11 (m, 6 H) 2.86 (s, 5 H) 1.91 - 2.13 (m, 4 H) 1.50 - 1.82 (m, 6 H). **LCMS** (ESI+): *m/z* 463.2/465.2 (M+H)

Analog 73

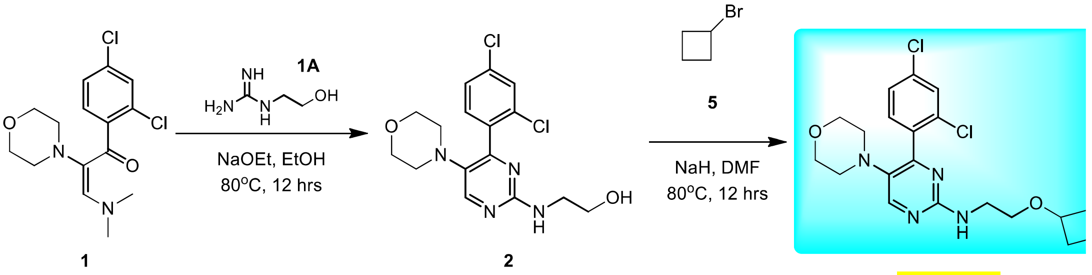

Analog 73

***N*-(2-cyclobutoxyethyl)-4-(2,4-dichlorophenyl)-5-morpholinopyrimidin-2-amine (Analog 73)**. To a solution of Compound 1 (500 mg, 1.52 mmol) and Compound 1A (313.23 mg, 3.04 mmol) in EtOH (3 mL) was added EtONa (413.40 mg, 6.07 mmol). The mixture was stirred at 80°C for 12 hrs. LCMS showed 25% of Compound 1 was remained and 42% of desired compound was detected. The reaction mixture was concentrated under reduced pressure to remove solvent. The residue was purified by flash silica gel chromatography (ISCO®; 12 g SepaFlash® Silica Flash Column, Eluent of 0~50% Ethyl acetate/Petroleum ether gradient @ 75 mL/min) (SiO2, Petroleum ether: Ethyl acetate=0:1, Rf(P1)=0.42) to afford Compound 2 (350 mg, 824.66 umol, 54.30% yield, 87% purity) as a yellow solid. MS-ESI (m/z) calcd for C16H18Cl2N4O2 [M+H]+: 369.1/371.1 Found 369.2/371.2. A solution of Compound 3 (200 mg, 2.77 mmol) in DCM (3 mL) was cooled to -20°C, then Py. (263.28 mg, 3.33 mmol, 268.65 uL) was added, then Tf2O (782.57 mg, 2.77 mmol, 457.65 uL) was added. The mixture was stirred at 25°C for 1 hr. TLC indicated Compound 3 was consumed and one major new spot with lower polarity was detected. The reaction mixture was diluted with H2O 5 mL and extracted with EtOAc (5 mL * 3). The combined organic layers were washed with brine (10 mL * 1), dried over anhydrous Na2SO4, filtered and concentrated under reduced pressure to give a residue to afford Compound 3 (230 mg, crude) as a yellow oil. To a solution of Compound 2 (100 mg, 270.82 umol) in DMF (2 mL) was added NaH (54.16 mg, 1.35 mmol, 60% purity) at 0°C. The mixture was stirred at 0°C for 20 min. Then Compound 5 (365.62 mg, 2.71 mmol, 255.68 uL) was added, the mixture was stirred at 80°C for 12 hrs. LCMS and showed 36% of Compound 2 was remained and 8% of desired compound was detected. The reaction mixture was concentrated under reduced pressure to remove solvent. The residue was purified by prep-HPLC (neutral condition, column: Phenomenex C18 80*40mm*3um; mobile phase: [water (NH4HCO3) - ACN]; B%: 40%-70%, 8 min) to afford **73** (8.49 mg, 19.53 umol, 7.21% yield, 97.37% purity) as a yellow gum. ^{1}**H NMR** (METHANOL-d_4 400MHz) δ ppm 8.19 - 8.31 (m, 1 H) 7.59 (s, 1 H) 7.44 (d, J=0.88 Hz, 2 H) 3.98 (quin, J=7.18 Hz, 1 H) 3.43 - 3.58 (m, 8 H) 2.72 - 2.82 (m, 4 H) 2.09 - 2.28 (m, 2 H) 1.82 - 2.00 (m, 2 H) 1.60 - 1.76 (m, 1 H) 1.42 - 1.59 (m, 1 H). LCMS (ESI+): m/z 423.1/425.1 (M+H)

Analog 74

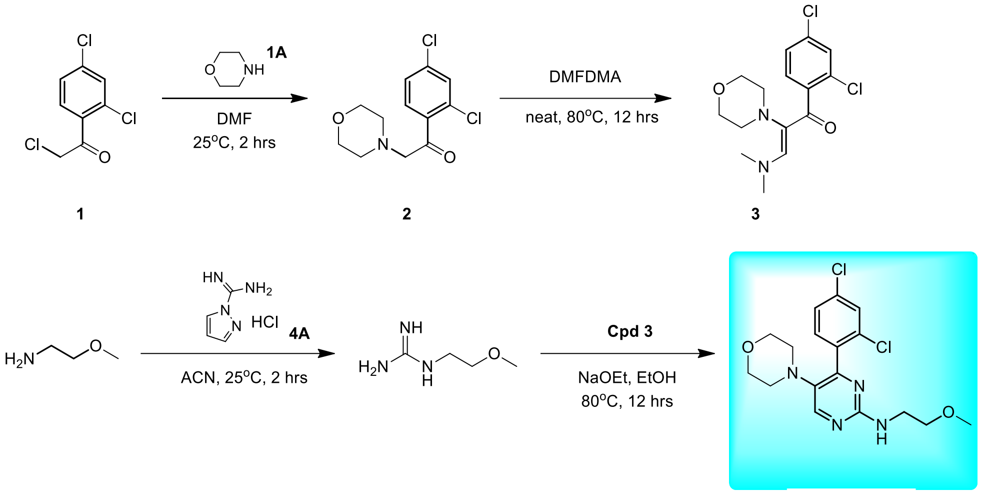

4 5 Analog 74

4-(2,4-dichlorophenyl)-*N*-(2-methoxyethyl)-5-morpholinopyrimidin-2-amine (Analog 74). To a solution of Compound 1 (5 g, 22.37 mmol) and Compound 1A (3.90 g, 44.74 mmol, 3.94 mL) in DMF (50 mL) was added DIEA (8.67 g, 67.11 mmol, 11.69 mL). The mixture was stirred at 25°C for 2 hrs. TLC indicated Compound 1 was consumed completely and one major new spot with larger polarity was detected. The reaction mixture was concentrated under reduced pressure to remove solvent. The residue was purified by flash silica gel chromatography (ISCO®; 40 g SepaFlash® Silica Flash Column, Eluent of 0~8% Ethyl acetate/Petroleum ether gradient @ 80 mL/min) (SiO2, Petroleum ether : Ethyl acetate=3:1, Rf(P1)=0.22) to afford Compound 2 (3 g, 9.74 mmol, 43.54% yield, 89% purity) as a red oil. MS-ESI (m/z) calcd for C12H13Cl2NO2 [M+H]+: 274.0/276.0 Found 274.0/276.0. A solution of Compound 2 (1 g, 3.65 mmol) in DMFDMA (8.97 g, 75.28 mmol, 10.00 mL) was stirred at 80°C for 12 hrs. TLC indicated Compound 2 was consumed completely and two major new spots with larger polarity was detected. The reaction mixture was concentrated under reduced pressure to remove solvent. The residue was purified by flash silica gel chromatography (ISCO®; 40 g SepaFlash® Silica Flash Column, Eluent of 0~10% Ethyl acetate/Petroleum ether gradient @ 100mL/min) (SiO2, Petroleum ether : Ethyl acetate=3:1, Rf(P1)=0.19) to afford Compound 3 (2.1 g, 95.09% purity, 43.75% yield) as a yellow solid. MS-ESI (m/z) calcd for C15H18Cl2N2O2 [M+H]+:329.1/331.1.Found 329.2/331.2. To a solution of Compound 4 (200 mg, 2.66 mmol, 231.48 uL) and Compound 4A (468.37 mg, 3.20 mmol) in ACN (2 mL) was stirred at 25°C for 2 hrs. LCMS showed Compound 4 was consumed completely and 100% of desired compound was detected. The mixture was concentrated under reduced pressure to remove solvent to afford Compound 5 (320 mg, crude, HCl) as a colourless liquid. MS-ESI (m/z) calcd for C4H11N3O [M+H]+: 118.1. Found 118.2. A solution of Compound 3 (40 mg, 121.50 umol) and Compound 5 (28.47 mg, 243.00 umol) EtOH (1 mL) was added EtONa (33.07 mg, 486.00 umol). The mixture was stirred at 80°C for 12 hrs. LCMS and HPLC showed 16% of Compound 3 was remained and 54% of desired compound was detected. The reaction was concentrated under reduced pressure to remove solvent. The residue was purified by prep-HPLC (basic condition) (Waters Xbridge BEH C18 100*30mm*10um; mobile phase: [water (NH3H2O + NH4HCO3) - ACN]; B%: 25%-55%, 8 min) to afford **74** (19.01 mg , 48.68 umol, 40.06% yield , 98.14% purity) as a yellow solid. **^{1}H NMR** (METHANOL-d_4 400MHz) δ ppm 8.24 (s, 1 H) 7.56 - 7.61 (m, 1 H) 7.44 (d, J=1.10 Hz, 2 H) 3.52 - 3.58 (m, 4 H) 3.47 - 3.52 (m, 4 H) 3.36 (s, 3 H) 2.72 - 2.81 (m, 4 H). LCMS (ESI+): m/z 383.1/385.1 (M+H)

Analog 76

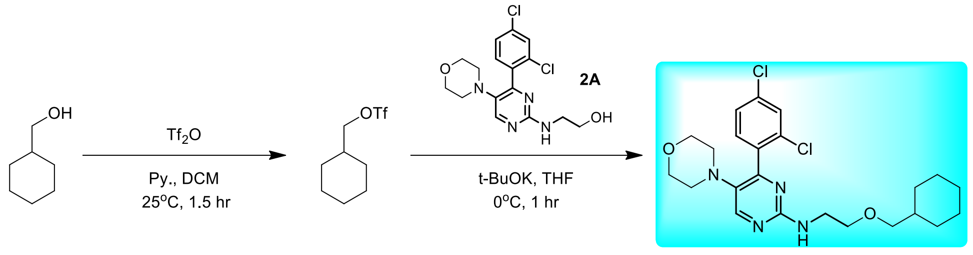

Analog 76

***N*-(2-(cyclohexylmethoxy)ethyl)-4-(2,4-dichlorophenyl)-5-morpholinopyrimidin-2-amine (Analog 76)**. To a solution of Compound 1 (500 mg, 4.38 mmol, 537.63 uL) in DCM (5 mL) was added Py. (381.00 mg, 4.82 mmol, 388.78 uL) at 0°C under N2. The mixture was stirred at 0°C for 5 min. Then Tf2O (1.36 g, 4.82 mmol, 794.73 uL) was added dropwise, the mixture was stirred at 0°C for 0.5 hr, then the mixture was stirred at 25°C for 1 hr. LCMS showed Compound 1 was consumed completely. The reaction mixture was diluted with H2O 5 mL and extracted with EtOAc (5 mL * 3). The combined organic layers were washed with brine (10 mL * 1), dried over anhydrous Na2SO4, filtered and concentrated under reduced pressure to afford Compound 2 (580 mg, crude) as a yellow oil. To a solution of Compound 2A (100 mg, 270.82 umol) in THF (2 mL) was added t-BuOK (91.17 mg, 812.47 umol) at 0°C. The mixture was stirred at 0°C for 10 min. Then Compound 2 (133.38 mg, 541.65 umol) was added, the mixture was stirred at 0°C for 1 hr. LCMS and HPLC showed 38% of Compound 4 was remained and 47% of desired compound was detected. The mixture was concentrated under reduced pressure to remove solvent. The residue was purified by prep-HPLC (TFA condition, column: Phenomenex Luna 80*30mm*3um; mobile phase: [water (TFA) - ACN]; B%: 55%-85%, 8 min). to afford **76** (33.53 mg, 56.35 umol, 20.81% yield, 97.38% purity, TFA) as a yellow gum. **^{1}H NMR** (METHANOL-*d*$_4$ 400MHz) δ ppm 8.19 (s, 1 H) 7.66 (d, J=1.75 Hz, 1 H) 7.47 - 7.60 (m, 2 H) 3.63 (br dd, J=10.63, 4.28 Hz, 4 H) 3.46 - 3.57 (m, 4 H) 3.27 (d, J=6.58 Hz, 2 H) 2.71 - 2.87 (m, 4 H) 1.69 (br d, J=10.08 Hz, 5 H) 1.53 (br dd, J=6.03, 3.18 Hz, 1 H) 1.09 - 1.31 (m, 3 H) 0.80 - 1.01 (m, 2 H). LCMS (ESI+): m/z 465.1/467.0 (M+H).

Analog 75

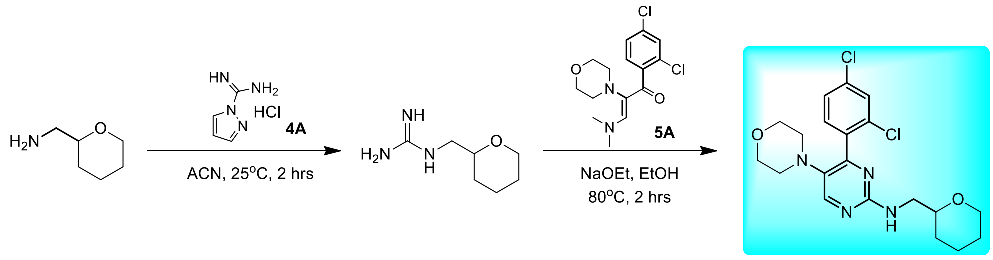

Analog 75

4-(2,4-dichlorophenyl)-5-morpholino-*N*-((tetrahydro-2*H*-pyran-2-yl)methyl)pyrimidin-2-amine (Analog 75). To a solution of Compound 4 (200 mg, 1.74 mmol) in EtOH (1 mL) and Compound 4A

(305.44 mg, 2.08 mmol) in ACN (2 mL) was stirred at 25 °C for 2 hrs. LCMS showed Compound 4 was consumed completely and 100% of desired compound was detected. The mixture was concentrated under reduced pressure to remove solvent to afford Compound 5 (270 mg, crude, HCl) as a colourless liquid. MS-ESI (m/z) calcd for C7H15N3O [M+H]+: 158.1. Found 158.2. A solution of Compound 5 (50 mg, 151.87 umol) and Compound 5A (47.75 mg, 303.75 umol) in EtOH (1 mL) was added EtONa (41.34 mg, 607.49 umol). The mixture was stirred at 80°C for 12 hrs. LCMS and HPLC showed 10% of Compound 5 was remained and 52% of desired compound was detected. The reaction was concentrated under reduced pressure to remove solvent. The residue was purified by prep-HPLC (TFA condition, column: Phenomenex luna C18 100*40mm*5 um; mobile phase: [water (TFA) - ACN]; B%: 35%-80%, 8 min) to afford **75** (17.23 mg, 31.64 umol, 20.83% yield, 98.67% purity, TFA) as a yellow solid. ^{1}**H NMR** (METHANOL-*d*$_4$ 400MHz) δ ppm 8.19 (s, 1 H) 7.64 (d, J=1.63 Hz, 1 H) 7.45 - 7.55 (m, 2 H) 3.93 - 4.03 (m, 1 H) 3.48 - 3.59 (m, 6 H) 3.34 - 3.45 (m, 2 H) 2.71 - 2.82 (m, 4 H) 1.87 (br s, 1 H) 1.50 - 1.69 (m, 4 H) 1.27 - 1.42 (m, 1 H). LCMS (ESI+): m/z 423.0/425.0 (M+H)

Analog 77

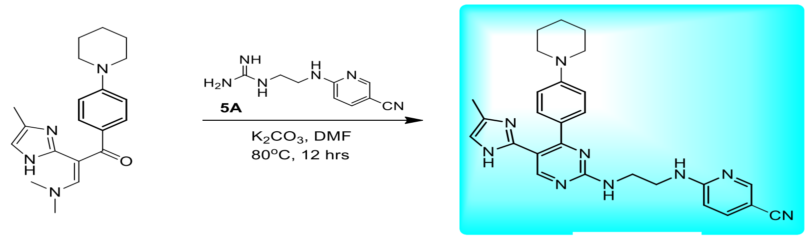

Analog 77

6-(2-((4-(2,4-dichlorophenyl)-5-(oxazol-2-yl)pyrimidin-2-yl)amino)ethoxy)nicotinonitrile (Analog 77). To a solution of **Compound 4B** (79.14 mg, 385.65 umol) in EtOH (3 mL) was added NaOEt (52.49 mg, 771.30 umol) and the mixture was stirred at 80°C for 0.5 hr, then **Compound 4** (120 mg, 385.65 umol) was added, and the resulting mixture was stirred at 80°C for 1 hr. LC-MS showed 35% **Compound 4** was remained and 25% desired product was detected. The reaction mixture was filtered and the filtrate was collected. The filtrate was purified by Prep-HPLC (neutral condition) (column: Phenomenex Gemini-NX C18 75*30mm*3um; mobile phase: [water (10mM NH_4HCO_3)-ACN]; B%: 40%-60%, 6min) to afford *77* (27.2 mg, 60.01 umol, 15.56% yield, 100% purity) as a white solid. ^{1}**H NMR** (METHANOL-*d*$_4$ 400MHz) δ ppm 8.87 (br s, 1 H) 8.47 (br s, 1 H) 7.81 - 8.02 (m, 1 H) 7.72 (d, *J*=0.61 Hz, 1 H) 7.49 (d, *J*=1.96 Hz, 1 H) 7.41 - 7.45 (m, 1 H) 7.37 (br s, 1 H) 7.13 (d, *J*=0.61 Hz, 1 H) 6.88 (br s, 1 H) 4.56 - 4.69 (m, 2 H) 3.89 (br s, 2 H). **LCMS** (ESI+): *m/z* 453.1/455.1 (M+H)

Analog 78

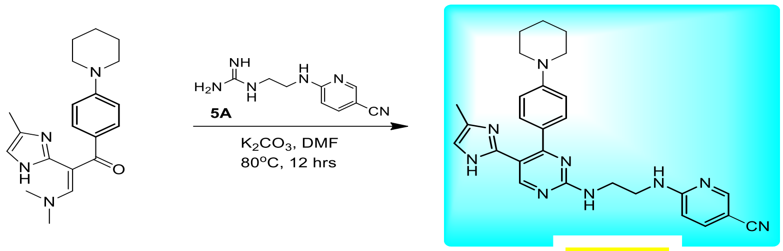

5

Analog 78

6-(2-((4-(2,4-dichlorophenyl)-5-(thiazol-2-yl)pyrimidin-2-yl)amino)ethoxy)nicotinonitrile (Analog 78). To a solution of **Compound 4B** (112.88 mg, 550.07 umol) in EtOH (2 mL) was added EtONa (74.87 mg, 1.10 mmol) at 20°C, then the mixture was stirred at 80°C for 10 min, then another solution of **Compound 4** (180 mg, 550.07 umol) in EtOH (2 mL) was added at 80°C, then the mixture was stirred at 80°C for 2 hrs. LCMS showed 49% of **Compound 4** was remained and 11% of desired product was detected. Then EtONa (74.87 mg, 1.10 mmol) was added at 20°C, the mixture was stirred at 80°C for 12 hrs. LCMS showed 13% of **Compound 4** was remained and 51% of desired product was detected. The reaction was filtered. The filtrate was purified by Prep-HPLC (neutral condition) (column: Waters Xbridge Prep OBD C18 150*40mm*10um; mobile phase: [water (10 mM NH_4HCO_3)-ACN]; B%: 45%-75%, 8 min) to afford ***78*** (41.9 mg, 88.70 umol, 16.13% yield, 99.36% purity) as a pale yellow solid. **^{1}H NMR** (METHANOL-d_4 400MHz) δ ppm 8.93 (br s, 1 H) 8.49 (br s, 1 H) 7.92 (br s, 1 H) 7.74 (d, J=3.30 Hz, 1 H) 7.55 (d, J=1.96 Hz, 1 H) 7.49 (dd, J=8.19, 1.96 Hz, 1 H) 7.43 (d, J=3.30 Hz, 1 H) 7.39 (br d, J=8.07 Hz, 1 H) 6.91 (br d, J=6.72 Hz, 1 H) 4.61 (br s, 2 H) 3.88 (br s, 2 H). **LCMS** (ESI+): *m*/*z* 469.1/471.0 (M+H)

Analog 79

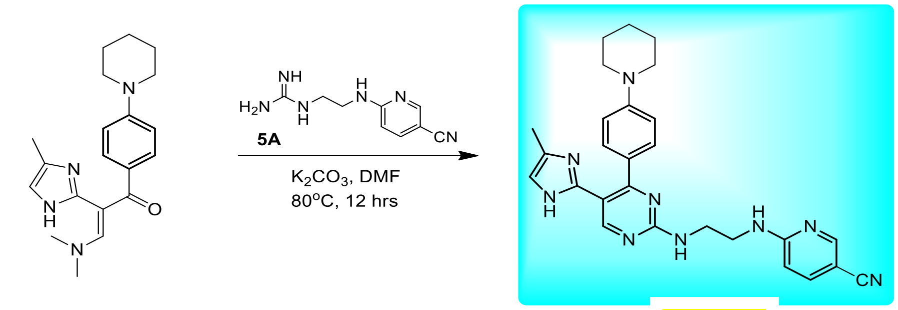

6-(2-((4-(2,4-dichlorophenyl)-5-(1*H*-imidazol-2-yl)pyrimidin-2-yl)amino)ethoxy)-nicotinonitrile (Analog 79). To a solution of **Compound 1** (105 mg, 511.66 umol) in EtOH (1.5 mL) was added EtONa (139.27 mg, 2.05 mmol) at 20°C, then the mixture was stirred at 80°C for 15 min, then another solution of **Compound 3a** (158.70 mg, 511.66 umol) in EtOH (1.5 mL) was added at 80°C, then the mixture was stirred at 80°C for 12 hrs. LCMS showed 9% of **Compound 1** was remained and 28% of desired product was detected. The reaction was filtered. The filtrate was purified by Prep-HPLC (neutral condition) (column: Phenomenex Gemini-NX 150*30mm*5um; mobile phase: [water (10 mM NH_4HCO_3)-ACN]; B%: 25%-55%, 8 min) to afford ***79*** (16.4 mg, 36.18 umol, 7.07% yield, 99.79% purity) as a pale yellow solid. ^{1}**H NMR** (METHANOL-d_4 400MHz) δ ppm 8.51 (br d, *J*=18.46 Hz, 2 H) 7.89 (br s, 1 H) 7.30 - 7.44 (m, 3 H) 6.97 (s, 2 H) 6.89 (br s, 1 H) 4.61 (t, *J*=5.32 Hz, 2 H) 3.87 (t, *J*=5.38 Hz, 2 H). **LCMS** (ESI+): *m*/*z* 452.1/454.1 (M+H)

Analog 80

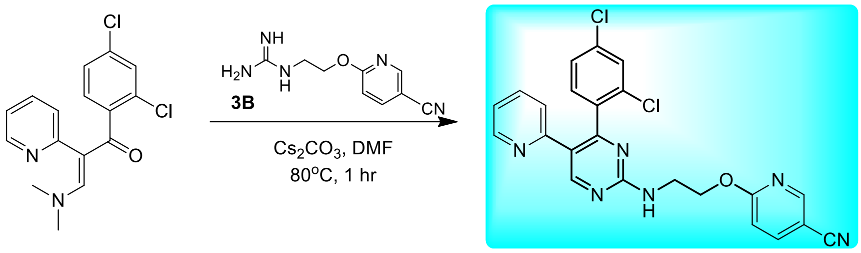

Analog 80

6-(2-((4-(2,4-dichlorophenyl)-5-(pyridin-2-yl)pyrimidin-2-yl)amino)ethoxy)nicotinonitrile (Analog 80). To a solution of **Compound 3** (160 mg, 498.13 umol) and **Compound 3B** (102.22 mg, 498.13 umol) in DMF (2 mL) was added Cs_2CO_3 (486.90 mg, 1.49 mmol) and the mixture was stirred at 80°C for 1 hr. The LC-MS showed **Compound 3** was consumed completely and one main peak with desired mass was detected. The reaction was filtered and the filtrate was collected. The filtrate was purified by Prep-HPLC (basic condition) (column: Waters Xbridge Prep OBD C18 150*40mm*10um; mobile phase: [water (0.05%$NH_3.H_2O$+10mM NH_4HCO_3)-ACN]; B%: 40%-70%, 8min) to afford ***80*** (35.6 mg, 75.95 umol, 15.25% yield, 98.85% purity) as a yellow solid. **^{1}H NMR** (METHANOL-d_4 400MHz) δ ppm 8.61 (br s, 1 H) 8.49 (br d, *J*=2.93 Hz, 2 H) 7.90 (br s, 1 H) 7.61 (td, *J*=7.73, 1.65 Hz, 1 H) 7.34 - 7.44 (m, 3 H) 7.24 (dd, *J*=6.72, 5.01 Hz, 1 H) 6.99 (br d, *J*=7.95 Hz, 1 H) 6.89 (br d, *J*=8.19 Hz, 1 H) 4.62 (br t, *J*=5.32 Hz, 2 H) 3.83 - 3.92 (m, 2 H). **LCMS** (ESI+): *m/z* 463.1/465.1 (M+H)

Analog 81

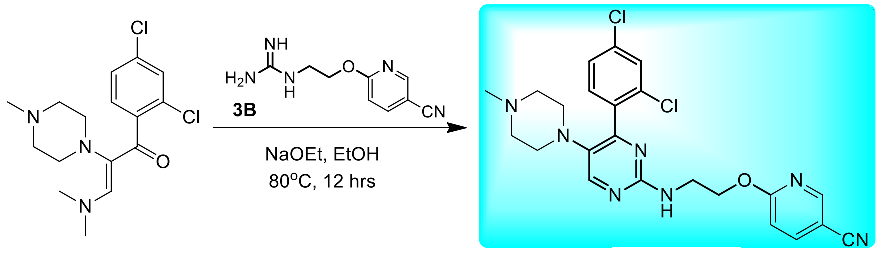

3

Analog 81

6-(2-((4-(2,4-dichlorophenyl)-5-(4-methylpiperazin-1-yl)pyrimidin-2-yl)amino)ethoxy)-nicotinonitrile (Analog 81). To a solution of **Compound 3B** (77.95 mg, 379.83 umol) in EtOH (2 mL) was added EtONa (103.39 mg, 1.52 mmol) at 20°C, then the mixture was stirred at 80°C for 10 min, then another solution of **Compound 3** (130 mg, 379.83 umol) in EtOH (2 mL) was added at 80°C, then the mixture was stirred at 80°C for 12 hrs. LCMS showed 48% of **Compound 3** remained and 23% of desired product was detected. The reaction was filtered. The filtrate was purified by Prep-HPLC (HCl condition) (column: Phenomenex luna C18 80*40mm*3 um;mobile phase: [water (0.04% HCl)-ACN]; B%: 15%-35%, 7 min), the HNMR was not clean, so the residue was further purified by Prep-HPLC(basic condition) (column: Waters Xbridge Prep OBD C18 150*40mm*10um; mobile phase: [water (0.05% NH_3H_2O+10 mM NH_4HCO_3)-ACN]; B%: 35%-65%, 8 min) to afford ***81*** (13.8 mg, 27.96 umol, 7.36% yield, 98.13% purity) as a yellow gum. **^{1}H NMR** (METHANOL-d_4 400MHz) δ ppm 8.47 (br d, *J*=1.59 Hz, 1 H) 8.19 - 8.30 (m, 1 H) 7.89 (br dd, *J*=8.68, 2.20 Hz, 1 H) 7.52 - 7.62 (m, 1 H) 7.35 - 7.48 (m, 2 H) 6.85 - 6.94 (m, 1 H) 4.50 - 4.62 (m, 2 H) 3.78 (br d, *J*=3.91 Hz, 2 H) 2.80 (br d, *J*=3.67 Hz, 4 H) 2.14 - 2.40 (m, 7 H). **LCMS** (ESI+): *m/z* 484.2/486.2 (M+H)

Analog 82

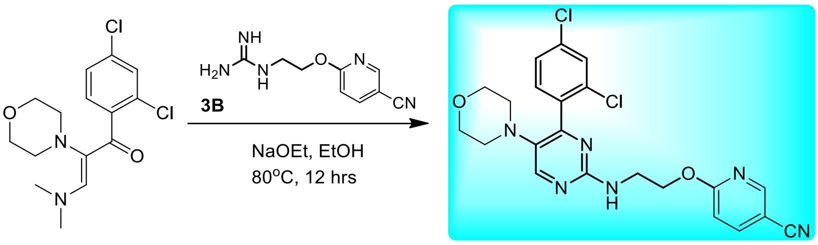

3

Analog 82

6-(2-((4-(2,4-dichlorophenyl)-5-morpholinopyrimidin-2-yl)amino)ethoxy)nicotinonitrile (Analog 82). To a solution of **Compound 3B** (124.67 mg, 607.49 umol) in EtOH (2 mL) was added EtONa (124.02 mg, 1.82 mmol) at 20°C, then the mixture was stirred at 80°C for 15min, then another solution of **Compound 3** (200 mg, 607.49 umol) in EtOH (2 mL) was added at 80°C, then the mixture was stirred at 80°C for 12 hrs. LCMS showed 53% of **Compound 3** was remained and 23% of desired product was detected. The reaction was filtered. The filtrate was purified by Prep-HPLC (HCl condition) (column: Welch Xtimate C18 150*25mm*5um; mobile phase: [water (0.04% HCl)-ACN]; B%: 30%-50%, 8 min), but the HNMR was not clean, then the residue was purified further by Prep-HPLC (basic condition) (column: Phenomenex Gemini-NX C18 75*30mm*3um; mobile phase: [water (0.05%NH_3H_2O+10 mM NH_4HCO_3)-ACN]; B%: 35%-65%, 8 min), HNMR was still not clean, then the residue was purified further by Prep-TLC (Petroleum ether: Ethyl acetate=1:1) (P1 Rf=0.25) to afford ***82*** (10 mg, 20.88 umol, 3.44% yield, 98.41% purity) as a light yellow gum. **^{1}H NMR** (METHANOL-d_4 400MHz) δ ppm 8.48 (d, J=1.76 Hz, 1 H) 8.23 (s, 1 H) 7.90 (dd, J=8.71, 2.09 Hz, 1 H) 7.58 (s, 1 H) 7.38 - 7.45 (m, 2 H) 6.88 (d, J=8.82 Hz, 1 H) 4.56 (t, J=5.40 Hz, 2 H) 3.78 (t, J=5.51 Hz, 2 H) 3.44 - 3.53 (m, 4 H) 2.71 - 2.79 (m, 4 H). **LCMS** (ESI+): m/z 471.1/473.1 (M+H)

Analog 85, 83

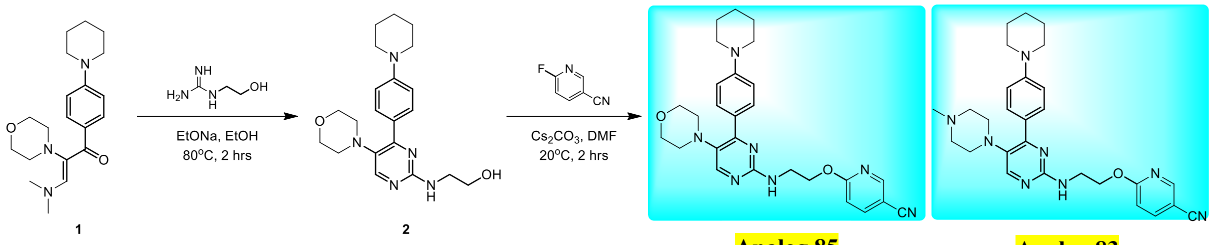

Analog 85 **Analog 83**

6-(2-((5-morpholino-4-(4-(piperidin-1-yl)phenyl)pyrimidin-2-yl)amino)ethoxy)-nicotinonitrile (Analog 85). To a solution of 1-(2-hydroxyethyl)guanidine (120.10 mg, 1.16 mmol) in EtOH (3 mL) was added NaOEt (118.88 mg,1.75 mmol) and the mixture was stirred at 80°C for 0.5 hr, then **Compound 1** (200 mg, 582.31 umol) was added, and the resulting mixture was stirred at 120°C for 6 hrs under microwave. To a solution of **Compound 2** (30 mg, 78.23 umol) in THF (2 mL) was added t-BuOK (26.33 mg, 234.69 umol) and the mixture was stirred at 20°C for 0.5 hr, then 6-fluoropyridine-3-carbonitrile (14.33 mg, 117.34 umol) was added, and the resulting mixture was stirred at 20°C for 1.5 hr. LC-MS showed **Compound 2** was consumed completely and one main peak with desired mass was detected. The reaction mixture was concentrated under reduced pressure to remove solvent. The residue was purified by prep-HPLC (column: Phenomenex Gemini-NX C18 75*30mm*3um;mobile phase: [water(10mM NH4HCO3)-ACN];B%: 40%-70%,12min) to obtained ***A85*** (1.22 mg, 2.51 umol, 3.21% yield, 100% purity) as a yellow solid.

A similar procedure was used to make **6-(2-((5-(4-methylpiperazin-1-yl)-4-(4-(piperidin-1-yl)phenyl)pyrimidin-2-yl)amino)ethoxy)nicotinonitrile (Analog 83)**. **^{1}H NMR** (METHANOL-d_4

400MHz) δ ppm 8.29 (d, J=1.75 Hz, 1 H) 8.19 (s, 1 H) 8.13 (d, J=9.01 Hz, 2 H) 7.53 (br d, J=10.88 Hz, 1 H) 6.99 (d, J=8.88 Hz, 2 H) 6.54 (d, J=8.88 Hz, 1 H) 4.53 - 4.58 (m, 6 H) 3.69 - 3.85 (m, 6 H) 2.80 - 2.93 (m, 4 H) 1.60 - 1.76 (m, 6 H)). **LCMS** (ESI+): *m/z* 486.3 (M+H).

Analog 86

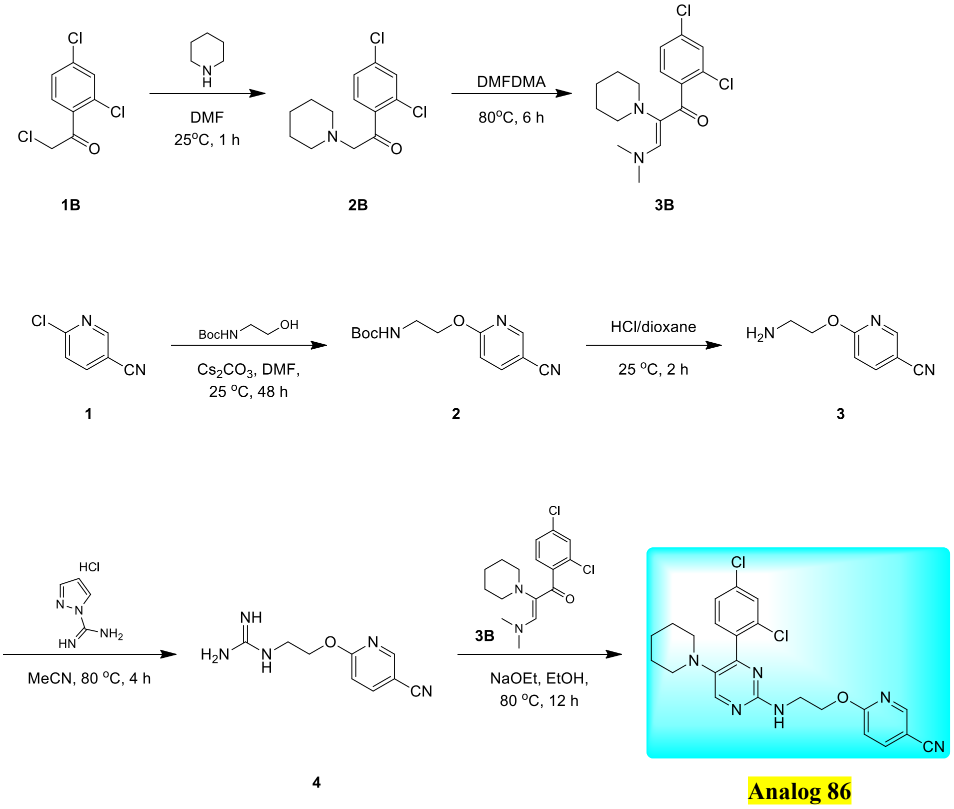

6-(2-((4-(2,4-dichlorophenyl)-5-(piperidin-1-yl)pyrimidin-2-yl)amino)ethoxy)-nicotinonitrile (Analog 86). To a mixture of **compound 1B** (2 g, 8.95 mmol, 1 *eq*) in DMF (20 mL) was added piperidine (7.62 g, 89.49 mmol, 8.84 mL, 10 *eq*) dropwise at 10°C under N_2. The mixture was stirred at 25 °C for 1 h. TLC (petroleum ether: ethyl acetate=5:1, R_f = 0.25) showed the product. The residue was poured into water (50 mL) and stirred for 5min. The aqueous phase was extracted with ethyl acetate (20 mL*2). The combined organic phase was washed with brine (15 mL), dried with anhydrous Na_2SO_4, filtered and concentrated in vacuum. The residue was purified by silica gel chromatography (petroleum ether/ethyl acetate=100/1, 10/1) to give **compound 2B** (2.1 g, 7.72 mmol, 86.22% yield) was obtained as yellow oil. HNMR (ET39965-75-P1A) showed correct product. **1H NMR (400MHz, CHLOROFORM-d)** δ = 7.45 - 7.40 (m, 2H), 7.29 (dd, J=2.0, 8.4 Hz, 1H), 3.64 (s, 2H), 2.53 - 2.41 (m, 4H), 1.56 (quin, J=5.6 Hz, 4H), 1.46 - 1.36 (m, 2H). A mixture of **compound 2B** (2.1 g, 7.72 mmol, 1 *eq*) in DMF-DMA (60 mL) was stirred at 80 °C for 6 hours. TLC (petroleum ether: ethyl acetate=5:1, R_f= 0.50) showed the material was consumed completely. LCMS showed desired mass. The residue was poured into water (20 mL) and stirred for 5 min. The aqueous phase was extracted with ethyl acetate (8 mL*3). The combined

organic phase was washed with brine (10 mL), dried with anhydrous Na_2SO_4, filtered and concentrated in vacuum. The residue was purified by prep-TLC (Petroleum ether: Ethyl acetate=5:1) to give **compound 3B** (1 g, 2.90 mmol, 37.62% yield, 95% purity) as a yellow solid. HNMR (ET39965-80-P1A) **1H NMR (400MHz, CHLOROFORM-d)** δ = 7.36 (d, J=1.9 Hz, 1H), 7.25 - 7.20 (m, 1H), 7.19 - 7.13 (m, 1H), 6.28 (s, 1H), 3.40 - 2.89 (m, 10H), 1.55 (br s, 6H) MS-ESI (m/z) calcd for C16H20Cl2N2O $[M+H]^+$: 327.0/329.0. To a mixture of tert-butyl N-(2-hydroxyethyl)carbamate (1.52 g, 9.41 mmol, 1.46 mL, 1.2 *eq*) in DMF (10 mL) was added Cs2CO3 (3.83 g, 11.76 mmol, 1.5 *eq*) at 25°C. Then **compound 1** (1 g, 7.84 mmol, 854.70 uL, 1 *eq*) was added to the mixture and stirred at 25 °C for 48 hrs. TLC (petroleum ether: ethyl acetate = 3:1, R_f = 0.60) showed the start material was consumed completely. The mixture was poured into water (40 mL). The aqueous phase was extracted with ethyl acetate (15 mL*2). The combined organic phase was washed with brine (10 mL), dried with anhydrous Na_2SO_4, filtered and concentrated in vacuum. The residue was purified by silica gel chromatography (petroleum ether/ethyl acetate=100/1, 1/1) to give **compound 2** (1.3 g, 4.94 mmol, 62.99% yield) was obtained as a white solid. **^{1}H NMR (400 MHz, CHLOROFORM-*d*)** δ ppm 8.47 (d, *J*=2.32 Hz, 1 H) 7.80 (dd, *J*=8.68, 2.32 Hz, 1 H) 6.83 (d, *J*=8.68 Hz, 1 H) 4.44 (t, *J*=5.26 Hz, 2 H) 3.55 (br d, *J*=5.14 Hz, 2 H) 1.45 (s, 9 H)

A mixture of **compound 2** (1.2 g, 4.56 mmol, 1 *eq*) in HCl/dioxane (4 M, 6.84 mL, 6 *eq*) was stirred at 25 °C for 2 h. LCMS showed the reaction was completed. The mixture was concentrated in reduced pressure at 40°C. it was not purified and used to next step directly. **Compound 3** (1.1 g, crude, HCl) was obtained as a white solid. **1H NMR (400MHz, DMSO-d6)** δ = 8.72 (d, J=2.1 Hz, 1H), 8.20 (br dd, J=2.3, 8.7 Hz, 4H), 7.03 (d, J=8.7 Hz, 1H), 4.58 - 4.45 (m, 1H), 4.53 (t, J=5.2 Hz, 1H), 3.23 (br d, J=4.6 Hz, 2H). MS-ESI (m/z) calcd for C8H9N3O $[M+H]^+$: 164.0. To a mixture of **compound 3** (300 mg, 1.50 mmol, 1 *eq*, HCl) and pyrazole-1-carboxamidine (330.40 mg, 2.25 mmol, 1.5 *eq*, HCl) in MeCN (3 mL) was added TEA (608.24 mg, 6.01 mmol, 836.64 uL, 4 *eq*) in one portion at 25°C. The mixture was stirred at 80 °C for 4 h. LCMS showed the product. The mixture was concentrated in vacuum at 45°C. The residue was not purified and used to next step directly. **Compound 4** (0.31 g, crude) was obtained as a white solid. MS-ESI (m/z) calcd for C9H11N5O $[M+H]^+$: 206.0.

To a mixture of (Z)-1-(2,4-dichlorophenyl)-3-(dimethylamino)-2-(1-piperidyl)prop-2-en-1-one (0.1 g, 305.58 umol, 1 *eq*) and **compound 4** (188.13 mg, 916.74 umol, 3 *eq*) in EtOH (5 mL) was added EtONa (103.97 mg, 1.53 mmol, 5 *eq*) in one portion at 25°C. The mixture was stirred at 80°C for 12 h. LCMS showed desired mass. The mixture was concentrated to remove EtOH, to the residue was added H2O (20 mL) and aqueous phase was extracted with ethyl acetate (20 mL*2). The combined organic phase was washed with brine (20mL), dried with anhydrous Na_2SO_4, filtered and concentrated in vacuum. The residue was purified by prep-HPLC: column: Phenomenex Luna 80*30mm*3um;mobile phase: [water(0.1%TFA)-ACN];B%: 50%-85%,8min to give **86** (1.65 mg, 3.45 umol, 1.13% yield, 98.08% purity) as a yellow solid. **^{1}H NMR** (METHANOL-d_4 400MHz) δ ppm 8.47 (d, J=1.96 Hz, 1 H) 8.16 (s, 1 H) 7.89 (dd, J=8.62, 2.26 Hz, 1 H) 7.58 (d, J=1.22 Hz, 1 H) 7.37 - 7.47 (m, 2 H) 6.88 (d, J=8.68 Hz, 1 H) 4.57 (t, J=5.38 Hz, 2 H) 3.81 (t, J=5.32 Hz, 2 H) 2.72 (br s, 4 H) 1.41 (br s, 6 H). **LCMS** (ESI+): *m/z* 469.0/471.0 (M+H)

Analog 87

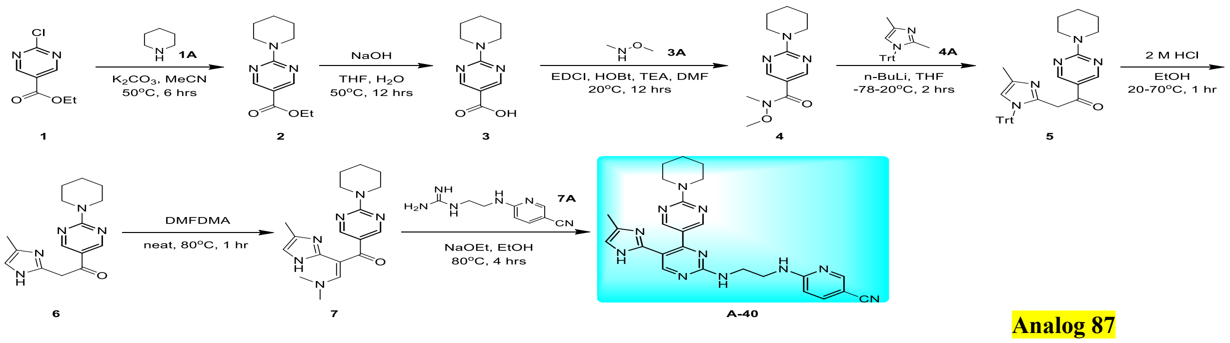

Analog 87

6-(2-((4-(2,4-dichlorophenyl)-5-morpholinopyrimidin-2-yl)(methyl)amino)ethoxy)-nicotinonitrile (Analog 87). To a stirred solution of **Compound 1** (400 mg, 1.44 mmol) in EtOAc (6 mL) was added HCl/EtOAc (4 M, 8.65 mL). The resulting mixture was stirred at 15°C for 1 hr. TLC indicated the reaction was completed. The reaction solution was filtered and concentrated under reduced pressure to give ***Compound 2*** (300 mg, crude) as a white solid. To a solution of **Compound 2** (93.00 mg, 435.26 umol, HCl) in DMF (3 mL) was added NaH (34.82 mg, 870.52 umol, 60% purity) at 0°C, then **Compound 2A** (60 mg, 174.10 umol) was added. The mixture was stirred at 15°C for 2 hrs. LC-MS showed the reaction was completed. The reaction mixture was quenched by poured into saturated NH_4Cl solution 5 mL and extracted with EtOAc (2 mL * 3). The combined organic layers were washed with brine (3 mL * 3), dried over Na_2SO_4, filtered and concentrated under reduced pressure to give a residue. The residue was purified by prep-HPLC (column: Phenomenex Luna C18 75*30mm*3um; mobile phase: [water (0.2%FA) - ACN]; B%: 20%-60%, 8 min) to give Compound ***87*** (18.6 mg, 38.32 umol, 22.01% yield) as a pale yellow solid. **^{1}H NMR** (METHANOL-d_4 400MHz) δ ppm 8.42 (s, 1H), 8.29 (d, J=2.1 Hz, 1H), 7.66 - 7.58 (m, 2H), 7.50 - 7.43 (m, 2H), 6.69 (d, J=9.0 Hz, 1H), 4.64 (t, J=5.4 Hz, 2H), 4.06 (t, J=5.5 Hz, 2H), 3.58 - 3.50 (m, 4H), 3.15 (s, 3H), 2.85 - 2.77 (m, 4H). **LCMS** (ESI+): *m/z* 485.2/487.2 (M+H)

Analog 88

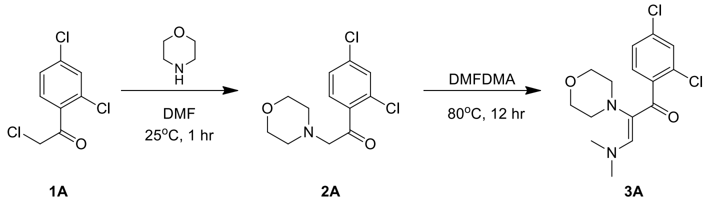

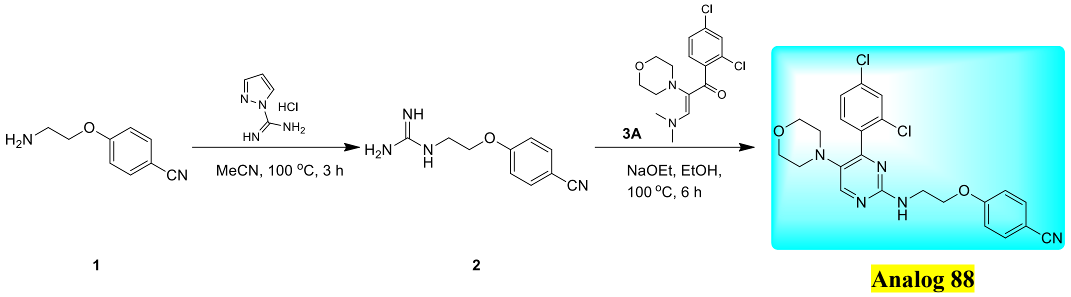

Analog 88

4-(2-((4-(2,4-dichlorophenyl)-5-morpholinopyrimidin-2-yl)amino)ethoxy)benzonitrile (Analog 88). A mixture of **compound 1A** (5 g, 22.37 mmol, 1 *eq*) and morpholine (19.49 g, 223.73 mmol, 19.69 mL, 10 *eq*) in DMF (50 mL) The mixture was stirred at 25 °C for 1 hours. TLC (petroleum ether: ethyl acetate=10:1, R_f=0.6) indicated reactant 1A was consumed completely and **one new spot** formed. The mixture was poured into water and extracted with ethyl acetate (200 mL*3). The combined organic phase was washed with brine (300 mL*2), dried with anhydrous Na_2SO_4, filtered and concentrated in vacuum. The residue was purified by column chromatography (SiO2, **Petroleum ether/Ethyl acetate=100/1** to 0/**1, R_f = 0.3**). **Compound 2A** (6 g, 21.89 mmol, 97.83% yield) was obtained as a yellow oil. **1H NMR (400MHz, CHLOROFORM-d)** δ = 7.50 - 7.44 (m, 2H), 7.34 (dd, J=1.9, 8.3 Hz, 1H), 3.80 - 3.70 (m, 6H), 2.67 - 2.55 (m, 4H). The mixture of **compound 2A** (3 g, 10.94 mmol, 1 *eq*) in DMFDMA (90 mL) was stirred at 80 °C for 12 hours. TLC (petroleum ether: ethyl acetate=1:1, R_f= 0.3) indicated reactant 2A was consumed completely and **one new spot** formed. The mixture was poured into water and extracted with ethyl acetate (200 mL*3). The combined organic phase was washed with brine (300 mL*2), dried with anhydrous Na_2SO_4, filtered and concentrated in vacuum. The residue was purified by column chromatography (SiO2, **Petroleum ether/Ethyl acetate=100/1** to 3/**1, R_f = 0.3**). **Compound 3A** (2 g, 6.07 mmol, 55.51% yield) was obtained as a yellow solid. The structure was confirmed by HNMR. **1H NMR (400MHz, CHLOROFORM-d)** δ = 7.36 (s, 1H), 7.26 - 7.21 (m, 1H), 7.20 - 7.14 (m, 1H), 6.33 (s, 1H), 3.72 (br s, 6H), 3.33 - 2.98 (m, 8H). The mixture of **compound 1** (0.2 g, 1.23 mmol, 1 *eq*) and pyrazole-1-carboxamidine (216.90 mg, 1.48 mmol, 1.2 *eq*, HCl) in MeCN (5 mL) was stirred at 100 °C for 3 hours. LCMS showed the desired ms was detected. Wihtout workup and the mixture was concentrated. Wihtout purification and the residue was used for the next step directly. **Compound 2** (0.3 g, crude) was obtained as a white solid. MS-ESI (m/z) calcd for C10H12N4O $[M+H]^+$: 205.0. To a mixture of **compound 2** (0.2 g, 979.30 umol, 1 *eq*) and **compound 3A** (386.89 mg, 1.18 mmol, 1.2 *eq*) in EtOH (5 mL) was added NaOEt (199.92 mg, 2.94 mmol, 3 *eq*) under N_2. The mixture was stirred at 100 °C for 6 hours. LCMS showed the desired ms was detected. Without workup and the mixture was concentrated. The residue was purified by prep-HPLC:column: Phenomenex luna C18 80*40mm*3 um;mobile phase: [water(0.1%TFA)-ACN];B%: 45%-75%,7min to give compound **88** (10.96 mg, 21.95 umol, 2.24% yield, 94.19% purity) as a yellow solid. **^{1}H NMR**

(METHANOL-d_4 400MHz) δ ppm 8.25 (s, 1 H) 7.55 - 7.65 (m, 3 H) 7.47 (s, 2 H) 7.09 (d, J=8.82 Hz, 2 H) 4.25 (t, J=5.73 Hz, 2 H) 3.81 (t, J=5.73 Hz, 2 H) 3.47 - 3.53 (m, 4 H) 2.73 - 2.80 (m, 4 H). **LCMS** (ESI+): *m/z* 470.1/472.1 (M+H).

Analog 89

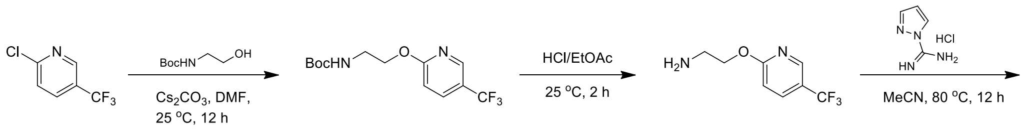

1 2 3

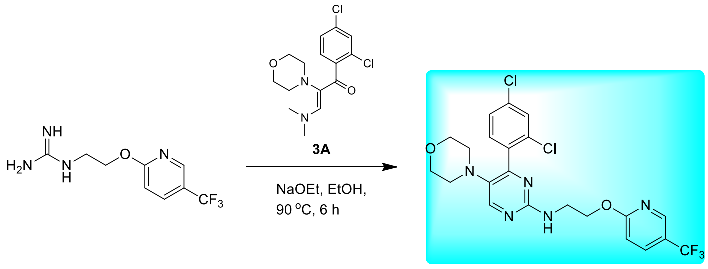

4 **Analog 89**

4-(2,4-dichlorophenyl)-5-morpholino-*N*-(2-((5-(trifluoromethyl)pyridin-2-yl)oxy)ethyl)-pyrimidin-2-amine (Analog 89). To a mixture of **compound 1** (0.5 g, 2.75 mmol, 352.11 uL, 1 *eq*) and tert-butyl N-(2-hydroxyethyl)carbamate (1.55 g, 9.64 mmol, 1.49 mL, 3.5 *eq*) in DMF (7 mL) was added Cs2CO3 (2.24 g, 6.89 mmol, 2.5 *eq*). The mixture was stirred at 25°C for 12 hours. TLC (petroleum ether: ethyl acetate=3:1, R_f= 0.5) indicated **compound 1** was consumed completely and **one new spot** formed. The mixture was poured into water and extracted with ethyl acetate (30 mL*2). The combined organic phase was washed with brine (50 mL), dried with anhydrous Na_2SO_4, filtered and concentrated in vacuum. The residue was purified by column chromatography (SiO2, **Petroleum ether/Ethyl acetate=100/1 to 3:1, R_f= 0.5**). **Compound 2** (0.72 g, 2.35 mmol, 85.35% yield) was obtained as a white solid. **[1]H**

NMR (400 MHz, CHLOROFORM-*d*) δ ppm 8.42 (s, 1 H) 7.78 (br d, *J*=8.58 Hz, 1 H) 6.83 (d, *J*=8.70 Hz, 1 H) 4.94 (br s, 1 H) 4.43 (br t, *J*=4.83 Hz, 2 H) 3.55 (br d, *J*=4.65 Hz, 2 H) 1.45 (s, 8 H). A mixture of **compound 2** (0.72 g, 2.35 mmol, 1 *eq*) in HCl/dioxane (8 mL) was stirred at 25 °C for 2 hrs under N_2 atmosphere. TLC (petroleum ether: ethyl acetate=1:1) showed the reaction was completed. The mixture was concentrated to give residue. The crude was used next step directly, **compound 3** (0.6 g, crude, HCl) as a white solid. **1H NMR (400 MHz, DMSO-d6)**

δ ppm 8.59 (s, 1 H) 8.44 (br s, 3 H) 8.10 (dd, J=8.82, 2.43 Hz, 1 H) 7.08 (br s, 2 H) 7.04 (d, J=8.82 Hz, 1 H) 4.54 (t, J=5.29 Hz, 2 H) 3.15 - 3.26 (m, 2 H). A mixture of **compound 3** (0.2 g, 824.31 umol, 1 *eq*, HCl) pyrazole-1-carboxamidine (181.24 mg, 1.24 mmol, 1.5 *eq*, HCl) TEA (250.23 mg, 2.47 mmol, 344.20 uL, 3 *eq*) in MeCN (3 mL) was stirred at 80 °C for 12 h. LCMS showed the mixture was completed. The mixture was concentrated to give residue. The crude was used next step directly. **Compound 4** (0.2 g, crude) as white solid.

MS-ESI (m/z) calcd for C9H11F3N4O $[M+H]^+$: 249.1. To a mixture of **compound 4** (0.2 g, 805.79 umol, 1 *eq*) (Z)-1-(2,4-dichlorophenyl)-3-(dimethylamino)-2-morpholino-prop-2-en-1-one (265.28 mg, 805.79 umol, 1 *eq*) in EtOH (5 mL) was added NaOEt (164.50 mg, 2.42 mmol, 3 *eq*) at 25 °C, the mixture was stirred at 90 °C for 6 h. LCMS showed desired peak with desired MS. The mixture was concentrated to give residue, to the residue was added H2O (50 mL) and DCM (50 mL), aqueous phase was extracted with DCM (50 mL*2). The combined organic phase was washed with brine (50 mL*2), dried with anhydrous Na_2SO_4, filtered and concentrated in vacuum. The residue was purified by prep-HPLC: :column: Phenomenex luna C18 80*40mm*3 um;mobile phase: [water(0.1%TFA)-ACN];B%: 53%-78%,7min to give **89** (56.57 mg, 89.66 umol, 88.68% yield, 99.59% purity, TFA) as a yellow solid. **^{1}H NMR** (METHANOL-d_4 400MHz) *δ* ppm 8.41 (s, 1 H) 8.23 (s, 1 H) 7.88 (dd, J=8.93, 2.09 Hz, 1 H) 7.59 (s, 1 H) 7.43 (s, 2 H) 6.91 (d, J=8.82 Hz, 1 H) 4.56 (t, J=5.40 Hz, 2 H) 3.80 (t, J=5.40 Hz, 2 H) 3.45 - 3.54 (m, 4 H) 2.72 - 2.79 (m, 4 H). **LCMS** (ESI+): *m/z* 514.2/516.2 (M+H)

Analog 90

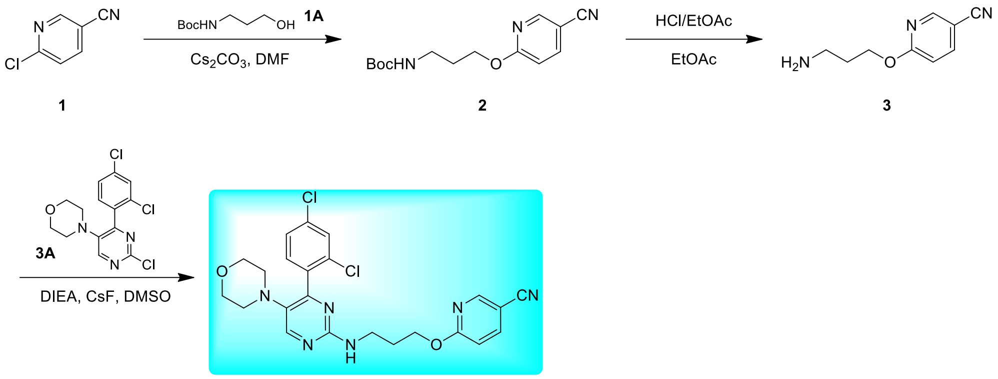

Analog 90

6-(3-((4-(2,4-dichlorophenyl)-5-morpholinopyrimidin-2-yl)amino)propoxy)nicotinonitrile (Analog 90). To a mixture of **Compound 1** (1 g, 7.22 mmol) in DMF (30 mL) was added Cs_2CO_3 (498.59 mg, 1.53 mmol), **Compound 1A** (1.52 g, 8.66 mmol, 1.49 mL) at 25°C, the mixture was stirred at 80°C for 12 hrs. LCMS showed the reaction was completed. The mixture was added to H_2O (50 mL), the aqueous phase was extracted with EtOAc (50 mL * 2). The combined organic phase was washed with brine (50 mL * 2), dried with anhydrous Na_2SO_4, filtered and concentrated in vacuum. The residue was purified by flash silica gel chromatography to give ***Compound 2*** (1 g, 3.61 mmol, 49.96% yield) as white solid. MS-ESI (m/z) calcd for $C_{14}H_{19}N_3O_3$ [M-56+H]: 222.1 Found 222.3. To a stirred solution of **Compound 2** (200 mg, 721.19 umol) in EtOAc (6 mL) was added HCl/EtOAc (4 M, 2.16 mL). The resulting mixture was stirred at 20°C for 1 hr. TLC showed the mixture was completed. The mixture was concentrated to give ***Compound 3*** (130 mg, crude, HCl) as white solid. To a mixture of **Compound 3** (124.00 mg, 580.34 umol, HCl) in DMSO (3 mL) was added DIEA (112.51 mg, 870.52 umol, 151.63 uL), CsF (88.16 mg, 580.34 umol, 21.40 uL) and **Compound 3A** (100 mg, 290.17 umol), the mixture was stirred at 130 °C for 12 hrs. LCMS showed **Compound 3A** was consumed completely and desired mass was detected. The mixture was added to H_2O (5 mL), the aqueous phase was extracted with EtOAc (5 mL * 2). The combined organic phase was washed with brine (5 mL * 2), dried with anhydrous Na_2SO_4, filtered and concentrated in vacuum. The residue was purified by prep-HPLC (column: Waters Xbridge Prep OBD C18 150*40mm*10um; mobile phase: [water (0.05% NH_3H_2O + 10mM NH_4HCO_3) - ACN]; B%: 40%-60%, 8 min) to give ***90*** (19 mg, 37.69 umol, 12.99% yield, 96.28% purity) as yellow solid. ^{1}**H NMR** (METHANOL-d_4 400MHz) δ ppm 8.49 (d, J=2.0 Hz, 1H), 8.19 (s, 1H), 7.91 (dd, J=2.2, 8.7 Hz, 1H), 7.57 (d, J=1.5 Hz, 1H), 7.47 - 7.36 (m, 2H), 6.87 (d, J=8.9 Hz, 1H), 4.47 (t, J=6.0 Hz, 2H), 3.54 (t, J=6.6 Hz, 2H), 3.51 - 3.45 (m, 4H), 2.83 - 2.67 (m, 4H), 2.11 - 2.04 (m, 2H). **LCMS** (ESI+): *m*/*z* 485.2/487.2 (M+H).

Analog 91

THE END

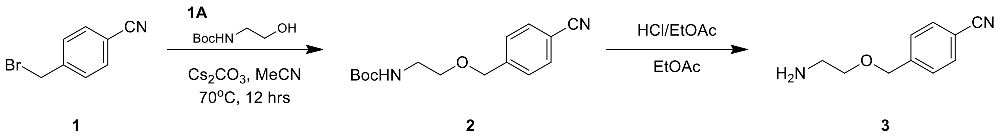

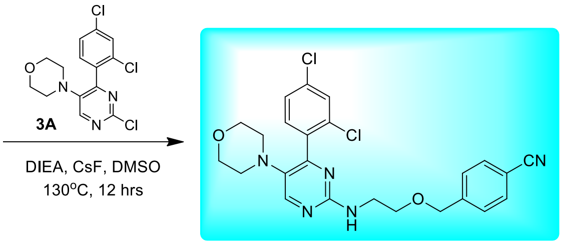

Analog 91

4-((2-((4-(2,4-dichlorophenyl)-5-morpholinopyrimidin-2-yl)amino)ethoxy)methyl)-benzonitrile (Analog 91). To a stirred solution of **Compound 1A** (296.01 mg, 1.84 mmol, 284.63 uL) in MeCN (10 mL) was added Cs_2CO_3 (997.19 mg, 3.06 mmol) and **Compound 1** (300 mg, 1.53 mmol). The resulting mixture was stirred at 70°C for 12 hrs. LCMS showed the reaction was completed. The reaction mixture was quenched by pouring into water 10 mL and extracted with DCM (10 mL * 4), the organic layer was dried over Na_2SO_4, filtered and concentrated under reduced pressure to give a residue. The residue was purified by flash silica gel chromatography to give ***Compound 2*** (350 mg, 1.27 mmol, 82.77% yield) as a white solid.

General procedure for preparation of Compound 3

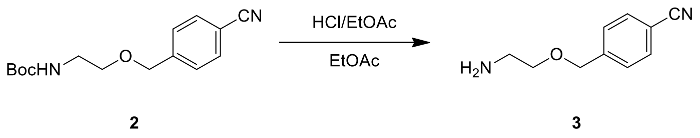

To a stirred solution of **Compound 2** (350 mg, 1.27 mmol) in EtOAc (3 mL) was added HCl/EtOAc (4 M, 7.60 mL). The resulting mixture was stirred at 15°C for 1 hr. LCMS showed the reaction was completed. The reaction mixture was filtered and the filter cake was washed with 5 mL of EtOAc, the filter-cake was dried in vacuum to give ***Compound 3*** (150 mg, crude, HCl) as a white solid. To a stirred solution of **Compound 3** (150 mg, 705.30 umol, HCl) in DMSO (6 mL) was added DIEA (168.76 mg, 1.31 mmol, 227.44 uL), CsF (39.67 mg, 261.15 umol, 9.63 uL) and **Compound 3A** (90 mg, 261.15 umol), the resulting mixture was stirred at 130°C for 12 hrs. LCMS showed the reaction was completed. The reaction mixture was quenched by poured into water 8 mL and extracted with EtOAc (3 mL * 4), dried over Na_2SO_4, filtered and concentrated under reduced pressure to give a residue. The residue was purified by prep-HPLC (column: Waters Xbridge Prep OBD C18 150*40mm*10um; mobile phase: [water (0.05% NH_3H_2O + 10mM NH_4HCO_3) - ACN]; B%: 40%-70%, 8 min) to give ***91*** (23.7 mg, 48.93 umol, 18.74% yield, 100% purity) as a pale yellow solid. ^{1}**H NMR** (METHANOL-d_4 400MHz) δ ppm 8.24 (s, 1H), 7.65 (d, J=8.3 Hz, 2H), 7.58 (d, J=1.9 Hz, 1H), 7.50 (d, J=8.4 Hz, 2H), 7.46 - 7.37 (m, 2H),

4.63 (s, 2H), 3.71 - 3.65 (m, 2H), 3.63 - 3.57 (m, 2H), 3.53 - 3.46 (m, 4H), 2.81 - 2.72 (m, 4H). **LCMS** (ESI+): *m/z* 484.1/486.1 (M+H).

Analog 92

THE END

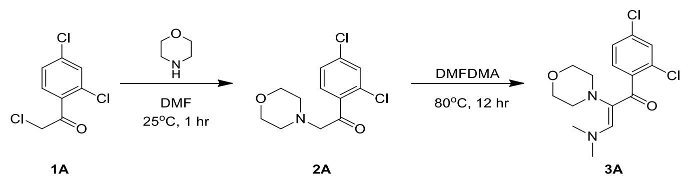

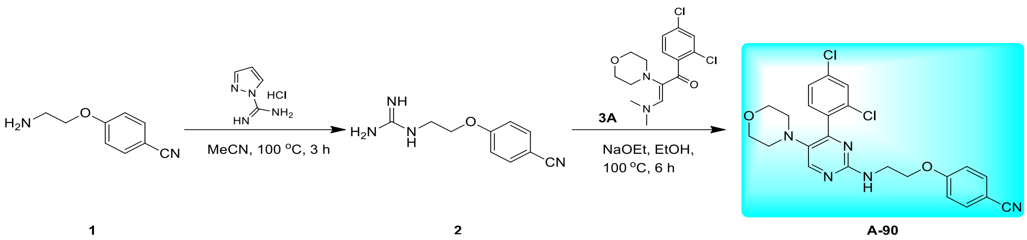

Analog 92

4-(2,4-dichlorophenyl)-*N*-(2-((5-methylpyridin-2-yl)oxy)ethyl)-5-morpholinopyrimidin-2-amine (Analog 92). To a solution of **Compound 1A** (2.39 g, 9.90 mmol) in DMF (20 mL) was added NaH (719.89 mg, 18.00 mmol, 60% purity) at 0°C, after stirred for 1 hr, **Compound 1** (1 g, 9.00 mmol, 934.58 uL) was added. The mixture was stirred at 80°C for 12 hrs. LCMS showed the mixture was completed. The reaction mixture was quenched by poured into water 30 mL and extracted with EtOAc (10 mL * 3). The combined organic layers were washed with brine (10 mL * 2), dried over Na_2SO_4, filtered and concentrated under reduced pressure to give a residue, which was purified by column chromatography to give ***Compound 2*** (0.72 g, 2.35 mmol, 85.35% yield) as a yellow solid. MS-ESI (m/z) calcd for $C_{22}H_{24}N_2O$ $[M+H]^+$: 333.2 Found 333.4. To a solution of **Compound 2** (0.5 g, 1.50 mmol) in THF (5 mL) was added Pd/C (200 mg, 10% purity). The mixture was stirred under H_2 (15Psi) at 15°C for 12 hrs. LCMS showed the mixture was completed. The reaction mixture was quenched by poured into water 30 mL and extracted with EtOAc (10 mL * 3). The combined organic layers were washed with brine (10 mL * 2), dried over Na_2SO_4, filtered and concentrated under reduced pressure to give ***Compound 3*** (200 mg, crude) as a colorless oil. MS-ESI (m/z) calcd for $C_8H_{12}N_2O$ $[M+H]^+$: 153.1 Found 153.3. To a solution of **Compound 3** (100 mg, 657.06 umol) in DMSO (4 mL) was added DIEA (67.94 mg, 525.65 umol, 91.56 uL) and CsF (59.89 mg, 394.24 umol, 14.54 uL), then **Compound 3A** (90.58 mg, 262.82 umol) was added. The mixture was stirred at 130°C for 12 hrs. LCMS showed the mixture was completed. The reaction mixture was poured into water 5 mL and extracted with EtOAc (2 mL * 3). The combined organic layers were washed with brine (2 mL * 2), dried over Na_2SO_4, filtered and concentrated under reduced pressure to give a residue which was purified by prep-HPLC (column: Phenomenex Luna C18 75*30mm*3um; mobile phase: [water (0.2%FA) - ACN]; B%: 40%-80%, 8 min) to give ***92*** (20 mg, 42.38 umol, 16.13% yield, 97.56% purity) as a yellow solid. **^{1}H NMR** (METHANOL-d_4 400MHz) δ ppm 8.24 (s, 1H), 7.88 (s, 1H), 7.58 (s, 1H), 7.48 (dd, J=2.3, 8.6 Hz, 1H), 7.42 (s, 2H), 6.71 (d, J=8.6 Hz, 1H), 4.38 (t, J=5.6 Hz, 2H), 3.75 (t, J=5.6 Hz, 2H), 3.54 - 3.46 (m, 4H), 2.80 - 2.72 (m, 4H), 2.23 (s, 3H). **LCMS** (ESI+): *m/z* 460.0/462.0 (M+H).

Analog 93

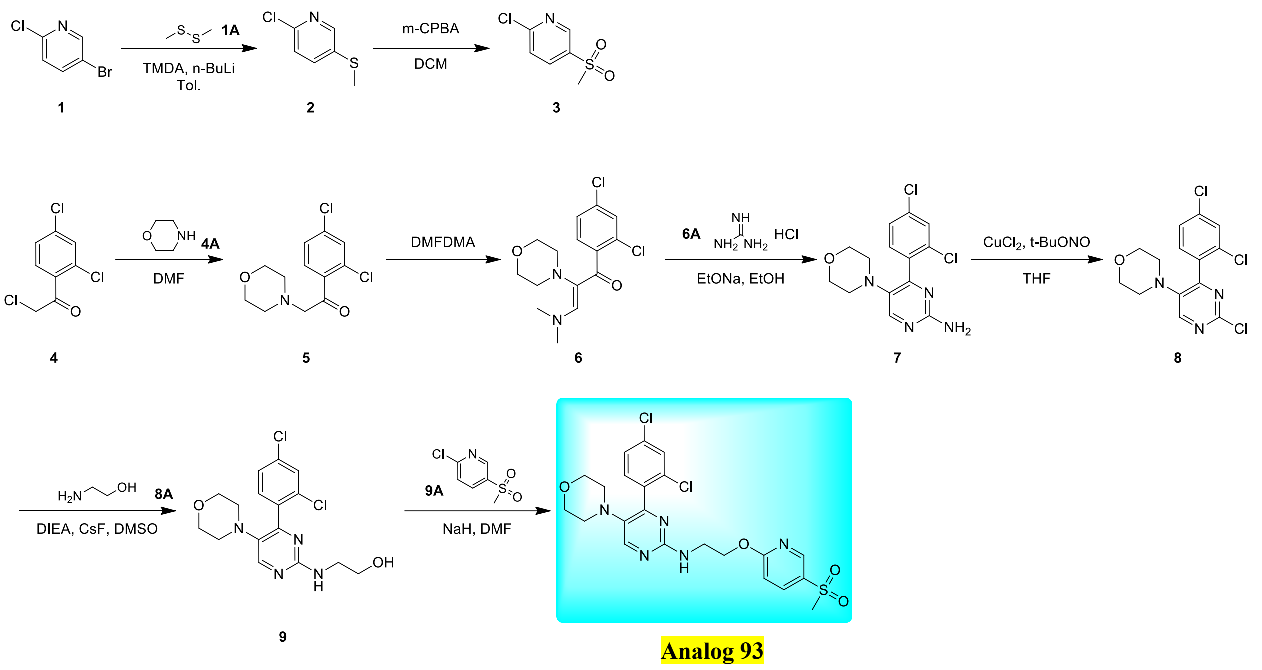

4-(2,4-dichlorophenyl)-*N*-(2-((5-(methylsulfonyl)pyridin-2-yl)oxy)ethyl)-5-morpholinopyrimidin-2-amine (Analog 93). To a solution of **Compound 1** (1 g, 5.20 mmol) and TMEDA (785.00 mg, 6.76 mmol, 1.02 mL) in Tol. (60 mL) at -75°C under N_2 was added n-BuLi (2.5 M, 2.49 mL) over 10 min, and the mixture stirred for 50 min before **Compound 1A** (587.39 mg, 6.24 mmol, 559.42 uL) was added. The reaction was stirred for 1 hr at -75°C. LCMS showed **Compound 1** was consumed completely and desired mass was detected. The mixture warmed to room temperature and quenched with saturated NH_4Cl solution (10 mL). The organic phase was collected, washed with brine, dried Na_2SO_4 and the solvent was removed under vacuum to give a residue. The residue was purified by flash silica gel chromatography (ISCO®; 10g SepaFlash® Silica Flash Column, Eluent of 0~10% Ethyl acetate/Petroleum ether gradient @ 80mL/min) to afford ***Compound 2*** (600 mg, 3.76 mmol, 72.33% yield) as a yellow oil. MS-ESI (m/z) calcd for C_6H_6ClNS $[M+H]^+$: 160.1/162.1 Found 160.2/162.1. To a stirred solution of **Compound 2** (300 mg, 1.88 mmol) in DCM (8 mL) was added m-CPBA (1.14 g, 5.64 mmol, 85% purity) portion wise at -20 °C and the reaction mixture was stirred at same temperature for 1 hr. LCMS showed **Compound 2** was consumed completely and desired mass was detected. The reaction mixture was quenched with saturated NH_4Cl solution 10 mL and the aqueous layer was extracted with EtOAc (2 * 10 mL). The combined organic layer was dried over anhydrous Na_2SO_4, filtered and concentrated under vacuum to afford ***Compound 3*** (350 mg, crude) as a white solid. A solution of **Compound 4** (20 g, 89.49 mmol), **Compound 4A** (77.97 g, 894.92 mmol, 78.75 mL) in DMF (200 mL). Then the reaction mixture was stirred at 20°C for 12 hrs. LCMS showed **Compound 4** was consumed completely and desired mass was detected. The residue was poured into H_2O 50 mL, then extracted with EtOAc (100 mL * 3). The combined organic layers were dried over Na_2SO_4, filtered and concentrated under reduced pressure to afford ***Compound 5*** (20 g, crude) as a brown oil. MS-ESI (m/z) calcd for $C_{12}H_{13}Cl_2NO_2$ $[M+H]^+$: 274.0/276.0 Found 274.1/276.2. A mixture of **Compound 5** (15 g, 54.72 mmol) in DMFDMA (200 mL) was stirred at 80°C for 5 hrs. LCMS showed **Compound 5** was consumed completely and desired mass was detected. The combined organic phase was washed with brine (20 mL * 2), dried with anhydrous Na_2SO_4, filtered and concentrated in vacuum to afford ***Compound 6*** (12 g, crude) as a brown oil. MS-ESI (m/z) calcd for $C_{15}H_{18}Cl_2N_2O_2$ $[M+H]^+$: 329.1/331.1 Found 329.2/331.2. To a solution of **Compound 6A** (5.01 g, 37.97 mmol, HCl) in EtOH (120 mL) was

added EtONa (6.20 g, 91.12 mmol) and **Compound 6** (10 g, 30.37 mmol) at 0°C. Then the reaction mixture was stirred at 70°C for 12 hrs. LCMS showed **Compound 6** was consumed completely and desired mass was detected. The residue was acidified by H_2O 70 mL, then extracted with EtOAc (30 mL * 3). The combined organic layers were dried over Na_2SO_4, filtered and concentrated under reduced pressure to afford ***Compound 7*** (12 g, crude) as a black solid. MS-ESI (m/z) calcd for $C_{14}H_{14}Cl_2N_4O$ $[M+H]^+$: 325.1/327.1 Found 325.2/327.2. A mixture of $CuCl_2$ (1.98 g, 14.76 mmol), t-BuONO (1.90 g, 18.45 mmol, 2.19 mL) in THF (20 mL) was stirred at 60 °C for 5 min, then **Compound 7** (4 g, 12.30 mmol) was added, the mixture was stirred at 60°C for 1 hr. LCMS showed **Compound 7** was consumed completely and desired mass was detected. The residue was diluted by H_2O 20 mL, then extracted with EtOAc (10 mL * 3). The combined organic layers were dried over Na_2SO_4, filtered and concentrated under reduced pressure to give a residue. The residue was purified by flash silica gel chromatography (ISCO®; 80 g SepaFlash® Silica Flash Column, Eluent of 0~13% Ethyl acetate/Petroleum ether gradient @ 80mL/min) to afford ***Compound 8*** (600 mg, 1.74 mmol, 14.15% yield) as a yellow solid. MS-ESI (m/z) calcd for $C_{14}H_{12}Cl_3N_3O$ $[M+H]^+$: 344.0/346.0 Found 344.2/346.1. To a solution of **Compound 8A** (88.62 mg, 1.45 mmol, 87.75 uL) in DMSO (1 mL) were added DIEA (150.01 mg, 1.16 mmol, 202.17 uL) and CsF (88.16 mg, 580.34 umol, 21.40 uL), then **Compound 8** (0.2 g, 580.34 umol) was added. The mixture was stirred at 130°C for 12 hrs. LCMS showed **Compound 8** was consumed completely and desired mass was detected. The residue was poured into H_2O 4 mL, then extracted with EtOAc (3 mL * 3). The combined organic layers were dried over Na_2SO_4, filtered and concentrated under reduced pressure to give a residue. The residue was purified by prep-HPLC (column: Phenomenex Luna C18 75*30mm*3um; mobile phase: [water (0.2%FA) - ACN]; B%: 1%-40%, 8 min) to afford ***Compound 9*** (70 mg, 189.58 umol, 32.67% yield) as a yellow solid. MS-ESI (m/z) calcd for $C_{16}H_{18}Cl_2N_4O_2$ $[M+H]^+$: 369.1/371.1 Found 369.3/371.2. To a solution of **Compound 9A** (41.52 mg, 216.66 umol) in DMF (2 mL) was added NaH (21.66 mg, 541.65 umol, 60% purity) at 0°C, then **Compound 9** (40 mg, 108.33 umol) was added. The mixture was stirred at 15°C for 2 hrs. LCMS showed **Compound 9** was consumed completely and desired mass was detected. The reaction mixture was quenched by pouring into sat.NH_4Cl 5 mL and extracted with EtOAc (2 mL * 3). The combined organic layers were washed with brine (3 mL * 3), dried over Na_2SO_4, filtered and concentrated under reduced pressure to give a residue. The residue was purified by prep-HPLC (column: Phenomenex Gemini-NX C18 75*30mm*3um; mobile phase: [water (0.05% NH_3H_2O + 10mM NH_4HCO_3) - ACN]; B%: 25%-70%, 8 min) to afford ***93*** (11.2 mg, 21.36 umol, 19.71% yield, 100% purity) as a yellow solid. **^{1}H NMR** (METHANOL-d_4 400MHz) δ ppm 8.52 - 8.36 (m, 2H), 7.75 (dd, J=2.4, 9.0 Hz, 1H), 7.63 (s, 1H), 7.54 - 7.42 (m, 2H), 6.60 (d, J=9.0 Hz, 1H), 4.55 (t, J=5.4 Hz, 2H), 3.83 (t, J=5.3 Hz, 2H), 3.58 - 3.45 (m, 4H), 3.07 (s, 3H), 2.88 - 2.73 (m, 4H). **LCMS** (ESI+): *m/z* 524.2/526.1 (M+H).

Analog 94

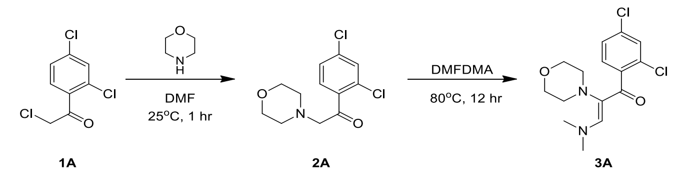

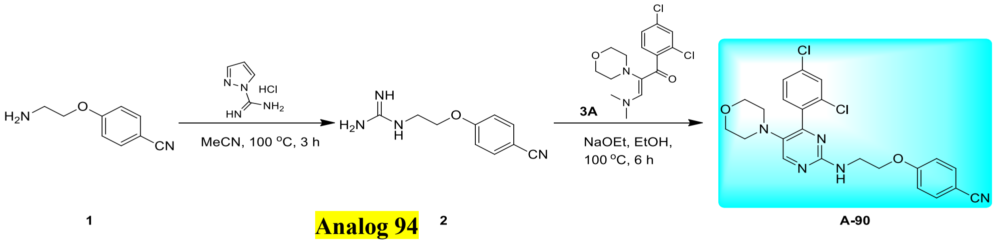

Analog 94

6-(2-((4-(2,4-dichlorophenyl)-5-morpholinopyrimidin-2-yl)amino)ethoxy)-*N*-methylnicotinamide (Analog 94). To a solution of **Compound 1A** (778.04 mg, 3.22 mmol) in DMF (10 mL) was added NaH (234.45 mg, 5.86 mmol, 60% purity) at 0°C, after stirred for 1 hr, **Compound 1** (0.5 g, 2.93 mmol) was added. The mixture was stirred at 80°C for 12 hrs. LCMS showed the reaction was completed. The reaction mixture was poured into water 30 mL and extracted with EtOAc (10 mL * 3). The combined organic layers were washed with brine (10 mL * 2), dried over Na_2SO_4, filtered and concentrated under reduced pressure to give a residue. The residue was purified by column chromatography (SiO_2, Petroleum ether/Ethyl acetate=10/1 to 1/1) to give ***Compound 2*** (0.8 g, 2.13 mmol, 72.70% yield) as a colorless oil. A mixture of **Compound 2** (0.6 g, 1.60 mmol), Pd/C (200 mg, 1.60 mmol, 10% purity) in THF (20 mL) was degassed and purged with H_2 for 3 times, and then the mixture was stirred at 15°C for 13 hrs under H_2 (15 Psi) atmosphere. LCMS showed the reaction was completed. The suspension was filtered through a pad of Celite and the pad was washed with THF (10 mL * 3). The combined filtrates were concentrated under vacuum to give ***Compound 3*** (0.3 g, crude) as a colorless oil and it was used directly. To a solution of **Compound 3** (99.13 mg, 507.80 umol) in DMSO (4 mL) was added DIEA (78.76 mg, 609.36 umol, 106.14 uL) and CsF (30.85 mg, 203.12 umol, 7.49 uL), then **Compound 3A** (70 mg, 203.12 umol) was added. The mixture was stirred at 130°C for 12 hrs. LCMS showed the reaction was completed. The reaction mixture was poured into water 15 mL and extracted with EtOAc (5 mL * 3). The combined organic layers were dried over Na_2SO_4, filtered and concentrated under reduced pressure to give a residue. The residue was purified by prep-HPLC (column: Phenomenex Luna C18 75*30mm*3um; mobile phase: [water (0.2%FA) - ACN]; B%: 25%-60%, 8 min) to give ***94*** (32.5 mg, 64.56 umol, 31.79% yield, 100% purity) as a yellow solid. **^{1}H NMR** (METHANOL-d_4 400MHz) δ ppm 8.55 (d, J=2.4 Hz, 1H), 8.23 (s, 1H), 8.02 (dd, J=2.5, 8.7 Hz, 1H), 7.57 (d, J=1.6 Hz, 1H), 7.45 - 7.37 (m, 2H), 6.82 (d, J=8.7 Hz, 1H), 4.52 (t, J=5.5 Hz, 2H), 3.78 (t, J=5.5 Hz, 2H), 3.52 - 3.45 (m, 4H), 2.90 (s, 3H), 2.79 - 2.72 (m, 4H). **LCMS** (ESI+): *m/z* 503.1/505.1 (M+H)

Analog 95

THE END

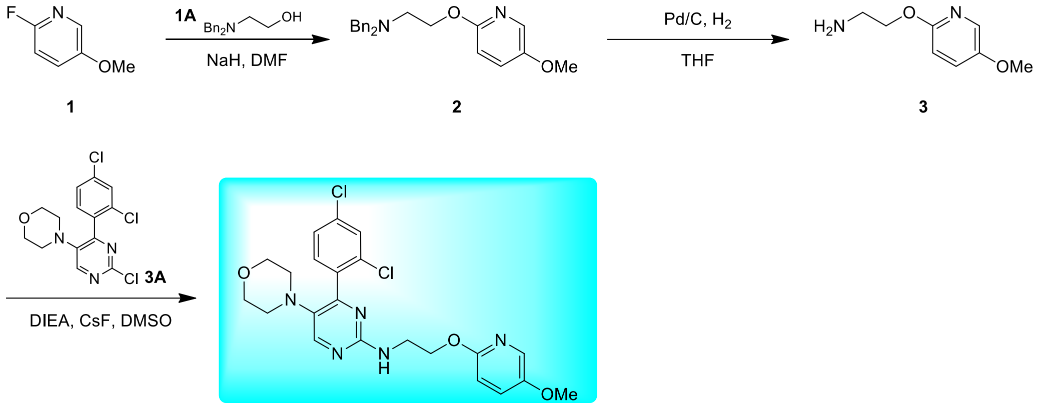

Analog 95

4-(2,4-dichlorophenyl)-*N*-(2-((5-methoxypyridin-2-yl)oxy)ethyl)-5-morpholinopyrimidin-2-amine (Analog 95). To a solution of **Compound 1A** (626.50 mg, 2.60 mmol) in DMF (10 mL) was added NaH (188.80 mg, 4.72 mmol, 60% purity) at 0°C, after stirred for 1 hr, **Compound 1** (0.3 g, 2.36 mmol) was added. The mixture was stirred at 80°C for 12 hrs. LCMS showed the reaction was completed. The reaction mixture was quenched by poured into water 30 mL and extracted with EtOAc (10 mL * 3). The combined organic layers were washed with brine (10 mL * 2), dried over Na_2SO_4, filtered and concentrated under reduced pressure to give a residue. The residue was purified by column chromatography (SiO_2, Petroleum ether/Ethyl acetate=5/1) to give ***Compound 2*** (0.70 g, crude) as a white solid. A mixture of **Compound 2** (0.15 g, 430.49 umol), Pd/C (50 mg, 430.49 umol, 10% purity) in THF (10 mL) was degassed and purged with H_2 for 3 times, and then the mixture was stirred at 15 °C for 1 hr under H_2 (15 Psi) atmosphere. LCMS showed the reaction was completed. The suspension was filtered through a pad of Celite and the pad was washed with THF (10 mL * 3). The combined filtrates were concentrated under vacuum to give ***Compound 3*** (50 mg, crude) as a white solid. To a solution of **Compound 3** (109.8 mg, 536.58 umol) in DMSO (6 mL) was added DIEA (101.25 mg, 783.45 umol, 136.47 uL) and CsF (39.66 mg, 261.15 umol, 9.63 uL), then **Compound 3A** (90 mg, 261.15 umol) was added. The mixture was stirred at 130°C for 12 hrs. LCMS showed the reaction was completed. The reaction mixture was poured into water 2 mL and extracted with EtOAc (2 mL * 3), dried over Na_2SO_4, filtered and concentrated under reduced pressure to give a residue**.** The residue was purified by prep-HPLC (column: Waters Xbridge Prep OBD C18 150*40mm*10um; mobile phase: [water (0.05% NH_3H_2O + 10mM NH_4HCO_3) - ACN]; B%: 40%-70%, 8 min) to give ***95*** (51.4 mg, 108.18 umol, 41.32% yield, 99.65% purity) as a yellow solid. **^{1}H NMR** (METHANOL-d_4 400MHz) δ ppm 8.24 (s, 1H), 7.73 (d, J=3.0 Hz, 1H), 7.59 - 7.56 (m, 1H), 7.43 - 7.41 (m, 2H), 7.30 (dd, J=3.1, 8.9 Hz, 1H), 6.75 (d, J=8.9 Hz, 1H), 4.37 (t, J=5.6 Hz, 2H), 3.74 (t, J=5.6 Hz, 2H), 3.51 - 3.48 (m, 4H), 3.31 (s, 3H), 2.81 - 2.71 (m, 4H). **LCMS** (ESI+): *m/z* 476.2/478.2 (M+H)

Analog 96

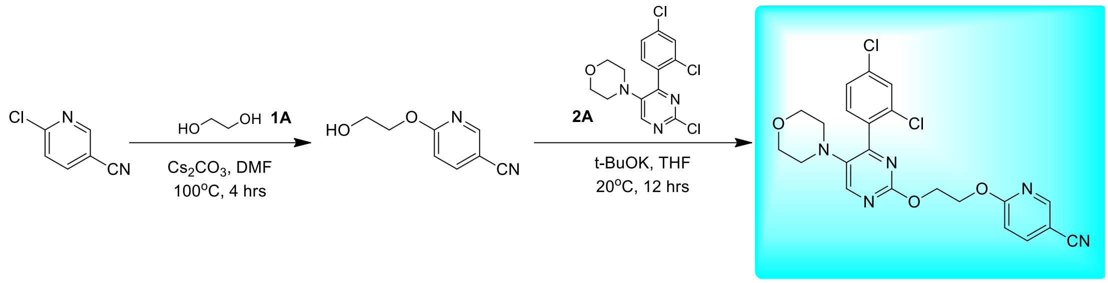

1 2 Analog 96

6-(2-((4-(2,4-dichlorophenyl)-5-morpholinopyrimidin-2-yl)oxy)ethoxy)nicotinonitrile (Analog 96). A mixture of **Compound 1** (1 g, 7.22 mmol), **Compound 1A** (895.93 mg, 14.43 mmol, 807.15 uL), Cs_2CO_3 (9.41 g, 28.87 mmol) in DMF (15 mL), and then the mixture was stirred at 100°C for 3 hrs. TLC indicated **Compound 1** was consumed completely. The reaction mixture was concentrated under reduced pressure to remove solvent. The residue was diluted with H_2O 15 mL and extracted with EtOAc (15 mL * 3). The combined organic layers were dried over anhydrous Na_2SO_4, filtered and concentrated under reduced pressure to give a residue. The residue was purified by flash silica gel chromatography (ISCO®; 4 g SepaFlash® Silica Flash Column, Eluent of 0~50% Ethyl acetate/Petroleum ether gradient @ 18 mL/min) (Petroleum ether : Ethyl acetate=5:1) (P1 Rf=0.24) to afford ***Compound 2*** (840 mg, 5.12 mmol, 70.90% yield) as a yellow solid. MS-ESI (m/z) calcd for $C_8H_8N_2O_2$ $[M+H]^+$: 165.1 Found 165.2. To a solution of **Compound 2** (28.58 mg, 174.10 umol) in DMF (1.5 mL) was added NaH (8.70 mg, 217.63 umol, 60% purity) at 20°C, then the mixture was stirred at 20°C for 0.5 hr, then **Compound 2A** (50 mg, 145.09 umol) was added at 20°C, then the mixture was stirred at 20°C for 12 hrs. LCMS showed **Compound 2** was consumed and 23% of desired product was detected. The reaction was added 1 mL MeOH, the mixture was filtered. The filtrate was purified by Prep-HPLC (neutral condition) (column: Waters Xbridge BEH C18 100*30mm*10um; mobile phase: [water (10mM NH_4HCO_3) - ACN]; B%: 60%-90%, 8 min) to afford ***96*** (11.6 mg, 24.05 umol, 16.57% yield, 97.91% purity) as a white solid. **^{1}H NMR** (METHANOL-d_4 400MHz) δ ppm 8.50 (d, J=1.63 Hz, 1 H) 8.45 (s, 1 H) 7.92 (dd, J=8.76, 2.38 Hz, 1 H) 7.63 (d, J=1.25 Hz, 1 H) 7.42 - 7.54 (m, 2 H) 6.90 (d, J=8.63 Hz, 1 H) 4.69 - 4.80 (m, 4 H) 3.48 - 3.58 (m, 4 H) 2.77 - 2.88 (m, 4 H). **LCMS** (ESI+): *m/z* 472.2/474.1 (M+H).

Analog 97

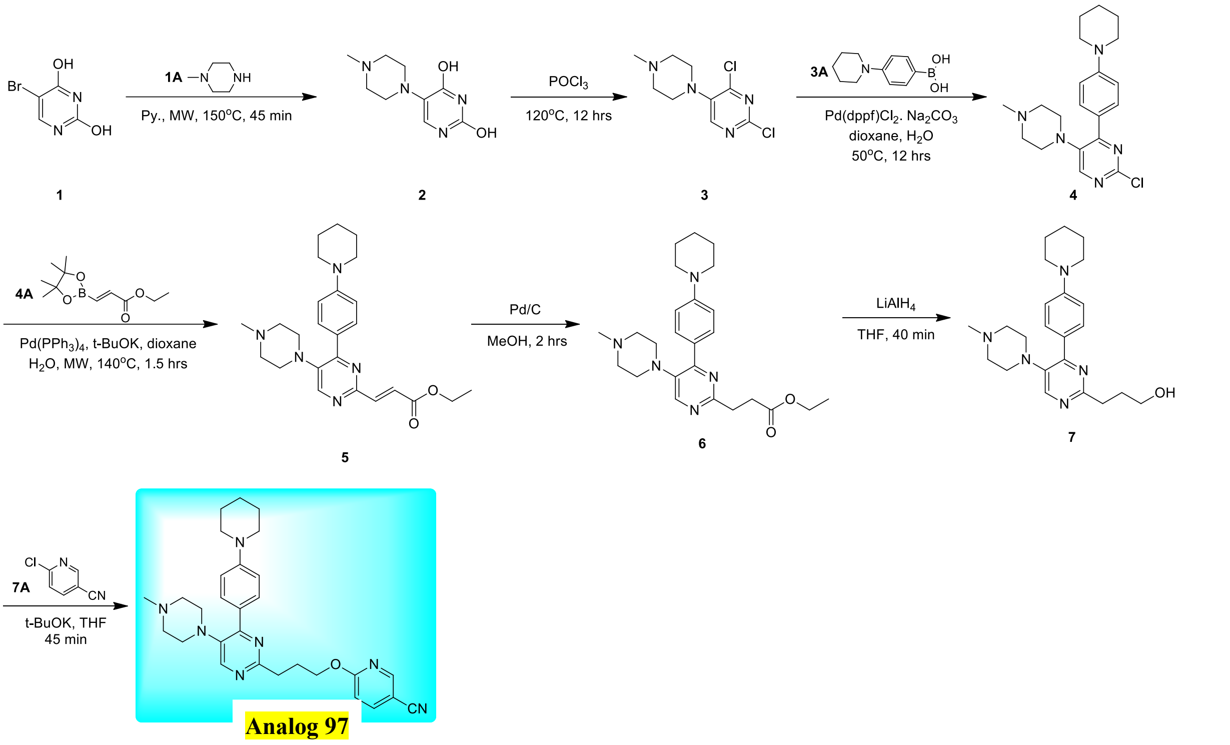

6-(3-(5-(4-methylpiperazin-1-yl)-4-(4-(piperidin-1-yl)phenyl)pyrimidin-2-yl)propoxy)-nicotinonitrile (Analog 97). To a solution of **Compound 1** (2 g, 10.47 mmol) in Py. (10 mL) was added **Compound 1A** (1.57 g, 15.71 mmol, 1.74 mL) at 20°C, then the mixture was stirred at 150°C for 45 min under microwave. LCMS showed **Compound 1** was consumed and a main peak with desired product was detected. The reaction was concentrated under vacuum. The residue was diluted with MeOH (100 mL) and the mixture was stirred at 20°C for 30 min, then the mixture was filtered and the filter-cake was dried under vacuum to afford ***Compound 2*** (3.35 g, crude) as a gray solid and it was used directly. MS-ESI (m/z) calcd for $C_9H_{14}N_4O_2$ $[M+H]^+$: 211.1 Found 211.3 **^{1}H NMR** DMSO-d6 400MHz δ ppm 11.07 (br s, 1 H) 10.49 (br s, 1 H) 6.73 (s, 1 H) 2.98 - 3.31 (m, 3 H) 2.83 (br s, 5 H) 2.25 (s, 3 H). **Compound 2** (5.05 g, 24.02 mmol) was added into $POCl_3$ (82.50 g, 538.05 mmol, 50 mL) slowly at 20°C, then the mixture was stirred at 120°C for 12 hrs. LCMS showed **Compound 2** was consumed and 54% of desired product was detected. The reaction was concentrated under vacuum. The residue was diluted with EtOAc (100 mL), the mixture was poured into saturated $NaHCO_3$ solution (100 mL), solid was appeared and the mixture was filtered and the filter-cake was dried under vacuum to afford ***Compound 3*** (520 mg, 2.10 mmol, 8.74% yield) as a yellow solid and it was used directly. MS-ESI (m/z) calcd for $C_9H_{12}Cl_2N_4$ $[M+H]^+$: 247.0/249.0 Found 247.2/249.1

To a solution of **Compound 3** (400 mg, 1.62 mmol) and **Compound 3A** (530 mg, 1.85 mmol) in dioxane (5 mL) and H_2O (0.75 mL) were added Pd(dppf)Cl_2 (118.44 mg, 161.86 umol) and Na_2CO_3 (514.67 mg, 4.86 mmol) at 20°C, then the mixture was stirred at 50°C for 12 hrs under N_2. LCMS showed 8% of **Compound 3** was remained and 25% of desired product was detected. The reaction was concentrated under vacuum. The residue was diluted with EtOAc (8 mL) and MeOH (3 mL), then solid was appeared and the mixture was filtered and the filter-cake was concentrated under vacuum to afford ***Compound 4*** (746 mg, crude) as a yellow solid and it was used directly. MS-ESI (m/z) calcd for $C_{20}H_{26}ClN_5$ $[M+H]^+$: 372.2/374.2 Found 372.3/374.3. To a solution of **Compound 4** (200 mg, 537.77 umol) and **Compound**

4A (145.89 mg, 645.32 umol) in dioxane (2 mL) and H_2O (0.2 mL) were added t-BuOK (120.69 mg, 1.08 mmol) and $Pd(PPh_3)_4$ (62.14 mg, 53.78 umol) at 20°C, then the mixture was stirred at 140°C under N_2 for 1.5 hrs under microwave. LCMS showed **Compound 4** was consumed and 16% of desired product was detected. The reaction was concentrated under vacuum. The residue was purified by Prep-TLC (Dichloromethane : Methanol=10:1) (P1 Rf=0.37) to afford ***Compound 5*** (60 mg, 137.75 umol, 25.62% yield) as a yellow liquid. MS-ESI (m/z) calcd for $C_{25}H_{33}N_5O_2$ $[M+H]^+$: 436.3 Found 436.4. To a solution of **Compound 5** (114 mg, 261.73 umol) in MeOH (5 mL) was added Pd/C (280 mg, 10% purity) at 20°C, then the mixture was stirred at 20°C for 2 hrs under H_2. TLC showed **Compound 5** was consumed and two new spots were detected. The reaction was filtered and the filtrate was concentrated under vacuum to afford ***Compound 6*** (116 mg, crude) as a yellow liquid and it was used directly. MS-ESI (m/z) calcd for $C_{25}H_{35}N_5O_2$ $[M+H]^+$: 438.3 Found 438.2. To a solution of **Compound 6** (116 mg, 265.10 umol) in THF (5 mL) was added $LiAlH_4$ (40.25 mg, 1.06 mmol) at 0°C, then the mixture was stirred at 0°C for 40 min. LCMS showed **Compound 6** was consumed and 41% of desired product was detected. The reaction was diluted with 5 mL THF and then 500 mg $Na_2SO_4.10H_2O$ was added in portions at 0°C, then the mixture was stirred at 20°C for 12 hrs. The mixture was filtered and the filtrate was concentrated under vacuum to afford ***Compound 7*** (121 mg, crude) as a yellow liquid and it was used directly. MS-ESI (m/z) calcd for $C_{23}H_{33}N_5O$ $[M+H]^+$: 396.3 Found 396.4

To a solution of **Compound 7** (60 mg, 151.69 umol) in THF (2 mL) was added t-BuOK (51.06 mg, 455.07 umol) at 0°C, then the mixture was stirred at 0°C for 5 min, then **Compound 7A** (37.04 mg, 303.38 umol) was added at 0°C, then the mixture was stirred at 0°C for 40 min. LCMS showed **Compound 7** was consumed and 34% of desired product was detected. The reaction was concentrated under vacuum. The residue was purified by Prep-HPLC (TFA condition) (column: Phenomenex luna C18 100*40mm*5 um; mobile phase: [water (0.1% TFA) - ACN]; B%: 5%-30%, 8 min) to afford ***97*** (21.36 mg, 34.18 umol, 22.53% yield, 97.87% purity, TFA) as a yellow gum. **^{1}H NMR** (METHANOL-d_4 400MHz) δ ppm 8.42 - 8.48 (m, 2 H) 8.18 (d, J=9.01 Hz, 2 H) 7.89 (dd, J=8.76, 2.38 Hz, 1 H) 7.35 (br d, J=8.76 Hz, 2 H) 6.78 (d, J=8.63 Hz, 1 H) 4.50 (t, J=6.13 Hz, 2 H) 3.45 - 3.59 (m, 6 H) 3.40 (br d, J=12.63 Hz, 2 H) 3.19 - 3.28 (m, 2 H) 3.01 - 3.15 (m, 4 H) 2.97 (s, 3 H) 2.36 (t, J=6.57 Hz, 2 H) 1.86 (br d, J=4.63 Hz, 4 H) 1.76 (br d, J=5.00 Hz, 2 H). **LCMS** (ESI+): *m/z* 498.2 (M+H)

Analog 98

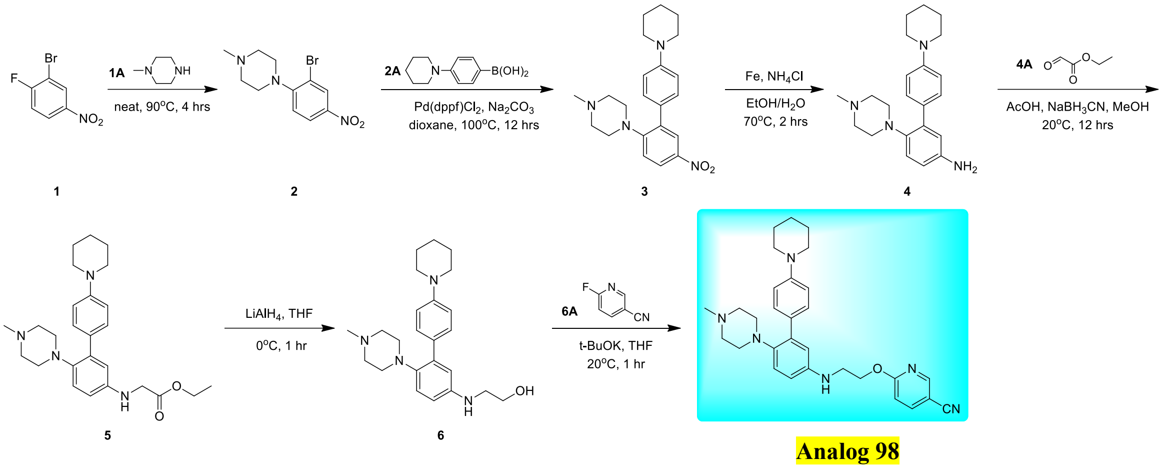

Analog 98

6-(2-((6-(4-methylpiperazin-1-yl)-4'-(piperidin-1-yl)-[1,1'-biphenyl]-3-yl)amino)ethoxy)-nicotinonitrile (Analog 98). A mixture of **Compound 1** (5 g, 22.73 mmol) and **Compound 1A** (22.77 g, 227.30 mmol, 25.21 mL) was stirred at 90°C for 4 hrs. TLC showed **Compound 1** was consumed and a new spot was formed. The reaction was cooled to room temperature, then 40 mL H_2O was added into the reaction, then solid was appeared and the mixture was filtered and the filter-cake was dried under vacuum to afford ***Compound 2*** (6.55 g, crude) as a yellow solid and it was used to next step directly. **^{1}H NMR** METHANOL-d4 400MHz δ ppm 8.42 (d, J=2.50 Hz, 1 H) 8.19 (dd, J=8.88, 2.50 Hz, 1 H) 7.25 (d, J=8.88 Hz, 1 H) 3.24 (br s, 4 H) 2.67 (br s, 4 H) 2.38 (s, 3 H). To a solution of **Compound 2** (500 mg, 1.67 mmol) in dioxane (16 mL) and H_2O (2.4 mL) and **Compound 2A** (342.45 mg, 1.67 mmol) was added Pd(dppf)Cl_2 (122.20 mg, 167.00 umol) and Na_2CO_3 (531.01 mg, 5.01 mmol) at 20°C. The mixture was stirred at 50°C for 12 hrs under N_2. LCMS showed **Compound 2** was consumed completely and 79% of desired compound was detected. The reaction mixture was concentrated under reduced pressure to remove solvent. The residue was diluted with H_2O 20 mL and extracted with EtOAc (20 mL * 3). The combined organic layers were dried over anhydrous Na_2SO_4, filtered and concentrated under vacuum to afford ***Compound 3*** (720 mg, crude) as a yellow oil. MS-ESI (m/z) calcd for $C_{22}H_{28}N_4O_2$ $[M+H]^+$: 381.2 Found 381.3. To a solution of **Compound 3** (720 mg, 1.89 mmol) in EtOH (12 mL) and H_2O (3 mL) was added Fe (528.39 mg, 9.46 mmol) and NH_4Cl (506.12 mg, 9.46 mmol) at 20°C. The mixture was stirred at 70°C for 2 hrs. LCMS showed **Compound 3** was consumed completely and 76.6% of desired compound was detected. The reaction mixture was filtered. The residue was purified by flash silica gel chromatography (ISCO®; 12 g SepaFlash ® Silica Flash Column, Eluent of 0~100% Ethyl acetate/Petroleum ether gradient @ 80 mL/min) (SiO_2, Dichloromethane : Methanol=10:1, Rf(P1)=0.30) to afford ***Compound 4*** (280 mg, 663.05 umol, 35.04% yield, 83% purity) as a yellow solid. MS-ESI (m/z) calcd for $C_{22}H_{30}N_4$ $[M+H]^+$: 351.3 Found 351.3. A solution of **Compound 4** (145 mg, 413.70 umol) and **Compound 4A** (126.70 mg, 620.54 umol, 50% purity) in AcOH (2 mL) and MeOH (0.3 mL) was stirred at 20°C for 1.5 hrs, then $NaBH_3CN$ (64.99 mg, 1.03 mmol) was added at 20°C, then the mixture was stirred at 20°C for 12 hrs. LCMS showed 9% of **Compound 4** was remained and 72% of desired product was detected. The reaction was basified with saturated Na_2CO_3 solution to pH = 8, then the mixture was extracted with DCM : MeOH (10:1) (6 mL * 3), the organic layer was dried over anhydrous Na_2SO_4, filtered and the filtrate was concentrated under vacuum to afford ***Compound 5*** (201 mg, crude) as a brown liquid and it was used directly. MS-ESI (m/z) calcd for $C_{26}H_{36}N_4O_2$ $[M+H]^+$: 437.3 Found 437.4. To a solution of **Compound 5** (201 mg, 460.39 umol) in THF (5 mL) was added $LiAlH_4$ (20.97 mg, 552.47 umol) at 0°C, then the mixture was stirred at 0°C for 1 hr. LCMS showed trace of **Compound 5** was remained and 65% of desired product was detected.

The reaction was quenched by saturated $MgSO_4$ solution (0.5 mL) slowly at 0°C, then Na_2SO_4 solid was added and the mixture was stirred at 20°C for 10 min, then the mixture was filtered and the filtrate was concentrated under vacuum. The residue was purified by Prep-TLC (Dichloromethane : Methanol=10:1) (P1 Rf=0.31) to afford ***Compound 6*** (58 mg, 147.00 umol, 31.93% yield) as a brown liquid. MS-ESI (m/z) calcd for $C_{24}H_{34}N_4O$ $[M+H]^+$: 395.3 Found 395.2. To a solution of **Compound 6** (58 mg, 147.00 umol) in THF (3 mL) was added t-BuOK (49.49 mg, 441.01 umol) at 0°C, then the mixture was stirred at 0°C for 5 min, then **Compound 6A** (35.90 mg, 294.00 umol) was added at 0°C, then the mixture was stirred at 0°C for 1 hr. LCMS showed trace of **Compound 6** was remained and 28% of desired product was detected. The residue was concentrated under vacuum. The residue was purified by Prep-HPLC (TFA condition, column: Phenomenex luna C18 100*40mm*5 um ; mobile phase: [water (0.1% TFA) - ACN] ; B%: 1%-30%, 8 min) to afford ***98*** (41.79 mg, 67.45 umol, 45.89% yield, 98.57% purity, TFA) as a pale yellow gum. **^{1}H NMR** (METHANOL-d_4 400MHz) δ ppm 8.48 (d, J=2.21 Hz, 1 H) 7.97 (dd, J=8.71, 2.32 Hz, 1 H) 7.70 - 7.82 (m, 4 H) 7.16 - 7.22 (m, 1 H) 7.06 - 7.12 (m, 1 H) 6.92 - 7.01 (m, 2 H) 4.60 (t, J=5.29 Hz, 2 H) 3.63 - 3.74 (m, 6 H) 3.39 (br d, J=9.04 Hz, 2 H) 3.05 - 3.18 (m, 2 H) 2.91 - 3.04 (m, 4 H) 2.88 (s, 3 H) 2.08 (quin, J=5.62 Hz, 4 H) 1.83 (br d, J=5.29 Hz, 2 H). **LCMS** (ESI+): *m/z* 497.2 (M+H).

Analog 99

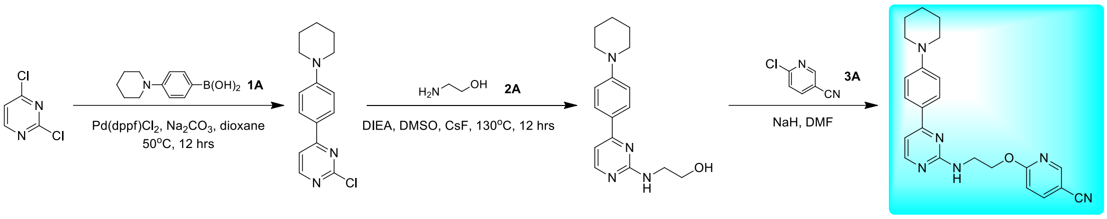

1 2 3 **Analog 99**

6-(2-((4-(4-(piperidin-1-yl)phenyl)pyrimidin-2-yl)amino)ethoxy)nicotinonitrile (Analog 99). To a solution of **Compound 1** (150 mg, 1.01 mmol) and **Compound 1A** (206.47 mg, 1.01 mmol) in dioxane (4 mL) and H_2O (0.6 mL) were added Pd(dppf)Cl_2 (73.67 mg, 100.69 umol) and Na_2CO_3 (320.15 mg, 3.02 mmol) at 20°C. The mixture was stirred at 50°C for 12 hrs under N_2. LCMS showed **Compound 1** was consumed completely and 42.7% of desired compound was detected. The reaction mixture was concentrated under reduced pressure to remove solvent. The residue was purified by flash silica gel chromatography (ISCO®; 4g SepaFlash® Silica Flash Column, Eluent of 0~20% Ethyl acetate/Petroleum ether gradient @ 100/min) (SiO_2, Petroleum ether : Ethyl acetate=5:1, Rf(P1)=0.23) to afford ***Compound 2*** (195 mg, 712.30 umol, 70.74% yield) as a yellow solid. MS-ESI (m/z) calcd for $C_{15}H_{16}ClN_3$ $[M+H]^+$: 274.1/276.1 Found 274.2/276.2. To a solution of **Compound 2** (90 mg, 328.76 umol) and **Compound 2A** (50.20 mg, 821.89 umol, 49.71 uL) in DMSO (2 mL) was added DIEA (127.47 mg, 986.27 umol, 171.79 uL) and CsF (74.91 mg, 493.13 umol, 18.18 uL) at 20°C. The mixture was stirred at 130°C for 12 hrs. LCMS showed **Compound 2** was consumed completely and 50.8% of desired compound was detected. The reaction mixture was diluted with H_2O 4 mL and extracted with EtOAc (4 mL * 3). The combined organic layers were washed with brine (4 mL * 1), dried over anhydrous Na_2SO_4, filtered and concentrated under reduced pressure to give a residue to afford ***Compound 3*** (52 mg, crude) as a yellow solid. MS-ESI (m/z) calcd for $C_{17}H_{22}N_4O$ $[M+H]^+$: 299.2 Found 299.3. To a solution of **Compound 3** (52 mg, 174.27 umol) in DMF (2 mL) was added NaH (8.36 mg, 209.13 umol, 60% purity). The mixture was stirred at 0°C for 10 min, then **Compound 3A** (28.98 mg, 209.13 umol) was added at 0°C, the mixture was stirred at 20°C for 2 hrs. LCMS and HPLC showed 12% of **Compound 3** remained and 37% of desired compound was detected. The reaction mixture was filtered. The residue was purified by prep-HPLC (TFA condition) (column: Phenomenex luna C18 100*40mm*5 um; mobile phase: [water (0.1% TFA) - ACN]; B%: 20%-44%, 8 min) to afford ***99*** (17.4 mg, TFA, 100% purity,17.10% yield) as a yellow solid. **^{1}H NMR** (METHANOL-d_4 400MHz) δ ppm 8.57 (d, J=2.19 Hz, 1 H) 8.05 (br d, J=6.58 Hz, 1 H) 7.97 (br d, J=8.55 Hz, 2 H) 7.84 (br s, 1 H) 7.30 (d, J=6.80 Hz, 1 H) 7.00 (br d, J=8.55 Hz, 2 H) 6.82 (br d, J=9.21 Hz, 1 H) 4.70 (t, J=5.26 Hz, 2 H) 4.06 (br s, 2 H) 3.52 (br s, 4 H) 1.72 (br s, 6 H). **LCMS** (ESI+): *m/z* 401.1 (M+H)

Analog 101, 102

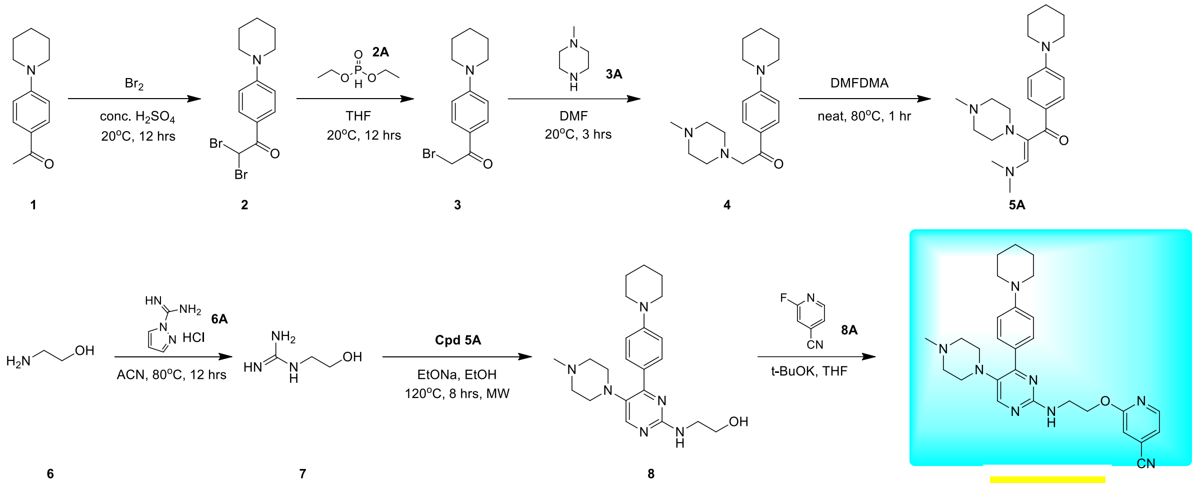

Analog 101

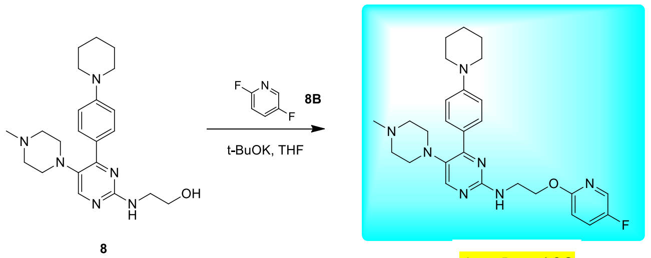

Analog 102

2-(2-((5-(4-methylpiperazin-1-yl)-4-(4-(piperidin-1-yl)phenyl)pyrimidin-2-yl)amino)ethoxy)isonicotinonitrile (Analog 101). To a solution of **Compound 1** (5 g, 24.60 mmol) in conc. H_2SO_4 (20 mL, 98% purity) was added Br_2 (5.90 g, 36.89 mmol, 1.90 mL) at 0°C. The mixture was stirred at 20°C for 12 hrs. LCMS showed **Compound 1** was consumed completely and one main peak with desired mass was detected. The reaction mixture was poured into ice-water slowly and the temperature was maintained at 0°C, solid was appeared, the mixture was filtered and the filter-cake was dried under vacuum to afford ***Compound 2*** (7.38 g, crude) as a yellow solid. MS-ESI (m/z) calcd for $C_{13}H_{15}Br_2NO$ $[M+H]^+$: 362.1/360.1/364.1. Found 361.9/359.9/364.0. To a solution of **Compound 2** (7.38 g, 20.44 mmol) in THF (80mL) was added another solution of **Compound 2A** (2.96 g, 21.46 mmol, 2.77 mL) and TEA (2.17 g, 21.46 mmol, 2.99 mL) in THF (20 mL) at 0°C. The mixture was stirred at 20°C for 12 hrs. LCMS showed **Compound 2** was consumed completely and one main peak with desired mass was detected. The reaction was concentrated under vacuum. The residue was poured into ice-water, yellow solid was appeared, then the mixture was filtered and the filter-cake was dried under vacuum to afford ***Compound 3*** (3.94 g, crude) as a yellow solid. MS-ESI (m/z) calcd for $C_{13}H_{16}BrNO$ $[M+H]^+$: 282.0/284.0. Found 282.2/284.2. To a solution of **Compound 3** (3.94 g, 13.96 mmol) in DMF (60 mL) was added **Compound 3A** (13.99 g, 139.63 mmol, 15.49 mL) at 20°C. The mixture was stirred at 20°C for 3 hrs. LCMS showed **Compound 3** was consumed completely and one main peak with desired mass was detected. The reaction mixture was concentrated under reduced pressure to remove DMF. The residue was diluted with H_2O 60 mL and extracted with EtOAc (60 mL × 3). The combined organic layers were dried over anhydrous Na_2SO_4, filtered and concentrated under reduced pressure to give a residue. The residue was purified by flash silica gel chromatography (ISCO®; 20 g SepaFlash® Silica Flash Column, Eluent of 0~70% Ethyl acetate/Petroleum ether gradient @ 100 mL/min) (SiO_2,.Dichloromethane : Methanol=10:1, Rf(P1)=0.10) to afford ***Compound 4*** (1.26 g, 4.18 mmol, 14.97% yield) as a yellow solid. MS-ESI (m/z) calcd for $C_{18}H_{27}N_3O$ $[M+H]^+$: 302.2. Found 302.4. A solution

of **Compound 4** (2.25 g, 7.46 mmol) in DMFDMA (17.26 g, 99.05 mmol, 20.45 mL) at 20°C. The mixture was stirred at 80°C for 1 hr. LC-MS showed **Compound 4** was consumed completely and one main peak with desired mass was detected. The reaction mixture was concentrated under reduced pressure to remove solvent to afford ***Compound 5A*** (2.60 g, crude) as a yellow solid. MS-ESI (m/z) calcd for $C_{21}H_{32}N_4O$ $[M+H]^+$: 357.3. Found 357.4. To a solution of **Compound 6** (5 g, 81.86 mmol, 4.95 mL) in ACN (50 mL) was added **Compound 6A** (12.00 g, 81.86 mmol, HCl) at 20°C. The mixture was stirred at 80°C for 12 hrs. LCMS showed **Compound 6** was consumed completely and 100% of desired compound was detected. The reaction mixture was concentrated under reduced pressure to remove ACN. The residue was purified by prep-HPLC (basic condition, column: Agela DuraShell C18 250*70mm*10um; mobile phase: [water (0.05% ammonia hydroxide v/v) - ACN]; B%: 0%-0%, 8 min) to afford ***Compound 7*** (1 g, 9.70 mmol, 11.85% yield, 100% purity) as a white solid. MS-ESI (m/z) calcd for $C_3H_9N_3O$ $[M+H]^+$: 104.1. Found 104.1. To a solution of **Compound 7** (200.00 mg, 561.00 umol) and **Compound 5A** (200 mg, 1.43 mmol, 4 eq, HCl) in EtOH (3 mL) was added EtONa (146.26 mg, 2.15 mmol) at 20°C. The mixture was stirred in the microwave at 120°C for 8 hrs. LCMS and HPLC showed **Compound 7** was consumed completely and 18% of desired compound was detected. The reaction mixture was filtered. The residue was purified by prep-HPLC (TFA condition, column: Phenomenex Gemini-NX C18 75*30mm*3um; mobile phase: [water (0.1%TFA) - ACN]; B%: 5%-25%, 12 min) to afford ***Compound 8*** (20 mg, 28.60 umol, 7.98% yield, 73% purity, TFA) as a yellow oil. MS-ESI (m/z) calcd for $C_{22}H_{32}N_6O$ $[M+H]^+$: 397.3. Found 397.3. To a solution of **Compound 8** (15 mg, 29.38 umol, TFA) in THF (1 mL) was added t-BuOK (9.89 mg, 88.14 umol). The mixture was stirred at 20°C for 10 min, then **Compound 8A** (10.76 mg, 88.14 umol) was added. The mixture was stirred at 20°C for 1 hr. LCMS and HPLC showed **Compound 8** was consumed completely and 39.5% of desired compound was detected. The reaction mixture was filtered. The residue was purified by prep-HPLC (TFA condition, column: Phenomenex luna C18 100*40mm*5 um; mobile phase: [water (0.1%TFA) - ACN]; B%: 10%-47%, 8 min) to afford ***101*** (11.93 mg, 23.99 umol, 100% purity, 52.23% yield, TFA) as a yellow gum. **^{1}H NMR** (METHANOL-d_4 400MHz) δ ppm 8.29 (d, J=5.14 Hz, 1 H) 8.23 (d, J=8.93 Hz, 2 H) 8.00 (s, 1 H) 7.11 - 7.25 (m, 3 H) 7.02 (s, 1 H) 4.61 (t, J=5.20 Hz, 2 H) 3.96 (br d, J=4.65 Hz, 2 H) 3.48 - 3.60 (m, 6 H) 3.26 (br s, 4 H) 2.96 (s, 5 H) 1.71 - 1.86 (m, 6 H). **LCMS** (ESI+): *m/z* 499.2 (M+H)

Analog 102

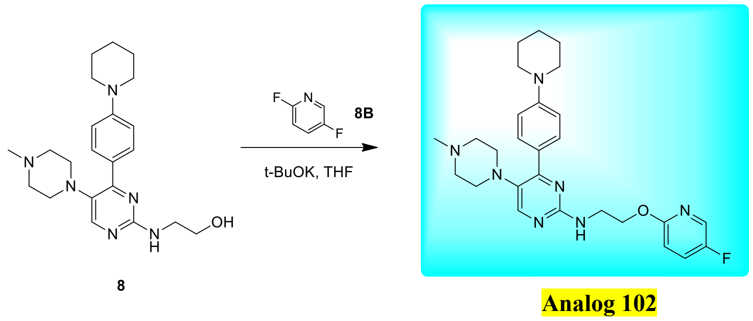

Analog 102

N*-(2-((5-fluoropyridin-2-yl)oxy)ethyl)-5-(4-methylpiperazin-1-yl)-4-(4-(piperidin-1-yl)phenyl)pyrimidin-2-amine (Analog 102)**. To a solution of **Compound 8** (80 mg, 201.75 umol) in THF (2 mL) was added t-BuOK (67.92 mg, 605.25 umol) and the mixture was stirred at 20°C for 10 min, then **Compound 8B** (69.65 mg, 605.25 umol) was added. The mixture was stirred at 70°C for 12 hrs. LCMS and HPLC showed **Compound 8** was consumed completely and 60.7% of desired compound was detected. The reaction mixture was concentrated under reduced pressure to remove THF. The residue was purified by prep-HPLC (TFA condition, column: Phenomenex luna C18 100*40mm*5 um; mobile phase: [water (0.1%TFA) - ACN]; B%: 15%-45%, 8 min) to afford ***102 (27.58 mg, 45.54 umol, 22.57% yield, 100% purity, TFA) as a yellow gum. ^{1}**H NMR** (METHANOL-d_4 400MHz) δ ppm 8.26 (d, J=8.91 Hz, 2 H) 7.95 - 8.03 (m, 2 H) 7.44 (td, J=8.44, 2.95 Hz, 1 H) 7.17 (br d, J=8.53 Hz, 2 H) 6.75 (dd, J=9.10, 3.58 Hz, 1 H) 4.51 (t, J=5.33 Hz, 2 H) 3.87 - 4.00 (m, 2 H) 3.44 - 3.61 (m, 6 H) 3.26 (br s, 4 H) 2.92 - 3.09 (m, 5 H) 1.76 (br s, 6 H). **LCMS** (ESI+): *m/z* 492.2 (M+H).

Analog 103

THE

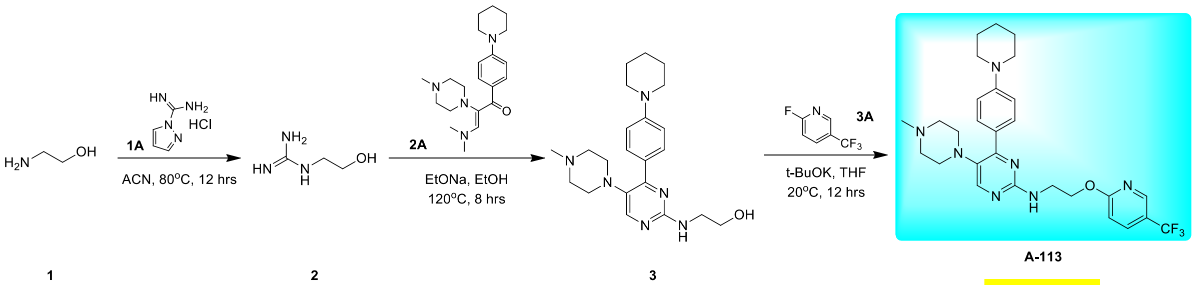

Analog 103

5-(4-methylpiperazin-1-yl)-4-(4-(piperidin-1-yl)phenyl)-*N*-(2-((5-(trifluoromethyl)pyridin-2-yl)oxy)ethyl)pyrimidin-2-amine (Analog 103). To a solution of Compound 1 (5 g, 81.86 mmol, 4.95 mL) in ACN (50 mL) was added Compound 1A (12.00 g, 81.86 mmol, HCl) at 20°C. The mixture was stirred at 80°C for 12 hrs. LCMS showed Compound 1 was consumed completely and 100% of desired

compound was detected. The reaction mixture was concentrated under reduced pressure to remove ACN. The residue was purified by prep-HPLC (basic condition, column: Agela DuraShell C18 250*70mm*10um; mobile phase: [water (0.05% ammonia hydroxide v/v) - ACN]; B%: 0%-0%, 8min) to afford Compound 2 (1 g, 9.70 mmol, 11.85% yield, 100% purity) as a white solid. MS-ESI (m/z) calcd for C3H9N3O [M+H]+: 104.1 Found 104.1. To a solution of Compound 2 (1 g, 2.81 mmol) and Compound 2A (1.16 g, 11.22 mmol) in EtOH (10 mL) was added EtONa (1.15 g, 16.83 mmol) at 20°C, then the mixture was stirred at 120°C for 8 hrs under microwave. LCMS showed Compound 2 was consumed and 28% of desired product was detected. The reaction was concentrated under vacuum. The filtrate was purified by flash silica gel chromatography (ISCO®; 12 g SepaFlash® Silica Flash Column, Eluent of 0~50% (Dichloromethane: Methanol=10:1)/Petroleum ether gradient @ 40 mL/min) (Dichloromethane: Methanol=10:1, P1 Rf=0.23) to afford Compound 3 (325 mg, 573.73 umol, 20.45% yield, 70% purity) as a yellow gum. MS-ESI (m/z) calcd for C22H32N6O [M+H]+: 396.3 Found 397.1 To a solution of Compound 3 (50 mg, 126.09 umol) in THF (2 mL) was added t-BuOK (42.45 mg, 378.28 umol) at 20°C, then the mixture was stirred at 20°C for 10 min, then Compound 3A (41.63 mg, 252.19 umol, 2.89 uL) was added at 20°C, then the mixture was stirred at 20°C for 12 hrs. LCMS showed Compound 3 was consumed and 39% of desired product was detected. The reaction was concentrated under vacuum. The residue was purified by Prep-HPLC (TFA condition) (column: Phenomenex luna C18 100*40mm*5 um; mobile phase: [water (0.1%TFA) - ACN]; B%: 15%-50%, 8 min) to afford A-113 (22.06 mg, 33.51 umol, 26.57% yield, 99.58% purity, TFA) as a yellow gum. ^{1}**H NMR** (DMSO-d_6 400MHz) δ ppm (400 MHz, METHANOL-d4) δ ppm 8.45 (s, 1 H) 8.28 (d, J=8.82 Hz, 2 H) 8.00 (s, 1 H) 7.88 (dd, J=8.82, 2.43 Hz, 1 H) 7.19 (br d, J=8.82 Hz, 2 H) 6.90 (d, J=8.60 Hz, 1 H) 4.64 (t, J=5.29 Hz, 2 H) 3.91 - 4.02 (m, 2 H) 3.46 - 3.59 (m, 6 H) 3.20 - 3.30 (m, 4 H) 2.93 - 3.07 (m, 5 H) 1.78 (br d, J=5.51 Hz, 6 H). LCMS (ESI+): m/z 542.2 (M+H)

Analog 104

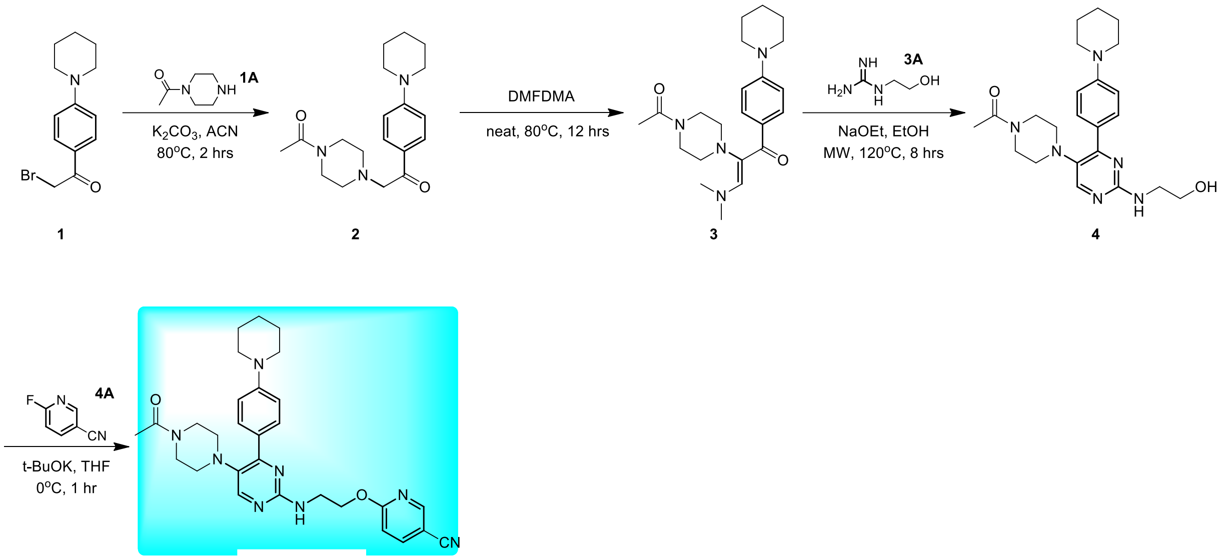

Analog 104

6-(2-((5-(4-acetylpiperazin-1-yl)-4-(4-(piperidin-1-yl)phenyl)pyrimidin-2-yl)amino)ethoxy)nicotinonitrile (Analog 104). To a solution of Compound 1 (1 g, 3.54 mmol) and Compound 1A (908.45 mg, 7.09 mmol) in ACN (5 mL) was added K2CO3 (1.47 g, 10.63 mmol) at 20°C. The mixture was stirred at 80°C for 2 hr. LCMS showed Compound 1 was consumed completely and 71% of desired compound was detected. The reaction mixture was concentrated under reduced pressure to remove ACN. The residue was diluted with H2O 10 mL and extracted with EtOAc (10 mL * 3). The combined organic layers were dried over anhydrous Na2SO4, filtered and concentrated under reduced pressure to give a residue. The residue was purified by flash silica gel chromatography (ISCO®; 12 g SepaFlash® Silica Flash Column, Eluent of 0~20% (Dichloromethane: Methanol)/Petroleum ether gradient @ 80 mL/min) (SiO2, Dichloromethane: Methanol =10:1, Rf (P1) =0.32) to afford Compound 2 (567 mg, 1.72 mmol, 48.57% yield) as a yellow solid. MS-ESI (m/z) calcd for C19H27N3O2 [M+H]+: 330.2 Found 330.3. A solution of Compound 2 (467 mg, 1.42 mmol) in DMFDMA (4.22 g, 24.21 mmol, 5 mL) was stirred at 80°C for 12 hrs. LCMS showed Compound 2 was consumed completely and 13% of desired compound was detected. The reaction mixture was concentrated under reduced pressure to afford Compound 3 (555 mg, crude) as a yellow solid. MS-ESI (m/z) calcd for C22H32N4O2 [M+H]+: 385.3. Found 385.3. To a solution of Compound 3 (347 mg, 902.44 umol) and Compound 3A (186.12 mg, 1.80 mmol) in EtOH (3 mL) was added EtONa (245.64 mg, 3.61 mmol) at 20°C. The mixture was stirred at 120°C for 8 hrs under microwave. LCMS showed Compound 3 was consumed completely and 63% of desired compound was detected. The reaction mixture was concentrated under reduced pressure to remove solvent. The residue was diluted with H2O 3 mL and extracted with EtOAc (3 mL * 3). The combined organic layers were dried over anhydrous Na2SO4, filtered and concentrated under reduced pressure to afford Compound 4 (407 mg, crude) as a yellow oil. MS-ESI (m/z) calcd for C23H32N6O2 [M+H]+: 425.3. Found 425.3. To a solution of Compound 4 (150 mg, 353.33 umol) in THF (3 mL) was added t-BuOK (118.94 mg, 1.06 mmol) at 0°C. The mixture was stirred at 0°C for 10 min, then Compound 4A (86.28 mg, 706.65 umol) was added. The mixture was stirred at 0°C for 1 hr. LCMS showed Compound 4 was consumed completely and 14% of desired compound was detected. The reaction mixture was concentrated under reduced pressure to remove solvent. The residue was purified by prep-HPLC (basic condition, column: Waters Xbridge BEH C18 100*30mm*10um; Mmobile phase: [water (0.05% NH3H2O+10mM NH4HCO3) - ACN]; B%: 40%-65%, 8 min) to afford **104** (12.74 mg, 23.90 umol, 6.76% yield, 98.78% purity) as a yellow

solid. **^{1}H NMR** (DMSO-d_6 400MHz) δ ppm 8.67 (d, J=2.13 Hz, 1 H) 8.04 - 8.20 (m, 4 H) 6.84 - 7.06 (m, 4 H) 4.49 (t, J=5.65 Hz, 2 H) 3.68 (q, J=5.48 Hz, 2 H) 3.48 (br d, J=4.02 Hz, 4 H) 3.26 (br d, J=5.27 Hz, 4 H) 2.69 - 2.82 (m, 4 H) 1.99 (s, 3 H) 1.59 (br s, 6 H). LCMS (ESI+): m/z 527.3 (M+H).

Analog 105

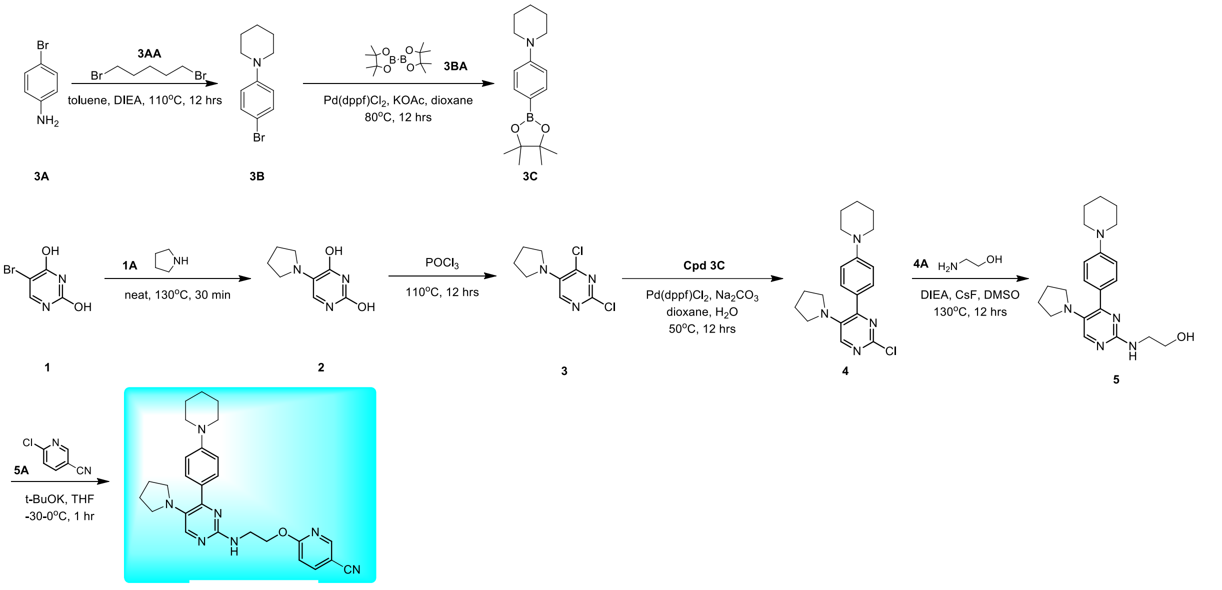

Analog 105

6-(2-((4-(4-(piperidin-1-yl)phenyl)-5-(pyrrolidin-1-yl)pyrimidin-2-yl)amino)ethoxy)nicotinonitrile (Analog 105). To a solution of Compound 3A (3.74 g, 21.74 mmol) in toluene (80 mL) was added DIEA (5.62 g, 43.49 mmol, 7.58 mL) at 20°C, then Compound 3AA (5 g, 21.74 mmol, 2.94 mL) in toluene (80 mL) was added at 20°C. The mixture was stirred at 110°C for 12 hrs. LCMS showed 32% of Compound 3A was remained and 55% of desired compound was detected. The reaction mixture was concentrated under reduced pressure to remove toluene. The residue was diluted with H2O 80 mL and extracted with EtOAc (80 mL * 3). The combined organic layers were dried over anhydrous Na2SO4, filtered and concentrated under reduced pressure to give a residue. The residue was purified by flash silica gel chromatography (ISCO®; 20 g SepaFlash® Silica Flash Column, Eluent of 0~2% Ethyl acetate/Petroleum ether gradient @ 80 mL/min) (SiO2, Petroleum ether : Ethyl acetate=5:1, Rf (P1)=0.81) to afford Compound 3B (4 g, 15.61 mmol, 71.78% yield, 93.7% purity) as a yellow solid. MS-ESI (m/z) calcd for C11H14BrN [M+H]+: 240.0/242.0 Found 240.2/242.2. To a solution of Compound 3B (100 mg, 416.43 umol) and Compound 3BA (126.90 mg, 499.72 umol) in dioxane (2 mL) was added KOAc (122.61 mg, 1.25 mmol) and Pd(dppf)Cl2 (30.47 mg, 41.64 umol) at 20°C. The mixture was stirred at 80°C for 12 hrs. LCMS showed Compound 3B was consumed completely and 33% of desired compound was detected. The reaction mixture was concentrated under reduced pressure to remove solvent. The residue was purified by flash silica gel chromatography (ISCO®; 12 g SepaFlash® Silica Flash Column, Eluent of 0~5% Ethyl acetate/Petroleum ether gradient

@ 80 mL/min) (SiO_2, Petroleum ether: Ethyl acetate=10:1, Rf(P1)=0.53) to afford Compound 3C (61 mg, 212.39 umol, 51.00% yield) as a yellow solid. MS-ESI (m/z) calcd for $C_{17}H_{26}BNO_2$ $[M+H]^+$: 288.2 Found 288.3 1H NMR (400 MHz, METHANOL-d4) δ ppm 7.59 (d, J=8.76 Hz, 2 H) 6.90 (d, J=8.63 Hz, 2 H) 3.17 - 3.28 (m, 4 H) 1.57 - 1.74 (m, 6 H) 1.32 (s, 12 H). To a solution of Compound 1 (3 g, 15.71 mmol) and Compound 1A (4.47 g, 62.83 mmol, 5.24 mL) was was stirred at 130°C for 30 min. LCMS showed Compound 1 was consumed completely and 98%of desired compound was detected. The reaction mixture was cooled to 20°C. The product was collected by filtration, washed with methanol and dried under reduced pressure to afford Compound 2 (2.49 g, crude) as a white solid. MS-ESI (m/z) calcd for $C_8H_{11}N_3O_2$ $[M+H]^+$: 182.1. Found 182.1 1H NMR (400 MHz, DMSO-d6) δ ppm 10.98 (br s, 1 H) 10.30 (br s, 1 H) 6.48 (s, 1 H) 2.78 - 3.18 (m, 4 H) 1.60 - 2.00 (m, 4 H). A solution of $POCl_3$ (24.75 g, 161.41 mmol, 15 mL) was stirred at 0°C for 10 min, then Compound 2 (2.49 g, 13.74 mmol) was added, The mixture was stirred at 110°C for 12 hrs. LCMS showed 8% of Compound 2 was remained and 69% of desired compound was detected. The reaction mixture was concentrated under reduced pressure to remove $POCl_3$. The residue was diluted with saturated $NaHCO_3$ 50 mL and extracted with EtOAc (50 mL * 3) and (DCM:MeOH=10:1) (50 mL * 1). The combined organic layers were washed with brine 50 mL, dried over anhydrous Na_2SO_4, filtered and concentrated under reduced pressure to give a residue. The residue was purified by flash silica gel chromatography (ISCO®; 12 g SepaFlash ® Silica Flash Column, Eluent of 0~5% Ethyl acetate/Petroleum ether gradient @ 80 mL/min) (SiO_2, Petroleum ether: Ethyl acetate=3:1, Rf(P1)=0.47) to afford Compound 3 (1.51 g, 6.92 mmol, 50.34% yield, 99.71% purity) as a white solid. MS-ESI (m/z) calcd for $C_8H_9Cl_2N_3$ $[M+H]^+$: 218.0/220.0. Found 218.1/220.1. To a solution of Compound 3 (400 mg, 1.83 mmol) and Compound 3C (632.14 mg, 2.20 mmol) in dioxane (4 mL) and H_2O (0.6 mL) was added Na_2CO_3 (583.21 mg, 5.50 mmol) and $Pd(dppf)Cl_2$ (134.21 mg, 183.42 umol) at 20°C. The mixture was stirred at 50°C for 12 hrs under N_2. LCMS showed Compound 3 was consumed completely and 47% of desired compound was detected. The reaction mixture was concentrated under reduced pressure to remove solvent. The residue was purified by flash silica gel chromatography (ISCO®; 12 g SepaFlash® Silica Flash Column, Eluent of 0~5% Ethyl acetate/Petroleum ether gradient @ 50mL/min) (SiO_2, Petroleum ether: Ethyl acetate=5:1, Rf (P1)=0.37) to afford Compound 4 (590 mg, 1.54 mmol, 84.06% yield, 89.6% purity) as a yellow solid. MS-ESI (m/z) calcd for $C_{19}H_{23}ClN_4$ $[M+H]^+$: 343.2/345.2. Found 343.3/345.3. To a solution of Compound 4 (590 mg, 1.72 mmol) and Compound 4A (262.78 mg, 4.30 mmol, 260.18 uL) in DMSO (1 mL) was added DIEA (667.19 mg, 5.16 mmol, 899.17 uL) and CsF (392.08 mg, 2.58 mmol, 95.17 uL) at 20°C. The mixture was stirred at 130°C for 12 hrs. LCMS showed 20% of Compound 4 was remained and 39% of desired compound was detected. The reaction mixture was diluted with H_2O 2 mL and extracted with EtOAc (2 mL * 3) and (DCM:MeOH=10:1) (2 mL * 1). The combined organic layers were washed with brine (5 mL * 1), dried over anhydrous Na_2SO_4, filtered and concentrated under reduced pressure to give a residue to afford Compound 5 (518 mg, crude) as a yellow oil. MS-ESI (m/z) calcd for $C_{21}H_{29}N_5O$ $[M+H]^+$: 368.2. Found 368.4. To a solution of Compound 5 (50 mg, 136.06 umol) in THF (1 mL) was added t-BuOK (22.90 mg, 204.09 umol) at 0°C. The mixture was stirred 0°C for 10 min. Then Compound 5A (20.74 mg, 149.67 umol) was added at -30°C. The mixture was stirred at -30°C for 1 hr. LCMS and showed 8.9% of Compound 5 was remained and 10% of desired compound was detected. The reaction mixture was concentrated under reduced pressure to remove solvent. The residue was purified by prep-HPLC (basic condition, column: Waters Xbridge BEH C18 100*30mm*10um; mobile phase: [water (0.05% NH_3H_2O+10mM NH_4HCO_3) - ACN]; B%: 45%-75%, 8 min) (basic condition, column: Waters Xbridge BEH C18 100*30mm*10um;

mobile phase: [water (10mM NH4HCO3) - ACN]; B%: 45%-85%, 8 min) to afford **105** (4.12 mg, 8.77 umol, 6.45% yield, 100% purity) as a yellow solid. 1H NMR (400 MHz, DMSO-d_6) δ ppm 8.65 - 8.69 (m, 1 H) 8.11 (dd, J=8.71, 2.32 Hz, 1 H) 8.04 (s, 1 H) 7.82 (d, J=8.82 Hz, 2 H) 6.97 (d, J=8.82 Hz, 1 H) 6.92 (d, J=9.04 Hz, 2 H) 6.75 (t, J=5.95 Hz, 1 H) 4.48 (t, J=5.84 Hz, 2 H) 3.65 (q, J=5.59 Hz, 2 H) 3.20 - 3.28 (m, 4 H) 2.82 (br s, 4 H) 1.76 (br s, 4 H) 1.58 (br d, J=4.63 Hz, 6 H). LCMS (ESI+): m/z 470.3 (M+H)

Analog 106

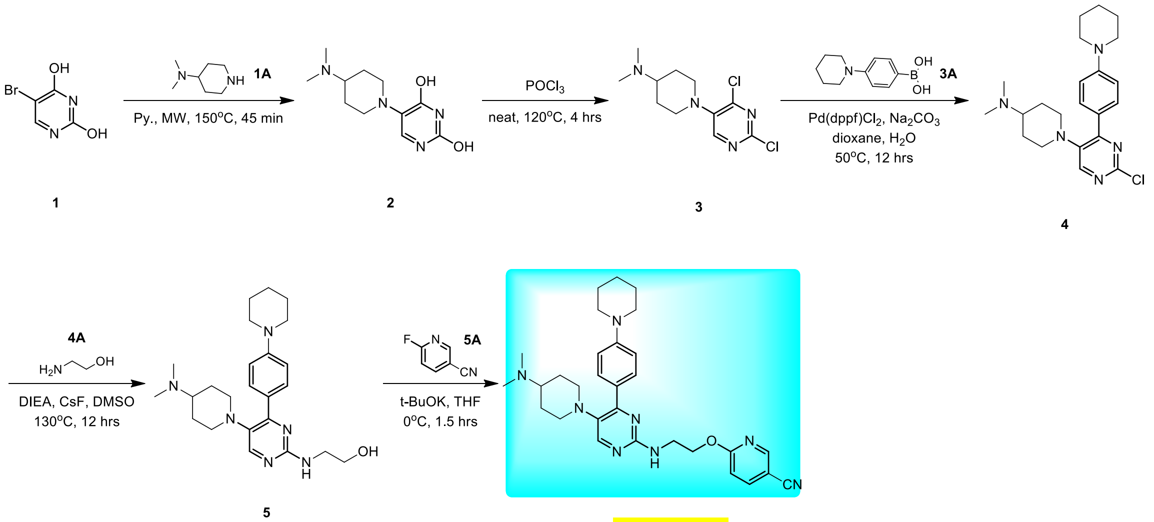

Analog 106

6-(2-((5-(4-(dimethylamino)piperidin-1-yl)-4-(4-(piperidin-1-yl)phenyl)pyrimidin-2-yl)amino)ethoxy)nicotinonitrile (Analog 106). To a solution of Compound 1 (1 g, 5.24 mmol) in Py. (10 mL) was added Compound 1A (1.01 g, 7.85 mmol) at 20°C, then the mixture was stirred at 150°C for 45 min. LCMS showed Compound 1 was consumed and a main peak with desired MS was detected. The reaction was concentrated under vacuum. EtOAc (20 mL) was added and the mixture was stirred at 20°C for 30 min, then the mixture was filtered and the filter-cake was dried under vacuum to afford Compound 2 (1.59 g, crude) as a pale yellow solid and it was used directly. MS-ESI (m/z) calcd for

C11H18N4O2 [M+H]+: 239.1 Found 239.1. Compound 2 (1.39 g, 5.83 mmol) was added into POCl3 (16.50 g, 107.61 mmol, 10 mL) slowly at 20°C, then the mixture was stirred at 120°C for 4 hrs. LCMS showed Compound 2 was consumed and 53% of desired product was detected. The reaction was concentrated under vacuum. The residue was diluted with EtOAc (20 mL), then the mixture was poured into saturated Na2CO3 solution (50 mL) slowly, then the mixture was extracted with DCM : MeOH (10:1) (20 mL * 3), the organic layer was dried over anhydrous Na2SO4, filtered and the filtrate was concentrated under vacuum. The residue was purified by flash silica gel chromatography (ISCO®; 4 g SepaFlash® Silica Flash Column, Eluent of 0~50~100% (DCM:MeOH=10:1)/Petroleum ether gradient @ 18 mL/min) (Dichloromethane : Methanol=10:1)(P1 RF=0.05) to afford Compound 3 (402 mg, 1.46 mmol, 25.04% yield) as a yellow solid. MS-ESI (m/z) calcd for C11H16Cl2N4 [M+H]+: 275.1/277.1. Found 275.2/277.2. To a solution of Compound 3 (392 mg, 1.42 mmol) and Compound 3A (450.05 mg, 1.57 mmol) in dioxane (4 mL) and H2O (0.6 mL) were added Pd(dppf)Cl2 (104.23 mg, 142.45 umol) and Na2CO3 (452.96 mg, 4.27 mmol) at 20°C, then the mixture was stirred at 50°C for 12 hrs under N2. LCMS showed 7.9% of Compound 3 was remained and 31% of desired product was detected. The reaction was concentrated under vacuum. The residue was purified by flash silica gel chromatography (ISCO®; 4 g SepaFlash® Silica Flash Column, Eluent of 0~50~100% DCM:MeOH (10:1)/Petroleum ether gradient @ 18 mL/min) (Dichloromethane : Methanol=10:1) (P1 Rf=0.32) to afford Compound 4 (280 mg, 448.05 umol, 31.45% yield, 64% purity) as a yellow liquid. MS-ESI (m/z) calcd for C22H30ClN5 [M+H]+: 400.2/403.2. Found 400.4/402.4. To a solution of Compound 4 (130 mg, 325.03 umol) and Compound 4A (49.63 mg, 812.58 umol, 49.14 uL) in DMSO (2 mL) were added DIEA (210.04 mg, 1.63 mmol, 283.07 uL) and CsF (74.06 mg, 487.55 umol, 17.98 uL, 1.5 eq) at 20°C, then the mixture was stirred at 130°C for 12 hrs. LCMS showed Compound 4 was consumed and 29% of desired product was detected. The reaction was cooled to room temperature and the mixture was diluted with H2O (5 mL), then the mixture was extracted with DCM:MeOH (10:1) (5 mL * 3), the organic layers were washed the brine (5 mL), then the organic layer was dried over anhydrous Na2SO4, filtered and the filtrate was concentrated under vacuum to afford Compound 5 (166 mg, crude) as a brown liquid and it was used directly. MS-ESI (m/z) calcd for C24H36N6O [M+H]+: 425.3. Found 425.2. To a solution of Compound 5 (146 mg, 343.87 umol) in THF (3 mL) was added t-BuOK (115.76 mg, 1.03 mmol) at 0°C, then the mixture was stirred at 0°C for 5 min, then Compound 5A (83.97 mg, 687.74 umol) was added at 0°C, then the mixture was stirred at 0°C for 1.5 hrs. LCMS showed Compound 5 was consumed and 20% of desired product was detected. The reaction was concentrated under vacuum. The residue was purified by Prep-HPLC (TFA condition) (column: Phenomenex luna C18 100*40mm*5 um; mobile phase: [water (0.1% TFA) - ACN]; B%: 5%-37%, 8 min), the residue was not pure, then the residue was purified by Prep-HPLC (basic condition) (column: Waters Xbridge Prep OBD C18 150*40mm*10um; mobile phase: [water (0.05% NH3H2O+10mM NH4HCO3) - ACN]; B%: 25%-55%, 8 min) to afford **106** (26.49 mg, 48.26 umol, 14.04% yield, 95.96% purity) as a yellow gum. ^{1}**H NMR** (METHANOL-d_4 400MHz) δ ppm 8.49 (d, J=2.00 Hz, 1 H) 8.07 (br d, J=8.88 Hz, 2 H) 7.99 (s, 1 H) 7.88 (dd, J=8.69, 2.19 Hz, 1 H) 6.96 (br d, J=8.88 Hz, 2 H) 6.86 (d, J=8.75 Hz, 1 H) 4.57 (t, J=5.38 Hz, 2 H) 3.80 (br t, J=5.44 Hz, 2 H) 3.28 (br d, J=5.63 Hz, 4 H) 3.08 (br d, J=11.13 Hz, 2 H) 2.58 (br t, J=11.38 Hz, 2 H) 2.31 (s, 6 H) 2.20 (br t, J=11.38 Hz, 1 H) 1.88 (br d, J=10.76 Hz, 2 H) 1.71 (br d, J=4.25 Hz, 4 H) 1.64 (br d, J=4.63 Hz, 2 H) 1.49 - 1.60 (m, 2 H). LCMS (ESI+): m/z 527.4 (M+H)

Analog 107

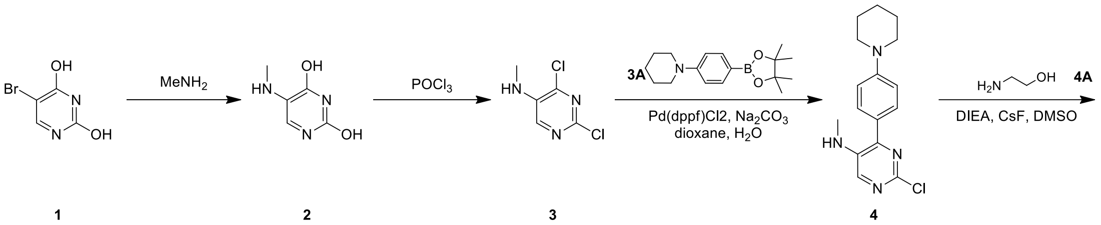

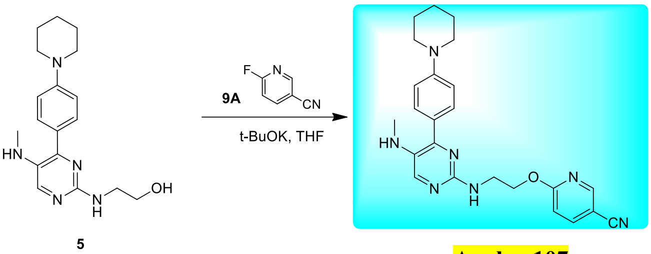

Analog 107

6-(2-((5-(methylamino)-4-(4-(piperidin-1-yl)phenyl)pyrimidin-2-yl)amino)ethoxy)nicotinonitrile (Analog 107). A solution of Compound 1 (2 g, 10.47 mmol) in MeNH2 (2 M, 31.42 mL) was stirred at 100°C in a sealed tube for 12 hrs. LCMS showed 40% of Compound 1 was remained and less desired compound was detected. The mixture was stirred at 100°C in a sealed tube for 12 hrs. LCMS showed Compound 1 was consumed completely and desired mass was detected. The reaction mixture was cooled to 20°C. The product was collected by filtration, the filter-cake was washed with methanol and dried under reduced pressure to afford Compound 2 (1.2 g, 8.50 mmol, 81.20% yield) as a yellow solid. MS-ESI (m/z) calcd for C5H7N3O2 [M+H]+: 142.1 Found 142.1. POCl3 (21.73 g, 141.72 mmol, 13.17 mL) was stirred at 0°C for 10 min, Compound 2 (0.8 g, 5.67 mmol) was added at 0°C. The mixture was stirred at 110°C for 12 hrs. TLC indicated the reaction completed and one major new spot polarity was detected. The reaction mixture was concentrated under reduced pressure to remove POCl3. The residue was diluted with saturated NaHCO3 20 mL and extracted with EtOAc (20 mL * 3) and (DCM:MeOH=10:1)(50 mL * 1). The combined organic layers were washed with brine (50 mL * 1), dried over anhydrous Na2SO4, filtered and concentrated under reduced pressure to give a residue. The residue was purified by flash silica gel chromatography (ISCO®; 4 g SepaFlash® Silica Flash Column, Eluent of 0~20% Ethyl acetate/Petroleum ether gradient @ 80 mL/min) to afford Compound 3 (0.5 g, crude) as a yellow solid. To a solution of Compound 3 (250 mg, 1.40 mmol) and Compound 3A (484.00 mg, 1.69 mmol) in dioxane (2.5 mL) and H2O (0.8 mL) were added Na2CO3 (446.54 mg, 4.21 mmol) and Pd(dppf)Cl2 (102.76 mg, 140.43 umol) at 20°C. The mixture was stirred at 50°C for 12 hrs under N2. LCMS showed Compound 3 was consumed completely and desired mass was detected. The reaction mixture was added to H2O (5 mL) and extracted with EtOAc (5 mL * 3). The combined organic layers were dried over Na2SO4, filtered and concentrated under reduced pressure to give a residue. The residue was purified by flash silica gel chromatography (ISCO®; 4g SepaFlash® Silica Flash Column, Eluent of 0~20% Ethyl acetate/Petroleum ether gradient @50 mL/min) to afford Compound 4 (250 mg, 825.62 umol, 58.79% yield) as a yellow solid. MS-ESI (m/z) calcd for C16H19ClN4 [M+H]+: 303.1/305.1 Found 303.3/305.3. To a solution of Compound 4 (0.1 g, 330.25 umol) and Compound 4A (50.43 mg, 825.62 umol, 49.93 uL), CsF (75.25 mg, 495.37 umol, 18.26 uL) in DMSO (1 mL) was added DIEA (128.04 mg, 990.75 umol, 172.57 uL) at 20°C. The mixture was stirred at 130°C for 12 hrs. LCMS showed 60% of Compound 4 was remained. Several new peaks were shown on LCMS and less desired compound was detected. The mixture was stirred at 130°C for 12 hrs. LCMS showed 30% of Compound 4 was remained. Several new peaks were shown on LCMS and less desired compound was

detected. The reaction mixture was added to H2O (5 mL) and extracted with EtOAc (5 mL * 3). The combined organic layers were dried over Na2SO4, filtered and concentrated under reduced pressure to afford Compound 5 (130 mg, crude) as yellow oil. MS-ESI (m/z) calcd for C18H25N5O [M+H]+: 328.2 Found 328.3. To a solution of Compound 5 (100 mg, 305.42 umol) in THF (3 mL) was added t-BuOK (102.81 mg, 916.25 umol) at -30°C. The mixture was stirred at -30°C for 10 min. Then Compound 9A (111.87 mg, 916.25 umol) was added. The mixture was stirred at -30°C for 1 hr. LCMS showed Compound 5 was consumed completely and desired mass was detected. The reaction mixture was added to H2O (5 mL) and extracted with EtOAc (5 mL * 3). The combined organic layers were dried over Na2SO4, filtered and concentrated under reduced pressure to give a residue. The residue was purified by prep-HPLC (column: Phenomenex luna C18 100*40mm*5 um; mobile phase: [water (0.1%TFA) - ACN]; B%: 15%-45%, 8 min) to afford **107** (7.2 mg, 15.59 umol, 5.10% yield, 93% purity) as a yellow oil. ^{1}**H NMR** (METHANOL-*d*$_4$ 400MHz) δ ppm. 8.5 (d, J=2.26 Hz, 1 H) 7.9 (d, J=8.91 Hz, 3 H) 7.4 (s, 1 H) 7.1 (br d, J=8.91 Hz, 2 H) 6.8 (d, J=8.66 Hz, 1 H) 4.6 (t, J=5.21 Hz, 2 H) 3.9 (t, J=5.21 Hz, 2 H) 3.4 - 3.6 (m, 4 H) 2.7 (s, 3 H) 1.7 (br s, 6 H). LCMS (ESI+): m/z 430.2 (M+H).

Analog 108

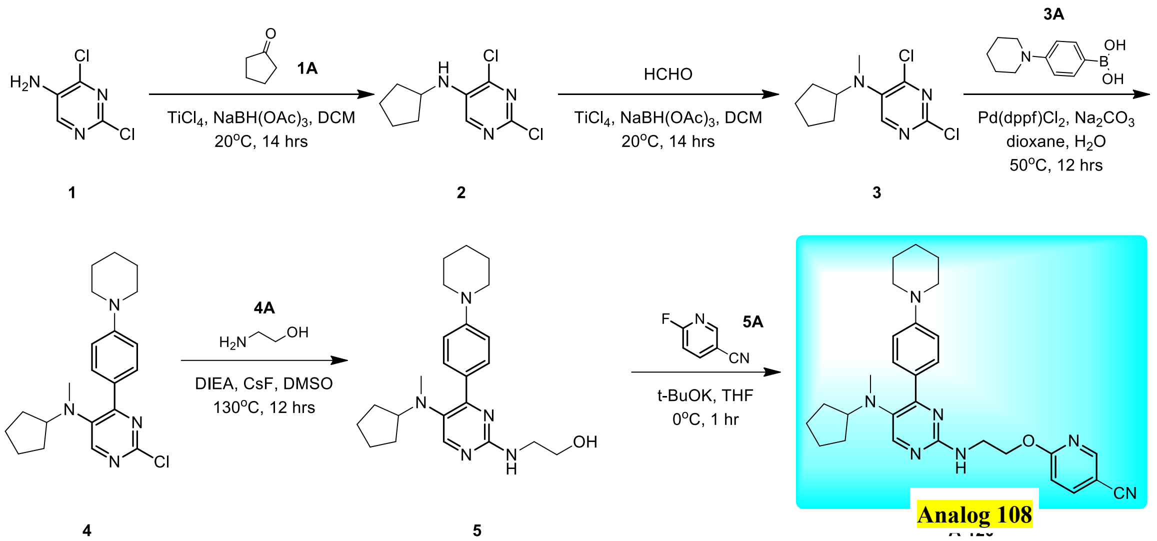

6-(2-((5-(cyclopentyl(methyl)amino)-4-(4-(piperidin-1-yl)phenyl)pyrimidin-2-yl)amino)ethoxy)nicotinonitrile (Analog 108). To a solution of Compound 1 (500.00 mg, 3.05 mmol) and Compound 1A (769.39 mg, 9.15 mmol, 809.88 uL) in DCM (6 mL) was added TiCl4 (1 M, 3.35 mL) at 0°C, then the mixture was stirred at 20°C for 2 hrs under N2, then NaBH(OAc)3 (1.94 g, 9.15 mmol) was added at 20°C, then the mixture was stirred at 20°C for 12 hrs. LCMS showed Compound 1 was consumed and 36% of desired product was detected. The reaction was concentrated under vacuum. The residue was purified by flash silica gel chromatography (ISCO®; 12 g SepaFlash® Silica Flash

Column, Eluent of 0~0% Ethyl acetate/Petroleum ether gradient @ 36 mL/min) to afford Compound 2 (692 mg, 2.98 mmol, 97.78% yield) as a yellow solid. MS-ESI (m/z) calcd for C9H11Cl2N3 [M+H]+: 232.0/234.0 Found 232.2/234.2. To a solution of Compound 2 (356 mg, 1.53 mmol) and HCHO (373.40 mg, 4.60 mmol, 342.57 uL, 37% purity) in DCM (6 mL) was added TiCl4 (1 M, 1.69 mL) at 0°C, then the mixture was stirred at 20°C for 2 hrs under N2, then NaBH(OAc)3 (975.20 mg, 4.60 mmol) was added at 20°C, then the mixture was stirred at 20°C for 12 hrs. LCMS showed 9.9% of Compound 2 was remained and 62% of desired product was detected. The reaction was concentrated under vacuum. The residue was purified by flash silica gel chromatography (ISCO®; 4 g SepaFlash® Silica Flash Column, Eluent of 0~10% Ethyl acetate/Petroleum ether gradient @ 18 mL/min) (Petroleum ether : Ethyl acetate=5:1) (P1 RF=0.70) to afford Compound 3 (125 mg, 507.85 umol, 33.11% yield) as a yellow oil. MS-ESI (m/z) calcd for C10H13Cl2N3 [M+H]+: 246.1/248.1. Found 246.2/248.2. To a solution of Compound 3 (250 mg, 1.02 mmol) and Compound 3A (208.28 mg, 1.02 mmol) in dioxane (2 mL) and H2O (0.3 mL) were added Pd(dppf)Cl2 (74.32 mg, 101.57 umol) and Na2CO3 (322.96 mg, 3.05 mmol) at 20°C, then the mixture was stirred at 50°C for 12 hrs under N2. LCMS showed trace of Compound 3 was remained and 67% of desired product was detected. The mixture was concentrated under vacuum. The residue was purified by flash silica gel chromatography (ISCO®; 4 g SepaFlash® Silica Flash Column, Eluent of 0~10% Ethyl acetate/Petroleum ether gradient @ 18 mL/min) (Petroleum ether : Ethyl acetate=5:1) (P1RF=0.56) to afford Compound 4 (181 mg, 453.82 umol, 44.68% yield, 93% purity) as a yellow liquid. MS-ESI (m/z) calcd for C21H27ClN4 [M+H]+: 371.2/373.2. Found 371.1/373.1. To a solution of Compound 4 (56 mg, 150.98 umol) and Compound 4A (23.06 mg, 377.44 umol, 22.83 uL) in DMSO (1 mL) were added DIEA (97.56 mg, 754.88 umol, 131.49 uL) and CsF (34.40 mg, 226.47 umol, 8.35 uL) at 20°C, then the mixture was stirred at 130°C for 12 hrs. LCMS showed 5% of Compound 4 was remained and 44% of desired product was detected. The reaction was diluted with H2O (3 mL), the mixture was extracted with EtOAc (3 mL * 3), the organic layer were combined and dried over anhydrous Na2SO4, filtered and the filtrate was concentrated under vacuum. The residue was purified by Prep-TLC (Petroleum ether : Ethyl acetate=0:1) (P1 RF=0.57) to afford Compound 5 (42.8 mg, 108.21 umol, 71.67% yield) as a yellow liquid. MS-ESI (m/z) calcd for C23H33N5O [M+H]+: 396.3. Found 396.4. To a solution of Compound 5 (32.8 mg, 82.92 umol) in THF (3 mL) was added t-BuOK (27.92 mg, 248.77 umol) at 0°C, then the mixture was stirred at 0°C for 20 min, then Compound 5A (20.25 mg, 165.85 umol) was added at 0°C, then the mixture was stirred at 0°C for 40 min. LCMS showed trace of Compound 5 was remained and 61% of desired product was detected. The reaction was concentrated under vacuum. The residue was purified by Prep-HPLC (TFA condition) (column: Phenomenex luna C18 100*40mm*5 um; mobile phase: [water (TFA) - ACN]; B%: 15%-60%, 8 min) to afford **108** (13.7 mg, 21.78 umol, 26.27% yield, 97.25% purity, TFA) as a yellow solid. **^{1}H NMR** (METHANOL-d_4 400MHz) δ ppm 8.52 (d, J=2.00 Hz, 1 H) 8.34 (br d, J=9.13 Hz, 2 H) 8.01 (s, 1 H) 7.81 - 7.89 (m, 1 H) 7.12 (br d, J=8.88 Hz, 2 H) 6.83 (br d, J=8.63 Hz, 1 H) 4.68 (br t, J=5.19 Hz, 2 H) 4.00 (br t, J=4.94 Hz, 2 H) 3.51 - 3.59 (m, 4 H) 3.42 - 3.48 (m, 1 H) 2.58 (s, 3 H) 1.73 - 1.85 (m, 8 H) 1.62 - 1.71 (m, 2 H) 1.48 - 1.61 (m, 4 H). LCMS (ESI+): m/z 498.2 (M+H).

Analog 109

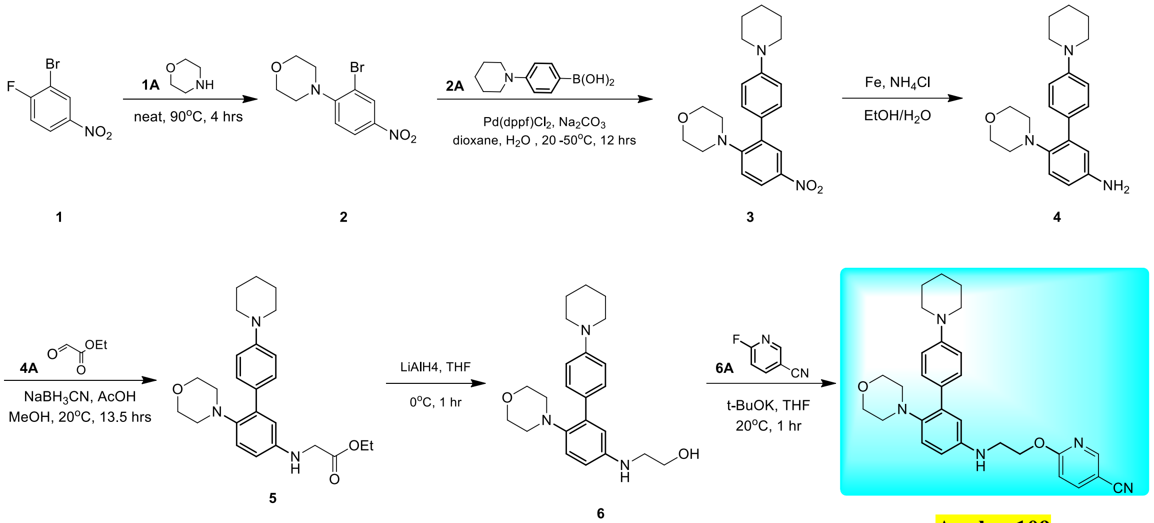

Analog 109

6-(2-((6-morpholino-4'-(piperidin-1-yl)-[1,1'-biphenyl]-3-yl)amino)ethoxy)nicotinonitrile (Analog 109). A mixture of Compound 1 (5 g, 22.73 mmol) and Compound 1A (19.80 g, 227.28 mmol, 20.00 mL) was stirred at 90°C for 4 hrs. LCMS showed Compound 1 was consumed completely and 64% of desired compound was detected. The reaction was cooled to 20°C, then 40 mL H2O was added into the reaction, then solid was appeared and the mixture was filtered and the filter-cake was dried under vacuum to afford Compound 2 (5.92 g, crude) as a yellow solid. MS-ESI (m/z) calcd for C10H11BrN2O3 [M+H]+: 287.0/ 289.0 Found 287.1/289.1. To a solution of Compound 2 (400 mg, 1.39 mmol) and Compound 2A (600.20 mg, 2.09 mmol) in dioxane (6 mL) and H2O (0.9 mL) was added Pd(dppf)Cl2 (101.94 mg, 139.32 umol) and Na2CO3 (442.99 mg, 4.18 mmol) at 20°C. The mixture was stirred at 50°C for 12 hrs under N2. LCMS showed Compound 2 was consumed completely and 35% of desired compound was detected. The reaction mixture was concentrated under reduced pressure to remove solvent. The residue was purified by flash silica gel chromatography (ISCO®; 12 g SepaFlash® Silica Flash Column, Eluent of 0~5% Ethyl acetate/Petroleum ether gradient @ 80 mL/min) (SiO2, Petroleum ether : Ethyl acetate=5:1, P1Rf=0.29). The crude product was triturated with EtOAc (5 mL) and the filtrate-cake was dried under vacuum to afford Compound 3 (220 mg, 598.74 umol, 42.98% yield) as a yellow solid. MS-ESI (m/z) calcd for C21H25N3O3 [M+H]+: 368.2 Found 368.3. To a solution of Compound 3 (350 mg, 952.54 umol) in EtOH (3 mL) and H2O (0.6 mL) was added NH4Cl (254.76 mg, 4.76 mmol) and Fe (265.97 mg, 4.76 mmol) at 20°C. The mixture was stirred at 70°C for 1 hr. LCMS showed Compound 3 was consumed completely and 43.5% of desired compound was detected. The mixture was filtered. The residue was diluted with H2O 5 mL and extracted with EtOAc (5 mL * 3). The combined organic layers were dried over anhydrous Na2SO4, filtered and concentrated under reduced pressure to give a residue to afford Compound 4 (147 mg, crude) as a yellow solid. MS-ESI (m/z) calcd for C21H27N3O [M+H]+: 338.2. Found 338.3. A solution of Compound 4 (147 mg, 435.61 umol) and Compound 4A (133.41 mg, 653.42 umol, 50% purity) in MeOH (0.3 mL) and AcOH (2 mL) was stirred at 20°C for 1.5 hrs, then NaBH3CN (68.44 mg, 1.09 mmol) was added at 20°C . The mixture was stirred at 20°C for 12 hrs. LCMS showed 2% of Compound 4 was remained and 56% of desired compound was detected. The mixture was filtered. The residue was diluted with H2O 5 mL and extracted with EtOAc (5 mL * 3). The combined organic layers were dried over anhydrous Na2SO4, filtered and concentrated under reduced pressure to give a residue. The residue was purified by prep-TLC (SiO2, Petroleum ether : Ethyl acetate=1:1, P1Rf=0.52) to afford Compound 5 (150 mg, 85% purity, 35.39% yield) as a yellow oil. MS-ESI (m/z) calcd for C25H33N3O3 [M+H]+: 424.3. Found 424.4.

A solution of Compound 5 (150 mg, 354.15 umol) in THF (2 mL) was added LiAlH4 (16.13 mg, 424.98 umol) at 0°C. The mixture was stirred at 0°C for 1 hr. LCMS showed Compound 5 was consumed completely and 68% of desired compound was detected. The reaction mixture was quenched by saturated MgSO4 solution (1mL) slowly at 0°C, then Na2SO4 was added and mixture was stirred at 20°C for 10 min, then the mixture was filtered and the filtrate was concentrated under vacuum. The residue was purified by prep-TLC (SiO2, Ethyl acetate : Petroleum ether=2:1, P1Rf=0.37) to afford Compound 6 (63 mg, 165.13 umol, 46.63% yield) as a yellow oil. MS-ESI (m/z) calcd for C23H31N3O2 [M+H]+: 382.2. Found 382.3. To a solution of Compound 6 (48 mg, 125.82 umol) in THF (1 mL) was added t-BuOK (42.35 mg, 377.45 umol) at 0°C. The mixture was stirred at 0°C for 10 min, then Compound 6A (46.09 mg, 377.45 umol) was added, the mixture was stirred at 0°C for 1 hr. LCMS and HPLC showed 6 % of Compound 6 was remained and 37 % of desired compound was detected. The reaction mixture was concentrated under reduced pressure to remove solvent. The residue was purified by prep-HPLC (basic condition, column: Waters Xbridge BEH C18 100*30mm*10um; mobile phase: [water (0.05% NH3H2O+10mM NH4HCO3) - ACN]; B%: 60%-95%, 8 min) to afford **109** (10.70 mg, 20.80 umol, 16.53% yield, 94.01% purity) as a yellow solid. **^{1}H NMR** (DMSO-d_6 400MHz) δ ppm 8.66 (d, J=2.00 Hz, 1 H) 8.15 (dd, J=8.76, 2.25 Hz, 1 H) 7.46 (d, J=8.63 Hz, 2 H) 7.00 (d, J=8.63 Hz, 1 H) 6.93 (br d, J=8.63 Hz, 2 H) 6.82 - 6.88 (m, 1 H) 6.52 (br d, J=2.50 Hz, 2 H) 5.54 (br t, J=5.69 Hz, 1 H) 4.47 (br t, J=5.69 Hz, 2 H) 3.49 (br s, 4 H) 3.37 - 3.45 (m, 2 H) 3.12 - 3.20 (m, 4 H) 2.61 (br s, 4 H) 1.62 (br d, J=3.88 Hz, 4 H) 1.54 (br d, J=4.63 Hz, 2 H) LCMS (ESI+): m/z 484.3 (M+H)

Analog 110

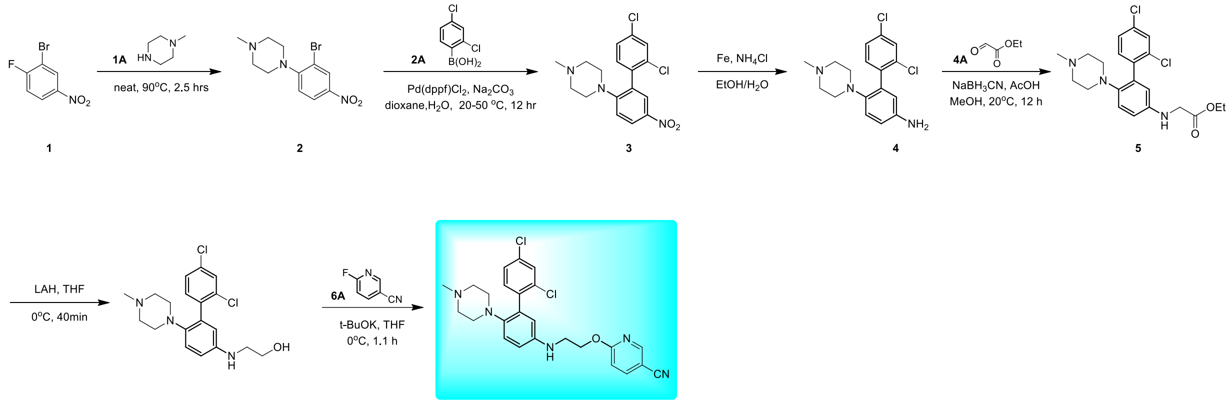

Analog 110

6-(2-((2',4'-dichloro-6-(4-methylpiperazin-1-yl)-[1,1'-biphenyl]-3-yl)amino)ethoxy)nicotinonitrile (Analog 110). A mixture of Compound 1 (5 g, 22.73 mmol) and Compound 1A (22.77 g, 227.30 mmol, 25.21 mL) was stirred at 90°C for 2.5 hrs. TLC (Petroleum ether : Ethyl acetate=5:1) (P1Rf=0.00) showed Compound 1 was consumed and a new spot was formed. The reaction was cooled to room temperature, then 40 mL H2O was added into the reaction, then solid was appeared and the mixture was filtered and the filter-cake was dried under vacuum to afford Compound 2 (6.7 g, crude) as a yellow solid and it was used to next step directly. 1H NMR METHANOL-d4 400MHz δ ppm 8.37 (d, J=2.57 Hz, 1 H) 8.15 (dd, J=8.92, 2.69 Hz, 1 H) 7.22 (d, J=8.92 Hz, 1 H) 3.22 (br s, 4 H) 2.66 (br s, 4 H) 2.37 (s, 3 H) To a solution of Compound 2 (1 g, 3.33 mmol) and Compound 2A (762.89 mg, 4.00 mmol) in dioxane (10 mL) and H2O (1.5 mL) were added Pd(dppf)Cl2 (243.78 mg, 333.17 umol) and

Na_2CO_3 (1.06 g, 9.99 mmol) at 20°C, then the mixture was stirred at 50°C for 12 hrs under N_2. LCMS showed 9% of Compound 2 was consumed and 33% of desired product was detected. The reaction was concentrated under vacuum. The residue was purified by flash silica gel chromatography (ISCO®; 4 g SepaFlash® Silica Flash Column, Eluent of 0~30% DCM : MeOH (10:1)/Petroleum ether gradient @ 18 mL/min) (Dichloromethane : Methanol=10:1) (P1Rf=0.54) to afford Compound 3 (1.18 g, 2.67 mmol, 80.27% yield, 83% purity) as a yellow liquid. MS-ESI (m/z) calcd for $C_{17}H_{17}Cl_2N_3O_2$ [M+H]+: 365.1/367.1 Found 366.0/368.0. To a solution of Compound 3 (500 mg, 1.37 mmol) in EtOH (8 mL) and H_2O (2 mL) were added Fe (381.24 mg, 6.83 mmol) and NH_4Cl (365.13 mg, 6.83 mmol) at 20°C, then the mixture was stirred at 70°C for 3 hrs. LCMS showed Compound 3 was consumed and 80% of desired product was detected. The reaction was concentrated under vacuum. The residue was purified by flash silica gel chromatography (ISCO®; 4 g SepaFlash® Silica Flash Column, Eluent of 0~20~70~100% (DCM : MeOH=10:1)/Petroleum ether gradient @ 18 mL/min) (Dichloromethane : Methanol=10:1) (P1Rf=0.33) to afford Compound 4 (348 mg, 1.03 mmol, 75.81% yield) as a yellow liquid. MS-ESI (m/z) calcd for $C_{17}H_{19}Cl_2N_3$ [M+H]+: 335.1/337.1. Found 336.2/338.2. A solution of Compound 4 (148 mg, 440.14 umol) and Compound 4A (134.80 mg, 660.21 umol, 50% purity) in AcOH (2 mL) and MeOH (0.3 mL) was stirred at 20°C for 1.5 hrs, then $NaBH_3CN$ (69.15 mg, 1.10 mmol) was added at 20°C, then the mixture was stirred at 20°C for 12 hrs. LCMS showed Compound 4 was consumed and 60% of desired product was detected. The reaction was basified with saturated Na_2CO_3 solution to pH=8, then the mixture was extracted with DCM : MeOH (10:1) (6 mL*3), the organic layer was dried over anhydrous Na_2SO_4, filtered and the filtrate was concentrated under vacuum to afford Compound 5 (191 mg, crude) as a yellow liquid and it was used directly. MS-ESI (m/z) calcd for $C_{21}H_{25}Cl_2N_3O_2$ [M+H]+: 421.1/423.1. Found 422.2/424.2.To a solution of ethyl Compound 5 (222 mg, 525.63 umol) in THF (5 mL) was added $LiAlH_4$ (23.94 mg, 630.76 umol) at 0°C, then the mixture was stirred at 0°C for 40 min. TLC (Dichloromethane : Methanol=10:1) (P1Rf=0.33) showed Compound 5 was consumed and two spots were formed. The reaction was diluted with 5 mL THF, then 0.5 mL saturated $MgSO_4$ solution was added dropwise at 0°C, then the mixture was dried over anhydrous Na_2SO_4, filtered and the filtrate was concentrated under vacuum. The residue was purified by Prep-TLC (Ethyl acetate : Ethanol=3:1) (P1Rf =0.25) to afford Compound 6 (118 mg, 214.09 umol, 40.73% yield, 69% purity) as a yellow liquid. To a solution of Compound 6 (118 mg, 310.27 umol) in THF (4 mL) was added t-BuOK (104.45 mg, 930.82 umol) at 0°C, then the mixture was stirred at 0°C for 5 min, then Compound 6A (75.77 mg, 620.55 umol) was added at 0°C, then the mixture was stirred at 0°C for 1 h. LCMS showed trace of Compound 6 was remained and 31% of desired product was detected. The reaction was concentrated under vacuum. The residue was purified by Prep-TLC (Dichloromethane : Methanol=10:1) (P1Rf=0.32), the residue was purified by Prep-HPLC (neutral condition) (column: Waters Xbridge BEH C18 100*30mm*10um; mobile phase : [water (10mM NH_4HCO_3) -ACN]; B% : 45%-75%, 10 min) to afford **110** (12.3 mg, 24.45 umol, 7.88% yield, 95.89% purity) as a pale yellow solid. 1H NMR METHANOL -d4 400MHz δ ppm 8.48 (d, J=1.75 Hz, 1 H) 7.93 (dd, J=8.69, 2.31 Hz, 1 H) 7.50 (d, J=2.00 Hz, 1 H) 7.31 - 7.37 (m, 1 H) 7.27 - 7.31 (m, 1 H) 7.03 (d, J=8.63 Hz, 1 H) 6.91 (d, J=8.76 Hz, 1 H) 6.73 (dd, J=8.63, 2.75 Hz, 1 H) 6.52 (d, J=2.75 Hz, 1 H) 4.54 (t, J=5.69 Hz, 2 H) 3.49 (t, J=5.63 Hz, 2 H) 2.75 (br s, 4 H) 2.18 - 2.42 (m, 7 H) LCMS (ESI+): m/z 482.1/484.0 (M+H)

Analog 111

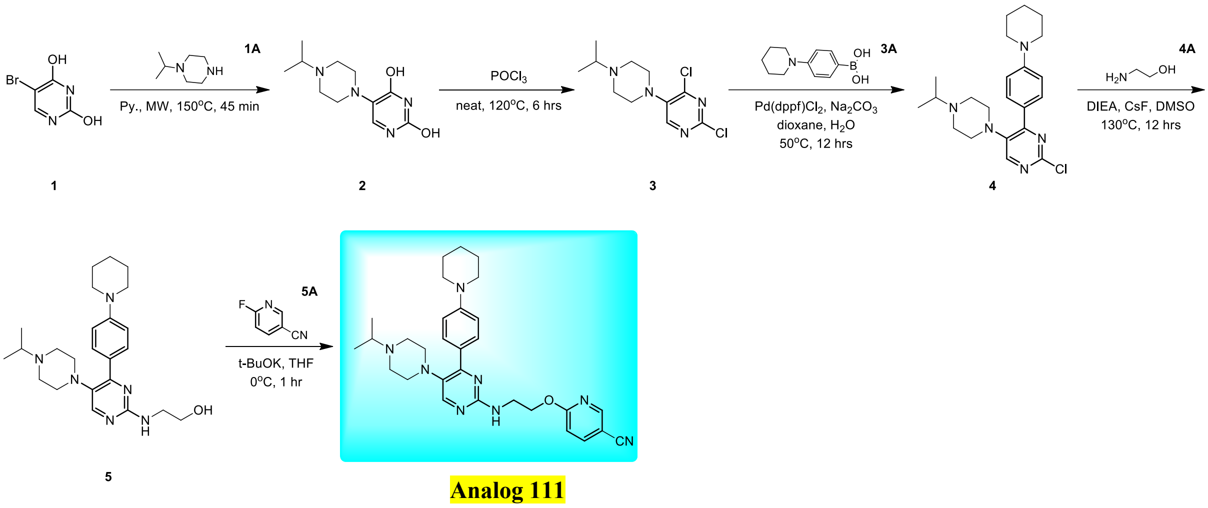

Analog 111

6-(2-((5-(4-isopropylpiperazin-1-yl)-4-(4-(piperidin-1-yl)phenyl)pyrimidin-2-yl)amino)ethoxy)-nicotinonitrile (Analog 111). A solution of Compound 1 (3 g, 15.71 mmol) and Compound 1A (3.02 g, 23.56 mmol, 3.37 mL) in Py. (9.80 g, 123.89 mmol, 10 mL) was stirred at 150°C for 45 min in the microwave. LCMS showed Compound 1 was consumed completely and 81% of desired mass was detected. The reaction mixture was concentrated under reduced pressure to remove solvent. The reaction mixture was stirred in EtOAc (50 mL) for 30 min, then the mixture was filtered and the filter-cake was dried under vacuum to afford Compound 2 (2.5 g, crude) as a gray solid. MS-ESI (m/z) calcd for C11H18N4O2 [M+H]+: 239.1 Found 239.2. Compound 2 (8.4 g, 39.96 mmol) was added into POCl3 (49.50 g, 322.83 mmol, 30 mL) at 20°C. The mixture was stirred at 120°C for 6 hrs. LCMS showed Compound 2 was consumed completely and 57% of desired compound was detected. The reaction mixture was concentrated under reduced pressure to remove solvent. The residue was poured into water (100 mL) slowly, then the aqueous layer was basified with Na2CO3 solid to PH = 8, then the mixture was extracted with EtOAc (100 mL * 3). The combined organic layers were dried over anhydrous Na2SO4, filtered and concentrated under reduced pressure to give a residue. The residue was purified by flash silica gel chromatography (ISCO®; 40 g SepaFlash® Silica Flash Column, Eluent of 0~10% (Dichloromethane : Methanol=10:1)/Petroleum ether gradient @ 75 mL/min) (SiO2, Dichloromethane : Methanol=10:1, P1Rf=0.34) to afford Compound 3 (0.8 g, 3.24 mmol, 8.10% yield) as a yellow solid. MS-ESI (m/z) calcd for C11H16Cl2N4 [M+H]+: 275.1/ 277.1 Found 275.1/277.0. To a solution of Compound 3 (500 mg, 1.82 mmol) and Compound 3A (409.86 mg, 2.00 mmol) in dioxane (6 mL) and H2O (0.9 mL) was added Na2CO3 (577.75 mg, 5.45 mmol) and Pd(dppf)Cl2 (132.95 mg, 181.70 umol). The mixture was stirred at 50°C for 12 hrs under N2. LCMS showed 6% of Compound 3 remained and 60% of desired compound was detected. The reaction mixture was concentrated under reduced pressure to remove solvent. The residue was purified by flash silica gel chromatography (ISCO®; 12 g SepaFlash® Silica Flash Column, Eluent of 0~30% Ethyl acetate/Petroleum ether gradient @ 75 mL/min) (SiO2, Dichloromethane : Methanol=10:1, P1Rf=0.35) to afford Compound 4 (595 mg, 1.49 mmol, 81.87% yield) as a yellow solid. MS-ESI (m/z) calcd for C22H30ClN5 [M+H]+: 400.2/402.2. Found 400.2/402.2. To a solution of Compound 4 (200 mg, 500.05 umol) and Compound 4A (76.36 mg, 1.25 mmol, 75.61 uL) in DMSO (3 mL) was added DIEA (161.57 mg, 1.25 mmol, 217.75 uL) and CsF (113.94 mg, 750.08 umol, 27.66 uL). The mixture was stirred at 130°C for 12 hrs. LCMS showed Compound 4 was consumed and 27% of desired compound was detected. The reaction mixture was diluted with H2O 3 mL and extracted with EtOAc (3 mL * 3) and (Dichloromethane : Methanol=10:1) (5 mL * 3). The combined organic layers were washed with brine (15 mL * 1), dried over anhydrous Na2SO4, filtered

and concentrated under reduced pressure to give a residue. The residue was purified by prep-TLC (SiO_2, DCM : MeOH=10:1, P1Rf=0.23) to afford Compound 5 (190 mg, 447.50 umol, 89.49% yield) as a yellow oil. MS-ESI (m/z) calcd for $C_{24}H_{36}N_6O$ [M+H]+: 425.3. Found 425.3. To a solution of Compound 5 (100 mg, 235.53 umol) in THF (1 mL) was added t-BuOK (79.29 mg, 706.58 umol). The mixture was stirrd at 0°C for 10 min. Then Compound 5A (43.14 mg, 353.29 umol) was added. The mixture was stirrd at 0°C for 1 hr. LCMS and HPLC showed 24% of Compound 5 was remained and 20% of desired compound was detected. The reaction mixture was concentrated under reduced pressure to remove solvent. The residue was purified by prep-HPLC (basic condition, column: Waters Xbridge Prep OBD C18 150*40mm*10um; mobile phase: [water (NH_3H_2O + NH_4HCO_3) - ACN]; B%: 45%-75%, 8 min) to afford **111** (6.17 mg, 11.46 umol, 4.87% yield, 97.84% purity) as a yellow solid. **^{1}H NMR** (METHANOL-d_4 400MHz) δ ppm 8.49 (d, J=2.00 Hz, 1 H) 8.05 (d, J=8.88 Hz, 2 H) 8.02 (s, 1 H) 7.88 (dd, J=8.69, 2.19 Hz, 1 H) 6.96 (d, J=9.01 Hz, 2 H) 6.87 (d, J=8.75 Hz, 1 H) 4.58 (t, J=5.44 Hz, 2 H) 3.81 (t, J=5.44 Hz, 2 H) 3.28 (br s, 4 H) 2.86 (br d, J=4.63 Hz, 4 H) 2.52 - 2.75 (m, 5 H) 1.57 - 1.81 (m, 6 H) 1.10 (d, J=6.50 Hz, 6 H) LCMS (ESI+): m/z 527.4 (M+H)

Analog 112

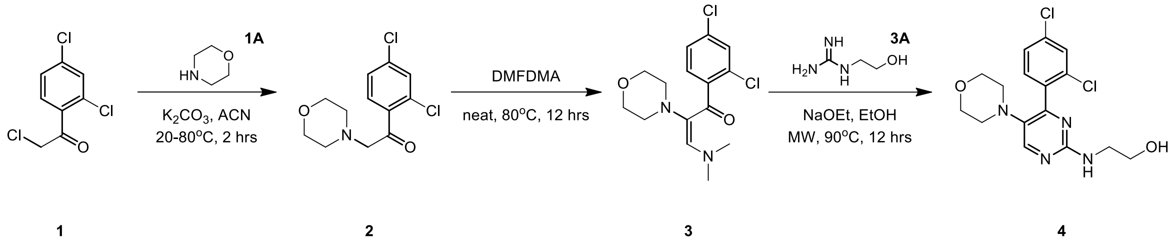

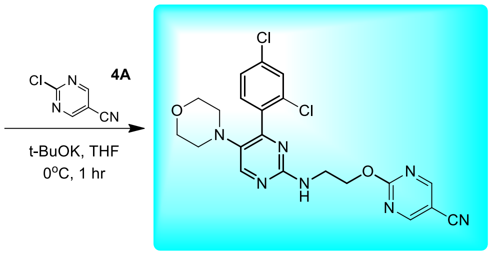

Analog 112

2-(2-((4-(2,4-dichlorophenyl)-5-morpholinopyrimidin-2-yl)amino)ethoxy)pyrimidine-5-carbonitrile (Analog 112). To a solution of Compound 1 (2 g, 8.95 mmol) and Compound 1A (1.56 g, 17.90 mmol, 1.58 mL) in ACN (20 mL) was added K2CO3 (3.71 g, 26.85 mmol) at 20°C. The mixture was stirred at 80°C for 2 hrs. LCMS showed Compound 1 was consumed completely and 70% of desired compound was detected. The reaction mixture was concentrated under reduced pressure to remove solvent. The residue was purified by flash silica gel chromatography (ISCO®; 20 g SepaFlash® Silica Flash Column, Eluent of 0~10% Ethyl acetate/Petroleum ether gradient @ 100 mL/min) (Petroleum ether: Ethyl acetate=1:1, P1Rf =0.27) to afford Compound 2 (500 mg, 1.82 mmol, 20.38% yield) as a yellow oil. MS-ESI (m/z) calcd for C12H13Cl2NO2 [M+H]+: 274.0/ 276.0 Found.274.2/276.2 A solution of Compound 2 (500 mg, 1.82 mmol) in DMFDMA (4.22 g, 24.21 mmol, 5 mL) was stirred at 80°C for 12 hrs. LCMS showed Compound 2 was consumed completely and 16% of desired compound was detected. The reaction mixture was concentrated under reduced pressure to remove solvent. The residue was purified by flash silica gel chromatography (ISCO®; 12g SepaFlash® Silica Flash Column, Eluent of 0~10% Ethyl acetate/Petroleum ether gradient @ 100mL/ min) (SiO2, Petroleum ether: Ethyl acetate=1:1, P1Rf =0.33) to afford Compound 3 (177 mg, 537.63 umol, 29.48% yield) as a yellow solid. MS-ESI (m/z) calcd for C15H18Cl2N2O2 [M+H]+: 329.1/ 331.1 Found 329.1/331.1. To a solution of Compound 3 (150 mg, 455.62 umol) and Compound 3A (93.97 mg, 911.24 umol) in EtOH (3 mL) was added EtONa (124.02 mg, 1.82 mmol) at 20°C. The mixture was stirred at 90°C for 12 hrs. LCMS showed 9% of Compound 3 was remained and 59% of desired compound was detected. The reaction mixture was concentrated under reduced pressure to remove solvent. The residue was purified by prep-TLC (SiO2, Ethyl acetate: Petroleum ether=2:1, P1Rf=0.21) to afford Compound 4 (73 mg, 197.70 umol, 43.39% yield) as a yellow oil. MS-ESI (m/z) calcd for C16H18Cl2N4O2 [M+H]+: 369.1/ 371.1. Found 369.0/371.0. To a solution of Compound 4 (63 mg, 170.62 umol) in THF (1 mL) was added t-BuOK (57.44 mg, 511.86 umol) at 0°C. The mixture was stirred at 0°C for 10 min, then Compound 4A (59.52 mg, 426.55 umol) was added, the mixture was stirred at 0°C for 1 hr. LCMS and HPLC showed 19% of Compound 4 was remained and 37% of desired compound was detected. The reaction mixture was concentrated under reduced pressure to remove solvent. The residue was purified by prep-HPLC (basic condition, column: Waters Xbridge BEH C18 100*30mm*10um ; mobile phase : [water (NH3H2O + NH4HCO3) - ACN] ; B%: 25%-65%, 8 min). The residue was purified by prep-HPLC (basic condition, column: Waters Xbridge BEH C18 100*30mm*10um; mobile phase: [water (NH3H2O + NH4HCO3) -

ACN]; B% : 30%-60%, 8 min) to afford **112** (6.74 mg, 13.88 umol, 8.14% yield, 97.27% purity) as a yellow solid. **^{1}H NMR** (METHANOL-d_4 400MHz) δ ppm 8.85 (s, 2 H) 8.22 (s, 1 H) 7.58 (d, J=1.97 Hz, 1 H) 7.42 (d, J=1.75 Hz, 1 H) 7.41 (s, 1 H) 4.64 (t, J=5.37 Hz, 2 H) 3.82 (t, J=5.48 Hz, 2 H) 3.45 - 3.53 (m, 4 H) 2.71 - 2.78 (m, 4 H). LCMS (ESI+): m/z 472.1/474.1 (M+H)

Analog 113

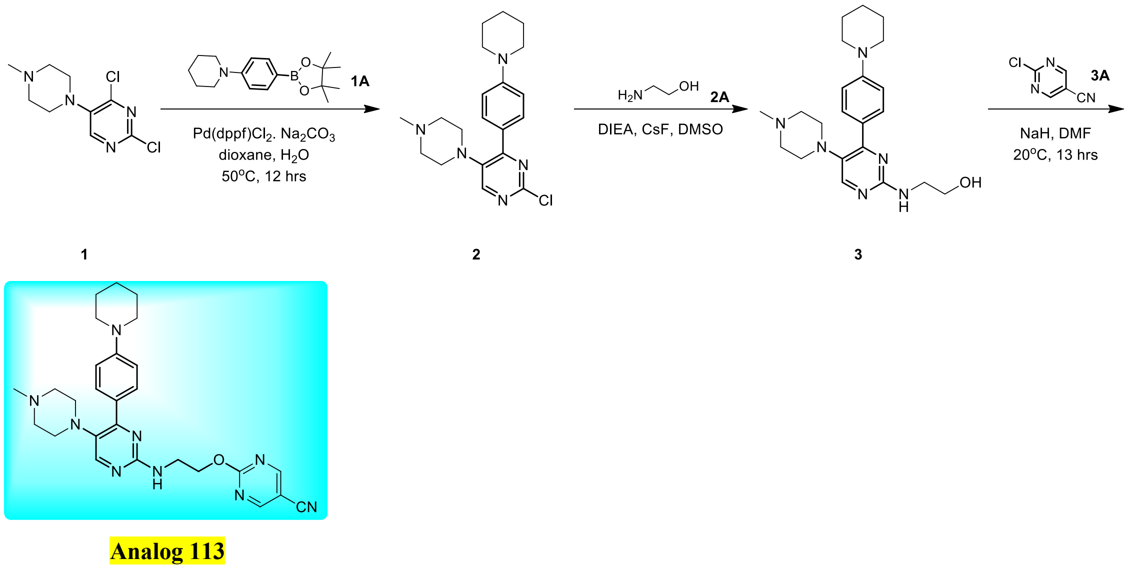

2-(2-((5-(4-methylpiperazin-1-yl)-4-(4-(piperidin-1-yl)phenyl)pyrimidin-2-yl)amino)ethoxy)-pyrimidine-5-carbonitrile (Analog 113). To a solution of Compound 1 (200 mg, 809.31 umol) and Compound 1A (348.66 mg, 1.21 mmol) in dioxane (4 mL) and H2O (0.6 mL) was added Na2CO3 (257.33 mg, 2.43 mmol) and Pd(dppf)Cl2 (59.22 mg, 80.93 umol) at 20°C. The mixture was stirred at 50°C for 12 hrs under N2. LCMS showed Compound 1 was consumed completely and 42% of desired compound was detected. The reaction mixture was concentrated under reduced pressure to remove solvent. The residue was purified by flash silica gel chromatography (ISCO®; 12 g SepaFlash® Silica Flash Column, Eluent of 0~20% (Dichloromethane : Methanol=10:1)/Petroleum ether gradient @ 50mL /min) (SiO2,

Dichloromethane : Methanol=10:1, P1Rf=0.41, plate1) to afford Compound 2 (196 mg, 527.02 umol, 65.12% yield) as a yellow solid. MS-ESI (m/z) calcd for C20H26ClN5 [M+H]+: 372.2/374.2 Found 372.1/374.1. To a solution of Compound 2 (248 mg, 666.84 umol) and Compound 2A (101.83 mg, 1.67 mmol, 100.82 uL) in DMSO (2 mL) was added DIEA (258.55 mg, 2.00 mmol, 348.45 uL) and CsF (151.94 mg, 1.00 mmol, 36.88 uL). The mixture was stirred at 130°C for 12 hrs. LCMS showed Compound 2 was consumed completely and 48% of desired compound was detected. The reaction mixture was concentrated under reduced pressure to remove solvent. The residue was diluted with H2O 2 mL and extracted with EtOAc (2 mL * 3). The combined organic layers were washed with solvent brine (5 mL * 1), dried over anhydrous Na2SO4, filtered and concentrated under reduced pressure to give a residue. The residue was purified by flash silica gel chromatography (ISCO®; 12 g SepaFlash® Silica Flash Column, Eluent of 0~15% (Dichloromethane: Methanol=10:1)/Petroleum ether gradient @ 50 mL/min) (SiO2, Dichloromethane : Methanol=10:1, P1Rf=0.27) to afford Compound 3 (140 mg, 323.58 umol, 48.53% yield, 91.65% purity) as a yellow solid. MS-ESI (m/z) calcd for C22H32N6O [M+H]+: 397.3 Found 397.2. To a solution of Compound 3 (100 mg, 252.19 umol) in DMF (2 mL) was added NaH (50.43 mg, 1.26 mmol, 60% purity) at 0°C. The mixture was stirred at 0°C for 1 hr, then Compound 3A (140.76 mg, 1.01 mmol) was added, the mixture was stirred at 20°C for 12 hrs. LCMS showed 4% of Compound 3 was consumed completely and 26% of desired compound was detected. The reaction mixture was filtered. The residue was purified by prep-HPLC (TFA condition, column : Phenomenex Luna 80*30mm*3um; mobile phase : [water (TFA) - ACN]; B% : 5%-35%, 8 min). The residue was purified by prep-HPLC (basic condition, column: Waters Xbridge BEH C18 100*30mm*10um; mobile phase: [water (NH3H2O + NH4HCO3) - ACN]; B% : 25%-65%, 8 min) to afford **113** (12.7 mg, 24.79 umol, 9.83% yield, 97.52% purity) as a yellow solid. **^{1}H NMR** (METHANOL-d_4 400MHz) δ ppm 8.80 (s, 2 H) 7.96 - 8.03 (m, 3 H) 6.95 (br d, J=9.01 Hz, 2 H) 4.66 (br t, J=5.19 Hz, 2 H) 3.87 (br t, J=5.13 Hz, 2 H) 3.27 - 3.30 (m, 4 H) 2.85 (br s, 4 H) 2.52 (br s, 4 H) 2.31 (s, 3 H) 1.60 - 1.77 (m, 6 H). LCMS (ESI+): m/z 500.3 (M+H)

Analog 114

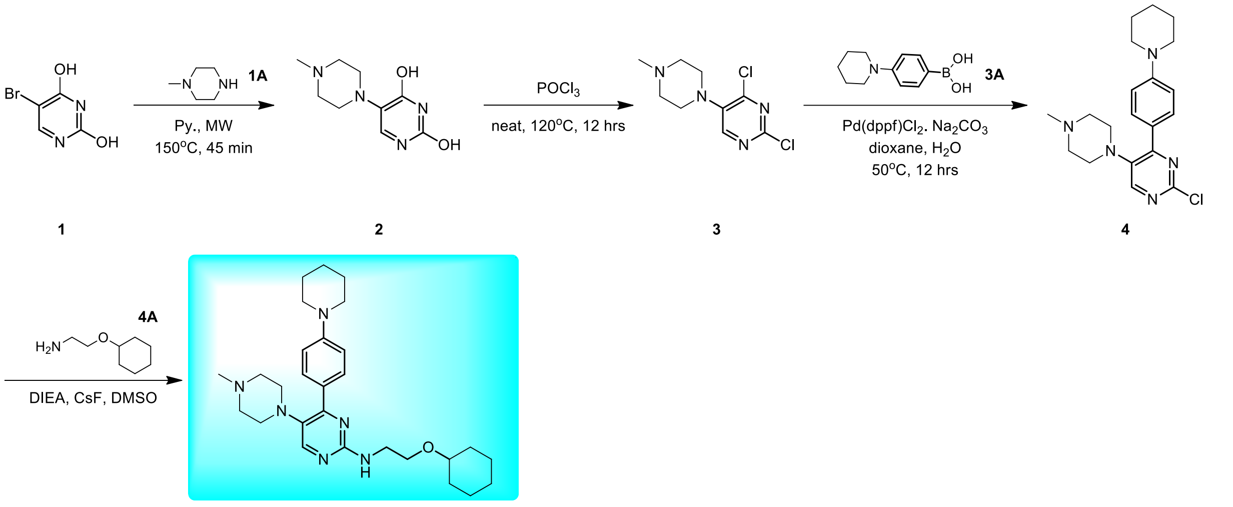

A-135

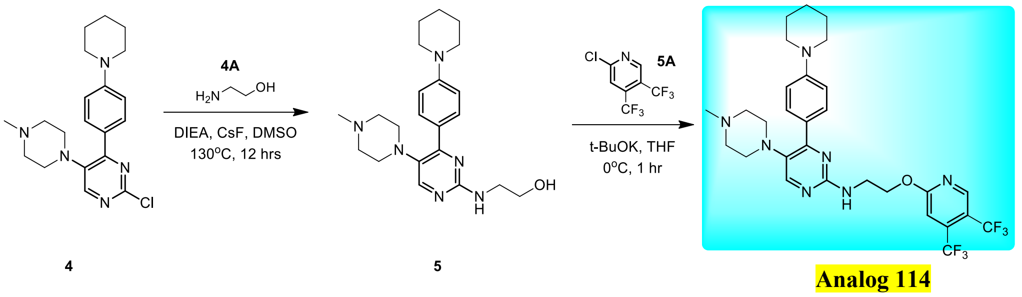

Analog 114

A solution of Compound 1 (3 g, 15.71 mmol) and Compound 1A (2.36 g, 23.56 mmol, 2.61 mL) in Py. (1.24 g, 15.71 mmol, 1.27 mL). The mixture was stirred at 150°C for 45 min in the microwave. LCMS showed Compound 1 was consumed completely and 91% of desired compound was detected. The reaction mixture was concentrated under vacuum. The residue was diluted with EtOAc (30 mL). The mixture was stirred at 20°C for 30 min. Then the mixture was filtered and the filter-cake was diluted with H2O (10 mL) and MeOH (10 mL) and then dried under vacuum to afford Compound 2 (7 g, crude) as a yellow solid. MS-ESI (m/z) calcd for C9H14N4O2 [M+H]+: 211.1 Found 211.3. Compound 2 (7.27 g, 34.58 mmol) was added into POCl3 (33.32 g, 217.31 mmol, 20.19 mL) at 20°C. The mixture was stirred at 120°C for 12 hrs. LCMS showed Compound 2 was consumed completely and 46% of desired compound was detected. The reaction mixture was concentrated under reduced pressure to remove solvent. Then EtOAc (100 mL) was added into the residue, then the organic layer was slowly pour into the saturated NaHCO3 solution to PH = 8, then the mixture was extracted with EtOAc (100 mL * 3) and (Dichloromethane : Methanol=10:1) (100 mL * 3). The combined organic layers were dried over anhydrous Na2SO4, filtered and concentrated under reduced pressure to give a residue. The residue was purified by flash silica gel chromatography (ISCO®; 40 g SepaFlash® Silica Flash Column, Eluent of 0~10% (Dichloromethane : Methanol=10:1)/Petroleum ether gradient @ 75 mL/min) (SiO2, Dichloromethane : Methanol=10:1, Rf(P1)=0.34) to afford Compound 3 (536 mg, 2.12 mmol, 6.13% yield, 97.76% purity) as a yellow solid. MS-ESI (m/z) calcd for C9H12Cl2N4 [M+H]+: 247.0/249.0. Found 347.2/249.2. To a solution of Compound 3 (200 mg, 809.31 umol) and Compound 3A (165.96 mg, 809.31 umol) in dioxane (4 mL) and H2O (0.6 mL) was added Na2CO3 (257.33 mg, 2.43 mmol) and Pd(dppf)Cl2 (59.22 mg, 80.93 umol). The mixture was stirred at 50°C for 12 hrs under N2. LCMS showed 10% of Compound 3 was remained and 42% of desired compound was detected. The reaction mixture was concentrated under reduced pressure to remove solvent. The residue was purified by flash

silica gel chromatography (ISCO®; 12 g SepaFlash® Silica Flash Column, Eluent of 0~30% (Dichloromethane : Methanol=10:1)/Petroleum ether gradient @ 75 mL/min) (SiO2, Dichloromethane : Methanol=10:1, Rf(P1)=0.38) to afford Compound 4 (179 mg, 380.23 umol, 46.98% yield, 79% purity) as a yellow solid. MS-ESI (m/z) calcd for C20H26ClN5 [M+H]+: 372.2/374.2. Found 372.3/374.3. To a solution of Compound 4 (100 mg, 268.89 umol) and Compound 4A (96.28 mg, 672.21 umol) in DMSO (2 mL) was added DIEA (173.76 mg, 1.34 mmol, 234.17 uL) and CsF (61.27 mg, 403.33 umol, 14.87 uL). The mixture was stirred at 140 °C for 12 hrs in the microwave. LCMS and HPLC showed Compound 4 was consumed completely and 11% of desired compound was detected. The reaction mixture was filtered. The residue was purified by prep-HPLC (basic condition, column: Waters Xbridge Prep OBD C18 150*40mm*10um; mobile phase: [water (NH3H2O + NH4HCO3) - ACN]; B%: 40%-80%, 8 min) to afford **A-135** (20 mg, 41.10 umol, 15.29% yield, 98.37% purity) as a yellow solid. **^{1}H NMR** (METHANOL-*d*4 400MHz) δ ppm 8.09 (br d, J=9.00 Hz, 2 H) 8.03 (s, 1 H) 6.99 (d, J=8.88 Hz, 2 H) 3.60 - 3.69 (m, 2 H) 3.50 - 3.59 (m, 2 H) 3.26 - 3.30 (m, 5 H) 2.86 (br t, J=4.32 Hz, 4 H) 2.41 - 2.63 (m, 4 H) 2.31 (s, 3 H) 1.84 - 2.00 (m, 1 H) 1.83 - 1.97 (m, 1 H) 1.39 - 1.81 (m, 10 H) 1.28 (q, J=9.01 Hz, 4 H)LCMS (ESI+): m/z 479.4 (M+H)

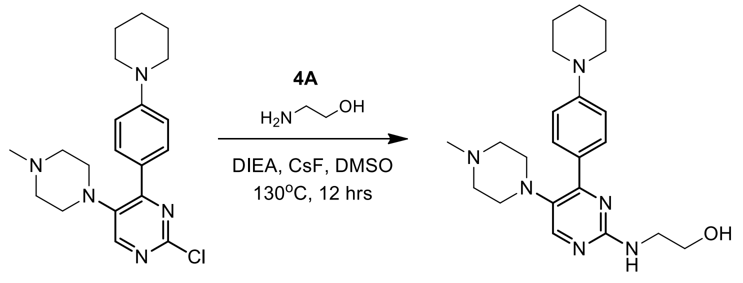

***N*-(2-((4,5-bis(trifluoromethyl)pyridin-2-yl)oxy)ethyl)-5-(4-methylpiperazin-1-yl)-4-(4-(piperidin-1-yl)phenyl)pyrimidin-2-amine (Analog 114)**. To a solution of Compound 4 (157 mg, 422.15 umol) and Compound 4A (64.47 mg, 1.06 mmol, 63.83 uL) in DMSO (2 mL) was added DIEA (163.68 mg, 1.27 mmol, 220.59 uL) and CsF (96.19 mg, 633.22 umol, 23.35 uL). The mixture was stirred at 130°C for 12 hrs. LCMS showed Compound 4 consumed and 24% of desired compound was detected. The residue was diluted with H2O 3 mL and extracted with Ethyl acetate (3 mL * 3). The combined organic layers were washed with brine (3mL * 3), filtered and concentrated under reduced pressure to give a residue. The residue was purified by prep-TLC (SiO2, Dichloromethane: Methanol= 10:1, Rf(P1)=0.40) to afford Compound 5 (36 mg, 90.79 umol, 21.51% yield) as a yellow solid. MS-ESI (m/z) calcd for C22H32N6O [M+H]+: 396.3. Found 397.4 A solution of Compound 5 (27 mg, 68.09 umol) in THF (2 mL) was added t-BuOK (22.92 mg, 204.27 umol). The mixture was stirred at 0°C for 20 min. Then Compound 5A (25.49 mg, 102.14 umol) was added. The mixture was stirred at 0°C for 1 hr under N2. LCMS and HPLC showed 8% of Compound 5 remained and 63% of desired compound was detected. The reaction mixture was concentrated under reduced pressure to remove solvent. The residue was purified by prep-HPLC (TFA condition, column: Phenomenex Luna 80*30mm*3um; mobile phase: [water (TFA) - ACN]; B%: 30%-60%, 8 min) to afford **114** (19.3 mg, 26.47 umol, 38.88% yield, 99.25% purity, TFA) as a yellow solid. **^{1}H NMR** (METHANOL-*d*4 400MHz) δ ppm 8.68 (s, 1 H) 8.22 (d, J=9.21 Hz, 2 H) 8.01 (s, 1 H) 7.23 (s, 1 H) 7.15 (br d, J=8.77 Hz, 2 H) 4.71 (t, J=5.26 Hz, 2 H) 3.90 -

4.06 (m, 2 H) 3.43 - 3.62 (m, 6 H) 3.22 - 3.30 (m, 4 H) 2.98 - 3.07 (m, 2 H) 2.96 (s, 3 H) 1.76 (br d, J=6.14 Hz, 6 H). LCMS (ESI+): m/z 610.1 (M+H)

Analog 115

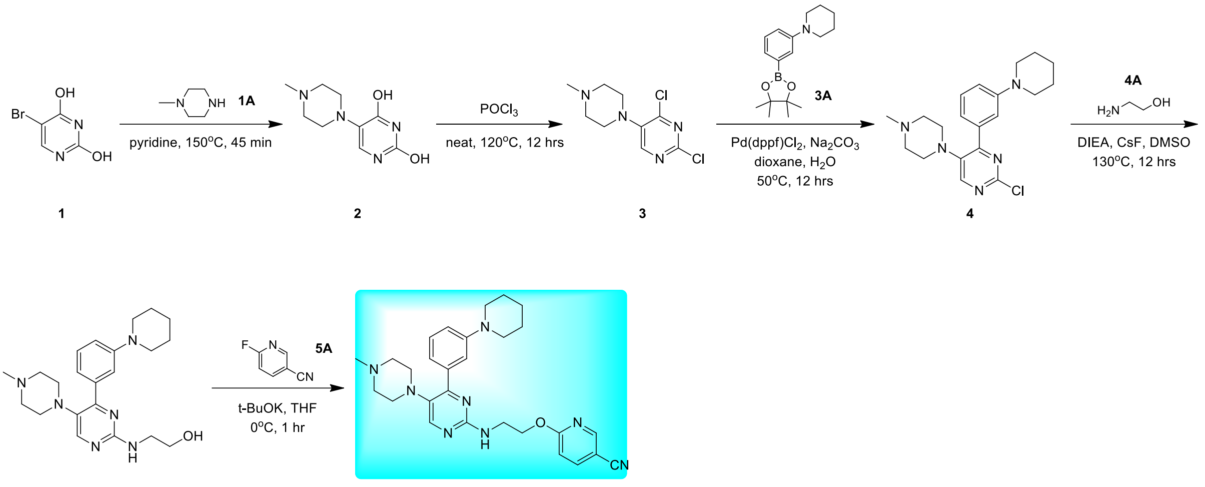

Analog 115

6-(2-((5-(4-methylpiperazin-1-yl)-4-(3-(piperidin-1-yl)phenyl)pyrimidin-2-yl)amino)ethoxy)nicotinonitrile (Analog 115). To a solution of **Compound 1** (2 g, 10.47 mmol) and **Compound 1A** (1.57 g, 15.71 mmol, 1.74 mL) in pyridine (6.53 g, 82.60 mmol, 6.67 mL) at 20°C. The mixture was stirred at 150°C for 45min in the microwave. LCMS showed **Compound 1** was consumed completely and 99% of desired compound was detected. The reaction mixture was concentrated under vacuum. The residue was diluted with EtOAc (30 mL). The mixture was stirred at 20°C for 30 min. Then the mixture was filtered and the filter-cake was dried under vacuum to afford ***Compound 2*** (3.14 g, crude) as a yellow solid. MS-ESI (m/z) calcd for $C_9H_{14}N_4O_2$ [M-H]: 209.1 Found 209.1. **Compound 2** (7.27 g, 34.58 mmol) was added into $POCl_3$ (33.32 g, 217.31 mmol, 20.19 mL) at 20°C. The mixture was stirred at 120°C for 12 hrs. LCMS showed **Compound 2** was consumed completely and 46% of desired compound was detected. The reaction mixture was concentrated under reduced pressure to remove solvent. Then EtOAc (100 mL) was added into the residue, then the organic layer was slowly poured into the saturated $NaHCO_3$ solution to PH = 8, then the mixture was extracted with EtOAc (100 mL * 3) and (Dichloromethane : Methanol=10:1) (100 mL * 3). The combined organic layers were dried over anhydrous Na_2SO_4, filtered and concentrated under reduced pressure to give a residue. The residue was purified by flash silica gel chromatography (ISCO®; 40 g SepaFlash® Silica Flash Column, Eluent of 0~10% (Dichloromethane : Methanol=10:1)/Petroleum ether gradient @ 75 mL/min) (SiO_2, Dichloromethane : Methanol=10:1, Rf(P1)=0.34) to afford ***Compound 3*** (536 mg, 2.12 mmol, 6.13% yield, 97.76% purity) as a yellow solid. MS-ESI (m/z) calcd for $C_9H_{12}Cl_2N_4$ $[M+H]^+$:247.0/249.0.Found 247.2/249.2. To a solution of **Compound 3** (200 mg, 809.31 umol) and **Compound 3**A (232.44 mg, 809.31 umol) in dioxane (3 mL) and H_2O (0.45 mL) was added Na_2CO_3 (257.33 mg, 2.43 mmol) and $Pd(dppf)Cl_2$ (59.22 mg, 80.93 umol). The mixture was stirred at 50°C for 12 hrs under N_2. LCMS showed **Compound 3** was consumed completely and 29% of desired compound was detected. The reaction mixture was concentrated under reduced pressure to remove solvent. The residue was purified

by prep-TLC (SiO_2, DCM: MeOH = 10:1, Rf(P1)=0.20) to afford ***Compound 4*** (50 mg, 108.90 umol, 13.46% yield, 81% purity) as a yellow oil. MS-ESI (m/z) calcd for $C_{20}H_{26}ClN_5$ $[M+H]^+$:372.2/374.2.Found 372.3/374.3 To a solution of **Compound 4** (50 mg, 134.44 umol) and **Compound 4A** (16.42 mg, 268.89 umol, 16.26 uL) in DMSO (1 mL) was added CsF (30.63 mg, 201.66 umol, 7.44 uL) and DIEA (52.13 mg, 403.33 umol, 70.25 uL). The mixture was stirred at 130°C for 12 hrs. LCMS showed **Compound 4** was consumed completely and 74% of desired compound was detected. The reaction mixture was diluted with H_2O 2 mL and extracted with EtOAc (2 mL * 3). The combined organic layers were washed with brine (5 mL * 1), dried over anhydrous Na_2SO_4, filtered and concentrated under reduced pressure to give a residue. The residue was purified by prep-TLC (SiO_2, DCM: MeOH = 20:1, Rf(P1)=0.32) to afford ***Compound 5*** (34 mg, 80.60 umol, 59.95% yield, 94% purity) as a yellow oil. MS-ESI (m/z) calcd for $C_{22}H_{32}N_6O$ [M-H]:397.3.Found 397.3. To a solution of **Compound 5** (34 mg, 85.74 umol) in THF (1 mL) was added t-BuOK (19.24 mg, 171.49 umol) at 0°C. Then **Compound 5A** (15.70 mg, 128.62 umol) was added. The mixture was stirred at 0°C for 1 hr. LCMS showed 82% of **Compound 5** was remained and 15% of desired compound was detected. Then the mixture was stirred at 0°C for 1 hr. LCMS showed 81% of **Compound 5** was remained and 18% of desired compound was detected. The reaction mixture was concentrated under reduced pressure to remove solvent. The residue was purified by prep-HPLC (TFA condition, column: Phenomenex Luna 80*30mm*3um; mobile phase: [water (TFA) - ACN]; B%: 15%-40%, 8 min) to afford ***115*** (9.06 mg, 14.36 umol, 16.74% yield, 97.07% purity, TFA) as a yellow oil. ^{1}**H NMR** (METHANOL-d_4 400MHz) δ ppm 8.47 (s, 1 H) 8.28 (s, 1 H) 8.20 (d, J=7.65 Hz, 1 H) 8.14 (s, 1 H) 7.89 (br d, J=7.53 Hz, 1 H) 7.75 - 7.80 (m, 1 H) 7.67 - 7.74 (m, 1 H) 6.86 (d, J=8.66 Hz, 1 H) 4.59 (t, J=5.27 Hz, 2 H) 3.88 (br t, J=5.14 Hz, 2 H) 3.66 (br t, J=5.08 Hz, 4 H) 3.47 (br d, J=6.78 Hz, 2 H) 3.02 - 3.20 (m, 6 H) 2.93 (s, 3 H) 2.05 (br s, 4 H) 1.81 (br s, 2 H). **LCMS** (ESI+): *m/z* 499.2 (M+H)

THE END

Supporting information 2 – Compound HNMR and Quality Control

Compound 4

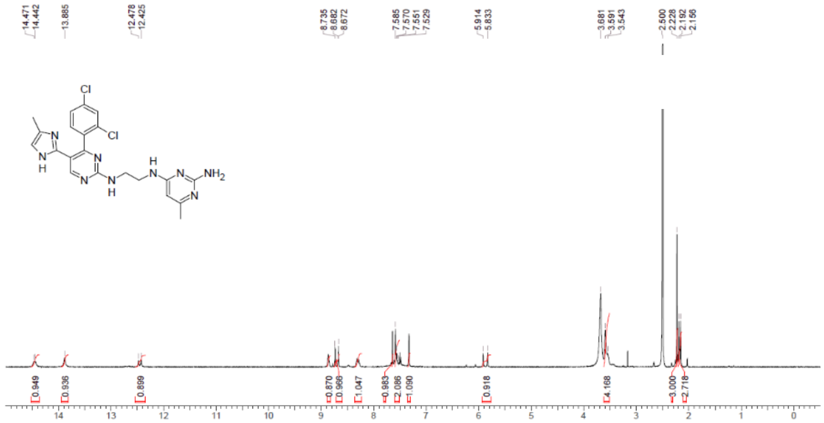

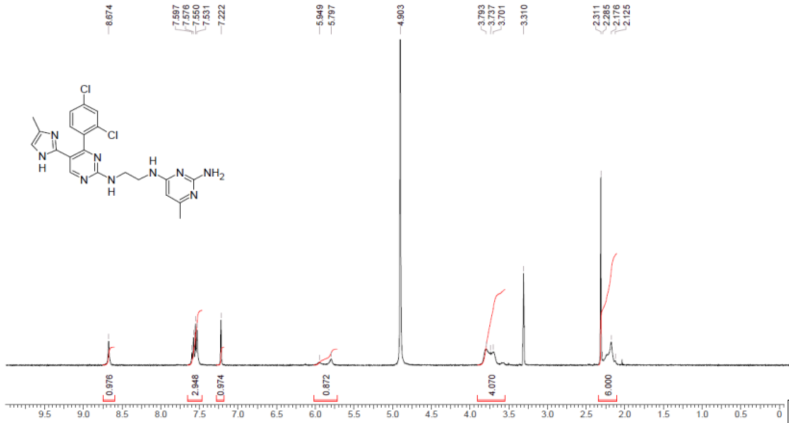

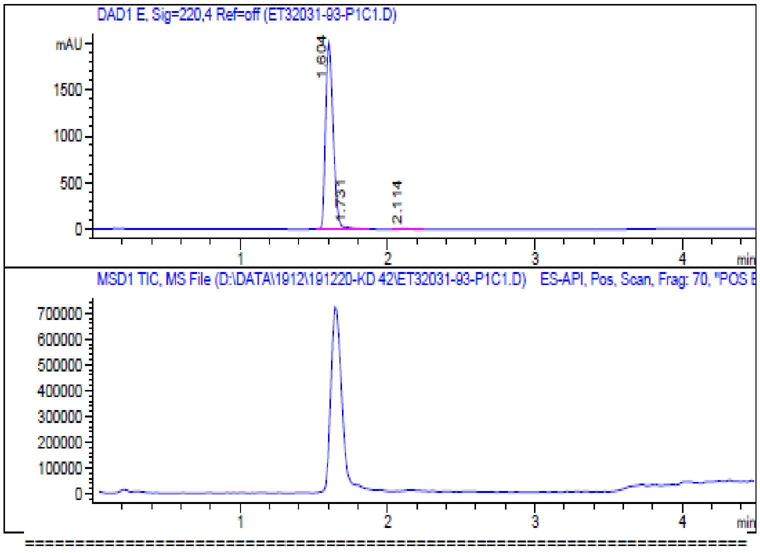

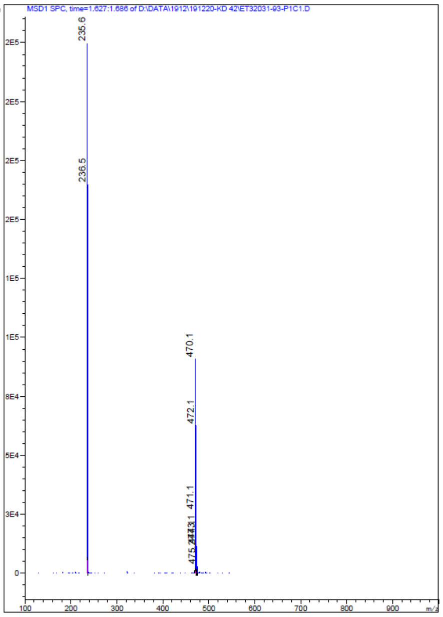

Compound 9

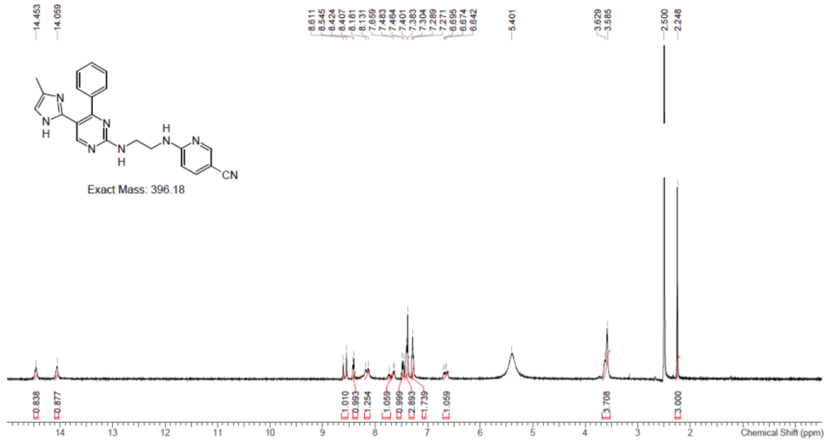

Exact Mass: 396.18
CN
Chemical Shift (ppm)

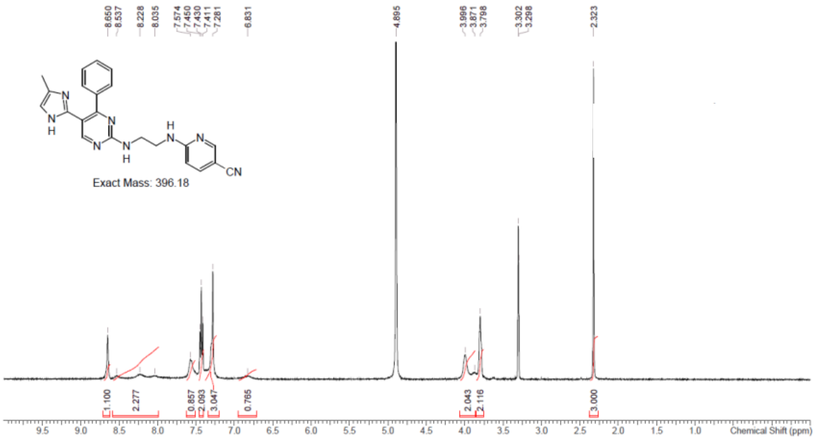

Exact Mass: 396.18
CN
Chemical Shift (ppm)

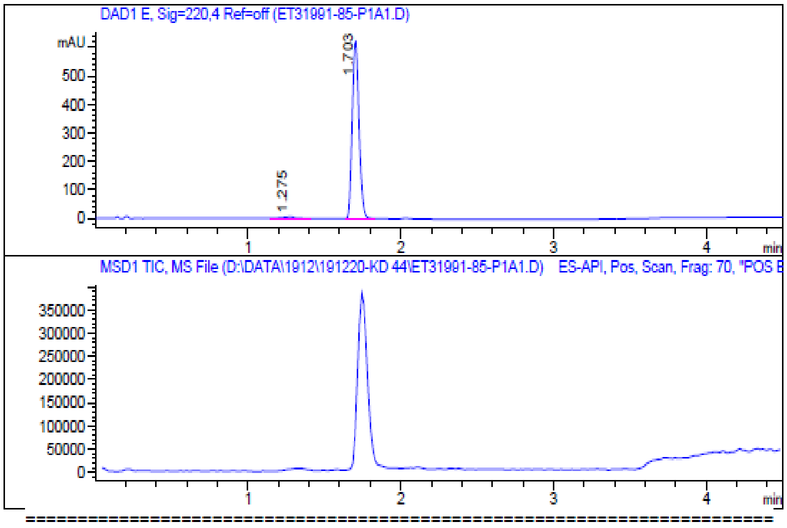

DAD1 E, Sig=220,4 Ref=off (ET31991-85-P1A1.D)
mAU
1.703
1.275
MSD1 TIC, MS File (D:\DATA\1912\191220-KD 44\ET31991-85-P1A1.D) ES-API, Pos, Scan, Frag: 70, "POS E
min

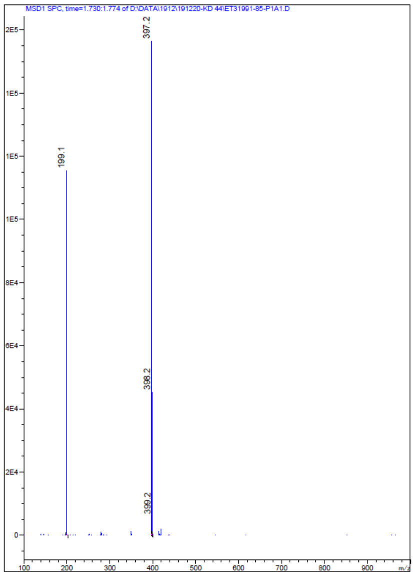

397.2
199.1
398.2
399.2

Compound 10

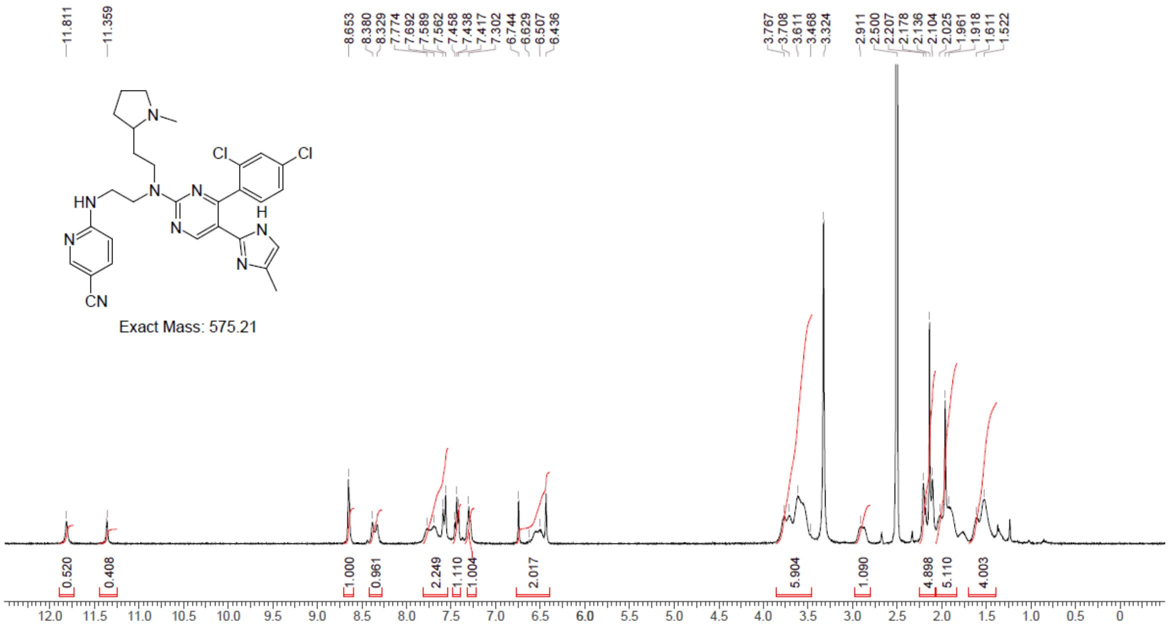
Exact Mass: 575.21

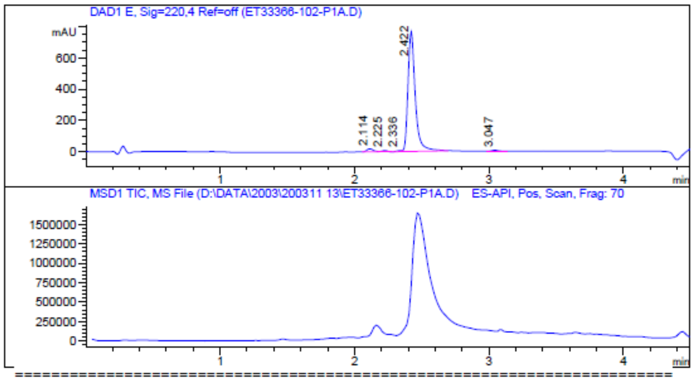
DAD1 E, Sig=220,4 Ref=off (ET33366-102-P1A.D)
MSD1 TIC, MS File (D:\DATA\2003\200311 13\ET33366-102-P1A.D) ES-API, Pos, Scan, Frag: 70

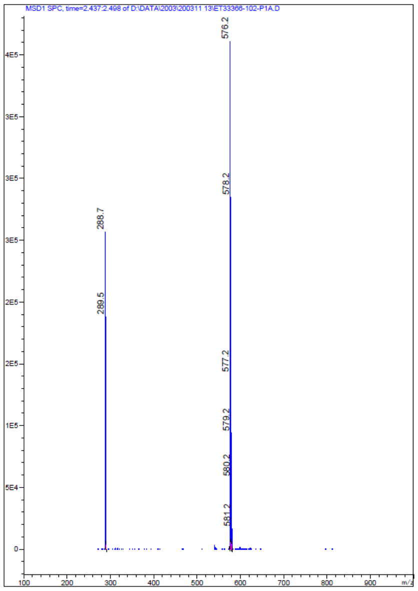
576.2
578.2
288.7
289.5

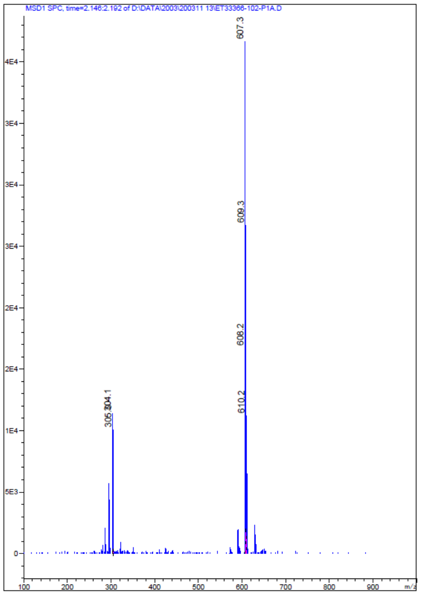
607.3
609.3
608.2
610.2

Compound 11

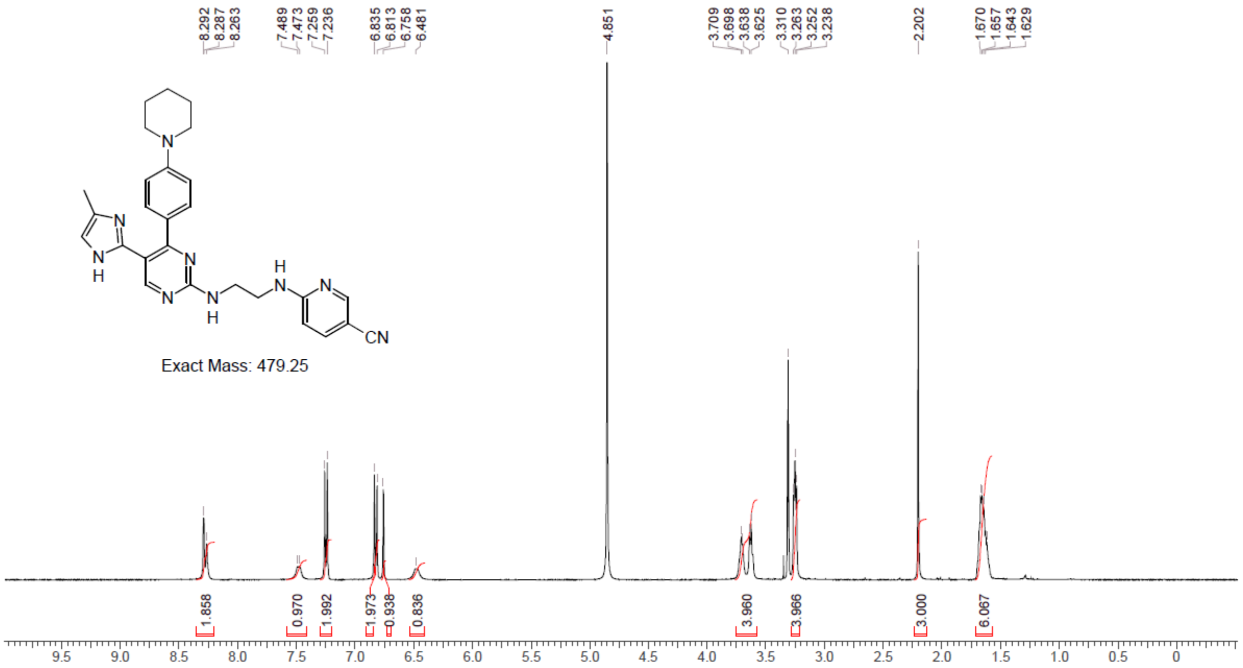

8.292
8.287
8.263
7.489
7.473
7.259
7.236
6.835
6.813
6.758
6.481
4.851
3.709
3.698
3.638
3.625
3.310
3.263
3.252
3.238
2.202
1.670
1.657
1.643
1.629
Exact Mass: 479.25
1.858
0.970
1.992
1.973
0.938
0.836
3.960
3.966
3.000
6.067

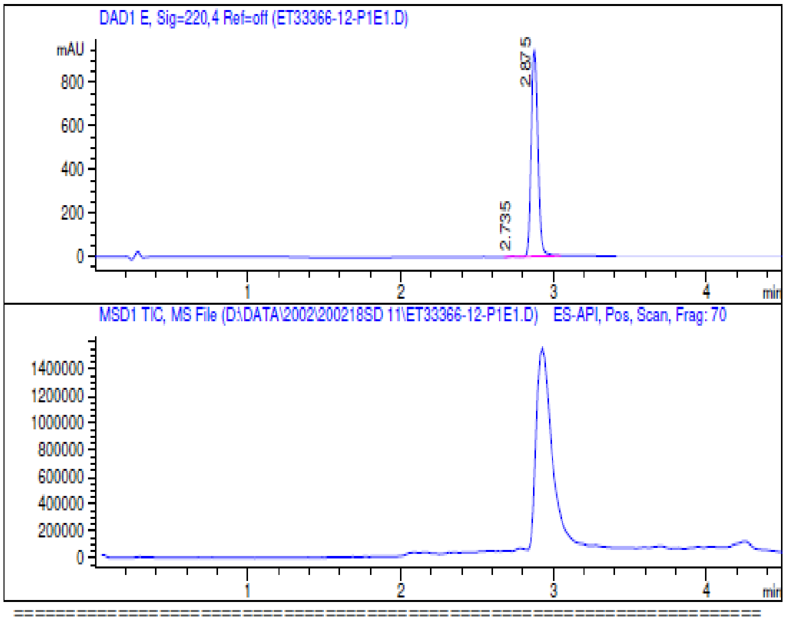

DAD1 E, Sig=220,4 Ref=off (ET33366-12-P1E1.D)
mAU
2.875
2.735
MSD1 TIC, MS File (D:\DATA\2002\200218SD 11\ET33366-12-P1E1.D) ES-API, Pos, Scan, Frag: 70

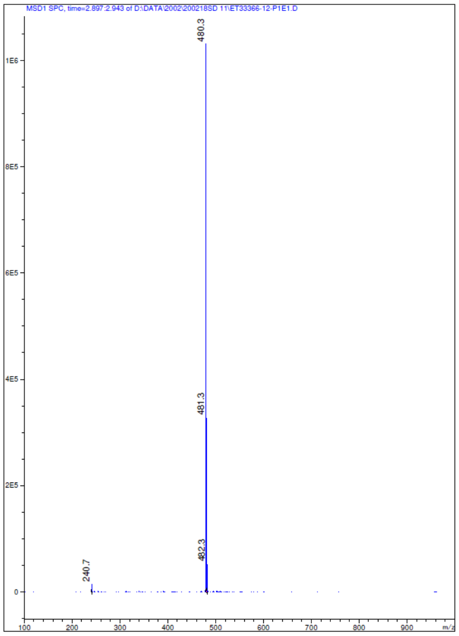

480.3

Compound 12

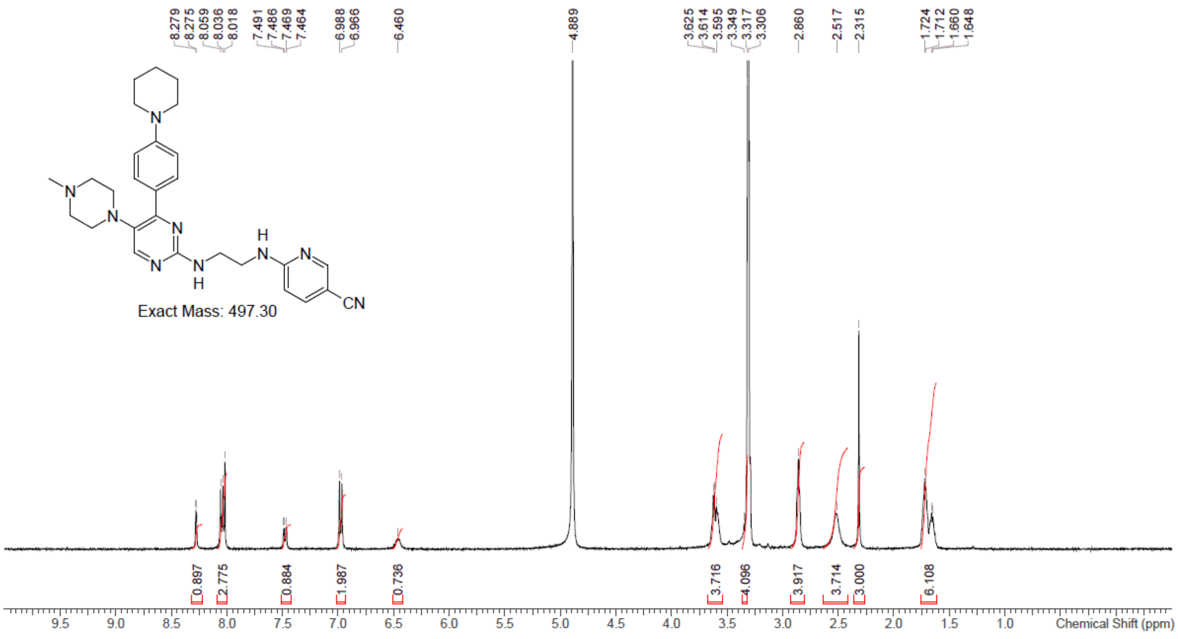
Exact Mass: 497.30
8.279
8.275
8.059
8.036
8.018
7.491
7.486
7.469
7.464
6.988
6.966
6.460
4.889
3.625
3.614
3.595
3.349
3.317
3.306
2.860
2.517
2.315
1.724
1.712
1.660
1.648
0.897
2.775
0.884
1.987
0.736
3.716
4.096
3.917
3.714
3.000
6.108
Chemical Shift (ppm)

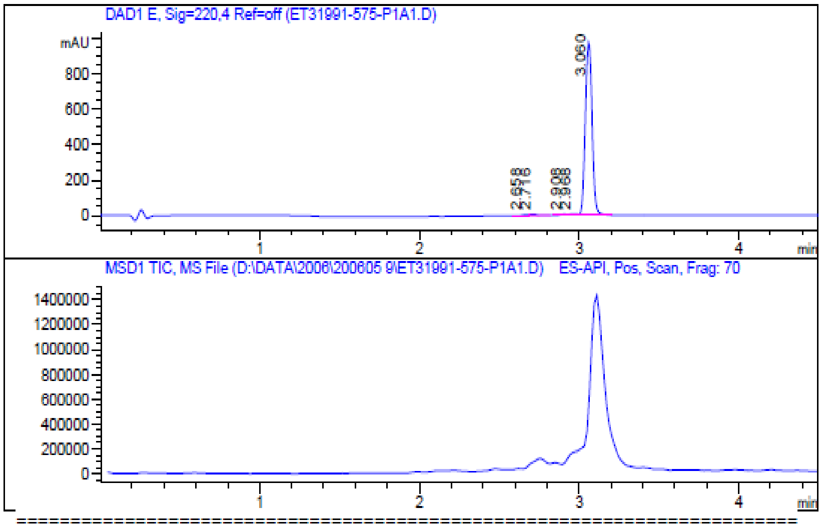
DAD1 E, Sig=220,4 Ref=off (ET31991-575-P1A1.D)
mAU
3.060
min
MSD1 TIC, MS File (D:\DATA\2006\200605 9\ET31991-575-P1A1.D) ES-API, Pos, Scan, Frag: 70
min

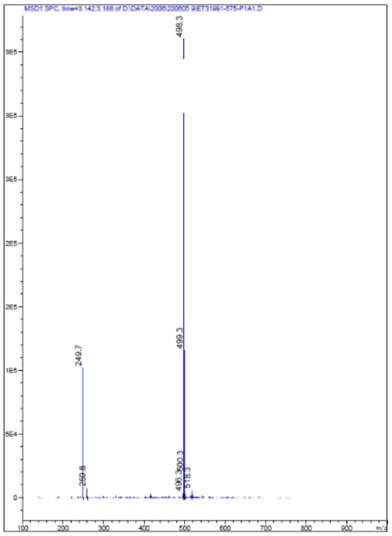
498.3
499.3
249.7
250.8
500.3
518.3

Compound 13

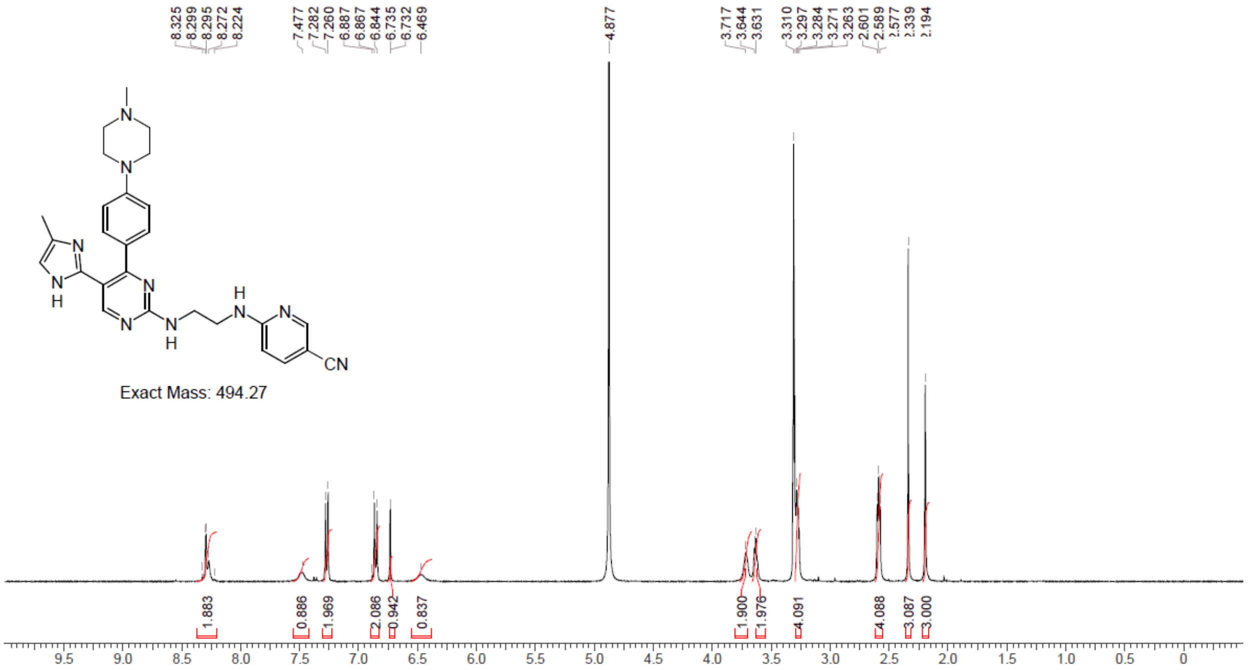

Exact Mass: 494.27
8.325
8.299
8.295
8.272
8.224
7.477
7.282
7.260
6.887
6.867
6.844
6.735
6.732
6.469
4.877
3.717
3.644
3.631
3.310
3.297
3.284
3.271
3.263
2.601
2.589
1.883
0.886
1.969
2.086
0.942
0.837
1.900
1.976
4.091
4.088
3.087
3.000

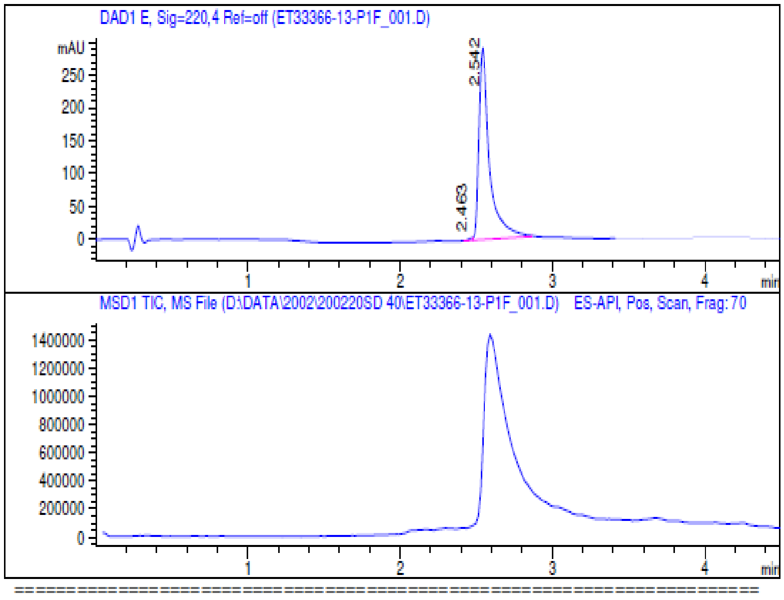

DAD1 E, Sig=220,4 Ref=off (ET33366-13-P1F_001.D)
mAU
2.542
2.463
MSD1 TIC, MS File (D:\DATA\2002\200220SD 40\ET33366-13-P1F_001.D) ES-API, Pos, Scan, Frag: 70
min

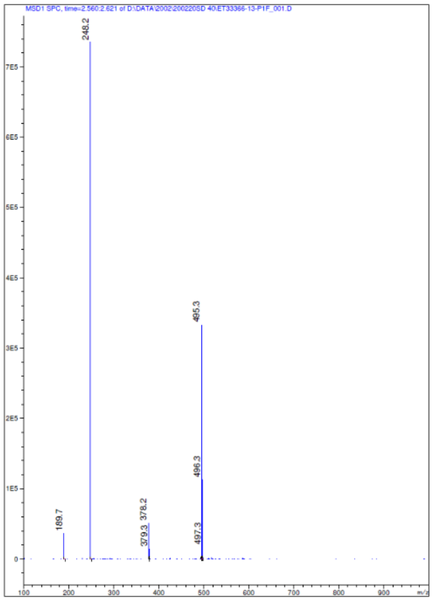

248.2
495.3
496.3
497.3
189.7
379.3
378.2

Compound 14

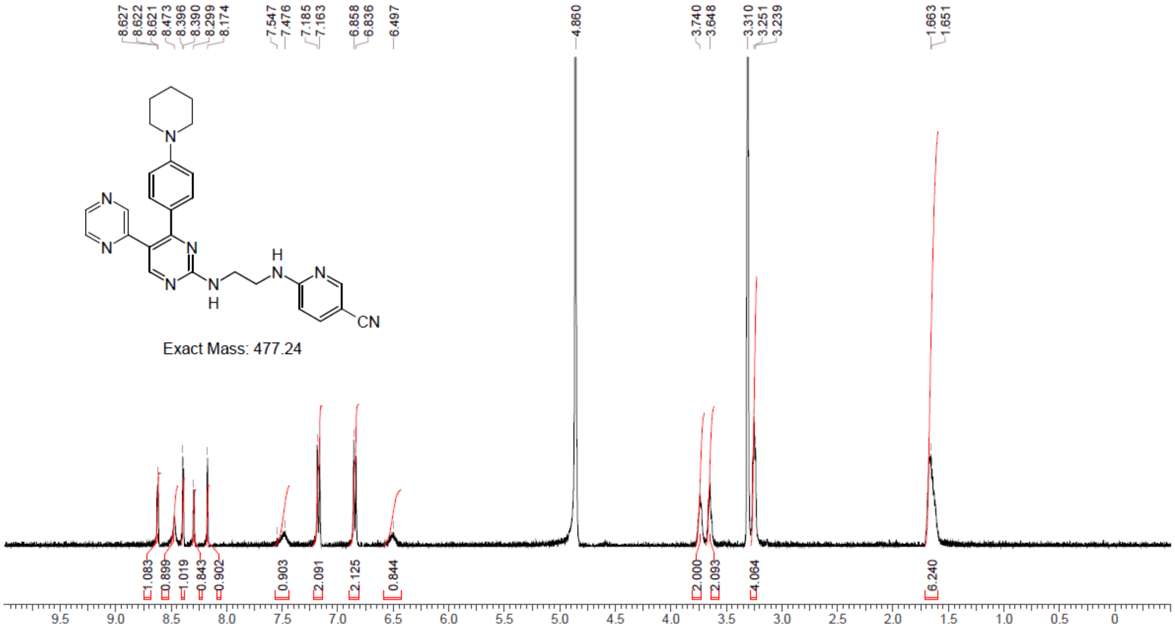

8.627
8.622
8.621
8.473
8.396
8.390
8.299
8.174
7.547
7.476
7.185
7.163
6.858
6.836
6.497
4.860
3.740
3.648
3.310
3.251
3.239
1.663
1.651
Exact Mass: 477.24
CN
1.083
0.899
1.019
0.843
0.902
0.903
2.091
2.125
0.844
2.000
2.093
4.064
6.240
9.5
9.0
8.5
8.0
7.5
7.0
6.5
6.0
5.5
5.0
4.5
4.0
3.5
3.0
2.5
2.0
1.5
1.0
0.5
0

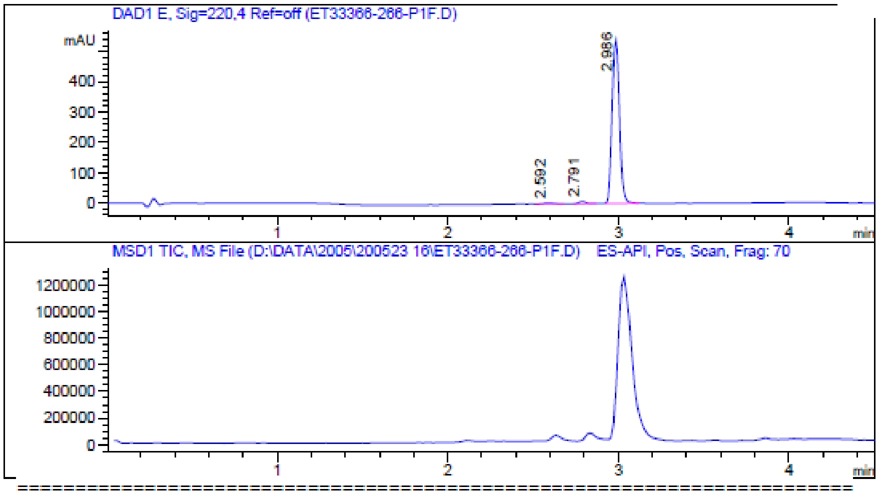

DAD1 E, Sig=220,4 Ref=off (ET33366-266-P1F.D)
mAU
2.986
2.592
2.791
min
MSD1 TIC, MS File (D:\DATA\2005\200523 16\ET33366-266-P1F.D) ES-API, Pos, Scan, Frag: 70
min

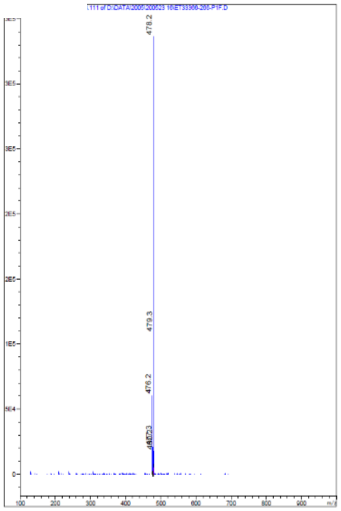

478.2
479.3
476.2

Compound 15

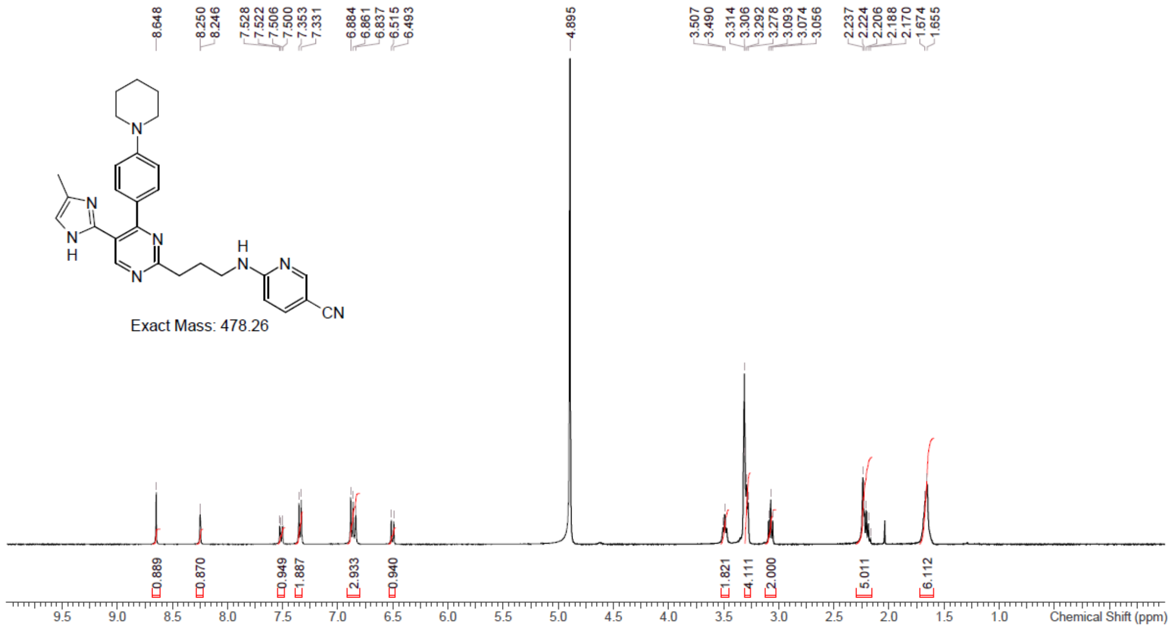

Exact Mass: 478.26
Chemical Shift (ppm)

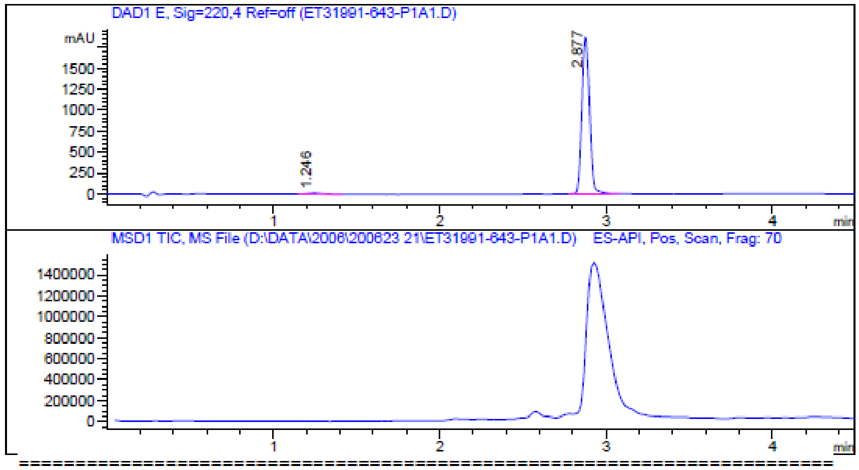

DAD1 E, Sig=220,4 Ref=off (ET31991-643-P1A1.D)
mAU
2.877
1.246
MSD1 TIC, MS File (D:\DATA\2006\200623 21\ET31991-643-P1A1.D) ES-API, Pos, Scan, Frag: 70
min

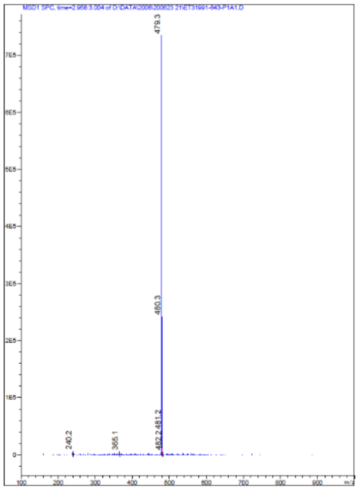

479.3
480.3
240.2
365.1

Compound 16

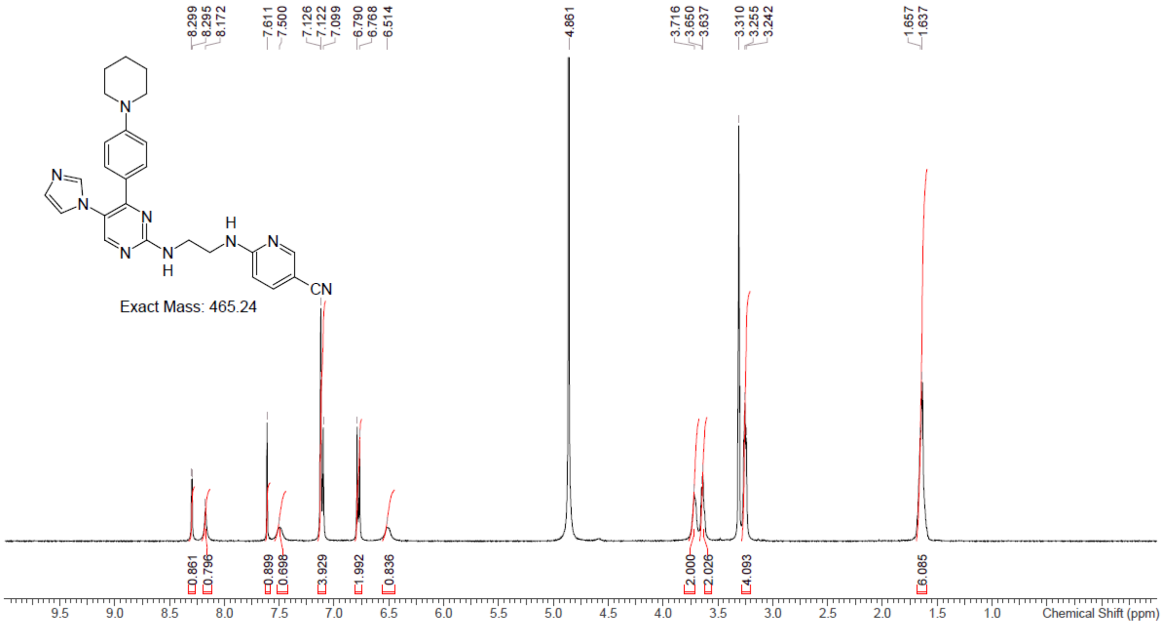

Exact Mass: 465.24
CN
Chemical Shift (ppm)

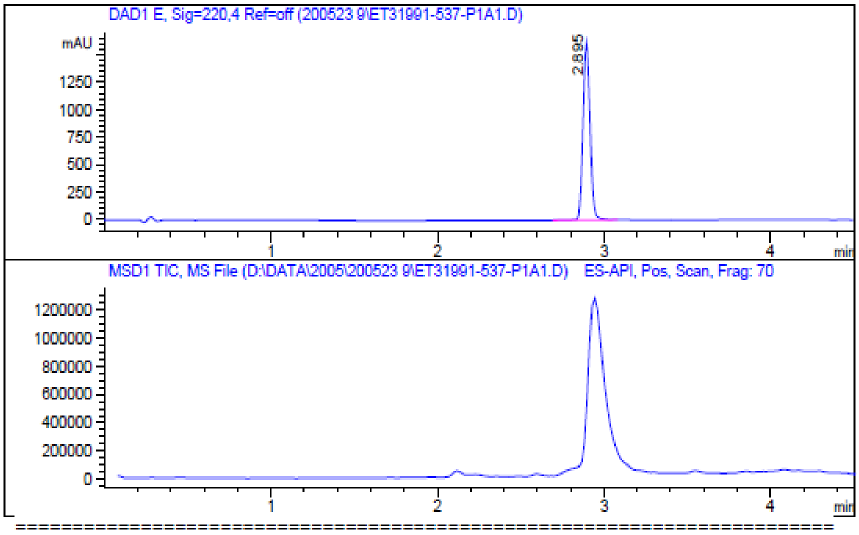

DAD1 E, Sig=220,4 Ref=off (200523 9\ET31991-537-P1A1.D)
mAU
2.895
min
MSD1 TIC, MS File (D:\DATA\2005\200523 9\ET31991-537-P1A1.D) ES-API, Pos, Scan, Frag: 70
min

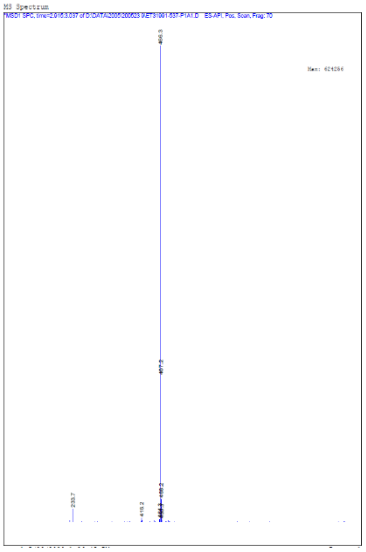

MS Spectrum

Compound 17

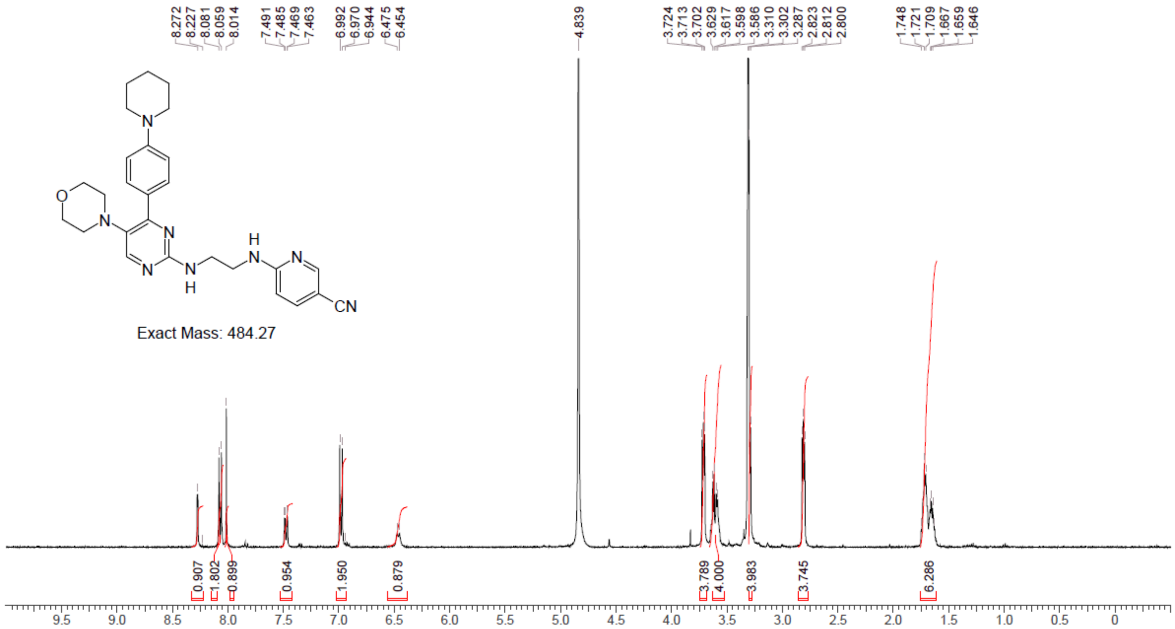

Exact Mass: 484.27

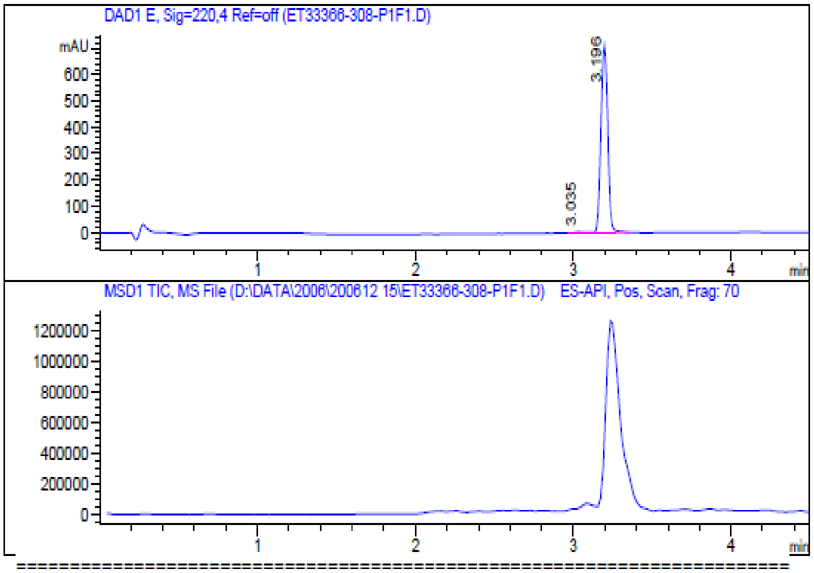

DAD1 E, Sig=220,4 Ref=off (ET33366-308-P1F1.D)
3.196
3.035
MSD1 TIC, MS File (D:\DATA\2006\200612 15\ET33366-308-P1F1.D) ES-API, Pos, Scan, Frag: 70

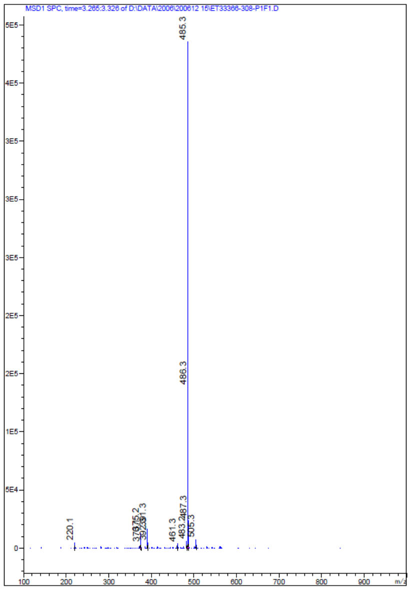

485.3
486.3
220.1

Compound 18

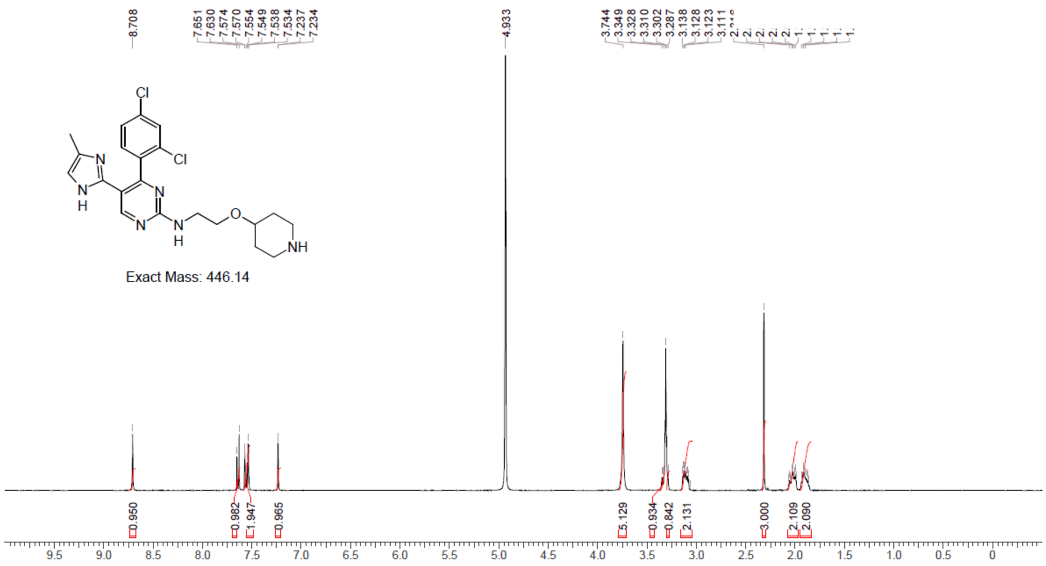

Exact Mass: 446.14

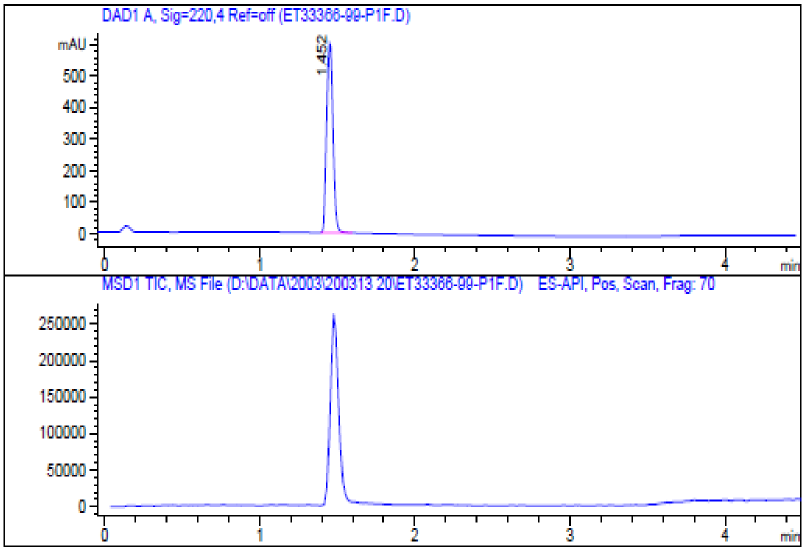

DAD1 A, Sig=220,4 Ref=off (ET33366-99-P1F.D)
mAU
1.452
MSD1 TIC, MS File (D:\DATA\2003\200313 20\ET33366-99-P1F.D) ES-API, Pos, Scan, Frag: 70
min

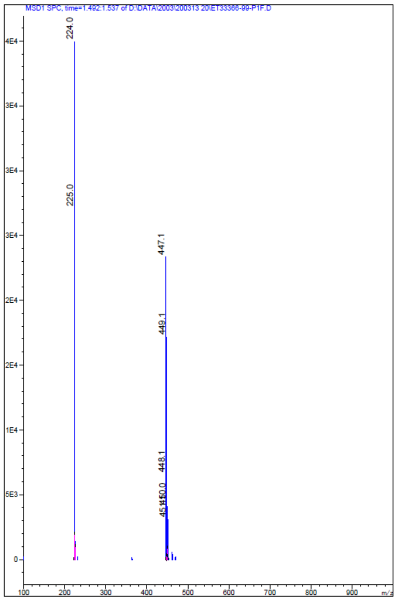

224.0
225.0
447.1
449.1
448.1

==

Compound 22

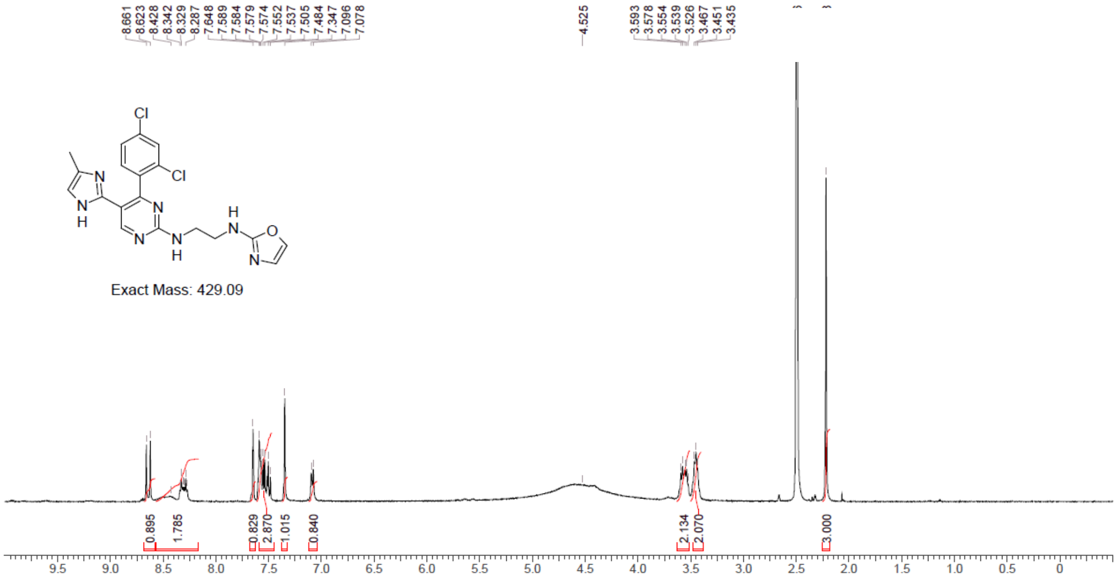
Exact Mass: 429.09

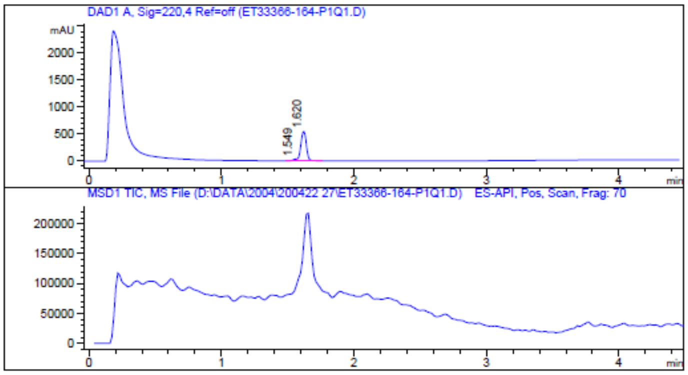
DAD1 A, Sig=220,4 Ref=off (ET33366-164-P1Q1.D)
MSD1 TIC, MS File (D:\DATA\2004\200422 27\ET33366-164-P1Q1.D) ES-API, Pos, Scan, Frag: 70

===

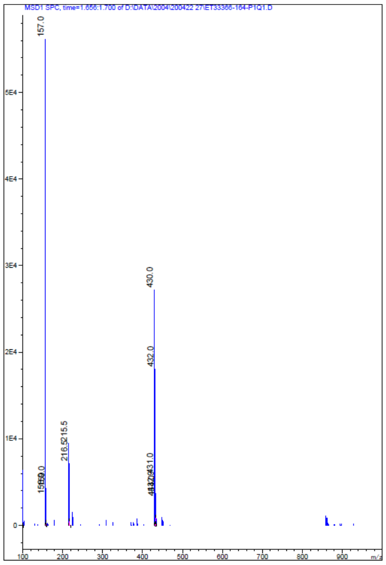

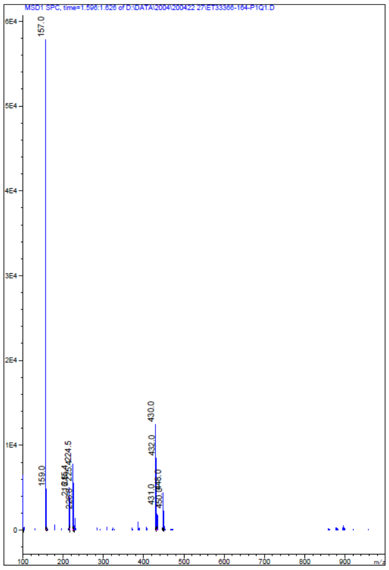

Compound 25

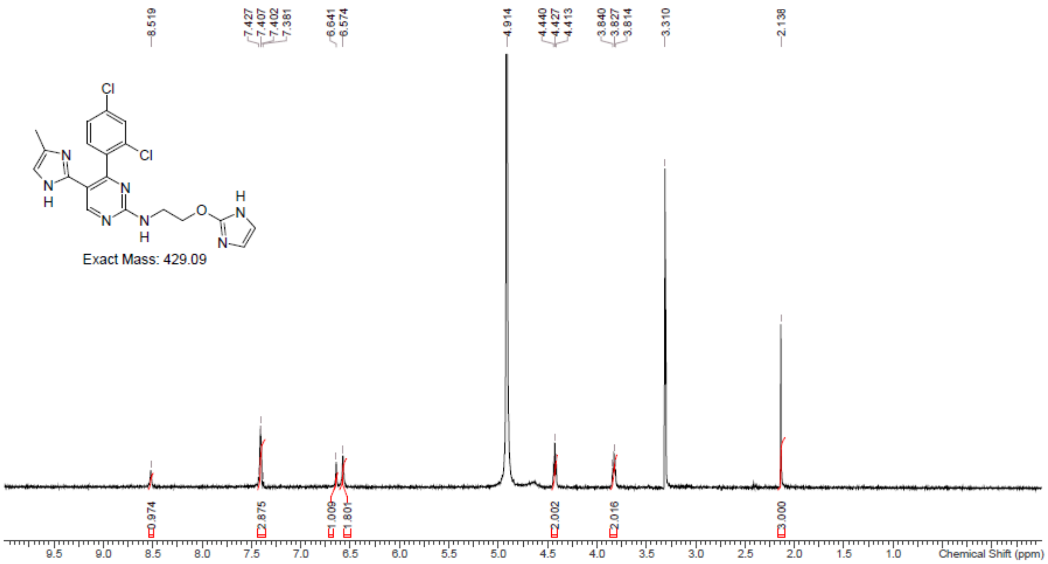

8.519
7.427
7.407
7.402
7.381
6.641
6.574
4.914
4.440
4.427
4.413
3.840
3.827
3.814
3.310
2.138
Cl
Cl
N
N
N
H
N
N
H
O
H
N
N
Exact Mass: 429.09
0.974
2.875
1.009
1.801
2.002
2.016
3.000
Chemical Shift (ppm)

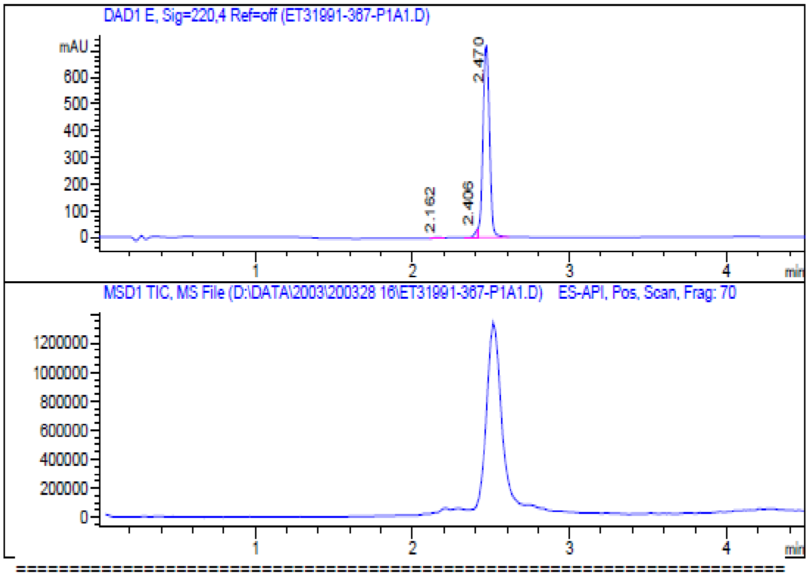

DAD1 E, Sig=220,4 Ref=off (ET31991-367-P1A1.D)
mAU
2.470
2.162
2.406
min
MSD1 TIC, MS File (D:\DATA\2003\200328 16\ET31991-367-P1A1.D) ES-API, Pos, Scan, Frag: 70
min

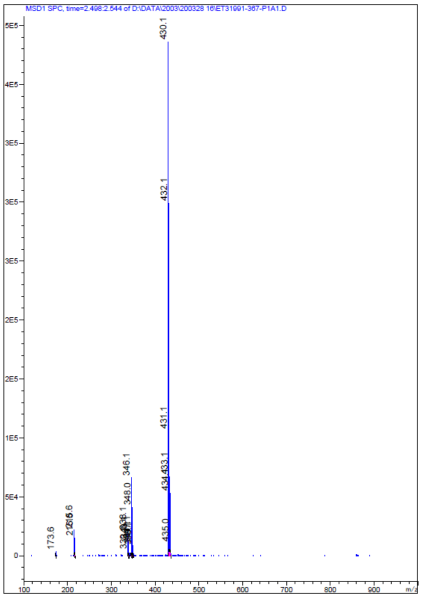

430.1
432.1
431.1
433.1
346.1
348.0
173.6

Compound 26

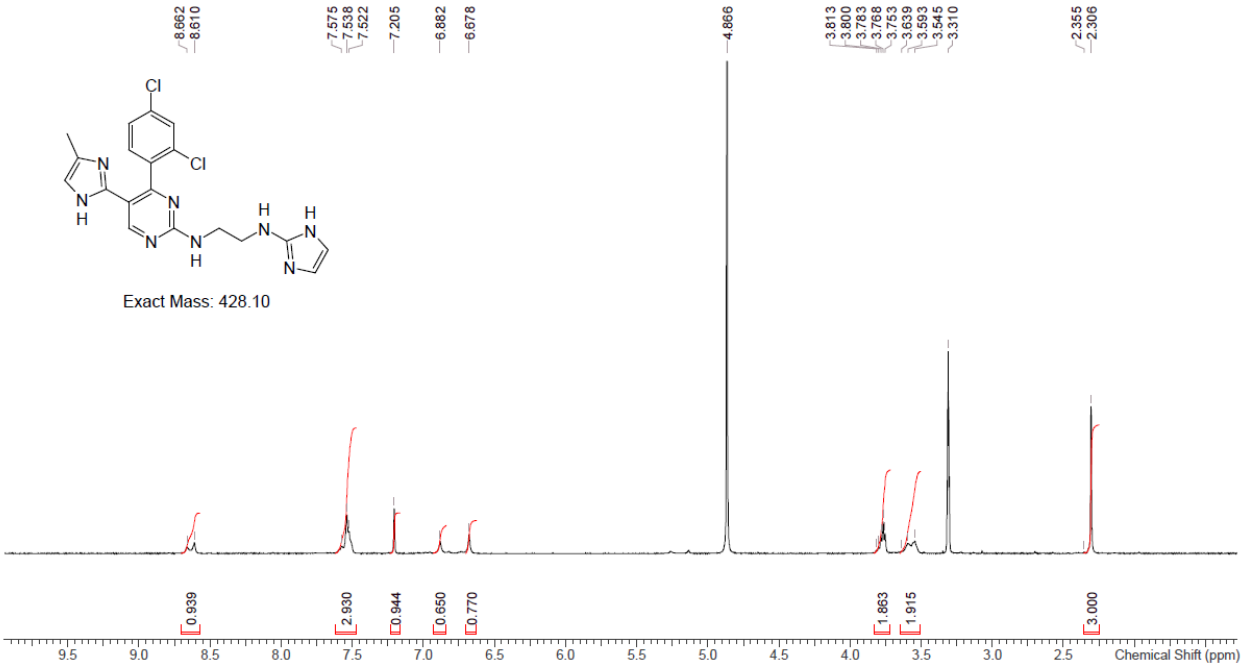

Exact Mass: 428.10
8.662
8.610
7.575
7.538
7.522
7.205
6.882
6.678
4.866
3.813
3.800
3.783
3.768
3.753
3.639
3.593
3.545
3.310
2.355
2.306
0.939
2.930
0.944
0.650
0.770
1.863
1.915
3.000
Chemical Shift (ppm)

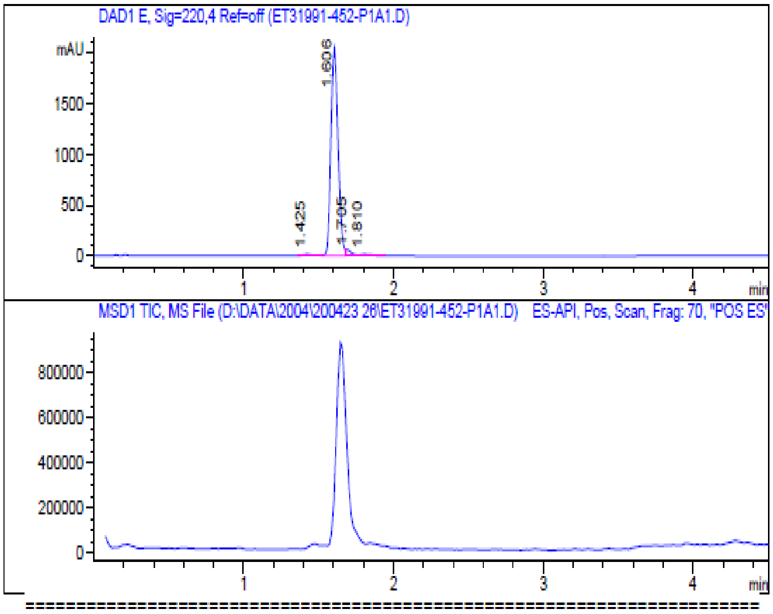

DAD1 E, Sig=220,4 Ref=off (ET31991-452-P1A1.D)
mAU
1.606
1.425
1.705
1.810
MSD1 TIC, MS File (D:\DATA\2004\200423 26\ET31991-452-P1A1.D) ES-API, Pos, Scan, Frag: 70, "POS ES"
min

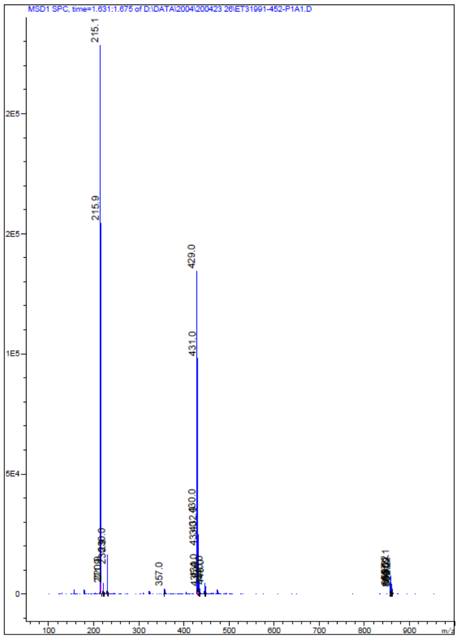

215.1
215.9
429.0
431.0
357.0

Compound 27

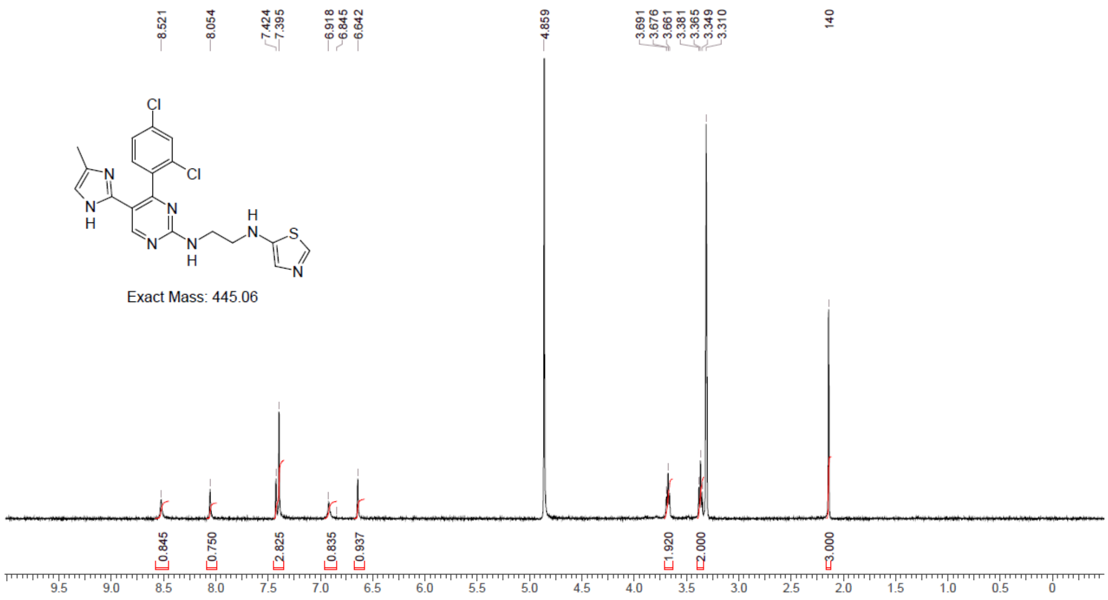

Exact Mass: 445.06

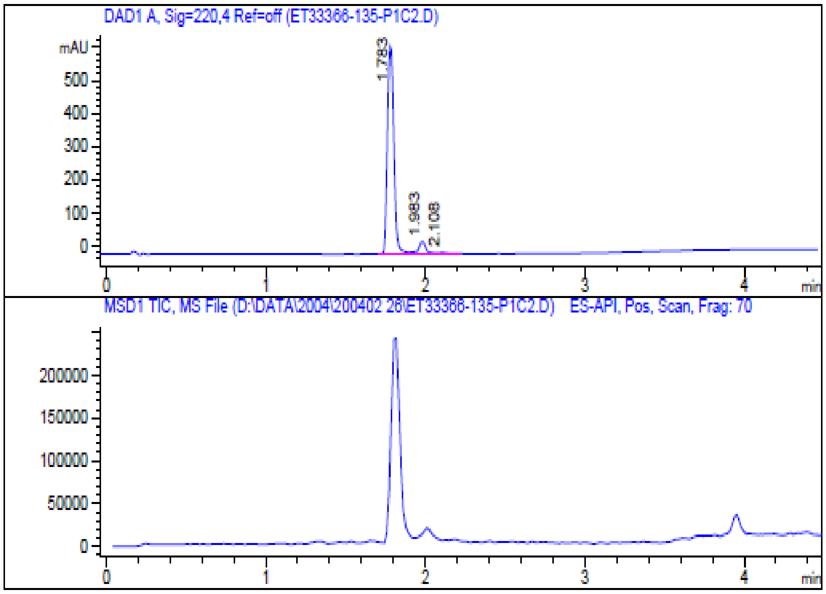

DAD1 A, Sig=220,4 Ref=off (ET33366-135-P1C2.D)
MSD1 TIC, MS File (D:\DATA\2004\200402 26\ET33366-135-P1C2.D) ES-API, Pos, Scan, Frag: 70

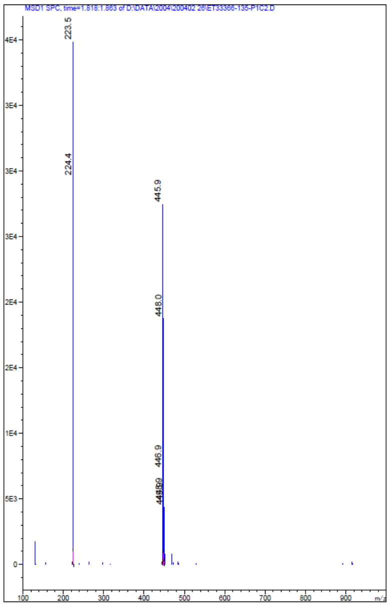

223.5
224.4
445.9
448.0
446.9

Compound 29

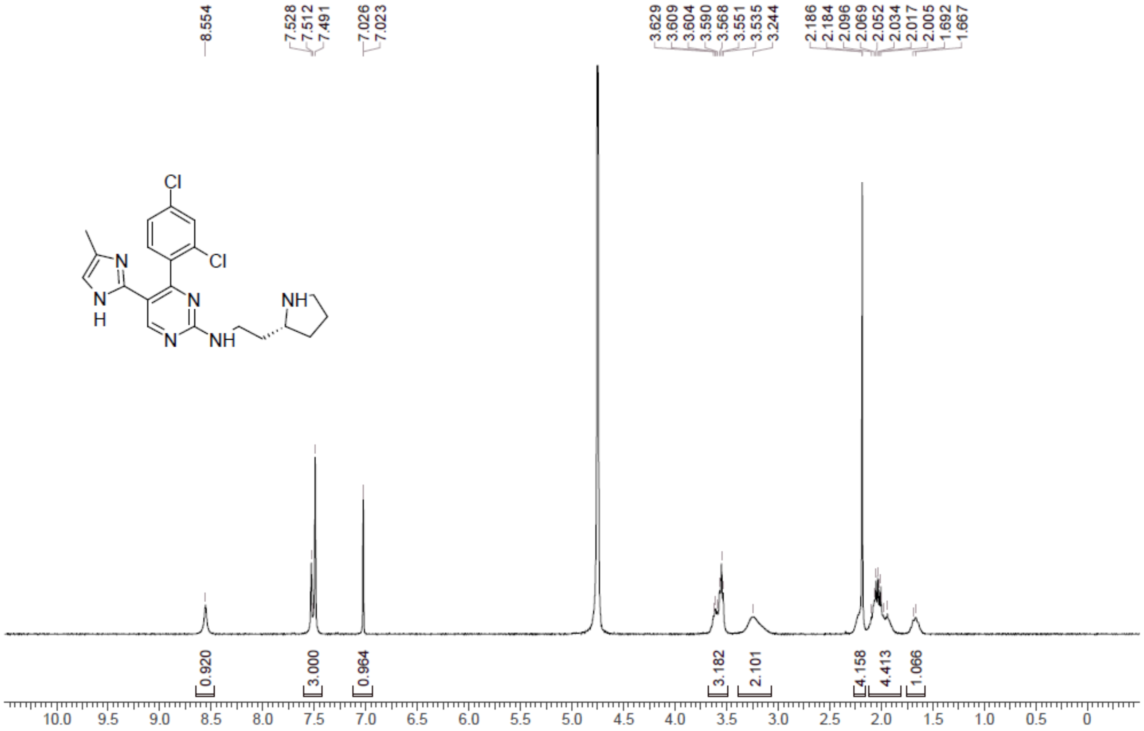
8.554
7.528
7.512
7.491
7.026
7.023
3.629
3.609
3.604
3.590
3.568
3.551
3.535
3.244
2.186
2.184
2.096
2.069
2.052
2.034
2.017
2.005
1.692
1.667
Cl
Cl
N
N
H
N
N
NH
NH
0.920
3.000
0.964
3.182
2.101
4.158
4.413
1.066
10.0
9.5
9.0
8.5
8.0
7.5
7.0
6.5
6.0
5.5
5.0
4.5
4.0
3.5
3.0
2.5
2.0
1.5
1.0
0.5
0

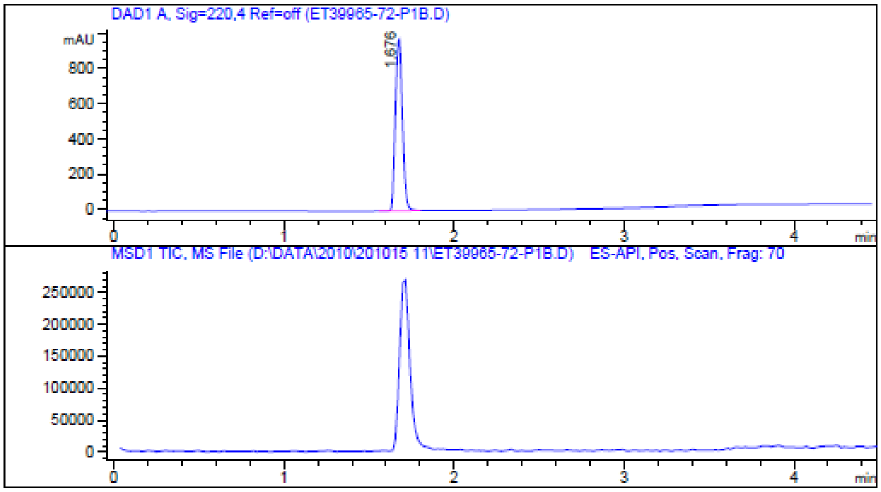
DAD1 A, Sig=220,4 Ref=off (ET39965-72-P1B.D)
mAU
800
600
400
200
0
1.676
0
1
2
3
4
min
MSD1 TIC, MS File (D:\DATA\2010\201015 11\ET39965-72-P1B.D) ES-API, Pos, Scan, Frag: 70
250000
200000
150000
100000
50000
0
0
1
2
3
4
min

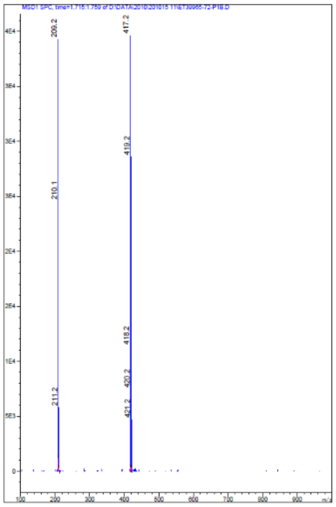
209.2
210.1
211.2
417.2
419.2
418.2
420.2
421.2

Compound 30

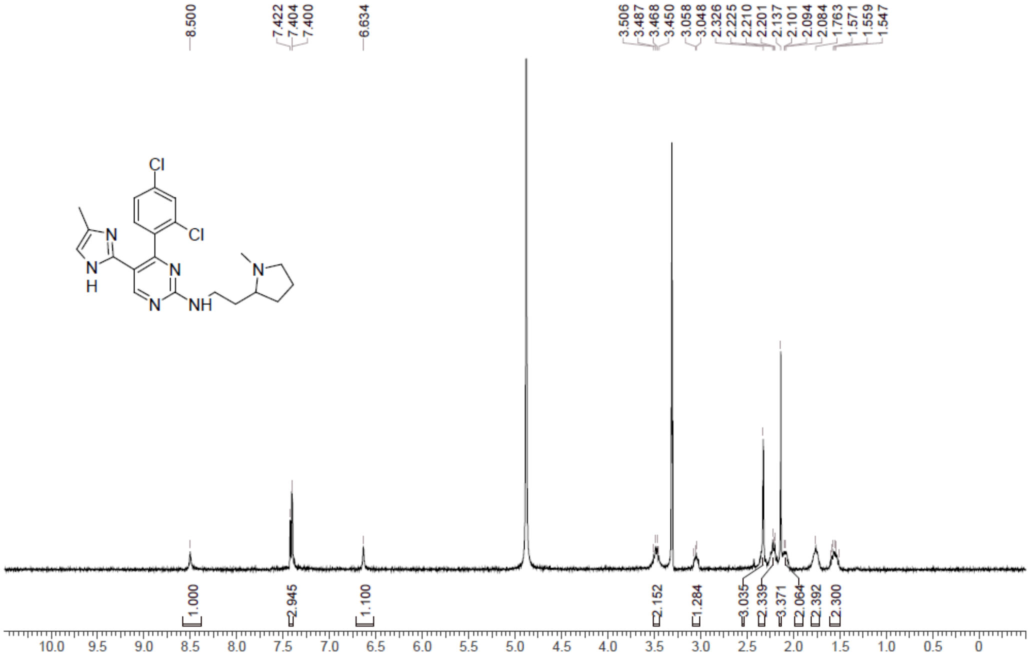

8.500
7.422
7.404
7.400
6.634
3.506
3.487
3.468
3.450
3.058
3.048
2.326
2.225
2.210
2.201
2.137
2.101
2.094
2.084
1.763
1.571
1.559
1.547
Cl
Cl
N
N
H
N
N
NH
N
1.000
2.945
1.100
2.152
1.284
3.035
2.339
3.371
2.064
2.392
2.300
10.0 9.5 9.0 8.5 8.0 7.5 7.0 6.5 6.0 5.5 5.0 4.5 4.0 3.5 3.0 2.5 2.0 1.5 1.0 0.5 0

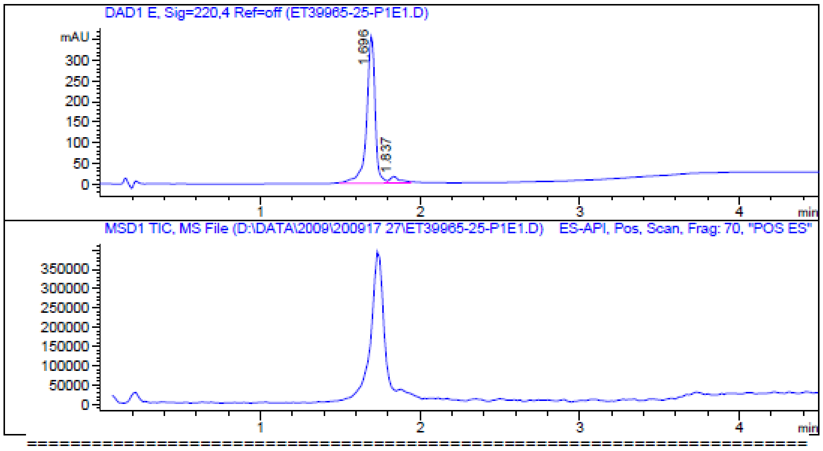

DAD1 E, Sig=220,4 Ref=off (ET39965-25-P1E1.D)
mAU
300
250
200
150
100
50
0
1.696
1.837
1 2 3 4 min
MSD1 TIC, MS File (D:\DATA\2009\200917 27\ET39965-25-P1E1.D) ES-API, Pos, Scan, Frag: 70, "POS ES"
350000
300000
250000
200000
150000
100000
50000
0
1 2 3 4 min

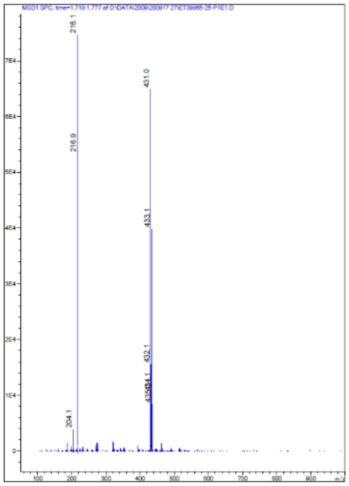

218.1
216.9
204.1
431.0
433.1
432.1

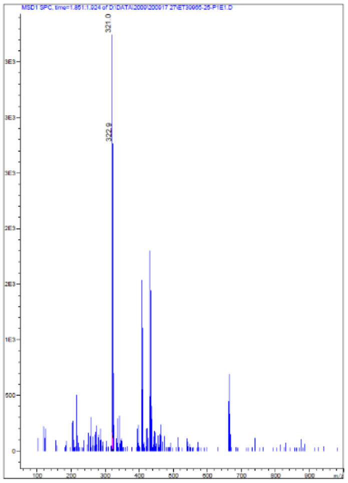

321.9
322.9

Compound 31

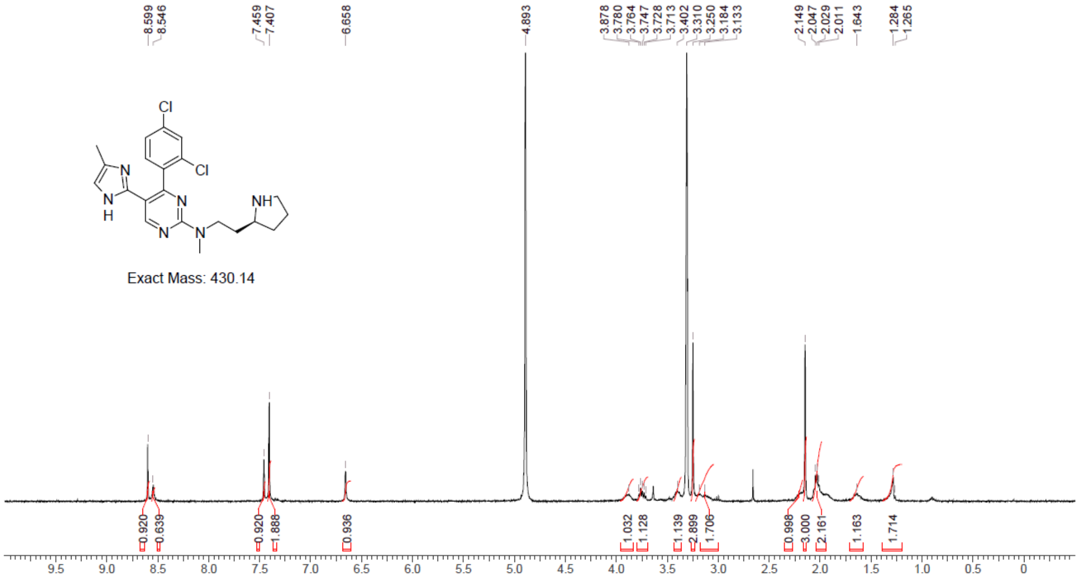

Cl
Cl
N
N
H
N
N
NH
N
Exact Mass: 430.14

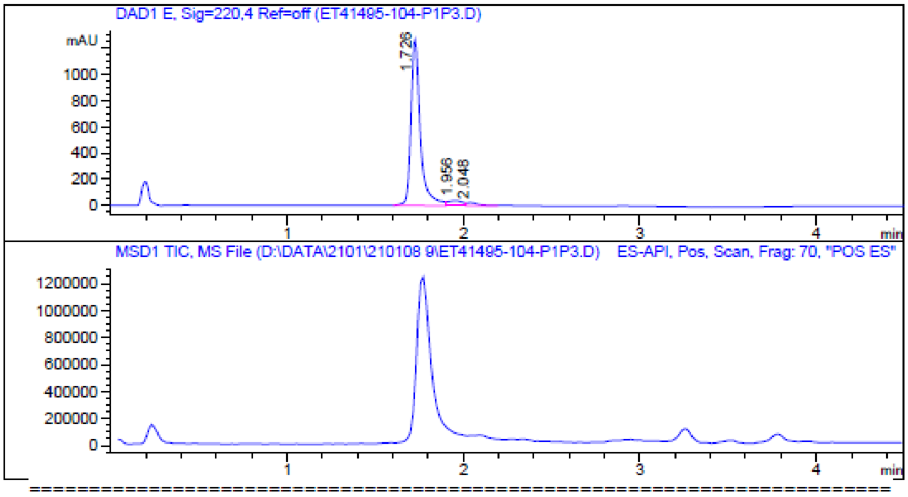

DAD1 E, Sig=220,4 Ref=off (ET41495-104-P1P3.D)
mAU
1.726
1.956
2.048
min
MSD1 TIC, MS File (D:\DATA\2101\210108 9\ET41495-104-P1P3.D) ES-API, Pos, Scan, Frag: 70, "POS ES"
min

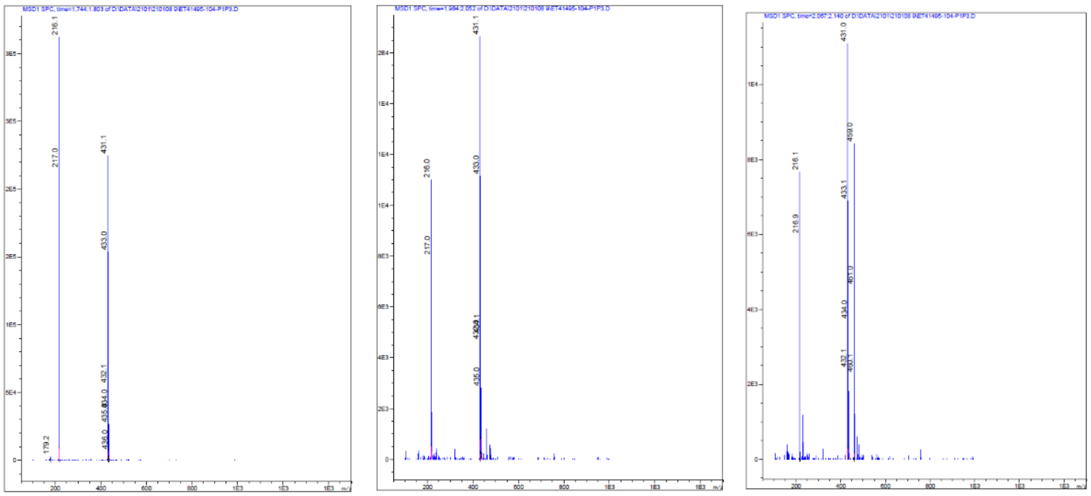

Compound 32

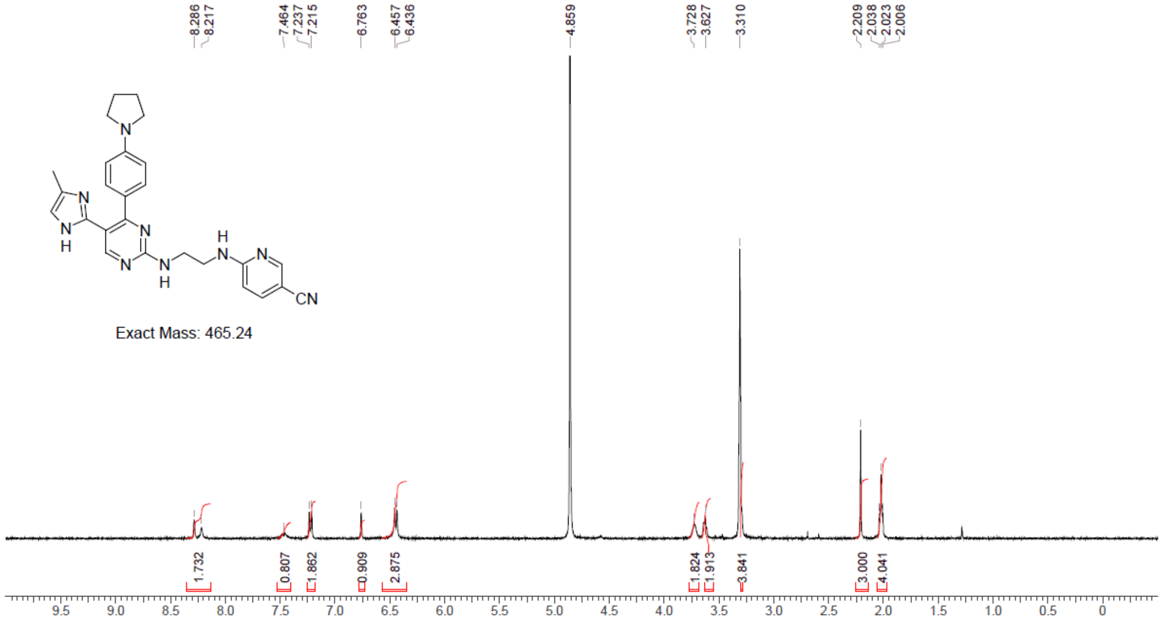

Exact Mass: 465.24

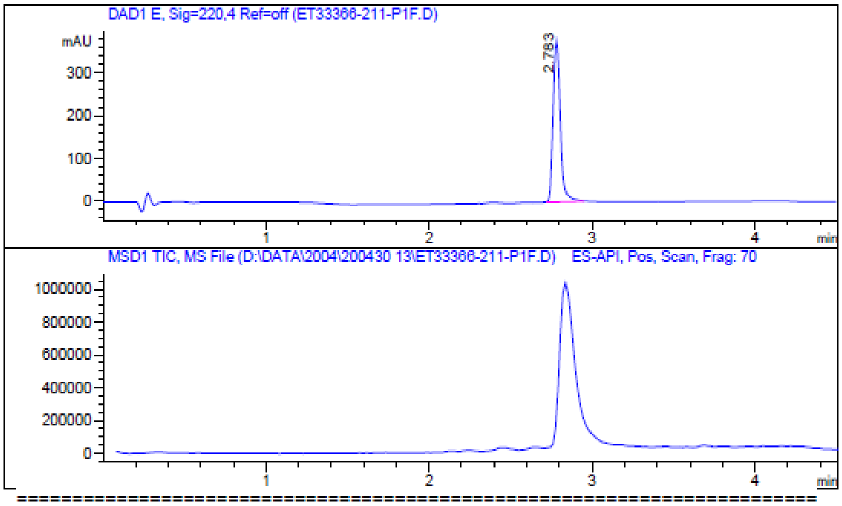

DAD1 E, Sig=220,4 Ref=off (ET33366-211-P1F.D)
mAU
2.783
MSD1 TIC, MS File (D:\DATA\2004\200430 13\ET33366-211-P1F.D) ES-API, Pos, Scan, Frag: 70
min

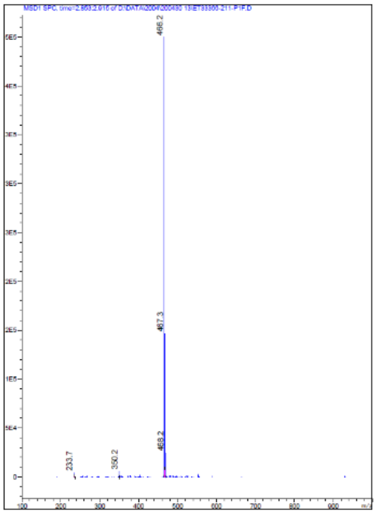

466.2
467.3
468.2
233.7
350.2

Compound 33

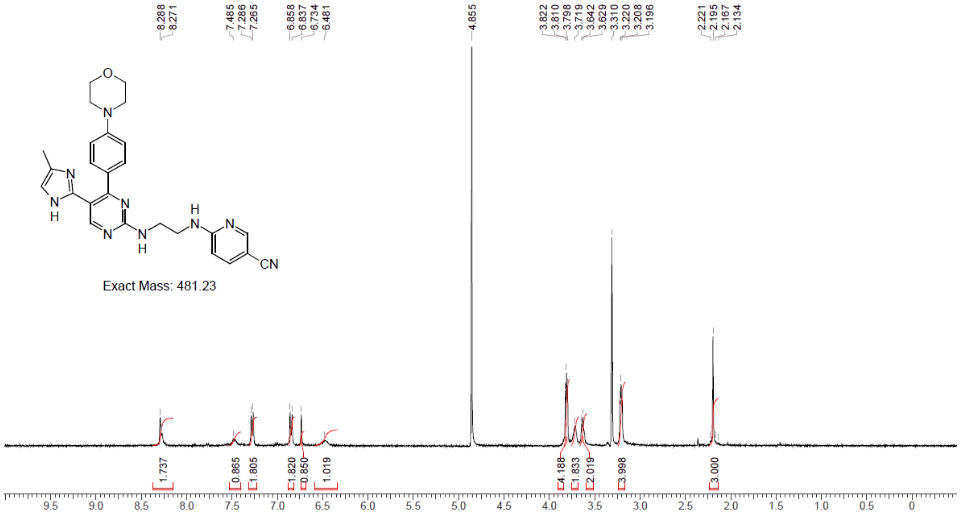

Exact Mass: 481.23

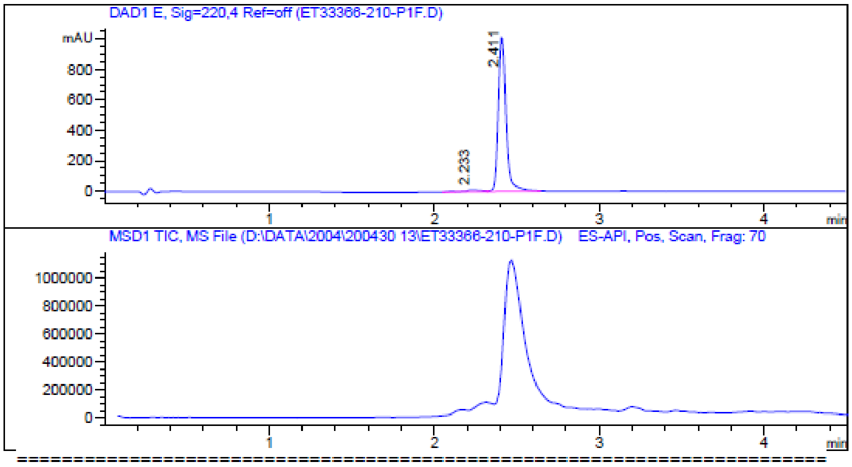

DAD1 E, Sig=220,4 Ref=off (ET33366-210-P1F.D)
MSD1 TIC, MS File (D:\DATA\2004\200430 13\ET33366-210-P1F.D) ES-API, Pos, Scan, Frag: 70

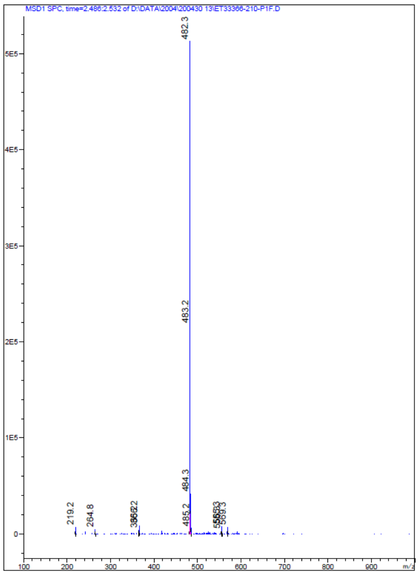

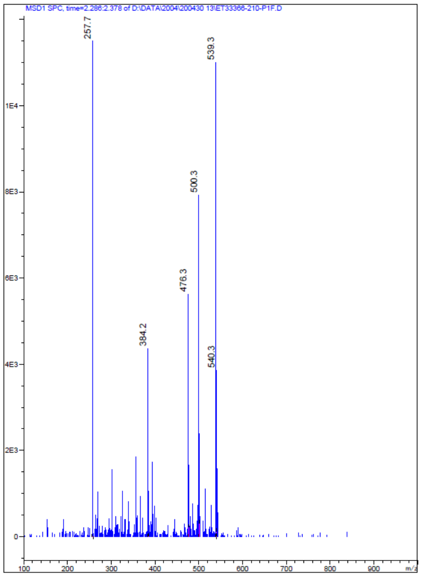

Compound 34

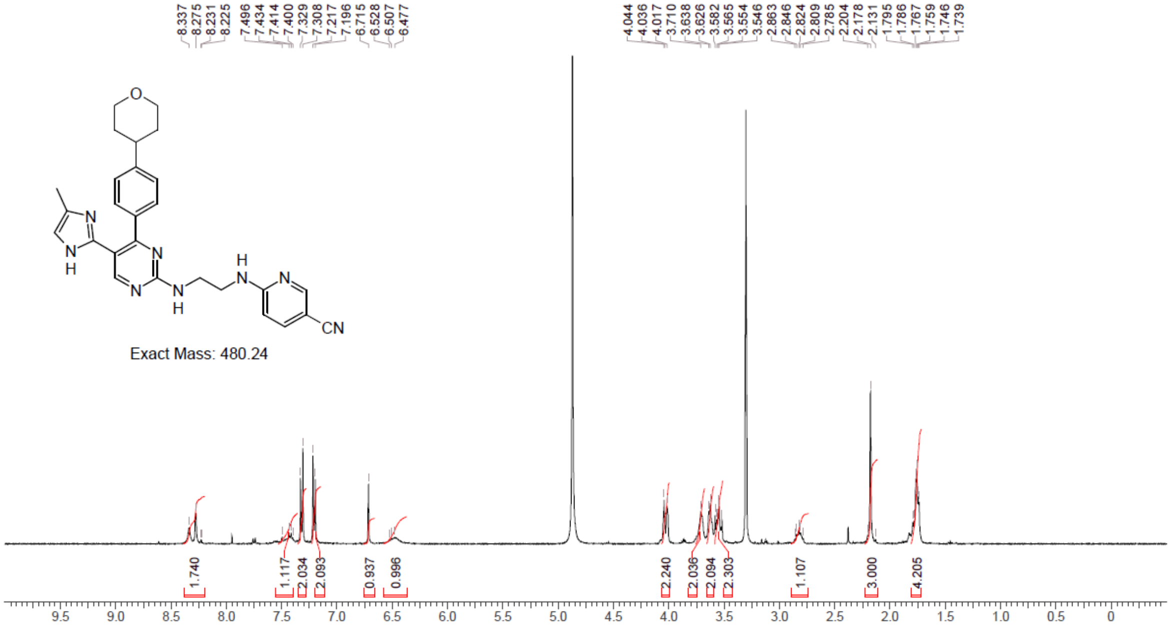

Exact Mass: 480.24

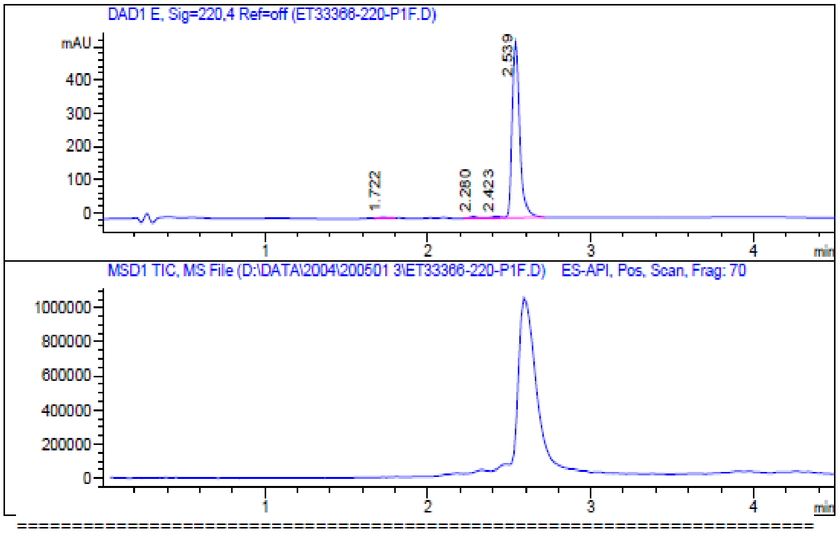

DAD1 E, Sig=220,4 Ref=off (ET33366-220-P1F.D)
mAU
2.539
1.722
2.280
2.423
min
MSD1 TIC, MS File (D:\DATA\2004\200501 3\ET33366-220-P1F.D) ES-API, Pos, Scan, Frag: 70
min

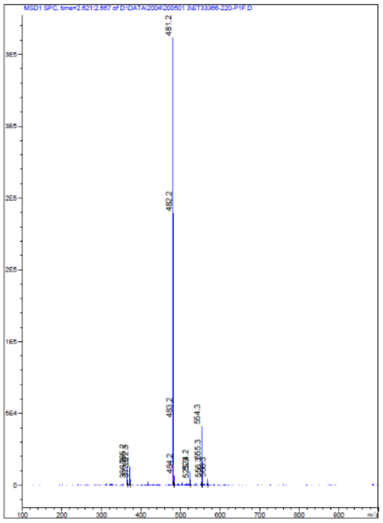

481.2
482.2
483.2
554.3

Compound 35

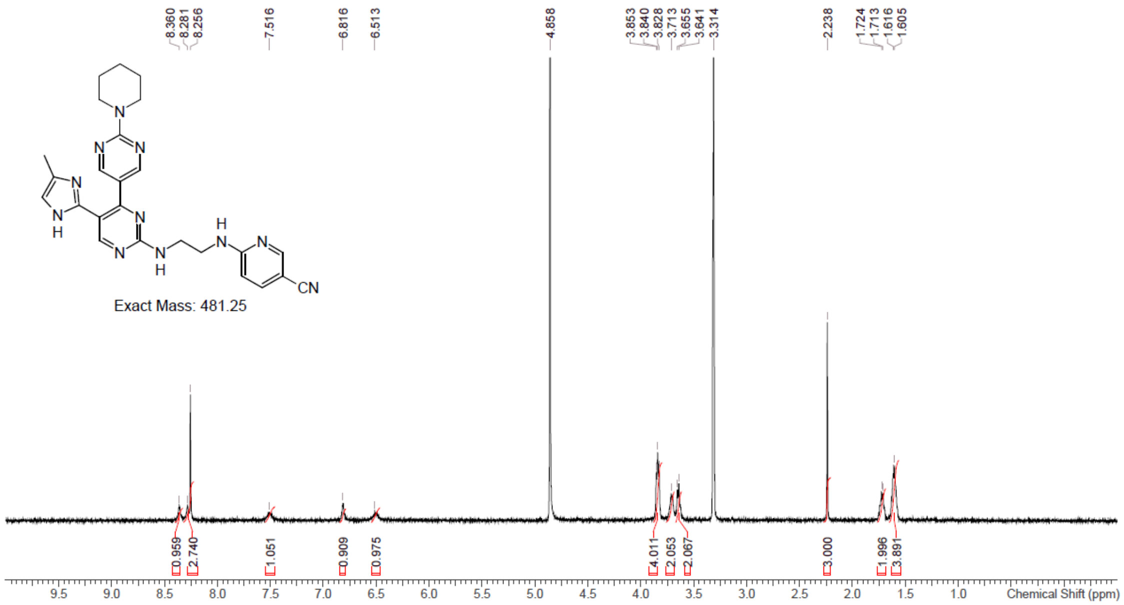

8.360
8.281
8.256
7.516
6.816
6.513
4.858
3.853
3.840
3.828
3.713
3.655
3.641
3.314
2.238
1.724
1.713
1.616
1.605
CN
Exact Mass: 481.25
0.959
2.740
1.051
0.909
0.975
4.011
2.053
2.067
3.000
1.996
3.891
9.5 9.0 8.5 8.0 7.5 7.0 6.5 6.0 5.5 5.0 4.5 4.0 3.5 3.0 2.5 2.0 1.5 1.0
Chemical Shift (ppm)

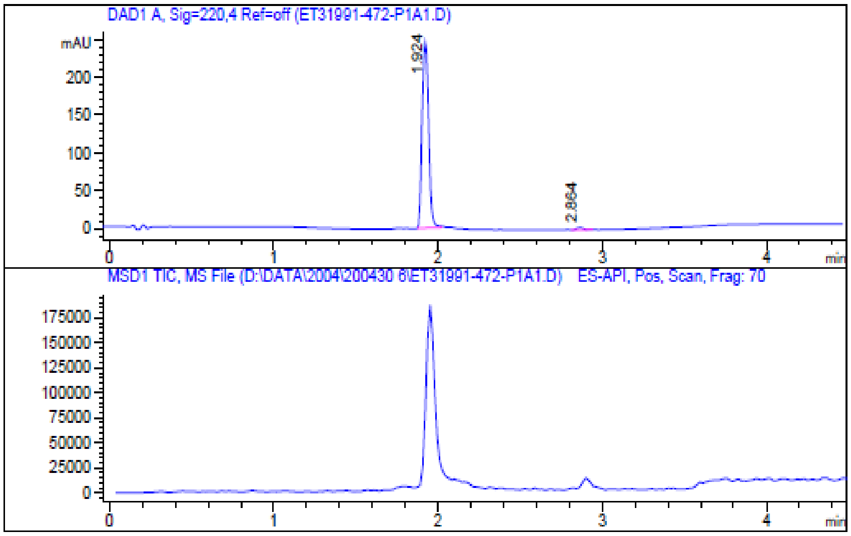

DAD1 A, Sig=220,4 Ref=off (ET31991-472-P1A1.D)
mAU
1.924
2.864
min
MSD1 TIC, MS File (D:\DATA\2004\200430 6\ET31991-472-P1A1.D) ES-API, Pos, Scan, Frag: 70
min

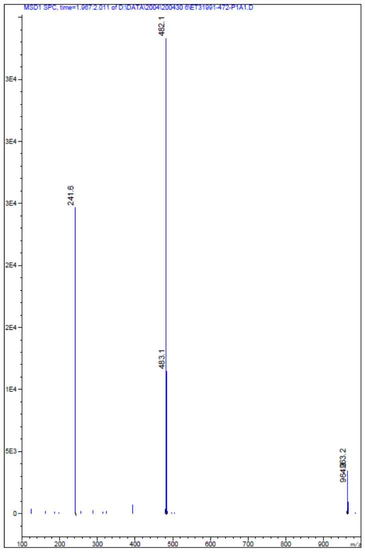

482.1
241.6
483.1
963.2

Compound 36

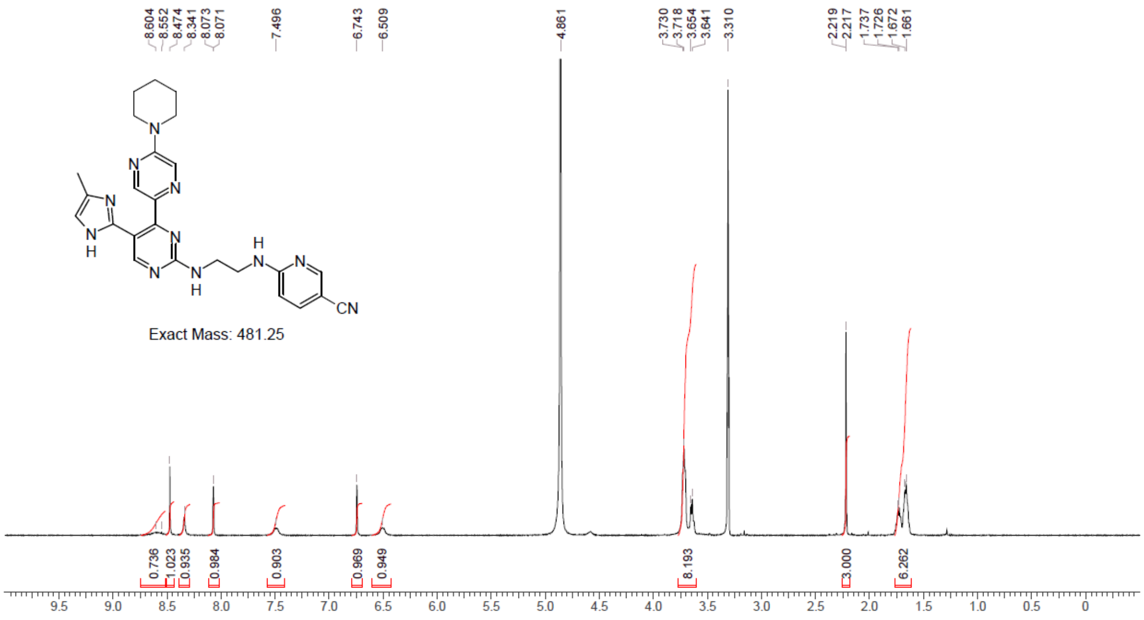
Exact Mass: 481.25

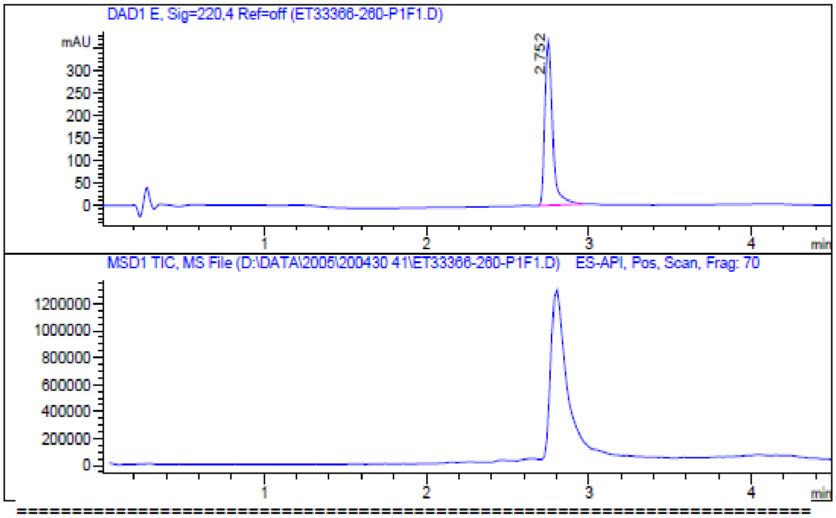
DAD1 E, Sig=220,4 Ref=off (ET33366-260-P1F1.D)
mAU
2.752
MSD1 TIC, MS File (D:\DATA\2005\200430 41\ET33366-260-P1F1.D) ES-API, Pos, Scan, Frag: 70
min

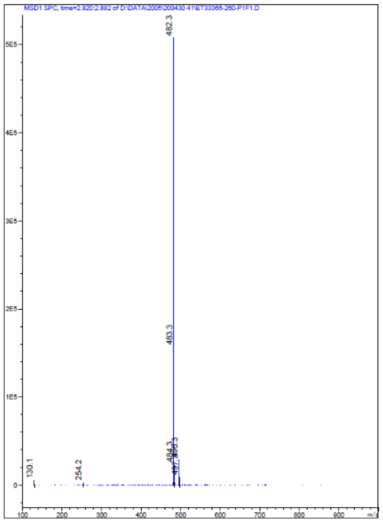
482.3
483.3
130.1
254.2

Compound 37

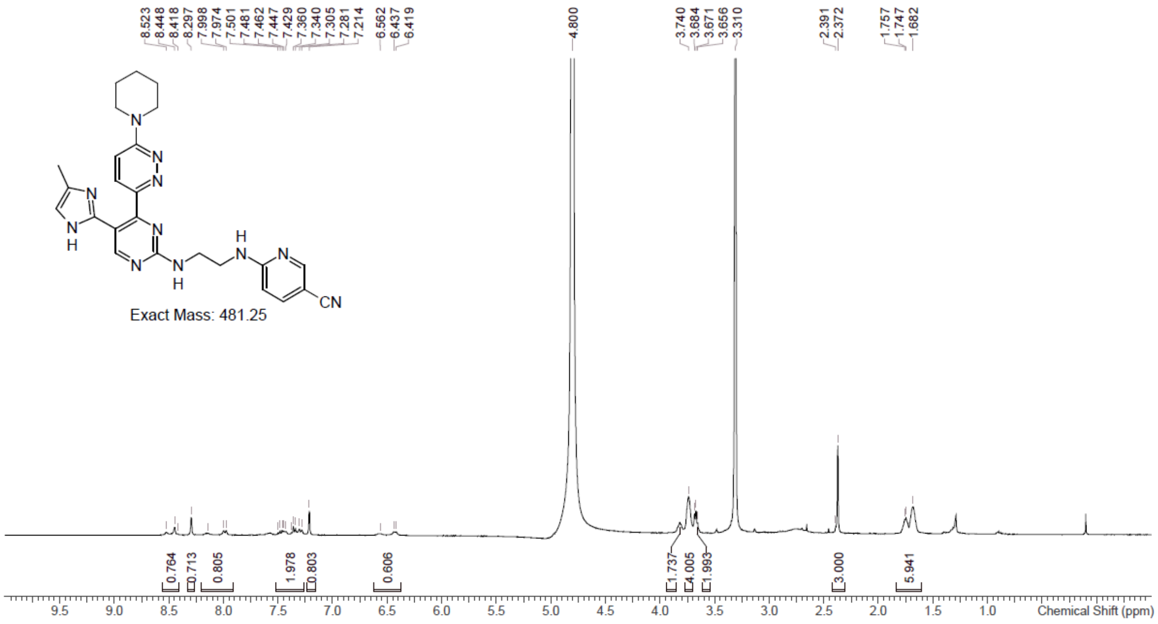
Exact Mass: 481.25
Chemical Shift (ppm)

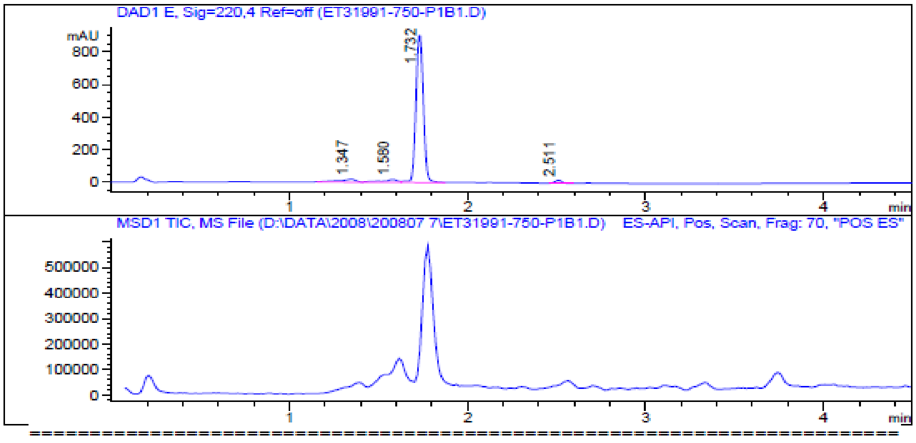
DAD1 E, Sig=220,4 Ref=off (ET31991-750-P1B1.D)
mAU
1.732
1.347
1.580
2.511
min
MSD1 TIC, MS File (D:\DATA\2008\200807 7\ET31991-750-P1B1.D) ES-API, Pos, Scan, Frag: 70, "POS ES"
min

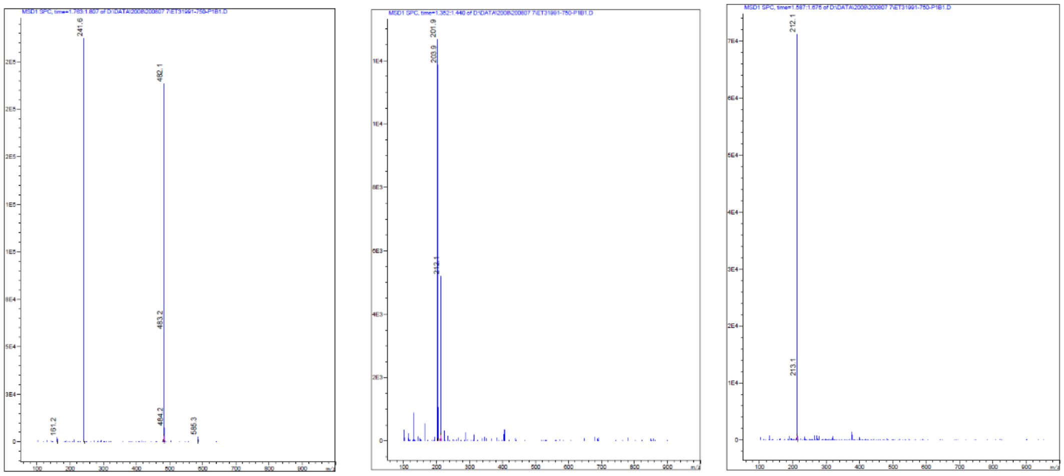

Compound 38

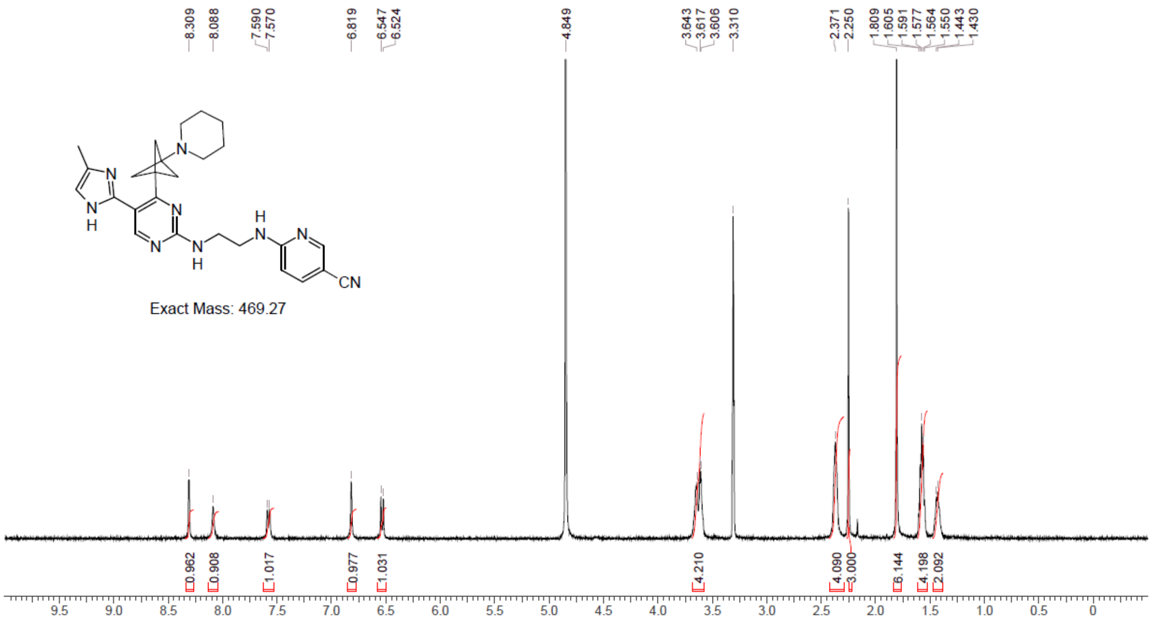
Exact Mass: 469.27
8.309
8.088
7.590
7.570
6.819
6.547
6.524
4.849
3.643
3.617
3.606
3.310
2.371
2.250
1.809
1.605
1.591
1.577
1.564
1.550
1.443
1.430
0.962
0.908
1.017
0.977
1.031
4.210
4.090
3.000
6.144
4.198
2.092

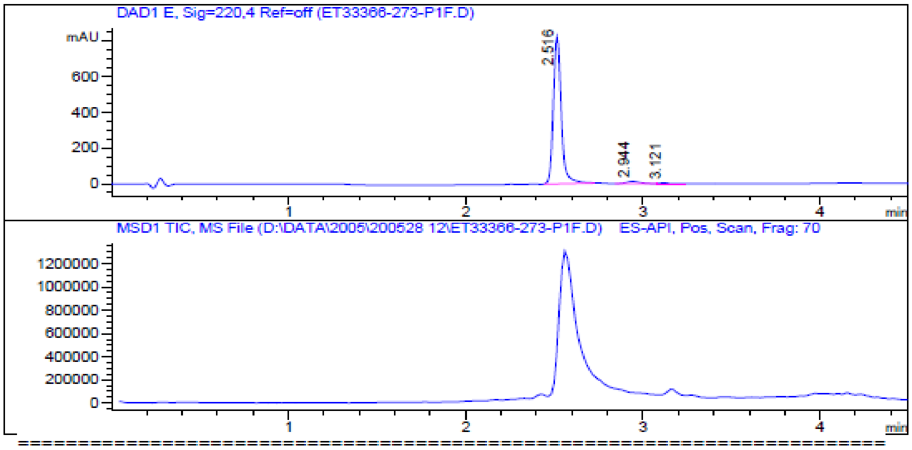
DAD1 E, Sig=220,4 Ref=off (ET33366-273-P1F.D)
mAU
2.516
2.944
3.121
min
MSD1 TIC, MS File (D:\DATA\2005\200528 12\ET33366-273-P1F.D) ES-API, Pos, Scan, Frag: 70
min

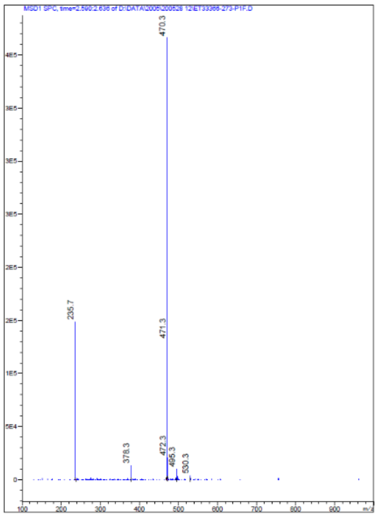
470.3
235.7
471.3
378.3
472.3
495.3
530.3

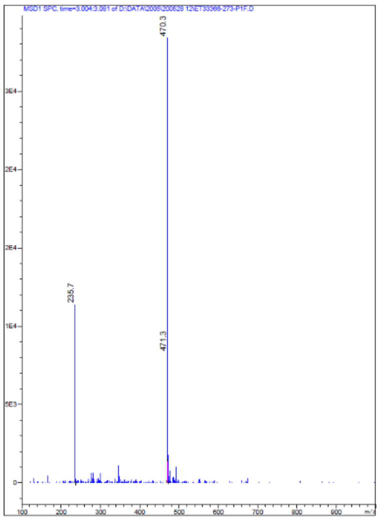
470.3
235.7
471.3

Compound 39

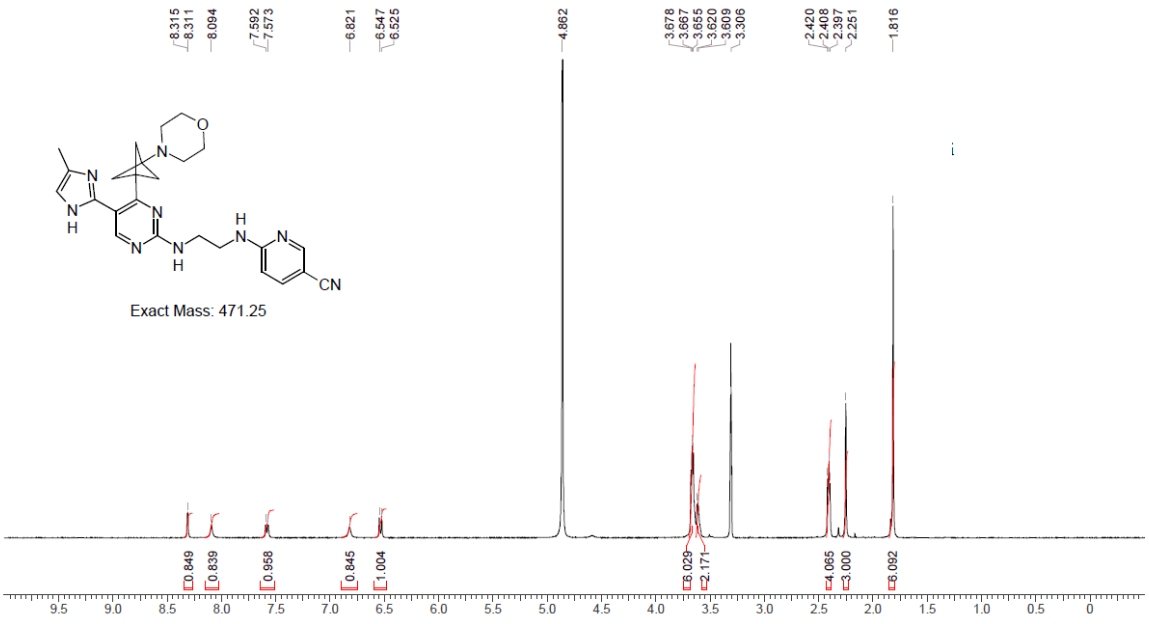
Exact Mass: 471.25

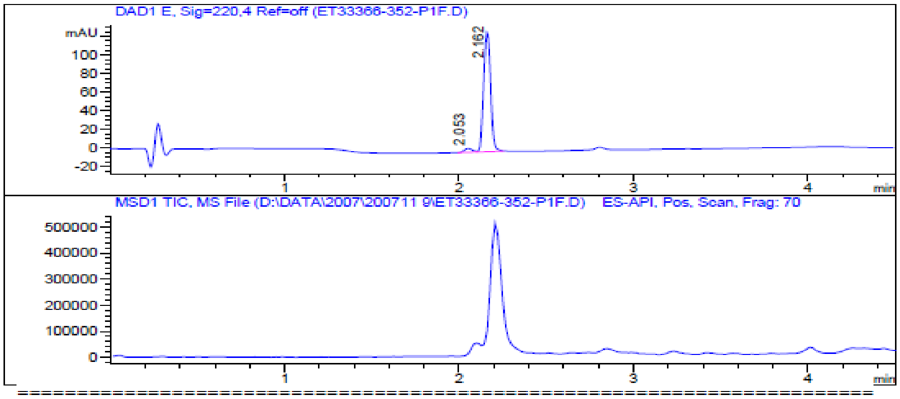
DAD1 E, Sig=220,4 Ref=off (ET33366-352-P1F.D)
MSD1 TIC, MS File (D:\DATA\2007\200711 9\ET33366-352-P1F.D) ES-API, Pos, Scan, Frag: 70

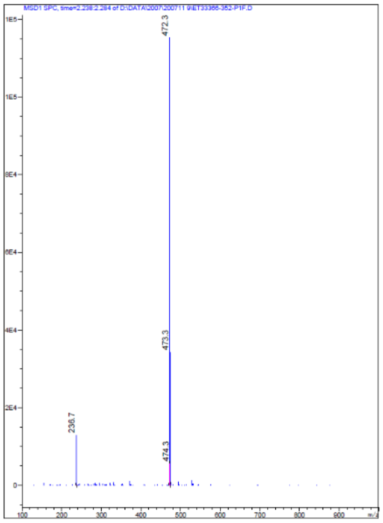

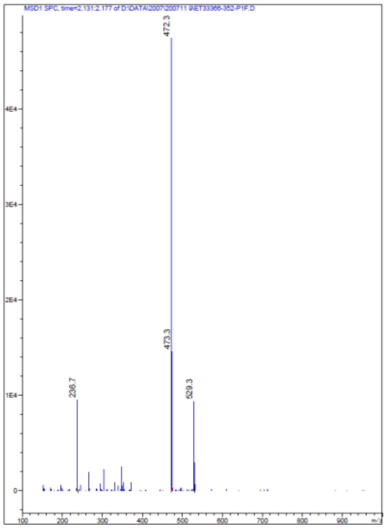

Compound 40

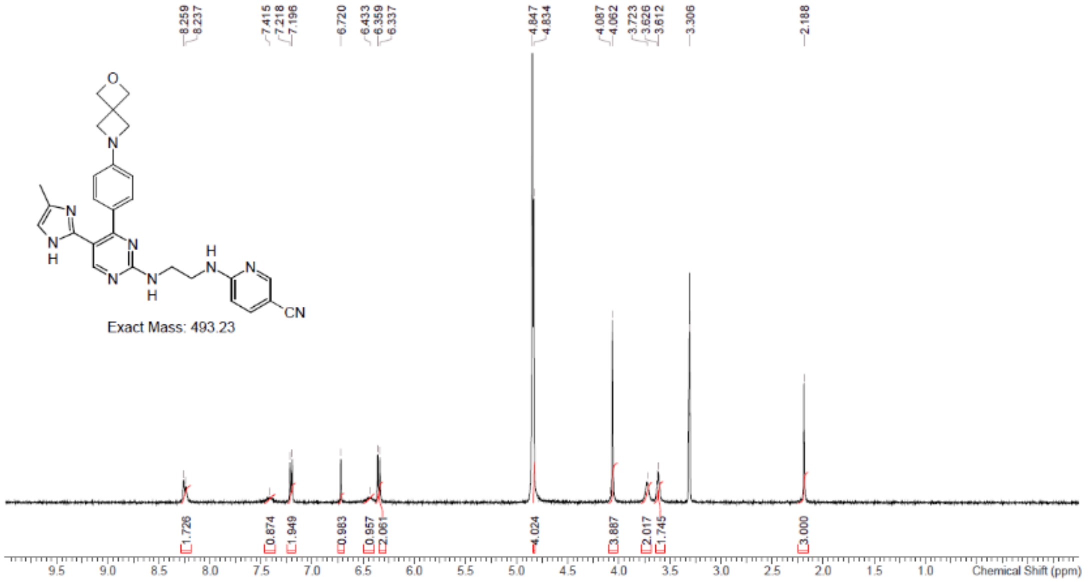

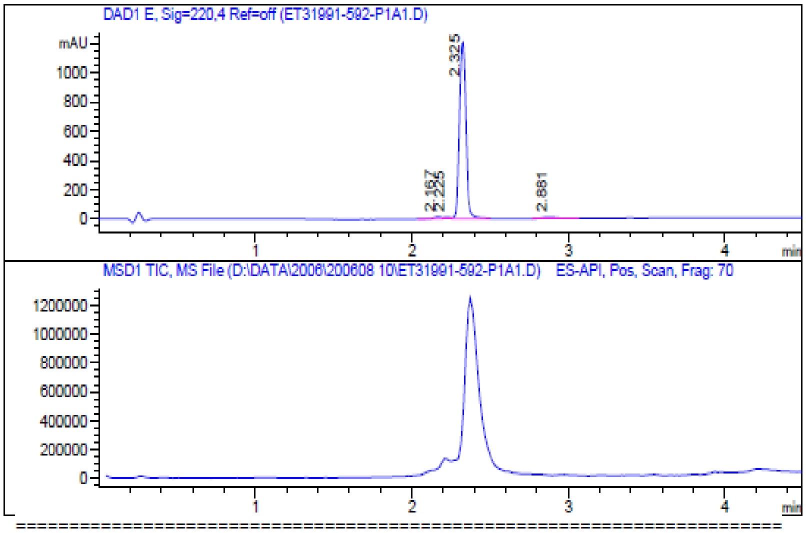

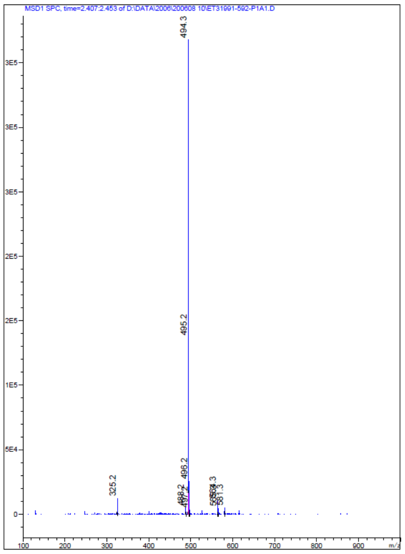

Compound 41

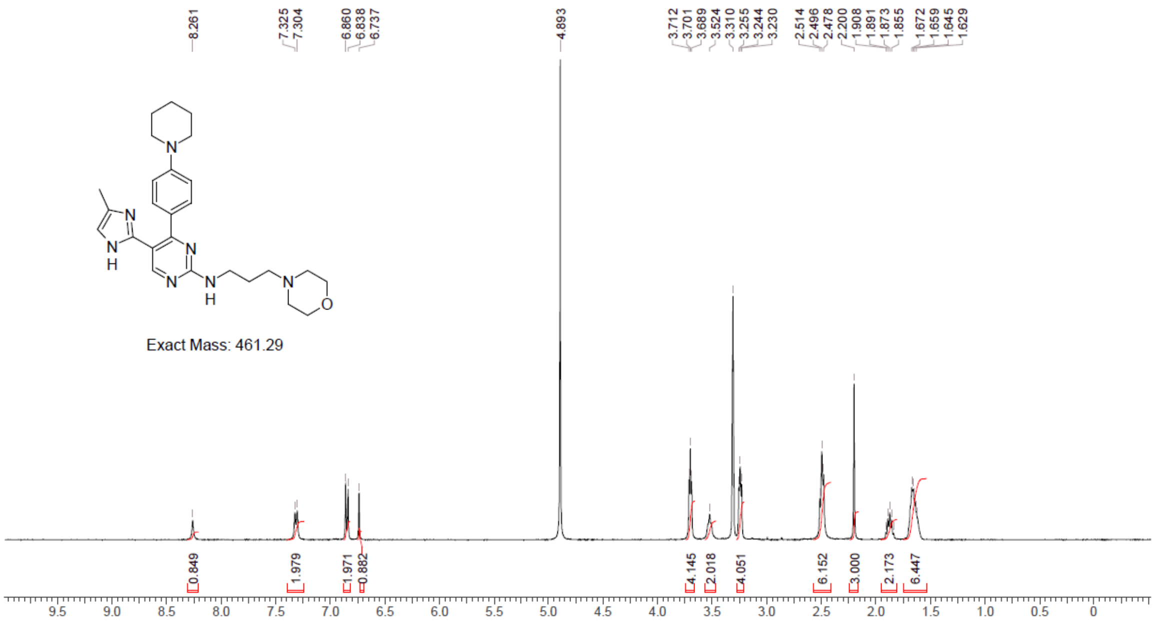

Exact Mass: 461.29
8.261
7.325
7.304
6.860
6.838
6.737
4.893
3.712
3.701
3.689
3.524
3.310
3.255
3.244
3.230
2.514
2.496
2.478
2.200
1.908
1.891
1.873
1.855
1.672
1.659
1.645
1.629
0.849
1.979
1.971
0.882
4.145
2.018
4.051
6.152
3.000
2.173
6.447

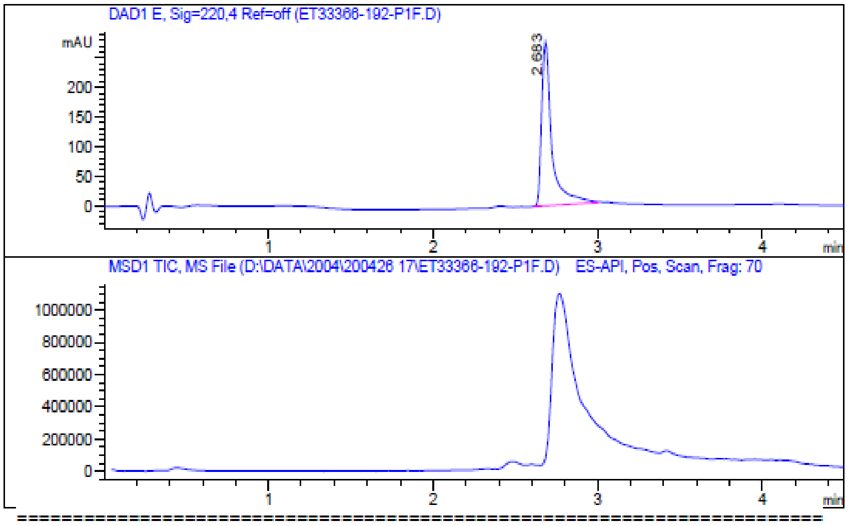

DAD1 E, Sig=220,4 Ref=off (ET33366-192-P1F.D)
mAU
2.683
MSD1 TIC, MS File (D:\DATA\2004\200426 17\ET33366-192-P1F.D) ES-API, Pos, Scan, Frag: 70
min

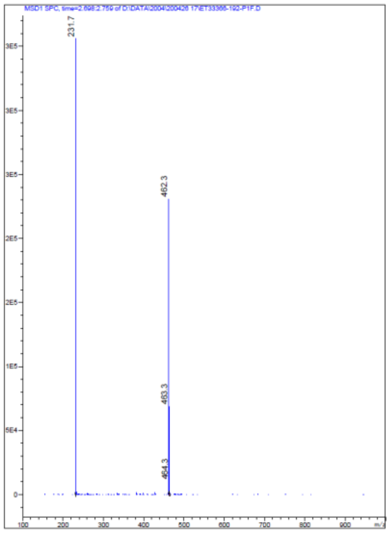

231.7
462.3
463.3
464.3

Compound 42

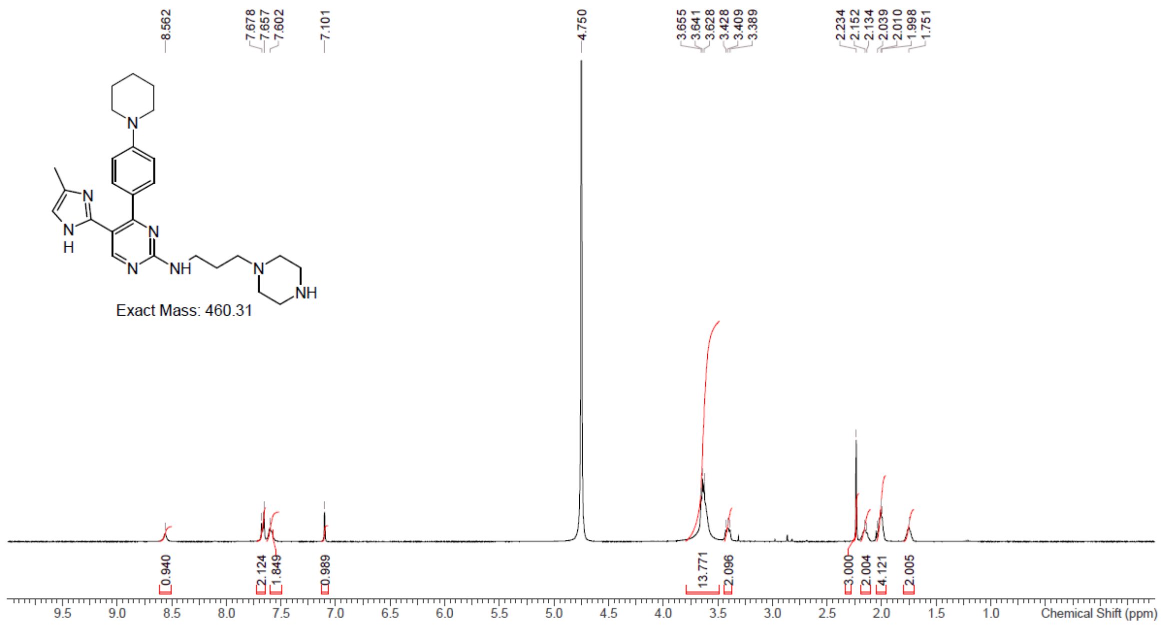

Exact Mass: 460.31
Chemical Shift (ppm)

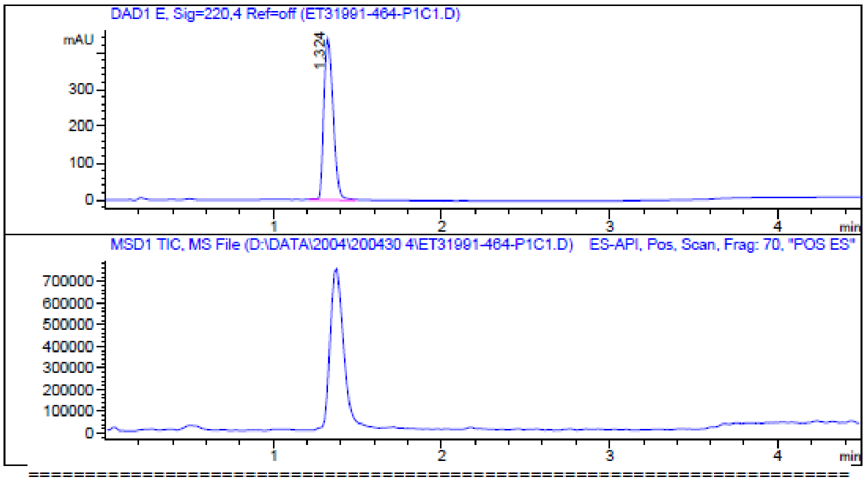

DAD1 E, Sig=220,4 Ref=off (ET31991-464-P1C1.D)
mAU
1.324
MSD1 TIC, MS File (D:\DATA\2004\200430 4\ET31991-464-P1C1.D) ES-API, Pos, Scan, Frag: 70, "POS ES"

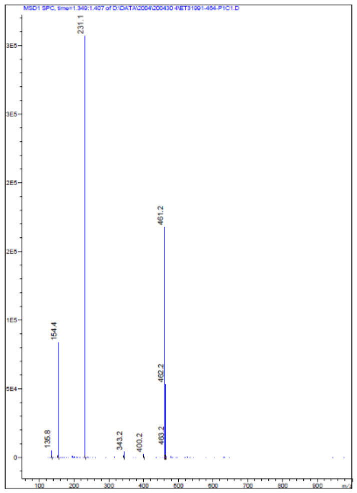

231.1
461.2
154.4
462.2

Compound 43

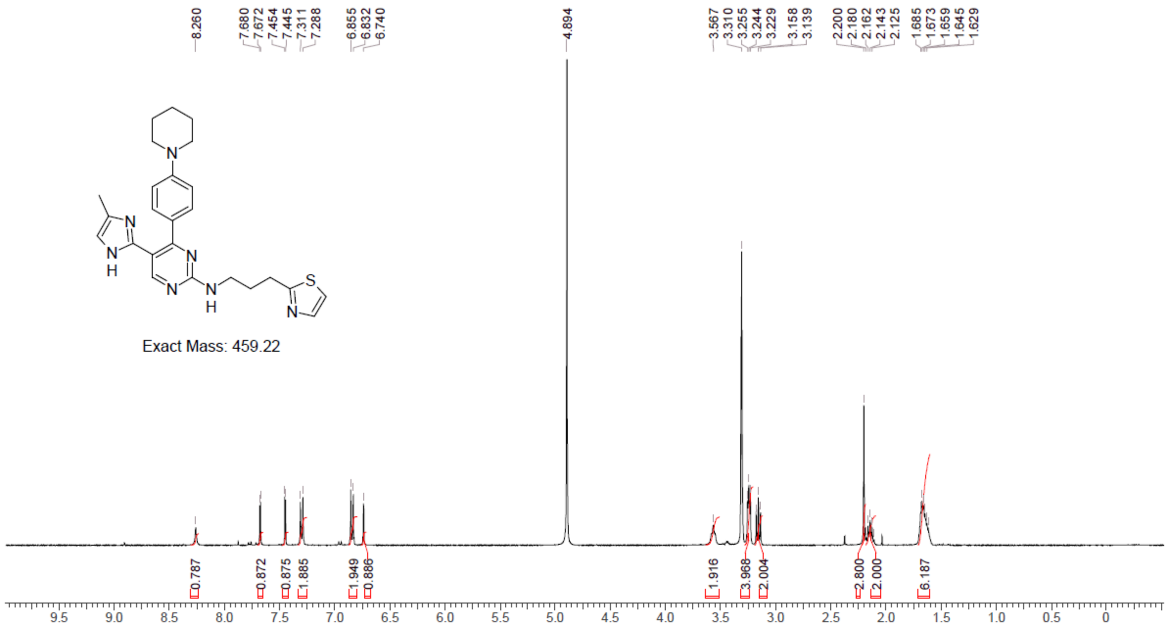

8.260
7.680 7.672 7.454 7.445 7.311 7.288
6.855 6.832 6.740
4.894
3.567 3.310 3.255 3.244 3.229 3.158 3.139
2.200 2.180 2.162 2.143 2.125
1.685 1.673 1.659 1.645 1.629
Exact Mass: 459.22
0.787 0.872 0.875 1.885 1.949 0.886 1.916 3.968 2.004 2.800 2.000 6.187

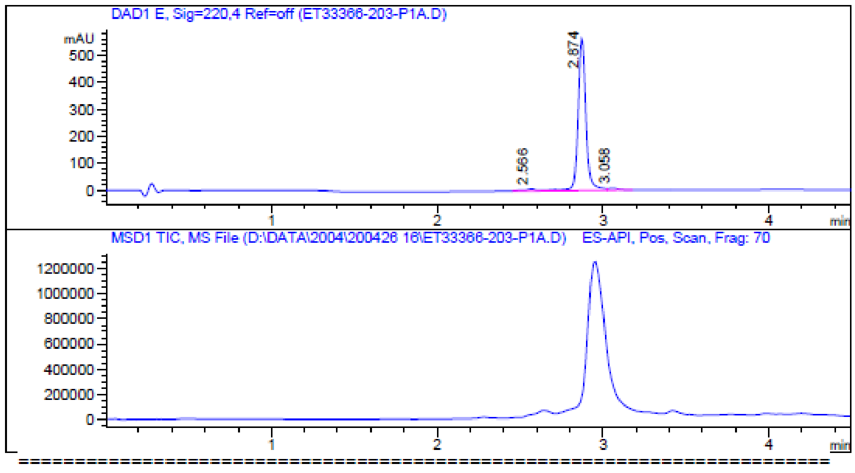

DAD1 E, Sig=220,4 Ref=off (ET33366-203-P1A.D)
mAU
2.874
2.566
3.058
min
MSD1 TIC, MS File (D:\DATA\2004\200426 16\ET33366-203-P1A.D) ES-API, Pos, Scan, Frag: 70
min

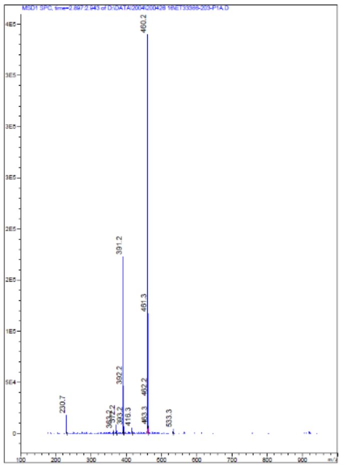

460.2
391.2
461.3

Compound 44

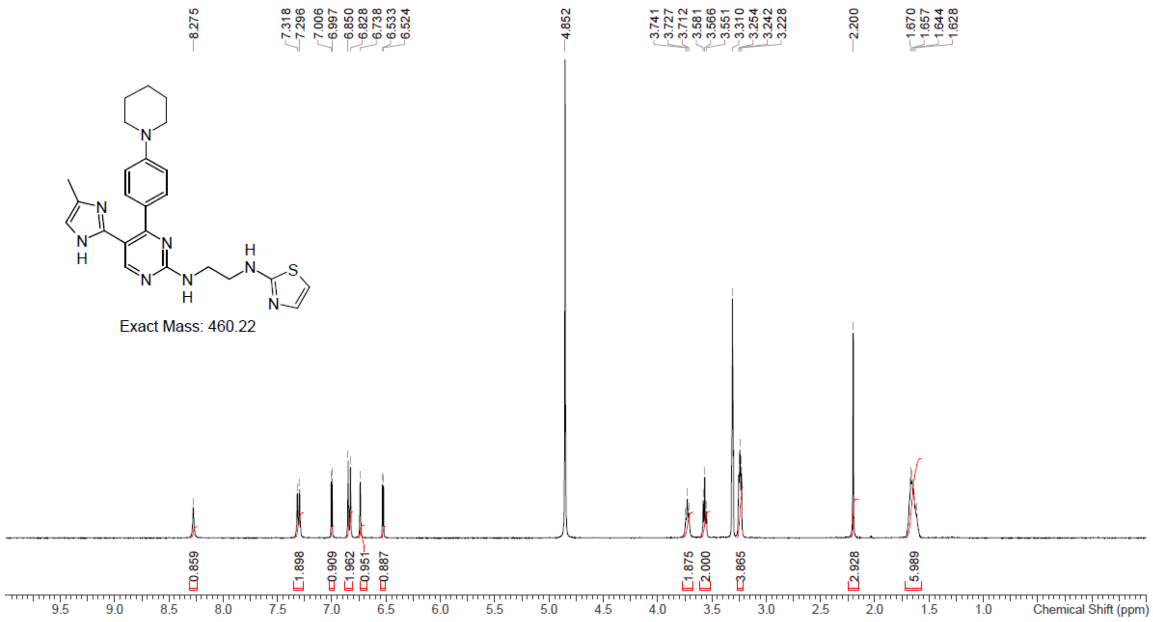

Exact Mass: 460.22
8.275
7.318
7.296
7.006
6.997
6.850
6.828
6.738
6.533
6.524
4.852
3.741
3.727
3.712
3.581
3.566
3.551
3.310
3.254
3.242
3.228
2.200
1.670
1.657
1.644
1.628
0.859
1.898
0.909
1.962
0.951
0.887
1.875
2.000
3.865
2.928
5.989
Chemical Shift (ppm)

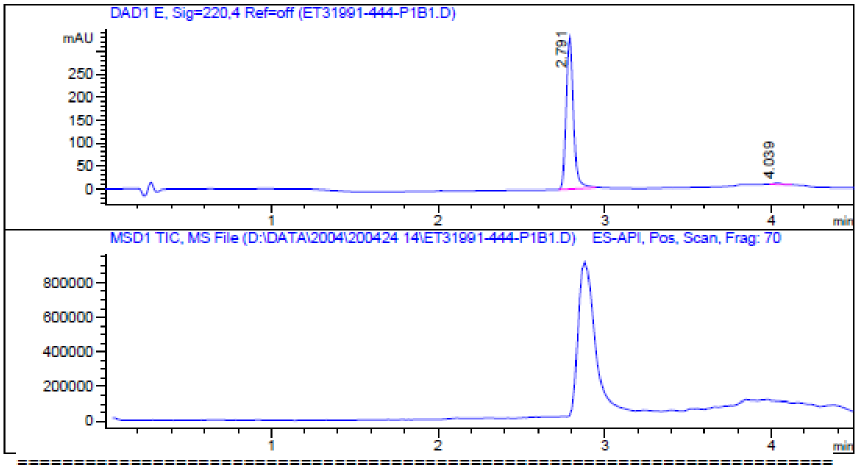

DAD1 E, Sig=220,4 Ref=off (ET31991-444-P1B1.D)
mAU
2.791
4.039
min
MSD1 TIC, MS File (D:\DATA\2004\200424 14\ET31991-444-P1B1.D) ES-API, Pos, Scan, Frag: 70
min

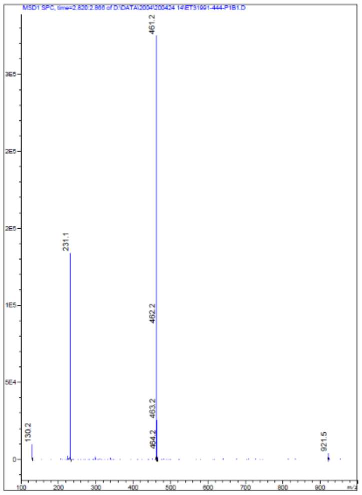

461.2
231.1
462.2
463.2
130.2
921.5

Compound 45

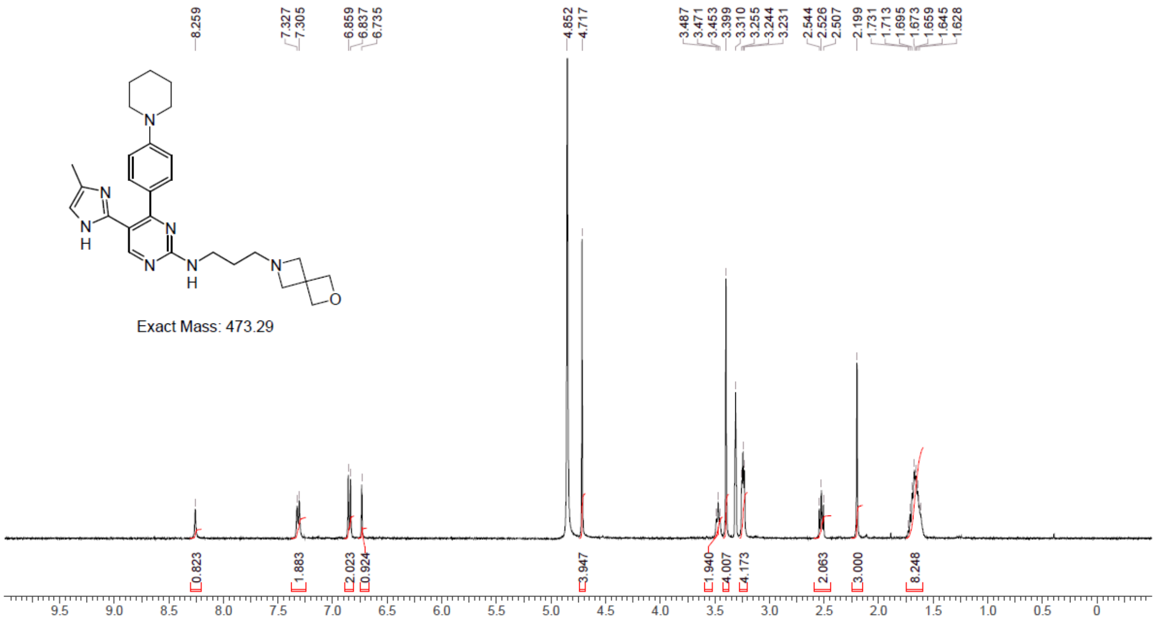

Exact Mass: 473.29
8.259
7.327
7.305
6.859
6.837
6.735
4.852
4.717
3.487
3.471
3.453
3.399
3.310
3.255
3.244
3.231
2.544
2.526
2.507
2.199
1.731
1.713
1.695
1.673
1.659
1.645
1.628
0.823
1.883
2.023
0.924
3.947
1.940
4.007
4.173
2.063
3.000
8.248

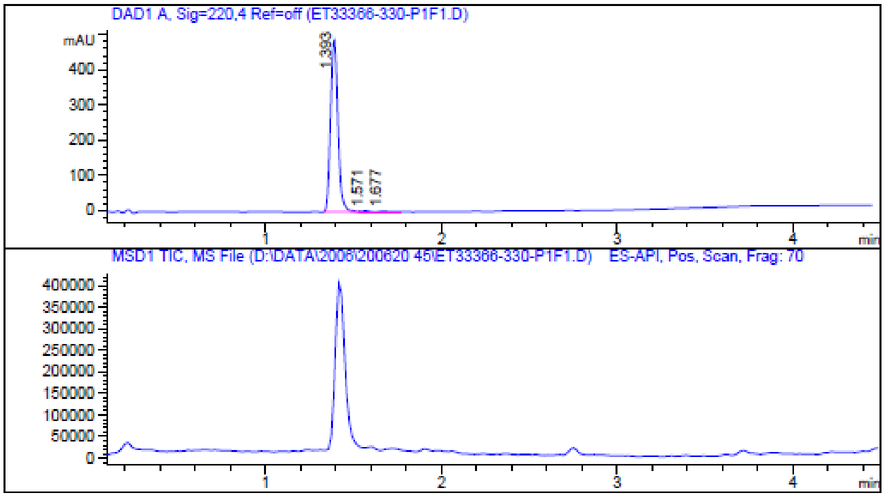

DAD1 A, Sig=220,4 Ref=off (ET33366-330-P1F1.D)
mAU
1.393
1.571
1.677
min
MSD1 TIC, MS File (D:\DATA\2006\200620 45\ET33366-330-P1F1.D) ES-API, Pos, Scan, Frag: 70
min

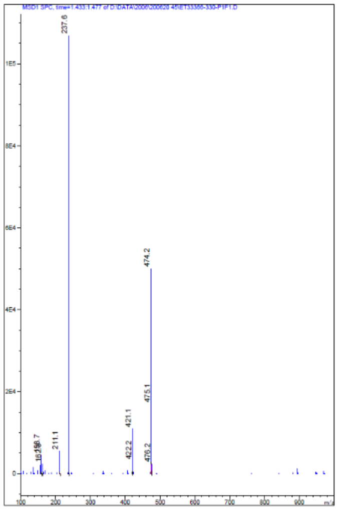

237.6
474.2
475.1
421.1
422.2
476.2
211.1

Compound 46

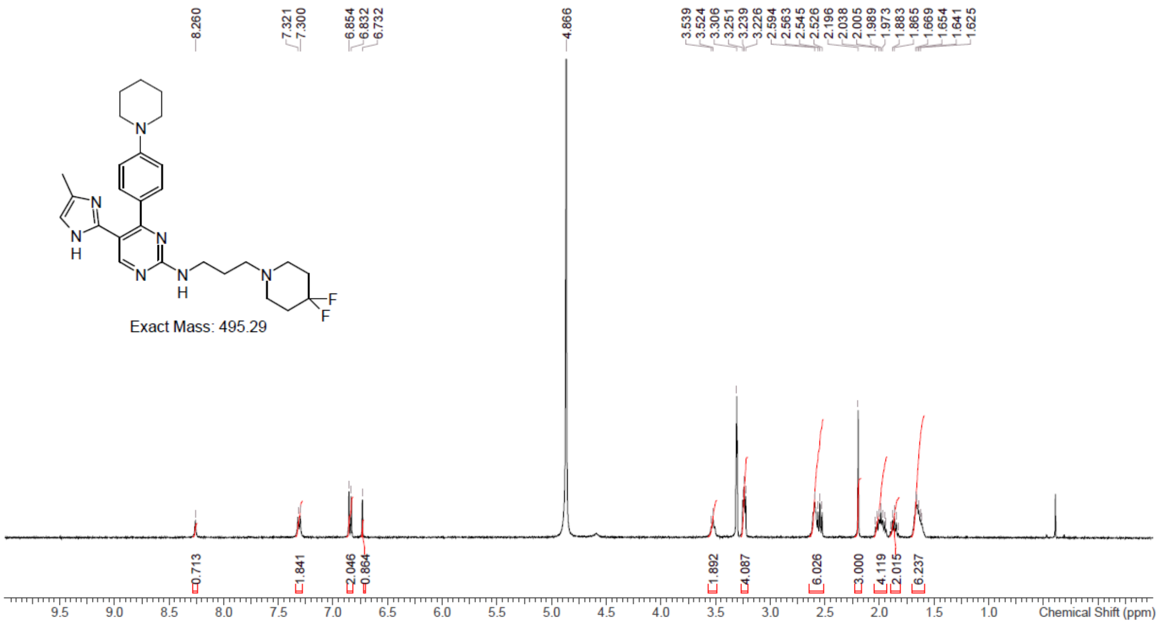

Exact Mass: 495.29
Chemical Shift (ppm)

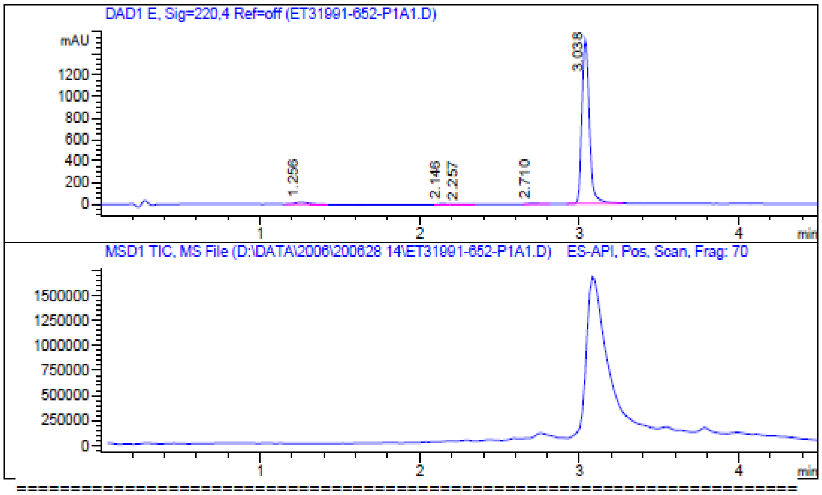

DAD1 E, Sig=220,4 Ref=off (ET31991-652-P1A1.D)
mAU
MSD1 TIC, MS File (D:\DATA\2006\200628 14\ET31991-652-P1A1.D) ES-API, Pos, Scan, Frag: 70

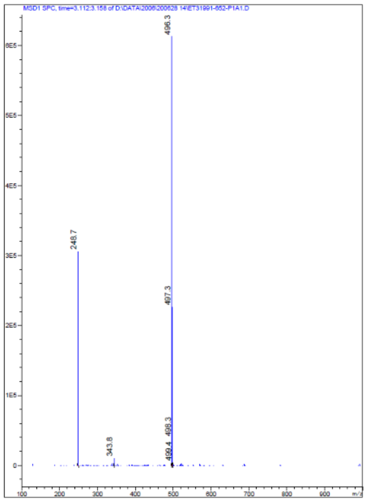

496.3
248.7
497.3

Compound 47

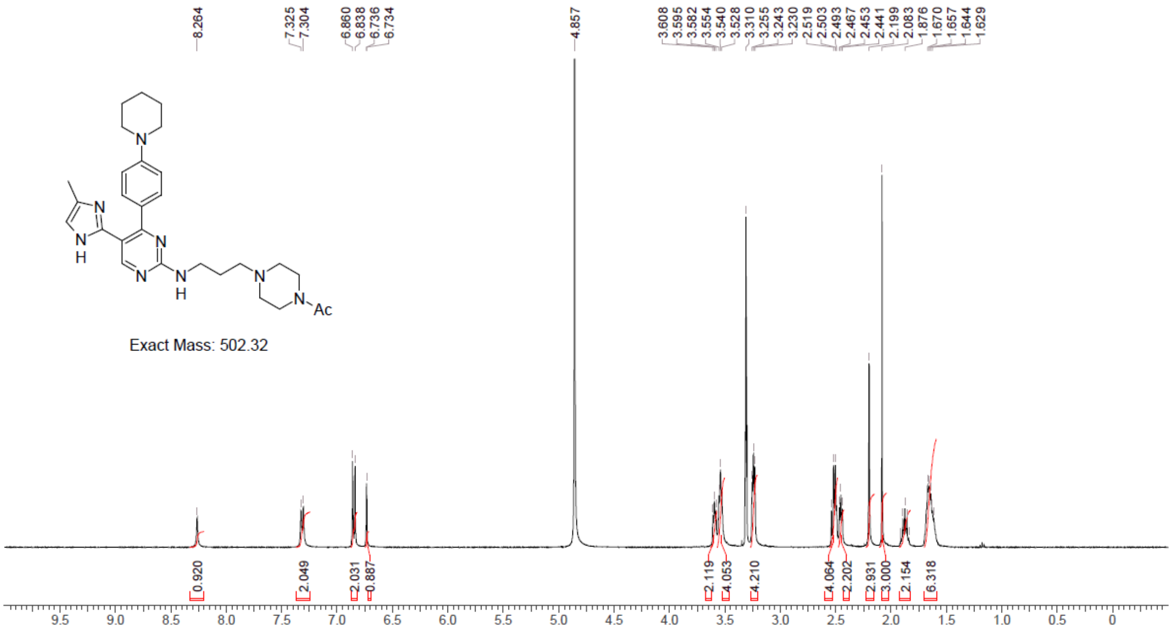

Exact Mass: 502.32

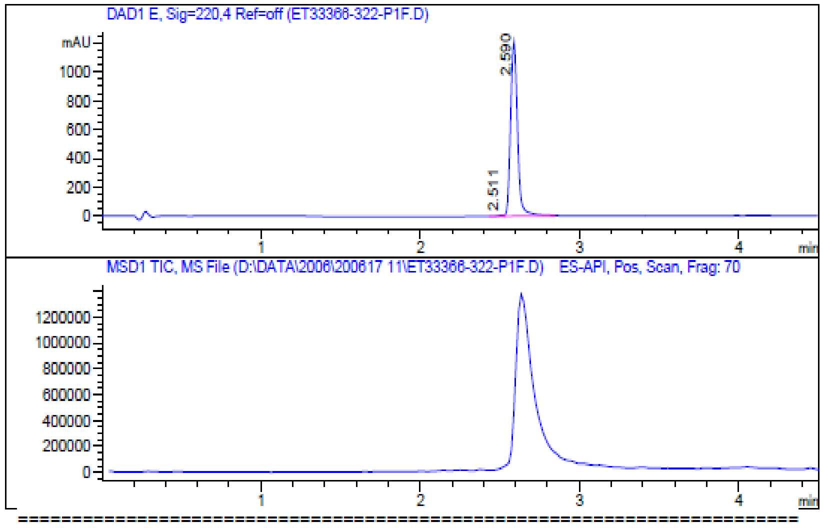

DAD1 E, Sig=220,4 Ref=off (ET33366-322-P1F.D)
mAU
2.590
2.511
MSD1 TIC, MS File (D:\DATA\2006\200617 11\ET33366-322-P1F.D) ES-API, Pos, Scan, Frag: 70

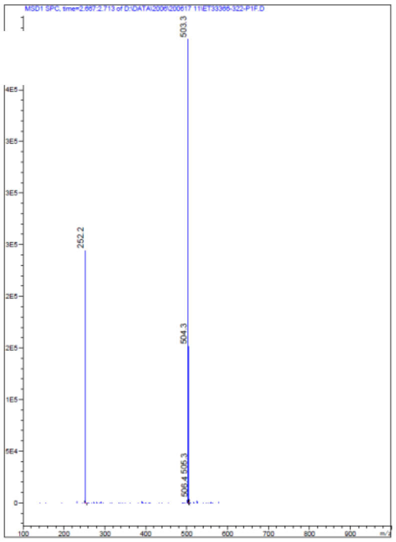

503.3
252.2
504.3

Compound 48

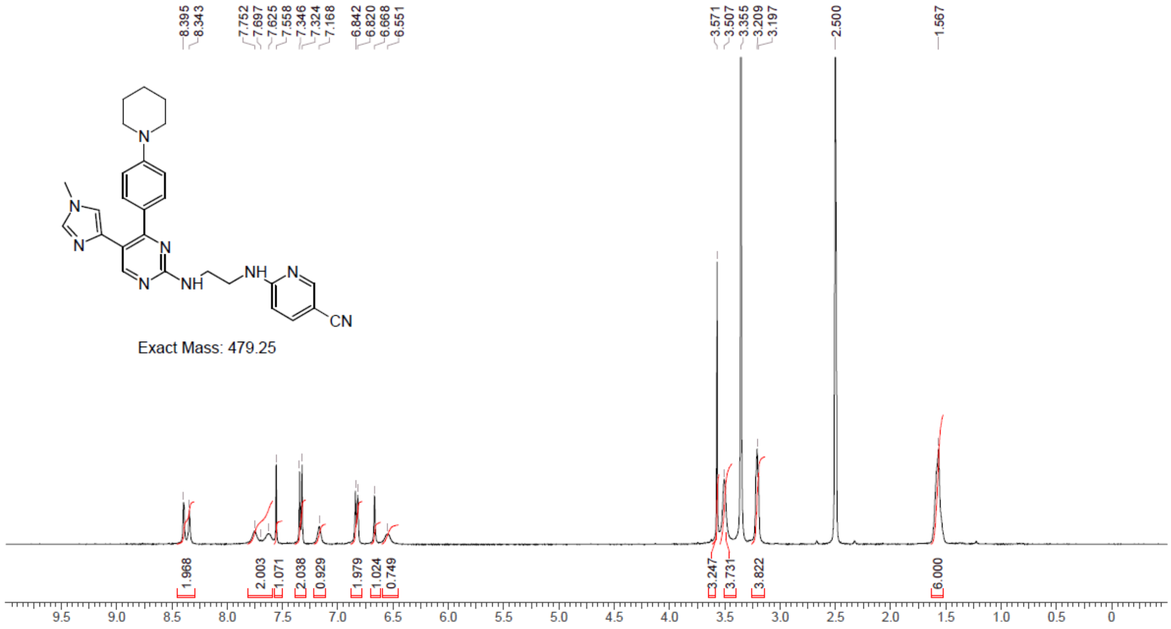
Exact Mass: 479.25

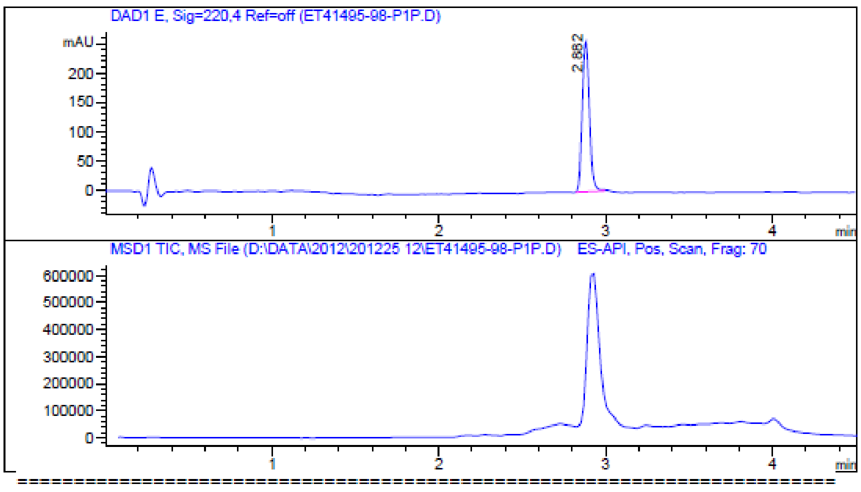
DAD1 E, Sig=220,4 Ref=off (ET41495-98-P1P.D)
MSD1 TIC, MS File (D:\DATA\2012\201225 12\ET41495-98-P1P.D) ES-API, Pos, Scan, Frag: 70

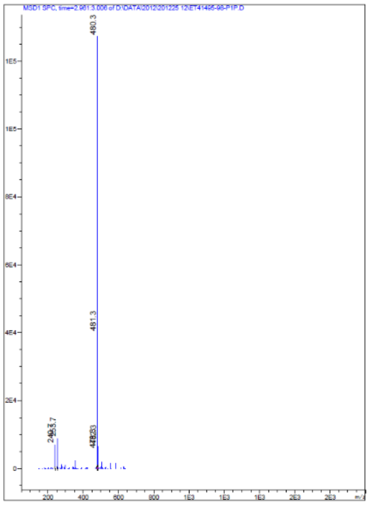
480.3
481.3

Compound 49

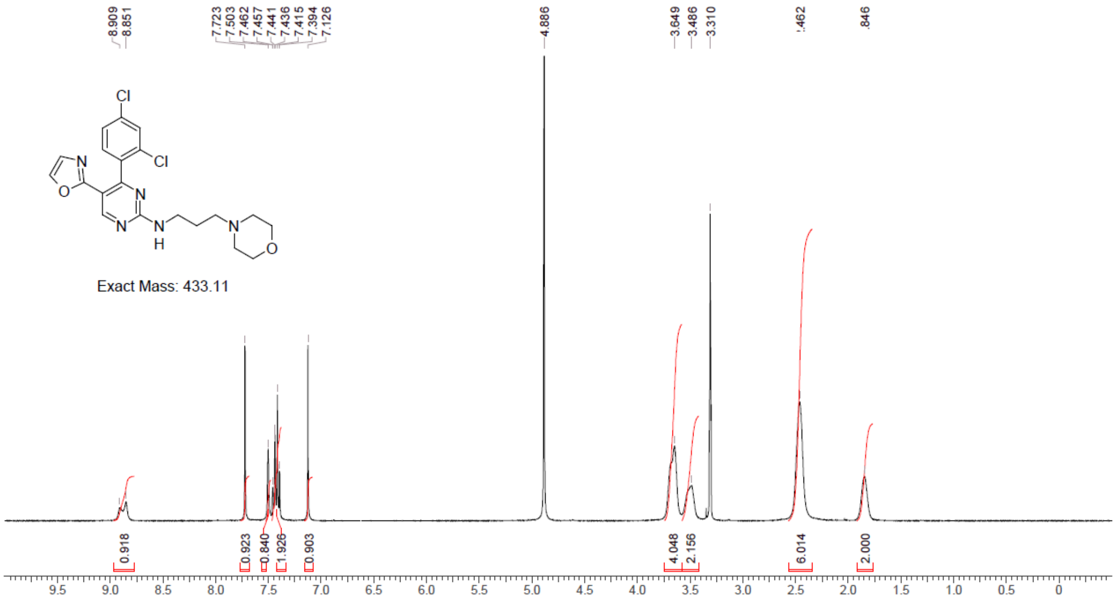

8.909
8.851
7.723
7.503
7.462
7.457
7.441
7.436
7.415
7.394
7.126
4.886
3.649
3.486
3.310
.462
.846
Cl
Cl
N
O
N
N
N
H
N
O
Exact Mass: 433.11
0.918
0.923
0.840
1.926
0.903
4.048
2.156
6.014
2.000
9.5 9.0 8.5 8.0 7.5 7.0 6.5 6.0 5.5 5.0 4.5 4.0 3.5 3.0 2.5 2.0 1.5 1.0 0.5 0

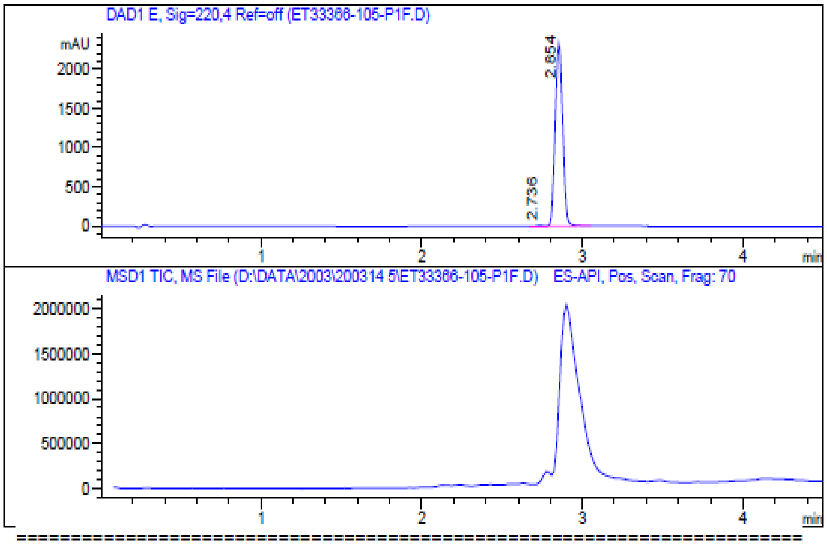

DAD1 E, Sig=220,4 Ref=off (ET33366-105-P1F.D)
mAU
2000
1500
1000
500
0
2.854
2.736
1 2 3 4 min
MSD1 TIC, MS File (D:\DATA\2003\200314 5\ET33366-105-P1F.D) ES-API, Pos, Scan, Frag: 70
2000000
1500000
1000000
500000
0
1 2 3 4 min

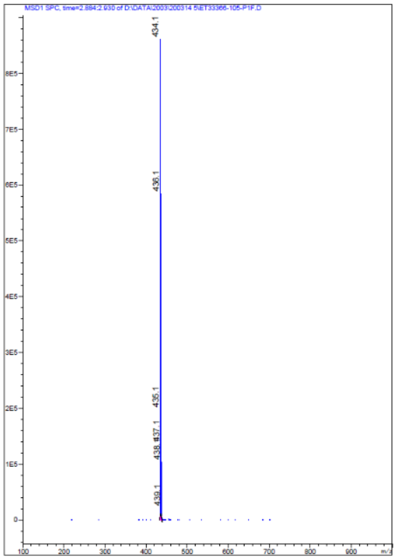

434.1
436.1
435.1
437.1
438.1
439.1

Compound 50

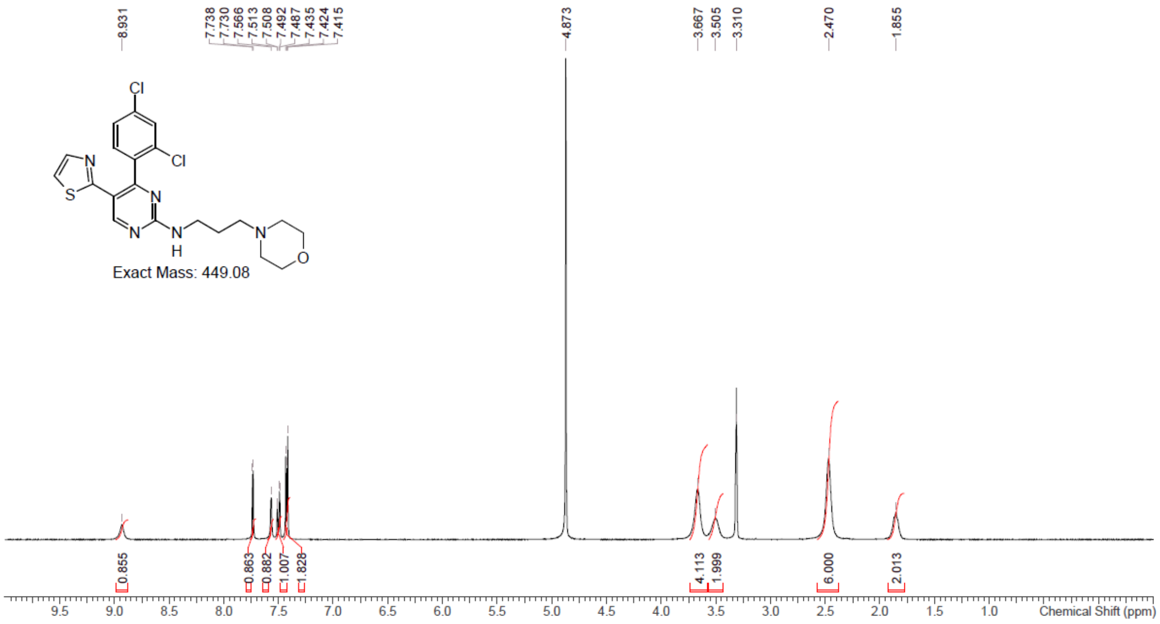

8.931
7.738
7.730
7.566
7.513
7.508
7.492
7.487
7.435
7.424
7.415
4.873
3.667
3.505
3.310
2.470
1.855
Cl
Cl
N
S
N
N
N
H
N
O
Exact Mass: 449.08
0.855
0.863
0.882
1.007
1.828
4.113
1.999
6.000
2.013
9.5
9.0
8.5
8.0
7.5
7.0
6.5
6.0
5.5
5.0
4.5
4.0
3.5
3.0
2.5
2.0
1.5
1.0
Chemical Shift (ppm)

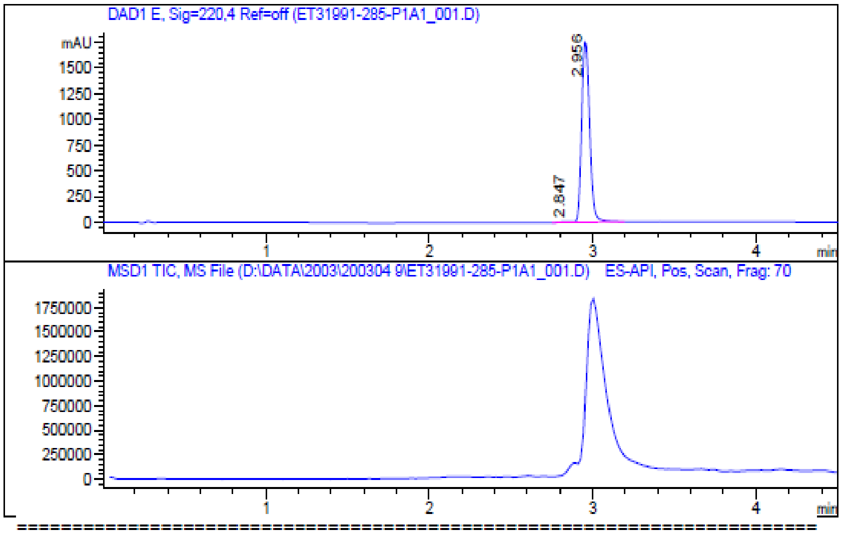

DAD1 E, Sig=220,4 Ref=off (ET31991-285-P1A1_001.D)
mAU
1500
1250
1000
750
500
250
0
2.956
2.847
1
2
3
4
min
MSD1 TIC, MS File (D:\DATA\2003\200304 9\ET31991-285-P1A1_001.D) ES-API, Pos, Scan, Frag: 70
1750000
1500000
1250000
1000000
750000
500000
250000
0
1
2
3
4
min

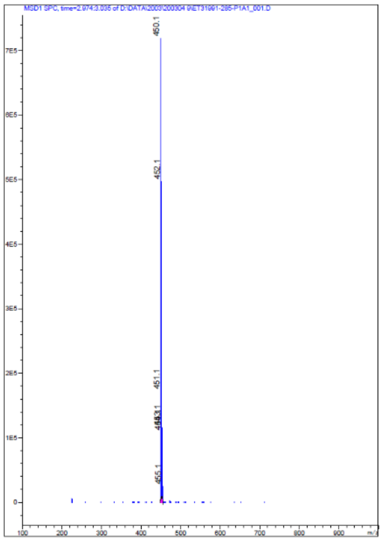

450.1
452.1
451.1

Compound 51

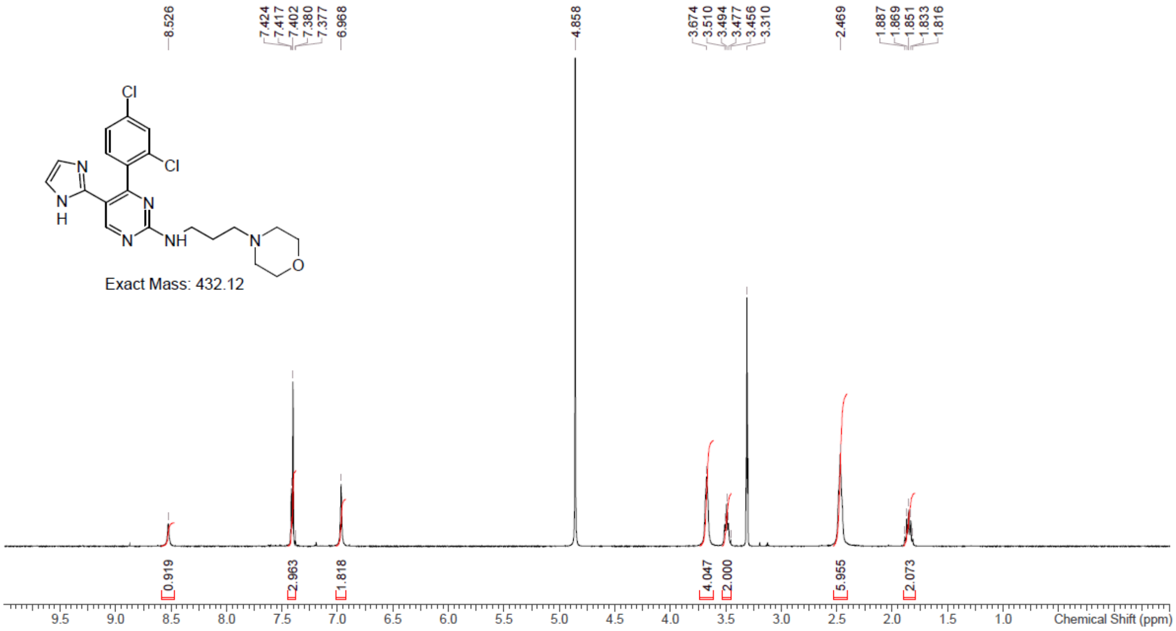

8.526
7.424
7.417
7.402
7.380
7.377
6.968
4.858
3.674
3.510
3.494
3.477
3.456
3.310
2.469
1.887
1.869
1.851
1.833
1.816
Cl
Cl
N
N
H
N
N
NH
N
O
Exact Mass: 432.12
0.919
2.963
1.818
4.047
2.000
5.955
2.073
Chemical Shift (ppm)

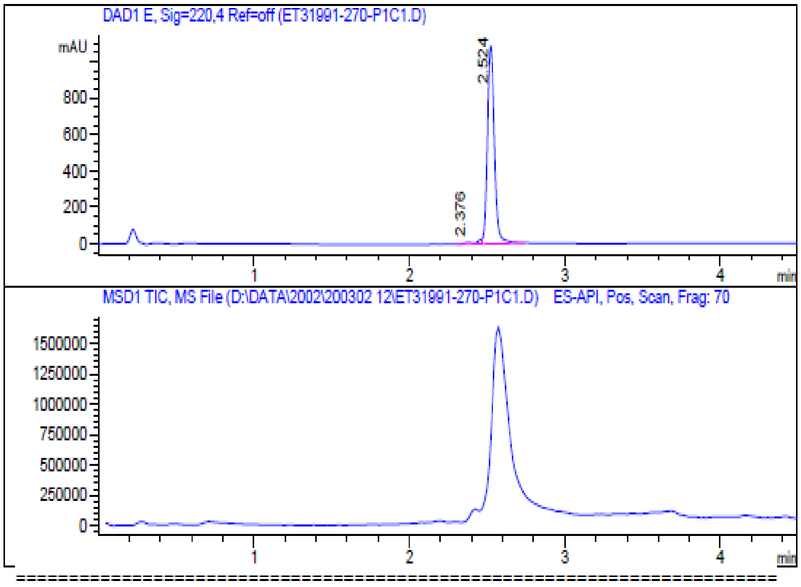

DAD1 E, Sig=220,4 Ref=off (ET31991-270-P1C1.D)
mAU
2.524
2.376
min
MSD1 TIC, MS File (D:\DATA\2002\200302 12\ET31991-270-P1C1.D) ES-API, Pos, Scan, Frag: 70
min

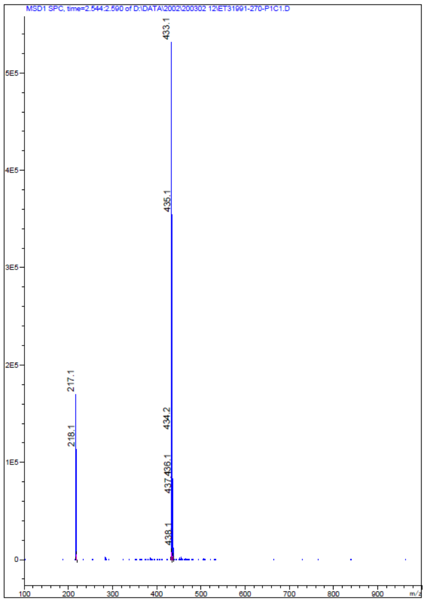

433.1
435.1
217.1
218.1
434.2
437.436.1

Compound 52

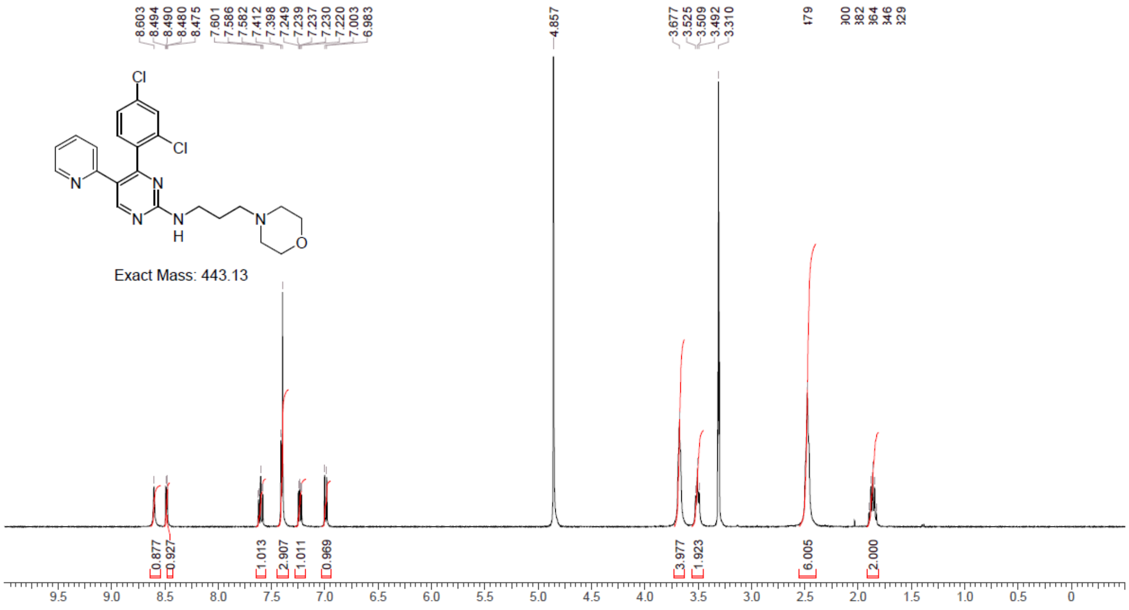
Exact Mass: 443.13

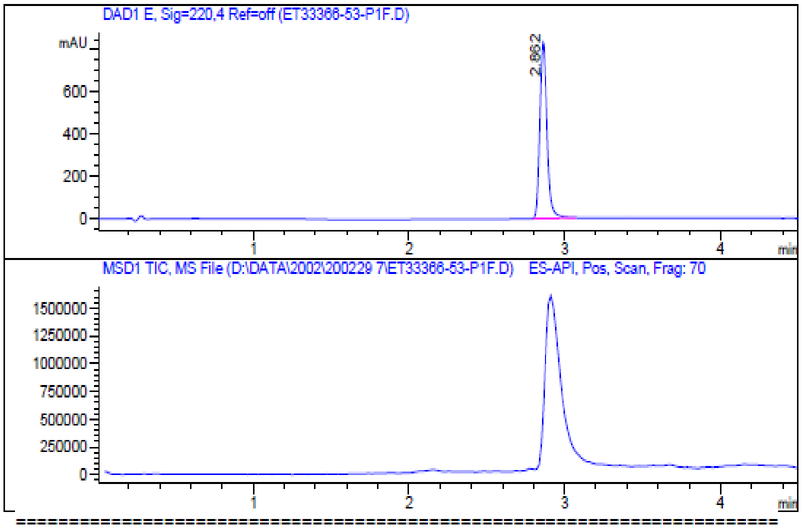
DAD1 E, Sig=220,4 Ref=off (ET33366-53-P1F.D)
MSD1 TIC, MS File (D:\DATA\2002\200229 7\ET33366-53-P1F.D)   ES-API, Pos, Scan, Frag: 70

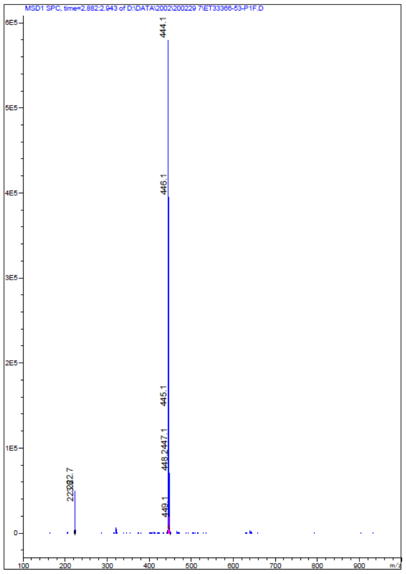

Compound 53

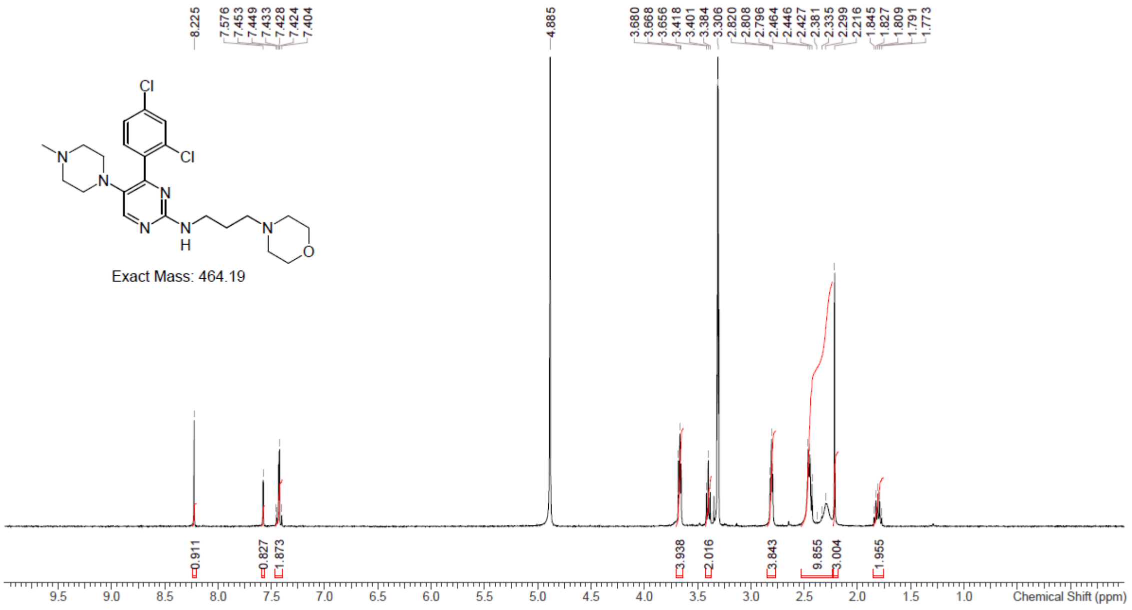

Exact Mass: 464.19
8.225
7.576
7.453
7.449
7.433
7.428
7.424
7.404
4.885
3.680
3.668
3.656
3.418
3.401
3.384
3.306
2.820
2.808
2.796
2.464
2.446
2.427
2.381
2.335
2.299
2.216
1.845
1.827
1.809
1.791
1.773
0.911
0.827
1.873
3.938
2.016
3.843
9.855
3.004
1.955
Chemical Shift (ppm)

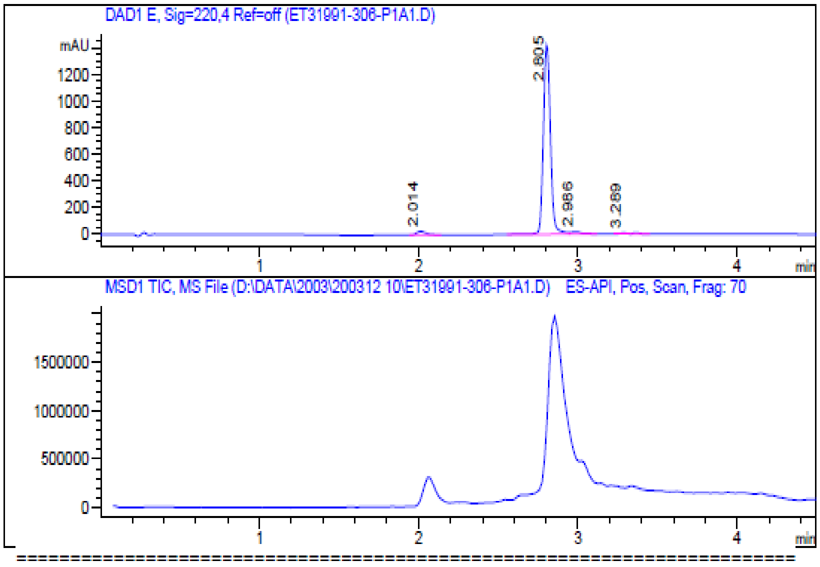

DAD1 E, Sig=220,4 Ref=off (ET31991-306-P1A1.D)
mAU
2.805
2.014
2.986
3.269
min
MSD1 TIC, MS File (D:\DATA\2003\200312 10\ET31991-306-P1A1.D) ES-API, Pos, Scan, Frag: 70
min

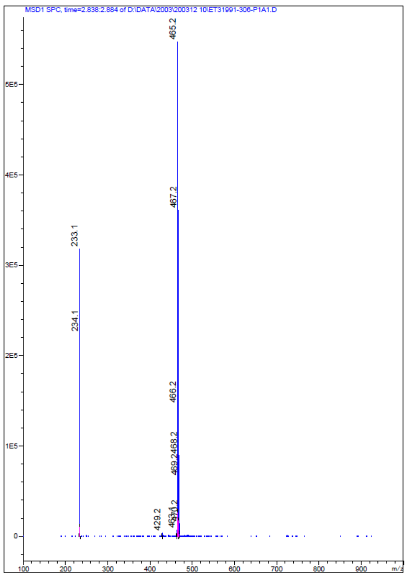

465.2
467.2
233.1
234.1
466.2
468.2
429.2

Compound 54

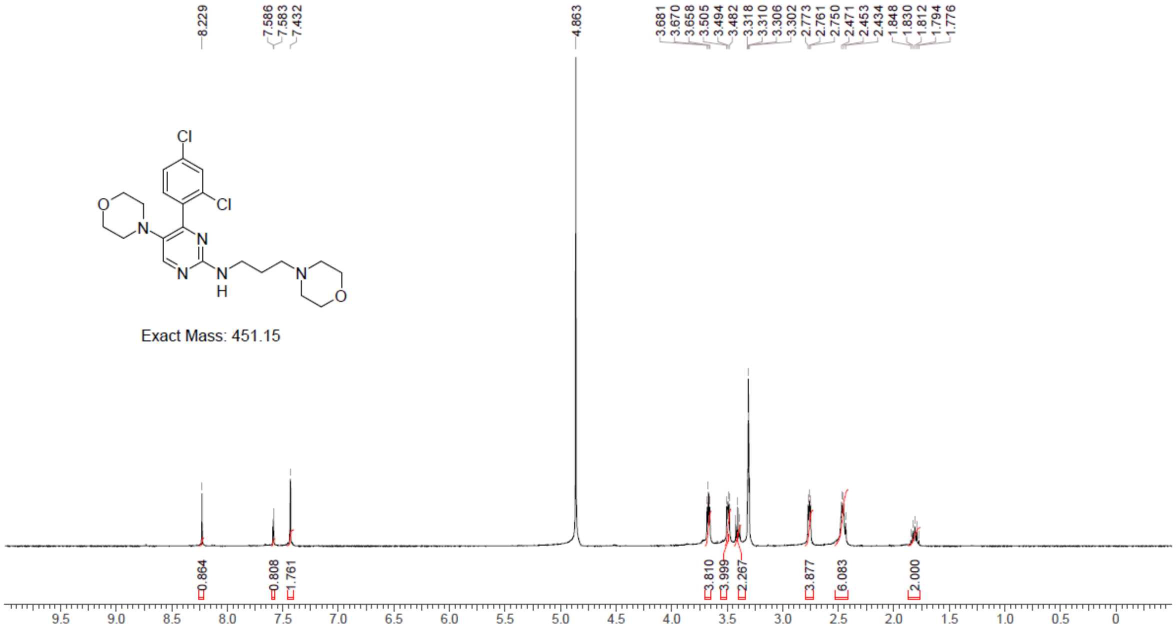

Exact Mass: 451.15

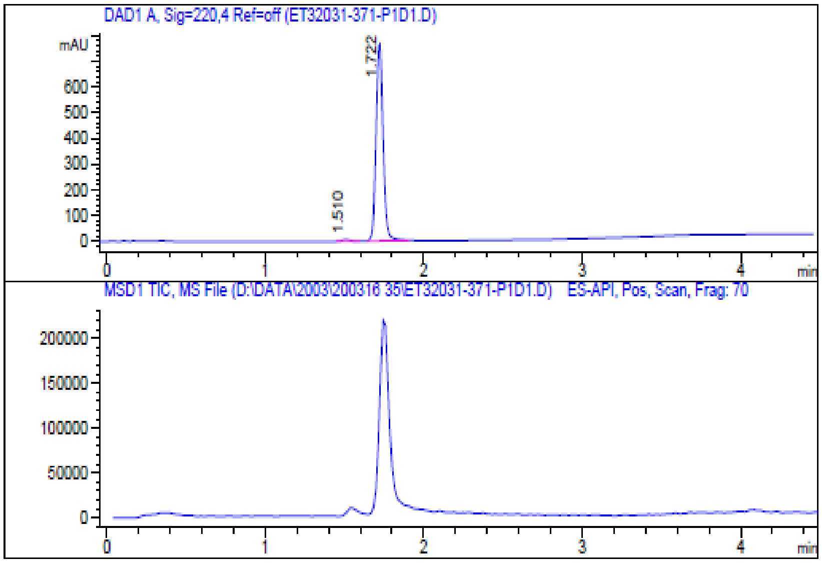

DAD1 A, Sig=220,4 Ref=off (ET32031-371-P1D1.D)
1.722
1.510
MSD1 TIC, MS File (D:\DATA\2003\200316 35\ET32031-371-P1D1.D) ES-API, Pos, Scan, Frag: 70

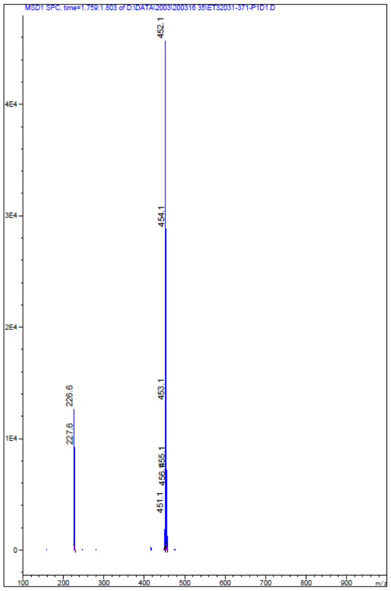

452.1
454.1
453.1
226.6
227.6

Compound 56

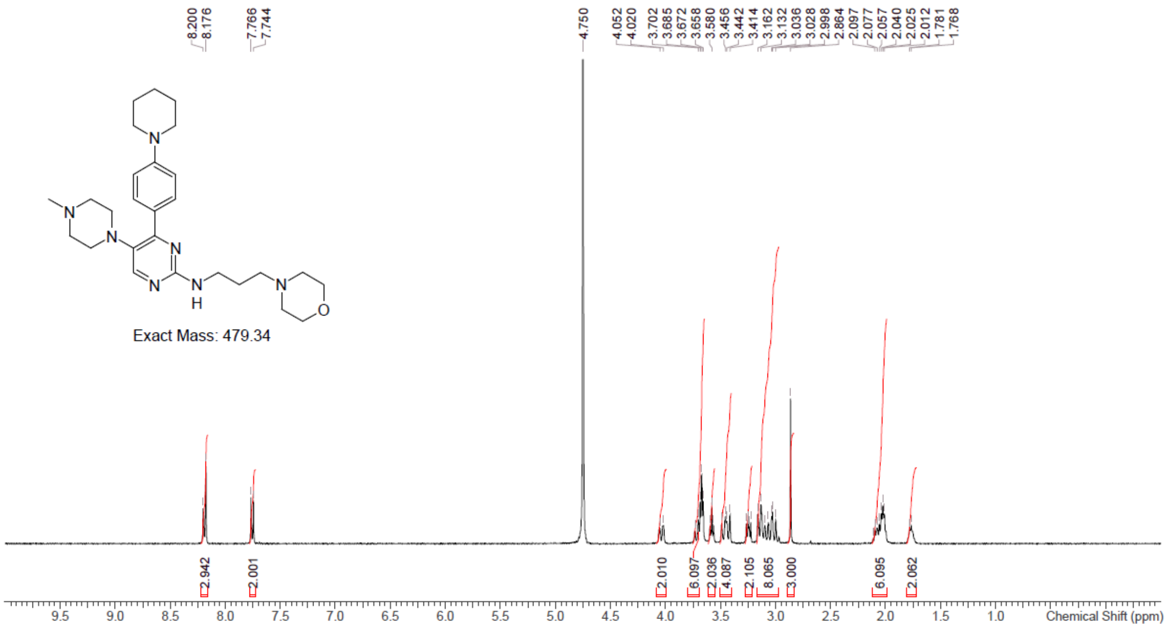

Exact Mass: 479.34
Chemical Shift (ppm)

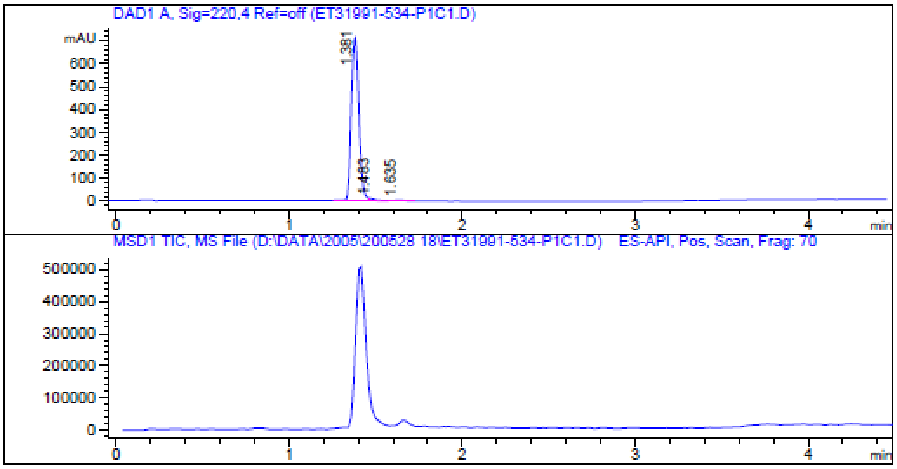

DAD1 A, Sig=220,4 Ref=off (ET31991-534-P1C1.D)
mAU
1.381
1.483
1.635
min
MSD1 TIC, MS File (D:\DATA\2005\200528 18\ET31991-534-P1C1.D) ES-API, Pos, Scan, Frag: 70
min

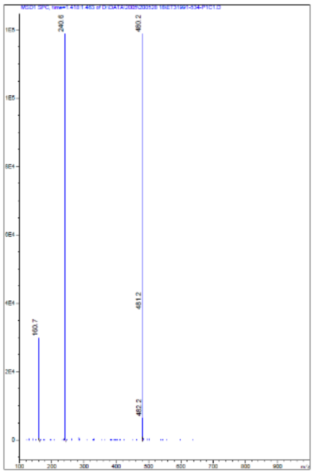

240.6
480.2
160.7
481.2
482.2

Compound 57

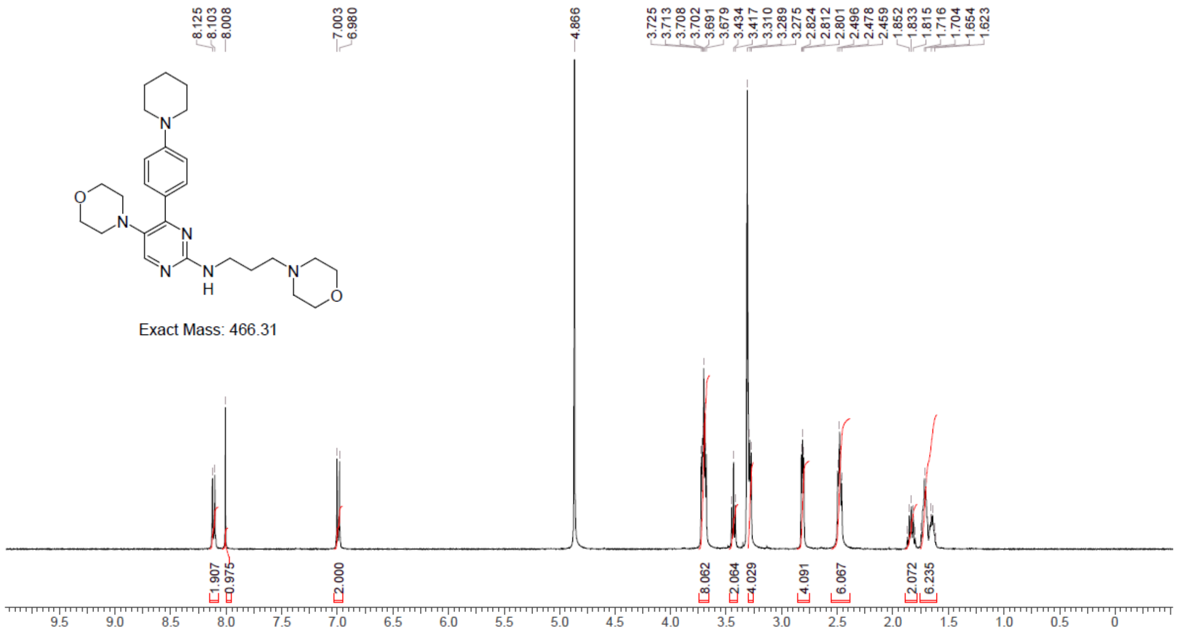

8.125
8.103
8.008
7.003
6.980
4.866
3.725
3.713
3.708
3.702
3.691
3.679
3.434
3.417
3.310
3.289
3.275
2.824
2.812
2.801
2.496
2.478
2.459
1.852
1.833
1.815
1.716
1.704
1.654
1.623
Exact Mass: 466.31
1.907
0.975
2.000
8.062
2.064
4.029
4.091
6.067
2.072
6.235
9.5 9.0 8.5 8.0 7.5 7.0 6.5 6.0 5.5 5.0 4.5 4.0 3.5 3.0 2.5 2.0 1.5 1.0 0.5 0

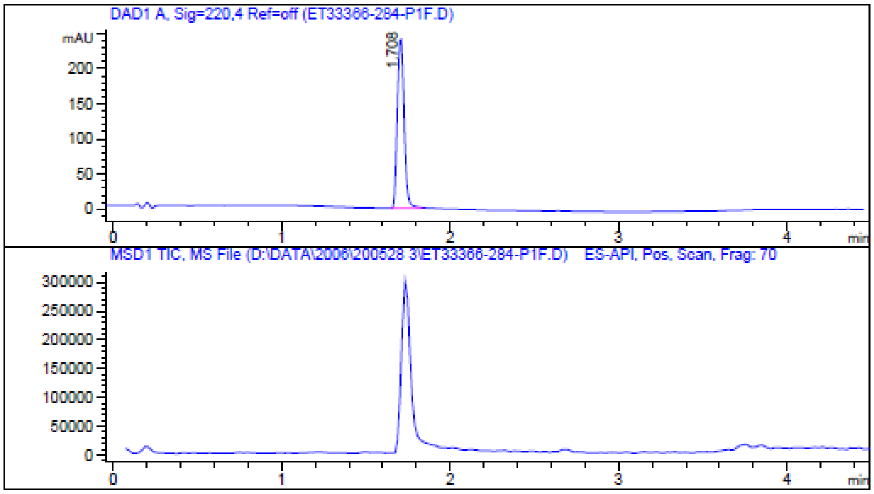

DAD1 A, Sig=220,4 Ref=off (ET33366-284-P1F.D)
mAU
1.708
MSD1 TIC, MS File (D:\DATA\2006\200528 3\ET33366-284-P1F.D) ES-API, Pos, Scan, Frag: 70
min

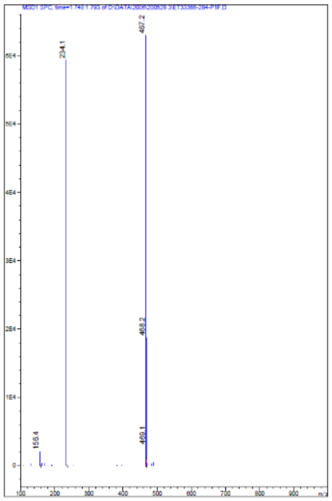

467.2
234.1
468.2
469.1
156.4

Compound 58

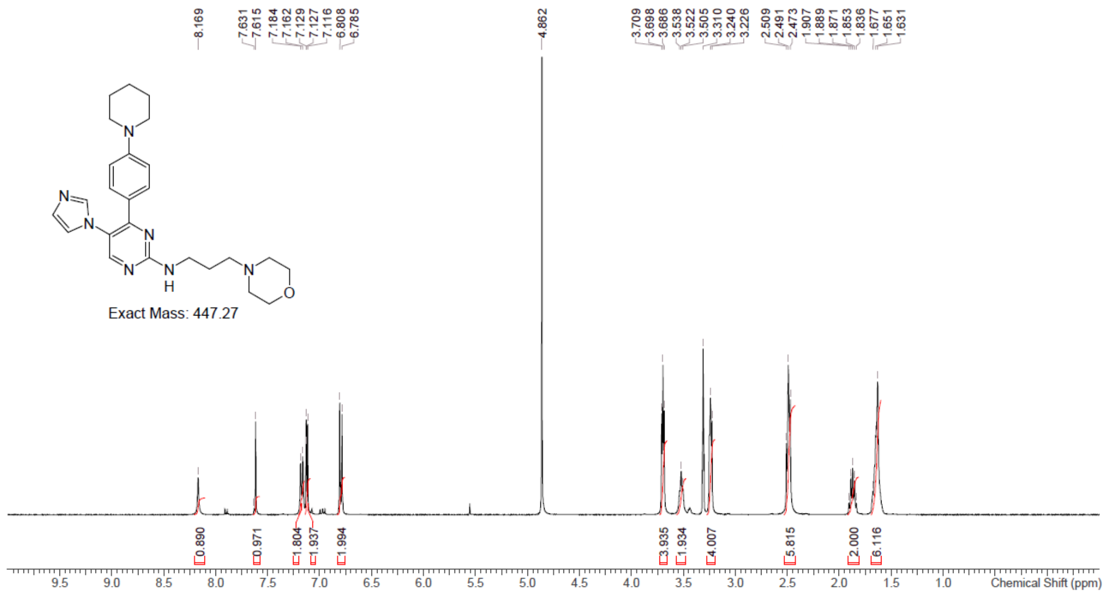

Exact Mass: 447.27
Chemical Shift (ppm)

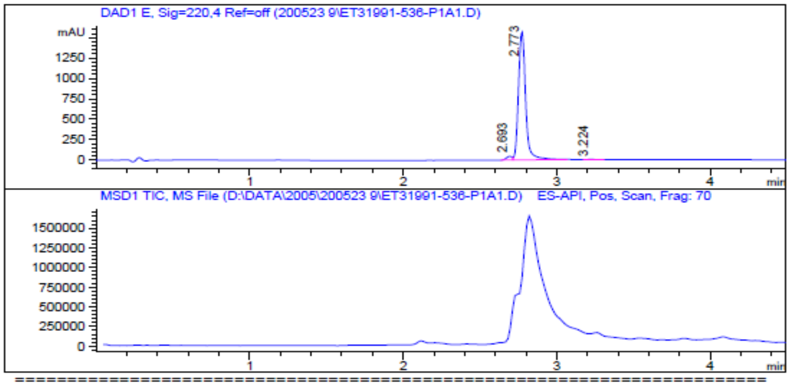

DAD1 E, Sig=220,4 Ref=off (200523 9\ET31991-536-P1A1.D)
mAU
2.773
2.693
3.224
MSD1 TIC, MS File (D:\DATA\2005\200523 9\ET31991-536-P1A1.D) ES-API, Pos, Scan, Frag: 70

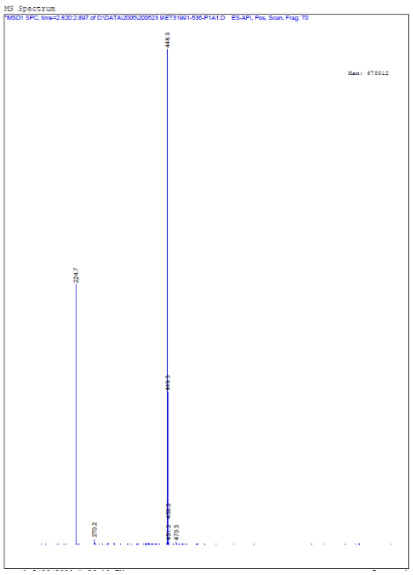

MS Spectrum
448.3
224.7

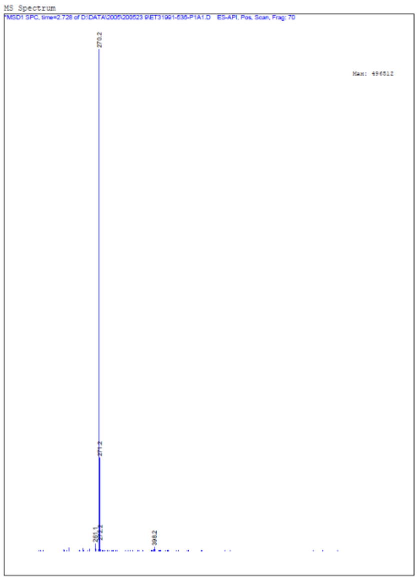

MS Spectrum
270.2
271.2

Compound 59

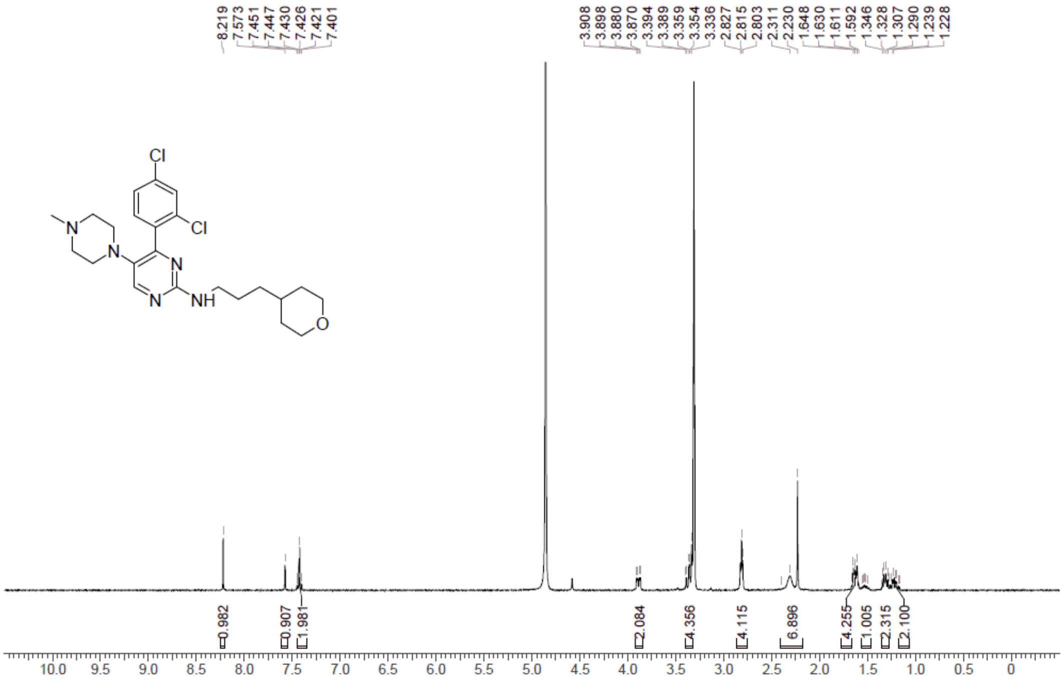

Cl
Cl
N
N
N
N
NH
O
8.219
7.573
7.451
7.447
7.430
7.426
7.421
7.401
3.908
3.898
3.880
3.870
3.394
3.389
3.359
3.354
3.336
2.827
2.815
2.803
2.311
2.230
1.648
1.630
1.611
1.592
1.346
1.328
1.307
1.290
1.239
1.228
0.982
0.907
1.981
2.084
4.356
4.115
6.896
4.255
1.005
2.315
2.100

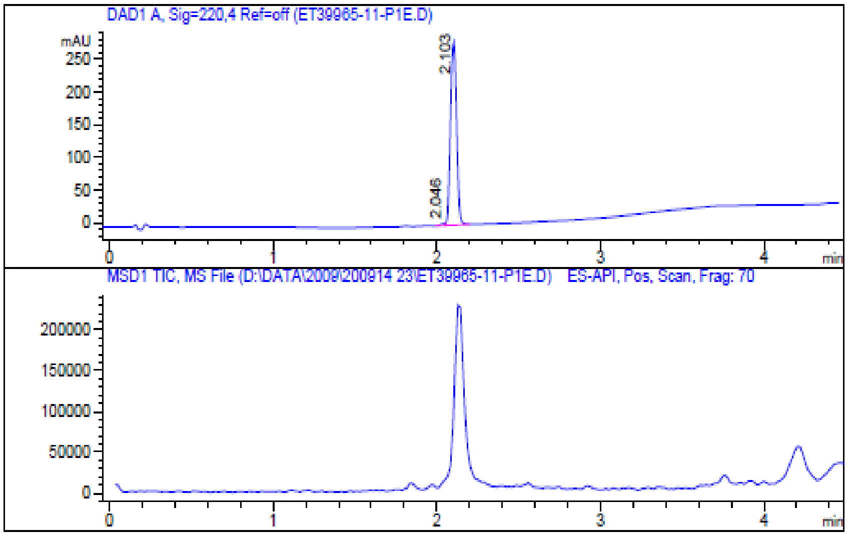

DAD1 A, Sig=220,4 Ref=off (ET39965-11-P1E.D)
mAU
2.103
2.046
min
MSD1 TIC, MS File (D:\DATA\2009\200914 23\ET39965-11-P1E.D) ES-API, Pos, Scan, Frag: 70
min

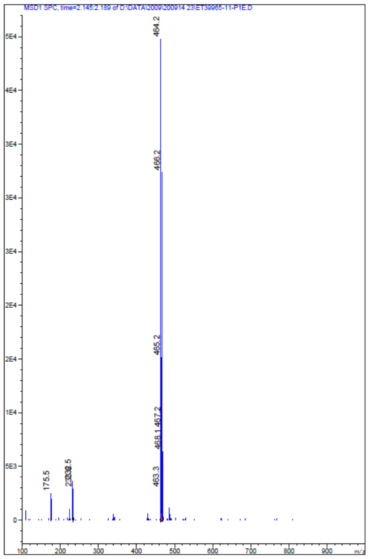

464.2
466.2
465.2
467.2
175.5

Compound 60

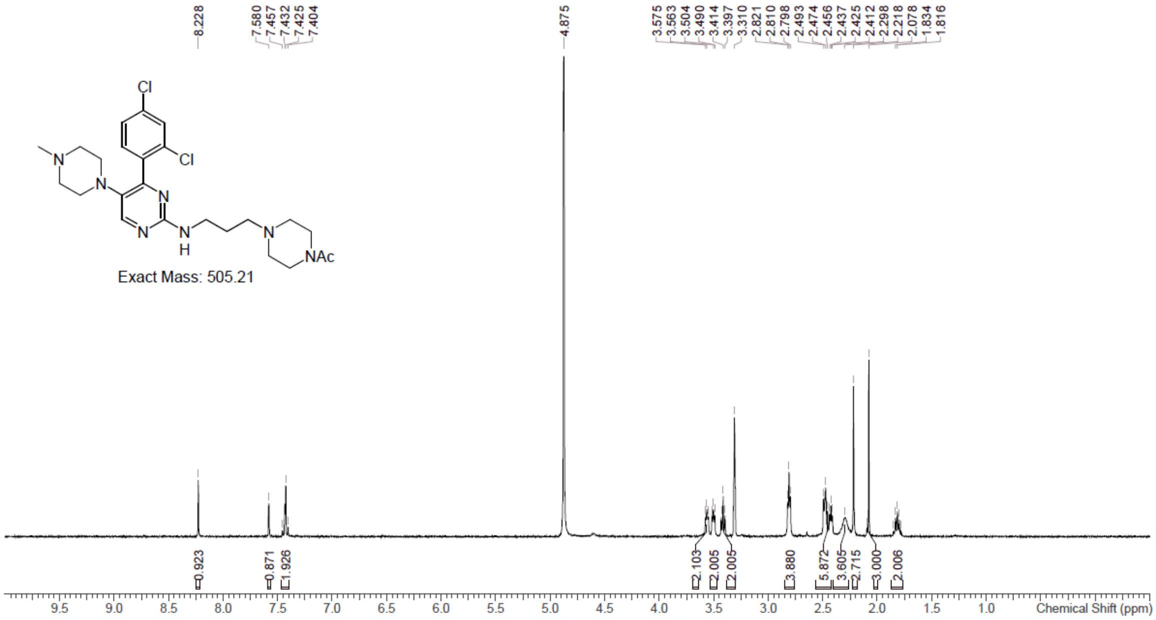
Cl
Cl
N
N
N
N
N
H
N
NAc
Exact Mass: 505.21
8.228
7.580
7.457
7.432
7.425
7.404
4.875
3.575
3.563
3.504
3.490
3.414
3.397
3.310
2.821
2.810
2.798
2.493
2.474
2.456
2.437
2.425
2.412
2.298
2.218
2.078
1.834
1.816
0.923
0.871
1.926
2.103
2.005
2.005
3.880
5.872
3.605
2.715
3.000
2.006
Chemical Shift (ppm)

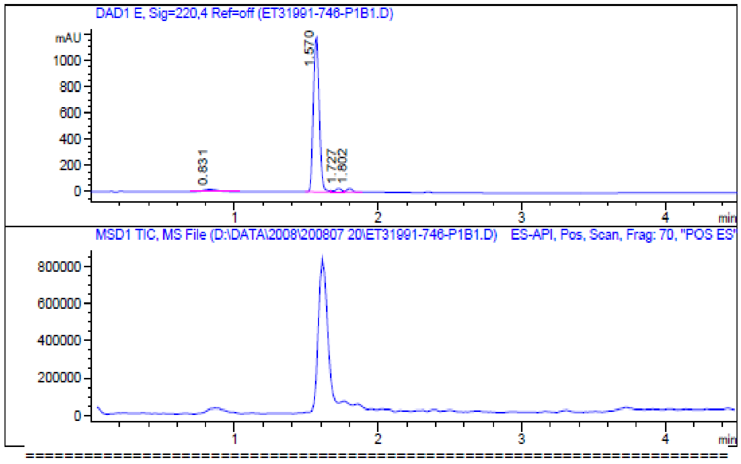
DAD1 E, Sig=220,4 Ref=off (ET31991-746-P1B1.D)
mAU
1.570
0.831
1.727
1.802
MSD1 TIC, MS File (D:\DATA\2008\200807 20\ET31991-746-P1B1.D) ES-API, Pos, Scan, Frag: 70, "POS ES"
min

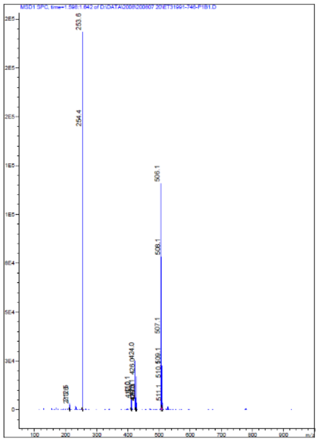
253.6
254.4
506.1
508.1
507.1

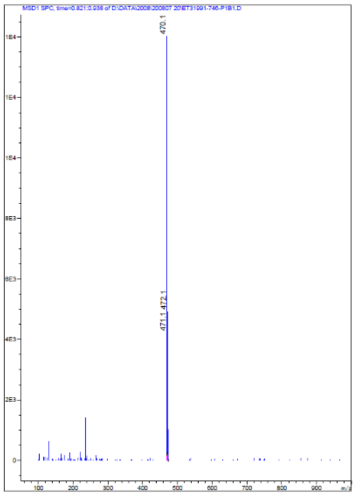
470.1

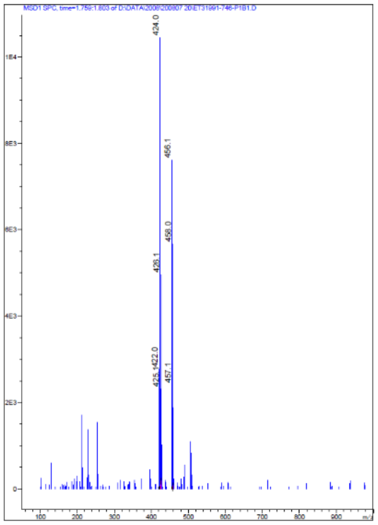
424.0
456.1
458.0
426.1
457.1

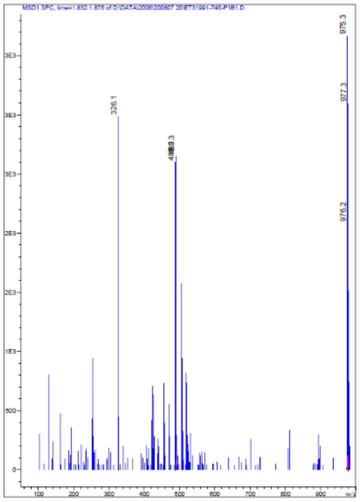
975.3
977.3
976.2
326.1

Compound 61

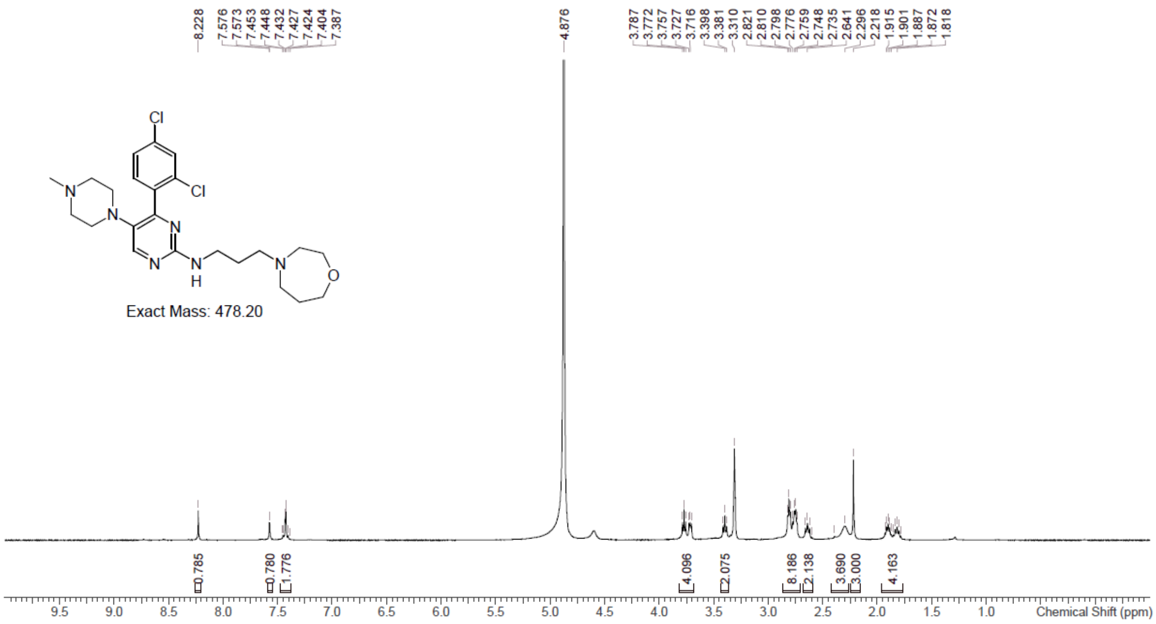

Exact Mass: 478.20
Chemical Shift (ppm)

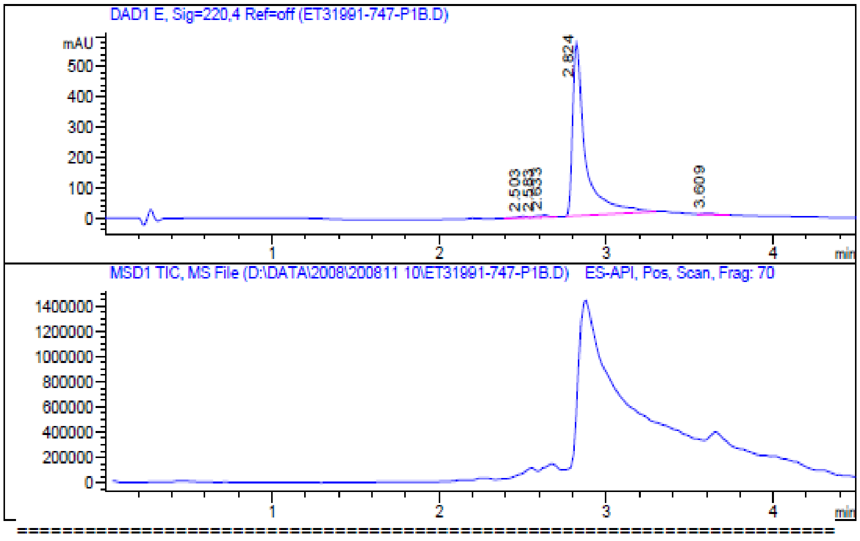

DAD1 E, Sig=220,4 Ref=off (ET31991-747-P1B.D)
MSD1 TIC, MS File (D:\DATA\2008\200811 10\ET31991-747-P1B.D) ES-API, Pos, Scan, Frag: 70

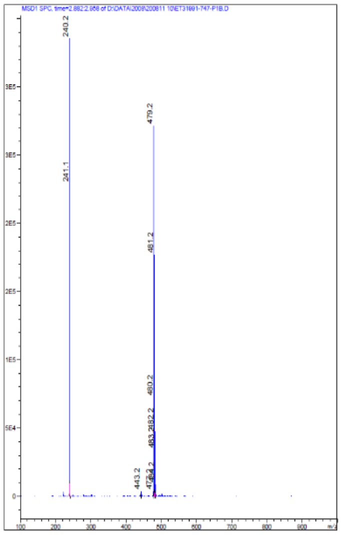

Compound 62

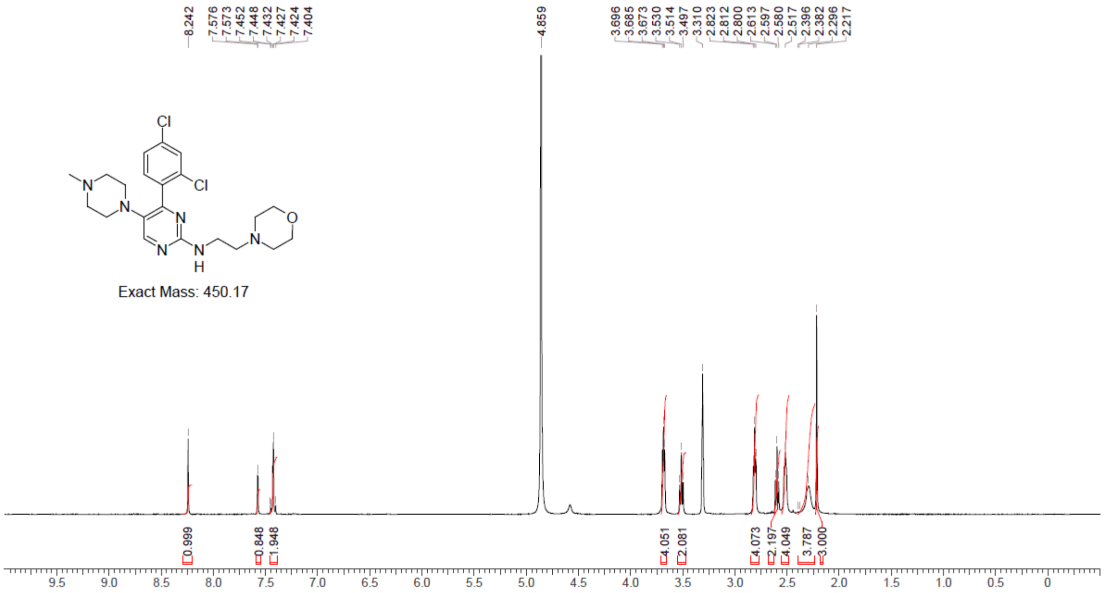

Exact Mass: 450.17
8.242
7.576 7.573 7.452 7.448 7.432 7.427 7.424 7.404
4.859
3.696 3.685 3.673 3.530 3.514 3.497 3.310 2.823 2.812 2.800 2.613 2.597 2.580 2.517 2.396 2.382 2.296 2.217
0.999 0.848 1.948 4.051 2.081 4.073 2.197 4.049 3.787 3.000

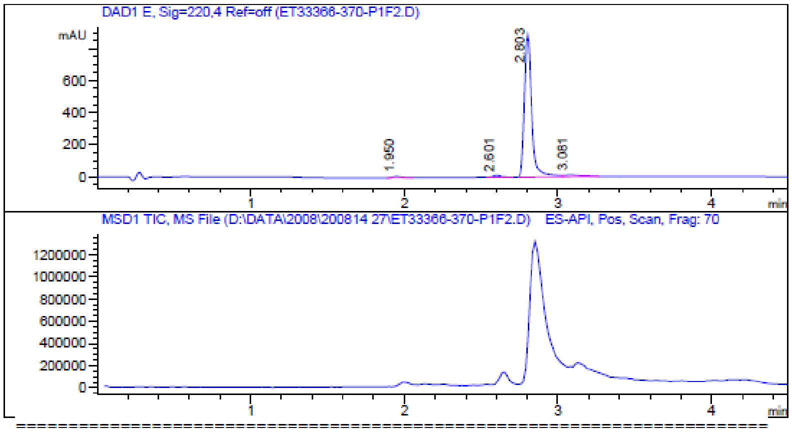

DAD1 E, Sig=220,4 Ref=off (ET33366-370-P1F2.D)
mAU
2.803
1.950
2.601
3.081
min
MSD1 TIC, MS File (D:\DATA\2008\200814 27\ET33366-370-P1F2.D) ES-API, Pos, Scan, Frag: 70
min

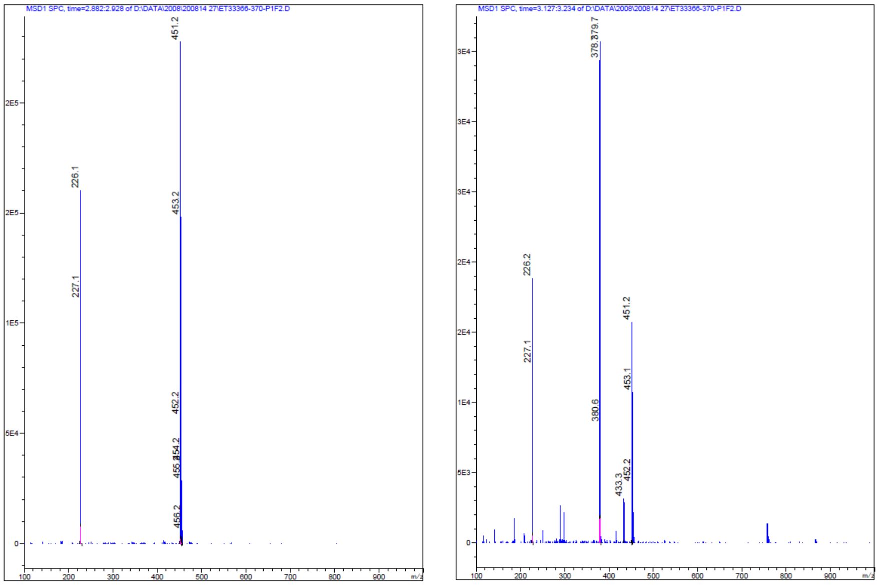

451.2
226.1
453.2
227.1
452.2
378.879.7
226.2
227.1
451.2
453.1
380.6
433.3
452.2

Compound 63

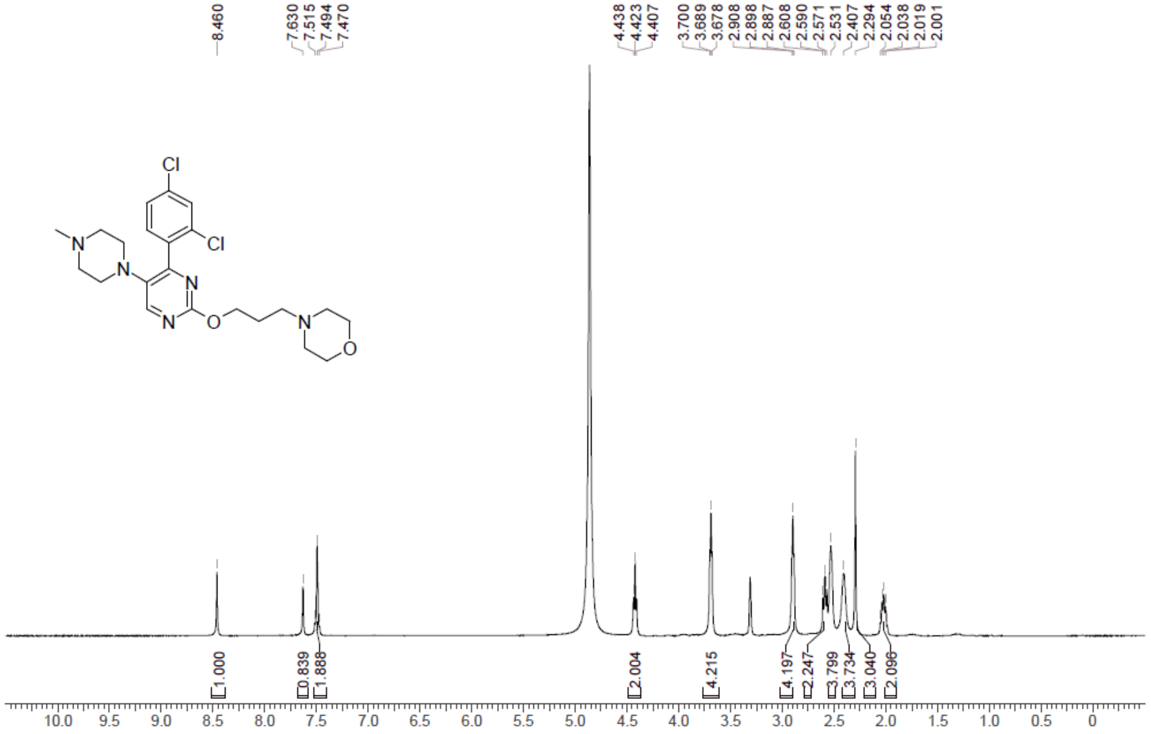

8.460
7.630
7.515
7.494
7.470
4.438
4.423
4.407
3.700
3.689
3.678
2.908
2.898
2.887
2.608
2.590
2.571
2.531
2.407
2.294
2.054
2.038
2.019
2.001
Cl
Cl
N
N
N
N
O
N
O
1.000
0.839
1.888
2.004
4.215
4.197
2.247
3.799
3.734
3.040
2.096
10.0 9.5 9.0 8.5 8.0 7.5 7.0 6.5 6.0 5.5 5.0 4.5 4.0 3.5 3.0 2.5 2.0 1.5 1.0 0.5 0

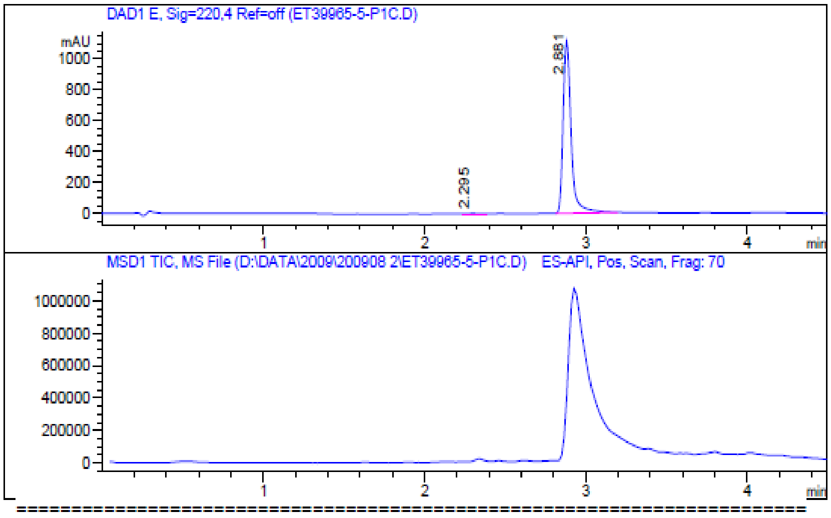

DAD1 E, Sig=220,4 Ref=off (ET39965-5-P1C.D)
mAU
2.881
2.295
MSD1 TIC, MS File (D:\DATA\2009\200908 2\ET39965-5-P1C.D) ES-API, Pos, Scan, Frag: 70
min

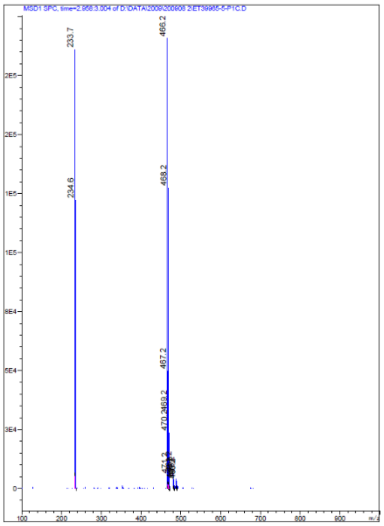

Compound 64

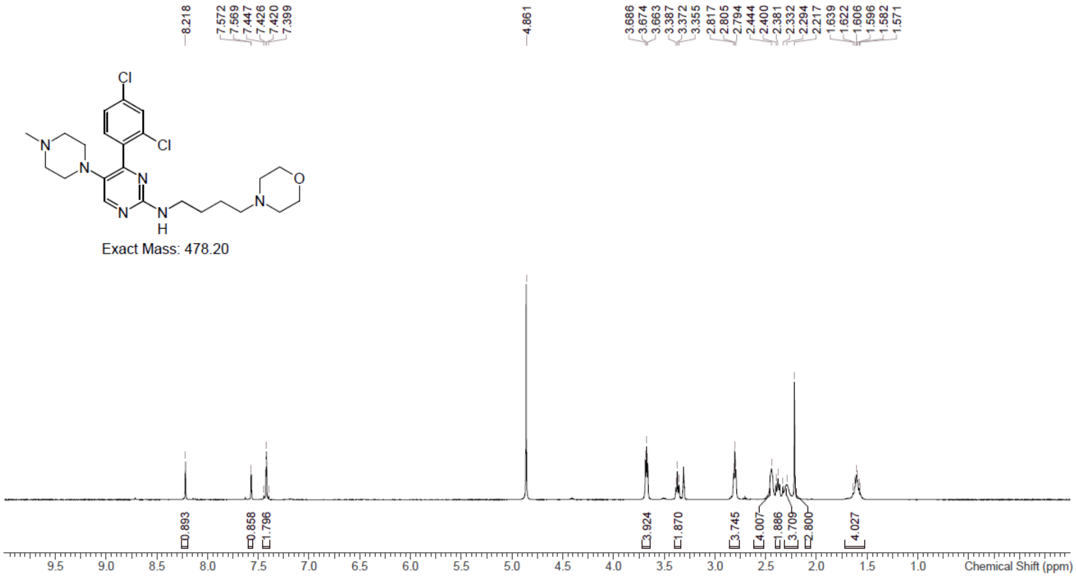
Exact Mass: 478.20
Chemical Shift (ppm)

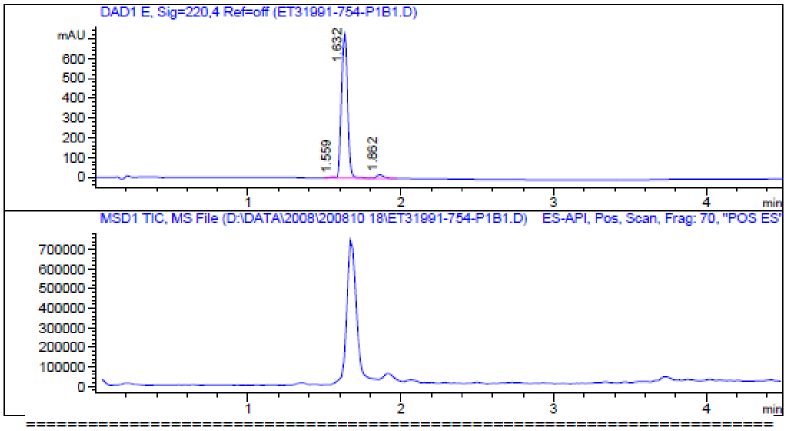
DAD1 E, Sig=220,4 Ref=off (ET31991-754-P1B1.D)
mAU
1.632
1.559
1.862
min
MSD1 TIC, MS File (D:\DATA\2008\200810 18\ET31991-754-P1B1.D) ES-API, Pos, Scan, Frag: 70, "POS ES"
min

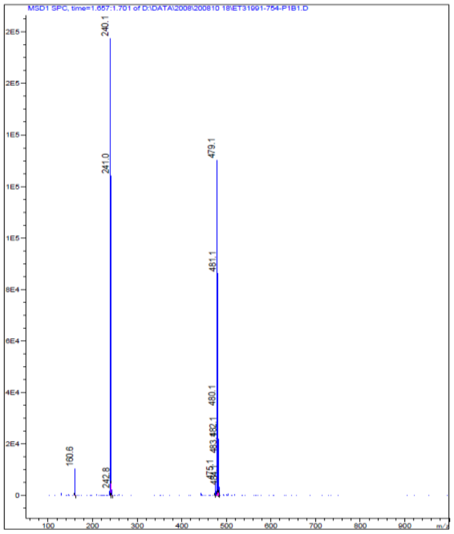
240.1
241.0
479.1
481.1
480.1
160.6
242.8

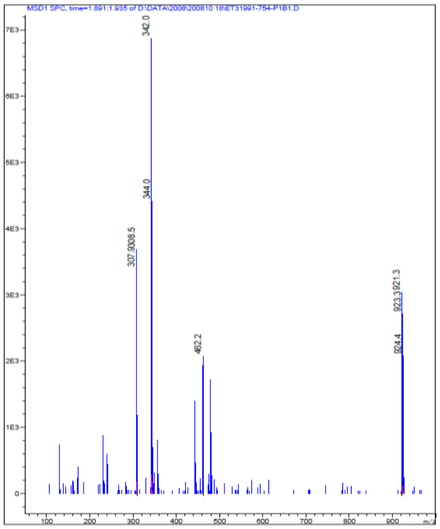
342.0
344.0
307.9308.5
462.2
923.3921.3
924.4

Compound 65

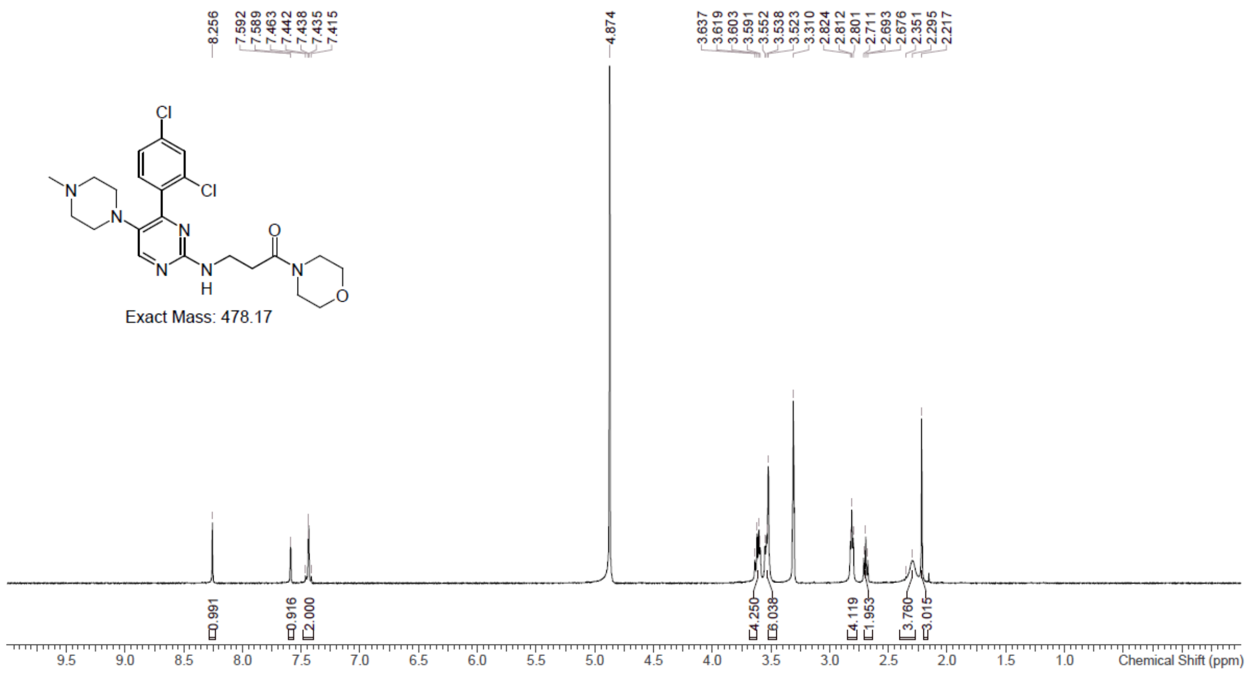

Exact Mass: 478.17
Chemical Shift (ppm)

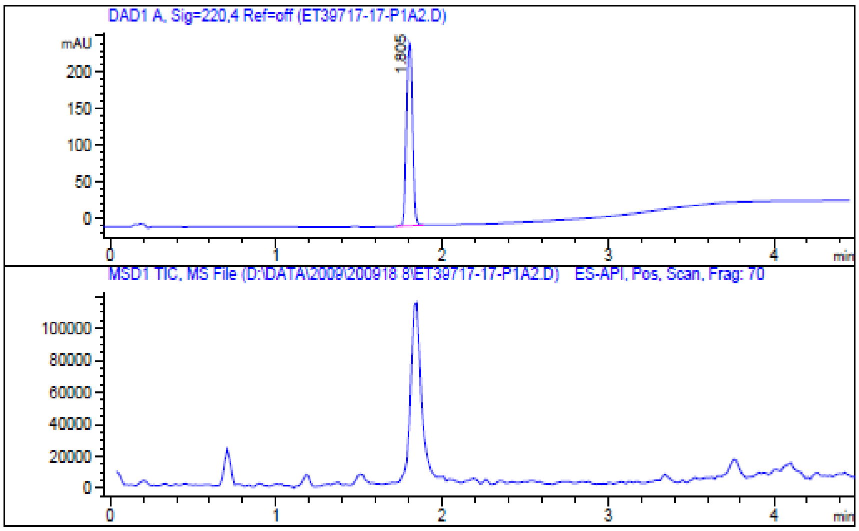

DAD1 A, Sig=220,4 Ref=off (ET39717-17-P1A2.D)
mAU
1.805
MSD1 TIC, MS File (D:\DATA\2009\200918 8\ET39717-17-P1A2.D) ES-API, Pos, Scan, Frag: 70
min

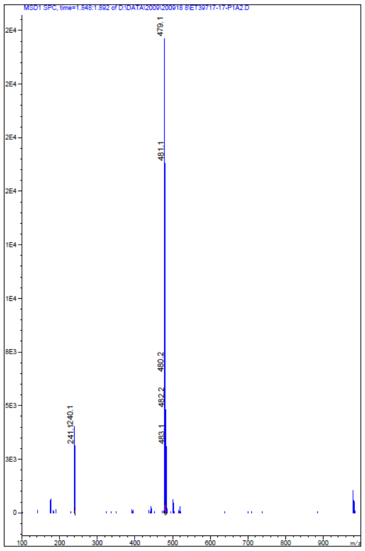

479.1
481.1
241.1
240.1

Compound 66

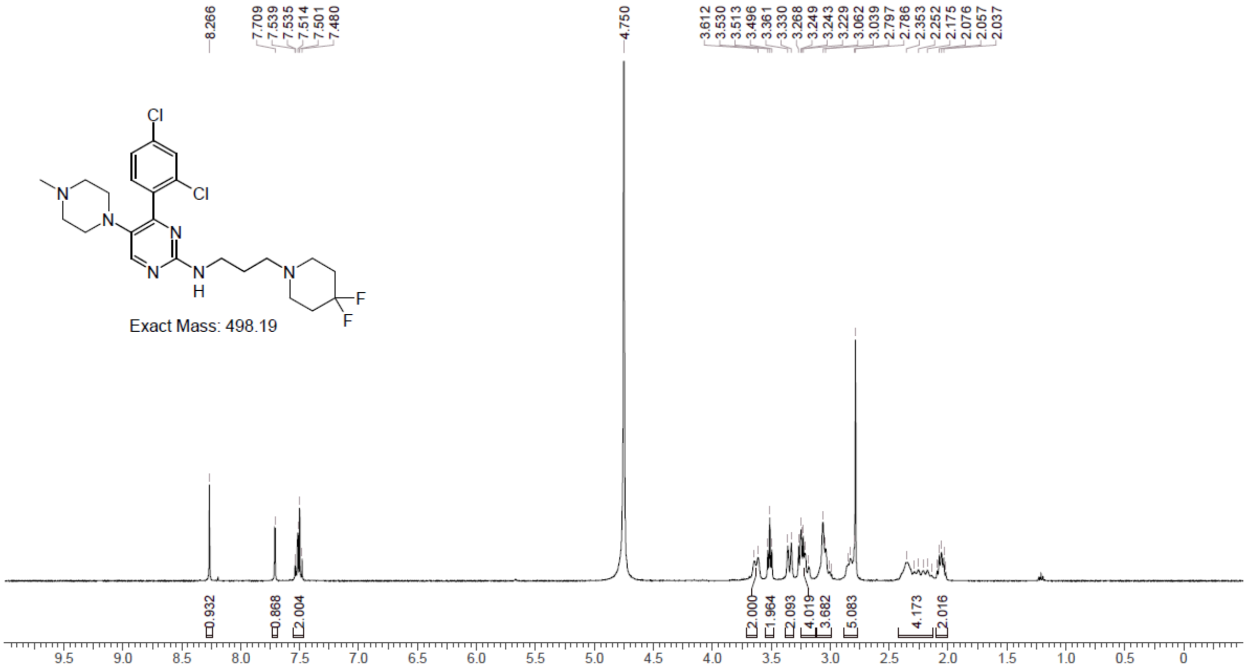

Cl
Cl
N
N
N
N
N
H
N
F
F
Exact Mass: 498.19

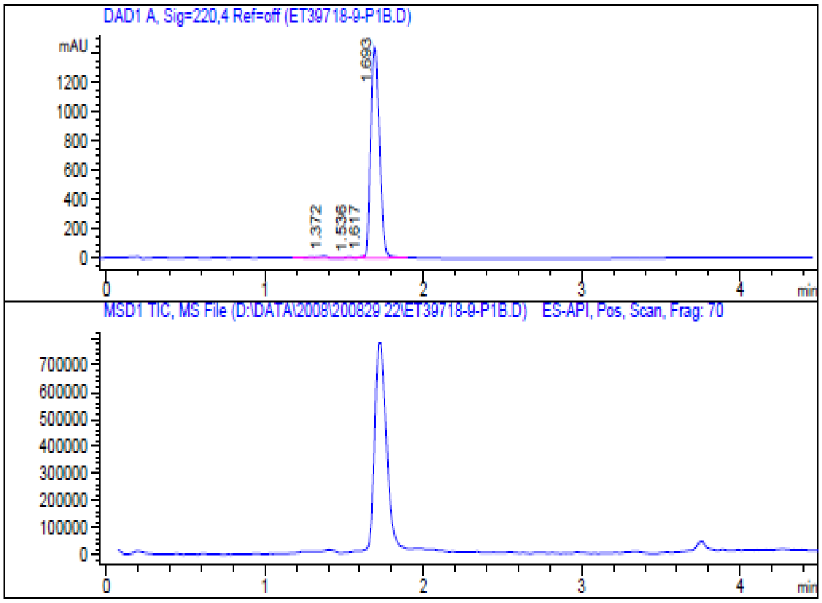

DAD1 A, Sig=220,4 Ref=off (ET39718-9-P1B.D)
mAU
1.693
1.372
1.536
1.617
min
MSD1 TIC, MS File (D:\DATA\2008\200829 22\ET39718-9-P1B.D) ES-API, Pos, Scan, Frag: 70

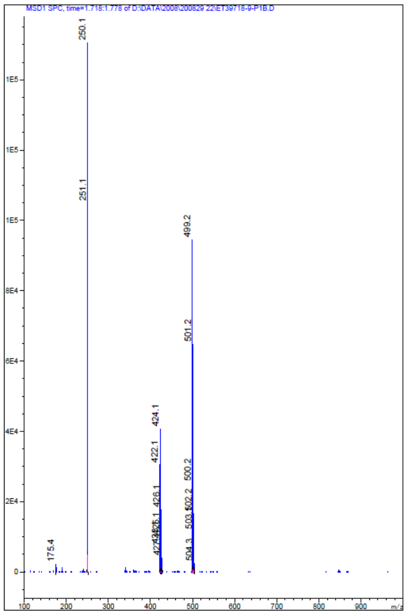

250.1
251.1
499.2
501.2
424.1
422.1
426.1
500.2
502.2
175.4

Compound 67

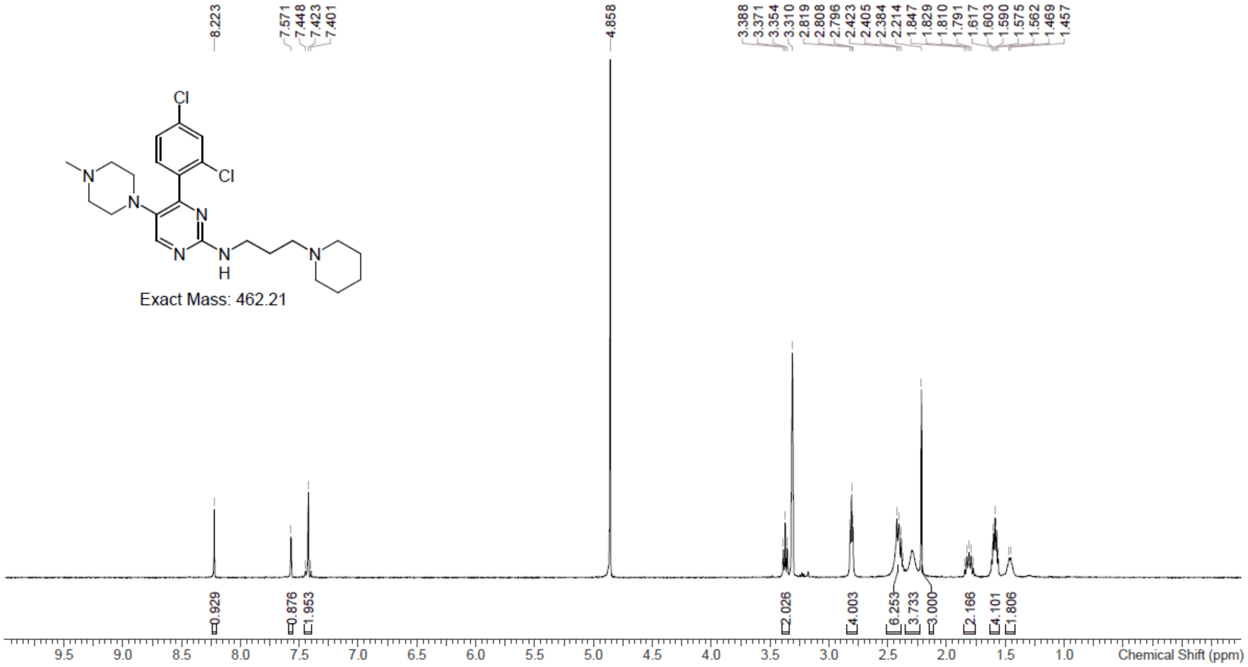

Exact Mass: 462.21
Chemical Shift (ppm)

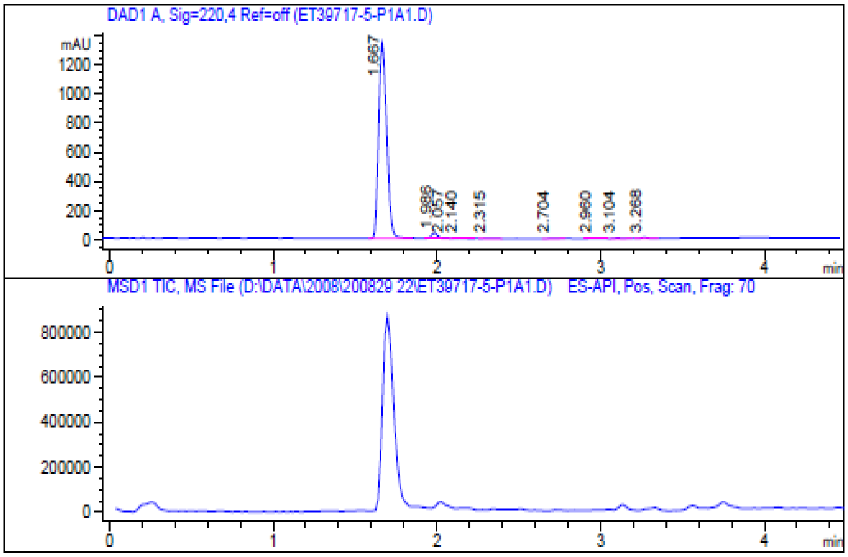

DAD1 A, Sig=220,4 Ref=off (ET39717-5-P1A1.D)
mAU
MSD1 TIC, MS File (D:\DATA\2008\200829 22\ET39717-5-P1A1.D)   ES-API, Pos, Scan, Frag: 70

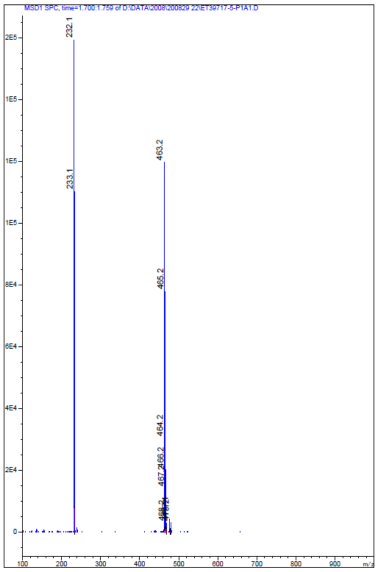

Compound 68

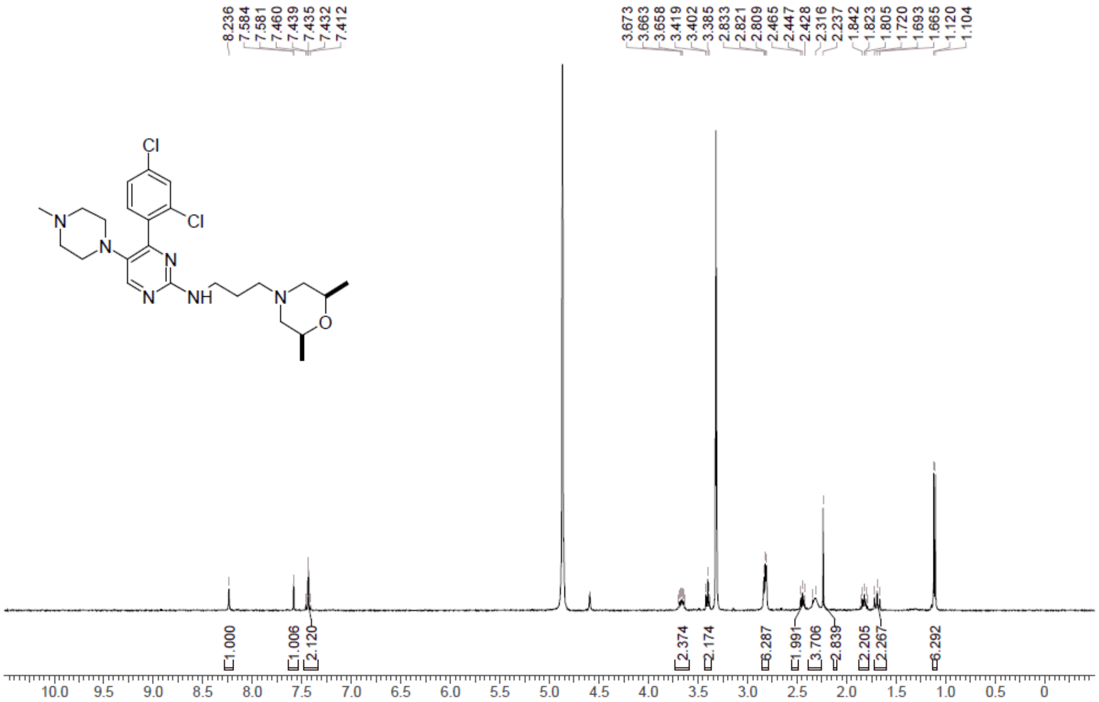

8.236
7.584
7.581
7.460
7.439
7.435
7.432
7.412
3.673
3.663
3.658
3.419
3.402
3.385
2.833
2.821
2.809
2.465
2.447
2.428
2.316
2.237
1.842
1.823
1.805
1.720
1.693
1.665
1.120
1.104
Cl
Cl
N
N
N
N
NH
N
O
1.000
1.006
2.120
2.374
2.174
6.287
1.991
3.706
2.839
2.205
2.267
6.292
10.0 9.5 9.0 8.5 8.0 7.5 7.0 6.5 6.0 5.5 5.0 4.5 4.0 3.5 3.0 2.5 2.0 1.5 1.0 0.5 0

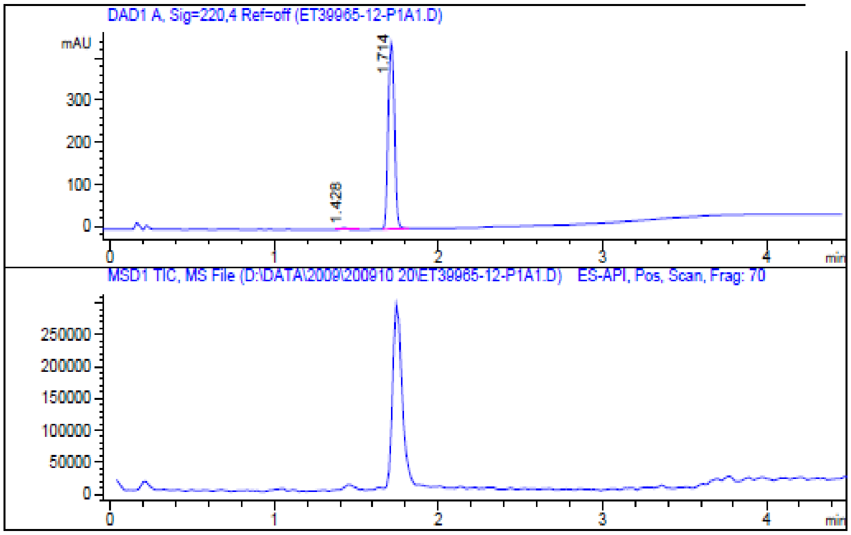

DAD1 A, Sig=220,4 Ref=off (ET39965-12-P1A1.D)
mAU
1.714
1.428
min
MSD1 TIC, MS File (D:\DATA\2009\200910 20\ET39965-12-P1A1.D) ES-API, Pos, Scan, Frag: 70
min

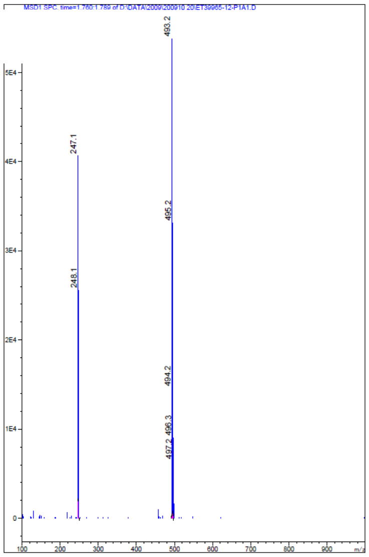

493.2
247.1
495.2
248.1
494.2
497.2
496.3

Compound 69

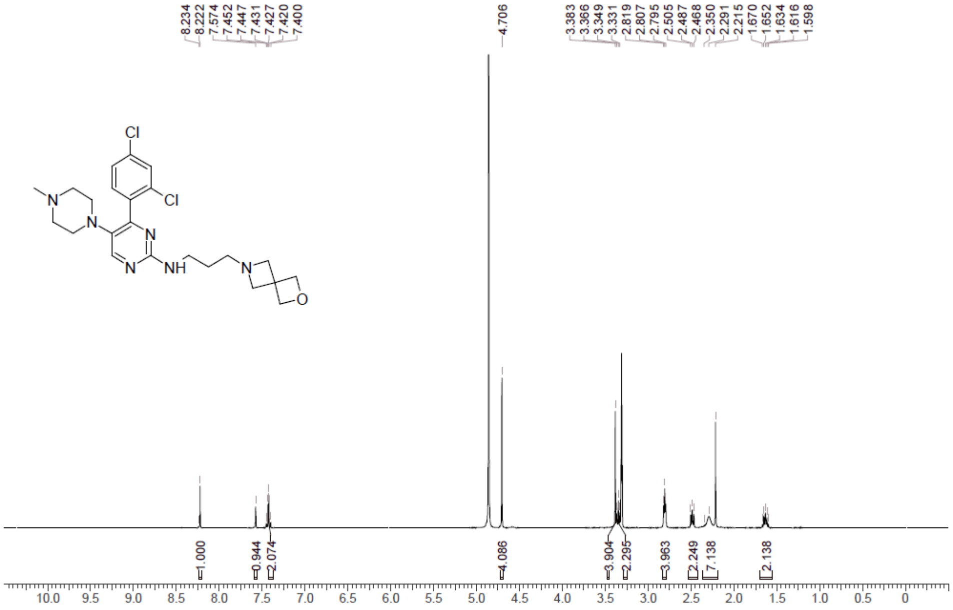

8.234
8.222
7.574
7.452
7.447
7.431
7.427
7.420
7.400
4.706
3.383
3.366
3.349
3.331
2.819
2.807
2.795
2.505
2.487
2.468
2.350
2.291
2.215
1.670
1.652
1.634
1.616
1.598
Cl
Cl
N
N
N
N
NH
N
O
1.000
0.944
2.074
4.086
3.904
2.295
3.963
2.249
7.138
2.138
10.0 9.5 9.0 8.5 8.0 7.5 7.0 6.5 6.0 5.5 5.0 4.5 4.0 3.5 3.0 2.5 2.0 1.5 1.0 0.5 0

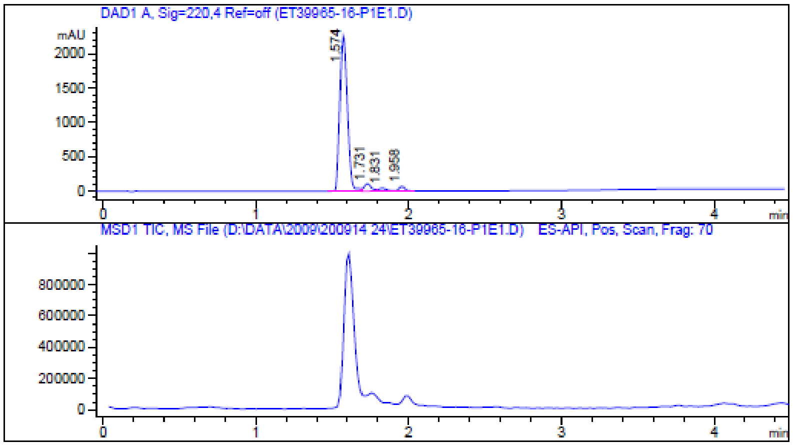

DAD1 A, Sig=220,4 Ref=off (ET39965-16-P1E1.D)
mAU
1.574
1.731
1.831
1.958
min
MSD1 TIC, MS File (D:\DATA\2009\200914 24\ET39965-16-P1E1.D) ES-API, Pos, Scan, Frag: 70
min

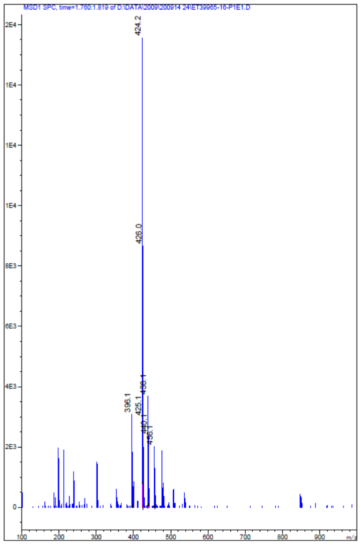

424.2
426.0

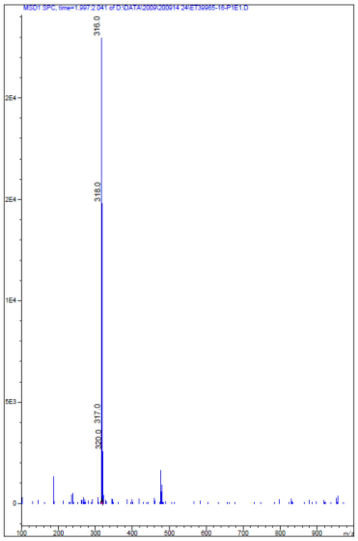

316.0
318.0

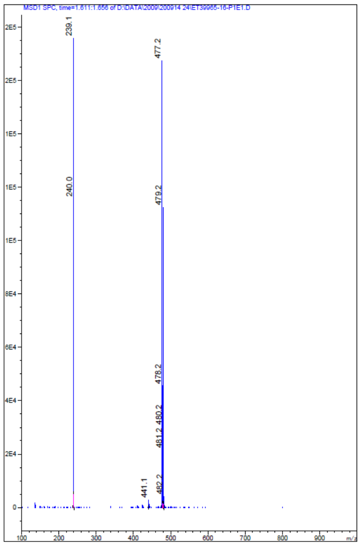

239.1
240.0
477.2
479.2

Compound 70

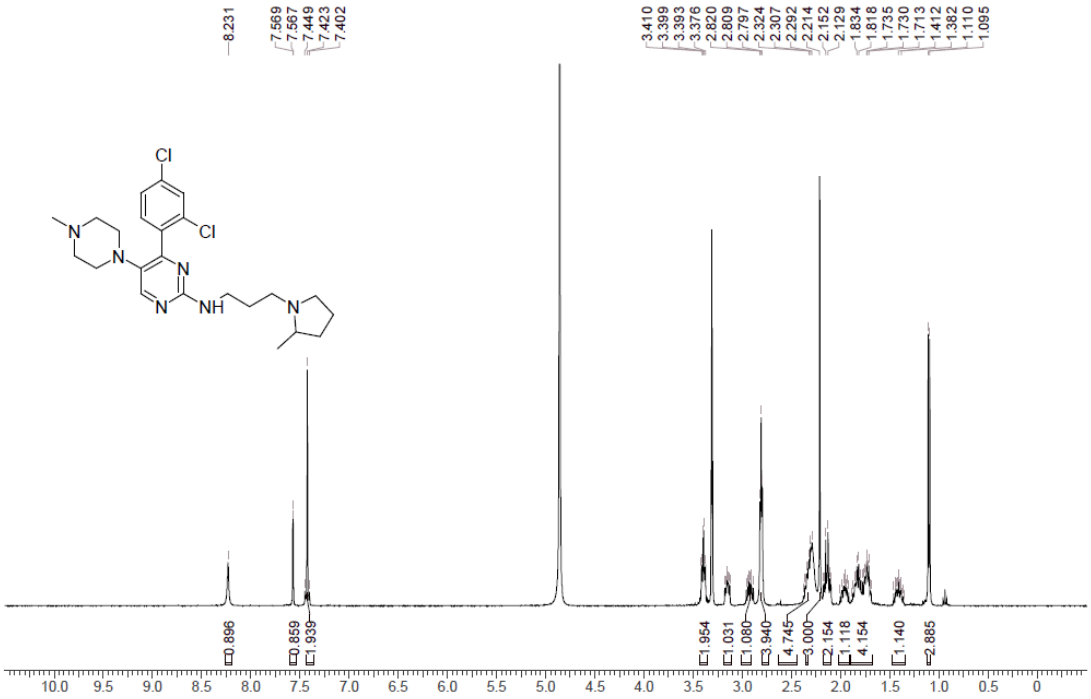

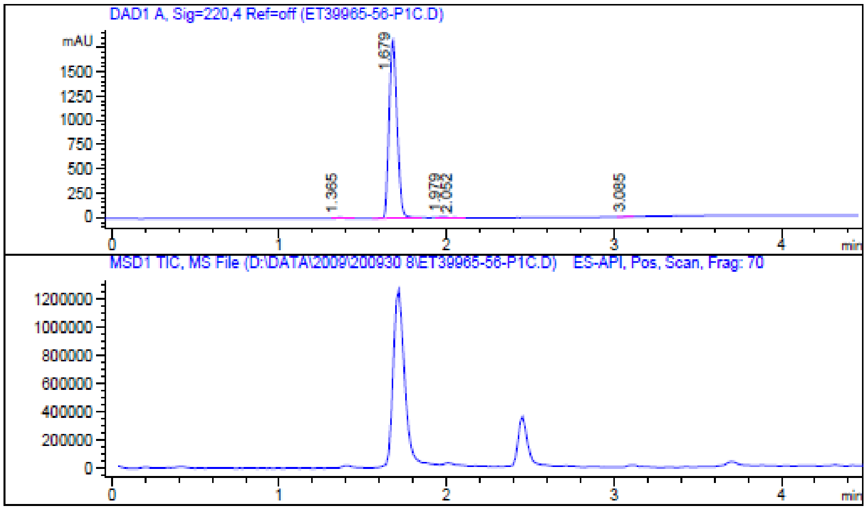
DAD1 A, Sig=220,4 Ref=off (ET39965-56-P1C.D)
mAU
1.679
1.365
1.979
2.052
3.085
min
MSD1 TIC, MS File (D:\DATA\2009\200930 8\ET39965-56-P1C.D) ES-API, Pos, Scan, Frag: 70
min

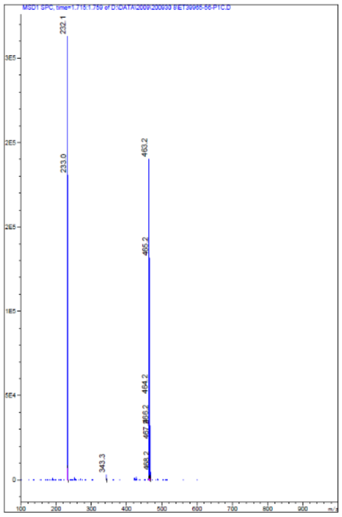

Compound 71

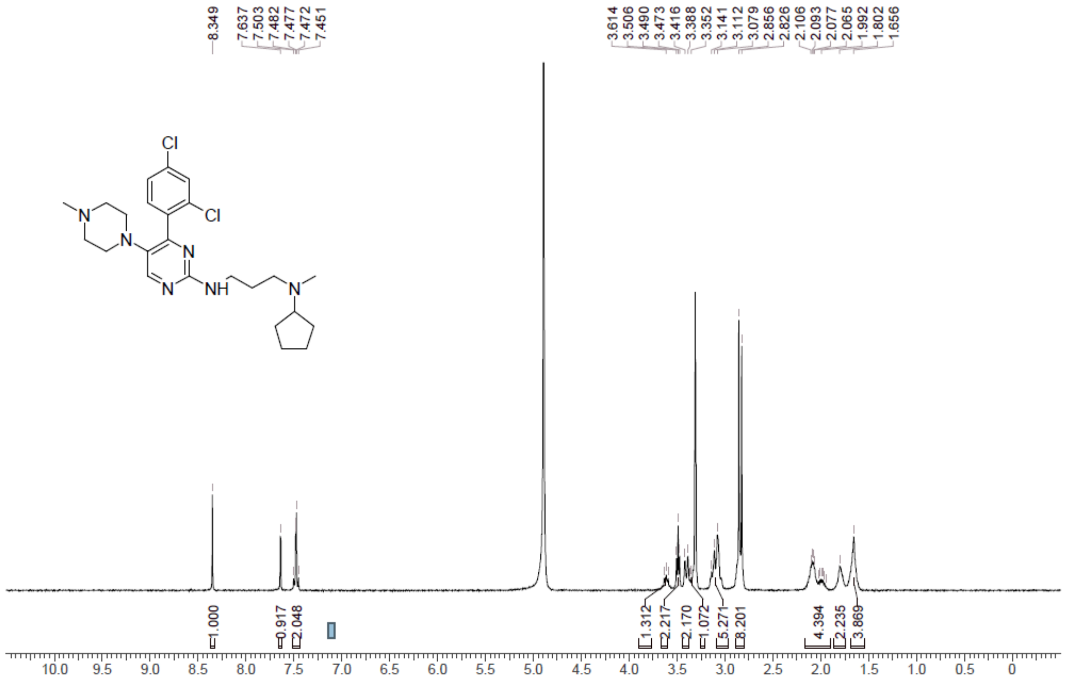

8.349
7.637
7.503
7.482
7.477
7.472
7.451
3.614
3.506
3.490
3.473
3.416
3.388
3.352
3.141
3.112
3.079
2.856
2.826
2.106
2.093
2.077
2.065
1.992
1.802
1.656
Cl
Cl
N
N
N
N
NH
N
1.000
0.917
2.048
1.312
2.217
2.170
1.072
5.271
8.201
4.394
2.235
3.869
10.0
9.5
9.0
8.5
8.0
7.5
7.0
6.5
6.0
5.5
5.0
4.5
4.0
3.5
3.0
2.5
2.0
1.5
1.0
0.5
0

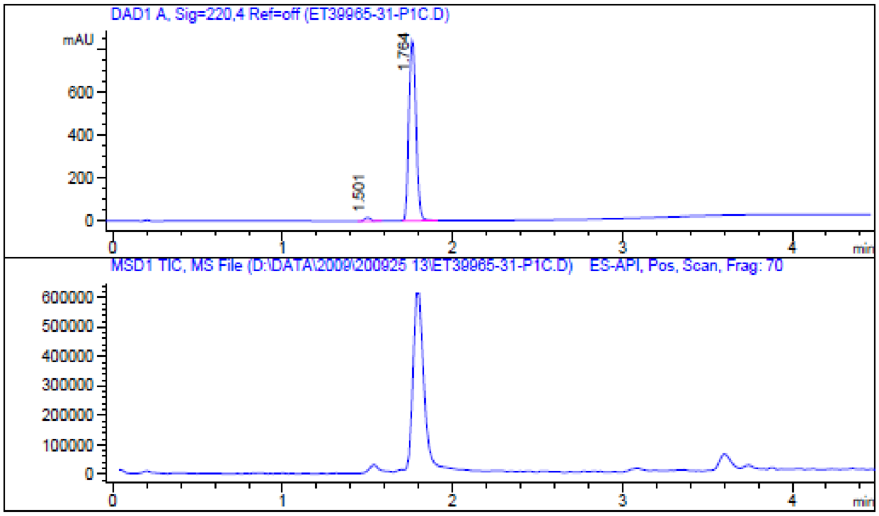

DAD1 A, Sig=220,4 Ref=off (ET39965-31-P1C.D)
mAU
600
400
200
0
1.764
1.501
0
1
2
3
4
min
MSD1 TIC, MS File (D:\DATA\2009\200925 13\ET39965-31-P1C.D) ES-API, Pos, Scan, Frag: 70
600000
500000
400000
300000
200000
100000
0
0
1
2
3
4
min

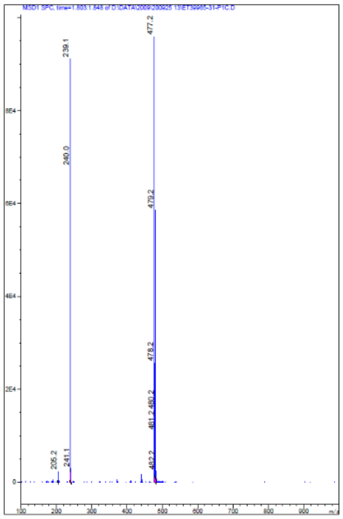

477.2
239.1
240.0
479.2
478.2
480.2
205.2
241.1

Compound 72

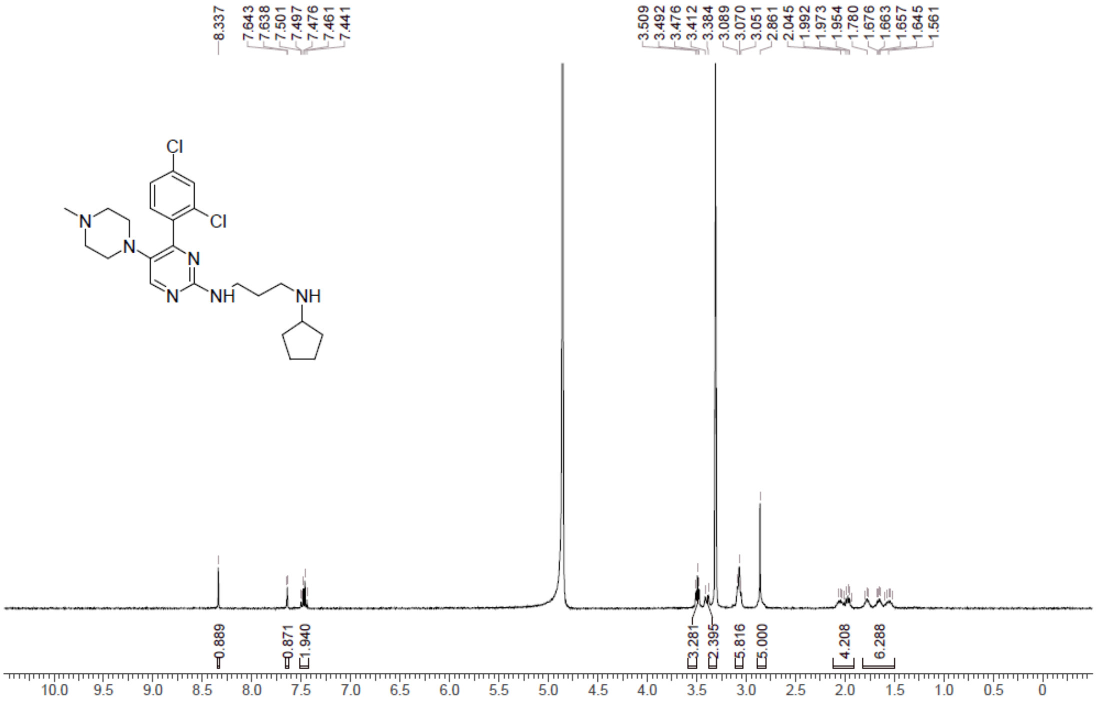

Cl
Cl
N
N
N
N
NH
NH
8.337
7.643
7.638
7.501
7.497
7.476
7.461
7.441
3.509
3.492
3.476
3.412
3.384
3.089
3.070
3.051
2.861
2.045
1.992
1.973
1.954
1.780
1.676
1.663
1.657
1.645
1.561
0.889
0.871
1.940
3.281
2.395
5.816
5.000
4.208
6.288
10.0 9.5 9.0 8.5 8.0 7.5 7.0 6.5 6.0 5.5 5.0 4.5 4.0 3.5 3.0 2.5 2.0 1.5 1.0 0.5 0

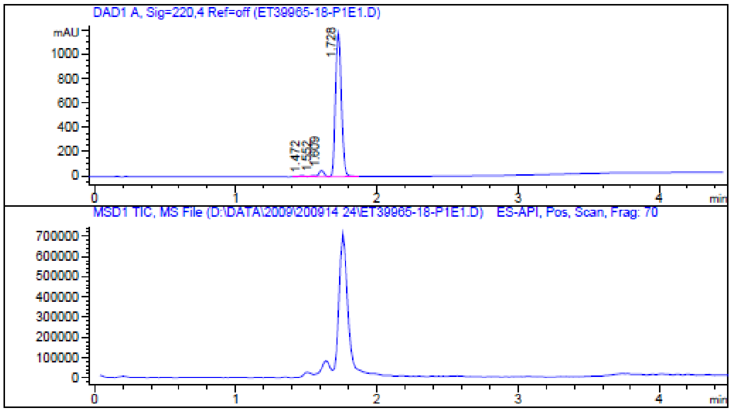

DAD1 A, Sig=220,4 Ref=off (ET39965-18-P1E1.D)
mAU
1.728
1.472
MSD1 TIC, MS File (D:\DATA\2009\200914 24\ET39965-18-P1E1.D) ES-API, Pos, Scan, Frag: 70
min

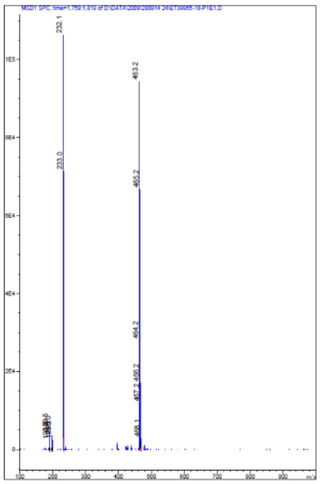

232.1
233.0
463.2
465.2

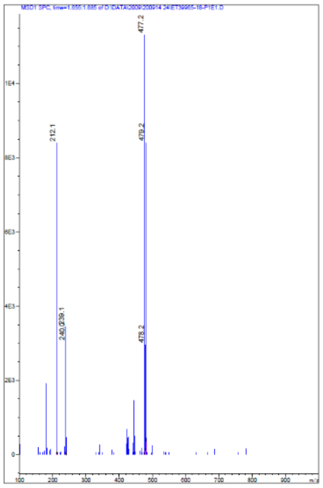

477.2
212.1
479.2
478.2

Compound 73

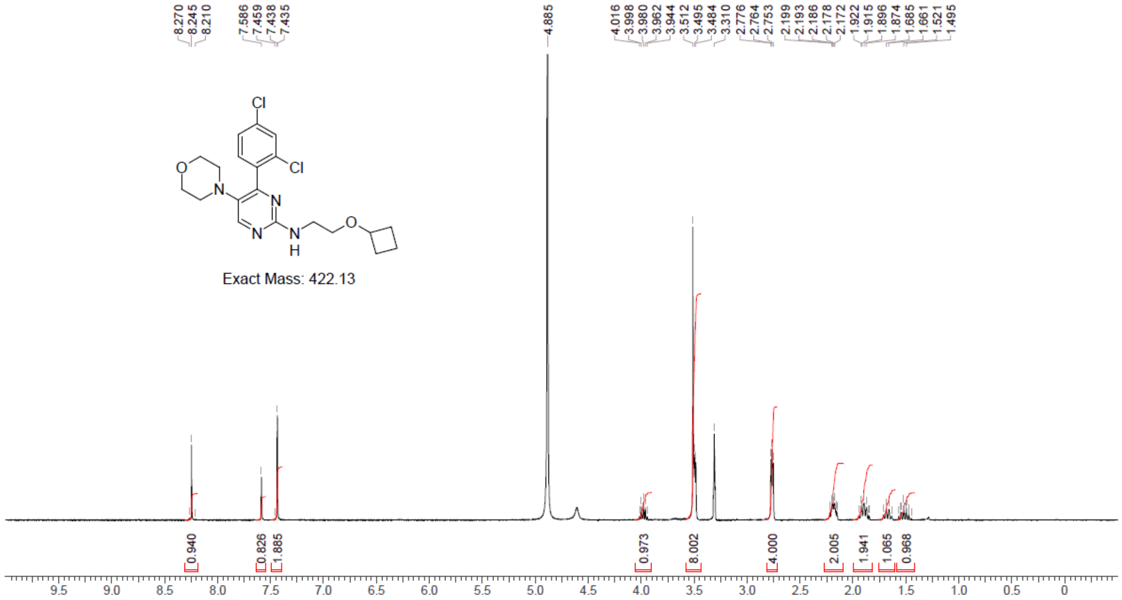

Cl
Cl
O
N
N
N
N
H
O
Exact Mass: 422.13

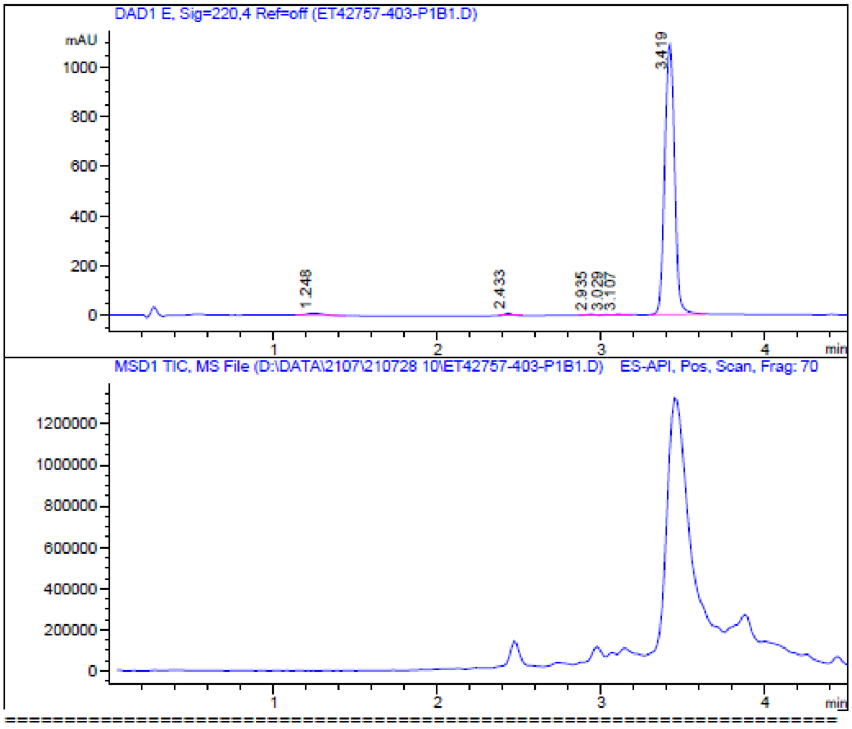

DAD1 E, Sig=220,4 Ref=off (ET42757-403-P1B1.D)
mAU
3.419
1.248
2.433
2.935
3.029
3.107
MSD1 TIC, MS File (D:\DATA\2107\210728 10\ET42757-403-P1B1.D) ES-API, Pos, Scan, Frag: 70

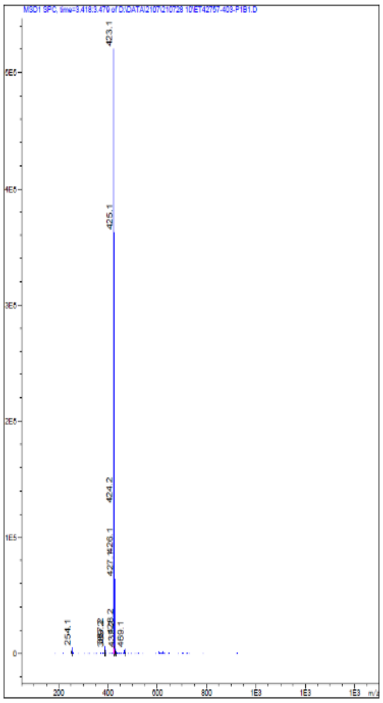

423.1
425.1
424.2
426.1
254.1

Compound 74

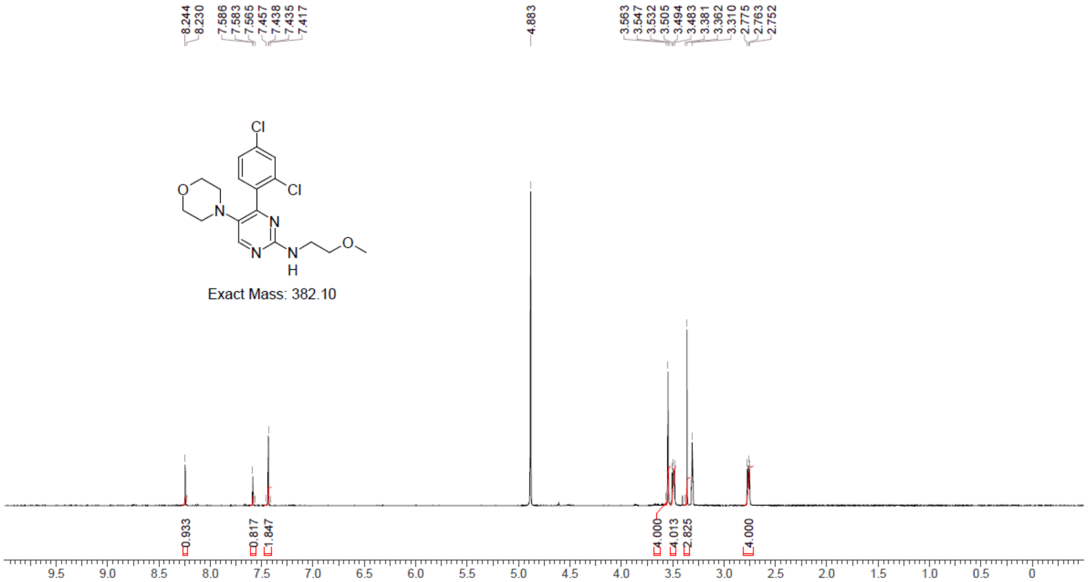
Exact Mass: 382.10

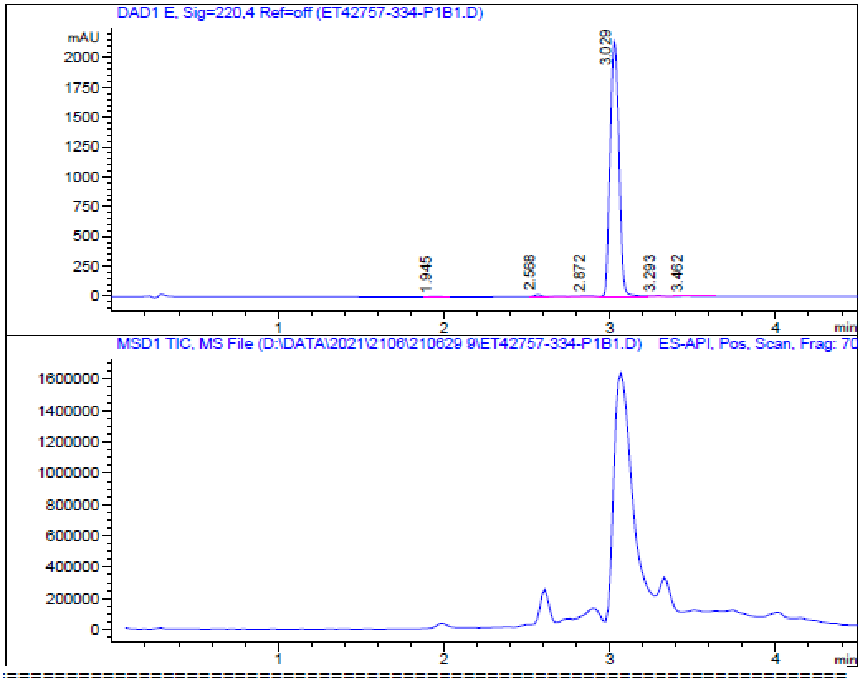
DAD1 E, Sig=220,4 Ref=off (ET42757-334-P1B1.D)
MSD1 TIC, MS File (D:\DATA\2021\2106\210629 9\ET42757-334-P1B1.D) ES-API, Pos, Scan, Frag: 70

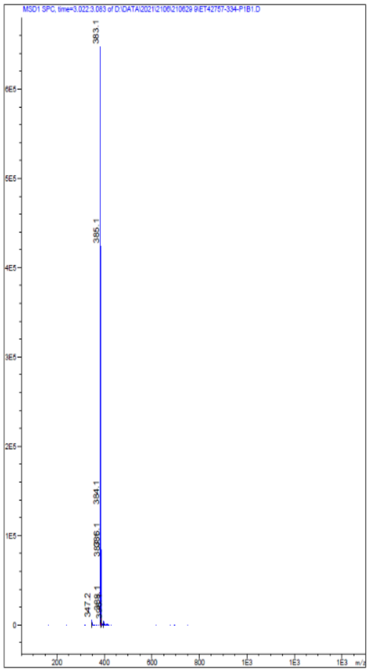

Compound 75

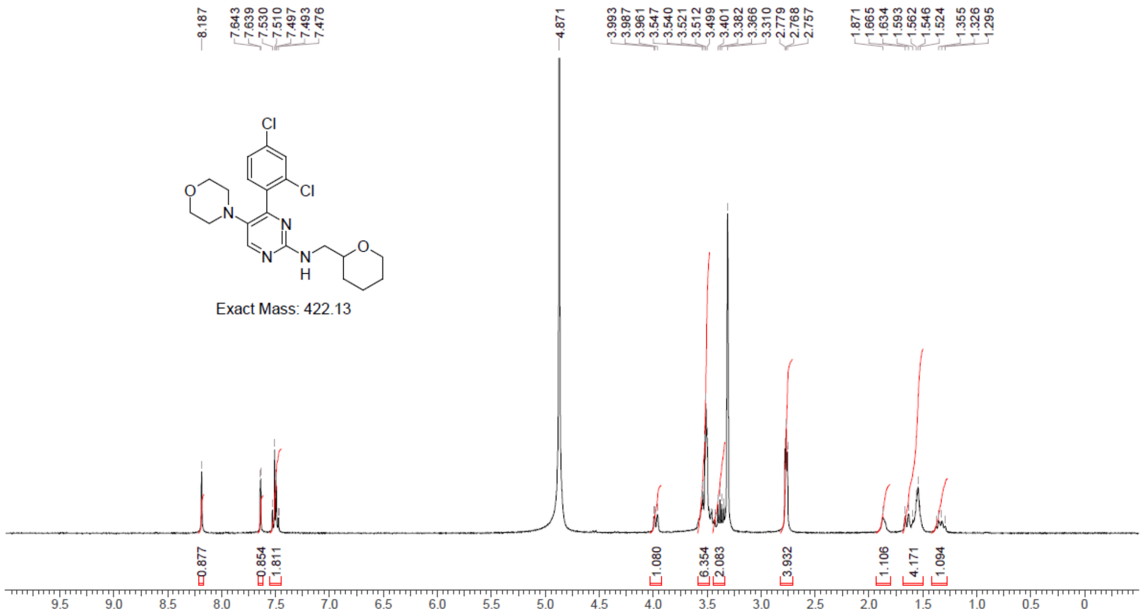

8.187
7.643
7.639
7.530
7.510
7.497
7.493
7.476
4.871
3.993
3.987
3.961
3.547
3.540
3.521
3.512
3.499
3.401
3.382
3.366
3.310
2.779
2.768
2.757
1.871
1.665
1.634
1.593
1.562
1.546
1.524
1.355
1.326
1.295
Cl
Cl
O
N
N
N
N
H
O
Exact Mass: 422.13
0.877
0.854
1.811
1.080
6.354
2.083
3.932
1.106
4.171
1.094
9.5
9.0
8.5
8.0
7.5
7.0
6.5
6.0
5.5
5.0
4.5
4.0
3.5
3.0
2.5
2.0
1.5
1.0
0.5
0

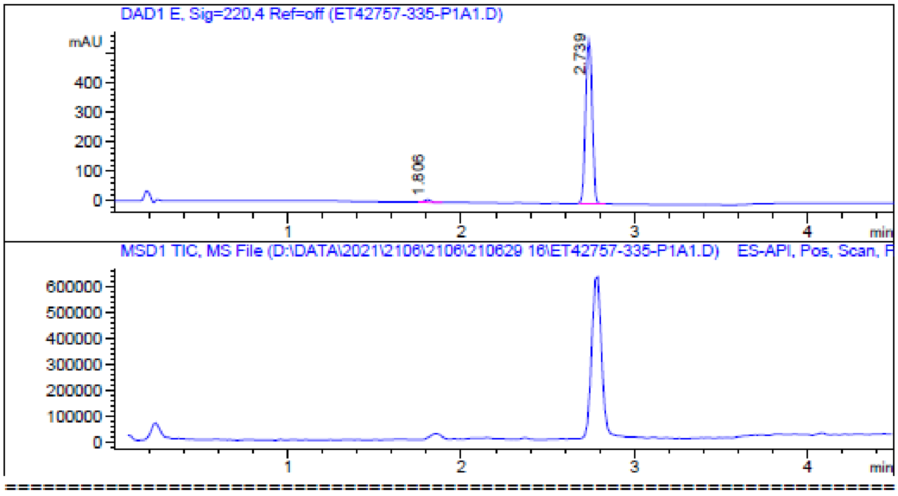

DAD1 E, Sig=220,4 Ref=off (ET42757-335-P1A1.D)
mAU
400
300
200
100
0
2.739
1.806
1
2
3
4
min
MSD1 TIC, MS File (D:\DATA\2021\2106\2106\210629 16\ET42757-335-P1A1.D) ES-API, Pos, Scan, F
600000
500000
400000
300000
200000
100000
0
1
2
3
4
min

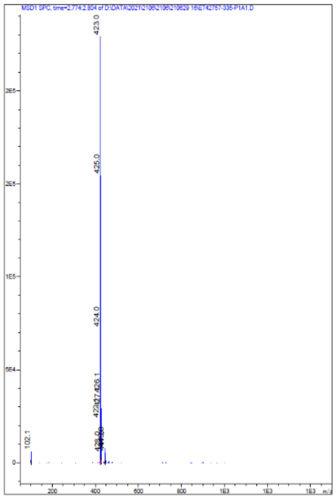

423.0
425.0
424.0
102.1

Compound 76

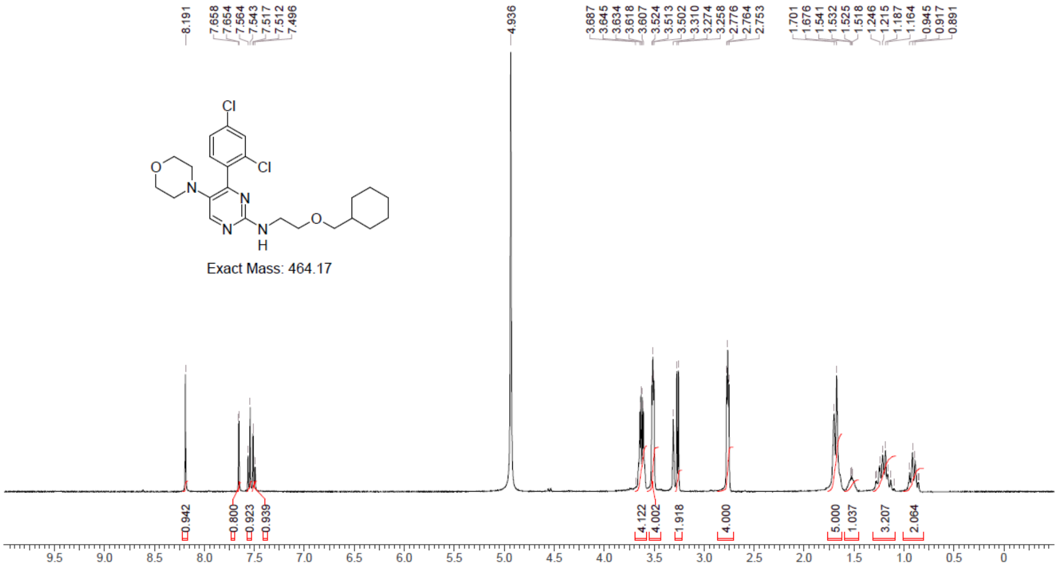
Exact Mass: 464.17

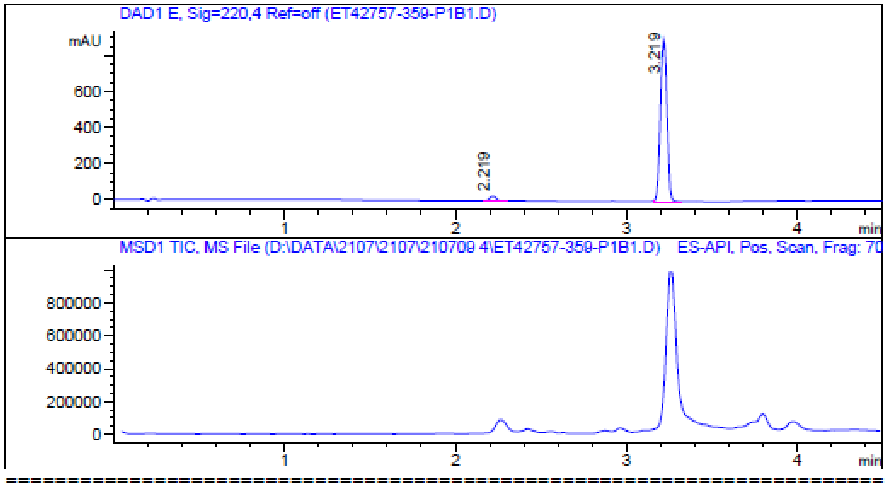
DAD1 E, Sig=220,4 Ref=off (ET42757-359-P1B1.D)
MSD1 TIC, MS File (D:\DATA\2107\2107\210709 4\ET42757-359-P1B1.D) ES-API, Pos, Scan, Frag: 70

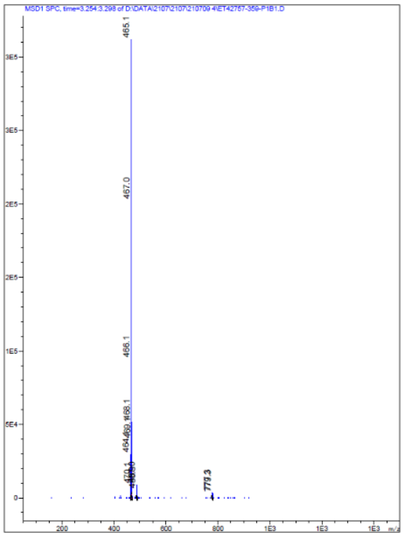

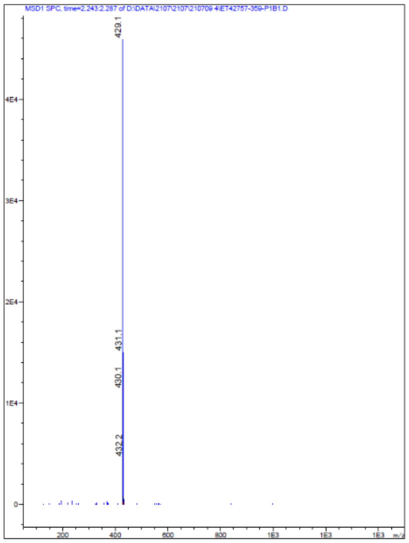

Compound 77

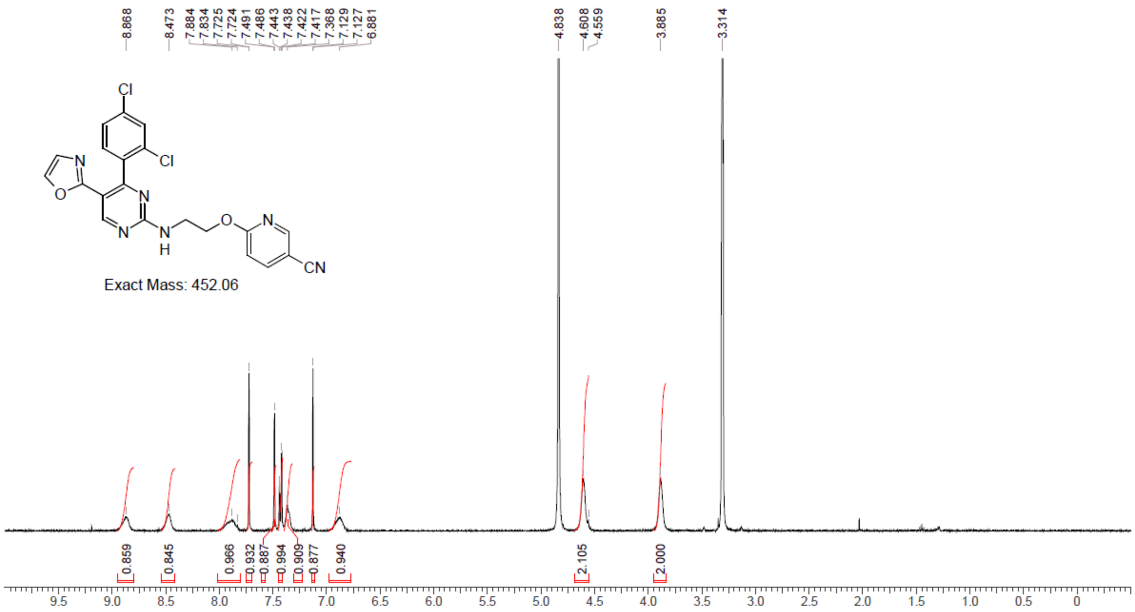

Exact Mass: 452.06

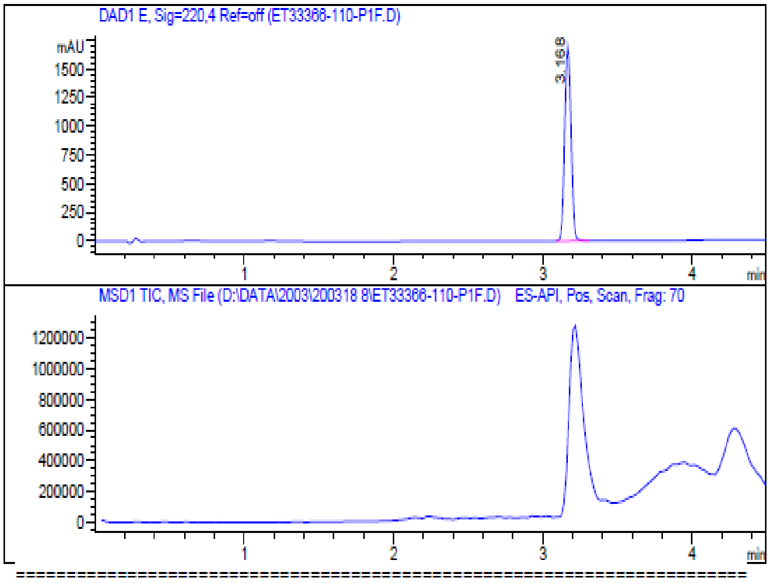

DAD1 E, Sig=220,4 Ref=off (ET33366-110-P1F.D)
MSD1 TIC, MS File (D:\DATA\2003\200318 8\ET33366-110-P1F.D) ES-API, Pos, Scan, Frag: 70

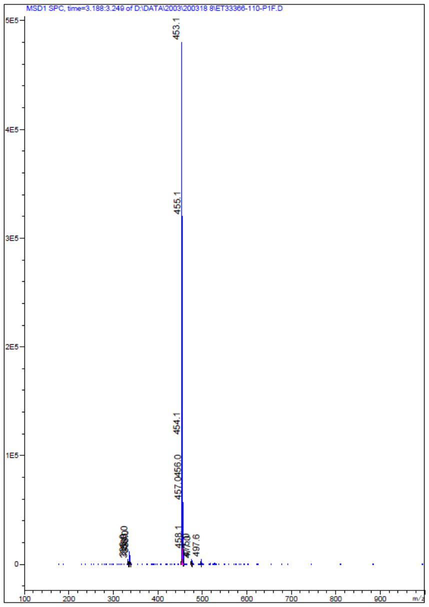

453.1
455.1
454.1

Compound 78

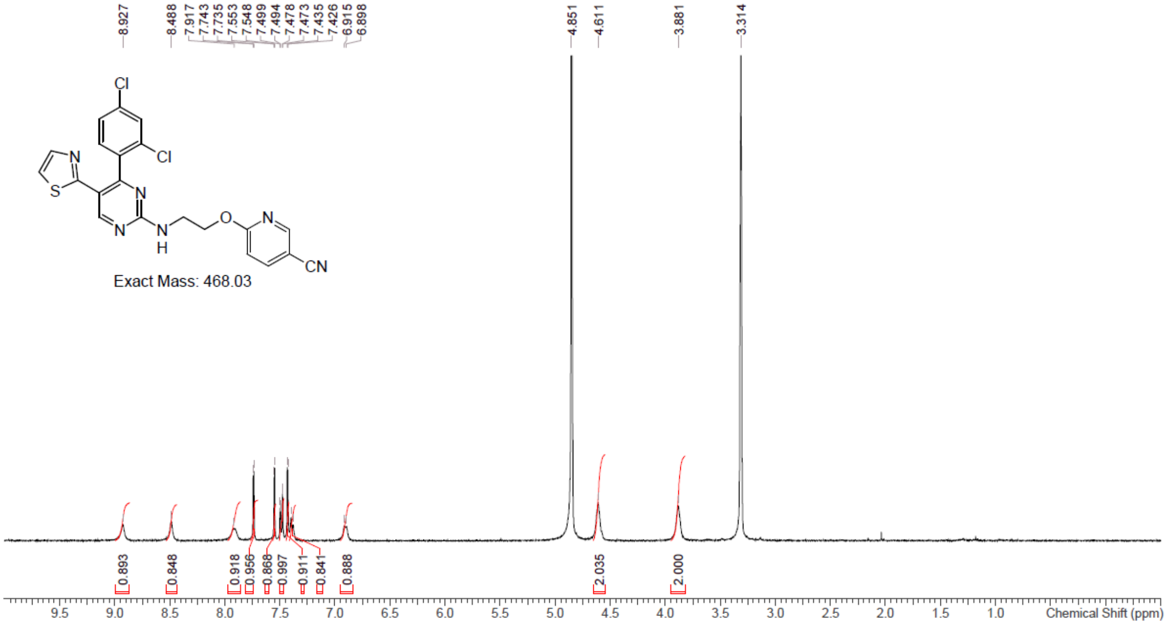
8.927
8.488
7.917
7.743
7.735
7.553
7.548
7.499
7.494
7.478
7.473
7.435
7.426
6.915
6.898
4.851
4.611
3.881
3.314
Cl
Cl
N
S
N
N
N
H
O
N
CN
Exact Mass: 468.03
0.893
0.848
0.918
0.956
0.868
0.997
0.911
0.841
0.888
2.035
2.000
Chemical Shift (ppm)

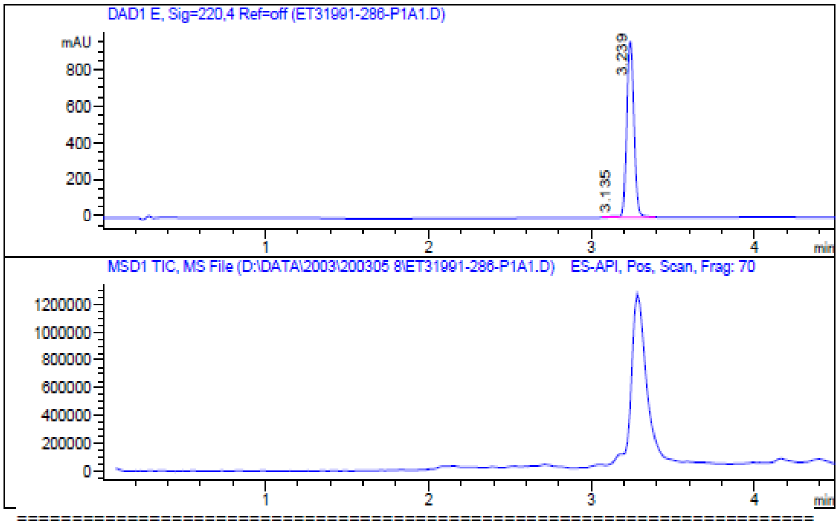
DAD1 E, Sig=220,4 Ref=off (ET31991-286-P1A1.D)
mAU
3.239
3.135
min
MSD1 TIC, MS File (D:\DATA\2003\200305 8\ET31991-286-P1A1.D) ES-API, Pos, Scan, Frag: 70
min

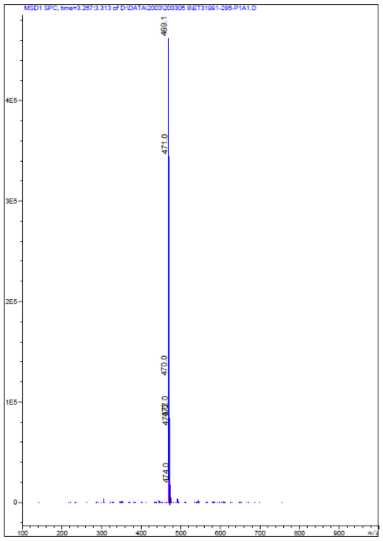
469.1
471.0
470.0
474.0

Compound 79

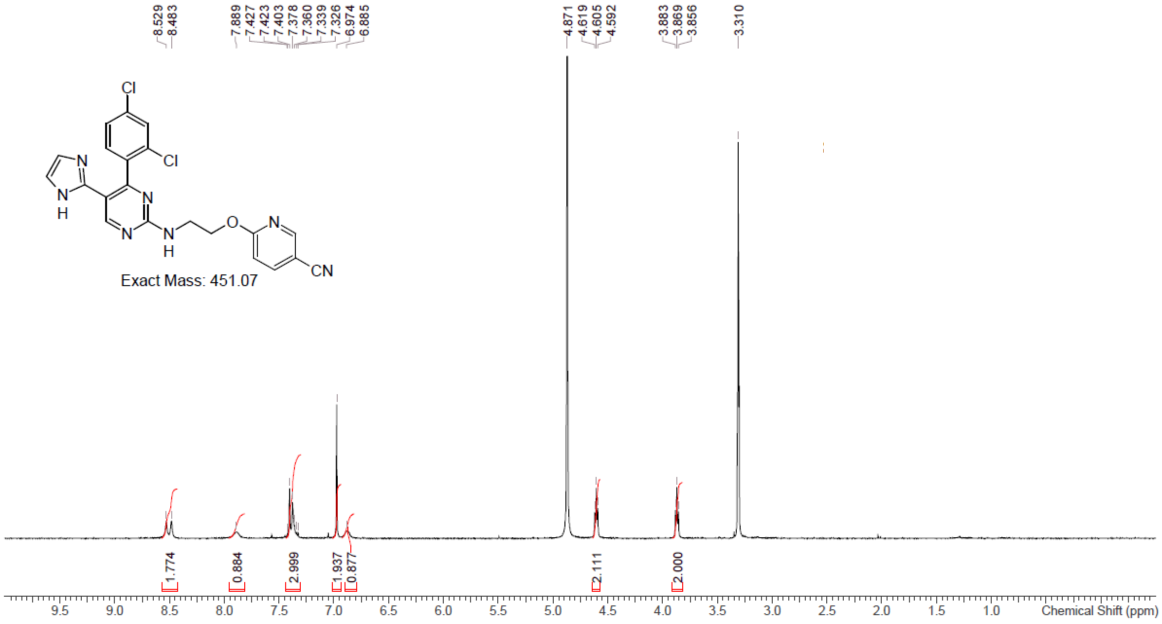
Exact Mass: 451.07
Chemical Shift (ppm)

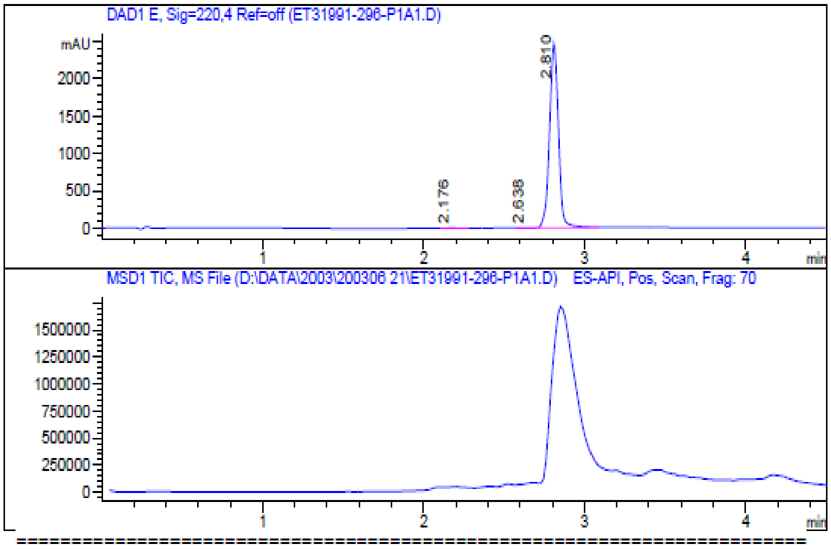
DAD1 E, Sig=220,4 Ref=off (ET31991-296-P1A1.D)
MSD1 TIC, MS File (D:\DATA\2003\200306 21\ET31991-296-P1A1.D) ES-API, Pos, Scan, Frag: 70

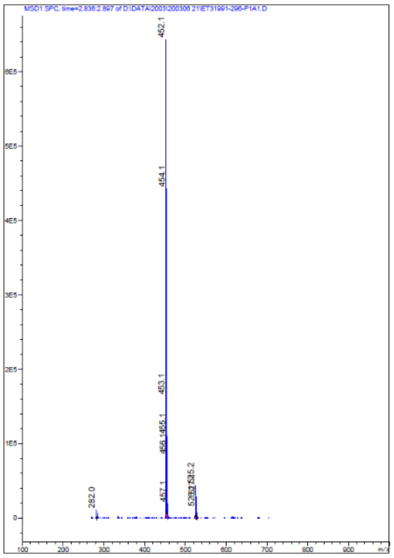

Compound 80

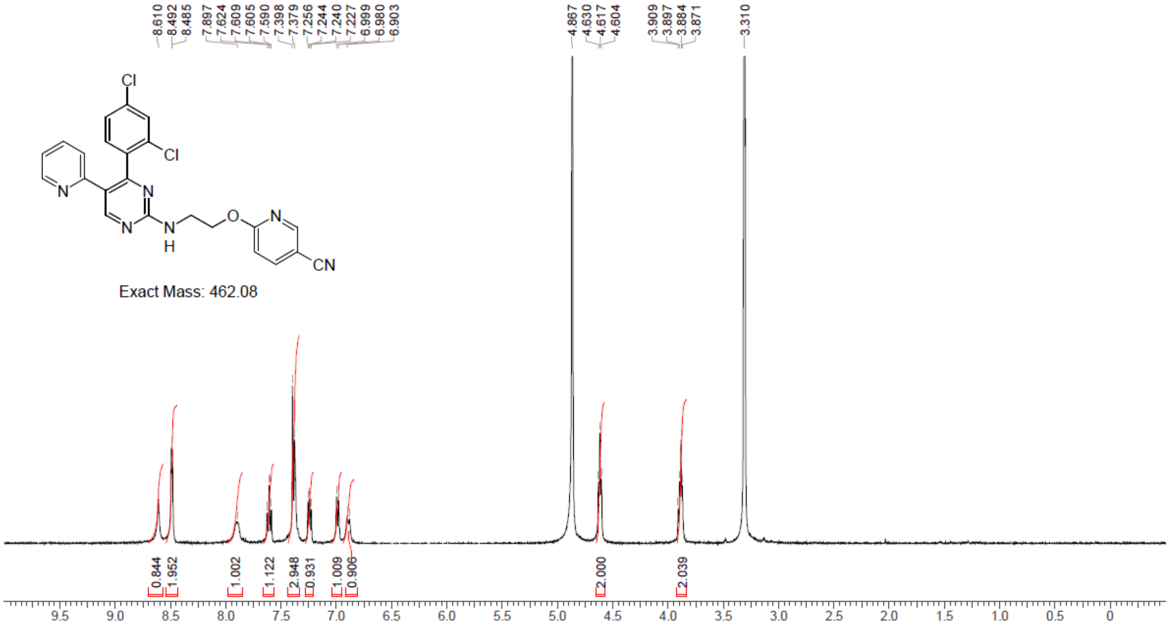
Cl
Cl
N
N
N
N
H
O
N
CN
Exact Mass: 462.08

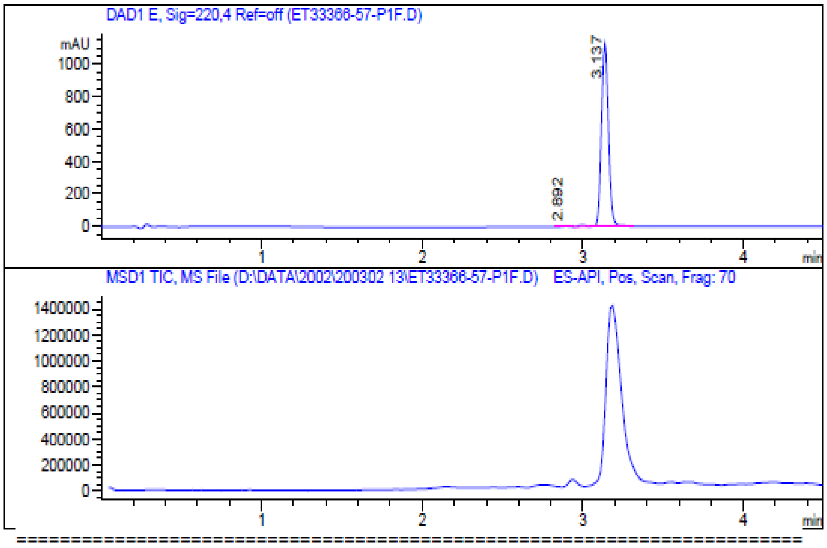
DAD1 E, Sig=220,4 Ref=off (ET33366-57-P1F.D)
MSD1 TIC, MS File (D:\DATA\2002\200302 13\ET33366-57-P1F.D) ES-API, Pos, Scan, Frag: 70

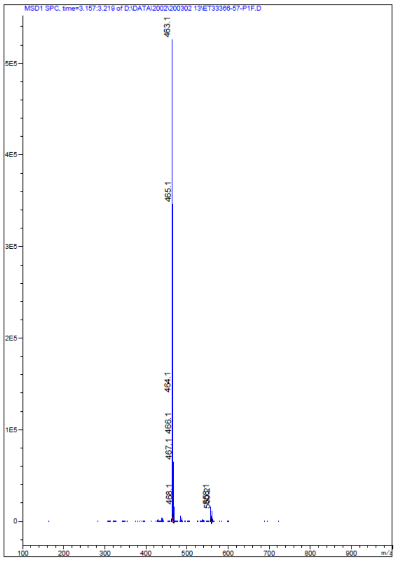

Compound 81

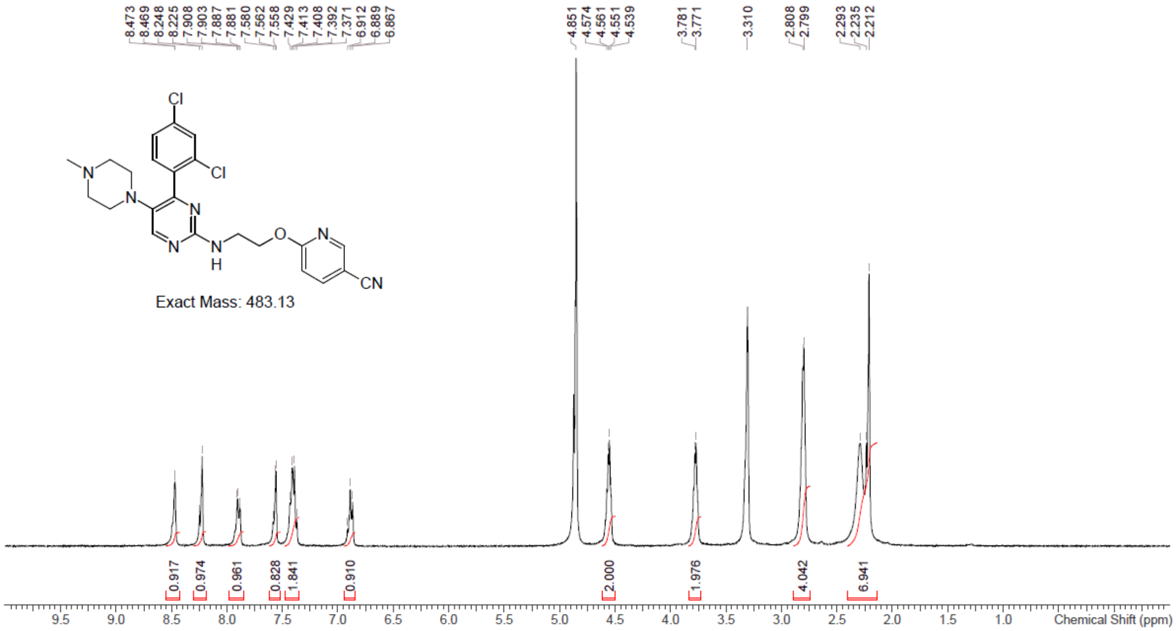
8.473 8.469 8.248 8.225 7.908 7.903 7.887 7.881 7.580 7.562 7.558 7.429 7.413 7.408 7.392 7.371 6.912 6.889 6.867
4.851 4.574 4.561 4.551 4.539
3.781 3.771
3.310
2.808 2.799
2.293 2.235 2.212
Cl
Cl
N
N
N
N
N
H
O
N
CN
Exact Mass: 483.13
0.917 0.974 0.961 0.828 1.841 0.910 2.000 1.976 4.042 6.941
Chemical Shift (ppm)

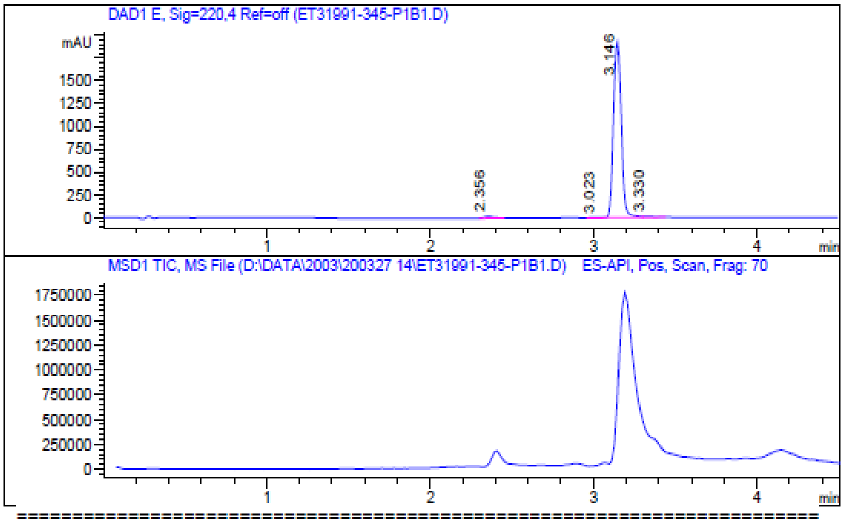
DAD1 E, Sig=220,4 Ref=off (ET31991-345-P1B1.D)
mAU
3.146
2.356
3.023
3.330
MSD1 TIC, MS File (D:\DATA\2003\200327 14\ET31991-345-P1B1.D) ES-API, Pos, Scan, Frag: 70

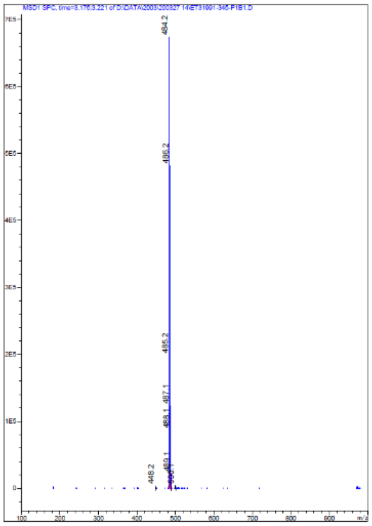
484.2
486.2

Compound 82

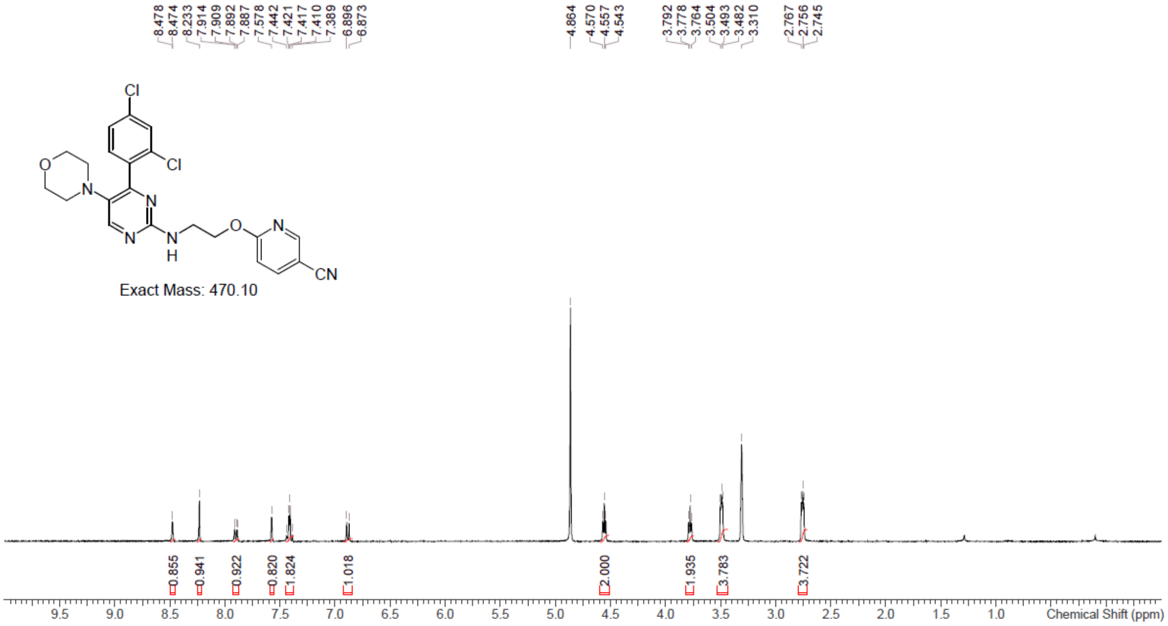

8.478
8.474
8.233
7.914
7.909
7.892
7.887
7.578
7.442
7.421
7.417
7.410
7.389
6.896
6.873
4.864
4.570
4.557
4.543
3.792
3.778
3.764
3.504
3.493
3.482
3.310
2.767
2.756
2.745
Exact Mass: 470.10
0.855
0.941
0.922
0.820
1.824
1.018
2.000
1.935
3.783
3.722
Chemical Shift (ppm)

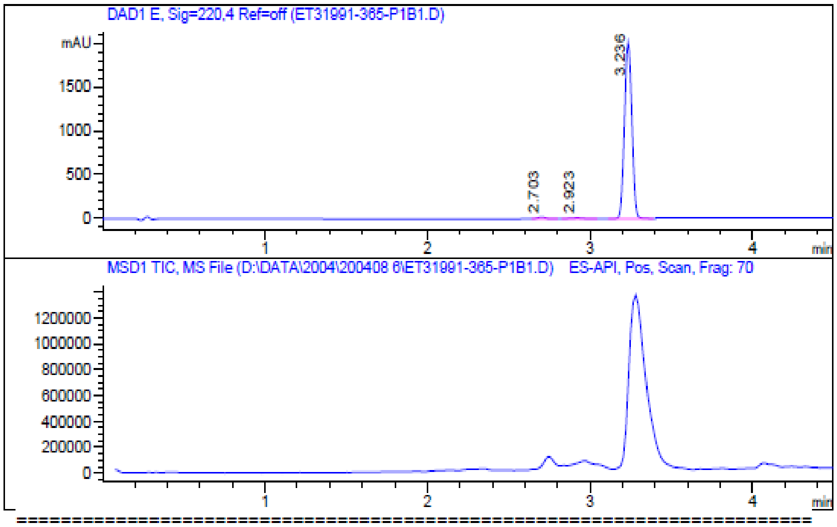

DAD1 E, Sig=220,4 Ref=off (ET31991-365-P1B1.D)
mAU
3.236
2.703
2.923
MSD1 TIC, MS File (D:\DATA\2004\200408 6\ET31991-365-P1B1.D) ES-API, Pos, Scan, Frag: 70
min

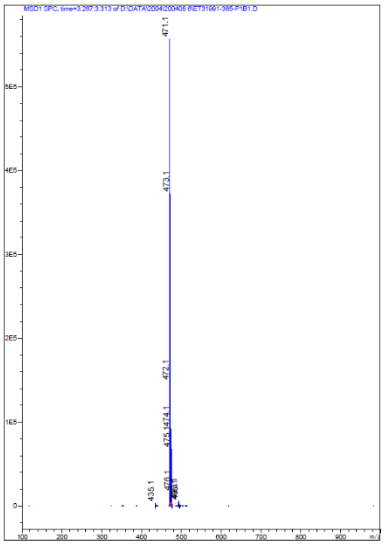

471.1
473.1
472.1
435.1

Compound 83

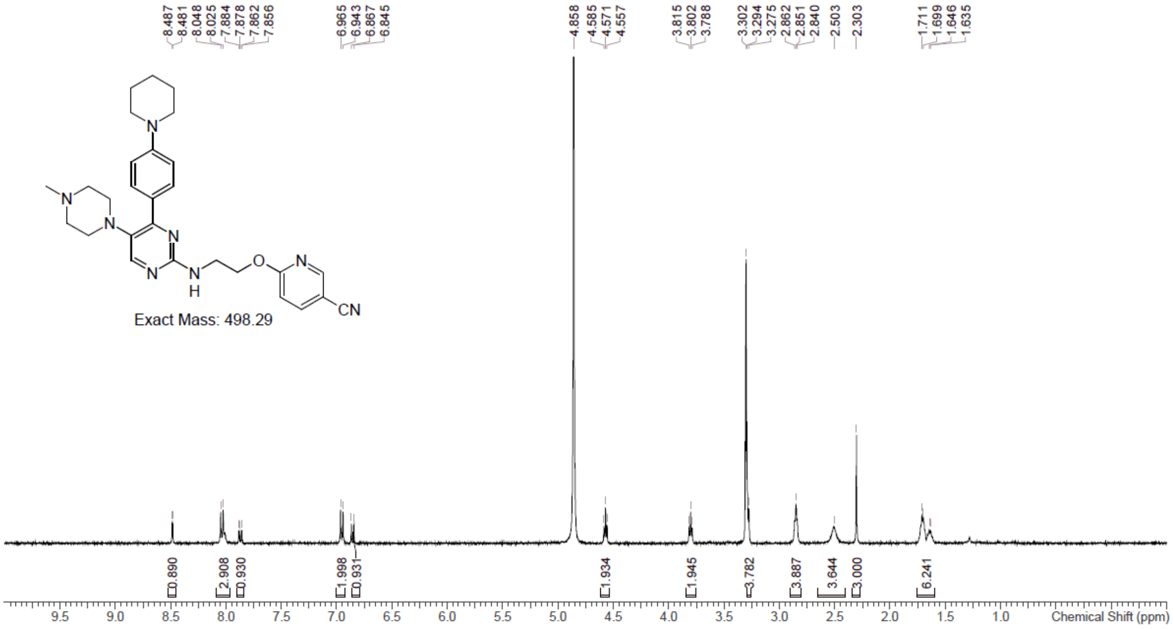

8.487 8.481 8.048 8.025 7.884 7.878 7.862 7.856
6.965 6.943 6.867 6.845
4.858 4.585 4.571 4.557
3.815 3.802 3.788
3.302 3.294 3.275 2.862 2.851 2.840 2.503 2.303
1.711 1.699 1.646 1.635
N
N
N
N
N
N
H
O
CN
Exact Mass: 498.29
0.890 2.908 0.930 1.998 0.931 1.934 1.945 3.782 3.887 3.644 3.000 6.241
Chemical Shift (ppm)

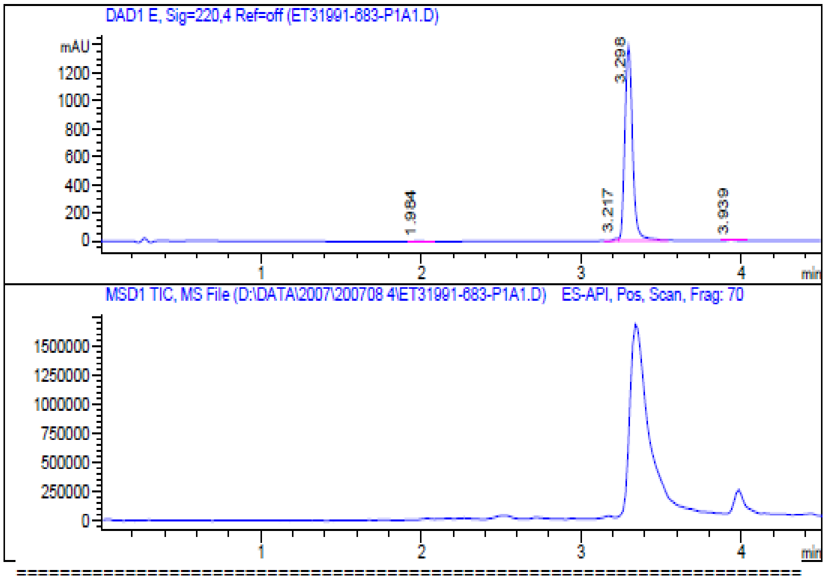

DAD1 E, Sig=220,4 Ref=off (ET31991-683-P1A1.D)
mAU
3.298
1.984
3.217
3.939
min
MSD1 TIC, MS File (D:\DATA\2007\200708 4\ET31991-683-P1A1.D) ES-API, Pos, Scan, Frag: 70
min

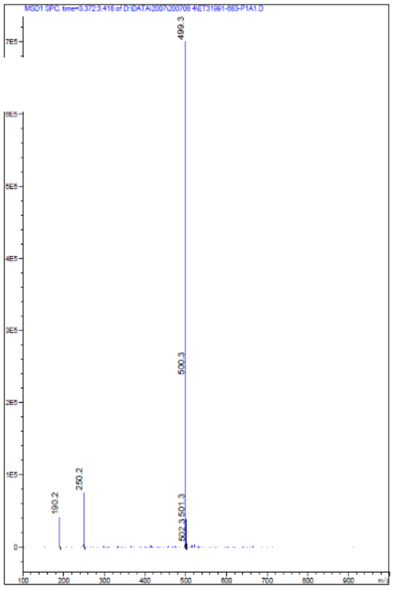

499.3
500.3
501.3
502.3
190.2
250.2

Compound 84

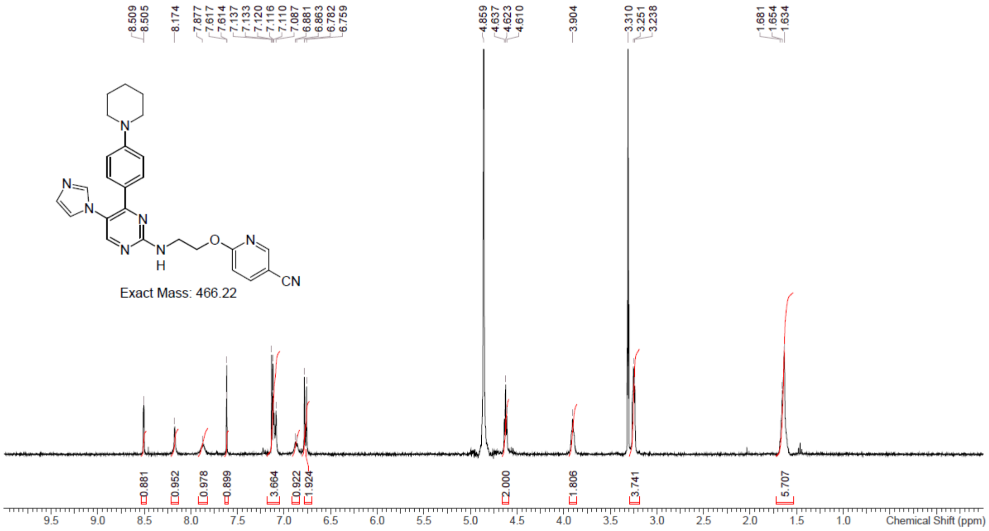

Exact Mass: 466.22
Chemical Shift (ppm)

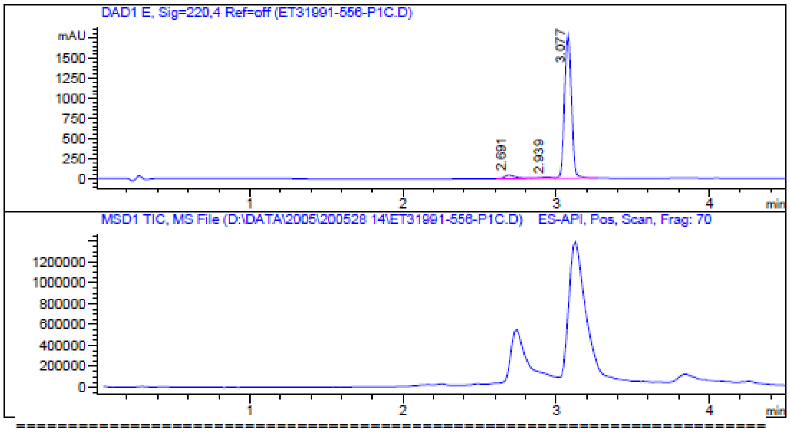

DAD1 E, Sig=220,4 Ref=off (ET31991-556-P1C.D)
3.077
2.691
2.939
MSD1 TIC, MS File (D:\DATA\2005\200528 14\ET31991-556-P1C.D) ES-API, Pos, Scan, Frag: 70

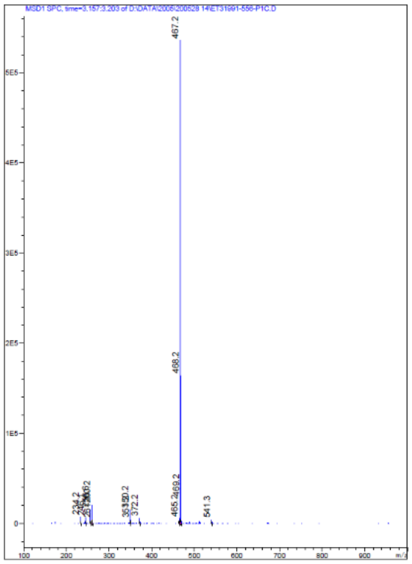

467.2
468.2
541.3

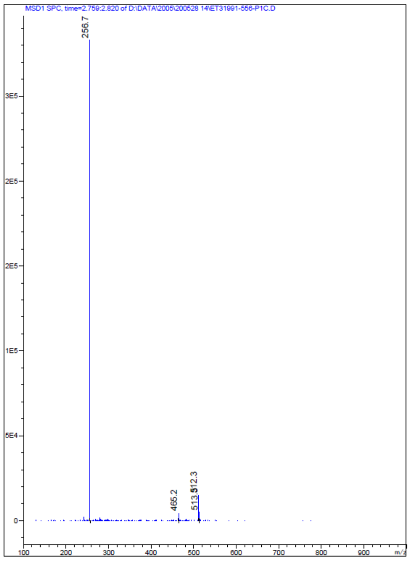

256.7
465.2
512.3

Compound 85

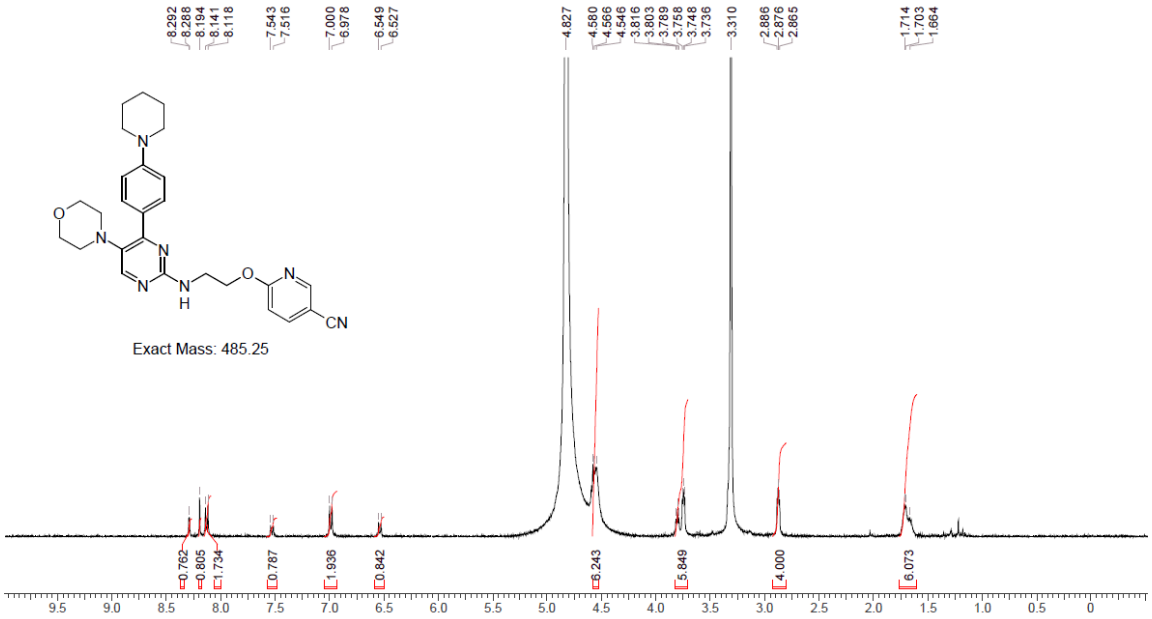

Exact Mass: 485.25

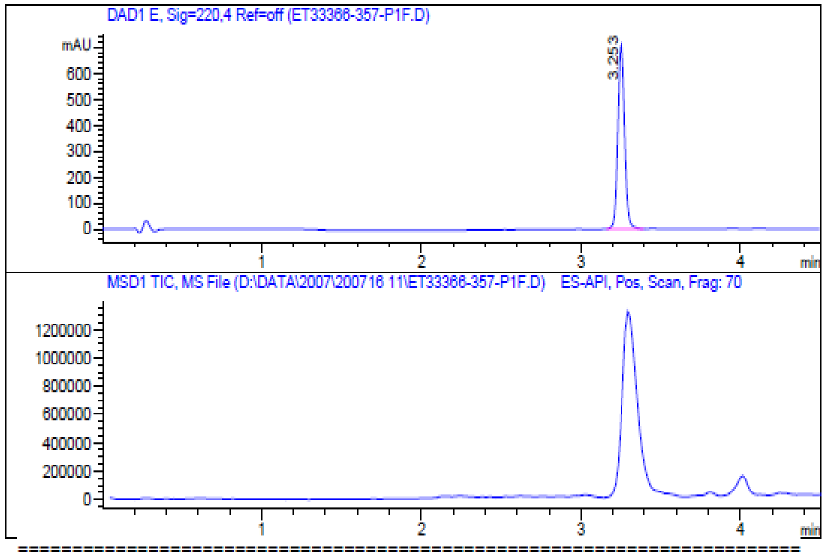

DAD1 E, Sig=220,4 Ref=off (ET33366-357-P1F.D)
MSD1 TIC, MS File (D:\DATA\2007\200716 11\ET33366-357-P1F.D) ES-API, Pos, Scan, Frag: 70

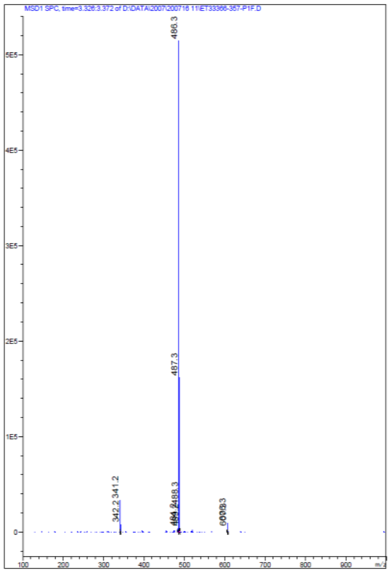

486.3
487.3
341.2

Compound 86

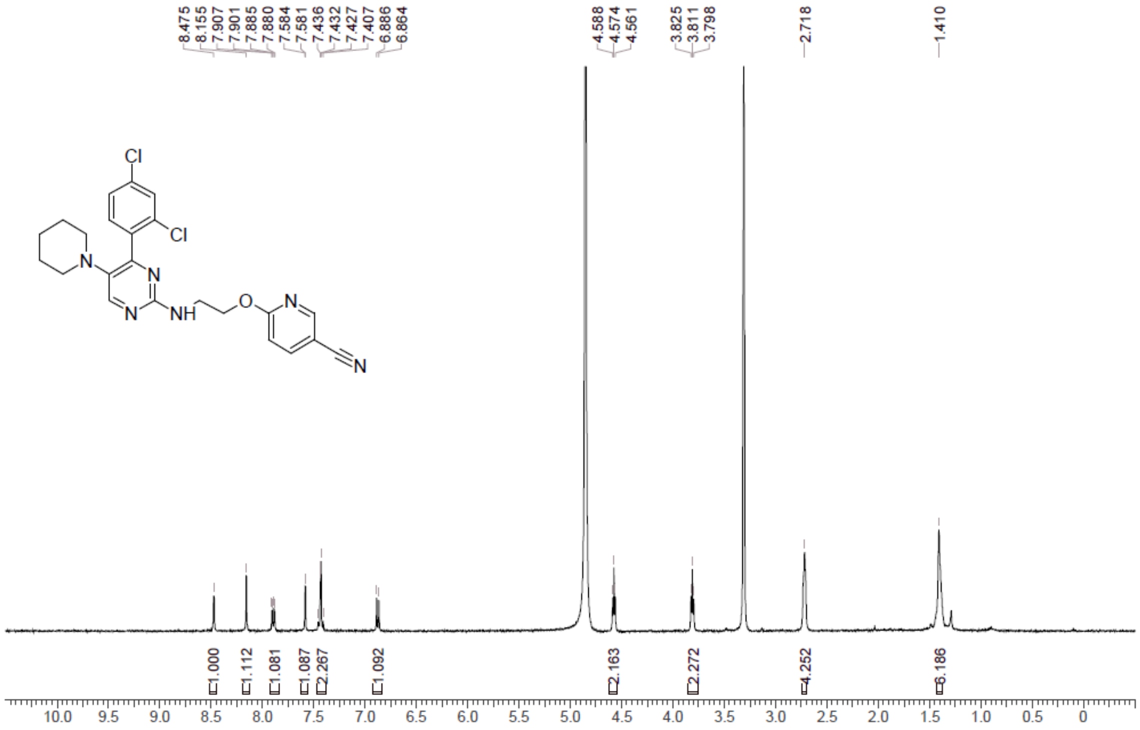

8.475
8.155
7.907
7.901
7.885
7.880
7.584
7.581
7.436
7.432
7.427
7.407
6.886
6.864
4.588
4.574
4.561
3.825
3.811
3.798
2.718
1.410
1.000
1.112
1.081
1.087
2.267
1.092
2.163
2.272
4.252
6.186
10.0 9.5 9.0 8.5 8.0 7.5 7.0 6.5 6.0 5.5 5.0 4.5 4.0 3.5 3.0 2.5 2.0 1.5 1.0 0.5 0

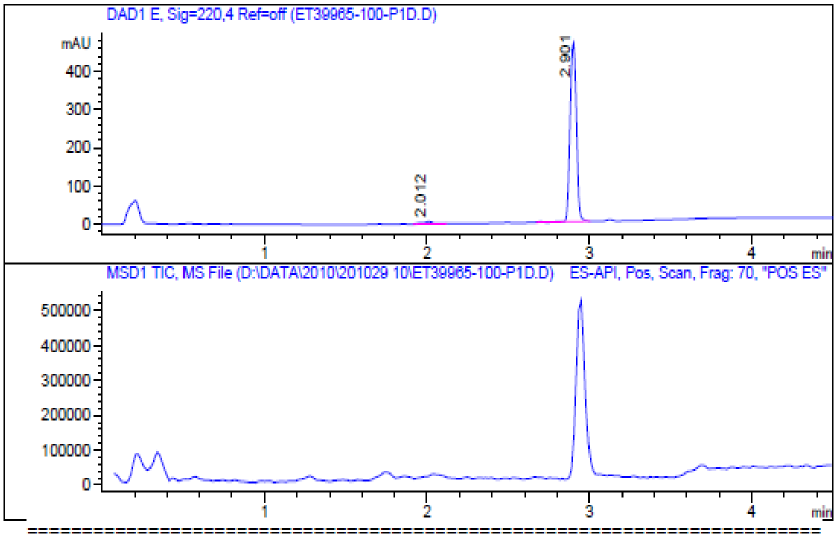

DAD1 E, Sig=220,4 Ref=off (ET39965-100-P1D.D)
mAU
400
300
200
100
0
2.012
2.901
1 2 3 4 min
MSD1 TIC, MS File (D:\DATA\2010\201029 10\ET39965-100-P1D.D) ES-API, Pos, Scan, Frag: 70, "POS ES"
500000
400000
300000
200000
100000
0
1 2 3 4 min

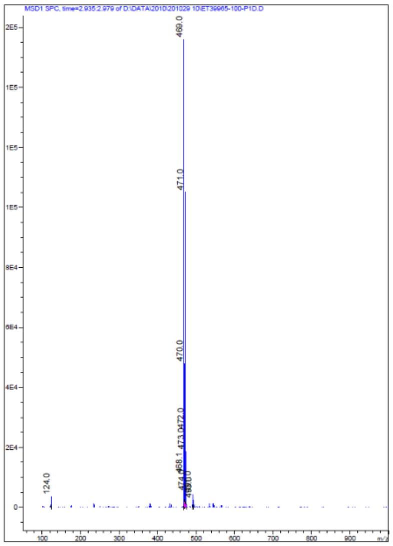

469.0
471.0
470.0
124.0

Compound 87

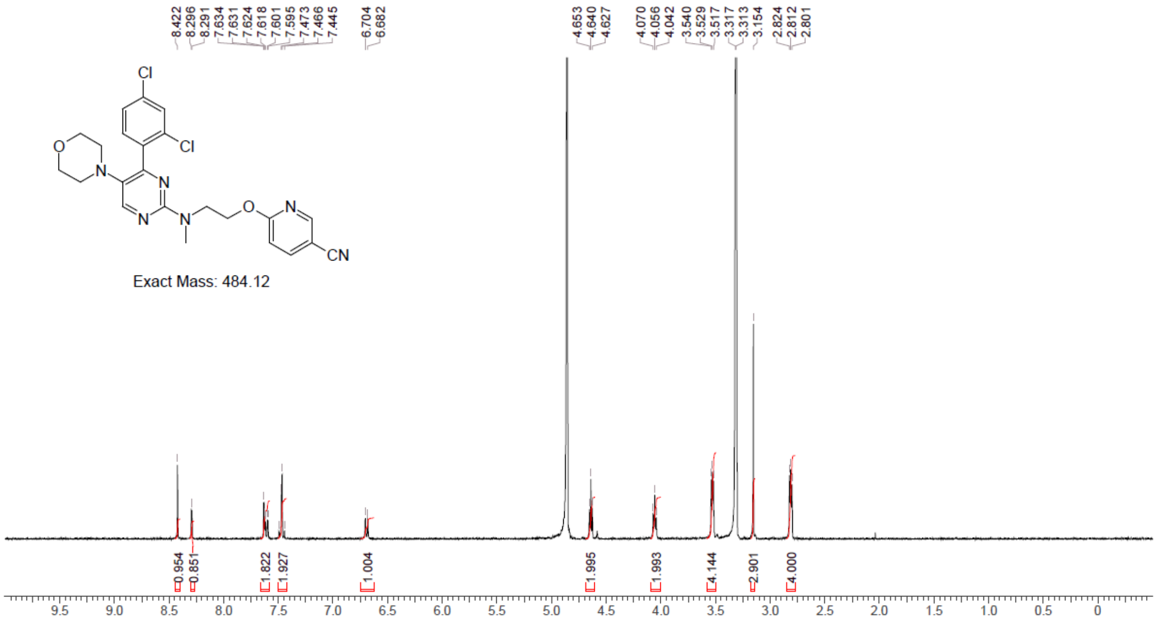

Cl
Cl
O
N
N
N
N
O
N
CN
Exact Mass: 484.12

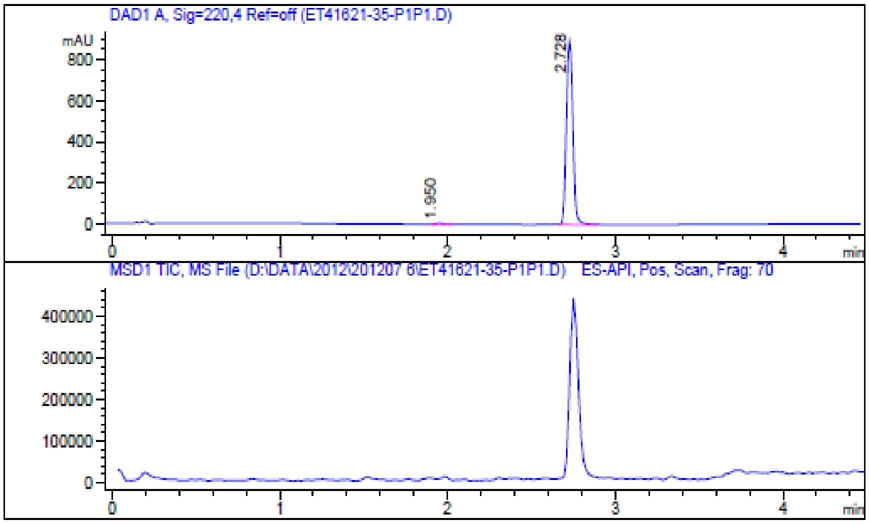

DAD1 A, Sig=220,4 Ref=off (ET41621-35-P1P1.D)
mAU
2.728
1.950
min
MSD1 TIC, MS File (D:\DATA\2012\201207 6\ET41621-35-P1P1.D) ES-API, Pos, Scan, Frag: 70
min

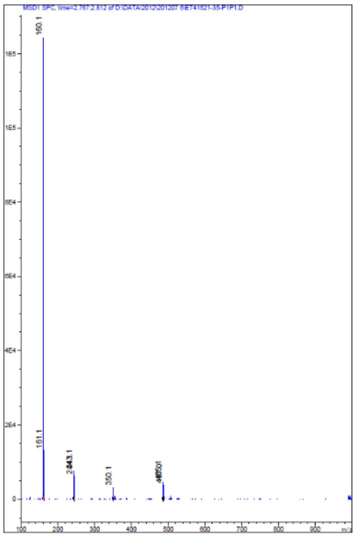

160.1
161.1
243.1
350.1

Compound 88

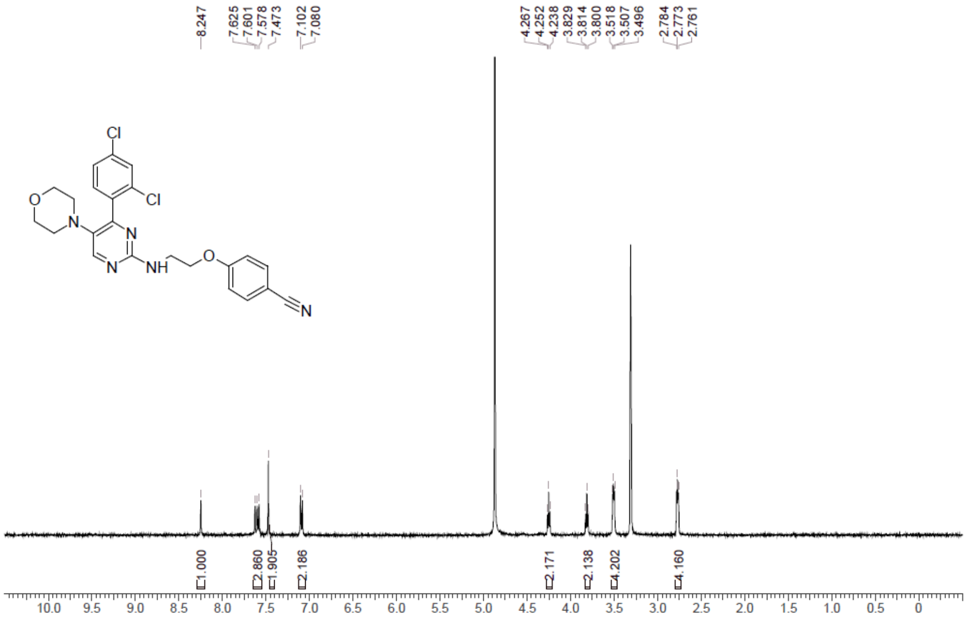

8.247
7.625
7.601
7.578
7.473
7.102
7.080
4.267
4.252
4.238
3.829
3.814
3.800
3.518
3.507
3.496
2.784
2.773
2.761
Cl
Cl
O
N
N
N
NH
O
N
1.000
2.860
1.905
2.186
2.171
2.138
4.202
4.160

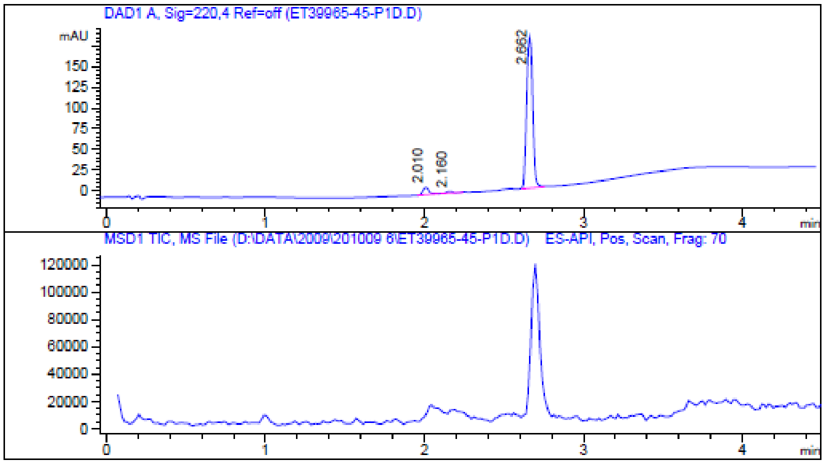

DAD1 A, Sig=220,4 Ref=off (ET39965-45-P1D.D)
mAU
2.662
2.010
2.160
min
MSD1 TIC, MS File (D:\DATA\2009\201009 6\ET39965-45-P1D.D) ES-API, Pos, Scan, Frag: 70
min

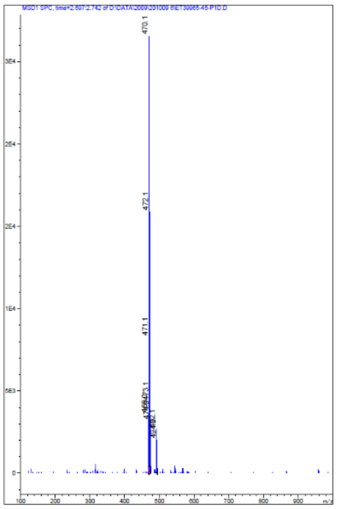

470.1
472.1
471.1

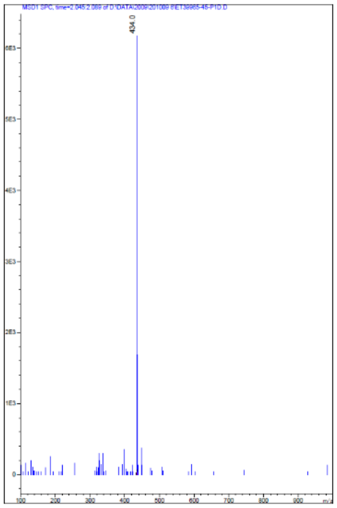

434.0

Compound 89

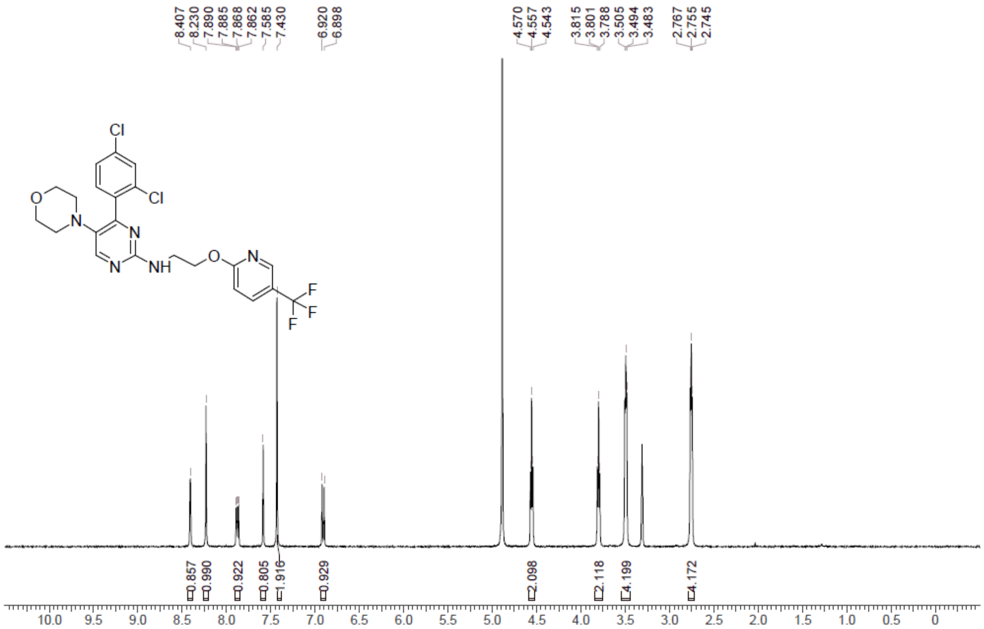
8.407 8.230 7.890 7.885 7.868 7.862 7.585 7.430 6.920 6.898 4.570 4.557 4.543 3.815 3.801 3.788 3.505 3.494 3.483 2.767 2.755 2.745
0.857 0.990 0.922 0.805 1.916 0.929 2.098 2.118 4.199 4.172
10.0 9.5 9.0 8.5 8.0 7.5 7.0 6.5 6.0 5.5 5.0 4.5 4.0 3.5 3.0 2.5 2.0 1.5 1.0 0.5 0

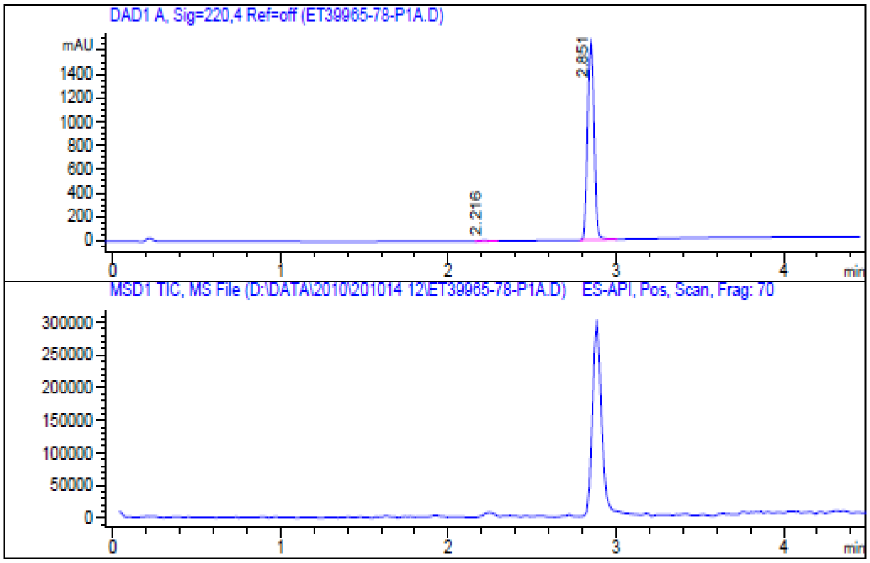
DAD1 A, Sig=220,4 Ref=off (ET39965-78-P1A.D)
mAU
2.851
2.216
min
MSD1 TIC, MS File (D:\DATA\2010\201014 12\ET39965-78-P1A.D) ES-API, Pos, Scan, Frag: 70
min

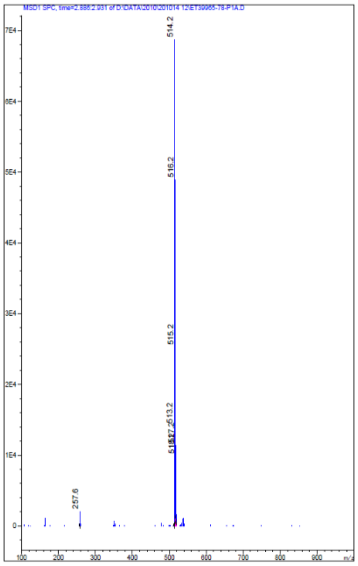
514.2
516.2
515.2
513.2
257.6

Compound 90

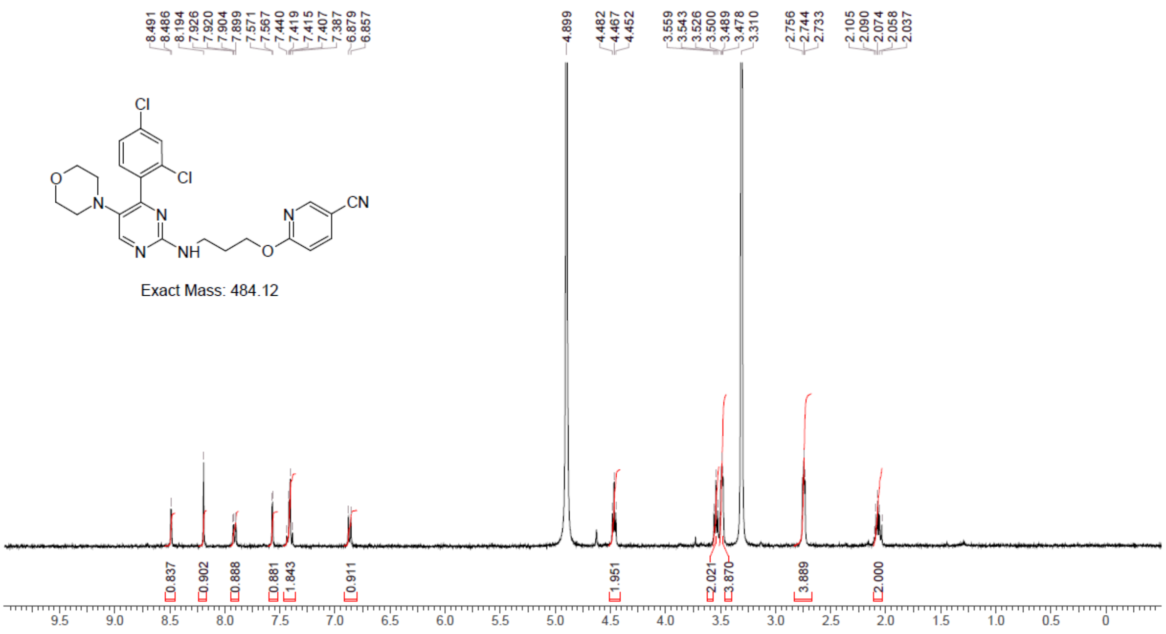
Exact Mass: 484.12
8.491
8.486
8.194
7.926
7.920
7.904
7.899
7.571
7.567
7.440
7.419
7.415
7.407
7.387
6.879
6.857
4.899
4.482
4.467
4.452
3.559
3.543
3.526
3.500
3.489
3.478
3.310
2.756
2.744
2.733
2.105
2.090
2.074
2.058
2.037
0.837
0.902
0.888
0.881
1.843
0.911
1.951
2.021
3.870
3.889
2.000

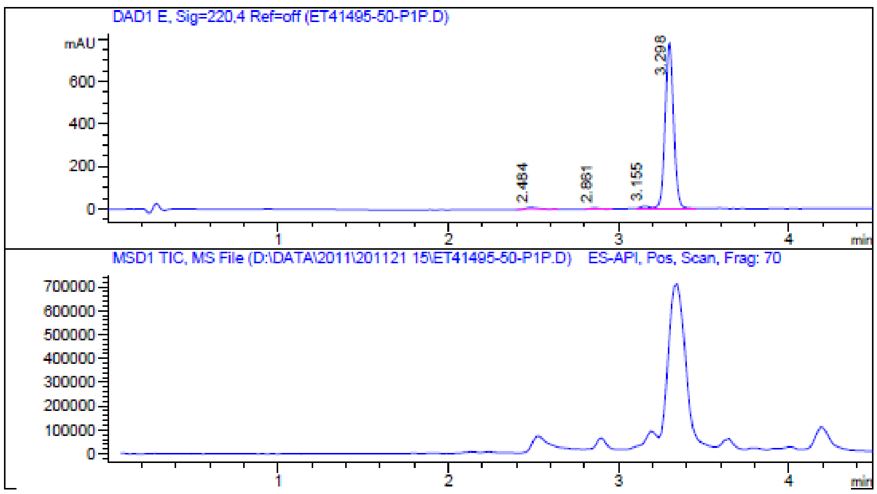
DAD1 E, Sig=220,4 Ref=off (ET41495-50-P1P.D)
mAU
3.298
2.484
2.861
3.155
MSD1 TIC, MS File (D:\DATA\2011\201121 15\ET41495-50-P1P.D) ES-API, Pos, Scan, Frag: 70
min

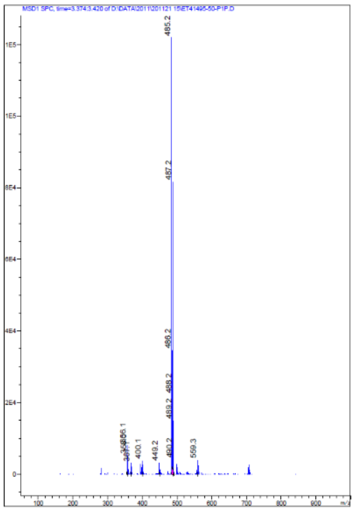
485.2
487.2
486.2
488.2
400.1
449.2
559.3
==

Compound 91

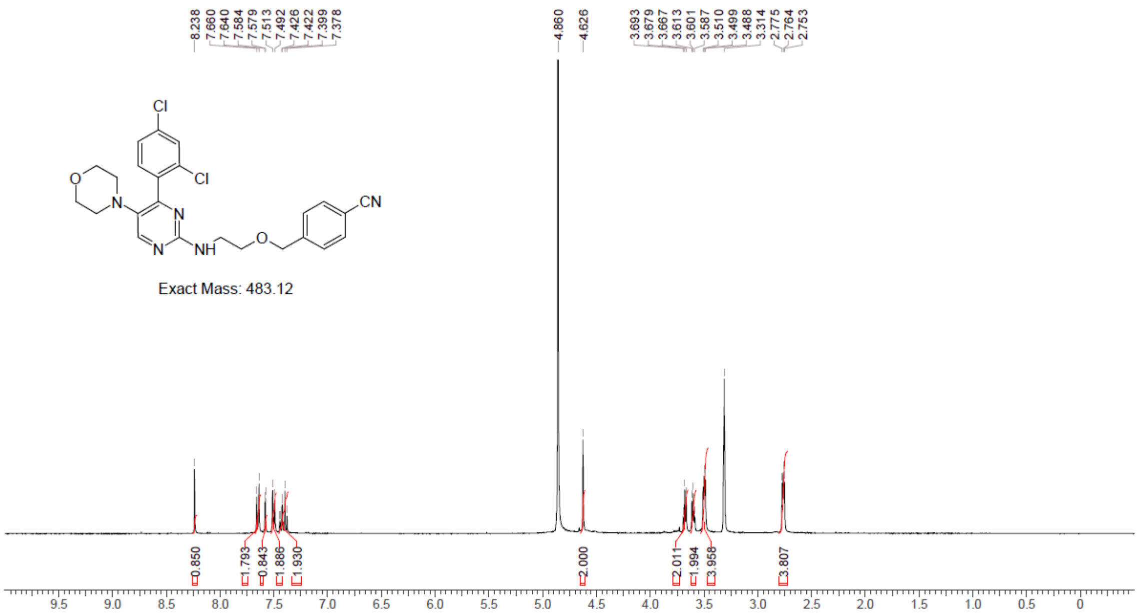

8.238
7.660
7.640
7.584
7.579
7.513
7.492
7.426
7.422
7.399
7.378
4.860
4.626
3.693
3.679
3.667
3.613
3.601
3.587
3.510
3.499
3.488
3.314
2.775
2.764
2.753
Cl
Cl
O
N
N
N
NH
O
CN
Exact Mass: 483.12
0.850
1.793
0.843
1.886
1.930
2.000
2.011
1.994
3.958
3.807
9.5
9.0
8.5
8.0
7.5
7.0
6.5
6.0
5.5
5.0
4.5
4.0
3.5
3.0
2.5
2.0
1.5
1.0
0.5
0

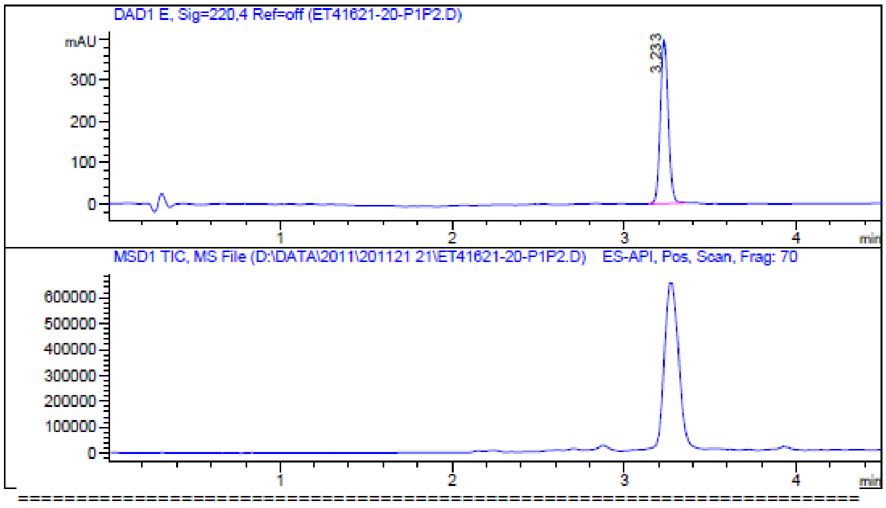

DAD1 E, Sig=220,4 Ref=off (ET41621-20-P1P2.D)
mAU
300
200
100
0
3.233
1
2
3
4
min
MSD1 TIC, MS File (D:\DATA\2011\201121 21\ET41621-20-P1P2.D) ES-API, Pos, Scan, Frag: 70
600000
500000
400000
300000
200000
100000
0
1
2
3
4
min

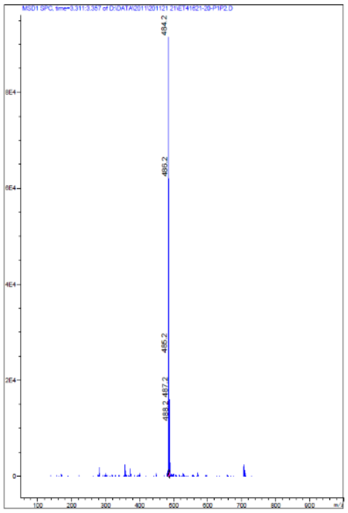

484.2
486.2
485.2
487.2
488.2

Compound 92

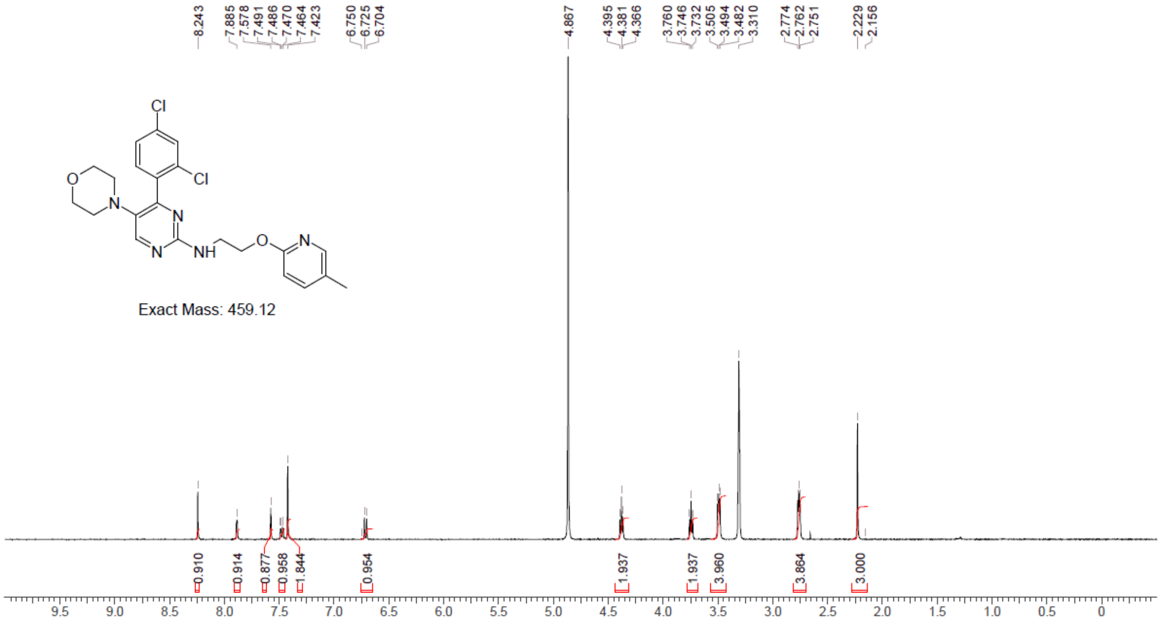
Cl
Cl
O
N
N
N
NH
O
N
Exact Mass: 459.12
8.243
7.885
7.578
7.491
7.486
7.470
7.464
7.423
6.750
6.725
6.704
4.867
4.395
4.381
4.366
3.760
3.746
3.732
3.505
3.494
3.482
3.310
2.774
2.762
2.751
2.229
2.156
0.910
0.914
0.877
0.958
1.844
0.954
1.937
1.937
3.960
3.864
3.000
9.5
9.0
8.5
8.0
7.5
7.0
6.5
6.0
5.5
5.0
4.5
4.0
3.5
3.0
2.5
2.0
1.5
1.0
0.5
0

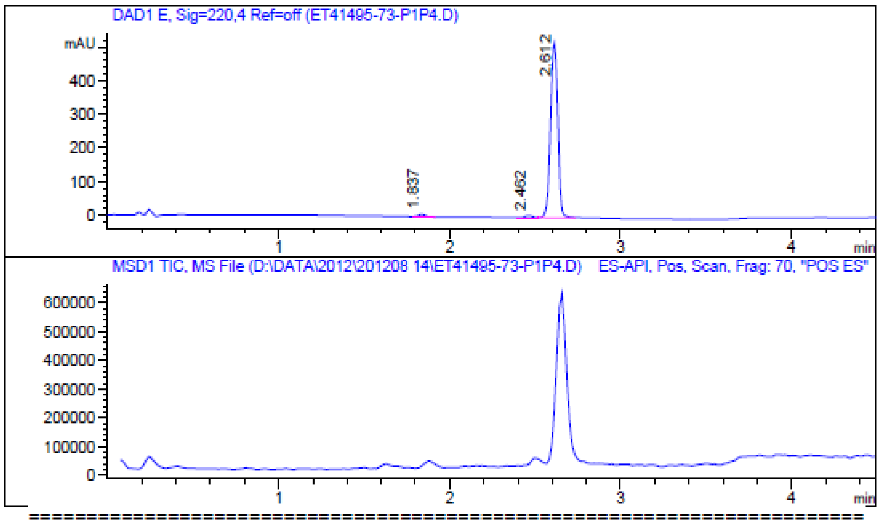
DAD1 E, Sig=220,4 Ref=off (ET41495-73-P1P4.D)
mAU
400
300
200
100
0
2.612
1.837
2.462
1
2
3
4
min
MSD1 TIC, MS File (D:\DATA\2012\201208 14\ET41495-73-P1P4.D) ES-API, Pos, Scan, Frag: 70, "POS ES"
600000
500000
400000
300000
200000
100000
0
1
2
3
4
min
==

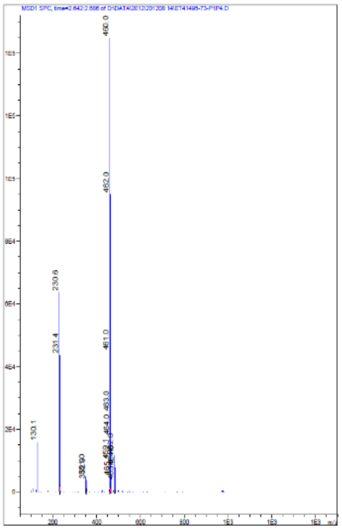
460.0
462.0
230.6
231.4
461.0
130.1

Compound 93

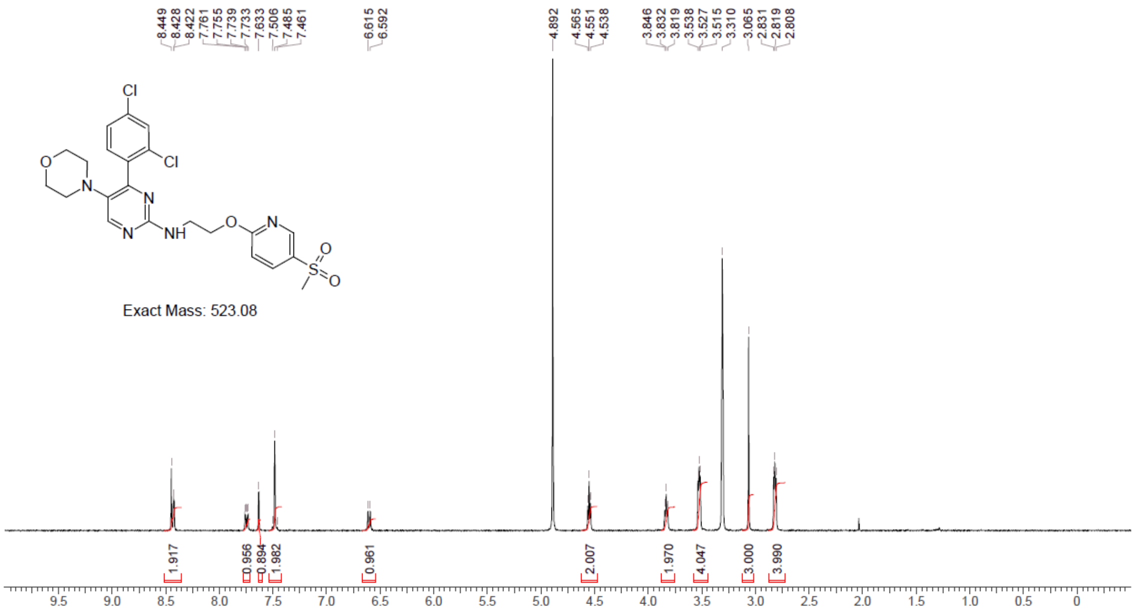
Exact Mass: 523.08

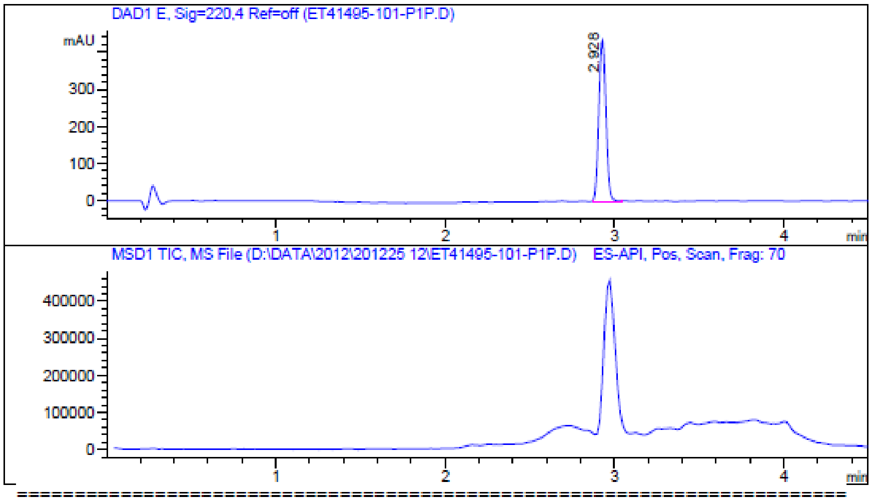
DAD1 E, Sig=220,4 Ref=off (ET41495-101-P1P.D)
MSD1 TIC, MS File (D:\DATA\2012\201225 12\ET41495-101-P1P.D) ES-API, Pos, Scan, Frag: 70

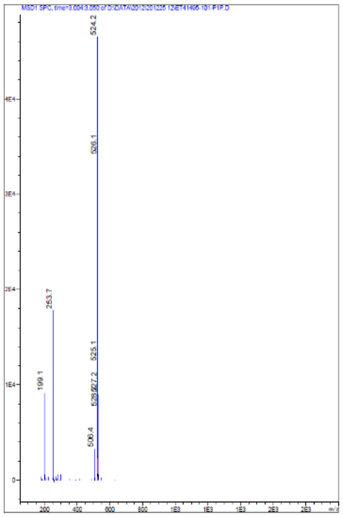

Compound 94

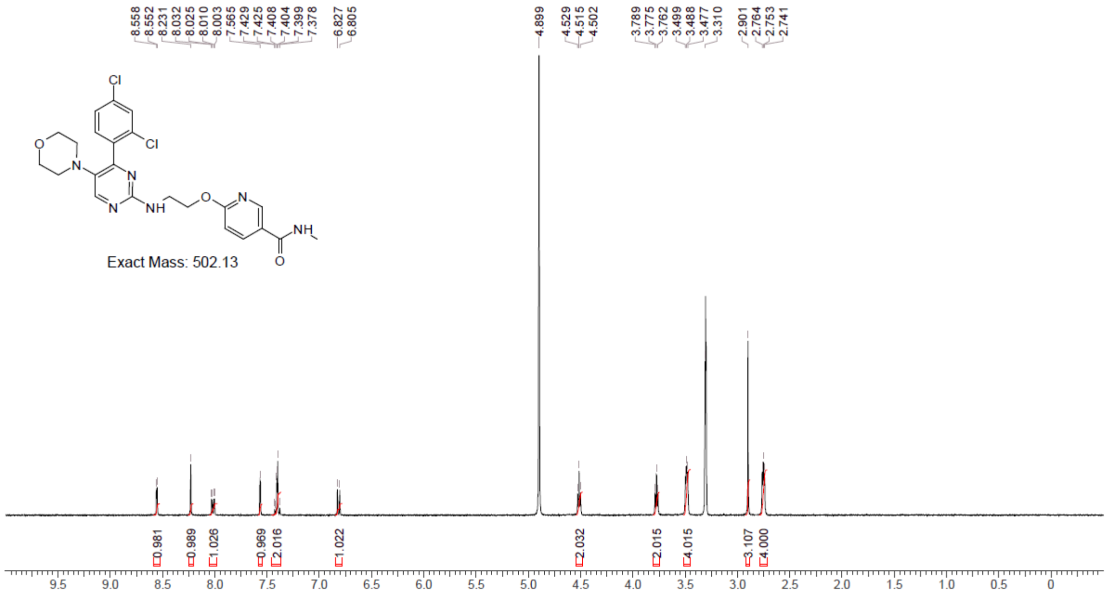
Exact Mass: 502.13

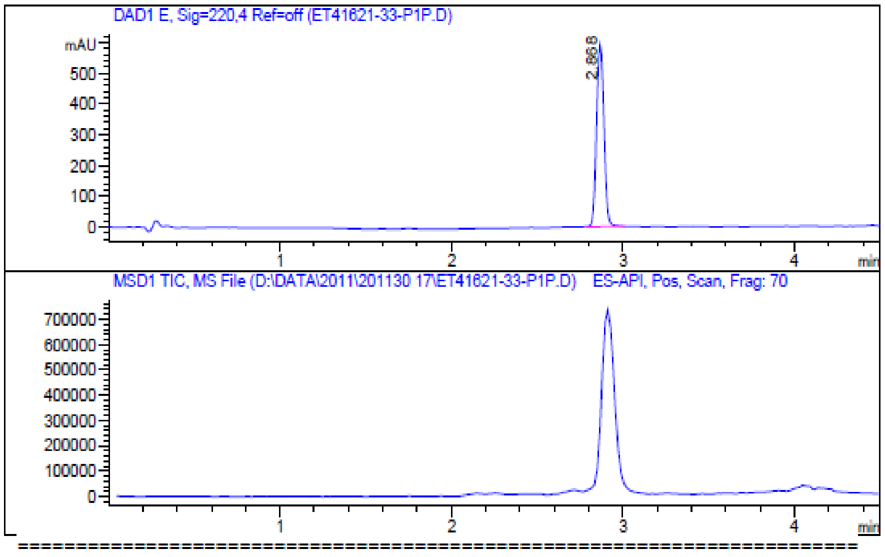
DAD1 E, Sig=220,4 Ref=off (ET41621-33-P1P.D)
MSD1 TIC, MS File (D:\DATA\2011\201130 17\ET41621-33-P1P.D) ES-API, Pos, Scan, Frag: 70

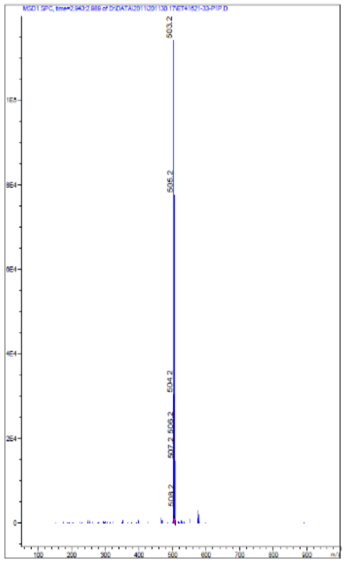

Compound 95

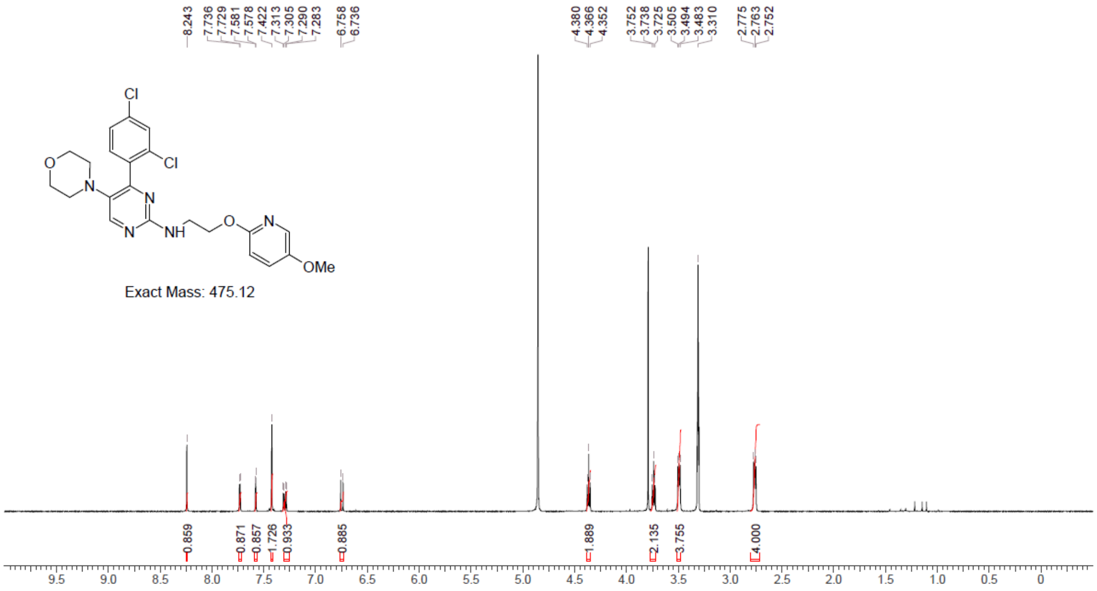

Cl
Cl
O
N
N
N
NH
O
N
OMe
Exact Mass: 475.12

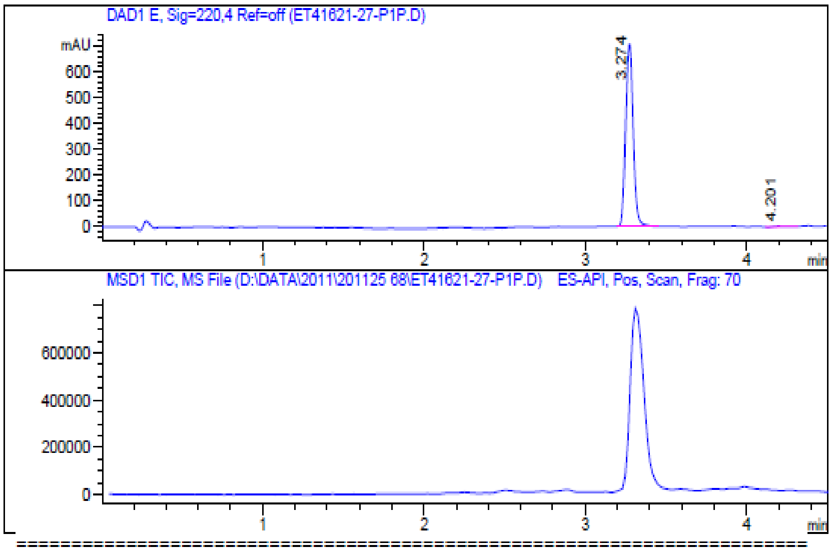

DAD1 E, Sig=220,4 Ref=off (ET41621-27-P1P.D)
mAU
3.274
4.201
min
MSD1 TIC, MS File (D:\DATA\2011\201125 68\ET41621-27-P1P.D)   ES-API, Pos, Scan, Frag: 70
min

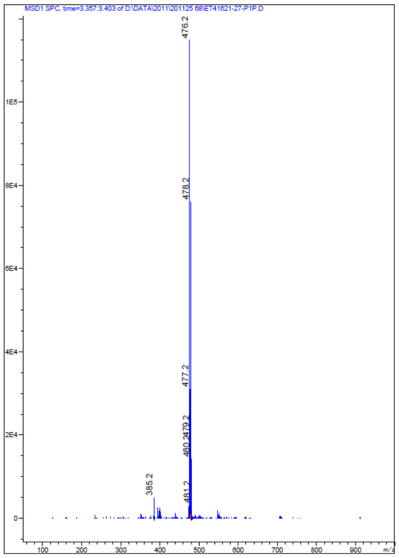

476.2
478.2
477.2
385.2

Compound 96

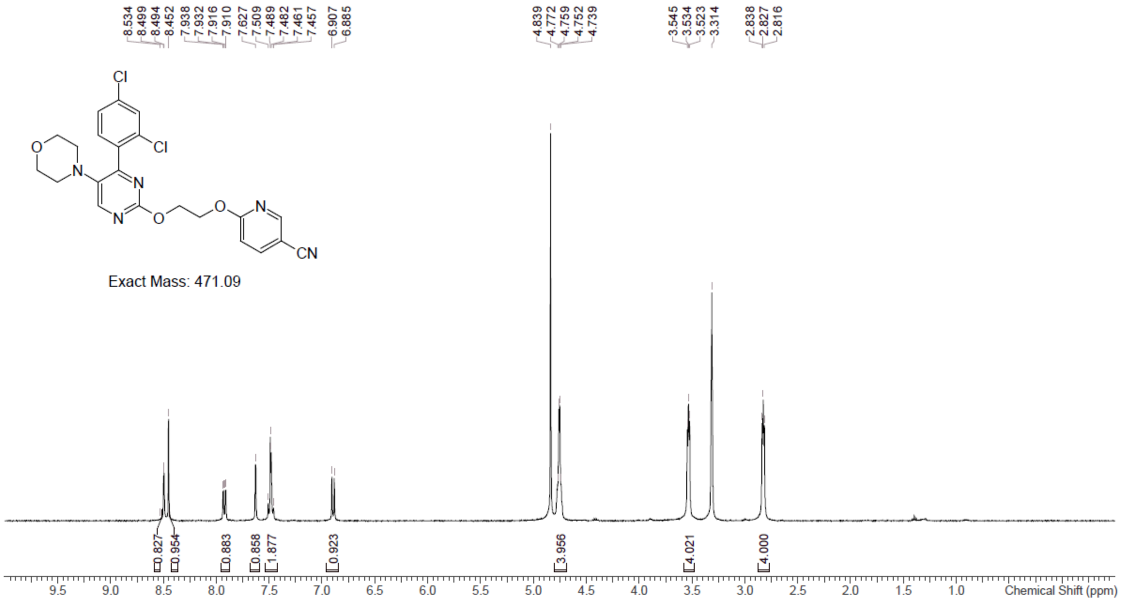

CN
Cl
N
O
Exact Mass: 471.09
Chemical Shift (ppm)

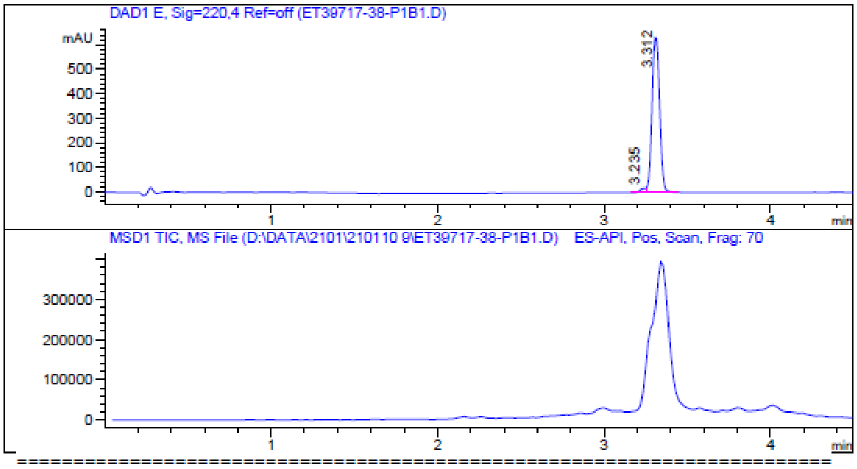

DAD1 E, Sig=220,4 Ref=off (ET39717-38-P1B1.D)
mAU
3.312
3.235
MSD1 TIC, MS File (D:\DATA\2101\210110 9\ET39717-38-P1B1.D) ES-API, Pos, Scan, Frag: 70
min

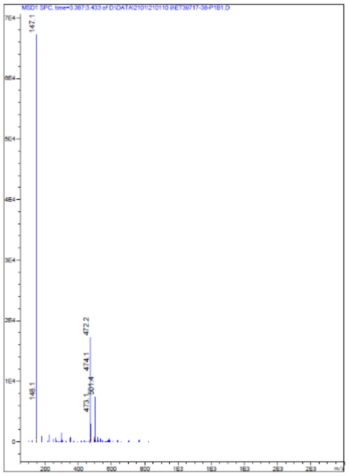

147.1
472.2
474.1

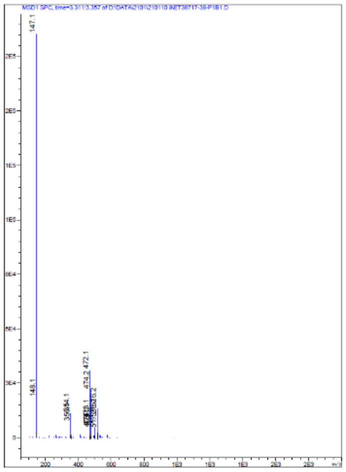

147.1
472.1

Compound 97

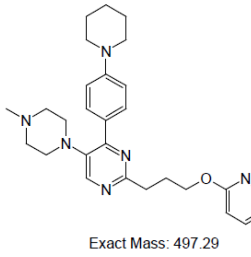
N
N
N
N
N
O
N
CN
Exact Mass: 497.29

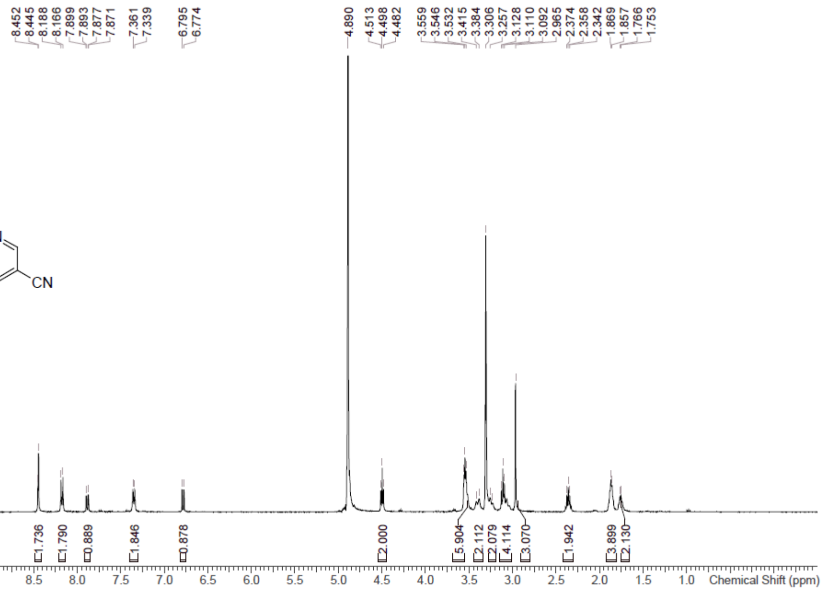
Chemical Shift (ppm)

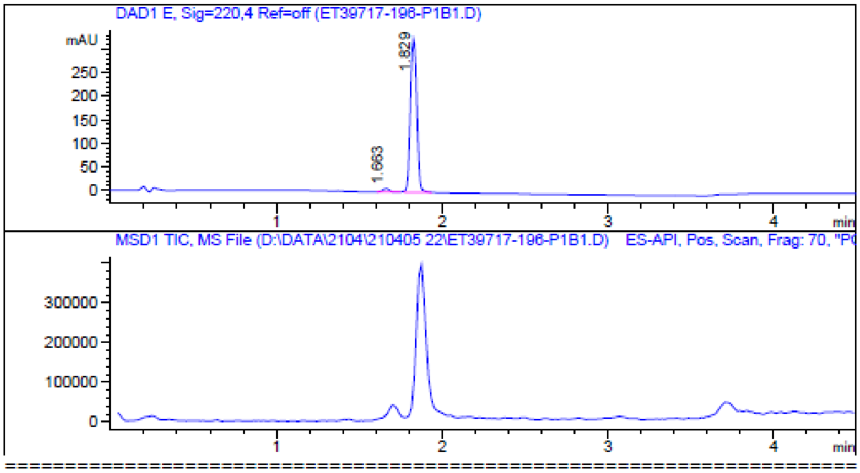
DAD1 E, Sig=220,4 Ref=off (ET39717-196-P1B1.D)
mAU
1.829
1.663
min
MSD1 TIC, MS File (D:\DATA\2104\210405 22\ET39717-196-P1B1.D) ES-API, Pos, Scan, Frag: 70, "P
min

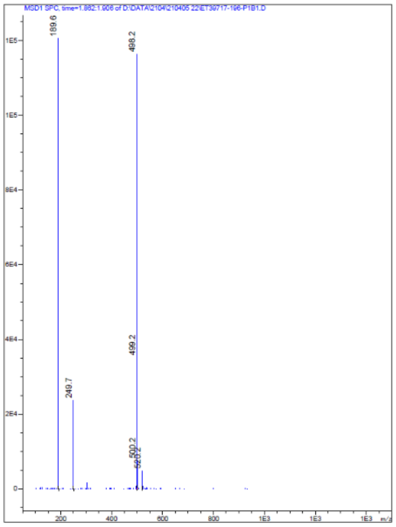
189.6
498.2
249.7
499.2
500.2

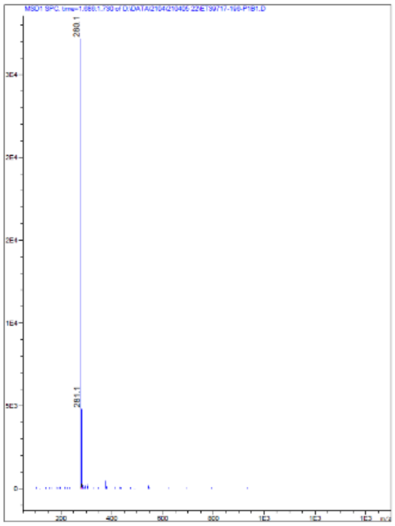
280.1
281.1

Compound 98

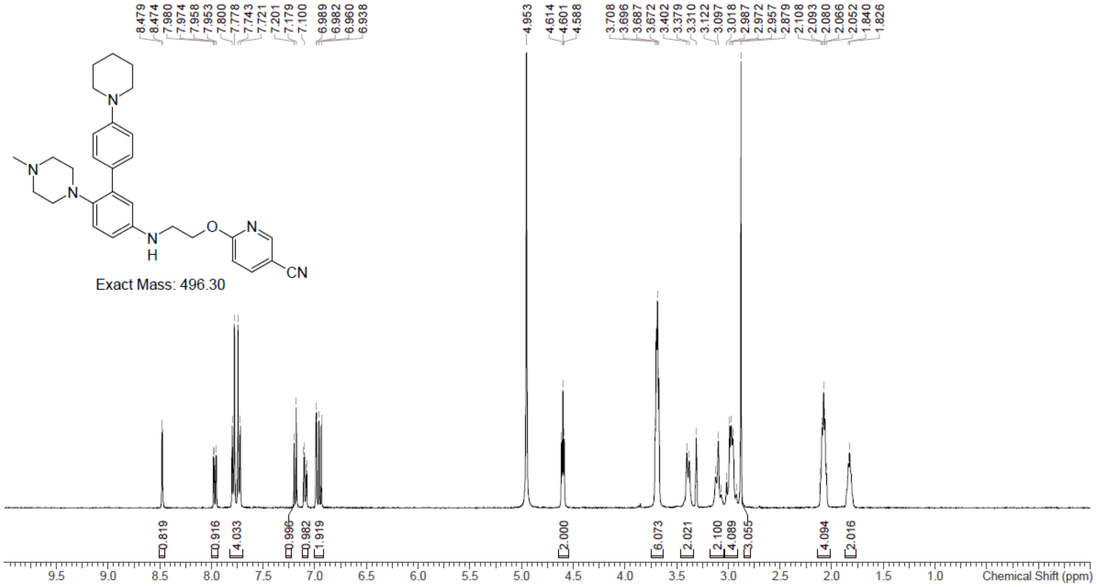

8.479
8.474
7.980
7.974
7.958
7.953
7.800
7.778
7.743
7.721
7.201
7.179
7.100
6.989
6.982
6.960
6.938
4.953
4.614
4.601
4.588
3.708
3.696
3.687
3.672
3.402
3.379
3.310
3.122
3.097
3.018
2.987
2.972
2.957
2.879
2.108
2.093
2.080
2.066
2.052
1.840
1.826
N
N
N
N
H
O
N
CN
Exact Mass: 496.30
0.819
0.916
4.033
0.996
0.982
1.919
2.000
6.073
2.021
2.100
4.089
3.055
4.094
2.016
9.5
9.0
8.5
8.0
7.5
7.0
6.5
6.0
5.5
5.0
4.5
4.0
3.5
3.0
2.5
2.0
1.5
1.0
Chemical Shift (ppm)

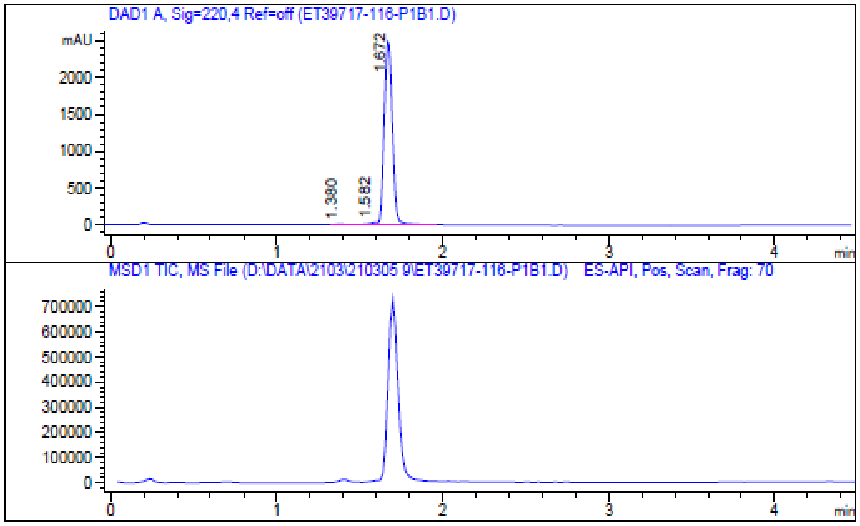

DAD1 A, Sig=220,4 Ref=off (ET39717-116-P1B1.D)
mAU
2000
1500
1000
500
0
1.672
1.380
1.582
0
1
2
3
4
min
MSD1 TIC, MS File (D:\DATA\2103\210305 9\ET39717-116-P1B1.D) ES-API, Pos, Scan, Frag: 70
700000
600000
500000
400000
300000
200000
100000
0
0
1
2
3
4
min

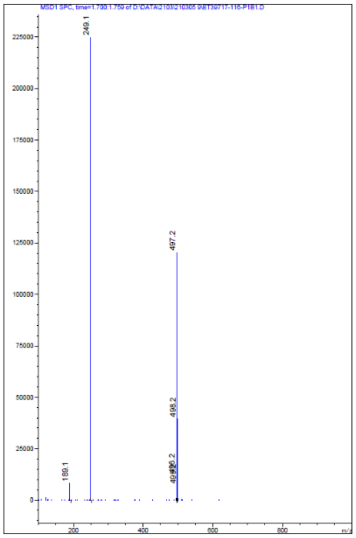

249.1
497.2
498.2
189.1

Compound 99

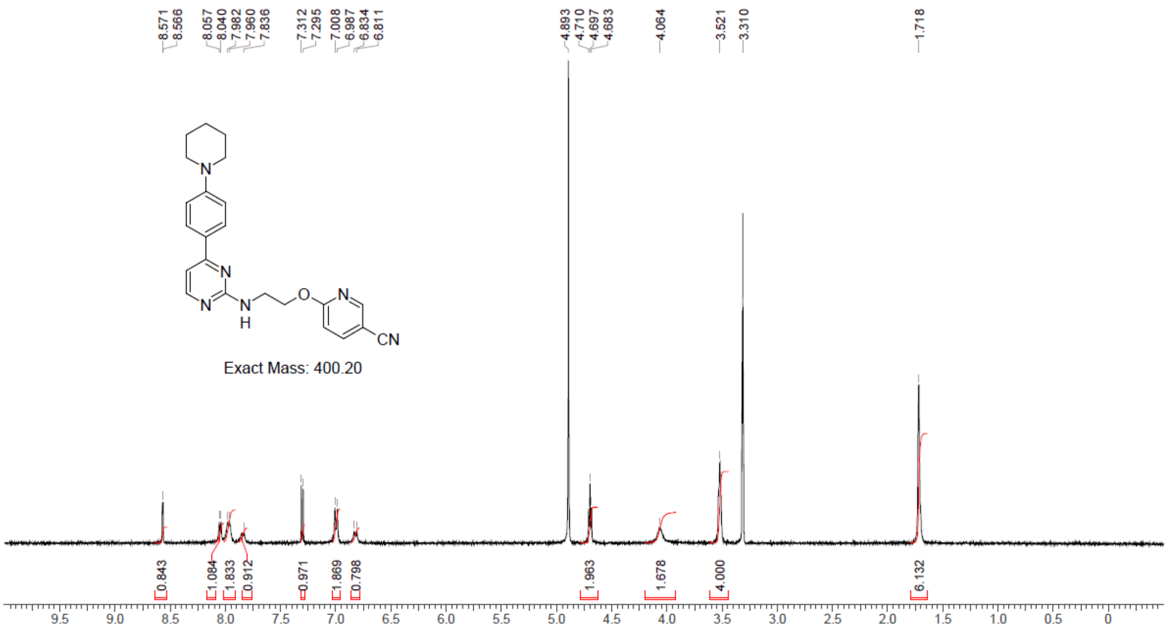

Exact Mass: 400.20

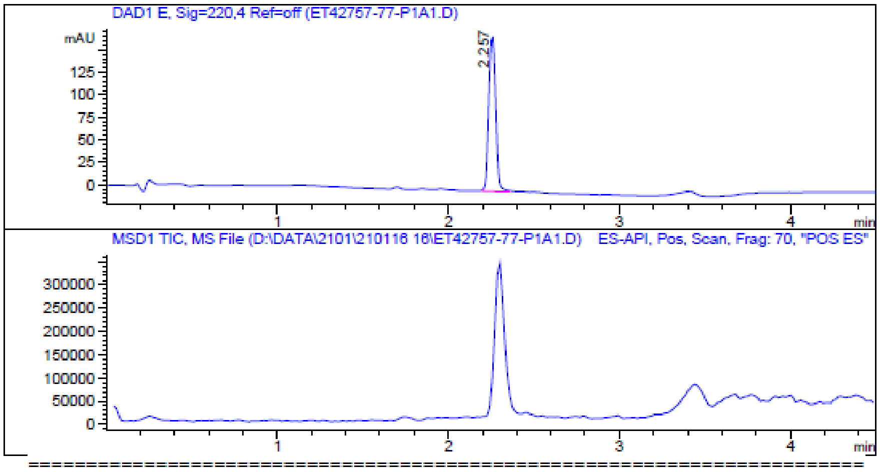

DAD1 E, Sig=220,4 Ref=off (ET42757-77-P1A1.D)
2.257
MSD1 TIC, MS File (D:\DATA\2101\210116 16\ET42757-77-P1A1.D) ES-API, Pos, Scan, Frag: 70, "POS ES"

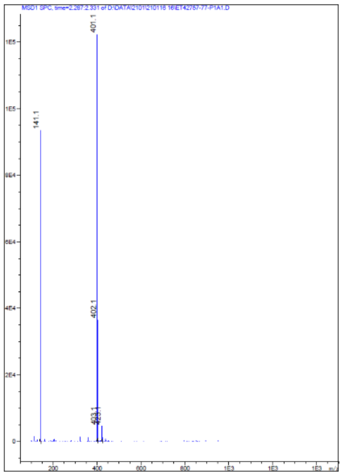

401.1
141.1
402.1

Compound 100

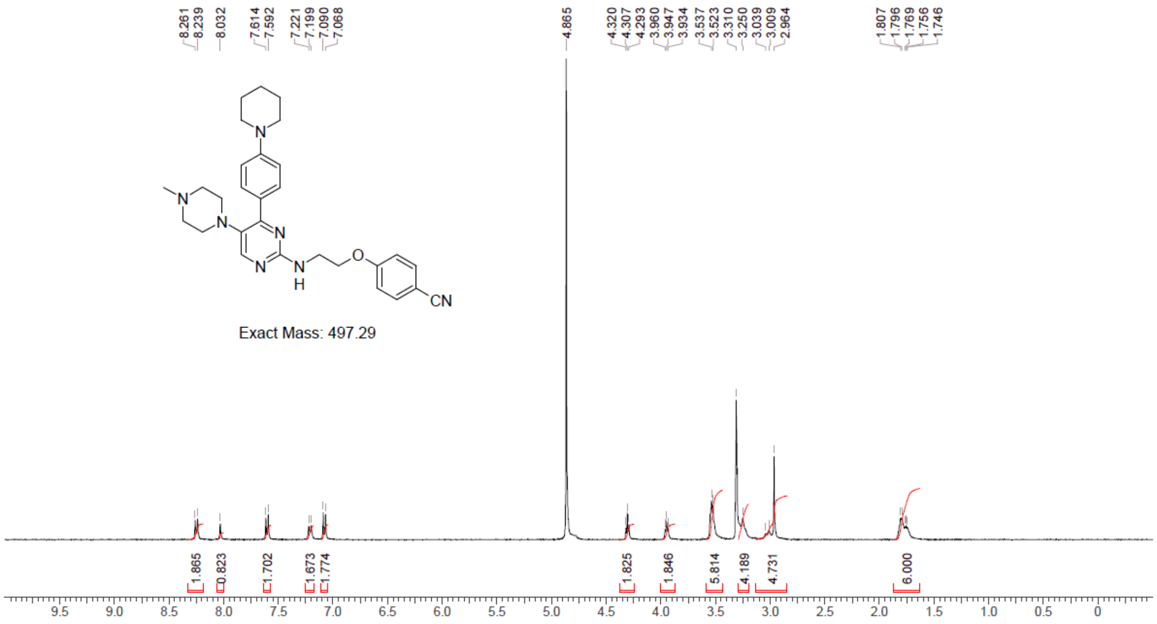

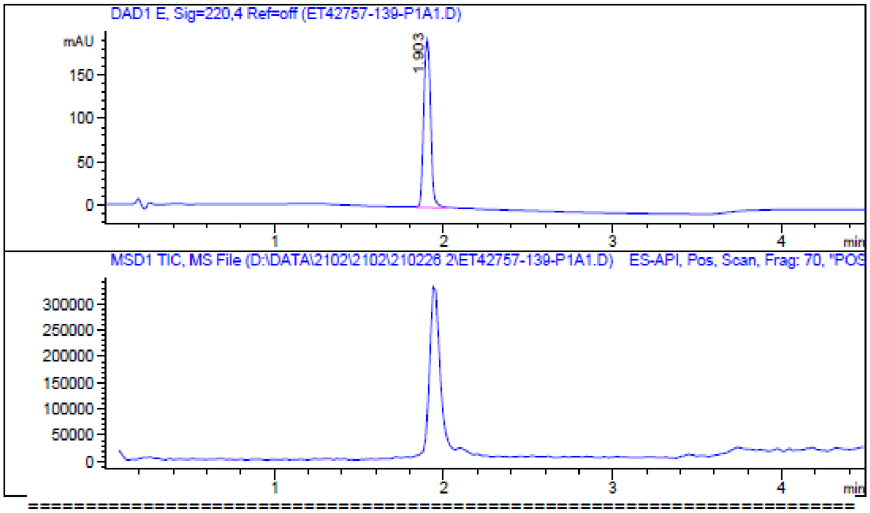

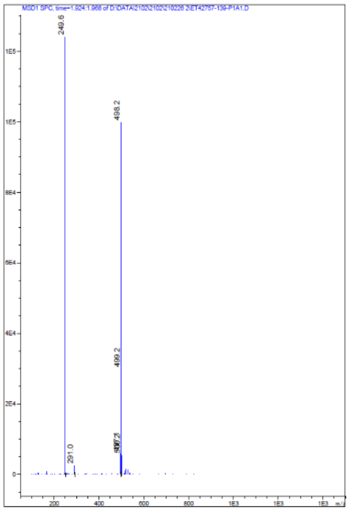

Compound 101

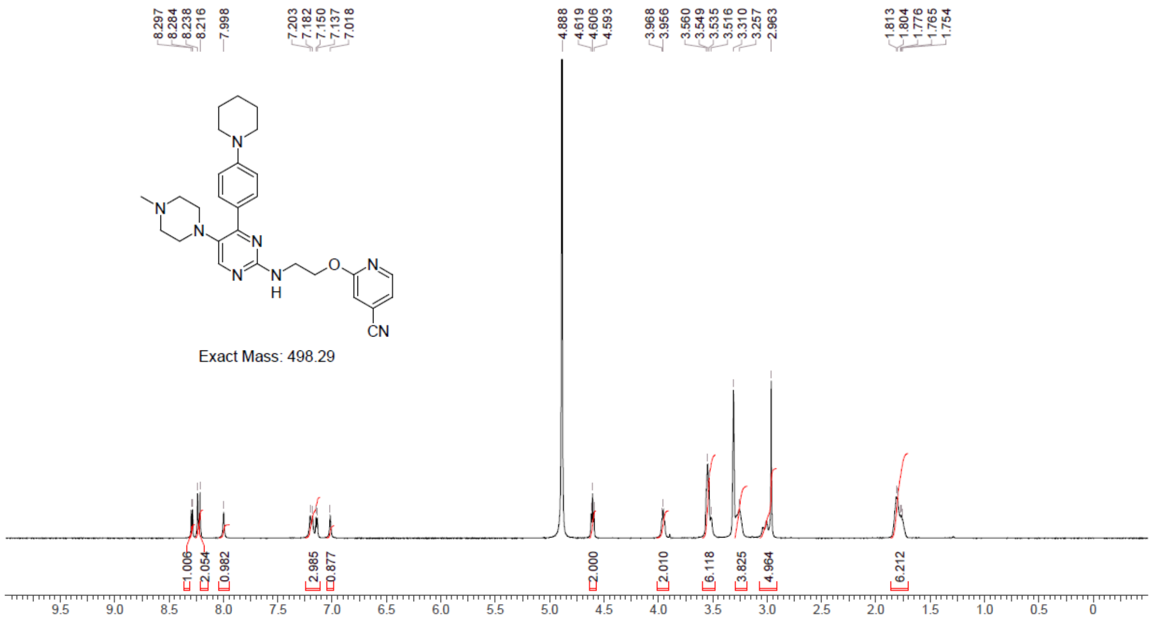

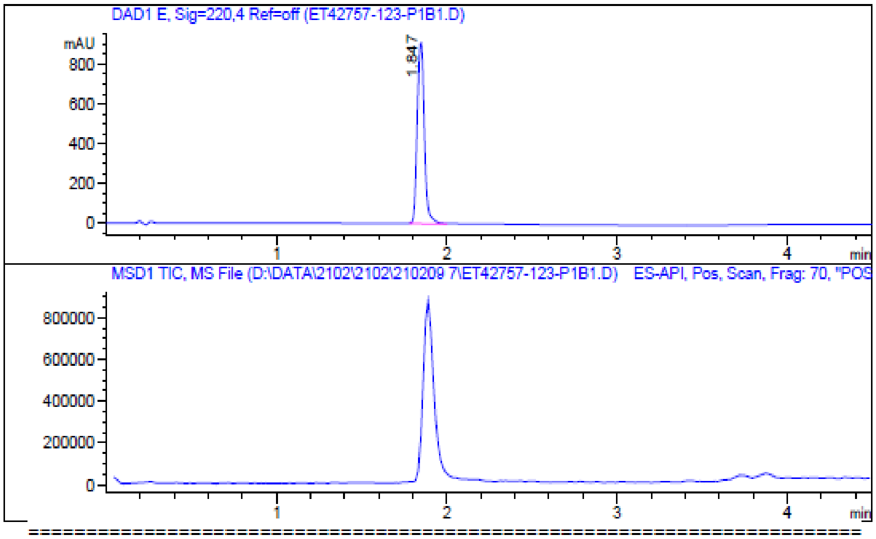

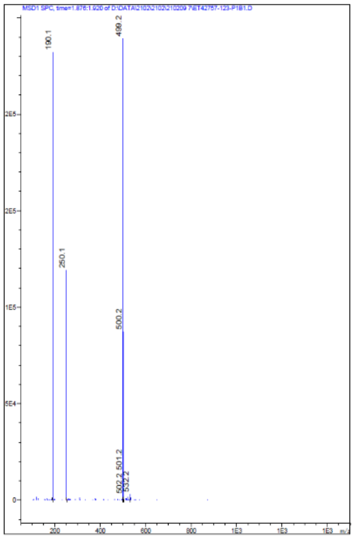

Compound 102

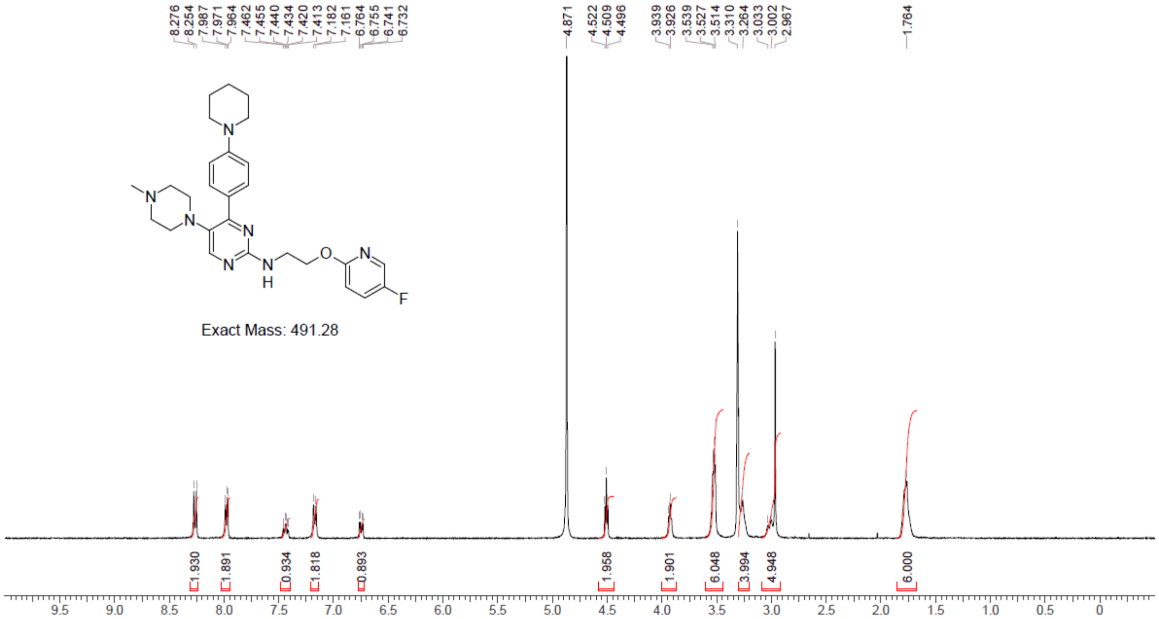

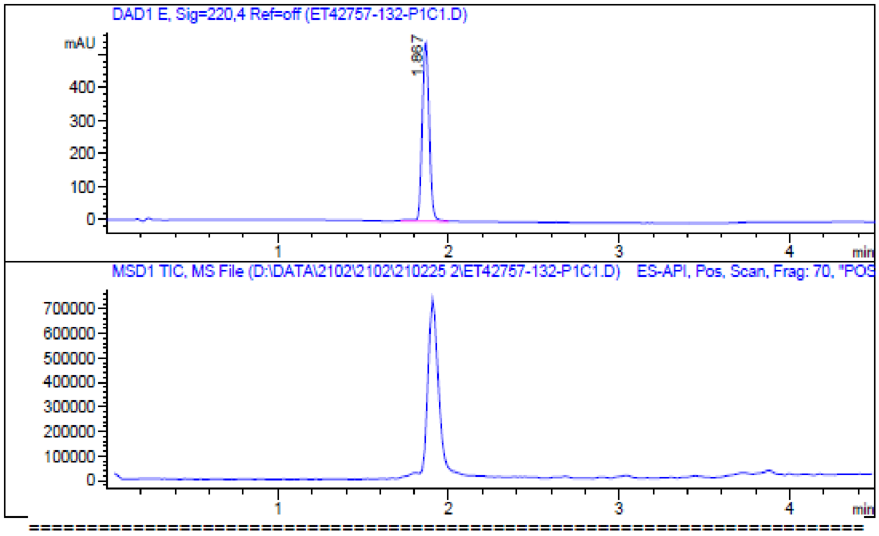

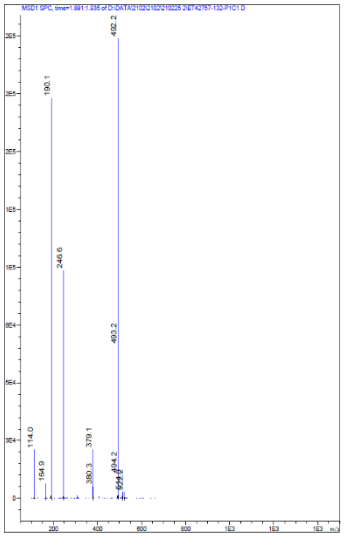

Compound 103

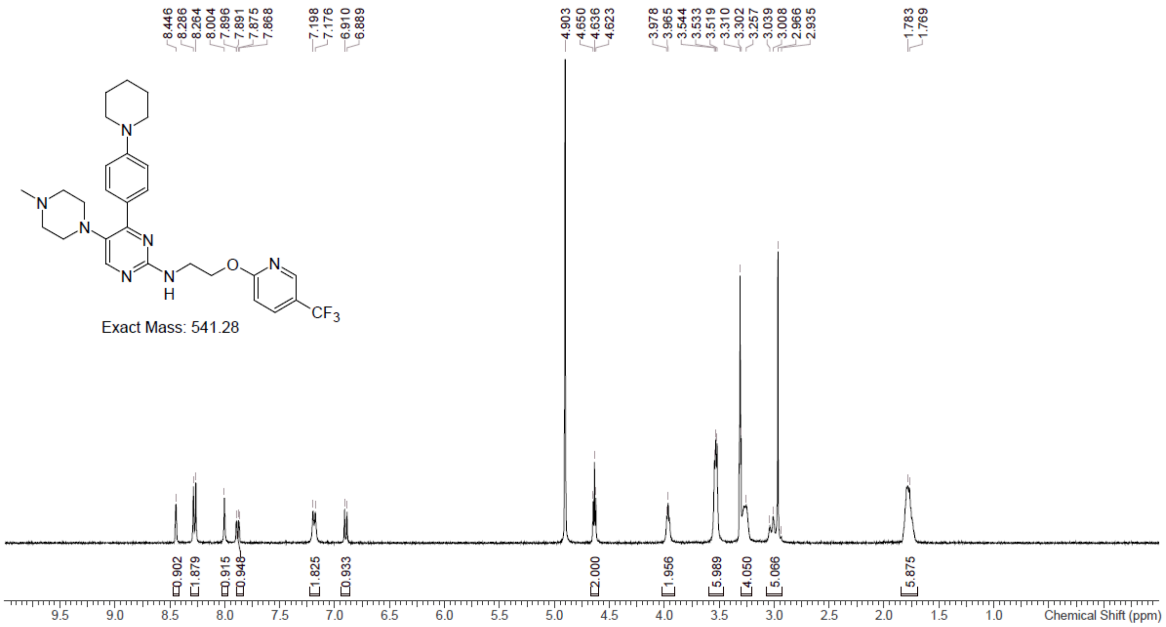

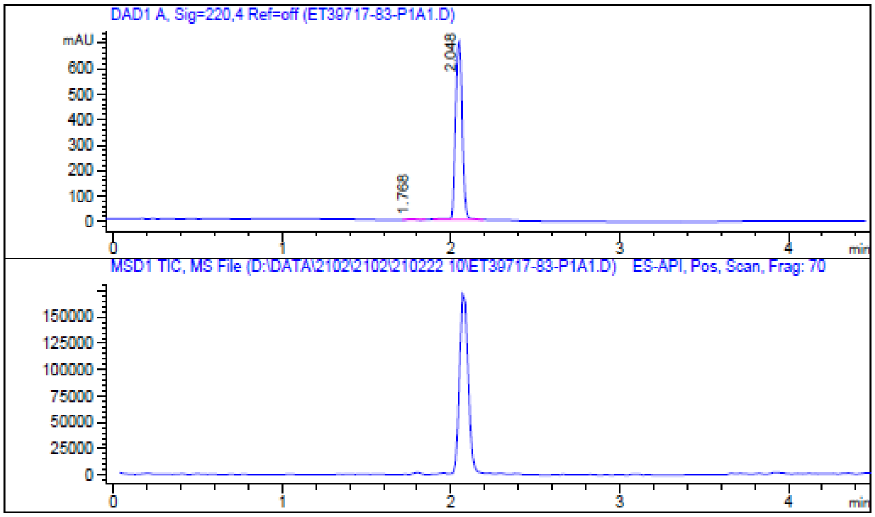

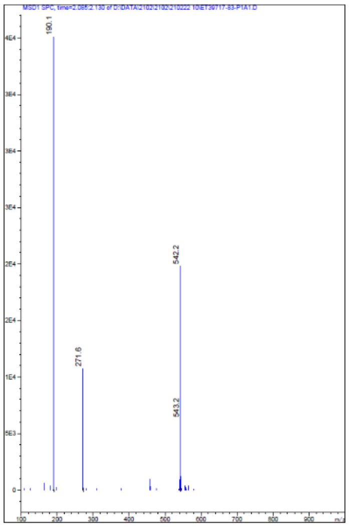

Compound 104

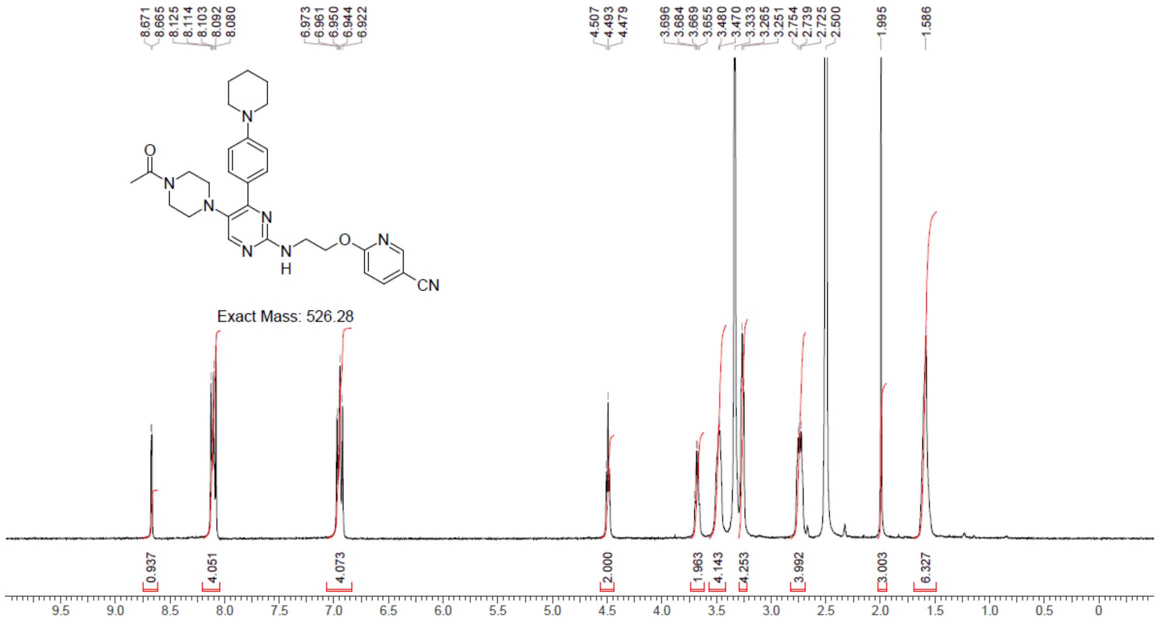

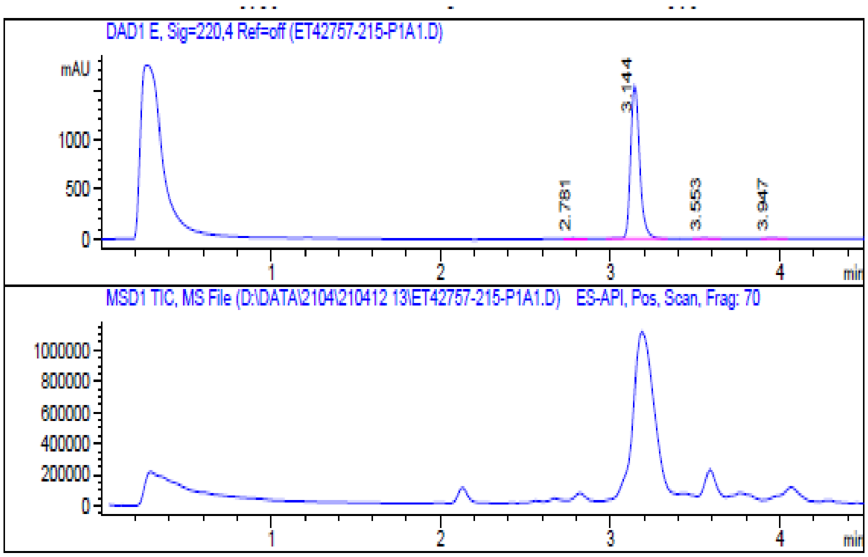

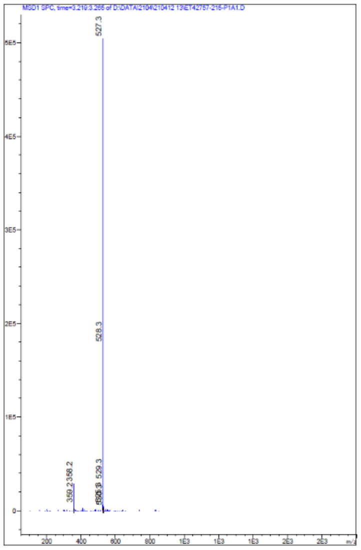

Compound 105

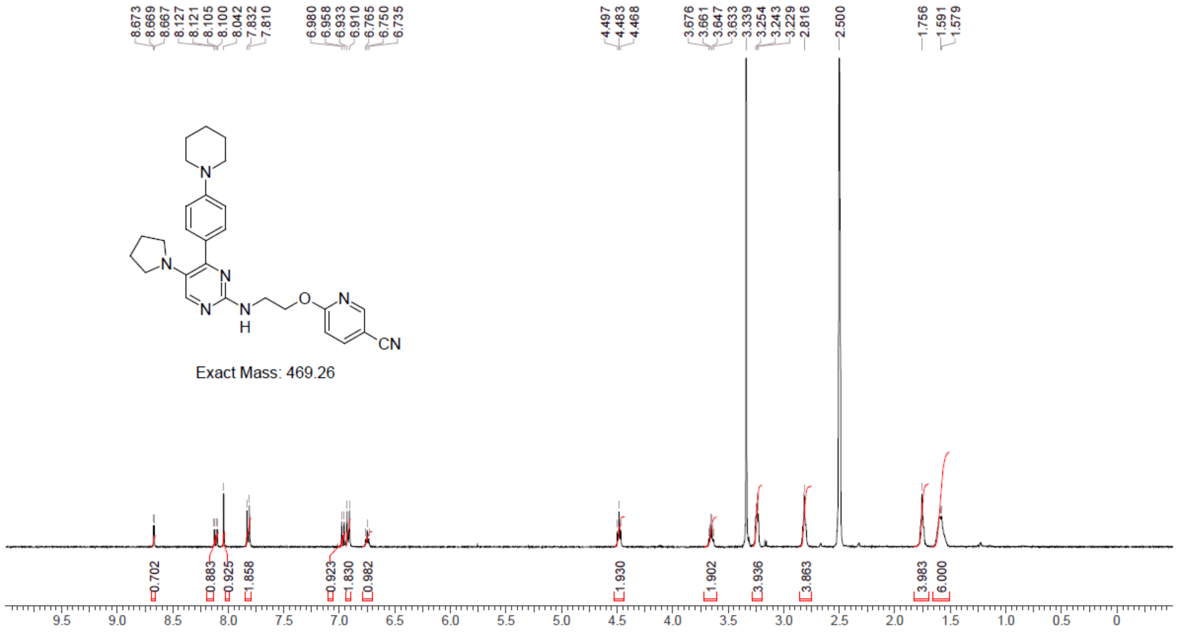

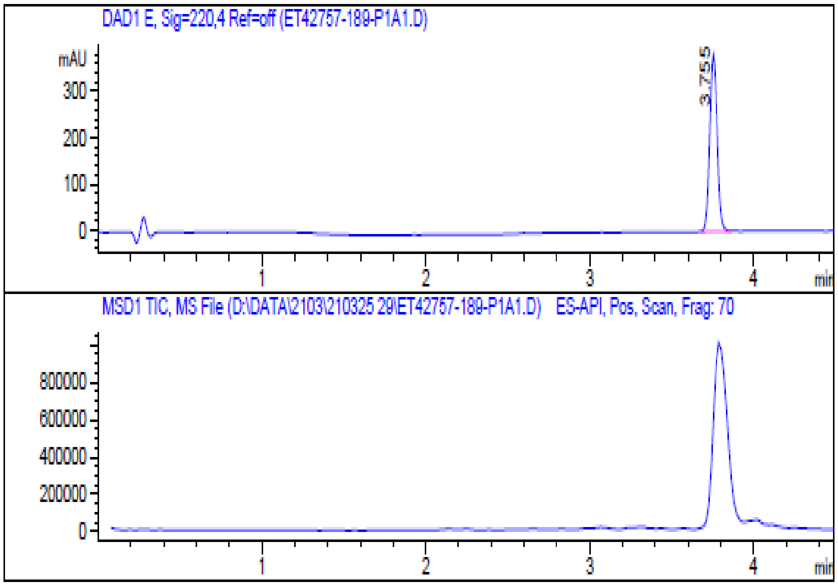

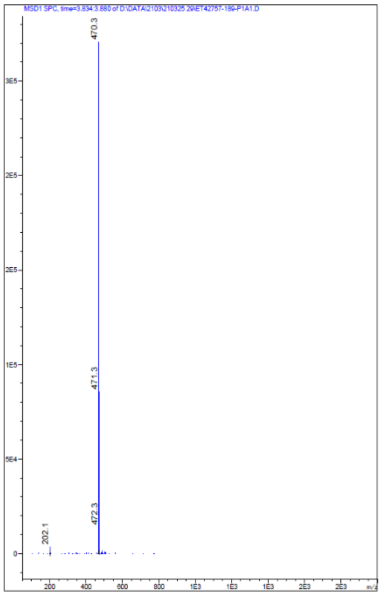

Compound 106

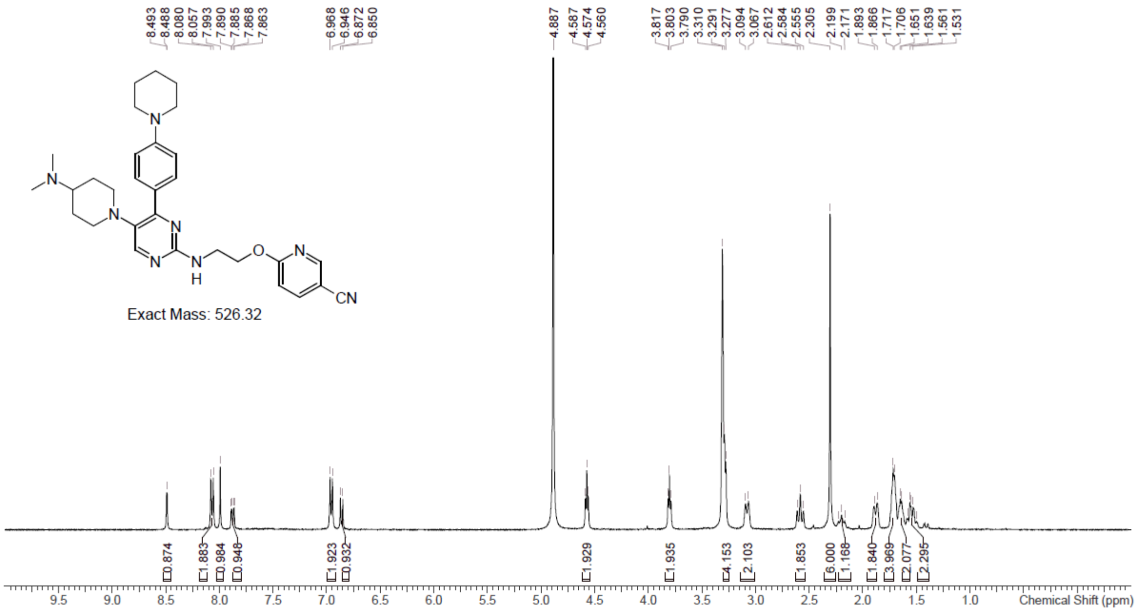

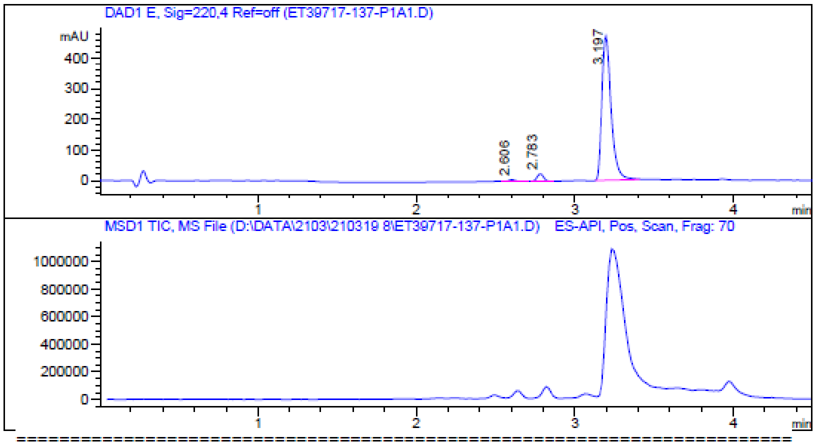

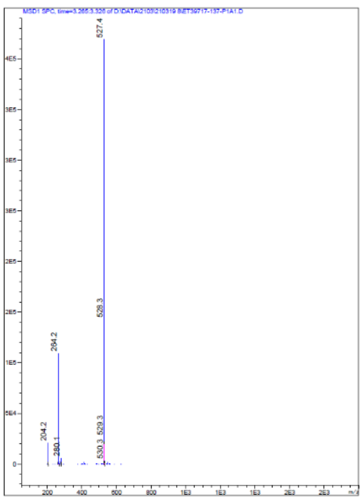

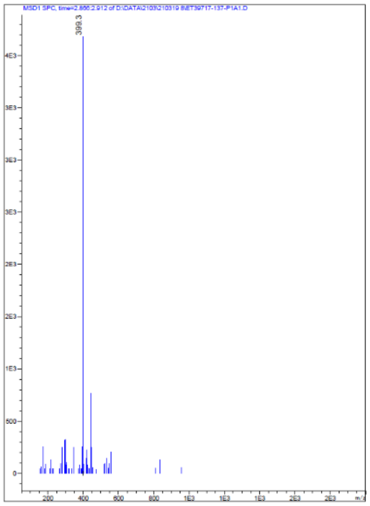

Compound 107

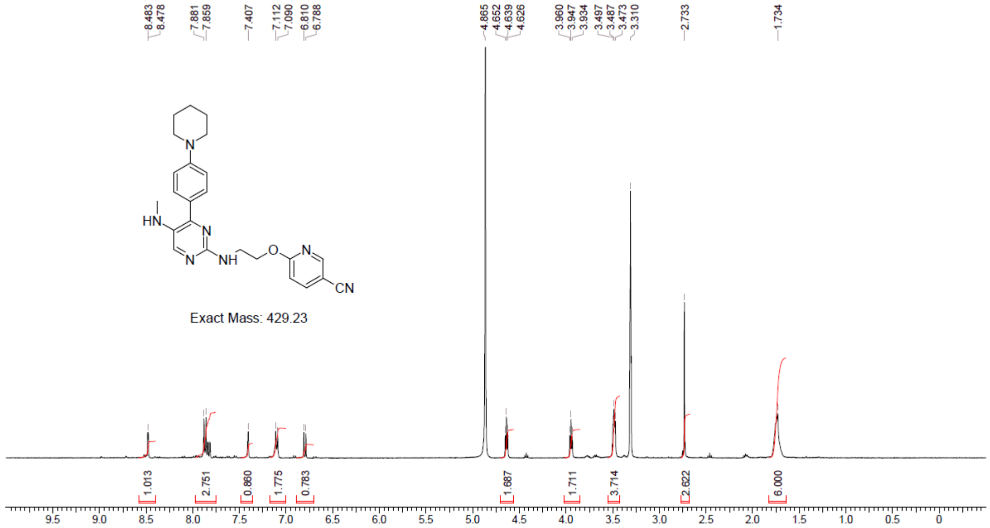

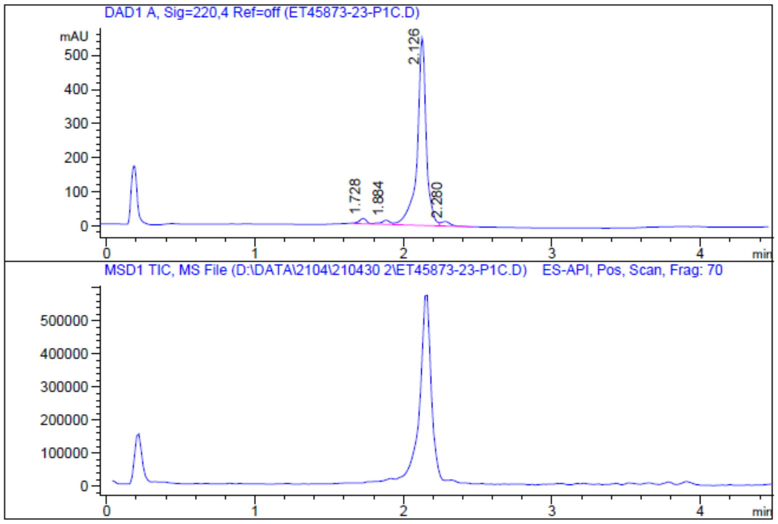

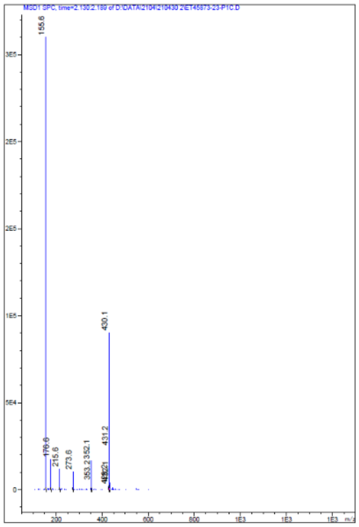

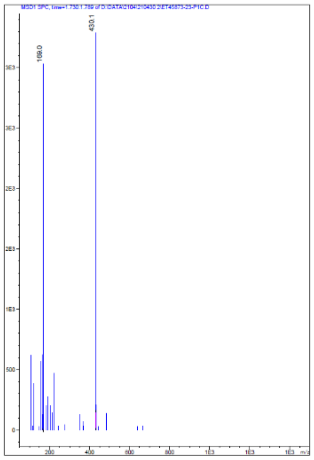

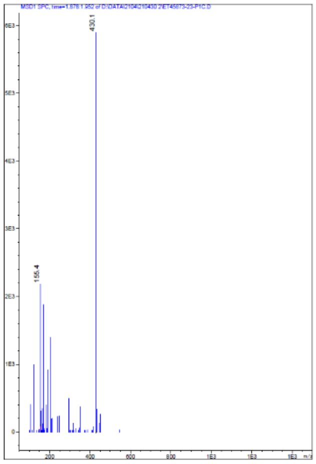

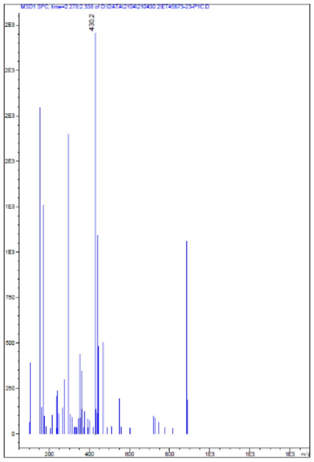

Compound 108

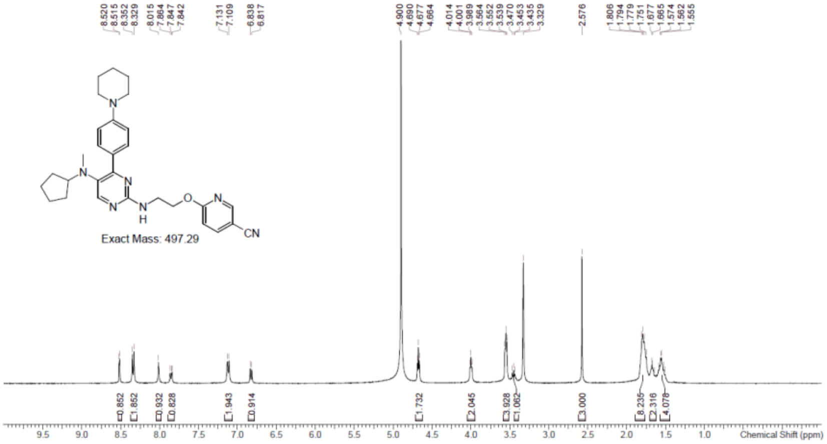

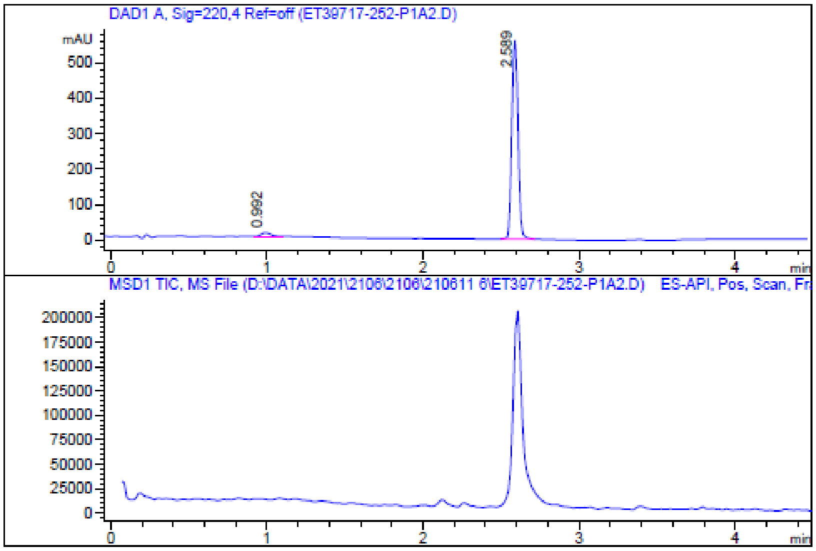

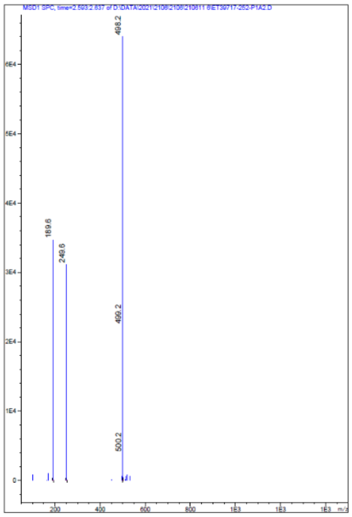

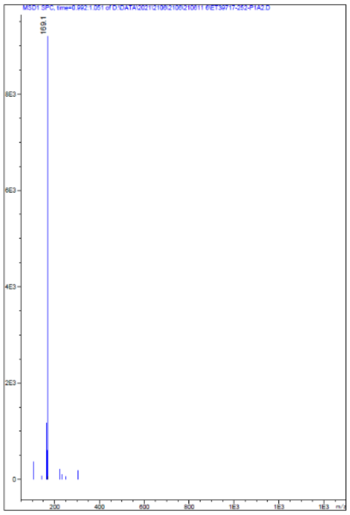

Compound 109

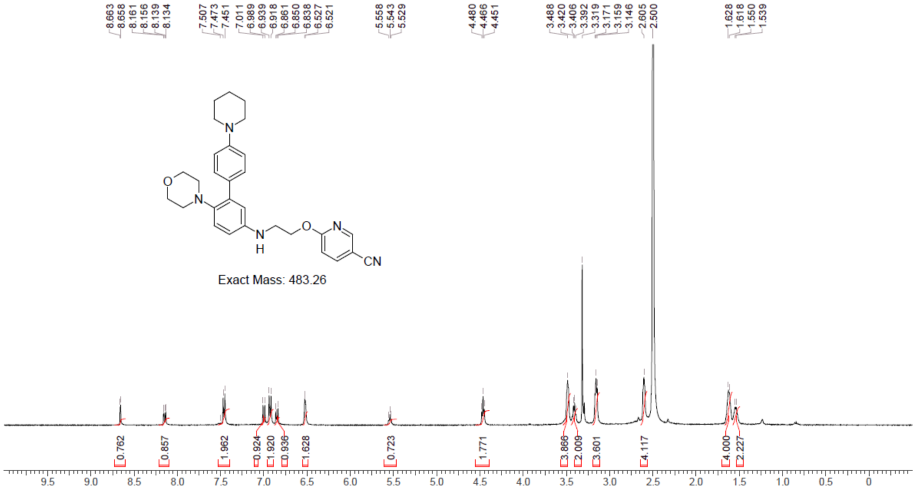

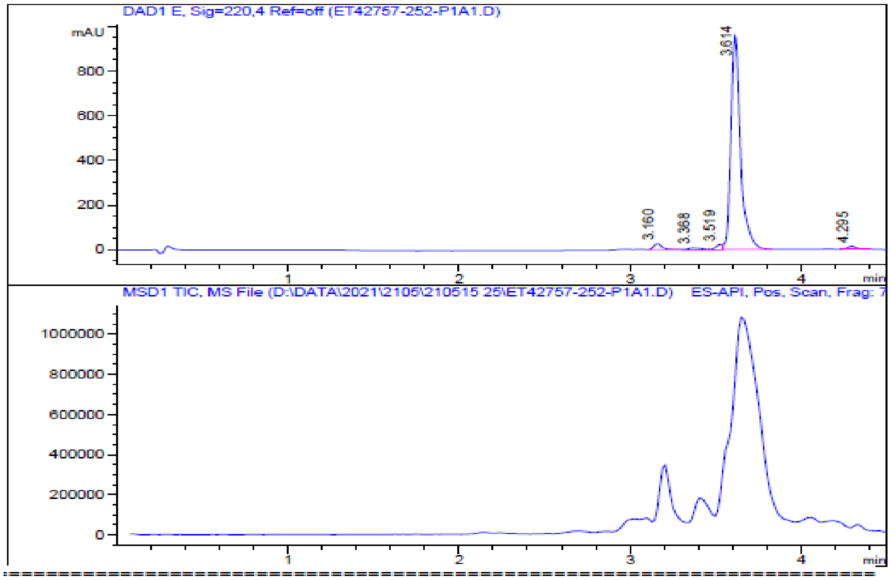

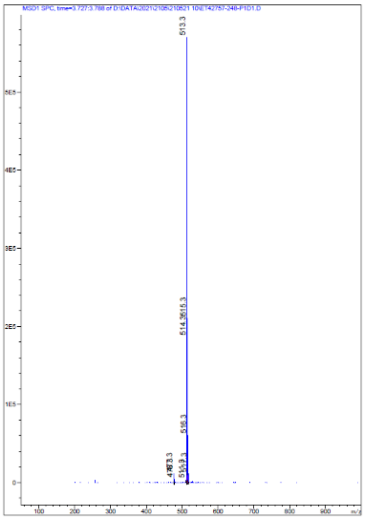

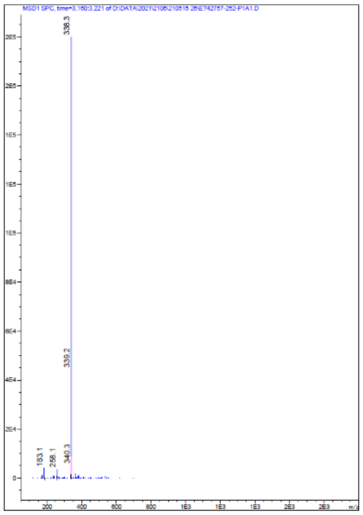

Compound 110

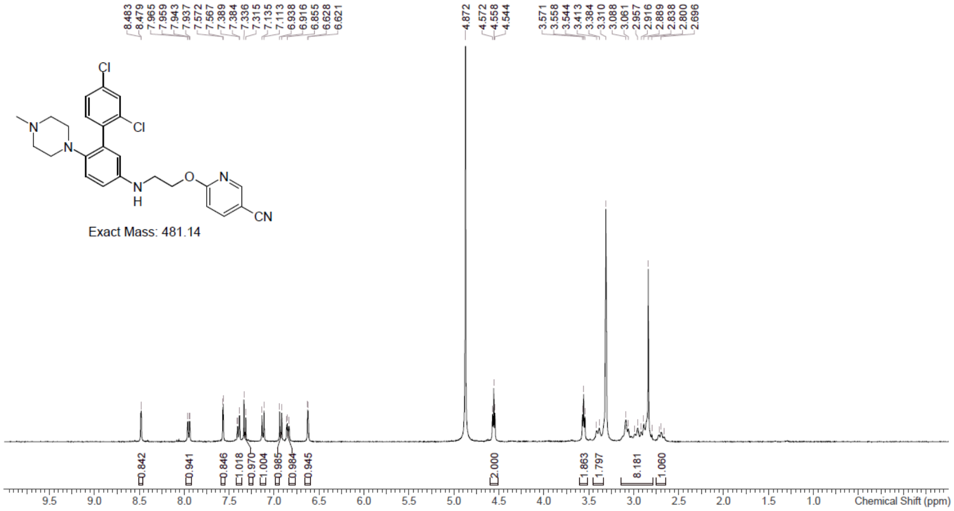

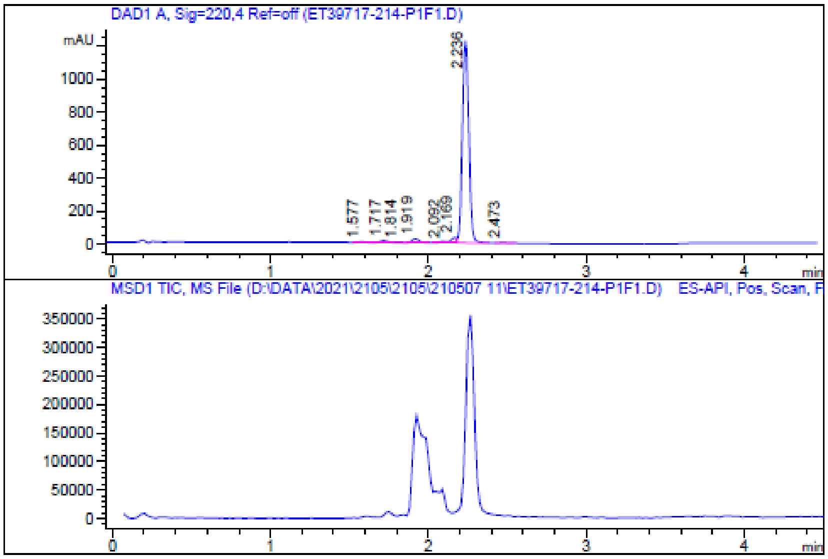

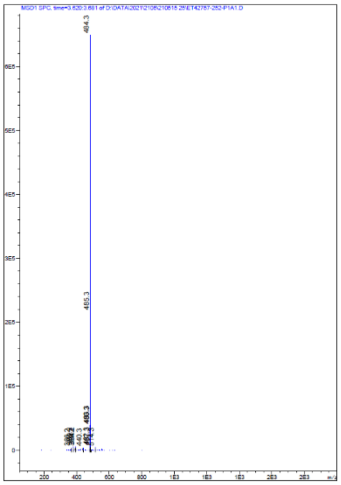

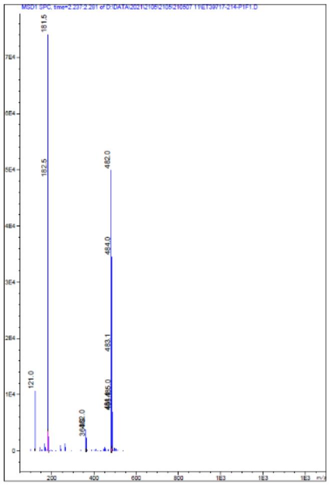

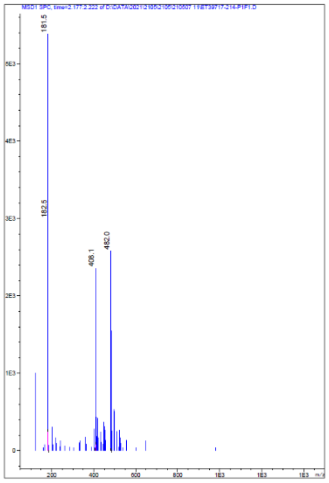

Compound 111

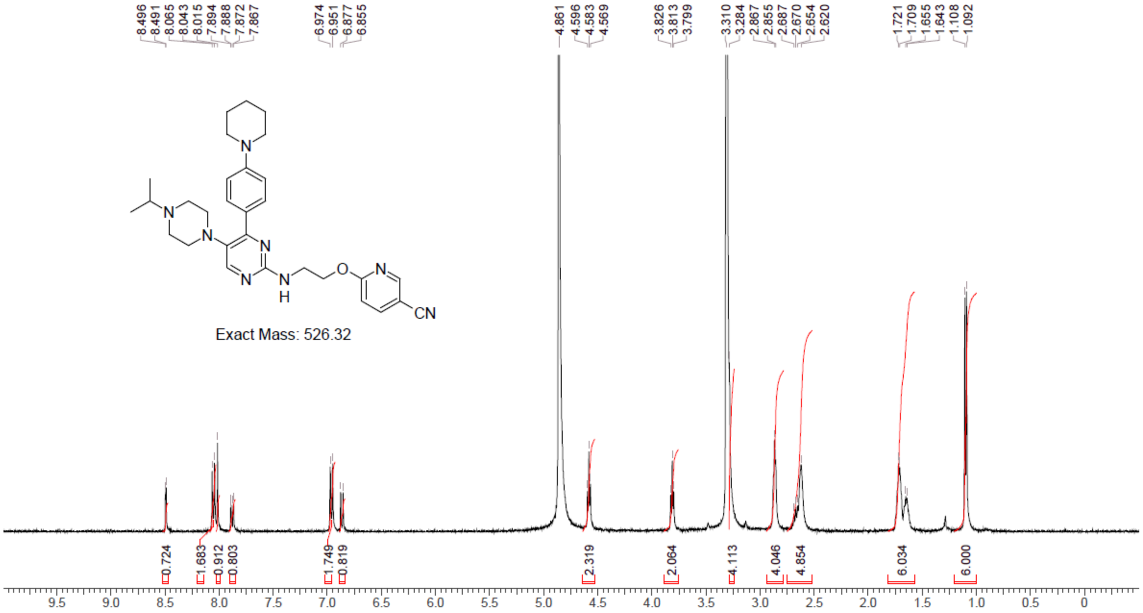

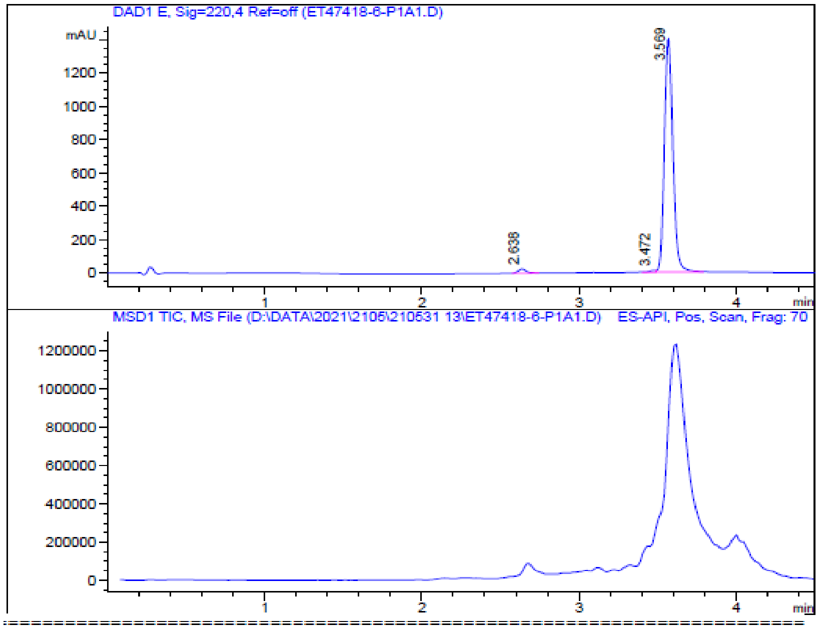

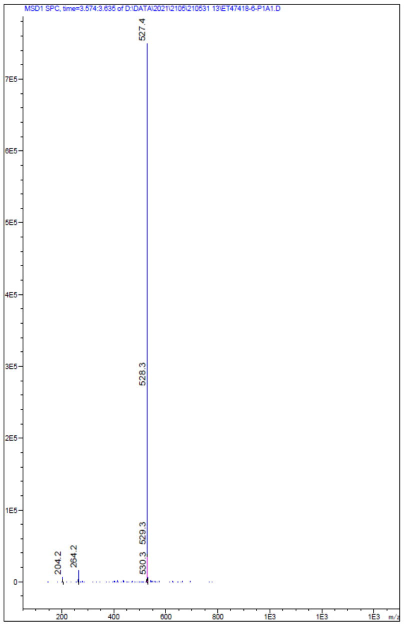

Compound 112

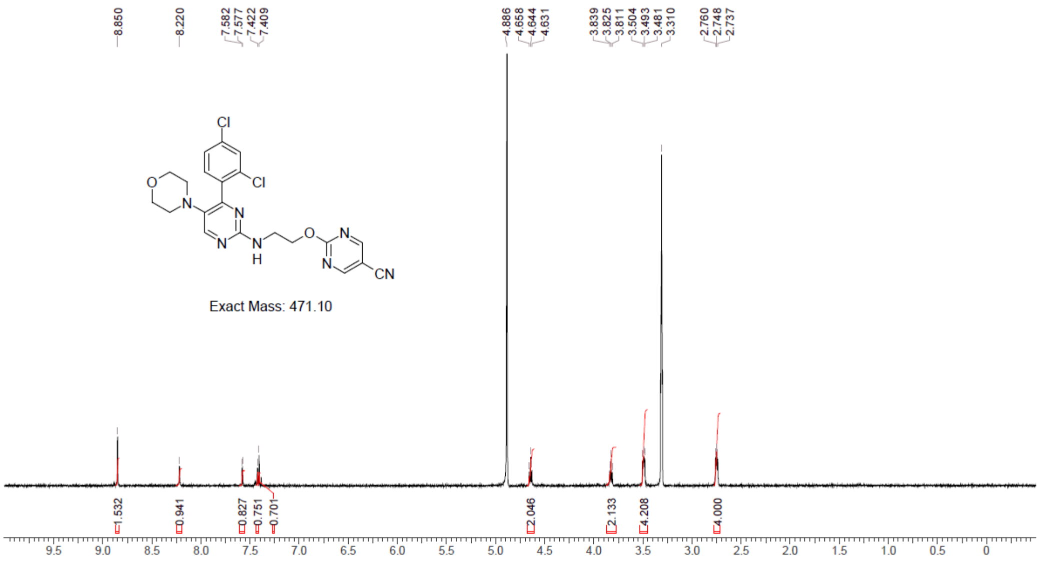

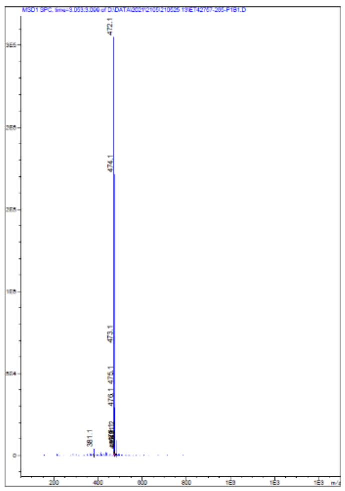

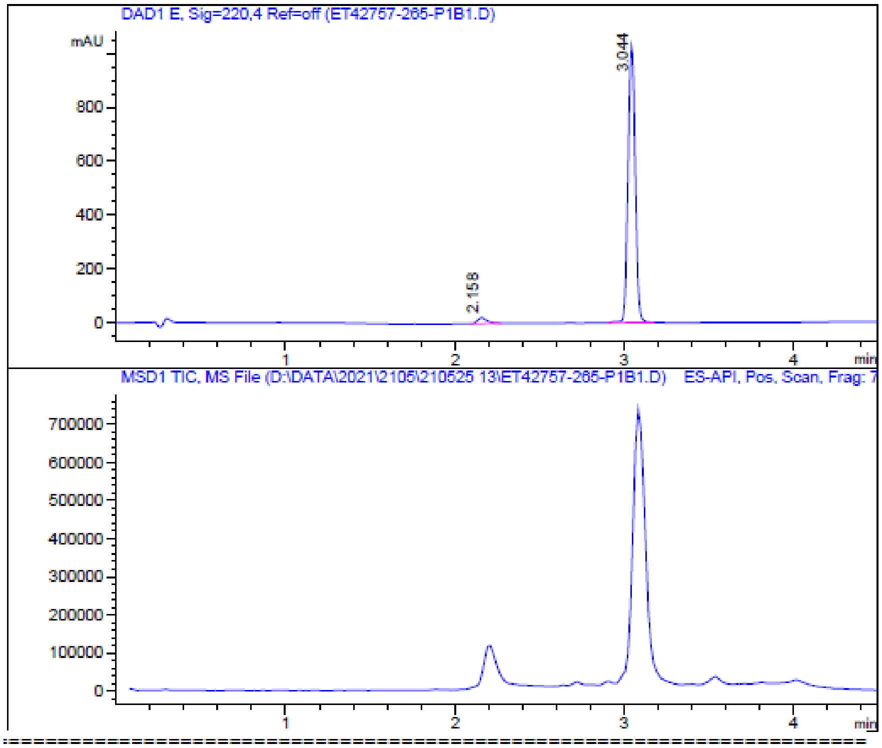

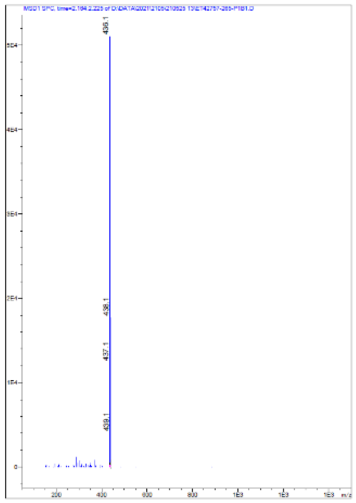

Compound 113

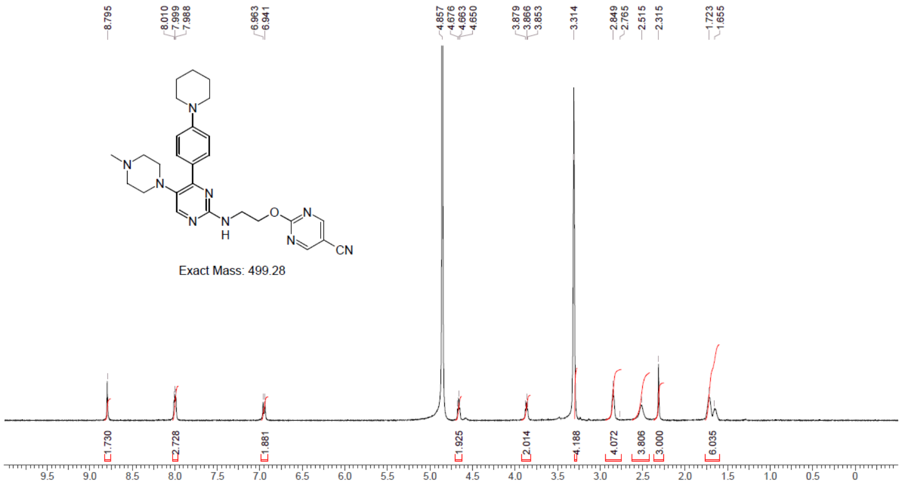

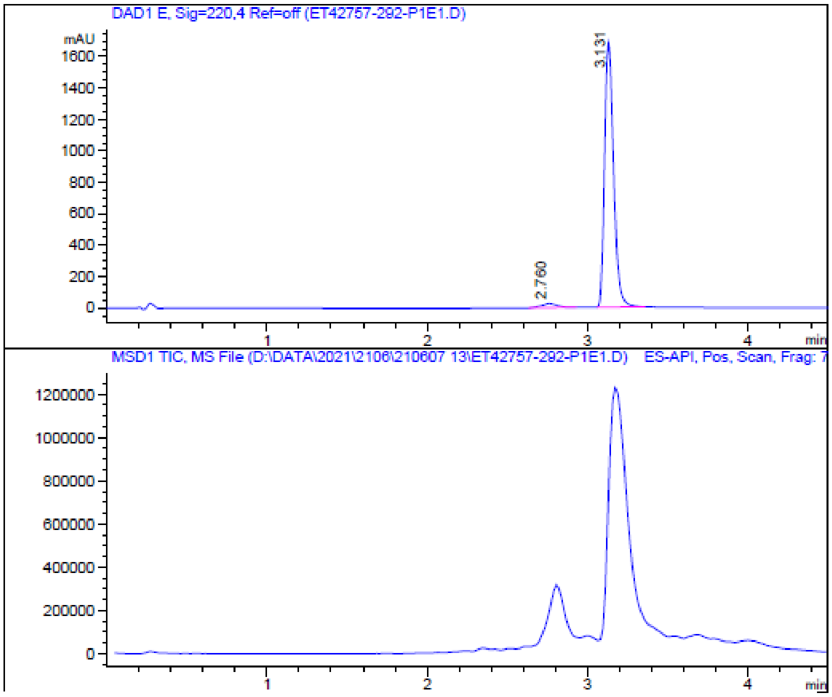

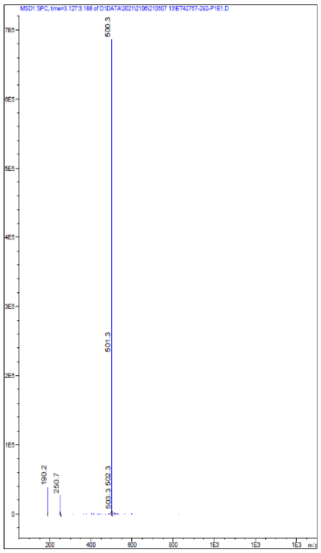

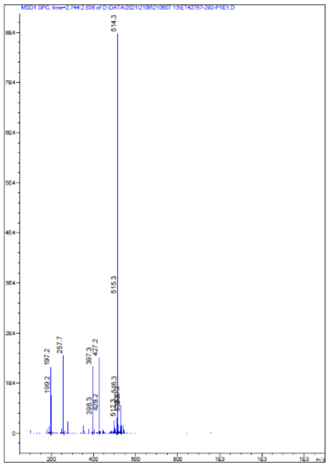

Compound 114

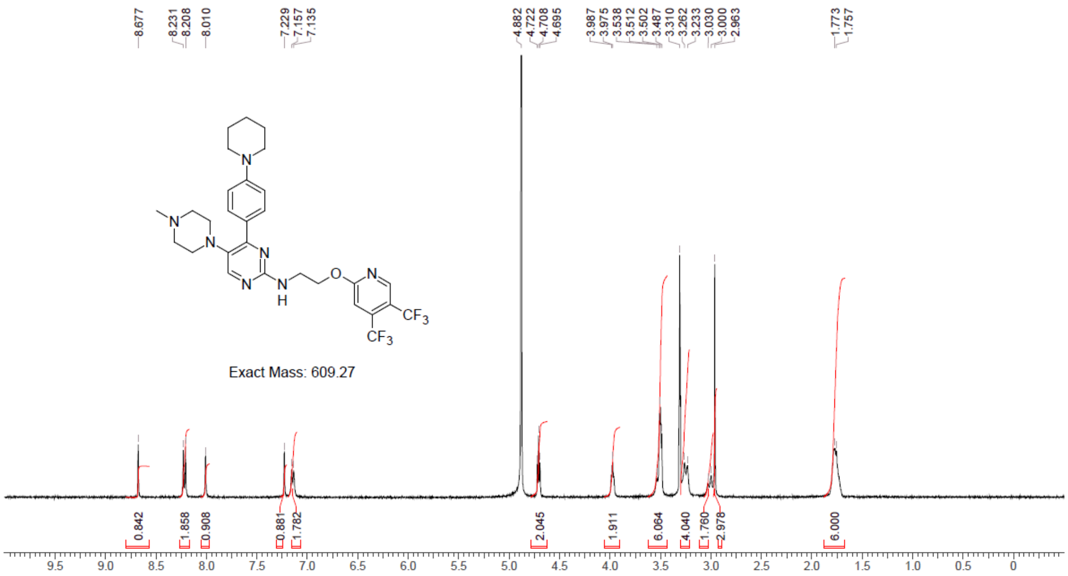

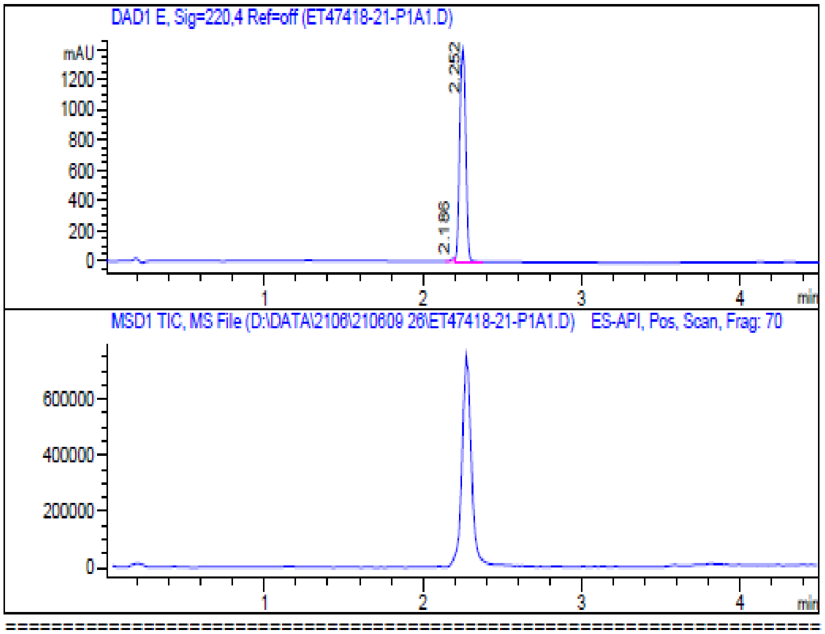

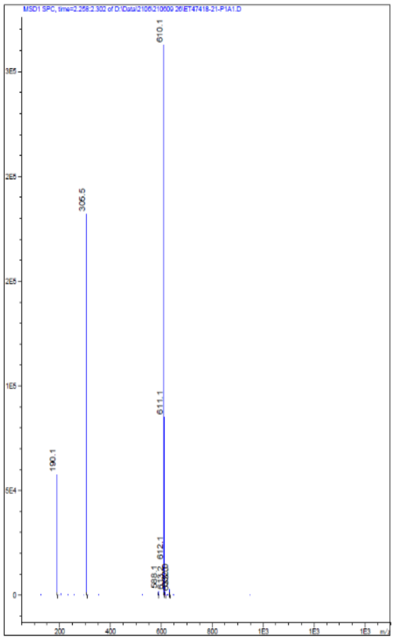

Compound 115

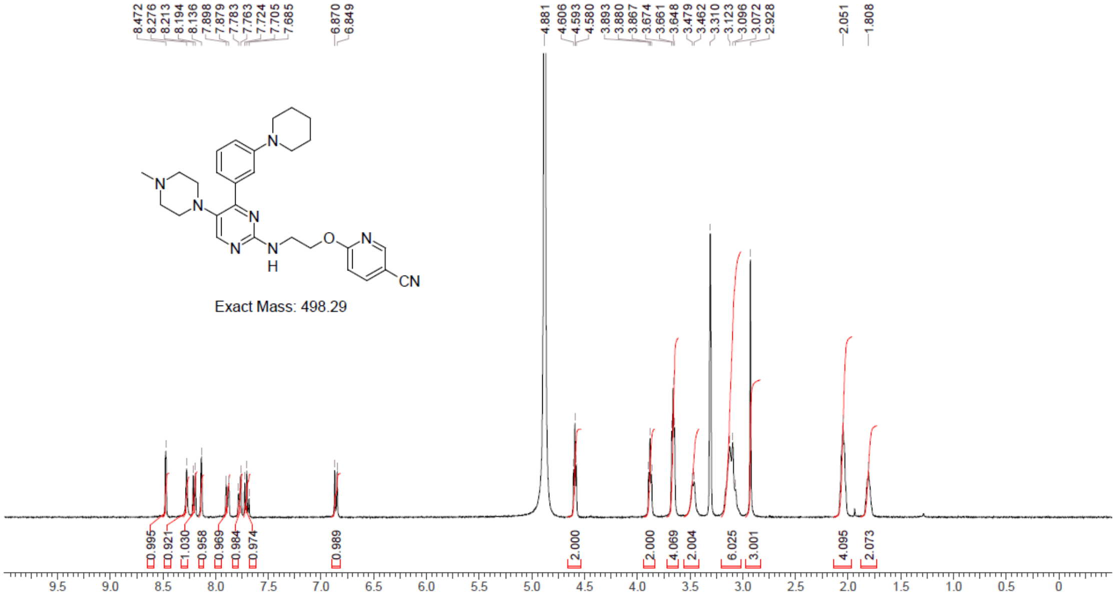

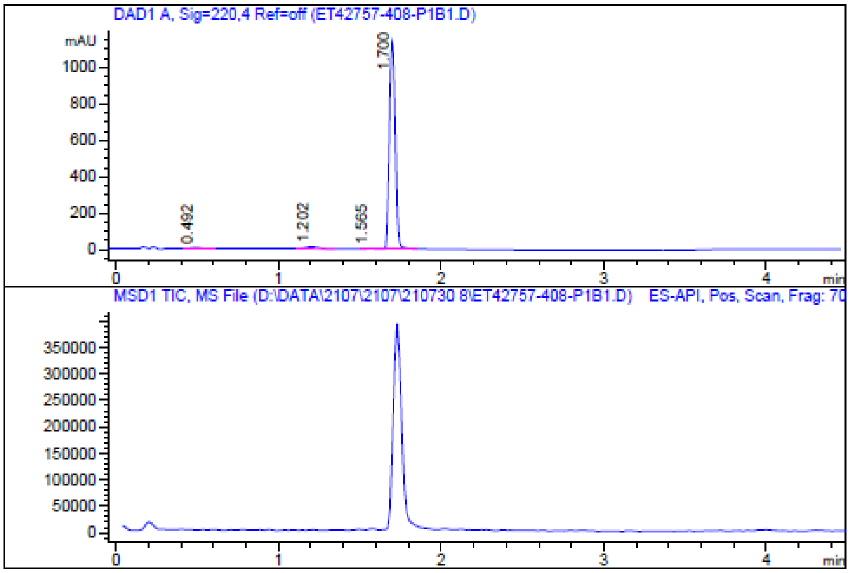

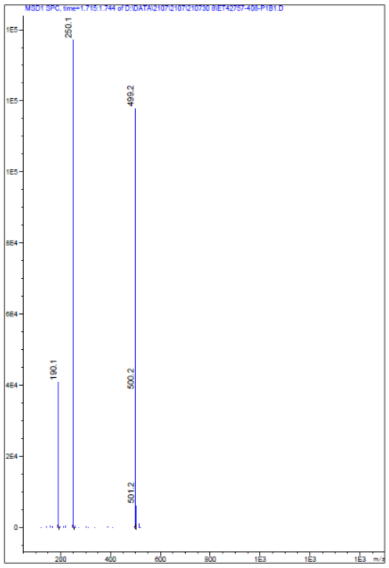

Supporting information 3 – GSK3 CETSA assay for CHIR99021 analogs

Analog 7,11

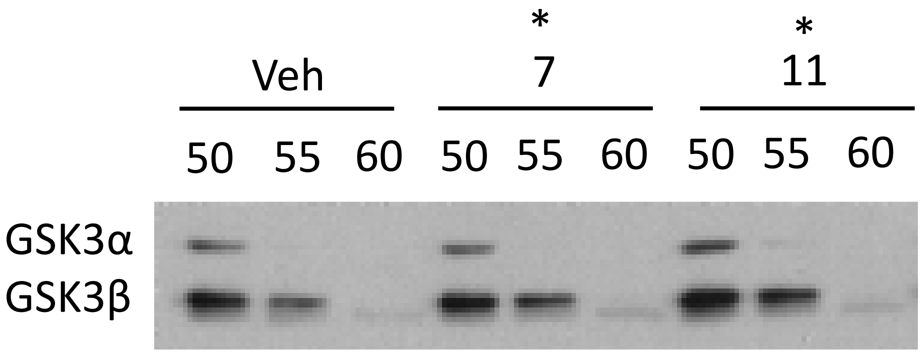

Analog 12, 57

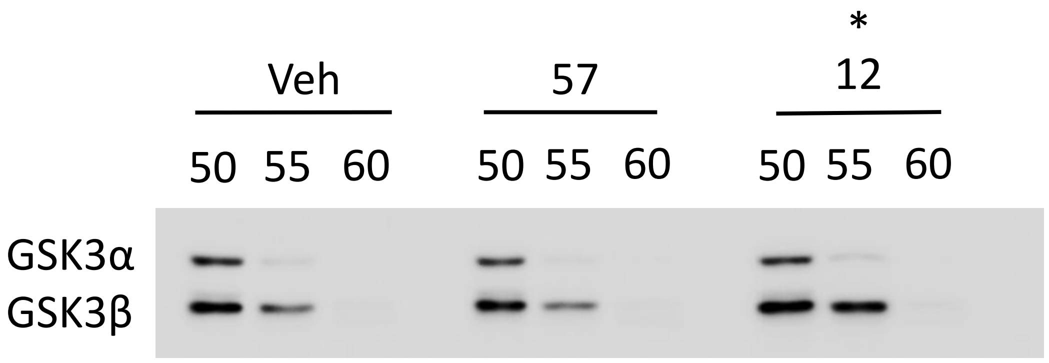

Analog 41

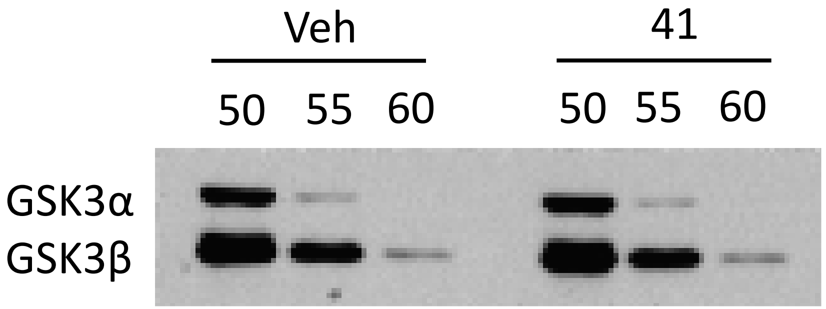

Analog 46

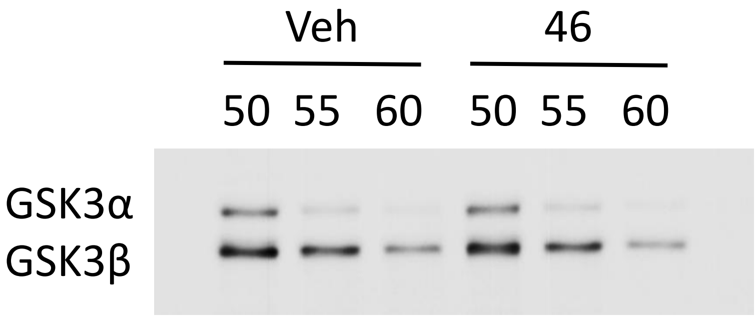

Analog 53

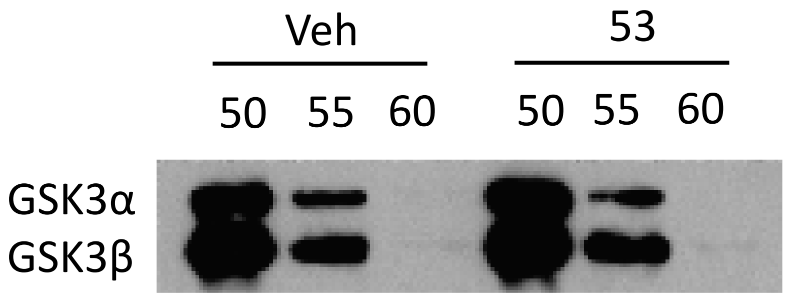

Analog 64

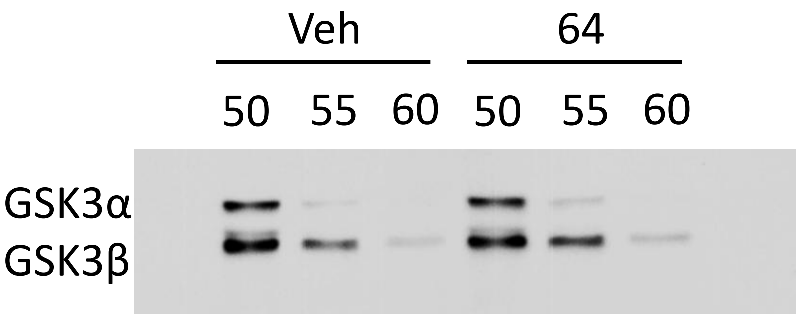

Analog 66

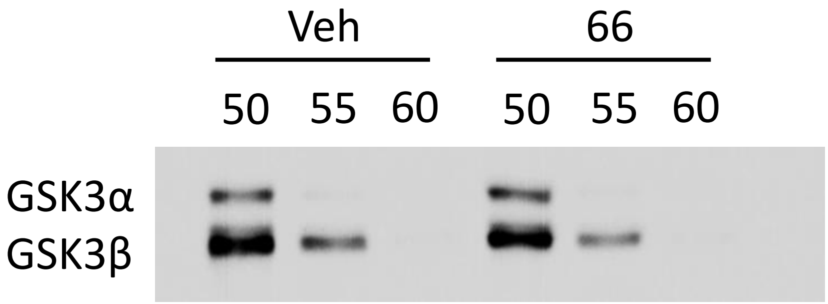

Analog 70

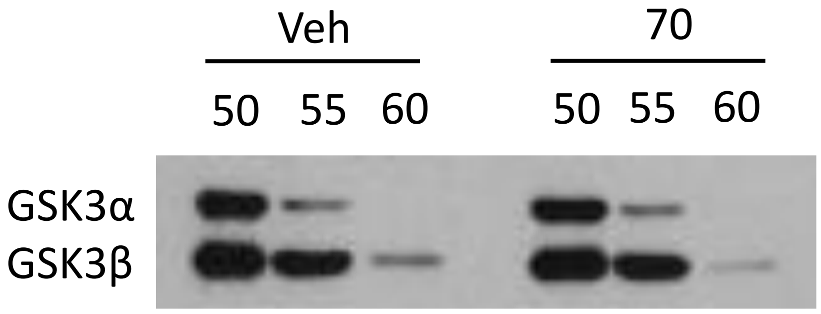

Analog 82

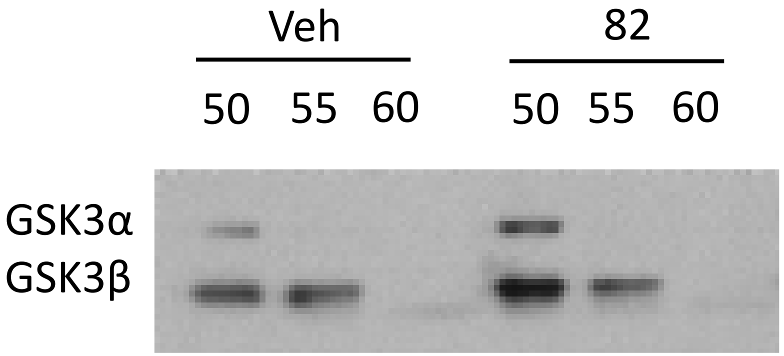

Analog 83

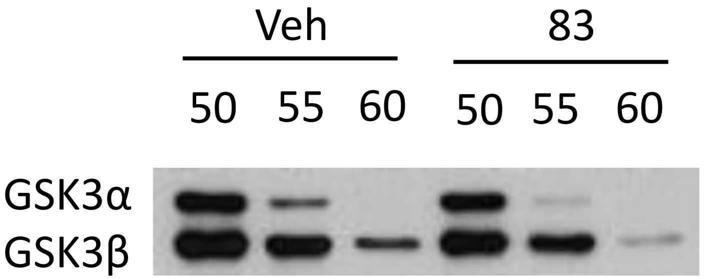

Analog 98, 100, 101

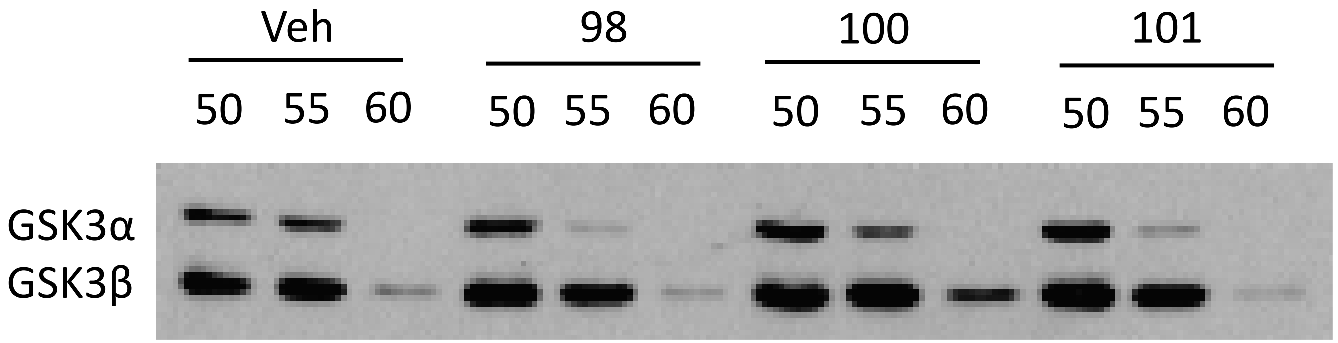

Analog 102, 103

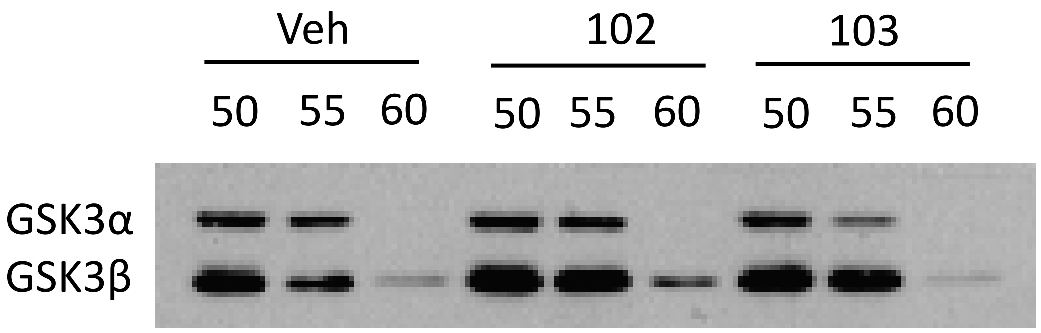

Supporting information 4 - GSK3β enzyme activity *in vitro*

Project #	Compound Name	Kinase Tested	[ATP] Tested (µM)	IC50 (nM)	Hillslope	R^2 Value	Conc of Compound (nM)	% Inhibition Point 1	% Inhibition Point 2	Development Reaction Interference	Test Compound Interference Coumarin	Test Compound Interference Fluorescein	Z'	Kinase Part# / Lot#	Graph	
SSBK11802_58074	CW1	GSK3B (GSK3 beta)	Km app	4.19	0.99	0.9997	10000	99	100	Pass	Pass	Pass	0.95	PV3365/2001427	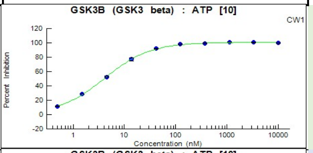	7
SSBK11802_58074	CW1	GSK3B (GSK3 beta)	Km app	4.19	0.99	0.9997	3330	100	100	Pass	Pass	Pass	0.95	PV3365/2001427		
SSBK11802_58074	CW1	GSK3B (GSK3 beta)	Km app	4.19	0.99	0.9997	1110	100	100	Pass	Pass	Pass	0.95	PV3365/2001427		
SSBK11802_58074	CW1	GSK3B (GSK3 beta)	Km app	4.19	0.99	0.9997	370	99	99	Pass	Pass	Pass	0.95	PV3365/2001427		
SSBK11802_58074	CW1	GSK3B (GSK3 beta)	Km app	4.19	0.99	0.9997	123	98	97	Pass	Pass	Pass	0.95	PV3365/2001427		
SSBK11802_58074	CW1	GSK3B (GSK3 beta)	Km app	4.19	0.99	0.9997	41.2	92	91	Pass	Pass	Pass	0.95	PV3365/2001427		
SSBK11802_58074	CW1	GSK3B (GSK3 beta)	Km app	4.19	0.99	0.9997	13.7	74	79	Pass	Pass	Pass	0.95	PV3365/2001427		
SSBK11802_58074	CW1	GSK3B (GSK3 beta)	Km app	4.19	0.99	0.9997	4.57	54	51	Pass	Pass	Pass	0.95	PV3365/2001427		
SSBK11802_58074	CW1	GSK3B (GSK3 beta)	Km app	4.19	0.99	0.9997	1.52	28	29	Pass	Pass	Pass	0.95	PV3365/2001427		
SSBK11802_58074	CW1	GSK3B (GSK3 beta)	Km app	4.19	0.99	0.9997	0.495	11	12	Pass	Pass	Pass	0.95	PV3365/2001427		
SSBK11802_58074	CW2	GSK3B (GSK3 beta)	Km app	696	0.89	0.9994	10000	89	91	Pass	Pass	Pass	0.95	PV3365/2001427	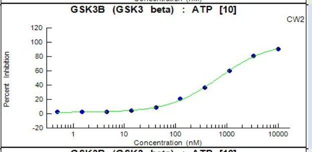	11
SSBK11802_58074	CW2	GSK3B (GSK3 beta)	Km app	696	0.89	0.9994	3330	80	81	Pass	Pass	Pass	0.95	PV3365/2001427		
SSBK11802_58074	CW2	GSK3B (GSK3 beta)	Km app	696	0.89	0.9994	1110	59	61	Pass	Pass	Pass	0.95	PV3365/2001427		
SSBK11802_58074	CW2	GSK3B (GSK3 beta)	Km app	696	0.89	0.9994	370	36	35	Pass	Pass	Pass	0.95	PV3365/2001427		
SSBK11802_58074	CW2	GSK3B (GSK3 beta)	Km app	696	0.89	0.9994	123	20	20	Pass	Pass	Pass	0.95	PV3365/2001427		
SSBK11802_58074	CW2	GSK3B (GSK3 beta)	Km app	696	0.89	0.9994	41.2	9	8	Pass	Pass	Pass	0.95	PV3365/2001427		
SSBK11802_58074	CW2	GSK3B (GSK3 beta)	Km app	696	0.89	0.9994	13.7	4	3	Pass	Pass	Pass	0.95	PV3365/2001427		
SSBK11802_58074	CW2	GSK3B (GSK3 beta)	Km app	696	0.89	0.9994	4.57	3	2	Pass	Pass	Pass	0.95	PV3365/2001427		
SSBK11802_58074	CW2	GSK3B (GSK3 beta)	Km app	696	0.89	0.9994	1.52	2	2	Pass	Pass	Pass	0.95	PV3365/2001427		
SSBK11802_58074	CW2	GSK3B (GSK3 beta)	Km app	696	0.89	0.9994	0.495	2	2	Pass	Pass	Pass	0.95	PV3365/2001427		
SSBK11802_58074	CW3	GSK3B (GSK3 beta)	Km app	>10000	-0.12	0.5903	10000	3	6	Pass	Pass	Pass	0.95	PV3365/2001427	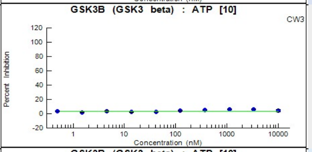	53
SSBK11802_58074	CW3	GSK3B (GSK3 beta)	Km app	>10000	-0.12	0.5903	3330	6	5	Pass	Pass	Pass	0.95	PV3365/2001427		
SSBK11802_58074	CW3	GSK3B (GSK3 beta)	Km app	>10000	-0.12	0.5903	1110	6	5	Pass	Pass	Pass	0.95	PV3365/2001427		
SSBK11802_58074	CW3	GSK3B (GSK3 beta)	Km app	>10000	-0.12	0.5903	370	5	5	Pass	Pass	Pass	0.95	PV3365/2001427		
SSBK11802_58074	CW3	GSK3B (GSK3 beta)	Km app	>10000	-0.12	0.5903	123	4	3	Pass	Pass	Pass	0.95	PV3365/2001427		
SSBK11802_58074	CW3	GSK3B (GSK3 beta)	Km app	>10000	-0.12	0.5903	41.2	2	2	Pass	Pass	Pass	0.95	PV3365/2001427		
SSBK11802_58074	CW3	GSK3B (GSK3 beta)	Km app	>10000	-0.12	0.5903	13.7	2	2	Pass	Pass	Pass	0.95	PV3365/2001427		
SSBK11802_58074	CW3	GSK3B (GSK3 beta)	Km app	>10000	-0.12	0.5903	4.57	3	4	Pass	Pass	Pass	0.95	PV3365/2001427		
SSBK11802_58074	CW3	GSK3B (GSK3 beta)	Km app	>10000	-0.12	0.5903	1.52	2	1	Pass	Pass	Pass	0.95	PV3365/2001427		
SSBK11802_58074	CW3	GSK3B (GSK3 beta)	Km app	>10000	-0.12	0.5903	0.495	3	3	Pass	Pass	Pass	0.95	PV3365/2001427		
SSBK11802_58074	CW4	GSK3B (GSK3 beta)	Km app	>10000	0.04	0.8158	10000	5	8	Pass	Pass	Pass	0.95	PV3365/2001427	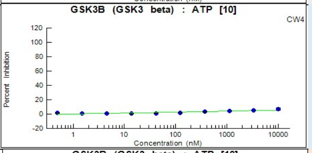	54
SSBK11802_58074	CW4	GSK3B (GSK3 beta)	Km app	>10000	0.04	0.8158	3330	5	4	Pass	Pass	Pass	0.95	PV3365/2001427		
SSBK11802_58074	CW4	GSK3B (GSK3 beta)	Km app	>10000	0.04	0.8158	1110	4	4	Pass	Pass	Pass	0.95	PV3365/2001427		
SSBK11802_58074	CW4	GSK3B (GSK3 beta)	Km app	>10000	0.04	0.8158	370	3	2	Pass	Pass	Pass	0.95	PV3365/2001427		
SSBK11802_58074	CW4	GSK3B (GSK3 beta)	Km app	>10000	0.04	0.8158	123	2	1	Pass	Pass	Pass	0.95	PV3365/2001427		
SSBK11802_58074	CW4	GSK3B (GSK3 beta)	Km app	>10000	0.04	0.8158	41.2	1	0	Pass	Pass	Pass	0.95	PV3365/2001427		
SSBK11802_58074	CW4	GSK3B (GSK3 beta)	Km app	>10000	0.04	0.8158	13.7	0	1	Pass	Pass	Pass	0.95	PV3365/2001427		
SSBK11802_58074	CW4	GSK3B (GSK3 beta)	Km app	>10000	0.04	0.8158	4.57	1	0	Pass	Pass	Pass	0.95	PV3365/2001427		
SSBK11802_58074	CW4	GSK3B (GSK3 beta)	Km app	>10000	0.04	0.8158	1.52	0	1	Pass	Pass	Pass	0.95	PV3365/2001427		
SSBK11802_58074	CW4	GSK3B (GSK3 beta)	Km app	>10000	0.04	0.8158	0.495	0	2	Pass	Pass	Pass	0.95	PV3365/2001427		
SSBK11802_58074	CW6	GSK3B (GSK3 beta)	Km app	>10000	0.66	0.9913	10000	19	29	Pass	Pass	Pass	0.95	PV3365/2001427	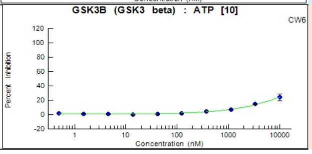	82
SSBK11802_58074	CW6	GSK3B (GSK3 beta)	Km app	>10000	0.66	0.9913	3330	15	13	Pass	Pass	Pass	0.95	PV3365/2001427		
SSBK11802_58074	CW6	GSK3B (GSK3 beta)	Km app	>10000	0.66	0.9913	1110	7	7	Pass	Pass	Pass	0.95	PV3365/2001427		
SSBK11802_58074	CW6	GSK3B (GSK3 beta)	Km app	>10000	0.66	0.9913	370	3	4	Pass	Pass	Pass	0.95	PV3365/2001427		
SSBK11802_58074	CW6	GSK3B (GSK3 beta)	Km app	>10000	0.66	0.9913	123	1	1	Pass	Pass	Pass	0.95	PV3365/2001427		
SSBK11802_58074	CW6	GSK3B (GSK3 beta)	Km app	>10000	0.66	0.9913	41.2	1	-1	Pass	Pass	Pass	0.95	PV3365/2001427		
SSBK11802_58074	CW6	GSK3B (GSK3 beta)	Km app	>10000	0.66	0.9913	13.7	-1	0	Pass	Pass	Pass	0.95	PV3365/2001427		
SSBK11802_58074	CW6	GSK3B (GSK3 beta)	Km app	>10000	0.66	0.9913	4.57	0	0	Pass	Pass	Pass	0.95	PV3365/2001427		
SSBK11802_58074	CW6	GSK3B (GSK3 beta)	Km app	>10000	0.66	0.9913	1.52	1	0	Pass	Pass	Pass	0.95	PV3365/2001427		
SSBK11802_58074	CW6	GSK3B (GSK3 beta)	Km app	>10000	0.66	0.9913	0.495	1	2	Pass	Pass	Pass	0.95	PV3365/2001427		
